# Dual catalytic enantioselective desymmetrization of allene-tethered cyclohexanones†

**DOI:** 10.1039/d0sc02878a

**Published:** 2020-06-24

**Authors:** Lin Zhang, Ken Yamazaki, Jamie A. Leitch, Ruben Manzano, Victoria A. M. Atkinson, Trevor A. Hamlin, Darren J. Dixon

**Affiliations:** Department of Chemistry, Chemistry Research Laboratory, University of Oxford 12 Mansfield Road Oxford UK; Department of Theoretical Chemistry, Amsterdam Institute of Molecular and Life Sciences (AIMMS), Amsterdam Center for Multiscale Modeling (ACMM), Vrije Universiteit Amsterdam De Boelelaan 1083 1081 HV Amsterdam The Netherlands t.a.hamlin@vu.nl

## Abstract

The construction of enantioenriched azabicyclo[3.3.1]nonan-6-one heterocycles *via* an enantioselective desymmetrization of allene-linked cyclohexanones, enabled through a dual catalytic system, that provides synchronous activation of the cyclohexanone with a chiral prolinamide and the allene with a copper(i) co-catalyst to deliver the stereodefined bicyclic core, is described. Successful application to oxygen analogues was also achieved, thereby providing a new enantioselective synthetic entry to architecturally complex bicyclic ethereal frameworks. The mechanistic pathway and the origin of enantio- and diastereoselectivities has been uncovered using density functional theory (DFT) calculations.

## Abstract 2

The construction of enantioenriched azabicyclo[3.3.1]nonan-6-one heterocycles *via* an enantioselective desymmetrization of allene-linked cyclohexanones, enabled through a dual prolinamide/copper(i) catalytic system, is described.

## Graphical abstract

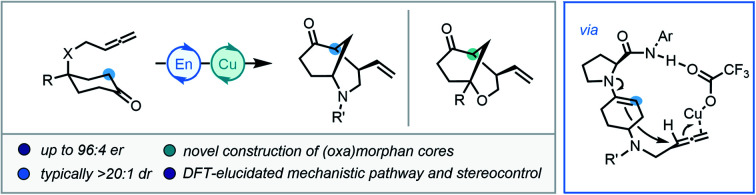

## Introduction

**Scheme 1:**
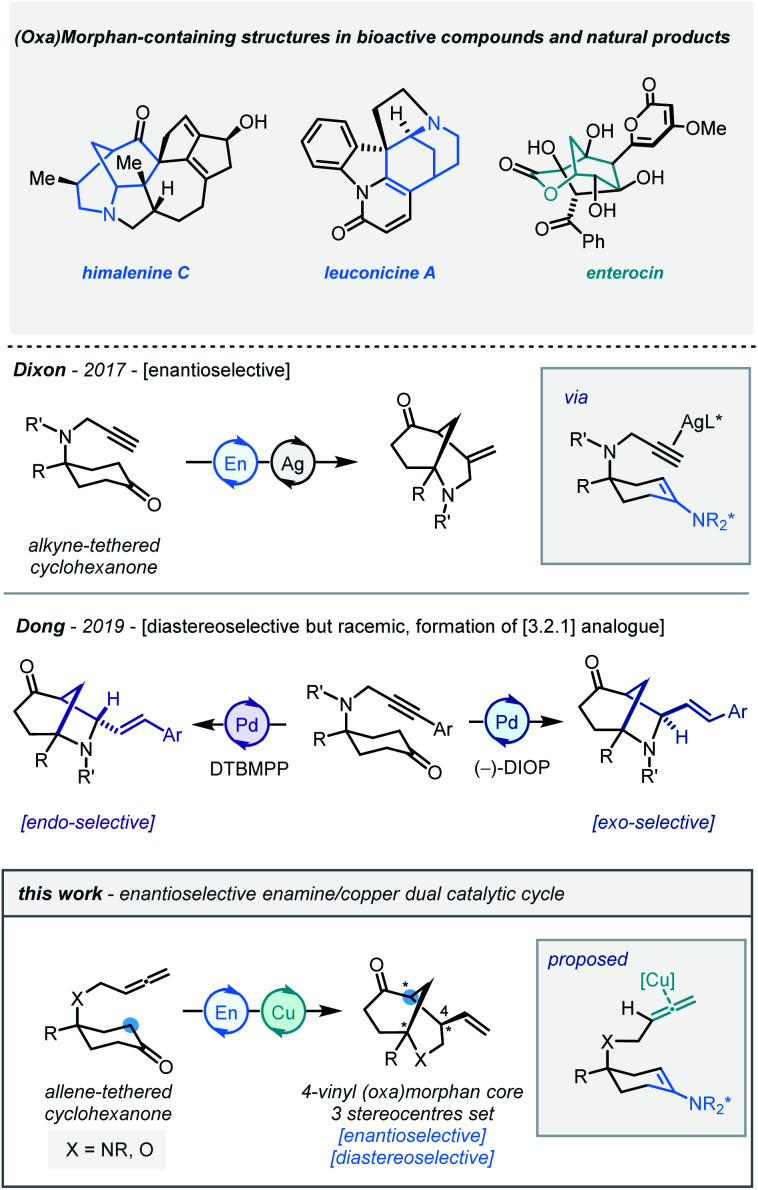
The design of an allene-linked cyclohexanone desymmetrization platform. En = enamine catalysis.

The morphan (azabicyclo[3.3.1]nonane) scaffold is a common and versatile subunit to many bioactive compounds, and serves as the core to a variety of natural products (Scheme 1), including himalenine C, daphniyunnine-type alkaloids, strychnine, and immunosuppressant FR901483.^1^ Moreover, oxygenated analogues are also found in natural products such as enterocin.^2^ Due to these viable biologically relevant applications, and often deficient quantities available from the natural source,^3^ the demand for efficient and stereocontrolled syntheses of these complex architectures has been a focus of research efforts in enantioselective synthesis in recent years.^4^

The enantioselective desymmetrization of structurally simple molecules is widely considered as one of the most powerful and elegant strategies to create new stereocenters.^5^ In this context, the desymmetrization of carbonyl compounds through intramolecular cyclization manifolds has proven to be an excellent access point to bridged and fused bicyclic rings. Through elegant contributions from the Jia,^6^ Lu,^7^ and Zhou^8^ groups amongst many others – including our own^9^ – significant progress has been made in this field. In 2017, our group demonstrated that the silver and chiral amine co-catalyzed intramolecular desymmetrization/cyclization of alkyne-linked cyclohexanones afforded the morphan core with high enantioselectivity.^10^ Recently, Dong and co-workers described an intramolecular Pd-catalyzed α-allylic alkylation of alkyne-linked cyclohexanone derivatives, notably with the ability to access both *exo* and *endo*-diastereomers of the [3.2.1] morphan analogue, albeit with insignificant enantioinduction^11^ (Scheme 1, middle). For downstream application in complex molecule total synthesis, we were drawn towards developing a new desymmetrization methodology for the construction of the 4-vinyl-morphan and their analogous oxygenated architectures. This would require the simultaneous stereocontrolled creation of three new stereogenic centres in a bicyclic core possessing multiple key groups and functionalities.

To date, only a handful of examples of enantioselective intramolecular cyclization of allene-linked carbonyl compounds have been reported.^12^ Inspired by this prior art, and building on our own findings, we reasoned that a suitable dual catalyst system could be identified to furnish such target morphan structures. We envisioned that an intramolecular cyclization of allene-tethered cyclohexanones could take place *via* enamine catalysis and allene activation by an appropriate metal catalyst (Scheme 1, bottom), and herein we wish to report our findings.

## Results & discussion

Our studies began using the nitrogen-linked allene-tethered cyclohexan-4-one derivative (**1a**) as a model substrate. Following preliminary co-catalyst studies, a promising copper-based system was identified where a prolinamide derivative (P1, 30 mol%), Cu(OTf)_2_ (10 mol%), 4-bromobenzoic acid (50 mol%) system in CPME at 120 °C, delivered the bicyclic product (**2a**) in a good yield (76%) and enantioselectivity (82 : 18 er, Table 1, entry 1). A survey of copper(i) and copper(ii) salts which have previously found use in allene activation^13^ (entries 2–6) demonstrated that the copper(i) complex Cu(MeCN)_4_PF_6_ (entry 4) performed most effectively, delivering the morphan architecture in almost quantitative yield and 87 : 13 er. Following this, a study of prolinamide catalysts (entries 7–10) identified that P4 improved the enantioselectivity of the transformation to 89.5 : 10.5 er and diastereoselectivity to >20 : 1 dr (entry 9). It was also found that reducing the reaction temperature to 100 °C extinguished reactivity (entry 11). Furthermore, diluting the reaction mixture was found to be beneficial to enantioselectivity (entries 12–13). A short re-investigation of the prolinamide catalyst at this point revealed that 2-naphthylprolinamide derivative (P6) outperformed all other catalysts, and therefore was adopted as the catalyst of choice (entry 14). Finally, an exchange of the acidic additive from 4-bromobenzoic acid to trifluoroacetic acid delivered a substantial increase in enantioselectivity to 96 : 4 er.

**Table 1:** Optimization of the dual catalytic enantioselective desymmetrization of allene-tethered cyclohexanones^a^

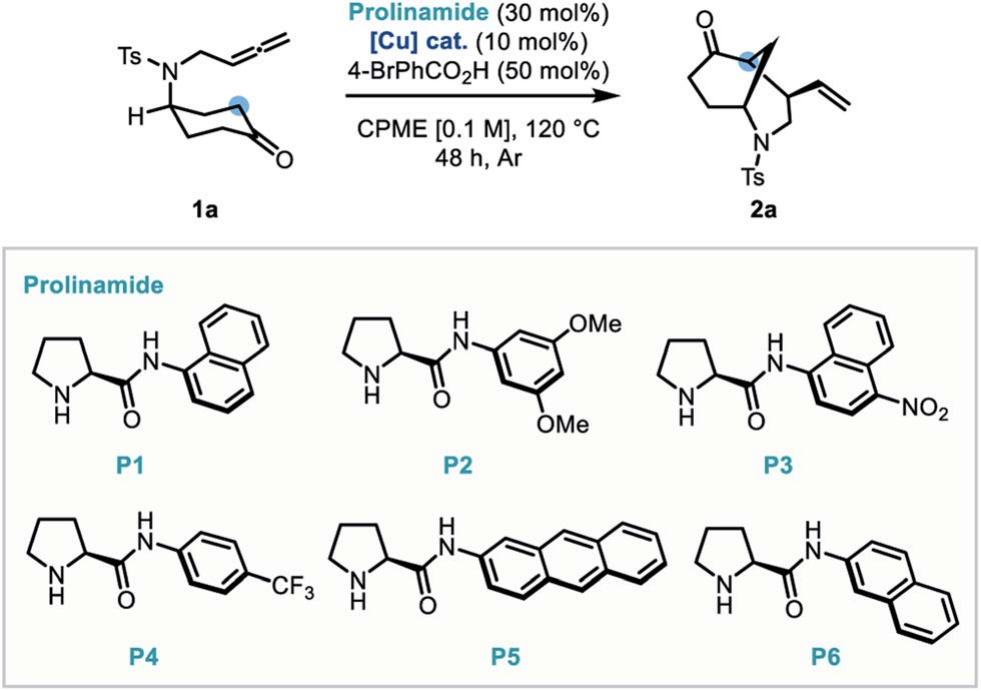					
Entry	[Cu] cat.	Prolinamide	**2a** b (%)	dr^c^	er^d^
1	Cu(OTf)2	P1	76	8 : 1	82 : 18
2	CuI	P1	44	>20 : 1	72 : 28
3	Cu(OAc)_2_	P1	40	7 : 1	70 : 30
4	Cu(MeCN)_4_PF_6_	P1	98	12 : 1	87 : 13
5	Cu(MeCN)_4_BF_4_	P1	76	11 : 1	78 : 22
6	Cu(acac)_2_	P1	93	10 : 1	80 : 20
7	Cu(MeCN)_4_PF_6_	P2	99	>20 : 1	82 : 18
8	Cu(MeCN)_4_PF_6_	P3	90	18 : 1	80 : 20
9	Cu(MeCN)_4_PF_6_	P4	99	>20 : 1	89.5 : 10:5
10	Cu(MeCN)_4_PF_6_	P5	90	18 : 1	89 : 11

**Change from entry 9**					
11	Reaction run at 100 °C		—	nd	nd
12	Reaction concentration [0.04 M]		91	>20 : 1	90 : 10
13	Reaction concentration [0.02 M]		93	>20 : 1	91 : 9
14^e^	P6 used as organocatalyst		82	>20 : 1	92.5 : 7.5
**15** e ^,^ f	**TFA used instead of 4-BrPhCO**_**2**_ **H**		**81**	**>20** **:** **1**	**96** **:** **4**

a General conditions: **1a** (0.2 mmol), [Cu] catalyst (0.02 mmol, 10 mol%), 4-bromobenzoic acid (0.1 mmol, 50 mol%), prolinamide catalyst (0.06 mmol, 30 mol%), in CPME (0.1 M, 2 mL) at 120 °C, under an argon atmosphere for 48 h.

b Isolated yield.

c dr calculated *via*^1^H NMR analysis of the crude reaction mixture.

d er value was determined *via* chiral HPLC analysis of the pure product.

e Reaction concentration [0.02 M].

f P6 (30 mol%) was used as prolinamide catalyst.

**Scheme 2:**
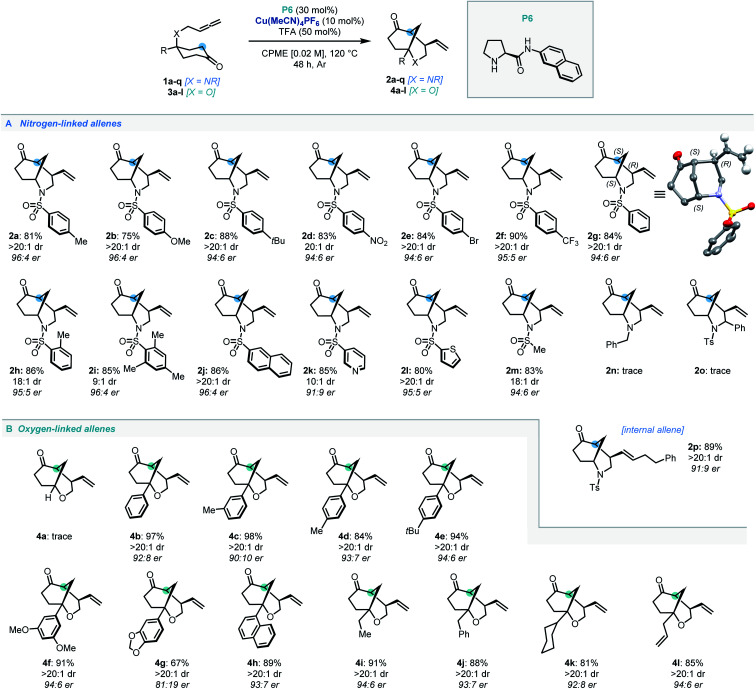
Substrate scope for the dual catalytic desymmetrization of allene-tethered cyclohexanones.

With the optimal conditions established, the scope of *N*-tethered substrates was explored (Scheme 2). Aryl sulfonamide-tethered allenic cyclohexanones with either electron-donating or electron-withdrawing groups at the *para*-position of the arene delivered morphan products in good yields with high enantioselectivity and diastereoselectivity (**2b–2f**, 94 : 6 to 96 : 4 er). Phenyl substituted compound **1g** afforded **2g** in 78% yield and 94 : 6 er. Importantly the absolute configuration of **2g** was confirmed as 1*S*,4*R*,5*S* by single-crystal X-ray diffraction analysis.^14^*Ortho*-methylated and multiply-substituted arene substrates were also well-tolerated, albeit with a slight drop in diastereoselectivity (**2h** and **2i**). Naphthyl derivative **1j** reacted smoothly to afford **2j** in 86% yield with 96 : 4 er. Substrates bearing heteroaromatic 3-pyridinyl (**1k**) and 2-thiophenyl (**1l**) substituents led to products **2k** and **2l** with great efficiency. Pleasingly, *N*-mesyl protected (**1m**) amines afforded the morphan bicycle with high enantioselectivity (94 : 6 er). Unfortunately, no product was observed when phenyl-substituted derivative (**1o**) was subjected to the optimized conditions, most likely due to excessive steric hindrance preventing the allene access to the enamine intermediate of the ketone moiety. Notably, the internal allene structure **1p** also reacted smoothly under the optimized conditions, providing **2p** in 89% yield with 91 : 9 er.

Following the success of the newly discovered dual catalytic system for the desymmetrization of nitrogen-linked allenes, our attention turned to their oxygen-substituted counterparts to construct the analogous 2-oxabicyclo[3.3.1]nonane heterocycle (oxamorphan for simplicity, herein). To the best of our knowledge, no enantioselective syntheses of such constructs *via* desymmetrization approaches or other stereoselective methodologies have been reported to date.^15^ Accordingly, application of this dual catalytic protocol would find added value in the synthesis of complex oxygen-containing heterocyclic constructs.^2^ Despite our first attempted application of the dual catalytic methodology to the cyclization of an oxygen-linked substrate (**3a**) being unsuccessful, installation of substitution at the α-position to the oxygen atom recovered reactivity, delivering the phenyl-substituted oxamorphan derivative (**4b**) as a single diastereomer in excellent yield and good enantioselectivity (92 : 8 er).^16^ All further examples of the oxamorphan core were also shown to proceed with exquisite diastereoselectivity (>20 : 1 dr). Substitution on the aromatic ring was shown to have little deleterious effect on the reactivity of substrates (**4c–4f**, 84–98% yields and 90 : 10 to 94 : 6 er). Use of a protected catechol-derived substrate, however, led to a decrease in both the yield and enantioselectivity (**4g**, 67% yield and 81 : 19 er). Pleasingly, structures with an alkyl group (ethyl, benzyl and cyclohexyl) functioned efficiently, affording **4i–4k** in high yields with good enantioselectivity. Moreover, allyl substitution was well-tolerated in this desymmetrization methodology (**4l**).

**Scheme 3:**
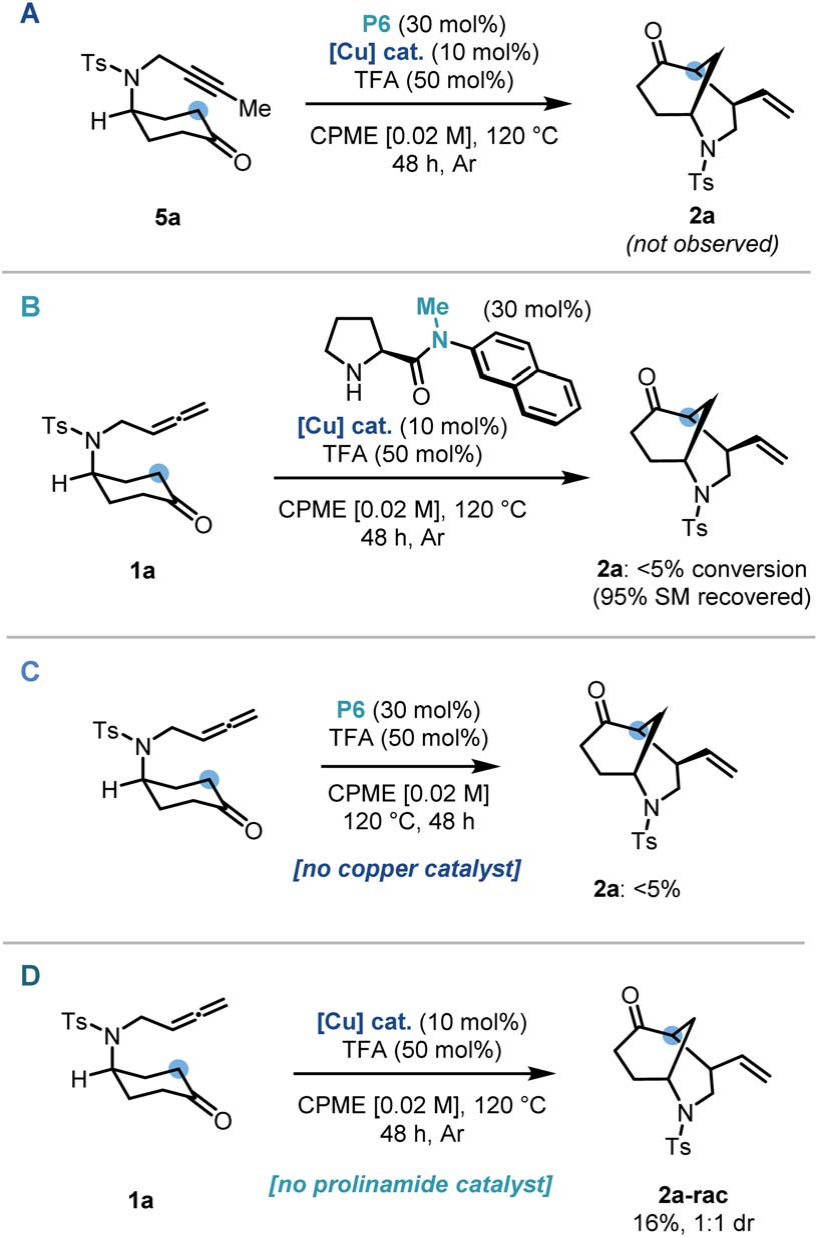
Control experiments: (A) use of an alkyne substrate. (B) Use of methylated catalyst. (C) No copper catalyst. (D) No prolinamide catalyst.

Control and deletion studies were carried out in order to probe the mechanistic pathway of our dual-catalytic entry to enantioenriched vinyl-morphan structures. A possible mechanistic pathway could proceed *via* an alkyne intermediate. To test this hypothesis, alkyne derivative (**5a**) was synthesized and subjected to the reaction conditions. However, no cyclized product was observed, hence ruling out a pathway *via* such an intermediate (Scheme 3A). During our preliminary scouting studies, prolinamide catalysts without an N–H bond (*e.g.* a tertiary amide) were found to be inferior in comparison to their secondary amide counterparts. In order to study this further, a methylated derivative of our optimized catalyst was employed under standard reaction conditions, and complete suppression of reactivity was indeed observed (Scheme 3B). Our protocol was then conducted whilst omitting each of the co-catalysts sequentially. In the absence the copper catalyst, no formation of the cyclized product was observed (Scheme 3C), whilst without the prolinamide catalyst, the morphan structure was still observed, albeit in only 16% yield and with no diastereoselectivity (Scheme 3D). This latter result points to an alternative minor pathway to racemic product presumably *via* an enol tautomer, which is operating under the reaction conditions. Accordingly, such background reactivity without the prolinamide catalyst is likely responsible for a slight erosion of enantioselectivity observed experimentally *vs.* that predicted from our computational calculations (*vide infra*).

In order to paint a full mechanistic picture, density functional theory analysis of the reaction pathway was performed. All the calculations reported herein were performed using the Amsterdam Density Functional (ADF) software.^17^ Equilibrium structures and transition structure geometries were optimized using the BLYP functional^18,19^ and the TZ2P basis set.^20^ This approach was extensively tested against *ab initio* reference benchmarks from hierarchical series up until CCSD(T).^21^ Solvent effects of Et_2_O (as CPME was not available) were accounted for using the conductor-like screen model (COSMO) of solvation.^22^ Dispersion interactions were included using Grimme's DFT-D3 correction with Becke–Johnson damping.^23^ The zeroth-order regular approximation (ZORA) was used to account for scalar relativistic effects.^24^ This level is referred to as COSMO(Et_2_O)-ZORA-BLYP-D3(BJ)/TZ2P. All stationary points have been verified, through vibrational analysis, to be minima (zero imaginary frequency) or transition states (one imaginary frequency). The character of the normal mode associated with the imaginary frequency has been analyzed to ensure it resembles the reaction under consideration. Optimized structures were illustrated using CYLview.^25^ Potential energies were refined by means of single point calculations using the M06 functional.^26^ This level is denoted COSMO(Et_2_O)-ZORA-M06/TZ2P//COSMO(Et_2_O)-ZORA-BLYP-D3(BJ)/TZ2P.

To understand the origin of enantio- and diastereocontrol of the key C–C bond forming step, we quantitatively analyzed the two competing transition structures using the activation strain model (ASM)^27^ of reactivity. ASM involves decomposing the electronic energy of the transition structure Δ*E*^‡^ into the strain Δ*E*^‡^_strain_ associated with the structural deformation of the reactants from their equilibrium geometry and the interaction Δ*E*^‡^_int_ between the deformed reactants (eqn (1)).^27^ The Δ*E*^‡^_strain_ is determined by the rigidity of the reactants and by the extent to which they must deform to achieve the geometry of the transition structure. The Δ*E*^‡^_int_ is usually stabilizing and is related to the electronic structure of the reactants and how they are mutually oriented over the course of the reaction.

1 Δ*E*^‡^ = Δ*E*^‡^_strain_ + Δ*E*^‡^_int_

Our DFT studies uncovered that the enantio- and diastereoselectivities are determined during the key nucleophilic attack of an enamine^28^ to a copper-coordinated allene^13^ in the intramolecular cyclization step.

**Scheme 4:**
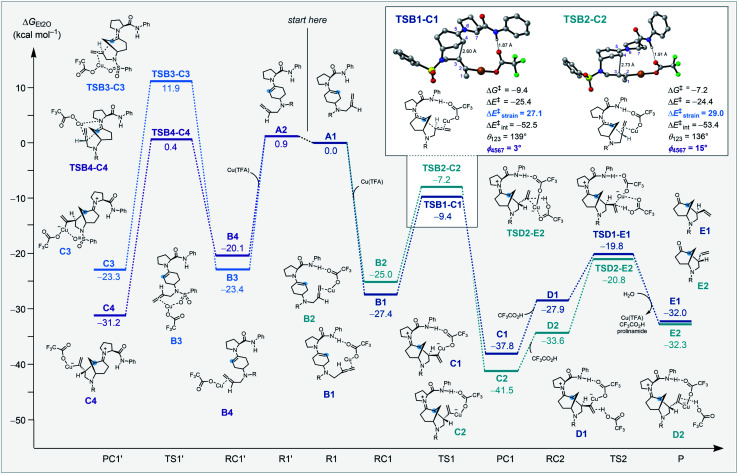
Computed reaction energy profile for the four possible pathways leading to the vinyl-substituted morphan core computed at COSMO(Et_2_O)-ZORA-M06/TZ2P//COSMO(Et_2_O)-ZORA-BLYP-D3(BJ)/TZ2P. R = SO_2_Ph.

Coordination of copper to the allene along with the interaction between the prolinamide and trifluoroacetate (ligated to the copper species) play important roles in the kinetically preferred transition structure associated with selective C–C bond formation. Enantioselectivity is determined by stabilization from the hydrogen bond interaction between the prolinamide N–H bond and the O atom of trifluoroacetate. This stabilizing interaction is only accessible when the copper-coordinated allene is positioned in close proximity to the amide. Diastereoselectivity is determined by the strain energy caused by the larger dihedral angle of the enamine (*φ*) and the smaller angle of the allene (*θ*, Scheme 4, inset). Optimal geometries of the enamine and the allene are planar and linear, respectively, and the destabilizing repulsion between the allene moiety and the pyrrolidine ring causes a greater distortion that results in a higher energy barrier.

Reaction of the cyclohexanone substrate and the prolinamide catalyst forms either *s-cis* enamine **A1** or *s-trans***A2**, and the small energy difference indicates a rapid equilibrium between these conformations. We hypothesized that the bulky substituent of the amide prevents reactivity from the top-surface of the enamine in each of the structures. The enamines and a Cu(i) species generate metal complexes and this process is highly exergonic. Reactant complexes **B1** and **B2** benefit from stabilizing hydrogen bonds between the amide N–H bond and an O atom on trifluoroacetate that are absent in **B3** and **B4**. The key C–C bond formation *via***TSB1-C1** proceeds with stabilizing hydrogen bonding interactions and no destabilizing steric clash between the prolinamide and allene, and is kinetically preferred by 2.2 kcal mol^−1^ compared to the next most favorable **TSB2-C2**. An activation strain analysis was performed on **TSB1-C1** and **TSB2-C2** to quantitatively understand the factors leading to the difference in computed reactivity and the results are summarized in (Scheme 4, inset). **TSB1-C1** goes with a lower energy barrier due to a less destabilizing activation strain (Δ*E*^‡^_strain_ = 27.1 kcal mol^−1^) compared to **TSB2-C2** (Δ*E*^‡^_strain_ = 29.0 kcal mol^−1^) that results from a smaller distortion of the enamine and the allene moieties. This step is exergonic by 10.4 kcal mol^−1^ and has a large Gibbs free energy barrier for the reverse reaction (28.4 kcal mol^−1^) that is likely irreversible. After the intramolecular cyclization, trifluoroacetic acid coordinates to the alkene of the alkylcopper(i) species **C1** to form a complex **D1**. The protonation process through **TSD1-E1** generates a vinyl group, which then regenerates Cu(TFA) and hydrolyzes the enamine to give the cyclized product **E1**.

## Conclusion

In conclusion, a prolinamide and copper(i) catalyzed highly enantio- and diastereoselective cycloisomerization of *N*/*O*-tethered allenic cyclohexanones has been demonstrated. This dual catalytic desymmetrization strategy showed broad substrate scope with respect to both the *N*-tethered and *O*-tethered substrates, affording a range of 4-vinyl-2-morphan and 4-vinyl-2-oxamorphan derivatives in high yields and enantioselectivity. DFT studies elucidated that the reaction proceeds through a key strain-minimized transition structure containing both prolinamide and copper catalysts, which were linked through a trifluoroacetate bridge, leading to the observed high enantio- and diastereoselectivities. Efforts to apply the findings of this methodology to complex molecule synthesis are ongoing, and the results will be disclosed in due course.

## Conflicts of interest

There are no conflicts to declare.

## Supplementary Material

SC-011-D0SC02878A-s001

SC-011-D0SC02878A-s002
