# Supplementary material for: Dual catalytic enantioselective desymmetrization of allene-tethered cyclohexanones
Source: Chem Sci. 2020 Jun 24;11(28):7444–50. doi: 10.1039/d0sc02878a (PMC8159440; doi:10.1039/d0sc02878a)
Supplement: SC-011-D0SC02878A-s001 [file SC-011-D0SC02878A-s001.pdf]

Electronic Supplementary Material (ESI) for Chemical Science.
This journal is © The Royal Society of Chemistry 2020

Supporting Information

for

Dual Catalytic Enantioselective Desymmetrization of Allene-Tethered Cyclohexanones

Lin Zhang,[a] Ken Yamazaki,[a] Jamie A. Leitch,[a] Ruben Manzano,[a] Victoria A. M. Atkinson,[a] Trevor A. Hamlin,*[b] and Darren J. Dixon*[a,]

[a] Department of Chemistry, Chemistry Research Laboratory, University of Oxford, Mansfield Road, Oxford, OX1 3TA (UK).

[b] Department of Theoretical Chemistry, Amsterdam Institute of Molecular and Life Sciences (AIMMS), and Amsterdam Center for Multiscale Modeling (ACMM), Vrije Universiteit Amsterdam, De Boelelaan 1083, 1081 HV Amsterdam (The Netherlands)

* E-mail: t.a.hamlin@vu.nl

* E-mail: darren.dixon@chem.ox.ac.uk

Contents

1: General Information....S3

2: Preliminary Optimization Studies S4

3: Synthesis of the Prolineamide Catalysts....S6

4: Preparation of Allene Substrates....S8

4.1: Synthesis of *N*/*O*-tethered substrates S8

4.2: Data for intermediates.... S22

4.3: Synthesis of *C*-tethered substrate S5a.... S30

5. Synthesis of Bicyclic Structures.... S33

6: Synthesis of Bicyclic Oxygen Variants S48

7: Computational Details.... S61

7.1: Approach and Main Findings.... S61

7.2: xyz Coordinates S62

8: References S101

9: NMR Spectra S102

9.1 Spectra of prolineamide catalysts S102

9.2 Spectra of starting materials.... S107

9.3: Spectra of products 2 and 4 .... S137

8.4 Spectra of the intermediates towards starting materials.... S162

1: General Information

Bulk solutions were evaporated under reduced pressure using a Büchi rotary evaporator. All solvents were commercially supplied. Petroleum ether refers to the fraction collected between 30-40 °C. Unless stated, reagents were obtained from commercial suppliers and used without further purification. Flash column chromatography (FCC) was carried out using Merck Silica gel 60, particle size 40-63μm. All reactions were followed by thin-layer chromatography (TLC) when practical, using Merck aluminium-backed Silica gel 60 F254 fluorescent treated silica which was visualized under UV light (λ_{max}= 254 or 365 nm) or by staining with aqueous basic potassium permanganate or vanillin solutions. HPLC separation was performed on Agilent Technologies 1200 series machine with the appropriate chiral column.

^{1}H, ^{13}C NMR spectra were recorded using Bruker DPX-200, Bruker AVF-400, Bruker AVG-400, Bruker AVH-400 and Bruker AVC-500 spectrometers using $CDCl_3$ (or other deuterated solvent as specified).

High resolution mass spectra (HRMS) were recorded on a Bruker μTOF mass spectrometer. Melting points were recorded in degrees Celsius (°C), using a Leica Galen III hot-stage microscope apparatus. Specific rotations were calculated from optical rotations measured using a Perkin Elmer Model 341 polarimeter with a sodium lamp and a cell length of 1 dm, concentrations (c) are reported in g/100 mL. Compound names are as generated by CambridgeSoft ChemBioDraw Ultra 12.0.

Where appropriate morphan and oxamorphan products are numbered using the following IUPAC nomenclature

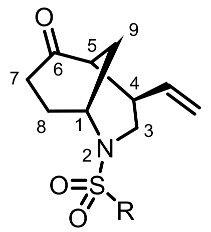

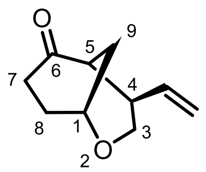

2: Preliminary Optimization Studies

Proof of concept on racemic series

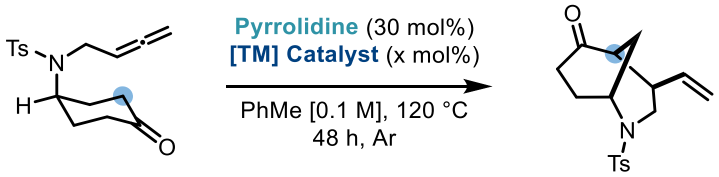

1a **2a**

entry	TM Catalyst (x mol%)	Conversion from **1a**	**2a** %[a]	dr[a]
**1**	**$Cu(OTf)_2$ (5 mol%)**	**50**	**50**	**>10:1**
2[b]	$Pd(OAc)_2$ (5 mol%)	100	17	>10:1
3[a]	$InCl_3$ (10 mol%)	6	0	-
4[a]	$IrCl_3$ (10 mol%)	31	0	-
5[a]	$AgNTf_2$ (10 mol%)	13	3	>10:1
6[a]	AgOAc (10 mol%)	8	4	>10:1
7[a]	$RuCl_3$ (10 mol%)	100	0	-

[a] = Calculated *via* ^{1}H NMR analysis of the crude reaction mixture vs. mesitylene as an internal standard. [b] = 70 °C. [c] = 4-BrPhCO_2H was added as an additive.

Chiral amine catalyst screening.

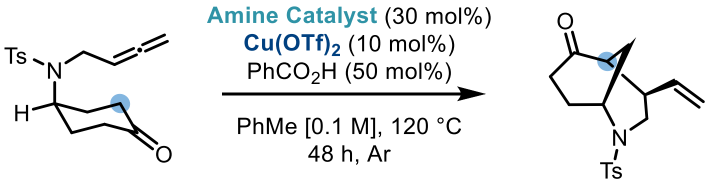

1a **2a**

entry	Amine catalyst	**2a** %[a]	dr[a]	er[b]
1	cat-**1a**	85	10:1	50:50
2	cat-**1b**	25	3:1	62:38
3	cat-**1c**	-	n/a	n/a
4	cat-**1d**	6	>10:1	68:32
5	cat-**1e**	63	>10:1	80:20
6	cat-**1f**	48	>10:1	81:19
7	cat-**1g**	12	1:1	50:50
8	cat-**1h** (**P1**)	68	8:1	72.5:27:5
9[c,d]	cat-**1h** (**P1**)	76	8:1	82:18

[a] = Calculated *via* ^{1}H NMR analysis of the crude reaction mixture vs. mesitylene as an internal standard. [b] = er were determined by HPLC on chiral columns of the purified product. [c] = CPME was used as solvent. [d] = 4-bromobenzoic acid was used as the acid additive.

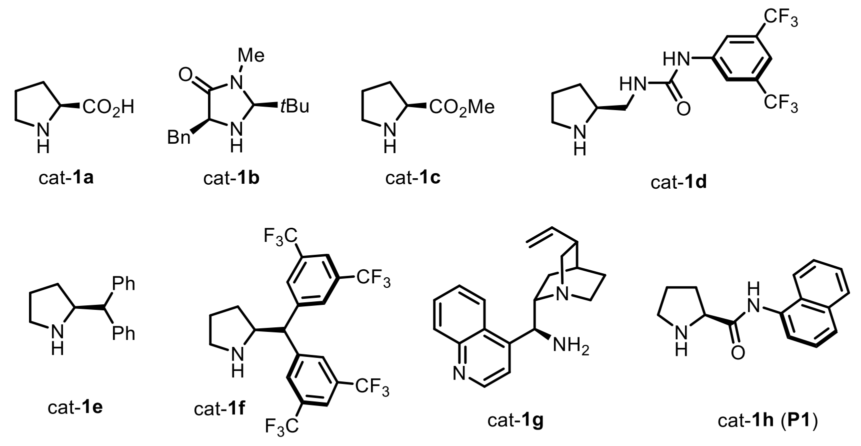

Further optimization studies **– [structures of P1-6 shown on next page]**

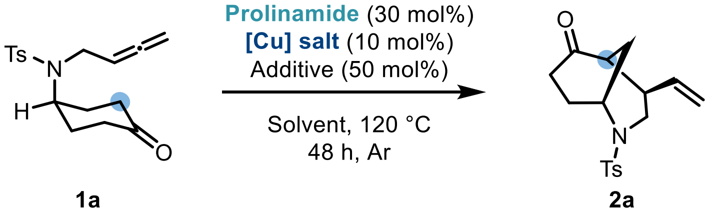

entry	Prolinamide	[Cu] salt	Additive	Solvent	[M]	**2a %**[a]	dr[a]	er[b]
1	P1	$Cu(OTf)_2$	$4\text{-}BrPhCO_2H$	DCE	0.1	55	>20:1	78:22
2	P1	$Cu(OTf)_2$	$4\text{-}BrPhCO_2H$	2-Butanol	0.1	45	>20:1	73:27
3	P1	$Cu(OTf)_2$	$4\text{-}BrPhCO_2H$	CPME	0.1	76	8:1	82:18
4	P1	$Cu(OTf)_2$	$4\text{-}BrPhCO_2H$	$PhCF_3$	0.1	39	13:1	76.5:23.5
5	P1	$Cu(OTf)_2$	$4\text{-}BrPhCO_2H$	PhOMe	0.1	12	n.d.	n.d.
6	P1	CuI	$4\text{-}BrPhCO_2H$	CPME	0.1	44	>20:1	82:18
7	P1	$Cu(OAc)_2$	$4\text{-}BrPhCO_2H$	CPME	0.1	40	7:1	72:28
8	P1	$Cu(MeCN)_4(PF_6)$	$4\text{-}BrPhCO_2H$	CPME	0.1	98	12:1	87:13
9	P1	$Cu(MeCN)_4(BF_4)$	$4\text{-}BrPhCO_2H$	CPME	0.1	76	11:1	78:22
10	P1	$Cu(tmhd)_2$	$4\text{-}BrPhCO_2H$	CPME	0.1	76	15:1	78:22
11	P1	$Cu(acac)_2$	$4\text{-}BrPhCO_2H$	CPME	0.1	93	10:1	80:20
12	P2	$Cu(MeCN)_4(PF_6)$	$4\text{-}BrPhCO_2H$	CPME	0.1	93	>20:1	82:18
13	P3	$Cu(MeCN)_4(PF_6)$	$4\text{-}BrPhCO_2H$	CPME	0.1	90	18:1	80:20
14	P4	$Cu(MeCN)_4(PF_6)$	$4\text{-}BrPhCO_2H$	CPME	0.1	99	>20:1	89.5:10.5
15	P5	$Cu(MeCN)_4(PF_6)$	$4\text{-}BrPhCO_2H$	CPME	0.1	90	18:1	89:11
16	P5	$Cu(MeCN)_4(PF_6)$	$4\text{-}BrPhCO_2H$	CPME	0.3	99	20:1	86:14
17	P5	$Cu(MeCN)_4(PF_6)$	$4\text{-}BrPhCO_2H$	CPME	0.04	91	>20:1	90:10
18	P5	$Cu(MeCN)_4(PF_6)$	$4\text{-}BrPhCO_2H$	CPME	0.02	91	>20:1	91:9
19	P5	$Cu(MeCN)_4(PF_6)$	$4\text{-}BrPhCO_2H$	CPME	0.005	33	n.d.	n.d.
20	P6	$Cu(MeCN)_4(PF_6)$	$4\text{-}BrPhCO_2H$	CPME	0.02	82	>20:1	92.5:7.5
21	P6	$Cu(MeCN)_4(PF_6$	AcOH	CPME	0.02	10	5:1	n.d.
22	P6	$Cu(MeCN)_4(PF_6$	TFA	CPME	0.02	79	>20:1	96:4
23	P6	$Cu(MeCN)_4(PF_6$	TsOH	CPME	0.02	9	n.d.	n.d.

[a] = Calculated *via* ^1H NMR analysis of the crude reaction mixture vs. mesitylene as an internal standard. [b] = er were determined by HPLC on chiral columns of the purified product

3: Synthesis of the Prolineamide Catalysts

Prolineamides **P1-P6** were prepared according to a literature procedure. The data of **P2** has been compared to the literature reported.

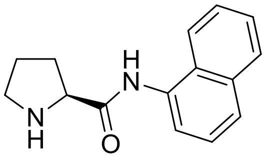

(**P1**) White solid, m.p. 63-64 °C; **[α]$_D$^{25}** = 15.9 (c = 1.0, $CHCl_3$); **^{1}H-NMR** (400 MHz, $CDCl_3$) δ 10.60 (s, 1H), 8.30 (d, *J* = 7.6 Hz, 1H), 7.87 (m, 2H), 7.65 (d, *J* = 8.4 Hz, 1H), 7.47-7.56 (m, 3H), 4.01 (dd, *J* = 4.8 Hz, 8.8 Hz, 1H), 3.08-3.20 (m, 2H), 2.23-2.33 (m, 2H), 2.10-2.18 (m, 1H), 1.75-1.90 (m, 2H); **^{13}C-NMR** (100 MHz, $CDCl_3$) δ 173.5, 134.0, 132.5, 128.7, 126.0, 125.9, 125.7, 124.3, 120.1, 117.6, 61.4, 47.5, 30.9, 26.4; **HRMS (ESI+)** m/z calculated for $C_{15}H_{16}N_2O$ $[M+H]^+$: 241.1335, found 241.1332.

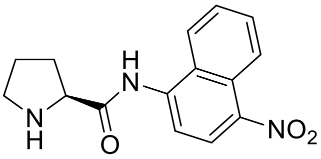

(**P3**) Yellow solid, m.p. 137-138 °C; **[α]$_D$^{25}** = 4.7 (c = 1.0, $CHCl_3$); **^{1}H-NMR** (400 MHz, DMSO-d_6) δ 11.14 (s, 1H), 8.51 (d, *J* = 8.0 Hz, 1H), 8.30-8.36 (m, 2H), 8.03 (d, *J* = 6.8 Hz, 1H), 7.75-7.83 (m, 2H), 3.94 (dd, *J* = 5.2 Hz, 9.2 Hz, 1H), 3.32-3.48 (m, 1H), 2.94-3.07 (m, 2H), 2.09-2.19 (m, 1H), 1.87-1.94 (m, 1H), 1.68-1.74 (m, 2H); **^{13}C-NMR** (100 MHz, DMSO-d_6) δ 174.7, 141.6, 139.5, 130.1, 128.1, 126.3, 125.8, 125.6, 123.7, 121.8, 114.9, 61.6, 47.2, 30.8, 26.7; **HRMS (ESI+)** m/z calculated for $C_{15}H_{15}N_3O_3$ $[M+H]^+$: 286.1186, found 286.1182.

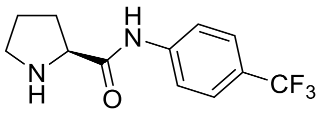

(**P4**) White solid, m.p. 105-106 °C; **[α]$_D$^{25}** = -48.6 (c = 1.0, $CHCl_3$); **^{1}H-NMR** (400 MHz, $CDCl_3$) δ 9.97 (s, 1H), 7.73 (d, *J* = 8.4 Hz, 2H), 7.57 (d, *J* = 8.4 Hz, 2H), 3.88 (dd, *J* = 5.2 Hz, 9.2 Hz, 1H), 3.07-3.13 (m, 1H), 2.95-3.01 (m, 1H), 2.15-2.27 (m, 2H), 1.99-2.07 (m, 1H), 1.70-1.83 (m, 2H); **^{13}C-NMR** (100 MHz, $CDCl_3$) δ 173.9, 140.8, 126.14, 126.10, 125.5 (q, *J* = 269.7 Hz), 118.8, 61.0, 47.3, 30.7, 26.3; **HRMS (ESI+)** m/z calculated for $C_{12}H_{13}F_3N_2O$ $[M+H]^+$: 259.1053, found 259.1051.

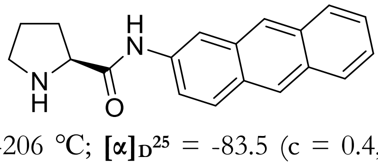

(**P5**) Off-white solid, m.p. 205-206 °C; **[α]$_D$^{25}** = -83.5 (c = 0.4, $CHCl_3$); **^{1}H-NMR** (400 MHz, $CDCl_3$) δ 9.95 (s, 1H), 8.52 (m, 1H), 8.37 (d, *J* = 6.8 Hz, 2H), 7.95-7.98 (m, 3H), 7.40-7.50 (m, 3H), 3.93 (dd, *J* = 5.2 Hz, 9.2 Hz, 1H), 3.09-3.15 (m, 1H), 3.00-3.06 (m, 1H), 2.22-2.31 (m, 1H), 2.07-2.15 (m, 1H), 2.01 (s, 1H), 1.72-1.86 (m, 2H); **^{13}C-NMR** (100 MHz, DMSO-d_6) δ 174.4, 136.0, 132.2, 132.1, 130.9, 129.3, 129.1, 128.5, 128.2, 126.3, 126.1, 125.4, 121.6, 114.3, 61.4, 47.3, 31.0,

26.4; **HRMS (ESI+)** m/z calculated for $C_{19}H_{18}N_2O$ $[M+H]^+$: 291.1492, found 291.1490.

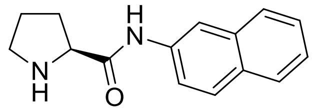

(**P6**) White solid, m.p. 86-87 ℃; **$[\alpha]_D^{25}$** = -106.9 (c = 0.7, $CHCl_3$); **^{1}H-NMR** (400 MHz, $CDCl_3$) δ 9.92 (s, 1H), 8.31 (d, *J* = 2.0 Hz, 1H), 7.76-7.81 (m, 3H), 7.56 (dd, *J* = 2.4 Hz, 8.8 Hz, 1H), 7.43-7.47 (m, 1H), 7.36-7.40 (m, 1H), 3.92 (dd, *J* = 5.2 Hz, 9.2 Hz, 1H), 3.08-3.14 (m, 1H), 2.99-3.05 (m, 1H), 2.20-2.30 (m, 2H), 2.05-2.13 (m, 1H), 1.71-1.86 (m, 2H); **^{13}C-NMR** (100 MHz, $CDCl_3$) δ 173.6, 135.3, 133.9, 130.5, 128.6, 127.6, 127.5, 126.3, 124.7, 119.6, 115.7, 61.1, 47.4, 30.8, 26.3; **HRMS (ESI+)** m/z calculated for $C_{15}H_{16}N_2O$ $[M+H]^+$: 241.1335, found 241.1334.

4: Preparation of Allene Substrates

4.1: Synthesis of *N/O*-tethered substrates

Synthesis of N-tethered substrates 1a-p

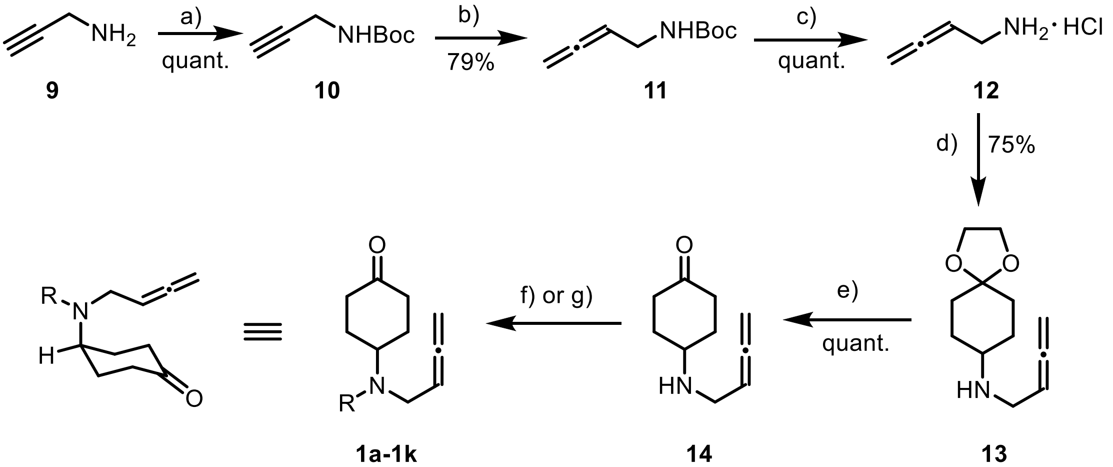

Reaction conditions: a) Boc_2O, DCM, rt; b) paraformaldehyde, CuI, Cy_2NH, 1,4-dioxane, reflux; c) conc. HCl (aq.), EtOH, rt; d) Et_3N, $NaBH(OAc)_3$, DCE, rt; e) 3M HCl (aq.), THF, 50 °C; f) $ArSO_2Cl$, Et_3N, DMAP, DCM, rt (R = $ArSO_2$); g) BnBr, DIPEA, CH_3CN, rt (R = Bn).

Typical synthetic procedure for compounds 1a-1m (with **1a** as an example):

To a solution of 2-propynylamine **9** (10.3 mL, 150.0 mmol) in DCM (200 mL) was added Boc_2O (32.7 g, 150.0 mmol). The mixture was stirred overnight at room temperature. After the starting materal was consumed, the reaction mixture was quenched with brine. The phases were separated and the aqueous phase was extracted with DCM (3×50 mL). The combined organic layers were dried over $MgSO_4$, filtered and evaporated. The resulted reaction product was purified by column chromatography to afford **10** quantitatively as a white solid.

A flask was charged with *tert*-butyl prop-2-yn-1-ylcarbamate **10** (4.66 g, 30.0 mmol), paraformaldehyde (2.25 g, 75.0 mmol), and CuI (2.86 g, 15.0 mmol) under argon atmosphere, dry 1,4-dioxane (130 mL) was then added, followed by Cy_2NH (10.7 mL, 54.0 mmol). The mixture was refluxed for 3~4 h, TLC showed full conversion. The reaction mixture was cooled to room temperature and quenched with brine. The phases were separated and the aqueous phase was extracted with Et_2O (3×40 mL). The combined organic layers were dried with $MgSO_4$, filtered and evaporated. The resulted reaction products were purified by column chromatography to afford compound **11** (79% yield).

To a solution of *tert*-butyl buta-2,3-dien-1-ylcarbamate **11** (3.38 g, 20 mmol) in ethanol (20 mL), excess of conc. HCl (aq.) (5.0 mL) was added carefully. The mixture was then stirred overnight at room temperature. The solvent was removed and gave the desired product **12** as a brown solid, used directly in the next step without further purification.

To a solution of 1,4-dioxaspiro[4.5]decan-8-one (2.50 g, 16.0 mmol), **12** (1.77 g, 16.8 mmol) and

Et_3N (2.34 mL, 16.8 mmol) in DCE (40 mL), was added $NaBH(OAc)_3$ (5.09 g, 24.0 mmol) slowly. The reaction mixture was stirred overnight at room temperature. The reaction was quenched with a saturated aqueous solution of NH_4Cl. The phases were separated and the aqueous phase was extracted with DCM (3×30 mL). The combined organic layers were dried over $MgSO_4$, filtered and evaporated to yield compound **13** quantitatively as brown solid. The crude amine was sufficiently pure to be used in the next step.

Acetal **13** (15.0 mmol) was dissolved in a 1:1 mixture of THF and 3M HCl (aq.) (50 mL) and the solution was stirred at 50 °C until full consumption of starting material. The reaction mixture was separated and the aqueous phase was basified with 3M NaOH (aq.), and extracted with EtOAc (4×30 mL). The combined organic layers were dried over $MgSO_4$, filtered and evaporated to yield 4-(buta-2,3-dien-1-ylamino)cyclohexanone **14** quantitatively as brown oil, which was used directly in the next step.

4-(Buta-2,3-dien-1-ylamino)cyclohexanone **14** (496 mg, 3.0 mmol), tosyl chloride (744 mg, 3.9 mmol), Et_3N (0.54 mL, 3.9 mmol) and DMAP (37 mg, 0.3 mmol) were dissolved in DCM (15 mL), and the reaction mixture was stirred at room temperature until the starting material was fully consumed. The reaction was quenched with a saturated aqueous solution of NH_4Cl. The phases were separated and the aqueous phase was extracted with DCM (2×15 mL). The combined organic layers were dried over $MgSO_4$, filtered and evaporated. The residue was purified by column chromatography to afford **1a** as a white solid (73% yield).

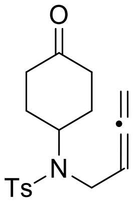

(**1a**) White solid, m.p. 103-104 °C; **^{1}H-NMR** (400 MHz, $CDCl_3$) δ 7.72 (d, *J* = 8.0 Hz, 2H), 7.29 (d, *J* = 8.0 Hz, 2H), 5.16 (p, *J* = 6.8 Hz, 1H), 4.73-4.75 (m, 2H), 4.17-4.25 (m, 1H), 3.84-3.80 (m, 2H), 2.42 (s, 3H), 2.35-2.45 (m, 4H), 1.89-1.95 (m, 4H); **^{13}C-NMR** (100 MHz, $CDCl_3$) δ 208.7, 208.4, 143.4, 138.0, 129.8, 126.8, 89.1, 55.5, 42.9, 40.0, 30.4, 21.5; **HRMS (ESI+)** m/z calculated for $C_{17}H_{21}NO_3S$ $[M+Na]^+$: 342.1134, found 342.1136.

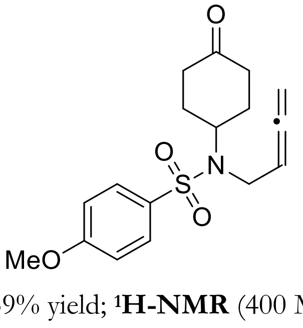

(**1b**) White solid, m.p. 105-106 °C; 59% yield; **^{1}H-NMR** (400 MHz, $CDCl_3$) δ 7.76 (d, *J* = 8.8 Hz, 2H), 6.95 (d, *J* = 8.8 Hz, 2H), 5.15 (p, *J* = 6.8 Hz, 1H), 4.72-4.75 (m, 2H), 4.15-4.23 (m, 1H), 3.86 (s, 3H), 3.81-3.84 (m, 2H), 2.34-2.41 (m, 4H), 1.88-1.95 (m, 4H); **^{13}C-NMR** (100 MHz, $CDCl_3$) δ 208.8, 208.3, 162.8, 132.6, 128.9, 114.3, 89.1, 76.7, 55.6, 55.4, 42.8, 40.0, 30.3; **HRMS (ESI+)** m/z calculated for $C_{17}H_{21}NO_4S$ $[M+H]^+$: 336.1264, found 336.1265.

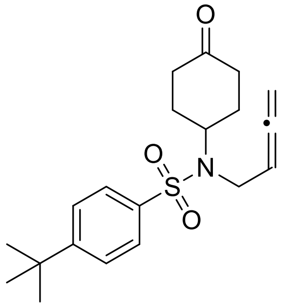

(**1c**) White solid, m.p. 89-90 °C; 82% yield; **^{1}H-NMR** (400 MHz, $CDCl_3$) δ 7.76 (d, *J* = 9.2 Hz, 2H), 7.50 (d, *J* = 8.8 Hz, 2H), 5.15 (p, *J* = 6.8 Hz, 1H), 4.72-4.75 (m, 2H), 4.20-4.29 (m, 1H), 3.84-3.87 (m, 2H), 2.34-2.43 (m, 4H), 1.92-1.99 (m, 4H), 1.34 (s, 9H); **^{13}C-NMR** (100 MHz, $CDCl_3$) δ 208.8, 208.4, 156.4, 137.9, 126.7, 126.1, 89.2, 76.7, 55.6, 42.9, 40.1, 35.1, 31.1, 30.1; **HRMS (ESI+)** m/z calculated for $C_{20}H_{27}NO_3S$ $[M+H]^+$: 362.1784, found 362.1784.

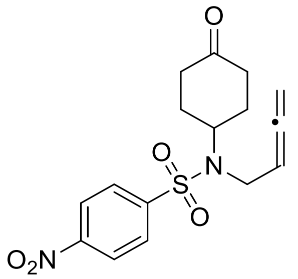

(**1d**) Yellow solid, m.p. 124-125 °C; 61% yield; **^{1}H-NMR** (400 MHz, $CDCl_3$) δ 8.36 (m, 2H), 8.03 (m, 2H), 5.14 (p, *J* = 6.8 Hz, 1H), 4.76-4.79 (m, 2H), 4.20-4.28 (m, 1H), 3.89-3.92 (m, 2H), 2.41-2.45 (m, 4H), 1.95-2.00 (m, 4H); **^{13}C-NMR** (100 MHz, $CDCl_3$) δ 208.6, 207.9, 150.0, 146.9, 128.1, 124.5, 88.5, 77.2, 56.2, 43.2, 39.9, 30.5; **HRMS (ESI+)** m/z calculated for $C_{16}H_{18}N_2O_5S$ $[M+Na]^+$: 373.0829, found 373.0829.

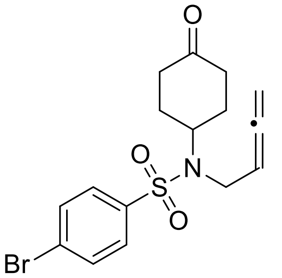

(**1e**) Brown solid, m.p. 89-90 °C; 78% yield; **^{1}H-NMR** (400 MHz, $CDCl_3$) δ 7.70 (m, 2H), 7.63 (m, 2H), 5.15 (p, *J* = 6.8 Hz, 1H), 4.74-4.77 (m, 2H), 4.16-4.25 (m, 1H), 3.83-3.86 (m, 2H), 2.39-2.43 (m, 4H), 1.91-1.97 (m, 4H); **^{13}C-NMR** (100 MHz, $CDCl_3$) δ 208.5, 208.4, 140.1, 132.5, 128.4, 127.5, 88.8, 76.9, 55.8, 43.0, 40.0, 34.1, 30.4; **HRMS (ESI+)** m/z calculated for $C_{16}H_{18}BrNO_3S$ $[M+Na]^+$: 406.0083, found 406.0083.

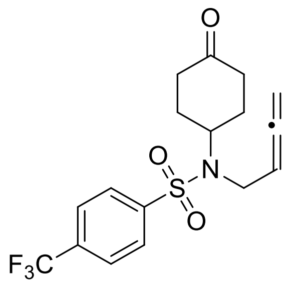

(**1f**) White solid, m.p. 87-88 °C;49% yield; **^{1}H-NMR** (400 MHz, $CDCl_3$) δ 7.98 (d, *J* = 8.0 Hz, 2H), 7.78 (d, *J* = 8.4 Hz, 2H), 5.15 (p, *J* = 6.8 Hz, 1H), 4.74-4.77 (m, 2H), 4.20-4.28 (m, 1H), 3.87-3.90 (m, 2H), 2.40-2.44 (m, 4H), 1.94-1.99 (m, 4H); **^{13}C-NMR** (100 MHz, $CDCl_3$) δ 208.5, 208.2, 144.7, 133.5,134.1, 128.5,127.3, 126.42, 126.38, 126.35, 126.31, 124.5 (q, *J* = 272 Hz), 121.8, 88.7, 77.2, 56.0, 43.1, 39.9, 30.5; **HRMS (ESI+)** m/z calculated for $C_{17}H_{18}F_3NO_3S$ $[M+Na]^+$: 396.0852, found 396.0851.

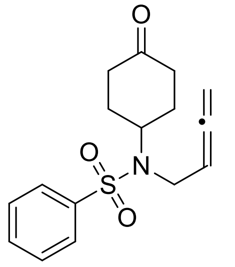

(**1g**) White solid, m.p. 129-130 °C; 63% yield; **^{1}H-NMR** (400 MHz, $CDCl_3$) δ 7.85-7.87 (m, 2H), 7.57-7.61 (m, 1H), 7.50-7.55 (m, 2H), 5.16 (p, *J* = 6.8 Hz, 1H), 4.73-4.76 (m, 2H), 4.20-4.27 (m, 1H), 3.85-3.88 (m, 2H), 2.36-2.43 (m, 4H), 1.91-1.97 (m, 4H); **^{13}C-NMR** (100 MHz, $CDCl_3$) δ 208.7, 208.4, 141.0, 132.6, 129.2, 126.8, 89.1, 76.8, 55.6, 43.0, 40.1, 30.4; **HRMS (ESI+)** m/z calculated for $C_{16}H_{19}NO_3S$ $[M+Na]^+$: 328.0978, found 328.0978.

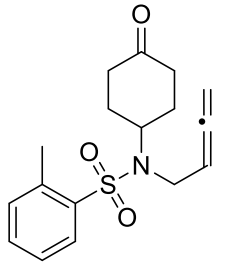

(**1h**) White solid, m.p. 66-67 °C; 75% yield; **^{1}H-NMR** (400 MHz, $CDCl_3$) δ 8.00 (d, *J* = 7.6 Hz, 1H), 7.47 (t, *J* = 8.0 Hz, 1H), 7.33 (t, *J* = 8.0 Hz, 2H), 5.08 (p, *J* = 6.8 Hz, 1H), 4.68-4.71 (m, 2H), 4.16-4.24 (m, 1H), 3.84-3.87 (m, 2H), 2.62 (s, 3H), 2.40-2.43 (m, 4H), 1.97-2.12 (m, 4H); **^{13}C-NMR** (100 MHz, $CDCl_3$) δ 208.7, 208.4, 138.1, 137.6, 132.9, 132.7, 129.9, 126.2, 88.8, 76.6, 55.1, 42.7, 40.1, 30.5, 20.3; **HRMS (ESI+)** m/z calculated for $C_{17}H_{21}NO_3S$ $[M+H]^+$: 320.1315, found 320.1316.

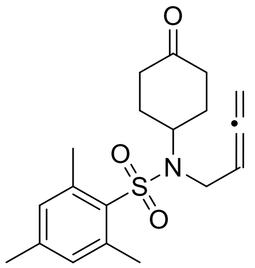

(**1i**) White solid, m.p. 139-140 °C; 67% yield; **^{1}H-NMR** (400 MHz, $CDCl_3$) δ 6.96 (s, 2H), 5.00 (p, *J* = 6.8 Hz, 1H), 4.60-4.73 (m, 2H), 4.14-4.26 (m, 1H), 3.70-3.84 (m, 2H), 2.61 (s, 6H), 2.37-2.50 (m, 4H), 2.30 (s, 3H), 1.96-2.21 (m, 4H); **^{13}C-NMR** (100 MHz, $CDCl_3$) δ 208.8, 208.2, 142.6, 140.1, 133.0, 132.0, 88.5, 76.3, 54.5, 42.1, 40.1, 30.2, 22.7, 20.9; **HRMS (ESI+)** m/z calculated for $C_{19}H_{25}NO_3S$ $[M+Na]^+$: 370.1447, found 370.1447.

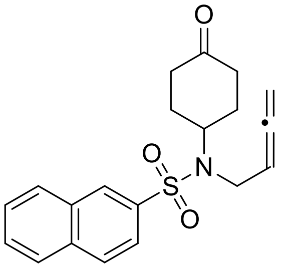

(**1j**) White solid, m.p. 90-91 °C; 80% yield; **^{1}H-NMR** (400 MHz, $CDCl_3$) δ 8.44 (d, *J* = 1.6 Hz, 1H), 7.96 (d, *J* = 8.4 Hz, 2H), 7.90 (d, *J* = 7.2 Hz, 1H), 7.80 (dd, *J* = 2.0 Hz, 8.4 Hz, 1H), 7.61-7.67 (m, 2H), 5.19 (p, *J* = 6.8 Hz, 1H), 4.72-4.75 (m, 2H), 4.27-4.34 (m, 1H), 3.91-3.94 (m, 2H), 2.35-2.46 (m, 4H), 1.92-1.99 (m, 4H); **^{13}C-NMR** (100 MHz, $CDCl_3$) δ 208.7, 208.4, 137.7, 134.7, 132.2, 129.6, 129.2, 128.8, 128.3, 127.9, 127.7, 122.0, 89.1, 76.8, 55.7, 43.0, 40.0 30.5; **HRMS (ESI+)** m/z calculated for $C_{20}H_{21}NO_3S$ $[M+Na]^+$: 378.1134, found 378.1134.

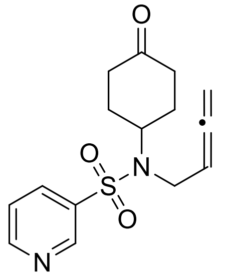

(**1k**) Brown solid, m.p. 109-110 °C; 59% yield; **^{1}H-NMR** (400 MHz, $CDCl_3$) δ 9.07 (d, *J* = 2.4 Hz, 1H), 8.80 (d, *J* = 4.8 Hz, 1H), 8.12-8.15 (m, 1H), 7.47 (dd, *J* = 4.8 Hz, 8.0 Hz, 1H), 5.13 (p, *J* = 6.8 Hz, 1H), 4.74-4.77 (m, 2H), 4.20-4.28 (m, 1H), 3.87-3.90 (m, 2H), 2.38-2.44 (m, 4H), 1.94-2.00 (m, 4H); **^{13}C-NMR** (100 MHz, $CDCl_3$) δ 208.6, 208.1, 153.2, 147.8, 137.8, 134.4, 123.8, 88.6, 77.1, 55.9, 43.0, 39.9, 30.5; **HRMS (ESI+)** m/z calculated for $C_{15}H_{18}N_2O_3S$ $[M+H]^+$: 307.1111, found 307.1110.

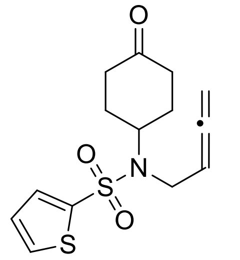

(**1l**) Brown solid, m.p. 104-105 °C; 71% yield; **^{1}H-NMR** (400 MHz, $CDCl_3$) δ 7.57-7.61 (m, 2H), 7.08-7.10 (m, 1H), 5.21 (p, *J* = 6.8 Hz, 1H), 4.77-4.80 (m, 2H), 4.24-4.32 (m, 1H), 3.86-3.89 (m, 2H), 2.38-2.45 (m, 4H), 1.91-1.99 (m, 4H); **^{13}C-NMR** (100 MHz, $CDCl_3$) δ 208.6, 208.5, 141.8, 131.8, 131.6, 127.4, 88.8, 76.9, 55.9, 43.1, 40.0, 30.3; **HRMS (ESI+)** m/z calculated for $C_{14}H_{17}NO_3S_2$ $[M+Na]^+$: 334.0542, found 334.0544.

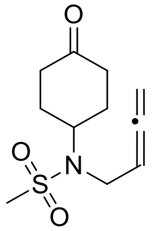

(**1m**) White solid, m.p. 67-68 °C; 48% yield; **^{1}H-NMR** (400 MHz, $CDCl_3$) δ 5.20 (p, *J* = 6.8 Hz, 1H), 4.81-4.84 (m, 2H), 4.14-4.22 (m, 1H), 3.85-3.88 (m, 2H), 2.93 (m, 3H), 2.45-2.49 (m, 4H), 2.11-2.16 (m, 2H), 1.97-2.08 (m, 2H); **^{13}C-NMR** (100 MHz, $CDCl_3$) δ 208.7, 208.5, 88.7, 77.1, 55.5, 42.6, 41.2, 39.9, 30.8; **HRMS (ESI+)** m/z calculated for $C_{11}H_{17}NO_3S$ $[M+H]^+$: 244.1002, found 244.1004.

To a solution of **14** (0.83 g, 5.0 mmol) in dry CH_3CN (20 mL) was added *N, N*-diisopropylethylamine (1.24 mL, 7.5 mmol), followed by benzyl bromide (0.65 mL, 5.5 mmol) and the reaction was stirred overnight at room temperature. Solvent was removed in vacuum, the residue product was diluted with sat. NH_4Cl (aq.), extracted with EtOAc (3×10 mL). The combined organic layers were washed with brine, dried over $MgSO_4$, filtered and concentrated in vacuum. The crude product was purified by FCC to afford **1o** as brown oil (0.46 g, 36%).

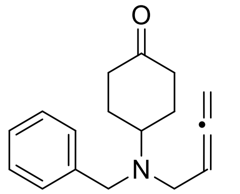

(**1n**) Brown oil; **^{1}H-NMR** (400 MHz, $CDCl_3$) δ 7.32-7.39 (m, 4H), 7.24-7.29 (m, 1H), 5.16 (p, *J* = 7.2 Hz, 1H), 4.72-4.74 (m, 2H), 3.74 (s, 2H), 3.23-3.26 (m, 2H), 3.12 (dt, *J* = 3.2 Hz, 10.0 Hz, 1H), 2.47-2.52 (m, 2H), 2.29-2.37 (m, 2H), 2.07-2.16 (m, 2H), 1.83-1.93 (m, 2H); **^{13}C-NMR** (100 MHz, $CDCl_3$) δ 211.3, 209.2, 140.1, 128.33, 128.25, 126.8, 87.4, 74.9, 56.2, 53.7, 48.8, 39.6, 28.2; **HRMS (ESI+)** m/z calculated for $C_{17}H_{21}NO$ $[M+H]^+$: 256.1696, found 256.1698.

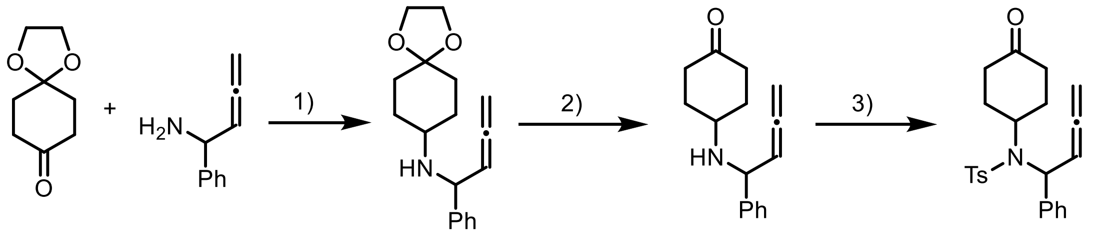

15 **16** **17** **1o**

Reaction conditions: 1) $NaBH(OAc)_3$, HOAc, DCE, rt; 2) 3M HCl (aq.), THF, 50 °C; 3) TsCl, pyridine, DCM, rt.

To a solution of 1,4-dioxaspiro[4.5]decan-8-one (2.34 g, 17.0 mmol) and 1-phenylbuta-2,3-dien-1-amine **15**[2] (2.66 g, 17.0 mmol) in DCE (70 mL), was added $NaBH(OAc)_3$ (10.8 g, 51.0 mmol) slowly, and then HOAc (0.97 mL, 17.0 mmol) was added. The reaction mixture was stirred overnight at room temperature. The reaction was quenched with brine. The phases were separated and the aqueous phase was extracted with DCM (3×40 mL). The combined organic layers were dried over $MgSO_4$, filtered and evaporated to yield compound **16** quantitatively as white solid. The crude amine was sufficiently pure to be used in the next step.

Acetal **16** (15.0 mmol) was dissolved in a 1:1 mixture of THF and 3M HCl (aq.) (30 mL) and the solution was stirred at 50 °C until starting material was full consumed. The reaction mixture was separated and the aqueous phase was basified with 3M NaOH (aq.), and extracted with EtOAc (4×30 mL). The combined organic layers were dried over $MgSO_4$, filtered and evaporated to yield ketone **17** quantitatively as brown oil, which was used directly in the next step without further purification.

4-((1-Phenylbuta-2,3-dien-1-yl)amino)cyclohexanone **17** (724 mg, 3.0 mmol) and TsCl (744 mg, 3.9 mmol) were dissolved in DCM (10 mL) and pyridine (10 mL), the reaction mixture was stirred at room temperature overnight. The solvent was removed in vacuum, and the residue was diluted with EtOAc and brine. The phases were separated and the aqueous phase was extracted with EtOAc (3×15 mL). The combined organic layers were dried over $MgSO_4$, filtered and evaporated. The crude product was purified by column chromatography to afford **1n** (42% yield).

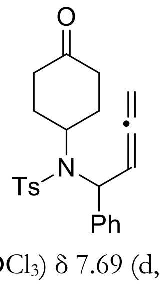

(**1o**) White solid; **^{1}H-NMR** (400 MHz, $CDCl_3$) δ 7.69 (d, J = 8.4 Hz, 2H), 7.53 (d, J = 8.4 Hz, 2H), 7.31-7.37 (m, 3H), 7.25-7.27 (m, 2H), 5.58-5.65 (m, 1H), 5.56 (d, J = 7.2 Hz, 1H), 4.60-4.70 (m, 2H), 3.63 (dt, J = 3.6 Hz, 12.0 Hz, 1H), 2.42 (s, 3H), 2.34-2.40 (m, 2H), 2.19-2.30 (m, 3H), 1.98-2.07 (m, 2H), 1.47-1.54 (m, 1H); **^{13}C-NMR** (100 MHz, $CDCl_3$) δ 208.9, 208.5, 142.9, 139.1, 138.8, 129.3, 128.3, 128.1, 128.0, 127.2, 89.0, 77.31, 60.2, 56.5, 40.3, 40.2, 31.4, 30.3, 21.4; **HRMS (ESI+)** m/z calculated for $C_{23}H_{25}NO_3S$ $[M+H]^+$: 396.1628, found 396.1629.

Synthetic procedure for substrate **1p**:

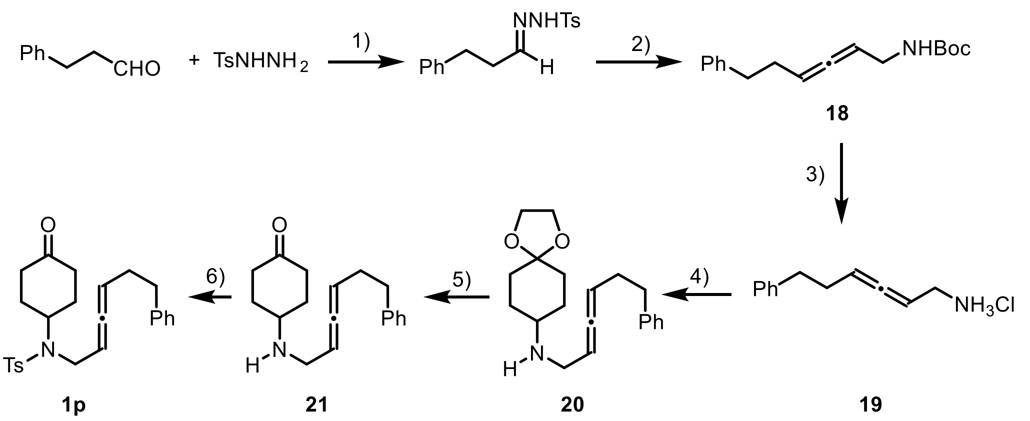

Reaction conditions: 1) 1,4-dioxane, 60 °C; 2) *tert*-butyl prop-2-yn-1-ylcarbamate, NaO^{t}Bu, CuI, 1,4-dioxane, 90 °C; 3) conc. HCl (aq.), EtOH, rt; 4) NaBH(OAc)$_3$, Et$_3$N, DCE, rt; 5) 3M HCl (aq.), THF, 50 °C; 6) TsCl, Et$_3$N, DMAP, DCM, rt.

3-Phenylpropanal (1.45 mL, 11.0 mmol) and 4-methylbenzenesulfonhydrazide (2.04 g, 11.0 mmol) were dissolved in 1,4-dioxane (30 mL), the mixture was stirred at 60 °C for 0.5 h. Sodium *tert*-butoxide (2.64 g, 27.5 mmol), *tert*-butyl prop-2-yn-1-ylcarbamate (0.78 g, 5.0 mmol) and CuI (0.19 g, 1.0 mmol) were then added into the mixture, stirred for another hour at 90 °C under argon atmosphere. The reaction was then cooled to room temperature and diluted with sat. NH$_4$Cl (aq.), extracted with EtOAc (3×20 mL). The combined organic layers were washed with brine, dried over MgSO$_4$, filtered and concentrated in vacuum. The crude product was purified by FCC to afford *tert*-butyl (6-phenylhexa-2,3-dien-1-yl)carbamate **18** as yellow oil (437 mg, 32%).

To a solution of *tert*-butyl (6-phenylhexa-2,3-dien-1-yl)carbamate **18** (1.37 g, 5.0 mmol) in ethanol (15 mL), excess of conc. HCl (aq.) (4.0 mL) was added carefully. The mixture was then stirred overnight at room temperature. The solvent was removed and afforded desired product **19** as a brown solid, used directly in the next step without further purification.

To a solution of 1,4-dioxaspiro[4.5]decan-8-one (0.63 g, 4.0 mmol), **19** (839 mg, 4.0 mmol), Et$_3$N (0.58 mL, 4.2 mmol) in DCE (15 mL), was added NaBH(OAc)$_3$ (1.27 g, 6.0 mmol). The reaction mixture was stirred overnight at room temperature. The reaction was quenched with a saturated aqueous solution of NH$_4$Cl. The phases were separated and the aqueous phase was extracted with DCM (3×20 mL). The combined organic layers were dried over MgSO$_4$, filtered and evaporated to yield amine **20** quantitatively as brown oil. The crude amine was sufficiently pure to be used in the next step.

Acetal **20** (1.25 g, 4.0 mmol) was dissolved in a 1:1 mixture of THF and 3M HCl (aq.) (10 mL) and the solution was stirred at 50 °C until full consumption of starting material. The reaction mixture was separated and the aqueous phase was basified with 3M NaOH (aq.), and extracted with EtOAc (4×30 mL). The combined organic layers were dried over MgSO$_4$, filtered and evaporated to yield 4-((6-phenylhexa-2,3-dien-1-yl)amino)cyclohexanone **21** quantitatively as brown oil, which was used directly in the next step.

4-((6-Phenylhexa-2,3-dien-1-yl)amino)cyclohexanone **21** (269 mg, 1.0 mmol), tosyl chloride (286 mg, 1.5 mmol), Et_3N (0.21 mL, 1.5 mmol) and DMAP (12 mg, 0.1 mmol) were dissolved in DCM (5 mL), and the reaction mixture was stirred at room temperature until the starting material was fully consumed. The reaction was quenched with a saturated aqueous solution of NH_4Cl. The phases were separated and the aqueous phase was extracted with DCM (2×15 mL). The combined organic layers were dried over $MgSO_4$, filtered and evaporated. The residue was purified by column chromatography to afford **1p** (195 mg, 46% yield).

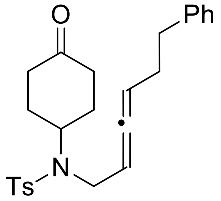

(**1p**) Pale yellow oil; **^{1}H-NMR** (400 MHz, $CDCl_3$) δ 7.70-7.73 (m, 2H), 7.27-7.30 (m, 2H), 7.24-7.26 (m, 2H), 7.14-7.17 (m, 3H), 5.14-5.20 (m, 1H), 5.07-5.14 (m, 1H), 4.12-4.20 (m, 1H), 3.72-3.78 (m, 1H), 3.58-3.65 (m, 1H), 2.63-2.76 (m, 2H), 2.41 (s, 3H), 2.21-2.38 (m, 6H), 1.79-1.91 (m, 4H); **^{13}C-NMR** (100 MHz, $CDCl_3$) δ 208.6, 204.1, 143.2, 141.2, 138.1, 129.7, 128.4, 128.2, 126.8, 125.8, 92.1, 90.1, 55.5, 43.4, 40.0, 39.9, 35.2, 30.4, 30.0, 29.9, 21.4; **HRMS (ESI+)** m/z calculated for $C_{25}H_{29}NO_3S$ $[M+H]^+$: 424.1941, found 424.1939.

Synthesis of O-tethered substrates 3a-l

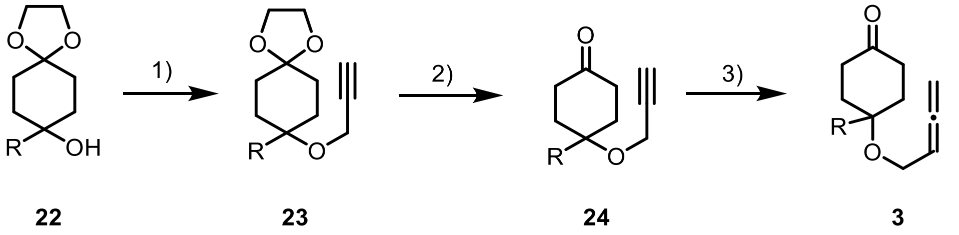

Reaction conditions: 1) *n*-BuLi, HMPA, 3-bromopropyne, THF, 0 °C~rt; 2) pyridinium *p*-toluenesulfonate, acetone, H_2O, 80 °C; 3) paraformaldehyde, CuI, Cy_2NH, 1,4-dioxane, 110 °C.

Typical synthetic procedure for compounds 3 (with **3a** as an example):

1,4-Dioxaspiro[4.5]decan-8-ol **22a** (6.17 g, 39.0 mmol) and hexamethylphosphoramide (6.79 mL, 39.0 mmol) were dissolved in anhydrous THF, and the mixture was stirred at 0 °C under argon atmosphere. And then *n*-BuLi (27 mL, 1.6 M) was added slowly into the reaction mixture over 10 minutes. 3-Bromopropyne (3.36 mL, 39.0 mmol, 80% in toluene) was added after 30 minutes. The reaction was allowed to warm to room temperature and stirred overnight. The reaction was quenched with brine. The phases were separated and the aqueous phase was extracted with EtOAc (3×40 mL). The combined organic layers were dried over Na_2SO_4, filtered, evaporated, the residue was purified by column chromatography to afford **23a** as yellow oil (3.52 g, 46% yield).

8-(Prop-2-yn-1-yloxy)-1,4-dioxaspiro[4.5]decane **23a** (2.16 g, 11 mmol) and pyridinium *p*-toluenesulfonate (553 mg, 2.2 mmol) were dissolved in acetone (22 mL) and water (11 mL). The mixture was stirred at 80 °C for 8 h. Acetone was removed under vacuum, and the aqueous phase was extracted with EtOAc (3×20 mL). The combined organic layers were dried over Na_2SO_4, filtered and evaporated. The residue was purified by column chromatography to afford 4-(prop-2-yn-1-yloxy)cyclohexanone **24a** quantitatively.

A flask was charged with 4-(prop-2-yn-1-yloxy)cyclohexanone **24a** (0.91 g, 6.0 mmol), paraformaldehyde (0.45 g, 15.0 mmol) and CuI (0.57 g, 3.0 mmol), the flask was evacuated and filled with argon (3~4 times). Anhydrous 1,4-dioxane (24 mL) was added, followed by the addition of Cy_2NH (2.15 mL, 10.8 mmol). The mixture was stirred at 110 °C under argon atmosphere for 4 h. The reaction mixture was cooled to room temperature and diluted with saturated aqueous solution of NH_4Cl. The phases were separated and the aqueous phase was extracted with EtOAc (3×15 mL). The combined organic layers were dried over anhydrous Na_2SO_4, filtered and evaporated. The residue was purified by column chromatography to afford **3a** as yellow oil (0.82 g, 82%).

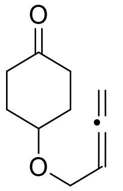

(**3a**) Yellow oil; **^{1}H-NMR** (400 MHz, $CDCl_3$) δ 5.25 (p, *J* = 6.8 Hz, 1H), 4.78-4.81 (m, 2H), 4.07-4.10 (m, 2H), 3.79-3.83 (m, 1H), 2.53-2.61 (m, 2H), 2.22-2.28 (m, 2H), 2.03-2.11 (m, 2H), 1.89-1.97 (m, 2H); **^{13}C-NMR** (100 MHz, $CDCl_3$) δ 211.2, 209.0, 88.0, 75.8, 71.8, 66.1, 37.2, 30.5; **HRMS**

(ESI+) m/z calculated for $C_{10}H_{14}O_2$ [M+H]$^+$: 167.1067, found 167.1066.

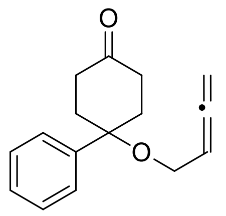

(**3b**) Yellow oil; 43% yield; **^{1}H-NMR** (400 MHz, $CDCl_3$) δ 7.44 (d, J = 8.0 Hz, 2H), 7.38 (t, J = 8.0 Hz, 2H), 7.30 (t, J = 8.0 Hz, 1H), 5.25 (p, J = 6.8 Hz, 1H), 4.76-4.79 (m, 2H), 3.70-3.73 (m, 2H), 2.80-2.89 (dt, J = 6.0 Hz, 14.0 Hz, 2H), 2.31-2.45 (m, 4H), 2.10-2.18 (dt, J = 4.8 Hz, 14.0 Hz, 2H); **^{13}C-NMR** (100 MHz, $CDCl_3$) δ 211.2, 208.8, 143.3, 128.5, 127.6, 125.8, 88.3, 76.5, 76.0, 60.9, 37.1, 35.3; **HRMS (ESI+)** m/z calculated for $C_{16}H_{18}O_2$ [M+H]$^+$: 243.1380,

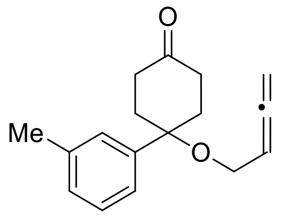

(**3c**) Yellow oil; 53% yield; **^{1}H-NMR** (400 MHz, $CDCl_3$) δ 7.20-7.24 (m, 3H), 7.06-7.10 (m, 1H), 5.24 (p, J = 6.8 Hz, 1H), 4.74-4.77 (m, 2H), 3.68-3.71 (m, 2H), 2.83 (dt, J = 6.0 Hz, 14.0 Hz, 2H), 2.37-2.42 (m, 2H), 2.35 (s, 3H), 2.28-2.33 (m, 2H), 2.12 (dt, J = 6.0 Hz, 14.0 Hz, 2H); **^{13}C-NMR** (100 MHz, $CDCl_3$) δ 211.4, 208.9, 143.3, 138.1, 128.4, 128.3, 126.6, 122.9, 88.3, 76.5, 76.0, 60.9, 37.1, 35.4, 21.6; **HRMS (ESI+)** m/z calculated for $C_{17}H_{20}O_2$ [M+Na]$^+$: 279.1356, found 279.1357.

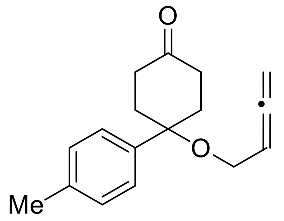

(**3d**) Yellow oil; 72% yield; **^{1}H-NMR** (400 MHz, $CDCl_3$) δ 7.31-7.34 (m, 2H), 7.17-7.19 (m, 2H), 5.25 (p, J = 6.8 Hz, 1H), 4.75-4.78 (m, 2H), 3.70-3.73 (m, 2H), 2.84 (dt, J = 6.0 Hz, 14.0 Hz, 2H), 2.38-2.44 (m, 2H), 2.35 (s, 3H), 2.29-2.36 (m, 2H), 2.13 (dt, J = 4.4 Hz, 14.0 Hz, 2H); **^{13}C-NMR** (100 MHz, $CDCl_3$) δ 211.3, 208.8, 140.3, 137.3, 129.2, 125.8, 88.4, 76.3, 75.9, 60.8, 37.1, 35.4, 20.9; **HRMS (ESI+)** m/z calculated for $C_{17}H_{20}O_2$ [M+Na]$^+$: 279.1356, found 279.1358.

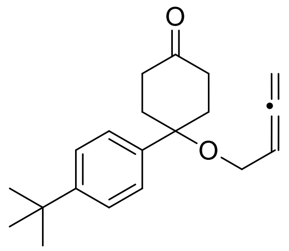

(**3e**) Yellow oil; 78% yield; **^{1}H-NMR** (400 MHz, $CDCl_3$) δ 7.34-7.39 (m, 4H), 5.26 (p, *J* = 6.8 Hz, 1H), 4.75-4.78 (m, 2H), 3.71-3.74 (m, 2H), 2.80-2.88 (dt, *J* = 6.0 Hz, 14.0 Hz, 2H), 2.38-2.45 (m, 2H), 2.30-2.34 (m, 2H), 2.09-2.17 (dt, *J* = 6.0 Hz, 14.0 Hz, 2H), 1.32 (s, 9H); **^{13}C-NMR** (100 MHz, $CDCl_3$) δ 211.4, 208.8, 150.4, 140.1, 125.5, 125.3, 88.4, 76.3, 75.9, 60.8, 37.1, 35.3, 34.4, 31.3; **HRMS (ESI+)** m/z calculated for $C_{20}H_{26}O_2$ $[M+Na]^+$: 321.1825, found 321.1826.

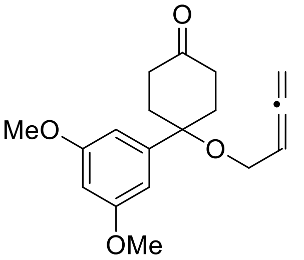

(**3f**) Yellow oil; 47% yield; **^{1}H-NMR** (400 MHz, $CDCl_3$) δ 6.59 (s, 1H), 6.58 (s, 1H), 6.39 (t, *J* = 2.0 Hz, 1H), 5.27 (p, *J* = 6.8 Hz, 1H), 4.76-4.79 (m, 2H), 3.80 (s, 6H), 3.74-3.77 (m, 2H), 2.77-2.86 (dt, *J* = 6.0 Hz, 14.0 Hz, 2H), 2.30-2.42 (m, 4H), 2.06-2.14 (dt, *J* = 6.0 Hz, 14.0 Hz, 2H); **^{13}C-NMR** (100 MHz, $CDCl_3$) δ 211.2, 208.9, 160.9, 146.1, 104.2, 99.1, 88.3, 76.7, 76.1, 61.0, 55.3, 37.1, 35.2; **HRMS (ESI+)** m/z calculated for $C_{18}H_{22}O_4$ $[M+H]^+$: 303.1591, found 303.1589.

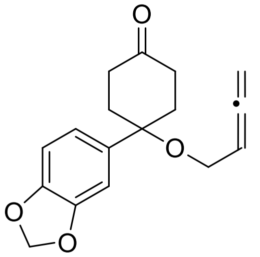

(**3g**) Yellow oil; 58% yield; **^{1}H-NMR** (400 MHz, $CDCl_3$) δ 6.97 (d, *J* = 2.0 Hz, 1H), 6.85 (dd, *J* = 2.0 Hz, 8.0 Hz, 1H), 6.78 (d, *J* = 8.0 Hz, 1H), 5.96 (s, 2H), 5.24 (p, *J* = 6.8 Hz, 1H), 4.76-4.79 (m, 2H), 3.69-3.72 (m, 2H), 2.80 (dt, *J* = 6.0 Hz, 14.0 Hz, 2H), 2.28-2.41 (m, 4H), 2.08 (dt, *J* = 6.0 Hz, 14.0 Hz, 2H); **^{13}C-NMR** (100 MHz, $CDCl_3$) δ 211.2, 208.9, 148.0, 147.0, 137.5, 119.0, 107.9, 106.7, 101.1, 88.3, 76.3, 76.0, 60.7, 37.1, 35.5; **HRMS (ESI+)** m/z calculated for $C_{17}H_{18}O_4$ $[M+Na]^+$: 309.1097, found 309.1098.

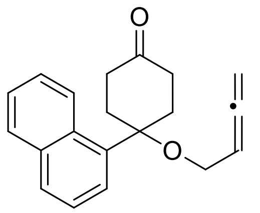

(**3h**) Yellow oil; 71% yield; **^{1}H-NMR** (400 MHz, $CDCl_3$) δ 9.02-9.04 (m, 1H), 7.86-7.89 (m, 1H), 7.81-7.83 (m, 1H), 7.48- 7.56 (m, 2H), 7.39-7.44 (m, 2H), 5.19 (p, *J* = 6.8 Hz, 1H), 4.69-4.72 (m, 2H), 3.69-3.71 (m, 2H), 2.90-3.06 (m, 4H), 2.38-2.42 (m, 2H), 2.11-2.29 (m, 2H); **^{13}C-NMR** (100 MHz, $CDCl_3$) δ 211.6, 208.9, 138.3, 134.6, 131.5, 129.4, 129.1, 126.6, 125.9, 125.6, 124.7, 124.6, 88.3, 75.9, 65.8, 61.0, 37.1, 15.3; **HRMS (ESI+)** m/z calculated for $C_{20}H_{20}O_2$ $[M+Na]^+$: 315.1356,

found 315.1351.

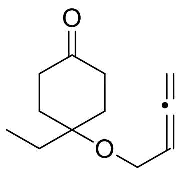

(**3i**) Yellow oil; 74% yield; **^{1}H-NMR** (400 MHz, $CDCl_3$) δ 5.27 (p, *J* = 6.8 Hz, 1H), 4.77-4.80 (m, 2H), 3.90-3.93 (m, 2H), 2.61 (dt, *J* = 6.0 Hz, 14.0 Hz, 2H), 2.12-2.20 (m, 2H), 1.61 (dt, *J* = 6.0 Hz, 14.0 Hz, 2H), 1.59 (q, *J* = 7.6 Hz, 2H), 0.90 (t, *J* = 7.6 Hz, 3H); **^{13}C-NMR** (100 MHz, $CDCl_3$) δ 212.2, 208.8, 88.6, 76.0, 74.4, 59.2, 36.7, 33.4, 29.0, 7.5; **HRMS (ESI+)** m/z calculated for $C_{12}H_{18}O_2$ $[M+H]^+$: 195.1380, found 195.1371.

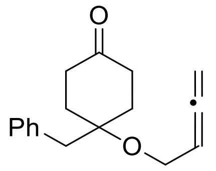

(**3j**) Yellow oil; 37% yield; **^{1}H-NMR** (400 MHz, $CDCl_3$) δ 7.22-7.31 (m, 3H), 7.15-7.17 (m, 2H), 5.34 (p, *J* = 6.8 Hz, 1H), 4.83-4.86 (m, 2H), 4.16-4.19 (m, 2H), 2.90 (s, 2H), 2.63 (dt, *J* = 6.0 Hz, 14.0 Hz, 2H), 2.09-2.20 (m, 4H), 1.71 (dt, *J* = 6.0 Hz, 14.0 Hz, 2H); **^{13}C-NMR** (100 MHz, $CDCl_3$) δ 211.6, 208.9, 136.8, 130.2, 128.2, 126.6, 88.4, 76.3, 75.2, 59.7, 42.9, 36.7, 33.5; **HRMS (ESI+)** m/z calculated for $C_{17}H_{20}O_2$ $[M+Na]^+$: 279.1356, found 279.1357.

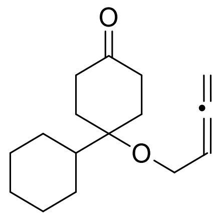

(**3k**) Yellow oil; 43% yield; **^{1}H-NMR** (400 MHz, $CDCl_3$) δ 5.27 (p, *J* = 6.8 Hz, 1H), 4.77-4.80 (m, 2H), 3.89-3.92 (m, 2H), 2.62 (dt, *J* = 6.0 Hz, 14.0 Hz, 2H), 2.16-2.21 (m, 2H), 1.96-2.03 (m, 2H), 1.76-1.82 (m, 4H), 1.58-1.74 (m, 4H), 1.08-1.27 (m, 3H), 0.96-1.05 (m, 2H); **^{13}C-NMR** (100 MHz, $CDCl_3$) δ 212.4, 208.8, 88.6, 76.9, 76.1, 58.5, 42.9, 36.8, 30.2, 27.5, 26.8, 26.5; **HRMS (ESI+)** m/z calculated for $C_{16}H_{24}O_2$ $[M+Na]^+$: 271.1669, found 271.1669.

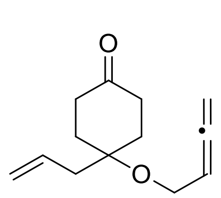

(**3l**) Yellow oil; 61% yield; **^{1}H-NMR** (400 MHz, $CDCl_3$) δ 5.76-5.86 (m, 1H), 5.26 (p, *J* = 6.8 Hz, 1H), 5.06-5.12 (m, 2H), 4.76-4.79 (m, 2H), 3.98-4.01 (m, 2H), 2.60 (dt, *J* = 6.0 Hz, 14.0 Hz, 2H), 2.31 (d, *J* = 7.2 Hz, 2H), 2.11-2.20 (m, 4H), 1.67 (dt, *J* = 6.0 Hz, 14.0 Hz, 2H); **^{13}C-NMR** (100

MHz, $CDCl_3$) δ 211.7, 208.8, 132.9, 118.3, 88.4, 76.1, 74.2, 59.5, 41.2, 36.7, 33.6; **HRMS (ESI+)** m/z calculated for $C_{13}H_{18}O_2$ $[M+H]^+$: 207.1380, found 207.1379.

4.2: Data for intermediates

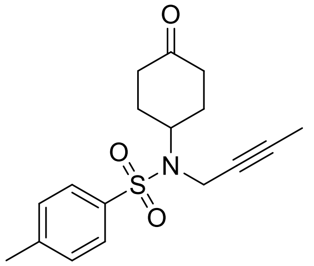

(**7**) White solid, m.p. 96-97 °C; **^{1}H-NMR** (400 MHz, $CDCl_3$) δ 7.79 (d, *J* = 8.0 Hz, 2H), 7.29 (d, *J* = 8.0 Hz, 2H), 4.16-4.24 (m, 1H), 4.04 (m, 2H), 2.43 (s, 3H), 2.37-2.41 (m, 4H), 1.96-2.05 (m, 4H), 1.66-1.67 (m, 3H); **^{13}C-NMR** (100 MHz, $CDCl_3$) δ 208.8, 143.4, 137.8, 129.4, 127.3, 80.7, 74.8, 55.5, 40.0, 33.0, 30.0, 21.5, 3.4; **HRMS (ESI+)** m/z calculated for $C_{17}H_{21}NO_3S$ $[M+Na]^+$: 342.1134, found 342.1134.

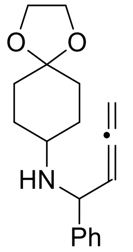

(**16**) Yellow oil; **^{1}H-NMR** (400 MHz, $CDCl_3$) δ 7.30-7.38 (m, 4H), 7.22-7.26 (m, 1H), 5.29 (q, *J* = 6.8 Hz, 1H), 4.77-4.85 (m, 2H), 4.42 (dt, *J* = 2.4 Hz, 7.2 Hz, 1H), 3.91 (s, 4H), 2.58-2.63 (m, 1H), 1.89-1.94 (m, 1H), 1.65-1.86 (m, 4H), 1.39-1.54 (m, 4H); **^{13}C-NMR** (100 MHz, $CDCl_3$) δ 207.4, 143.4, 128.4, 127.2, 127.0, 108.7, 94.8, 76.9, 64.20, 64.15, 58.8, 52.0, 32.8, 30.17, 30.15; **HRMS (ESI+)** m/z calculated for $C_{18}H_{23}NO_2$ $[M+H]^+$: 286.1802, found 286.1803.

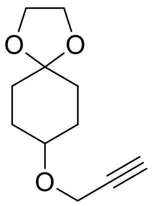

(**23a**) Colorless oil; 46% yield; **^{1}H-NMR** (400 MHz, $CDCl_3$) δ 4.13 (d, *J* = 2.4 Hz, 2H), 3.87-3.94 (m, 4H), 3.59-3.65 (m, 1H), 2.38 (t, *J* = 2.4 Hz, 1H), 1.68-1.85 (m, 6H), 1.50-1.57 (m, 2H); **^{13}C-NMR** (100 MHz, $CDCl_3$) δ 108.3, 80.3, 73.8, 73.7, 64.2, 55.2, 31.2, 28.2; **HRMS (ESI+)** m/z calculated for $C_{11}H_{16}O_3$ $[M+H]^+$: 197.1172, found 197.1168.

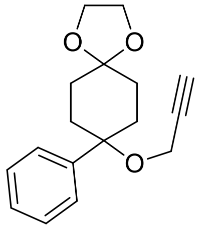

(**23b**) White semi-solid; 72% yield; **^{1}H-NMR** (400 MHz, $CDCl_3$) δ 7.46-7.48 (m, 2H), 7.36-7.39

(m, 2H), 7.27-7.31 (m, 1H), 3.96-4.03 (m, 4H), 3.79 (d, *J* = 2.4 Hz, 2H), 2.39 (t, *J* = 2.4 Hz, 1H), 2.02-2.19 (m, 6H), 1.67-1.70 (m, 2H); **^{13}C-NMR** (100 MHz, $CDCl_3$) δ 144.0, 128.4, 127.4, 125.9, 108.3, 80.7, 78.0, 73.3, 64.3, 64.1, 51.1, 33.1, 30.5; **HRMS (ESI+)** m/z calculated for $C_{17}H_{20}O_3$ $[M+Na]^+$: 295.1305, found 295.1306.

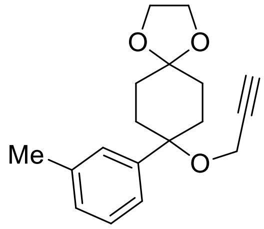

(**23c**) Pale yellow solid, m.p. 90-91 ^{o}C; 57% yield; **^{1}H-NMR** (400 MHz, $CDCl_3$) δ 7.28-7.31 (m, 3H), 7.12-7.14 (m, 1H), 3.98-4.06 (m, 4H), 3.81-3.82 (d, *J* = 2.4 Hz, 2H), 2.42 (t, *J* = 2.4 Hz, 1H), 2.40 (s, 3H), 2.02-2.21 (m, 6H), 1.68-1.72 (m, 2H); **^{13}C-NMR** (100 MHz, $CDCl_3$) δ 143.9, 138.0, 128.3, 128.1, 126.7, 123.0, 108.4, 80.8, 78.0, 73.2, 64.3, 64.2, 51.1, 33.1, 30.6, 21.6; **HRMS (ESI+)** m/z calculated for $C_{18}H_{22}O_3$ $[M+Na]^+$: 309.1461, found 309.1463.

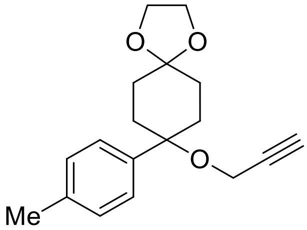

(**23d**) Pale yellow oil; 61% yield; **^{1}H-NMR** (400 MHz, $CDCl_3$) δ 7.32-7.40 (m, 4H), 7.25-7.29 (m, 1H), 5.30 (q, *J* = 6.8 Hz, 1H), 4.79-4.88 (m, 2H), 4.42-4.45 (m, 1H), 3.94 (s, 4H), 2.60-2.65 (m, 1H), 1.73-1.95 (m, 5H), 1.44-1.55 (m, 4H); **^{13}C-NMR** (100 MHz, $CDCl_3$) δ 140.9, 137.0, 129.1, 125.9, 108.5, 80.9, 77.9, 73.2, 64.3, 64.2, 51.0, 33.1, 30.6, 21.0; **HRMS (ESI+)** m/z calculated for $C_{18}H_{22}O_3$ $[M+Na]^+$: 309.1461, found 309.1463.

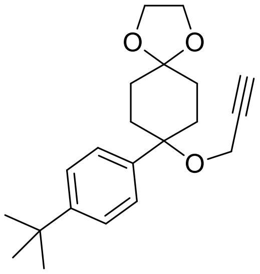

(**23e**) White solid, m.p. 99-100 ^{o}C; 48% yield; **^{1}H-NMR** (400 MHz, $CDCl_3$) δ 7.36 (s, 4H), 3.93-4.01 (m, 4H), 3.76-3.77 (d, *J* = 2.4 Hz, 2H), 2.36 (t, *J* = 2.4 Hz, 1H), 2.02-2.16 (m, 6H), 1.63-1.67 (m, 2H), 1.31 (s, 9H); **^{13}C-NMR** (100 MHz, $CDCl_3$) δ 150.2, 140.8, 125.6, 125.3, 108.5, 80.9, 77.8, 73.2, 64.3, 64.2, 51.1, 34.4, 33.1, 31.3, 30.6; **HRMS (ESI+)** m/z calculated for $C_{21}H_{28}O_3$ $[M+Na]^+$: 351.1931, found 351.1931.

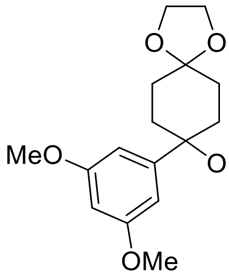

(**23f**) White solid, m.p. 94-95 °C; 60% yield; **¹H-NMR** (400 MHz, $CDCl_3$) δ 8.60 (d, *J* = 2.0 Hz, 2H), 6.36 (t, *J* = 2.0 Hz, 1H), 3.92-3.99 (m, 4H), 3.79 (d, *J* = 2.4 Hz, 2H), 3.78 (s, 6H), 2.37 (t, *J* = 2.4 Hz, 1H), 1.94-2.13 (m, 6H), 1.62-1.65 (m, 2H); **¹³C-NMR** (100 MHz, $CDCl_3$) δ 160.8, 146.8, 108.3, 104.1, 99.3, 80.7, 78.2, 73.4, 64.3, 64.1, 55.3, 51.2, 33.0, 30.5; **HRMS (ESI+)** m/z calculated for $C_{19}H_{24}O_5$ $[M+H]^+$: 333.1697, found 333.1697.

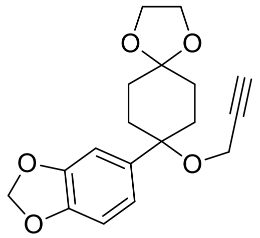

(**23g**) White solid, m.p. 91-92 °C; 69% yield; **¹H-NMR** (400 MHz, $CDCl_3$) δ 6.97 (d, *J* = 1.6 Hz, 1H), 6.88 (dd, *J* = 1.6 Hz, 8.0 Hz, 1H), 6.77 (d, *J* = 8.0 Hz, 1H), 5.95 (s, 2H), 3.92-4.00 (m, 4H), 3.75 (d, *J* = 2.8 Hz, 1H), 2.36 (t, *J* = 2.8 Hz, 1H), 1.92-2.13 (m, 6H), 1.62-1.66 (m, 2H); **¹³C-NMR** (100 MHz, $CDCl_3$) δ 147.9, 146.8, 138.1, 119.2, 108.4, 107.8, 106.8, 101.0, 80.8, 77.8, 73.3, 64.4, 64.2, 50.9, 33.3, 30.6; **HRMS (ESI+)** m/z calculated for $C_{18}H_{20}O_5$ $[M+Na]^+$: 339.1203, found 339.1203.

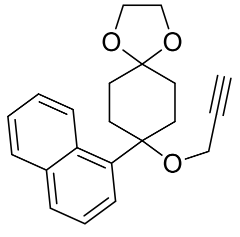

(**23h**) White solid, m.p. 97-98 °C; 51% yield; **¹H-NMR** (400 MHz, $CDCl_3$) δ 9.00 (d, *J* = 8.8 Hz, 1H), 7.84-7.87 (m, 1H), 7.79 (d, *J* = 8.0 Hz, 1H), 7.46-7.54 (m, 3H), 7.38 (t, *J* = 8.0 Hz, 1H), 3.95-4.04 (m, 4H), 3.75 (d, *J* = 2.4 Hz, 2H), 2.57-2.60 (m, 2H), 2.30-2.38 (m, 3H), 2.03-2.19 (m, 2H), 1.71-1.74 (m, 2H); **¹³C-NMR** (100 MHz, $CDCl_3$) δ 138.8, 134.6, 131.5, 129.2, 129.0, 126.7, 125.9, 125.5, 125.0, 124.7, 108.7, 80.9, 79.8, 73.3, 64.4, 64.1, 51.1, 30.6; **HRMS (ESI+)** m/z calculated for $C_{21}H_{22}O_3$ $[M+Na]^+$: 345.1461, found 345.1461.

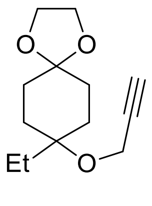

(**23i**) Colorless oil; 35% yield; **^{1}H-NMR** (400 MHz, $CDCl_3$) δ 3.98 (d, *J* = 2.4 Hz, 2H), 3.89-3.97 (m, 4H), 2.36 (t, *J* = 2.4 Hz, 1H), 1.80-1.91 (m, 4H), 1.46-1.55 (m, 6H), 0.87 (t, *J* = 7.6 Hz, 3H); **^{13}C-NMR** (100 MHz, $CDCl_3$) δ 108.9, 81.1, 75.8, 73.0, 64.2, 64.1, 49.2, 31.1, 30.2, 29.1, 7.4; **HRMS (ESI+)** m/z calculated for $C_{13}H_{20}O_3$ $[M+Na]^+$: 247.1305, found 247.1305.

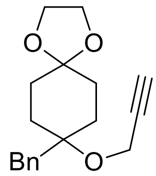

(**23j**) Yellow solid, m.p. 66-67 ^{o}C; 39% yield; **^{1}H-NMR** (400 MHz, $CDCl_3$) δ 7.26-7.30 (m, 2H), 7.21-7.23 (m, 1H), 7.17-7.19 (m, 2H), 4.20 (d, *J* = 2.4 Hz, 2H), 3.87-3.95 (m, 4H), 2.80 (s, 2H), 2.43 (t, *J* = 2.4 Hz, 1H), 1.75-1.91 (m, 4H), 1.53-1.63 (m, 4H); **^{13}C-NMR** (100 MHz, $CDCl_3$) δ 137.0, 130.3, 128.0, 126.3, 108.5, 80.7, 76.3, 73.6, 64.2, 64.1, 49.9, 42.8, 31.5, 30.3; **HRMS (ESI+)** m/z calculated for $C_{18}H_{22}O_3$ $[M+Na]^+$: 309.1461, found 309.1461.

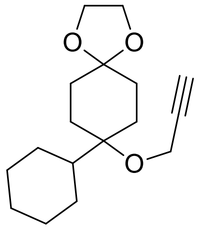

(**23k**) Colorless oil; 32% yield; **^{1}H-NMR** (400 MHz, $CDCl_3$) δ 3.97-3.98 (m, 2H), 3.90-3.96 (m, 4H), 2.35-2.36 (m, 1H), 1.86-1.94 (m, 2H), 1.65-1.79 (m, 9H), 1.45-1.54 (m, 3H), 1.11-1.24 (m, 3H), 0.97-1.06 (m, 2H); **^{13}C-NMR** (100 MHz, $CDCl_3$) δ 108.9, 81.0, 78.3, 73.0, 64.2, 64.1, 48.7, 43.0, 30.1, 27.8, 27.5, 26.9, 26.6; **HRMS (ESI+)** m/z calculated for $C_{17}H_{26}O_3$ $[M+Na]^+$: 301.1774, found 301.1774.

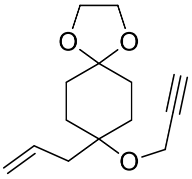

(**23l**) Colorless oil; 41% yield; **^{1}H-NMR** (400 MHz, $CDCl_3$) δ 5.77-5.87 (m, 1H), 5.01-5.08 (m, 2H), 4.04-4.05 (m, 2H), 3.87-3.95 (m, 4H), 2.36 (dt, *J* = 0.8 Hz, 2.4 Hz, 1H), 2.25 (dd, *J* = 0.8 Hz, 7.2 Hz, 2H), 1.76-1.90 (m, 4H), 1.46-1.58 (m, 4H); **^{13}C-NMR** (100 MHz, $CDCl_3$) δ 133.3, 117.8, 108.6, 80.9, 75.6, 73.2, 64.2, 64.1, 49.5, 41.4, 31.4, 30.1; **HRMS (ESI+)** m/z calculated for $C_{14}H_{20}O_3$ $[M+Na]^+$: 259.1305, found 259.1308.

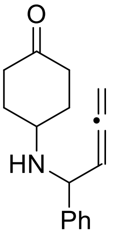

(**17**) Yellow oil; **¹H-NMR** (400 MHz, $CDCl_3$) δ 7.30-7.36 (m, 4H), 7.22-7.27 (m, 1H), 5.29 (q, *J* = 6.4 Hz, 1H), 4.78-4.87 (m, 2H), 4.39 (dt, *J* = 2.4 Hz, 6.8 Hz, 1H), 2.96-3.02 (m, 1H), 2.41-2.51 (m, 2H), 2.18-2.29 (m, 2H), 1.97-2.14 (m, 2H), 1.61-1.75 (m, 2H), 1.47 (s, 1H); **¹³C-NMR** (100 MHz, $CDCl_3$) δ 211.4, 207.4, 143.2, 128.5, 127.4, 126.9, 94.5, 77.2, 59.0, 50.9, 38.51, 38.48, 32.1, 32.0; **HRMS (ESI+)** m/z calculated for $C_{16}H_{19}NO$ $[M+H]^+$: 242.1539, found 242.1539.

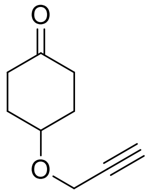

(**24a**) Colorless oil; quantitative yield; **¹H-NMR** (400 MHz, $CDCl_3$) δ 4.23 (m, 2H), 3.94-3.96 (m, 1H), 2.53-2.61 (m, 2H), 2.44 (t, *J* = 2.4 Hz, 1H), 2.23-2.29 (m, 2H), 2.06-2.14 (m, 2H), 1.91-1.99 (m, 2H); **¹³C-NMR** (100 MHz, $CDCl_3$) δ 210.8, 79.8, 74.3, 72.0, 55.6, 37.0, 30.3; **HRMS (ESI+)** m/z calculated for $C_9H_{12}O_2$ $[M+H]^+$: 153.0910, found 153.0911.

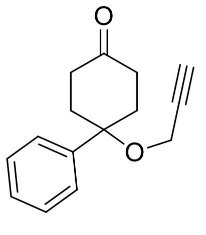

(**24b**) White solid, m.p. 47-48 °C; quantitative yield; **¹H-NMR** (400 MHz, $CDCl_3$) δ 7.44-7.47 (m, 2H), 7.37-7.41 (m, 2H), 7.30-7.34 (m, 1H), 3.87 (d, *J* = 2.4 Hz, 2H), 2.94 (dt, *J* = 6.0 Hz, 14.4 Hz, 2H), 2.40-2.48 (m, 2H), 2.41 (t, *J* = 2.4 Hz, 1H), 2.32-2.37 (m, 2H), 2.17 (dt, *J* = 4.8 Hz, 13.6 Hz, 2H); **¹³C-NMR** (100 MHz, $CDCl_3$) δ 210.9, 142.4, 128.7, 127.9, 125.8, 80.3, 77.6, 73.7, 51.5, 37.1, 35.3; **HRMS (ESI+)** m/z calculated for $C_{15}H_{16}O_2$ $[M+Na]^+$: 251.1043, found 251.1045.

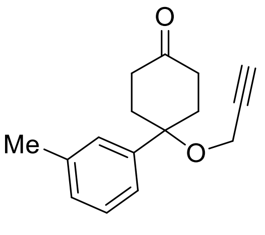

(**24c**) Pale yellow oil; 87% yield; **¹H-NMR** (400 MHz, $CDCl_3$) δ 7.19-7.24 (m, 3H), 7.08-7.10 (d, *J* = 7.2 Hz, 1H), 3.84 (d, *J* = 2.4 Hz, 2H), 2.89 (dt, *J* = 2.0 Hz, 14.4 Hz, 2H), 2.37-2.42 (m, 2H), 2.38 (t, *J* = 2.4 Hz, 1H), 2.34 (s, 3H), 2.27-2.32 (m, 2H), 2.11 (dt, *J* = 4.0 Hz, 14.4 Hz, 2H); **¹³C-NMR** (100 MHz, $CDCl_3$) δ 211.0, 142.4, 138.3, 128.7, 128.6, 126.6, 122.9, 80.5, 77.6, 73.7, 51.5, 37.1, 35.3,

21.6; **HRMS (ESI+)** m/z calculated for $C_{16}H_{18}O_2$ [M+Na]+ : 265.1199, found 265.1200.

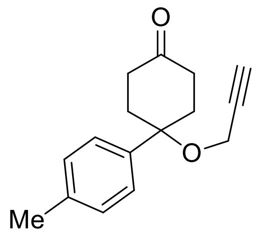

(**24d**) Yellow oil; 82% yield; **^{1}H-NMR** (400 MHz, $CDCl_3$) δ 7.32-7.35 (m, 2H), 7.18-7.20 (m, 2H), 3.86 (d, *J* = 2.4 Hz, 2H), 2.92 (dt, *J* = 6.0 Hz, 14.4 Hz, 2H), 2.39-2.46 (m, 2H), 2.40 (t, *J* = 2.4 Hz, 1H), 2.35 (s, 3H), 2.28-2.35 (m, 2H), 2.14 (dt, *J* = 4.8 Hz, 14.0 Hz, 2H); **^{13}C-NMR** (100 MHz, $CDCl_3$) δ 211.0, 139.4, 137.7, 129.4, 125.8, 80.5, 77.5, 73.6, 51.4, 37.1, 35.4, 21.0; **HRMS (ESI+)** m/z calculated for $C_{16}H_{18}O_2$ [M+Na]+ : 265.1199, found 265.1200.

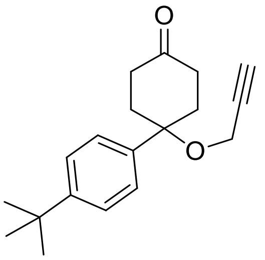

(**24e**) Colorless oil; 91% yield; **^{1}H-NMR** (400 MHz, $CDCl_3$) δ 7.35-7.41 (m, 4H), 3.87 (d, *J* = 2.4 Hz, 2H), 2.93 (dt, *J* = 6.0 Hz, 14.4 Hz, 2H), 2.41-2.47 (m, 2H), 2.40 (t, *J* = 2.4 Hz, 1H), 2.31-2.36 (m, 2H), 2.16 (dt, *J* = 4.8 Hz, 14.0 Hz, 2H), 1.32 (s, 9H); **^{13}C-NMR** (100 MHz, $CDCl_3$) δ 211.2, 150.9, 139.3, 125.6, 80.6, 77.5, 73.6, 51.5, 37.2, 35.4, 34.5, 31.3; **HRMS (ESI+)** m/z calculated for $C_{19}H_{24}O_2$ [M+Na]+ : 307.1669, found 307.1670.

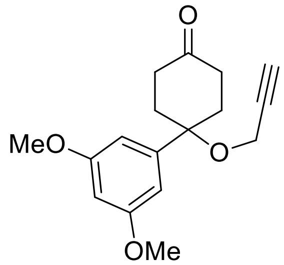

(24f) Yellow oil; 87% yield; **^{1}H-NMR** (400 MHz, $CDCl_3$) δ 6.59 (d, *J* = 2.4 Hz, 2H), 6.40 (t, *J* = 2.4 Hz, 1H), 3.90 (d, *J* = 2.4 Hz, 2H), 3.79 (s, 6H), 2.89 (dt, *J* = 6.0 Hz, 14.4 Hz, 2H), 2.29-2.45 (m, 4H), 2.42 (t, *J* = 2.4 Hz, 1H), 2.11 (dt, *J* = 4.4 Hz, 14.0 Hz, 2H); **^{13}C-NMR** (100 MHz, $CDCl_3$) δ 210.8, 161.0, 145.2, 104.2, 99.4, 80.4, 77.8, 73.8, 55.3, 51.7, 37.0, 35.2; **HRMS (ESI+)** m/z calculated for $C_{17}H_{20}O_4$ [M+Na]+ : 311.1254, found 311.1254.

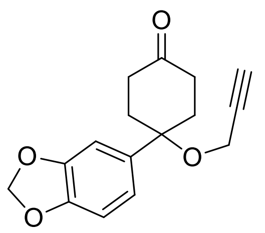

(**24g**) White solid, m.p. 107-108 °C; 89% yield; **^{1}H-NMR** (400 MHz, $CDCl_3$) δ 6.98 (d, *J* = 2.0 Hz, 1H), 6.88 (dd, *J* = 2.0 Hz, 8.0 Hz, 1H), 6.80 (d, *J* = 8.0 Hz, 1H), 5.98 (s, 2H), 3.87 (d, *J* = 2.4 Hz, 2H), 2.90 (dt, *J* = 2.0 Hz, 14.0 Hz, 2H), 2.30-2.44 (m, 4H), 2.41 (t, *J* = 2.4 Hz, 1H), 2.10 (dt, *J* = 4.8 Hz, 14.0 Hz, 2H); **^{13}C-NMR** (100 MHz, $CDCl_3$) δ 211.0, 148.2, 147.3, 136.5, 119.3, 108.1, 106.7, 101.2, 80.4, 77.4, 73.8, 51.4, 37.1, 35.5; **HRMS (ESI+)** m/z calculated for $C_{16}H_{16}O_4$ $[M+Na]^+$: 295.0941, found 295.0941.

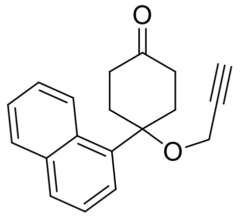

(**24h**) Colorless oil; 87% yield; **^{1}H-NMR** (400 MHz, $CDCl_3$) δ 8.98 (d, *J* = 8.8 Hz, 1H), 7.87-7.89 (m, 1H), 7.84 (d, *J* = 7.6 Hz, 1H), 7.49-7.58 (m, 2H), 7.39-7.46 (m, 2H), 3.87 (d, *J* = 2.4 Hz, 2H), 3.12 (dt, *J* = 2.0 Hz, 14.4 Hz, 2H), 2.91-2.95 (m, 2H), 2.39-2.43 (m, 2H), 2.36 (t, *J* = 2.4 Hz, 2.16-2.30 (m, 2H); **^{13}C-NMR** (100 MHz, $CDCl_3$) δ 211.3, 137.4, 134.7, 131.3, 129.8, 129.2, 126.3, 126.2, 125.7, 124.9, 124.7, 80.4, 79.4, 73.8, 51.6, 37.2, 30.9; **HRMS (ESI+)** m/z calculated for $C_{19}H_{18}O_2$ $[M+Na]^+$: 301.1199, found 301.1200.

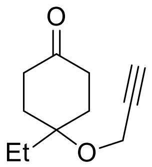

(**24i**) Colorless oil; 70% yield; **^{1}H-NMR** (400 MHz, $CDCl_3$) δ 4.06-4.13 (m, 2H), 2.62-2.71 (m, 2H), 2.39-2.41 (m, 1H), 2.09-2.25 (m, 4H), 1.53-1.67 (m, 4H), 0.88-0.93 (m, 3H); **^{13}C-NMR** (100 MHz, $CDCl_3$) δ 211.8, 80.6, 75.5, 73.5, 49.6, 36.7, 33.4, 28.8, 7.5; **HRMS (ESI+)** m/z calculated for $C_{11}H_{16}O_2$ $[M+H]^+$: 181.1223, found 181.1225.

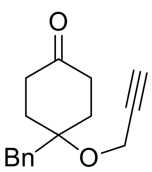

(**24j**) White solid, m.p. 100-101 °C; 89% yield; **^{1}H-NMR** (400 MHz, $CDCl_3$) δ 7.22-7.32 (m, 3H), 7.16-7.18 (m, 2H), 4.34 (d, *J* = 2.4 Hz, 2H), 2.91 (s, 2H), 2.69 (dt, *J* = 2.0 Hz, 14.4 Hz, 2H), 2.47 (t, *J* = 2.4 Hz, 1H), 2.09-2.21 (m, 4H), 1.73 (dt, *J* = 4.8 Hz, 14.0 Hz, 2H); **^{13}C-NMR** (100 MHz, $CDCl_3$)

δ 211.2, 136.4, 130.2, 128.3, 126.7, 80.3, 76.2, 74.0, 50.3, 42.8, 36.7, 33.4; **HRMS (ESI+)** m/z calculated for $C_{16}H_{18}O_2$ $[M+H]^+$: 243.1380, found 243.1381.

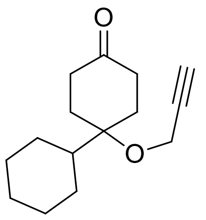

(**24k**) Colorless oil; 85% yield; **^{1}H-NMR** (400 MHz, $CDCl_3$) δ 4.08-4.09 (dd, *J* = 0.8 Hz, 2.4 Hz, 2H), 2.67 (dt, *J* = 6.0 Hz, 14.0 Hz, 2H), 2.40 (dt, *J* = 0.8 Hz, 2.4 Hz, 1H), 2.16-2.21 (m, 2H), 1.96-2.01 (m, 2H), 1.55-1.83 (m, 8H), 0.95-1.27 (m, 5H); **^{13}C-NMR** (100 MHz, $CDCl_3$) δ 212.0, 80.5, 78.0, 73.4, 49.1, 42.7, 36.7, 30.2, 27.5, 26.7, 26.4; **HRMS (ESI+)** m/z calculated for $C_{15}H_{22}O_2$ $[M+Na]^+$: 257.1512, found 257.1513.

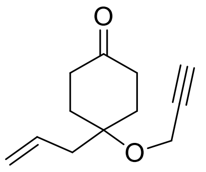

(**24l**) Colorless oil; 88% yield; **^{1}H-NMR** (400 MHz, $CDCl_3$) δ 5.77-5.88 (m, 1H), 5.07-5.15 (m, 2H), 4.18 (d, *J* = 2.4 Hz, 2H), 2.63-2.72 (m, 2H), 2.42 (t, *J* = 2.4 Hz, 1H), 2.35 (dt, *J* = 1.2 Hz, 7.2 Hz, 2H), 2.13-2.23 (m, 4H), 1.65-1.74 (m, 2H); **^{13}C-NMR** (100 MHz, $CDCl_3$) δ 211.4, 132.6, 118.6, 80.5, 75.3, 73.7, 50.0, 41.1, 36.7, 33.7; **HRMS (ESI+)** m/z calculated for $C_{12}H_{16}O_2$ $[M+Na]^+$: 215.1043, found 215.1044.

4.3: Synthesis of *C*-tethered substrate S5a

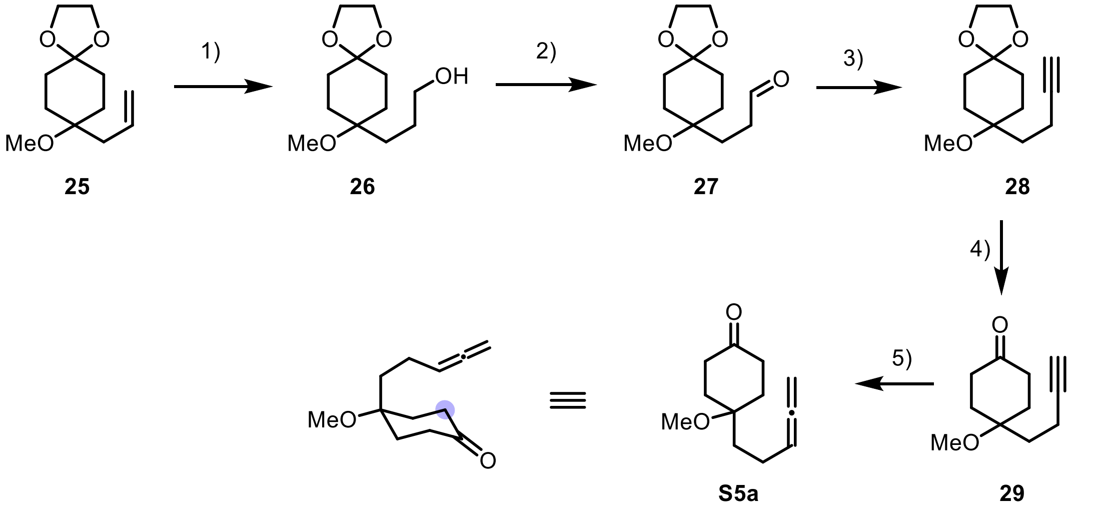

Reaction conditions: 1) BH_3-Me_2S, 3M NaOH (aq.), H_2O_2, THF, 0 °C~rt; 2) Dess-Martin periodinane, CH_2Cl_2, rt; 3) dimethyl (1-diazo-2-oxopropyl)phosphonate, K_2CO_3, CH_3OH, rt; 4) pyridinium *p*-toluenesulfonate, acetone, H_2O, 80 °C; 5) paraformaldehyde, CuI, Cy_2NH, 1,4-dioxane, 110 °C.

To a solution of 8-allyl-8-methoxy-1,4-dioxaspiro[4.5]decane **25**[3] (5.73 g, 27.0 mmol) in anhydrous THF (80 mL), BH_3-Me_2S (7.7 mL, 81.0 mmol) was added slowly at 0 °C under argon atmosphere. The mixture was stirred at room temperature for 1 h. And then the reaction mixture was cooled to 0 °C, 3M NaOH (aq.) (54 mL, 162.0 mmol) and 30% H_2O_2 (17 mL) were added dropwise and stirred for another 2 h. The mixture was extracted with $CHCl_3$ (3×50 mL), the combined organic layers were washed with brine, dried over anhydrous Na_2SO_4 and concentrated under vacuum. The crude residue was purified by column chromatography to afford 3-(8-methoxy-1,4-dioxaspiro[4.5]decan-8-yl)propan-1-ol **26** (5.29 g, 85%).

3-(8-Methoxy-1,4-dioxaspiro[4.5]decan-8-yl)propan-1-ol **26** (2.46 g, 11.0 mmol) was added slowly into a solution of Dess-Martin periodinane (5.6 g, 13.2 mmol) in DCM (50 mL), and the mixture was stirred at room temperature for 1.5 h. The mixture was diluted with Et_2O and added 5% NaOH (aq.), stirred for 10 min, extracted with Et_2O (3×30 mL), the combined organic layers were washed with 5% NaOH (aq.), water and brine, organic phase was dried over anhydrous Na_2SO_4, filtered and evaporated. The residue was purified by column chromatography to afford 3-(8-methoxy-1,4-dioxaspiro[4.5]decan-8-yl)propanal **27** (1.86 g, 74%).

Dimethyl (1-diazo-2-oxopropyl)phosphonate (1.38 g, 7.2 mmol) was added into a solution of K_2CO_3 (1.66 g, 12.0 mmol) and **27** (1.37 g, 6.0 mmol) in 20 mL CH_3OH. The reaction mixture was stirred at rt for 8 h. After all the starting materials was consumed, the reaction mixture was diluted with Et_2O, and washed with 5% $NaHCO_3$ (aq.), extracted with Et_2O (3×20 mL), the combined organic layers were dried over anhydrous Na_2SO_4, filtered and evaporated. The residue was purified by column chromatography to afford 8-(but-3-yn-1-yl)-8-methoxy-1,4-dioxaspiro[4.5]decane **28** as a white solid (1.18 g, 88%).

8-(But-3-yn-1-yl)-8-methoxy-1,4-dioxaspiro[4.5]decane **28** (1.68 g, 7.5 mmol) and pyridinium *p*-

toluenesulfonate (377 mg, 1.5 mmol) were dissolved in acetone (16 mL) and water (8 mL). The mixture was stirred at 80 °C for 8 h. Acetone was removed under vacuum, and the aqueous phase was extracted with EtOAc (3×20 mL). The combined organic layers were dried over Na_2SO_4, filtered and evaporated. The crude residue was purified by column chromatography to afford 4-(but-3-yn-1-yl)-4-methoxycyclohexanone **29** (1.08 g, 80%).

A flask was charged with 4-(but-3-yn-1-yl)-4-methoxycyclohexanone **29** (1.08 g, 6.0 mmol), paraformaldehyde (0.45 g, 15.0 mmol) and CuI (0.57 g, 3.0 mmol), the flask was evacuated and filled with argon (3~4 times). Anhydrous 1,4-dioxane (24 mL) was added, followed by the addition of Cy_2NH (2.15 mL, 10.8 mmol). The mixture was stirred at 110 °C under argon atmosphere for 4 h. The reaction was cooled to room temperature and diluted with saturated aqueous solution of NH_4Cl. The phases were separated and the aqueous phase was extracted with EtOAc (3×15 mL). The combined organic layers were dried over anhydrous Na_2SO_4, filtered and evaporated. The residue was purified by column chromatography to afford 4-methoxy-4-(penta-3,4-dien-1-yl)cyclohexanone **S5a** (0.82 g, 70%).

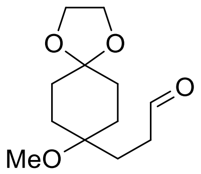

(**27**) White solid, m.p. 57-58 °C; **^{1}H-NMR** (400 MHz, $CDCl_3$) δ 9.76-9.81 (m, 1H), 3.87-3.95 (m, 4H), 3.07-3.09 (m, 3H), 2.43-2.48 (m, 2H), 1.73-1.79 (m, 6H), 1.43-1.57 (m, 4H); **^{13}C-NMR** (100 MHz, $CDCl_3$) δ 202.1, 108.6, 73.1, 64.2, 64.1, 48.4, 37.9, 31.1, 30.1, 27.6; **HRMS (ESI+)** m/z calculated for $C_{12}H_{20}O_4$ $[M+Na]^+$: 251.1254, found 251.1255.

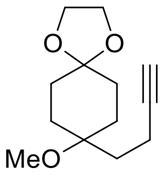

(**28**) White solid, m.p. 57-58 °C; **^{1}H-NMR** (400 MHz, $CDCl_3$) δ 3.89-3.96 (m, 4H), 3.13 (s, 3H), 2.16-2.20 (m, 2H), 1.93 (t, J = 2.4 Hz, 1H), 1.70-1.81 (m, 6H), 1.44-1.55 (m, 4H); **^{13}C-NMR** (100 MHz, $CDCl_3$) δ 108.7, 84.8, 73.3, 68.0, 64.3, 64.2, 48.5, 35.0, 31.0, 30.1, 12.3; **HRMS (ESI+)** m/z calculated for $C_{13}H_{20}O_3$ $[M+H]^+$: 225.1485, found 225.1487.

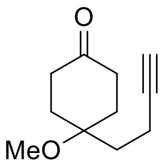

(**29**) White solid, m.p. 79-80 °C; **^{1}H-NMR** (400 MHz, $CDCl_3$) δ 3.23 (s, 3H), 2.55 (dt, J = 6.0 Hz, 14.4 Hz, 2H), 2.09-2.25 (m, 6H), 1.95 (t, J = 2.4 Hz, 1H), 1.78-1.82 (m, 2H), 1.63 (dt, J = 4.4 Hz, 14.0 Hz, 2H); **^{13}C-NMR** (100 MHz, $CDCl_3$) δ 211.4, 84.2, 73.1, 68.4, 48.8, 36.5, 34.6, 33.2, 12.4; **HRMS (ESI+)** m/z calculated for $C_{11}H_{16}O_2$ $[M+H]^+$: 181.1223, found 181.1224.

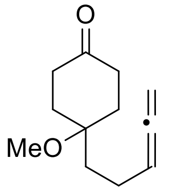

(**S5a**) Pale yellow oil; **^{1}H-NMR** (400 MHz, $CDCl_3$) δ 5.14 (p, *J* = 6.8 Hz, 1H), 4.68 (p, *J* = 3.6 Hz, 2H), 3.22 (s, 3H), 2.56 (dt, *J* = 1.6 Hz, 14.0 Hz, 2H), 2.08-2.24 (m, 4H), 1.98-2.05 (m, 2H), 1.58-1.66 (m, 4H); **^{13}C-NMR** (100 MHz, $CDCl_3$) δ 211.9, 208.3, 89.8, 75.5, 73.4, 48.7, 36.6, 34.7, 33.4, 21.5; **HRMS (ESI+)** m/z calculated for $C_{12}H_{18}O_2$ $[M+Na]^+$: 217.1199, found 217.1200.

5. Synthesis of Bicyclic Structures

Synthesis of 2a is representative

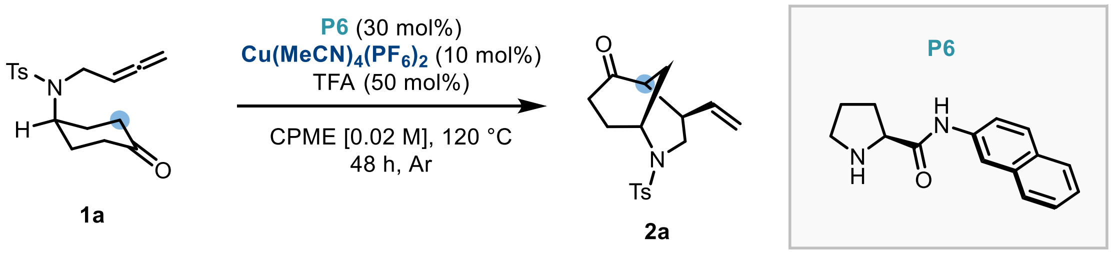

Allene **1a** (96 mg, 0.3 mmol), **P6** (21.6 mg, 0.09 mmol), $Cu(CH_3CN)_4PF_6$ (11 mg, 0.03 mmol) were added into a sealed tube, the tube was evacuated and filled with argon (3 times), anhydrous CPME (15 mL) and TFA (11 μL, 0.15 mmol) were then injected into the tube. The mixture was stirred at 120 °C for 48 h. The mixture was then cooled to room temperature, diluted with EtOAc and filtered through celite (eluting with additional 5 mL EtOAc). The filtrate was evaporated in vacuum and purified by column chromatography on silica gel to yield the desired cyclized product **2a** (81% yield, 91% ee).

Data for **2a**

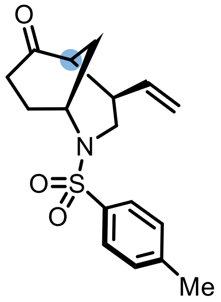

Pale yellow solid, m.p. 106-107 °C, 96:4 er; **[α]$_D^{25}$** = -43.1 (c = 0.85, $CHCl_3$); **^{1}H-NMR** (400 MHz, $CDCl_3$) δ 7.69 (d, *J* = 8.0 Hz, 2H), 7.32 (d, *J* = 8.0 Hz, 2H), 5.76-5.85 (m, 1H), 5.13-5.24 (m, 2H), 4.23-4.31 (m, 1H), 3.45 (dd, *J* = 4.0 Hz, 12.8 Hz, 1H), 3.17 (dd, *J* = 4.0 Hz, 12.8 Hz, 1H), 2.59-2.67 (m, 1H), 2.34-2.52 (m, 3H), 2.43 (s, 3H), 2.14-2.18 (m, 1H), 1.90-1.99 (m, 1H), 1.75-1.82 (m, 2H); **^{13}C-NMR** (100 MHz, $CDCl_3$) δ 213.0, 143.6, 137.0, 136.0, 129.8, 127.2, 117.0, 46.9, 46.5, 42.8, 40.1, 36.2, 26.8, 26.2, 21.5; **HRMS (ESI+)** m/z calculated for $C_{17}H_{22}NO_3S$ $[M+H]^+$: 320.1315, found 320.1317; **HPLC** (Chiralpak IA, hexane/isopropanol 90:10, 1.0 mL/min, λ=230 nm) tR = 21.9 min (major), 39.7 min (minor).

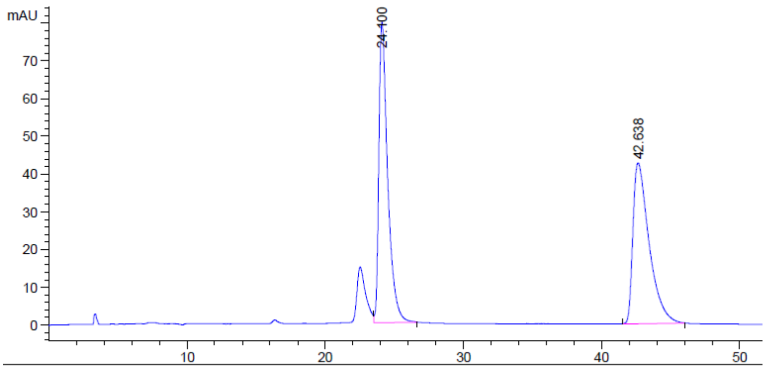

#	RetTime	Width	Area	Height	Area%
1	24.100	0.6579	3533.90601	79.60555	50.4495
2	42.638	1.1854	3470.93774	42.65058	49.5505

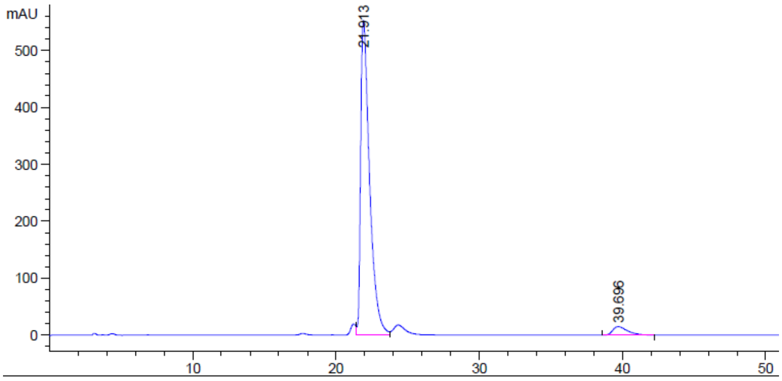

#	RetTime	Width	Area	Height	Area%
1	21.913	0.6455	2.39191e4	552.08978	95.6197
2	39.696	1.0701	1095.73315	14.96725	4.3803

Data for **2b**

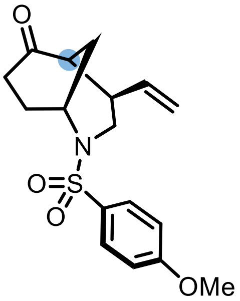

Pale yellow solid, m.p. 112-113 °C, 96:4 er, **[α]$_D^{25}$** = -42.7 (c = 0.55, $CHCl_3$); **^{1}H-NMR** (400 MHz, $CDCl_3$) δ 7.75 (d, *J* = 8.8 Hz, 2H), 6.99 (d, *J* = 8.8 Hz, 2H), 5.77-5.85 (m, 1H), 5.24 (dt, *J* = 1.2 Hz, 17.2 Hz, 1H), 5.17 (dt, *J* = 1.2 Hz, 17.2 Hz, 1H), 4.23-4.30 (m, 1H), 3.87 (s, 3H), 3.43 (dd, *J* = 4.4 Hz, 12.8 Hz, 1H), 3.17 (dd, *J* = 4.4 Hz, 12.8 Hz, 1H), 2.59-2.67 (m, 1H), 2.35-2.53 (m, 3H), 2.14-2.20 (m, 1H), 1.91-2.00 (m, 1H), 1.78-1.85 (m, 2H); **^{13}C-NMR** (100 MHz, $CDCl_3$) δ 213.0, 163.0, 137.1, 130.6, 129.4, 117.0, 114.3, 55.6, 46.8, 46.6, 42.7, 40.1, 36.3, 26.8, 26.2; **HRMS (ESI+)** m/z calculated for $C_{17}H_{22}NO_4S$ $[M+H]^+$: 336.1264, found 336.1266; **HPLC** (Chiralpak AD-H, hexane/isopropanol 90:10, 1.0 mL/min, λ=220 nm) tR = 37.3 min (major), 52.1 min (minor).

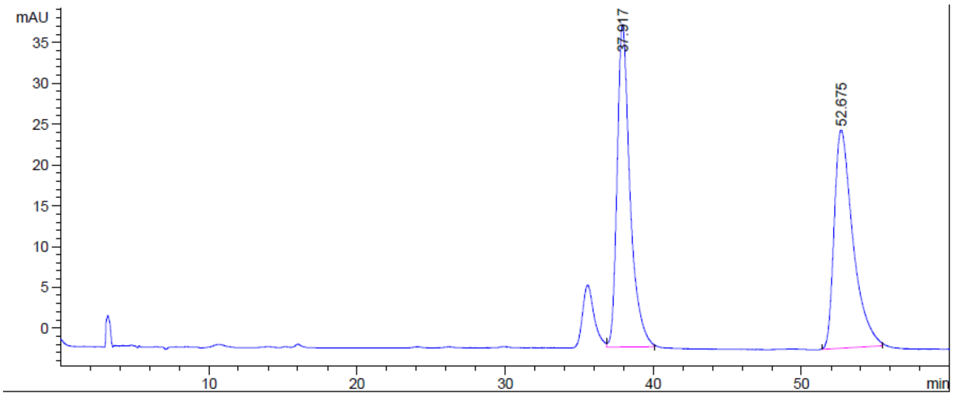

#	RetTime	Width	Area	Height	Area%
1	37.917	0.8979	2392.32495	39.54152	50.4455
2	52.675	1.2819	2350.07446	26.72606	49.5545

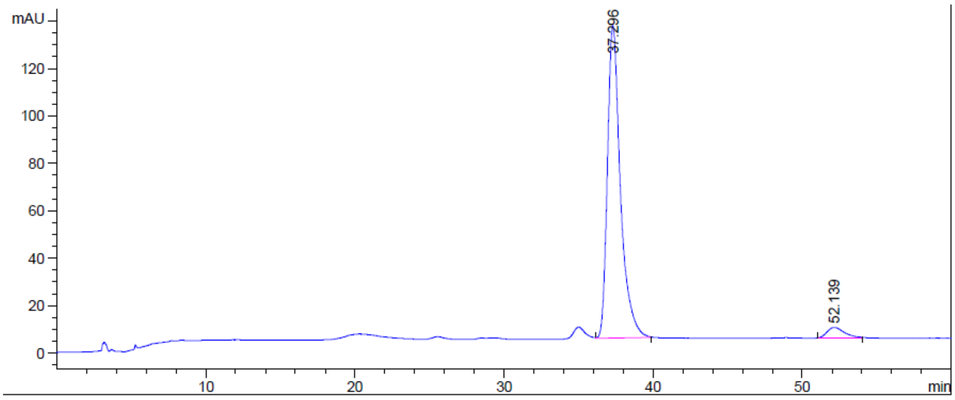

#	RetTime	Width	Area	Height	Area%
1	37.296	0.9060	7976.48877	131.80316	95.7369
2	52.139	1.0067	355.18863	4.44308	4.2631

Data for **2c**

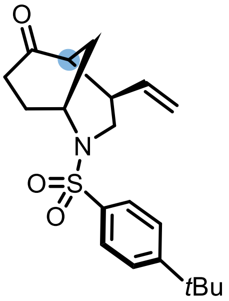

(**2c**) White solid, m.p. 101-102 °C, 94:6 er; **[α]$_D$^{25}** = -31.7 (c = 0.83, $CHCl_3$); **^{1}H-NMR** (400 MHz, $CDCl_3$) δ 7.70-7.74 (m, 2H), 7.50-7.53 (m, 2H), 5.75-5.84 (m, 1H), 5.12-5.23 (m, 2H), 4.25-4.32 (m, 1H), 3.45 (dd, *J* = 4.4 Hz, 12.8 Hz, 1H), 3.21 (dd, *J* = 4.8 Hz, 12.8 Hz, 1H), 2.59-2.68 (m, 1H), 2.35-2.55 (m, 3H), 2.15-2.21 (m, 1H), 1.92-2.01 (m, 1H), 1.78-1.88 (m, 2H), 1.34 (s, 9H); **^{13}C-NMR** (100 MHz, $CDCl_3$) δ 213.0, 156.6, 137.1, 135.9, 127.1, 126.2, 116.9, 46.9, 46.6, 42.7, 40.2, 36.2, 35.1, 31.1, 27.0, 26.2; **HRMS (ESI+)** m/z calculated for $C_{20}H_{27}NO_3S$ $[M+H]^+$: 362.1784, found 362.1785; **HPLC** (Chiralpak IA, hexane/isopropanol 90:10, 1.0 mL/min, λ=220 nm) tR = 14.7 min (major), 18.4 min (minor).

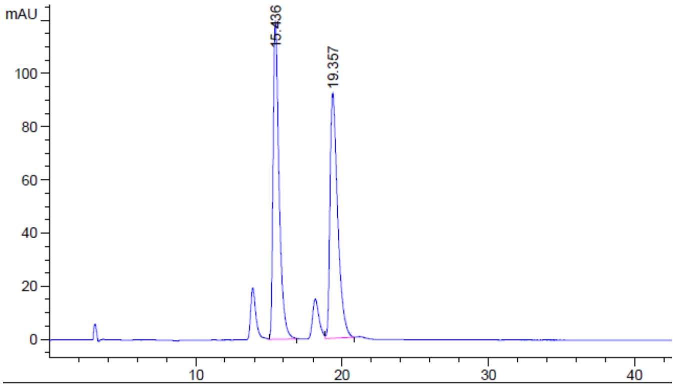

#	RetTime	Width	Area	Height	Area%
1	15.436	0.4259	3408.51392	119.80519	50.4423
2	19.357	0.5519	3348.73511	92.15520	49.5577

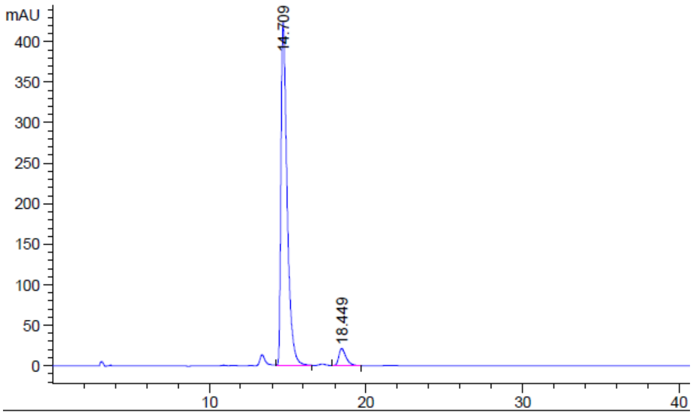

#	RetTime	Width	Area	Height	Area%
1	14.709	0.4125	1.16279e4	423.33237	94.3044
2	18.449	0.4937	702.27667	21.46550	5.6956

Data for **2d**

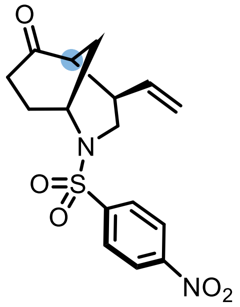

Yellow solid, m.p. 110-111 °C, 94:6 er; **[α]$_D^{25}$** = -41.0 (c = 0.84, $CHCl_3$); **^{1}H-NMR** (400 MHz, $CDCl_3$) δ 8.40 (m, 2H), 8.02 (m, 2H), 5.75-5.83 (m, 1H), 5.17-5.26 (m, 2H), 4.30-4.38 (m, 1H), 3.55 (dd, *J* = 4.0 Hz, 12.8 Hz, 1H), 3.23 (dd, *J* = 4.8 Hz, 12.8 Hz, 1H), 2.63-2.73 (m, 1H), 2.43-2.50 (m, 3H), 2.16-2.21 (m, 1H), 2.02-2.11 (m, 1H), 1.88 (dt, *J* = 3.2 Hz, 14.0 Hz, 1H), 1.72-1.79 (m, 1H); **^{13}C-NMR** (100 MHz, $CDCl_3$) δ 212.2, 150.1, 145.1, 136..5, 128.4, 124.5, 117.4, 47.3, 46.2, 43.0, 39.9, 36.2, 26.9, 26.0; **HRMS (ESI+)** m/z calculated for $C_{16}H_{18}N_2O_5S$ $[M+Na]^+$: 373.0829, found 373.0829; **HPLC** (Chiralpak AD-H, hexane/isopropanol 80:20, 1.0 mL/min, λ=220 nm) tR = 21.7 min (major), 39.3 min (minor).

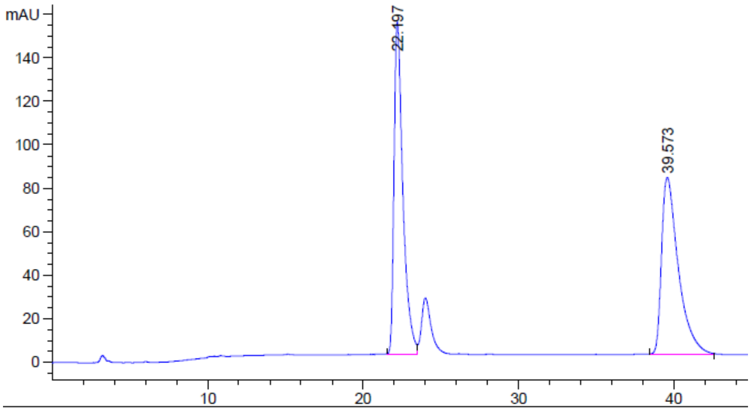

#	RetTime	Width	Area	Height	Area%
1	22.197	0.5929	6057.24414	152.66318	49.8361
2	39.573	1.1147	6097.08252	81.32496	50.1639

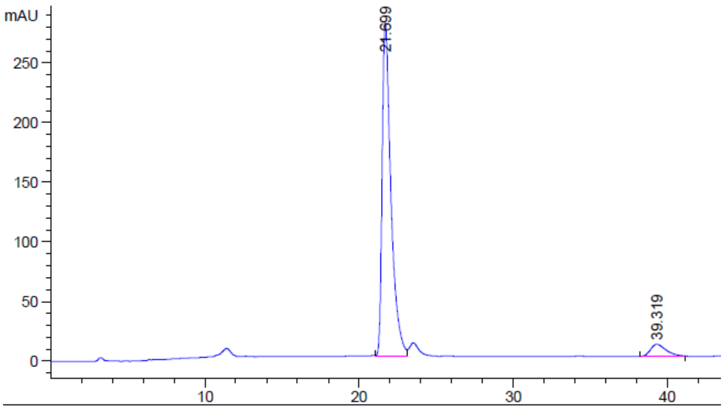

#	RetTime	Width	Area	Height	Area%
1	21.699	0.5938	1.10868e4	278.89624	93.9070
2	39.319	1.0263	719.34381	10.18103	6.0930

Data for **2e**

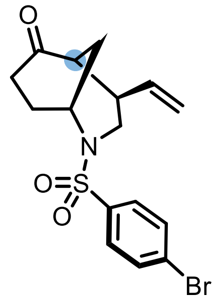

Yellow solid, m.p. 117-118 °C, 94:6 er; **[α]$_D$^{25}** = -36.6 (c = 0.56, $CHCl_3$); **^{1}H-NMR** (400 MHz, $CDCl_3$) δ 7.64-7.68 (m, 4H), 5.75-5.84 (m, 1H), 5.16-5.25 (m, 2H), 4.24-4.31 (m, 1H), 3.47 (dd, *J* = 4.0 Hz, 12.8 Hz, 1H), 3.19 (dd, *J* =4.8 Hz, 12.8 Hz, 1H), 2.60-2.69 (m, 1H), 2.37-2.54 (m, 3H), 2.13-2.22 (m, 1H), 1.95-2.02 (m, 1H), 1.77-1.85 (m, 2H); **^{13}C-NMR** (100 MHz, $CDCl_3$) δ 212.6, 138.1, 136.8, 132.5, 128.7, 127.8, 117.2, 47.1, 46.4, 42.9, 40.0, 36.2, 26.9, 26.1; **HRMS (ESI+)** m/z calculated for $C_{16}H_{18}BrNO_3S$ $[M+H]^+$: 384.0264, found 384.0264; **HPLC** (Chiralpak IA, hexane/isopropanol 90:10, 1.0 mL/min, λ=220 nm) tR = 21.9 min (major), 48.0 min (minor).

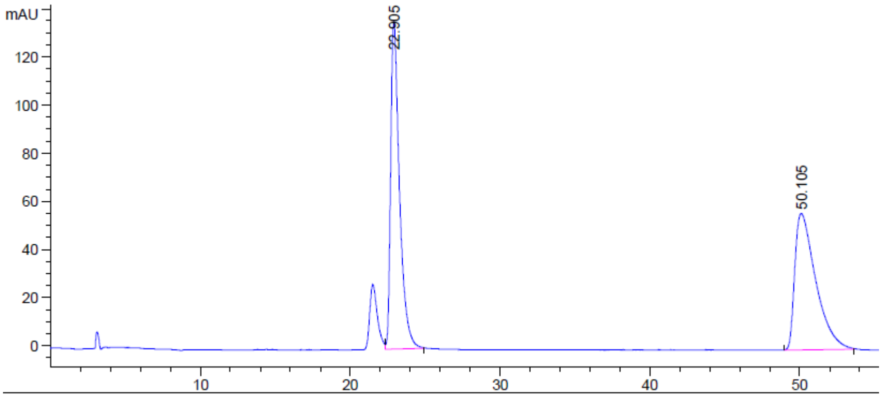

#	RetTime	Width	Area	Height	Area%
1	22.905	0.6156	5596.94531	136.13889	50.3783
2	50.105	1.4117	5512.88574	56.73282	49.6217

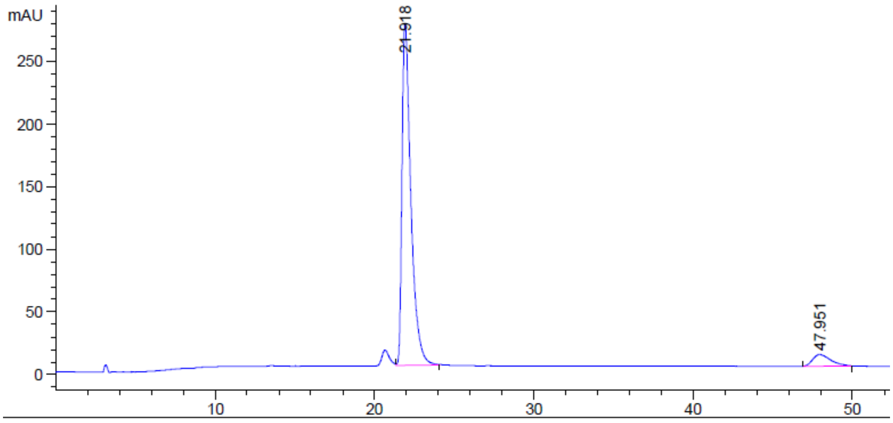

#	RetTime	Width	Area	Height	Area%
1	21.918	0.5813	1.06209e4	273.33844	93.5182
2	47.951	1.1178	736.14185	9.37748	6.4818

Data for **2f**

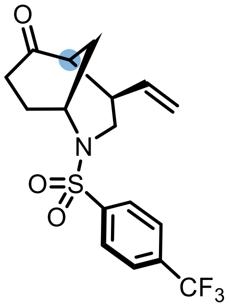

Yellow oil, 95:5 er; **[α]$_D^{25}$** = -37.7 (c = 1.0, $CHCl_3$); **^{1}H-NMR** (400 MHz, $CDCl_3$) δ 7.96 （d, *J* = 8.4 Hz, 2H）, 7.82 (d, *J* = 8.4 Hz, 2H), 5.75-5.83 (m, 1H), 5.25 (dt, *J* = 1.2 Hz, 17.2 Hz, 1H), 5.19 (dt, *J* = 1.2 Hz, 10.4 Hz, 1H), 4.30-4.36 (m, 1H), 3.52 (dd, *J* = 4.4 Hz, 12.8 Hz, 1H), 3.23 (dd, *J* = 4.4 Hz, 12.8 Hz, 1H), 2.63-2.70 (m, 1H), 2.39-2.55 (m, 3H), 2.16-2.22 (m, 1H), 1.99-2.08 (m, 1H), 1.72-1.87 (m, 2H); **^{13}C-NMR** (100 MHz, $CDCl_3$) δ 212.4, 142.8, 136.7, 134.7, 127.7, 126.4 (q, *J* = 3.0 Hz), 124.5 (q, *J* = 274.6 Hz), 117.2, 47.2, 46.3, 42.9, 40.0, 36.2, 26.9, 26.0; **HRMS (ESI+)** m/z calculated for $C_{17}H_{18}F_3NO_3S$ $[M+Na]^+$: 396.0852, found 396.0851; **HPLC** (Chiralpak IA, hexane/isopropanol 90:10, 1.0 mL/min, λ=220 nm) tR = 16.2 min (major), 33.3 min (minor).

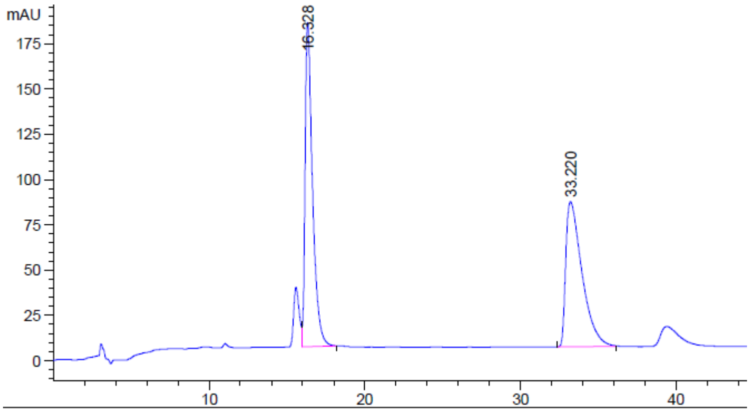

#	RetTime	Width	Area	Height	Area%
1	16.328	0.4916	5951.77344	179.15990	50.5826
2	33.220	1.0831	5814.66650	80.08616	49.4174

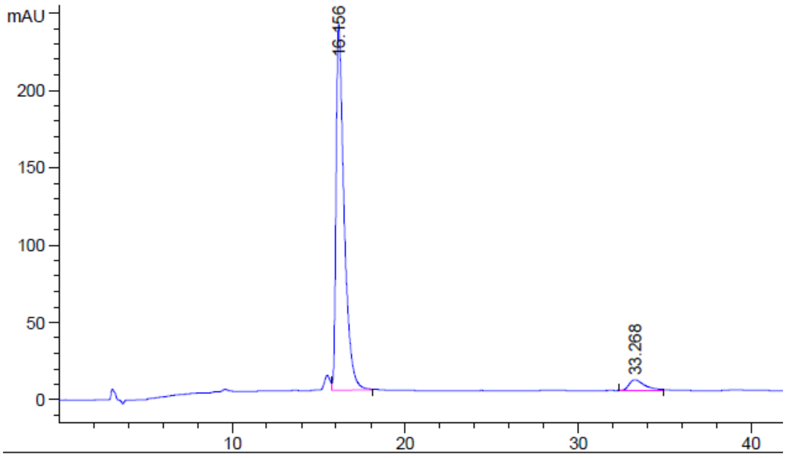

#	RetTime	Width	Area	Height	Area%
1	16.156	0.4837	7697.06201	236.51062	94.7425
2	33.268	0.8943	427.13242	6.93706	5.2575

Data for **2g**

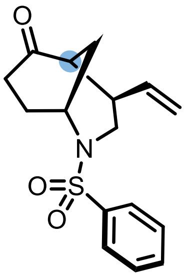

Pale yellow solid, m.p. 78-79 °C, 94:6 er; **[α]$_D$^{25}** = -55.5 (c = 1.22, $CHCl_3$); **^{1}H-NMR** (400 MHz, $CDCl_3$) δ 7.80-7.82 (m, 2H), 7.58-7.62 (m, 1H), 7.51-7.55 (m, 2H), 5.76-5.85 (m, 1H), 5.14-5.24 (m, 2H), 4.27-4.35 (m, 1H), 3.48 (dd, *J* = 4.4 Hz, 12.8 Hz, 1H), 3.20 (dd, *J* = 4.4 Hz, 12.8 Hz, 1H), 2.60-2.69 (m, 1H), 2.35-2.52 (m, 3H), 2.15-2.21 (m, 1H), 1.92-2.01 (m, 1H), 1.73-1.84 (m, 2H); **^{13}C-NMR** (100 MHz, $CDCl_3$) δ 212.9, 139.0, 136.9, 132.8, 129.2, 127.2, 117.1, 46.9, 46.5, 42.8, 40.1, 36.3, 26.8, 26.2; **HRMS (ESI+)** m/z calculated for $C_{16}H_{19}NO_3S$ [M+H]$^+$: 306.1158, found 306.1160; **HPLC** (Chiralpak IA, hexane/isopropanol 90:10, 1.0 mL/min, λ=220 nm) tR = 21.2 min (major), 33.4 min (minor).

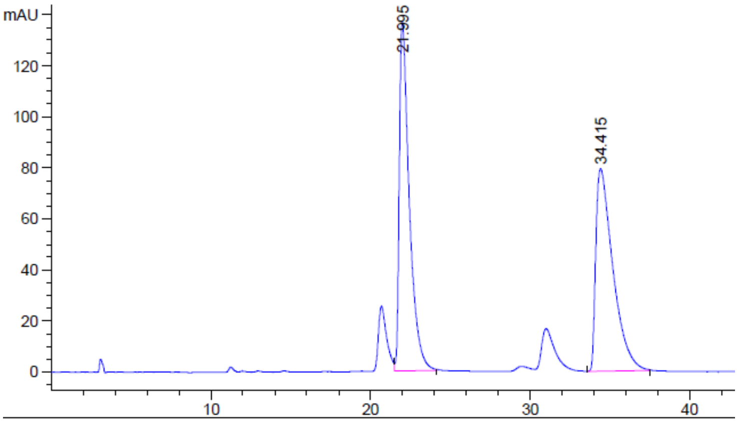

#	RetTime	Width	Area	Height	Area%
1	21.995	0.6463	5966.28809	136.41359	50.1923
2	34.415	1.1033	5920.56982	79.27924	49.8077

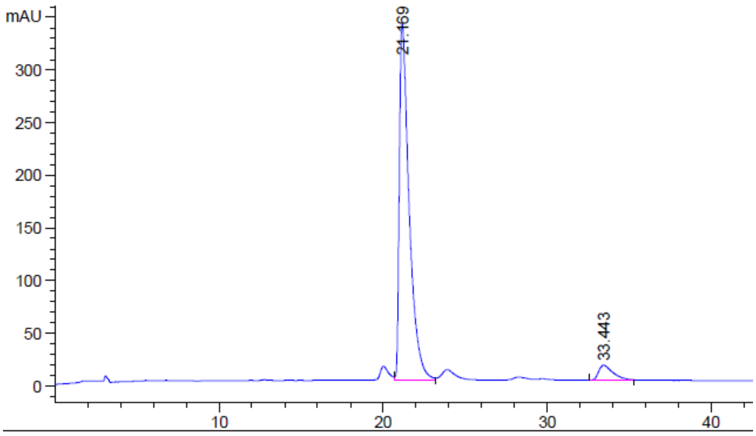

#	RetTime	Width	Area	Height	Area%
1	21.169	0.6124	1.40387e4	338.08481	94.1020
2	33.443	0.9114	879.90112	14.18967	5.8980

Data for **2h**

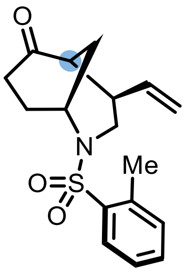

Yellow oil, 95:5 er; **[α]$_D^{25}$** = 27.8 (c = 1.0, $CHCl_3$); **^{1}H-NMR** (400 MHz, $CDCl_3$) δ 7.97-8.00 (m, 1H), 7.45-7.50 (m, 1H), 7.31-7.34 (m, 2H), 5.64-5.73 (m, 1H), 5.03 (dt, *J* = 1.2 Hz, 10.4 Hz, 1H), 4.84 (dt, *J* = 1.2 Hz, 10.4 Hz, 1H), 4.37-4.44 (m, 1H), 3.29-3.30 (m, 2H), 2.50-2.63 (m, 4H), 2.61 (s, 3H), 2.07-2.28 (m, 3H), 1.86-1.91 (m, 1H); **^{13}C-NMR** (100 MHz, $CDCl_3$) δ 213.2, 137.8, 137.1, 136.6, 133.1, 132.7, 130.5, 126.1, 116.6, 46.6, 46.0, 42.5, 39.5, 37.1, 27.3, 25.8, 20.3; **HRMS (ESI+)** m/z calculated for $C_{17}H_{21}NO_3SNa$ $[M+Na]^+$: 342.1134, found 342.1140; **HPLC** (Chiralpak IA, hexane/isopropanol 90:10, 1.0 mL/min, λ=220 nm) tR = 18.2 min (minor), 19.6 min (major).

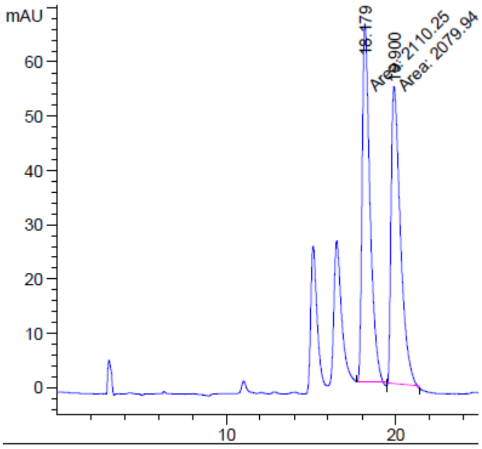

#	RetTime	Width	Area	Height	Area%
1	18.179	0.5340	2110.24683	65.86066	50.3617
2	19.900	0.6337	2079.93799	54.70107	49.6383

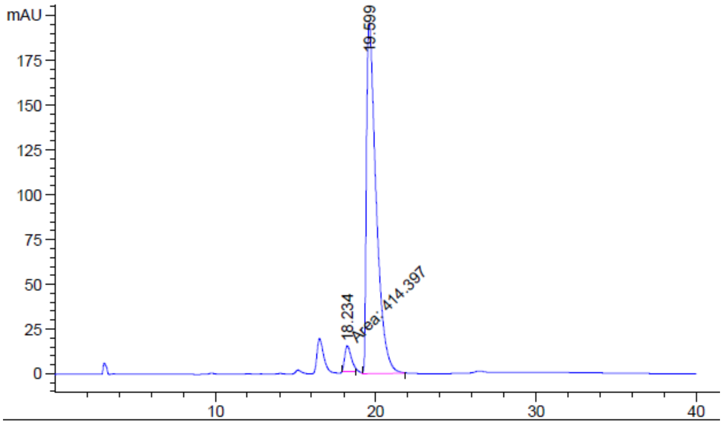

#	RetTime	Width	Area	Height	Area%
1	18.234	0.4778	414.39713	14.45441	4.7798
2	19.599	0.6278	8255.33008	195.82501	95.2202

Data for **2i**

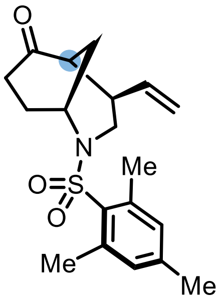

Pale yellow solid, m.p. 105-106 °C, 96:4 er; **[α]$_D^{25}$** = 31.3 (c = 0.88, $CHCl_3$); **^{1}H-NMR** (400 MHz, $CDCl_3$) δ 6.96 (s, 2H), 5.63 (m, 1H), 4.96-4.99 (m, 1H), 4.68-4.72 (m, 1H), 4.37-4.43 (m, 1H), 3.34 (dd, J = 4.4 Hz, 12.8 Hz, 1H), 3.09-3.16 (m, 1H), 2.55-2.63 (m, 3H), 2.60 (s, 6H), 2.49-2.53 (m, 1H), 2.16-2.34 (m, 3H), 2.31 (s, 3H), 1.90 (dt, J = 2.8 Hz, 13.6 Hz, 1H); **^{13}C-NMR** (100 MHz, $CDCl_3$) δ 213.3, 142.8, 140.5, 137.3, 132.0, 116.4, 100.0, 46.9, 45.6, 42.1, 39.2, 37.4, 27.4, 25.7, 22.7, 21.0; **HRMS (ESI+)** m/z calculated for $C_{19}H_{25}NO_3S$ $[M+H]^+$: 348.1628, found 348.1629; **HPLC** (Chiralpak IA, hexane/isopropanol 97:03, 1.0 mL/min, λ=220 nm) tR = 27.2 min (minor), 29.2 min (major).

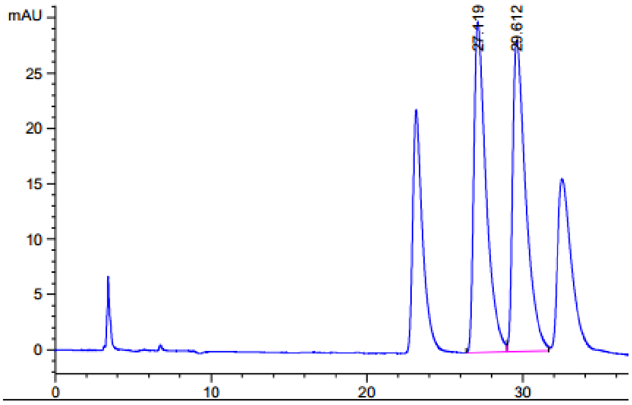

#	RetTime	Width	Area	Height	Area%
1	27.118	0.8172	4184.34033	75.90279	49.8440
2	29.611	0.8747	4210.54004	71.32658	50.1560

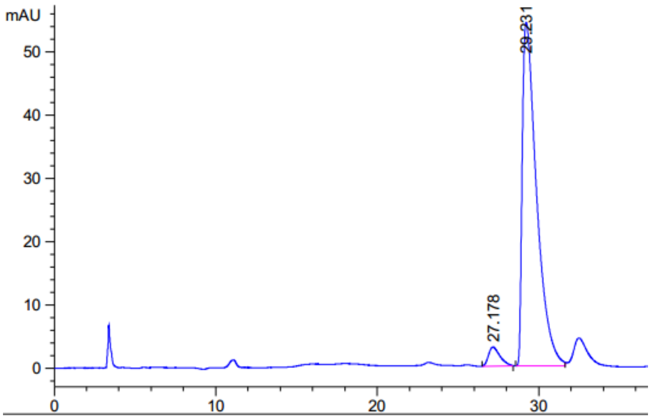

#	RetTime	Width	Area	Height	Area%
1	27.178	0.6788	150.14848	3.02637	4.3015
2	29.231	0.9134	3340.42407	54.32391	95.6985

Data for **2j**

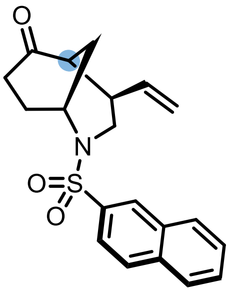

Pale yellow solid, m.p. 109-110 °C, 96:4 er; **[α]$_D^{25}$** = -52.3 (c = 1.0, $CHCl_3$); **^{1}H-NMR** (400 MHz, $CDCl_3$) δ 8.39 (m, 1H), 7.96-7.99 (m, 2H), 7.92-7.94 (d, *J* = 7.6 Hz, 1H), 7.79 (dd, *J* = 2.0 Hz, 8.4 Hz, 1H), 7.61-7.69 (m, 2H), 5.77-5.86 (m, 1H), 5.25 (dt, *J* = 1.2 Hz, 17.2 Hz, 1H), 5.25 (dt, *J* =1.2 Hz, 10.4 Hz, 1H), 4.34-4.41 (m, 1H), 3.56 (dd, *J* = 4.4 Hz, 12.8 Hz, 1H), 3.23 (dd, *J* = 4.4 Hz, 12.8 Hz, 1H), 2.61-2.70 (m, 1H), 2.34-2.51 (m, 3H), 2.16-2.22 (m, 1H), 1.93-2.03 (m, 1H), 1.79-1.85 (m, 2H); **^{13}C-NMR** (100 MHz, $CDCl_3$) δ 212.9, 137.0, 135.9, 134.8, 132.2, 129.6, 129.2, 128.9, 128.6, 127.9, 127.7, 122.4, 117.1, 47.0, 46.5, 42.9, 40.1, 36.3, 26.9, 26.2; **HRMS (ESI+)** m/z calculated for $C_{20}H_{21}NO_3S$ $[M+H]^+$: 356.1315, found 356.1316; **HPLC** (Chiralpak IA, hexane/isopropanol 90:10, 1.0 mL/min, λ=220 nm) tR = 36.8 min (major), 44.4 min (minor).

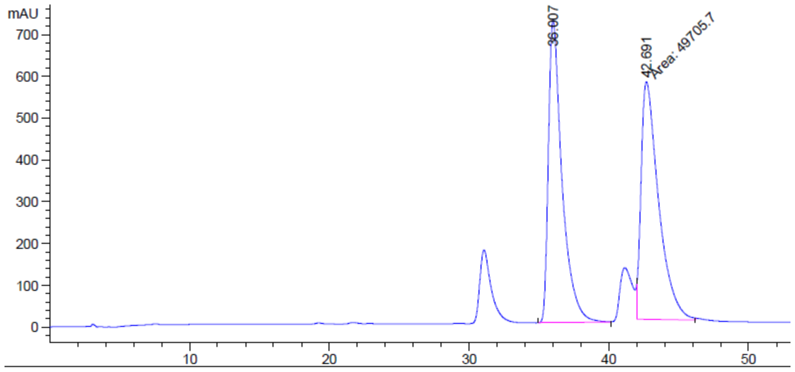

#	RetTime	Width	Area	Height	Area%
1	36.007	0.9986	4.92557e4	723.21130	49.7726
2	42.691	1.4564	4.97057e4	568.81696	50.2274

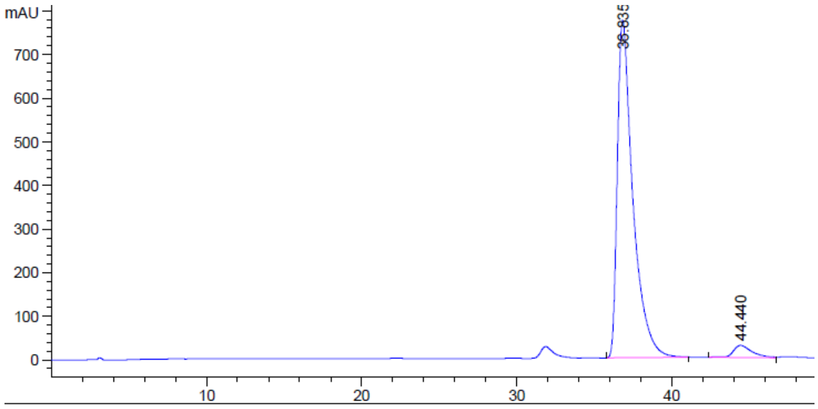

#	RetTime	Width	Area	Height	Area%
1	36.835	1.0269	5.39212e4	773.88025	95.8116
2	44.440	1.2383	2357.14429	27.93820	4.1884

Data for **2k**

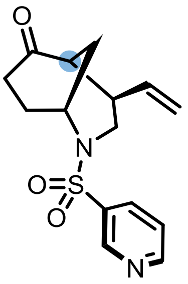

Pale yellow solid, m.p. 114-115 °C, 91:9 er; **[α]$_D^{25}$** = -48.0 (c = 1.0, $CHCl_3$); **^{1}H-NMR** (400 MHz, $CDCl_3$) δ 9.04 (d, *J* = 1.6 Hz, 1H), 8.83 (dd, *J* = 1.2 Hz, 4.8 Hz, 1H), 8.10 (dt, *J* = 2.0 Hz, 8.0 Hz, 1H), 7.48 (q, *J* = 4.8 Hz, 1H), 5.74-5.83 (m, 1H), 5.16-5.24 (m, 2H), 4.29-4.37 (m, 1H), 3.52 (dd, *J* = 4.0 Hz, 12.8 Hz, 1H), 3.23 (dd, *J* = 4.0 Hz, 12.8 Hz, 1H), 2.62-2.70 (m, 1H), 2.39-2.54 (m, 3H), 2.15-2.21 (m, 1H), 2.01-2.10 (m, 1H), 1.76-1.87 (m, 2H); **^{13}C-NMR** (100 MHz, $CDCl_3$) δ 212.3, 153.3, 148.0, 136.6, 135.8, 134.8, 123.8, 117.3, 47.1, 46.3, 42.9, 39.9, 36.2, 27.0, 26.0; **HRMS (ESI+)** m/z calculated for $C_{15}H_{18}N_2O_3S$ $[M+H]^+$: 307.1111, found 307.1110; **HPLC** (Chiralpak OD-H, hexane/isopropanol 85:15, 1.0 mL/min, λ=230 nm) tR = 41.5 min (major), 49.6 min (minor).

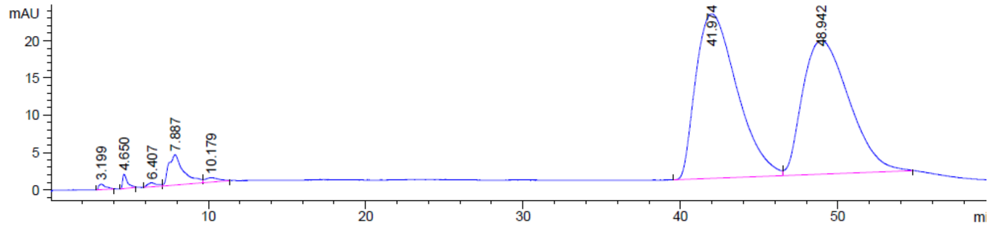

#	RetTime	Width	Area	Height	Area%
1	41.976	3.0333	4303.05029	23.64304	50.7268
2	48.952	3.5741	4179.74707	19.49092	49.2732

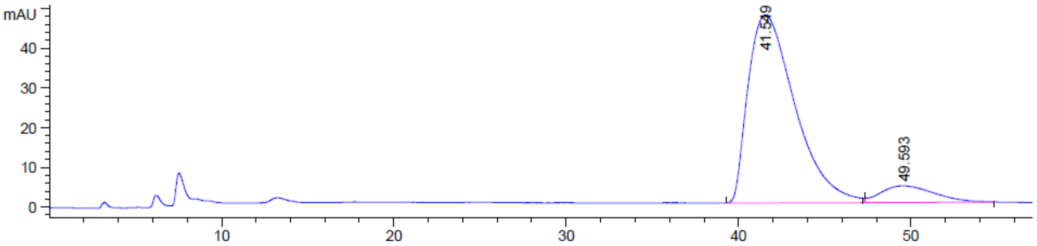

#	RetTime	Width	Area	Height	Area%
1	41.549	2.6805	8925.60742	47.32116	90.9978
2	49.593	2.4999	882.98846	4.14073	9.0022

Data for **2l**

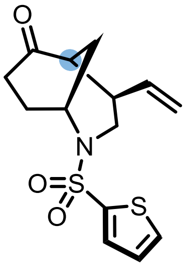

Yellow solid, m.p. 62-63 °C, 95:5 er; **[α]$_D^{25}$** = -34.4 (c = 0.7, $CHCl_3$); **^{1}H-NMR** (400 MHz, $CDCl_3$) δ 7.62 (dd, *J* = 1.2 Hz, 5.2 Hz, 1H), 7.58 (dd, *J* = 1.2 Hz, 4.0 Hz, 1H), 7.13 (dd, *J* = 4.0 Hz, 4.8 Hz, 1H), 5.77-5.86 (m, 1H), 5.27 (dt, *J* = 1.2 Hz, 17.2 Hz, 1H), 5.19 (dt, *J* = 1.2 Hz, 10.4 Hz, 1H), 4.25-4.33 (m, 1H), 3.45 (dd, *J* = 4.8 Hz, 12.8 Hz, 1H), 3.27 (dd, *J* = 4.8 Hz, 12.8 Hz, 1H), 2.63-2.73 (m, 1H), 2.37-2.60 (m, 3H), 2.17-2.23 (m, 1H), 1.94-2.03 (m, 1H), 1.81-1.92 (m, 2H); **^{13}C-NMR** (100 MHz, $CDCl_3$) δ 212.6, 139.4, 136.9, 132.1, 132.0, 127.6, 117.1, 47.5, 46.5, 42.9, 40.1, 36.0, 27.1, 26.3; **HRMS (ESI+)** m/z calculated for $C_{14}H_{17}NO_3S_2$ $[M+H]^+$: 312.0723, found 312.0723; **HPLC** (Chiralpak IA, hexane/isopropanol 90:10, 1.0 mL/min, λ=230 nm) tR = 22.3 min (major), 38.6 min (minor).

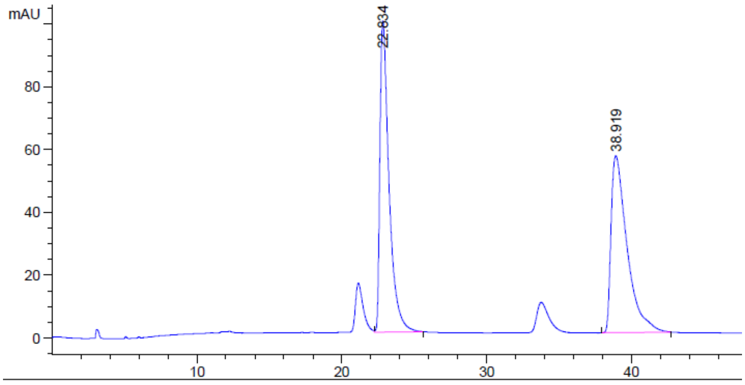

#	RetTime	Width	Area	Height	Area%
1	22.834	0.6496	4343.84033	99.04998	49.9772
2	38.919	1.1424	4347.80859	56.33756	50.0228

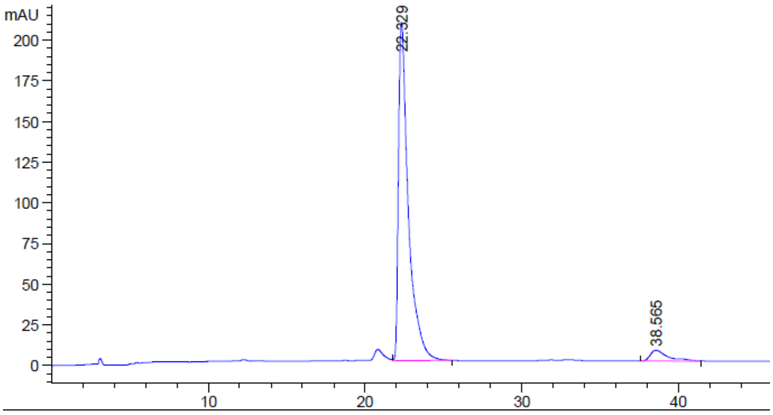

#	RetTime	Width	Area	Height	Area%
1	22.329	0.6416	9120.92383	207.98366	94.7481
2	38.565	1.0795	505.57678	6.60164	5.2519

Data for **2m**

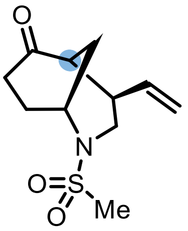

Pale yellow oil, 94:6 er; **[α]$_D^{25}$** = -30.9 (c = 1.0, $CHCl_3$); **^{1}H-NMR** (400 MHz, $CDCl_3$) δ 5.84-5.93 (m, 1H), 5.20-5.29 (m, 2H), 4.20-4.28 (m, 1H), 3.52 (dd, *J* = 4.0 Hz, 12.8 Hz, 1H), 3.33 (dd, *J* = 4.8 Hz, 12.8 Hz, 1H), 2.87 (s, 3H), 2.61-2.69 (m, 2H), 2.47-2.55 (m, 2H), 2.11-2.31 (m, 3H), 1.91 (dt, *J* = 3.2 Hz, 14.0 Hz, 1H); **^{13}C-NMR** (100 MHz, $CDCl_3$) δ 212.5, 136.9, 117.2, 46.8, 46.5, 42.9, 40.0, 38.2, 36.3, 28.0, 26.1; **HRMS (ESI+)** m/z calculated for $C_{11}H_{17}NO_3S$ $[M+Na]^+$: 266.0821, found 266.0823; **HPLC** (Chiralpak IA, hexane/isopropanol 90:10, 1.0 mL/min, λ=220 nm) tR = 21.6 min (major), 22.3 min (minor).

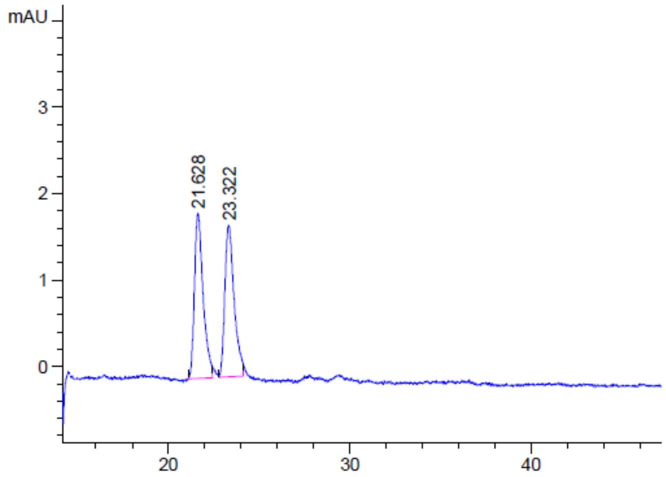

#	RetTime	Width	Area	Height	Area%
1	21.625	0.5066	184.34036	5.39600	49.9719
2	23.323	0.5209	184.54735	4.99640	50.0281

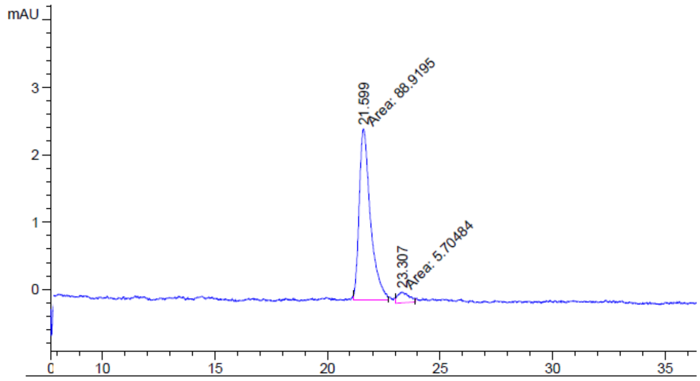

#	RetTime	Width	Area	Height	Area%
1	21.599	0.5824	88.91953	2.54453	93.9711
2	23.307	0.5969	5.70484	1.59298e-1	6.0289

Data for **2p**

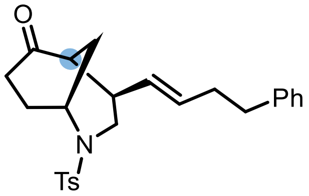

(**2p**) Yellow oil, m.p., 91:9 er; **[α]$_D^{25}$** = -19.2 (c = 0.86, $CHCl_3$). δ **^{1}H NMR** (400 MHz, $CDCl_3$) δ 7.70 (d, *J* = 8.3 Hz, 2H), 7.39 – 7.26 (m, 4H), 7.25 – 7.12 (m, 2H), 5.64 (dtd, *J* = 15.0, 6.8, 1.2 Hz, 1H), 5.37 (ddt, *J* = 15.5, 7.1, 1.4 Hz, 1H), 4.27 (dd, *J* = 6.1, 3.0 Hz, 1H), 3.34 (dd, *J* = 12.8, 4.5 Hz, 1H), 3.17 (dd, *J* = 12.7, 4.8 Hz, 1H), 2.72 – 2.63 (m, 2H), 2.59 (q, *J* = 3.2 Hz, 1H), 2.52 – 2.46 (m, 1H), 2.45 (s, 3H), 2.43 – 2.37 (m, 1H), 2.37 – 2.26 (m, 3H), 2.11 – 2.00 (m, 1H), 1.99 – 1.91 (m, 1H), 1.91 – 1.81 (m, 1H), 1.74 (dt, *J* = 13.9, 3.1 Hz, 1H). **^{13}C NMR** (101 MHz, $CDCl_3$) δ 213.2, 143.7, 141.7, 136.3, 132.1, 129.9, 129.6, 128.6, 128.4, 127.4, 125.9, 47.4, 47.1, 43.5, 39.6, 36.3, 35.7, 34.5, 27.4, 26.3, 21.7. **HRMS (ESI+) m/z** calculated for $C_{25}H_{29}NO_3S$ [M+H]$^+$: 424.1941, found 424.1937; **HPLC** (Chiralpak AD-H, hexane/isopropanol 90:10, 1.0 mL/min, λ=220 nm) tR = 35.9 min (minor), 38.1 min (major).

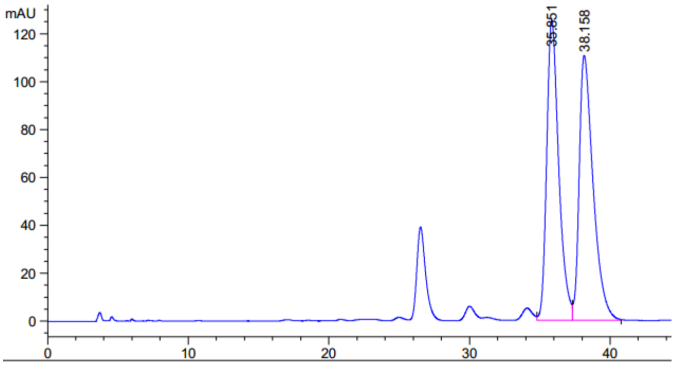

#	RetTime	Width	Area	Height	Area%
1	35.851	0.9021	7470.02490	125.55680	49.5324
2	38.158	1.0329	7611.06104	110.59578	50.4676

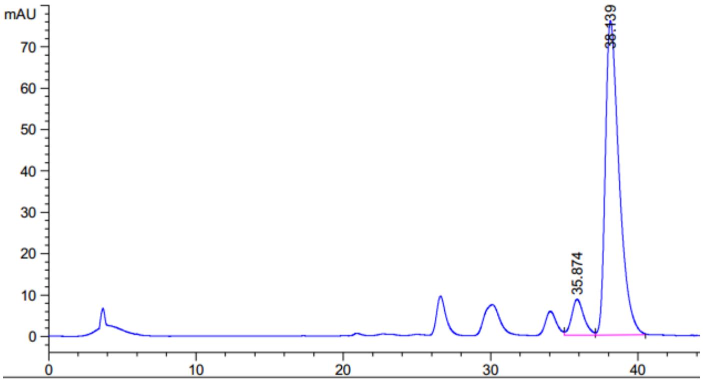

#	RetTime	Width	Area	Height	Area%
1	35.874	0.8541	497.61234	8.63753	9.1265
2	38.139	0.9789	4954.80322	75.96892	90.8735

6: Synthesis of Bicyclic Oxygen Variants

Synthesis of **4b** is representative

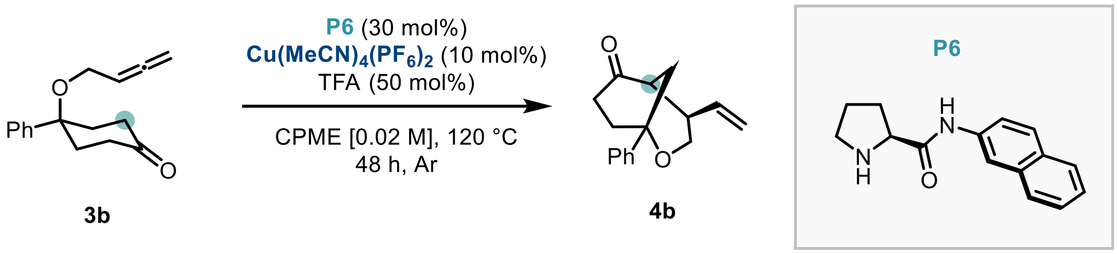

Allene **3b** (73 mg, 0.3 mmol), **P6** (21.6 mg, 0.09 mmol), $Cu(CH_3CN)_4PF_6$ (11 mg, 0.03 mmol) were added into a sealed tube, the tube was evacuated and filled with argon (3 times), anhydrous CPME (15 mL) and TFA (11 μL, 0.15 mmol) were then injected into the tube. The reaction mixture was stirred at 120 °C for 24 h. The mixture was then allowed to cool to room temperature, diluted with EtOAc and filtered through celite (eluting with additional 5 mL EtOAc). The filtrate was evaporated in vacuum and purified by column chromatography to afford the desired product **4b** (70.5 mg, 97% yield, 84% ee).

Data for **4b**

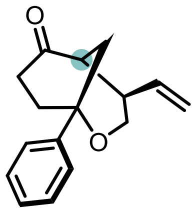

Pale yellow solid, 77-78 °C, 84% ee; **[α]$_D^{25}$** = 24.2 (c = 1.0, $CHCl_3$); **^{1}H-NMR** (400 MHz, $CDCl_3$) δ 7.43-7.46 (m, 2H), 7.36-7.40 (m, 2H), 7.27-7.31 (m, 1H), 5.71-5.80 (m, 1H), 5.16 (dt, *J* = 1.2 Hz, 17.2 Hz, 1H), 5.09 (dt, *J* = 1.2 Hz, 10.4 Hz, 1H), 3.92 (dd, *J* = 3.2 Hz, 12.0 Hz, 1H), 3.52 (dd, *J* = 4.8 Hz, 12.0 Hz, 1H), 3.00 (q, *J* = 8.0 Hz, 1H), 2.77-2.85 (m, 1H), 2.52-2.59 (m, 2H), 2.40 (dd, *J* = 4.0 Hz, 14.0 Hz, 1H), 2.30 (dt, *J* = 2.0 Hz, 14.0 Hz, 1H), 2.13-2.17 (m, 2H); **^{13}C-NMR** (100 MHz, $CDCl_3$) δ 213.5, 146.0, 136.6, 128.4, 127.2, 124.4, 116.3, 75.0, 63.1, 46.9, 43.3, 37.9, 36.1, 30.4; **HRMS (ESI+)** m/z calculated for $C_{16}H_{18}O_2$ [M+H]$^+$: 243.1380, found 243.1382; **HPLC** (Chiralpak IA, hexane/isopropanol 98:02, 1.0 mL/min, λ=220 nm) tR = 14.1 min (major), 19.3 min (minor).

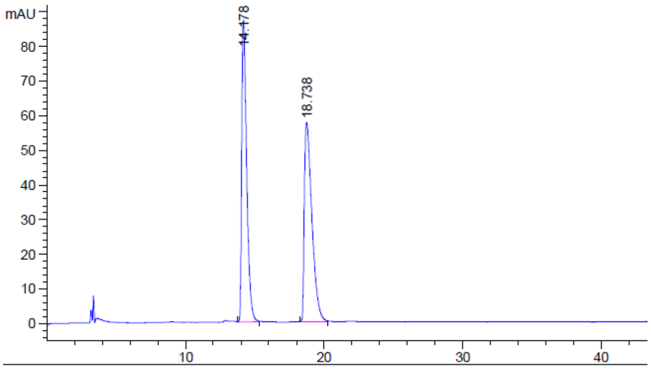

#	RetTime	Width	Area	Height	Area%
1	14.178	0.3801	2162.87109	86.98000	49.7824
2	18.738	0.5862	2181.77832	57.56450	50.2176

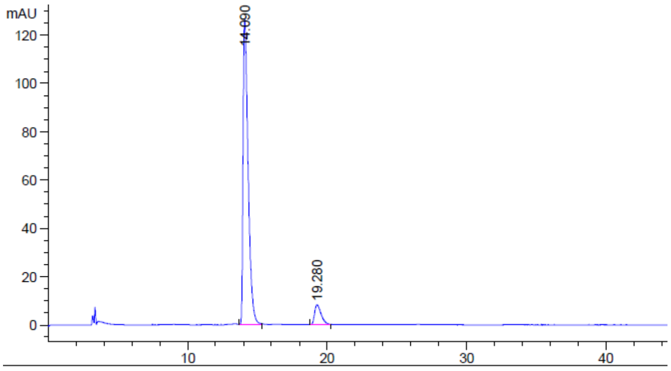

#	RetTime	Width	Area	Height	Area%
1	14.090	0.3883	3198.26855	125.91132	92.2327
2	19.280	0.5042	269.33801	8.22156	7.7673

Data for **4c**

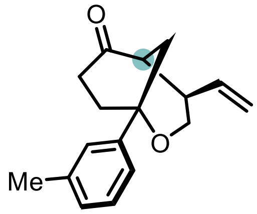

Pale yellow oil, 80% ee; **[α]$_D$^{25}** = 17.9 (c = 1.0, $CHCl_3$); **^{1}H-NMR** (400 MHz, $CDCl_3$) δ 7.20-7.24 (m, 3H), 7.07-7.09 (m, 1H), 5.68-5.77 (m, 1H), 5.13 (dt, J = 1.2 Hz, 17.2 Hz, 1 H), 5.06 (dt, J = 1.2 Hz, 10.4 Hz, 1H), 3.89 (dd, J = 3.2 Hz, 12.0 Hz, 1H), 3.49 (dd, J = 8.8 Hz, 12.0 Hz, 1H), 2.97 (q, J = 7.6 Hz, 1H), 2.74-2.82 (m, 1H), 2.49-2.56 (m, 2H), 2.33-2.38 (m, 1H), 2.35 (s, 3H), 2.27 (dt, J = 2.0 Hz, 14.0 Hz, 1H), 2.09-2.14 (m, 2H); **^{13}C-NMR** (100 MHz, $CDCl_3$) δ 213.6, 145.9, 138.0, 136.6, 128.3, 127.9, 125.1, 121.4, 116.3, 75.0, 63.1, 46.9, 43.3, 37.9, 36.1, 30.3, 21.6; **HRMS (ESI+)** m/z calculated for $C_{17}H_{20}O_2$ $[M+H]^+$: 257.1536, found 257.1537; **HPLC** (Chiralpak IA, hexane/isopropanol 98:02, 1.0 mL/min, λ=220 nm) tR = 12.3 min (major), 19.1 min (minor).

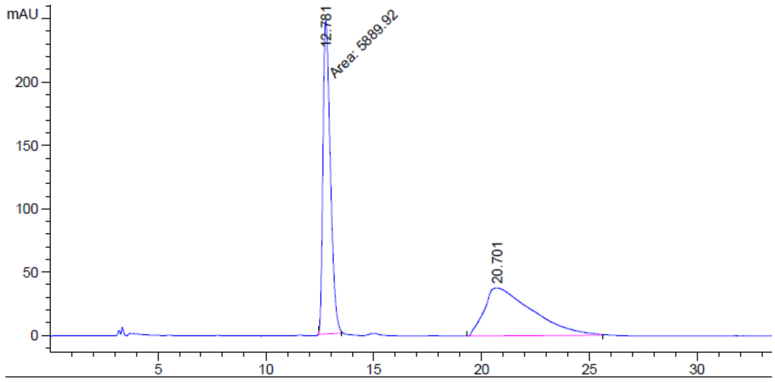

#	RetTime	Width	Area	Height	Area%
1	12.781	0.3970	5889.92188	247.26558	50.2091
2	20.701	1.9302	5840.86328	37.85310	49.7909

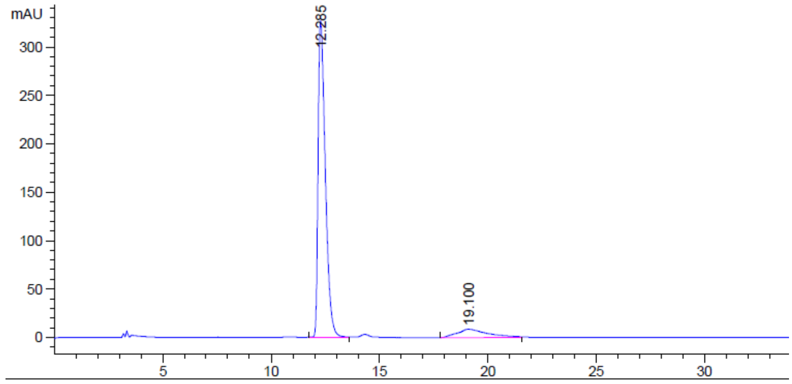

#	RetTime	Width	Area	Height	Area%
1	12.285	0.3540	7562.53076	326.72842	90.2035
2	19.100	1.2329	821.32123	8.45821	9.7965

Data for **4d**

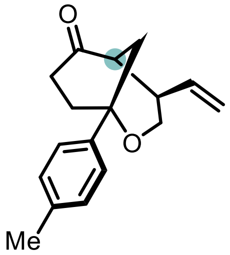

Pale yellow solid, 64-65 °C, 93:7 er; **[α]$_D^{25}$** = 19.5 (c = 0.8, $CHCl_3$); **^{1}H-NMR** (400 MHz, $CDCl_3$) δ 7.35 (d, *J* = 8.0 Hz, 2H), 7.20 (d, *J* = 8.0 Hz, 2H), 5.71-5.80 (m, 1H), 5.15 (dt, *J* = 1.2 Hz, 17.2 Hz, 1H), 5.09 (dt, *J* = 1.2 Hz, 10.4 Hz, 1H), 3.91 (dd, *J* = 7.2 Hz, 12.0 Hz, 1H), 3.51 (dd, *J* = 9.2 Hz, 12.0 Hz, 1H), 2.95-3.01 (m, 1H), 2.77-2.85 (m, 1H), 2.51-2.58 (m, 2H), 2.34-2.39 (m, 1H), 2.36 (s, 3H), 2.25-2.32 (m, 1H), 2.12-2.16 (m, 2H); **^{13}C-NMR** (100 MHz, $CDCl_3$) δ 213.6, 143.0, 136.8, 136.7, 129.1, 124.4, 116.3, 74.9, 63.1, 46.9, 43.3, 37.9, 36.1, 30.4, 21.0; **HRMS (ESI+)** m/z calculated for $C_{17}H_{20}O_2$ $[M+H]^+$: 257.1536, found 257.1538; **HPLC** (Chiralpak IA, hexane/isopropanol 99:01, 1.0 mL/min, λ=220 nm) tR = 13.1 min (major), 23.2 min (minor).

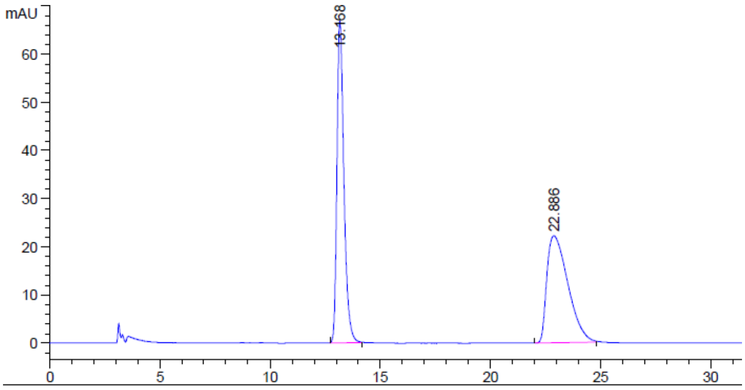

#	RetTime	Width	Area	Height	Area%
1	13.168	0.3403	1493.30762	66.93452	50.2884
2	22.886	1.0227	1476.17957	22.23059	49.7116

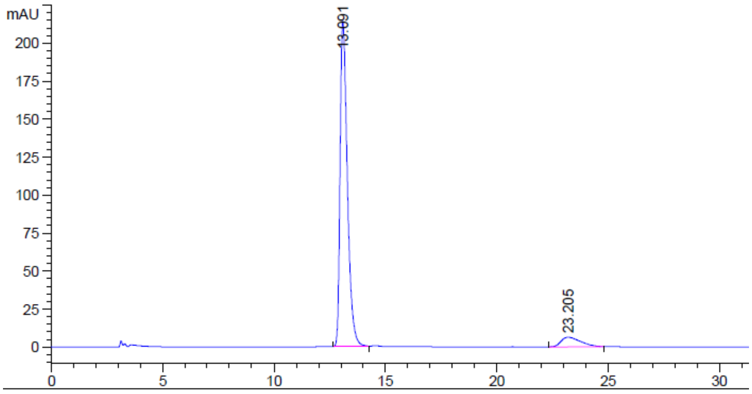

#	RetTime	Width	Area	Height	Area%
1	13.091	0.3490	4901.00391	214.16803	92.6577
2	23.205	0.8986	388.36334	6.34051	7.3423

Data for **4e**

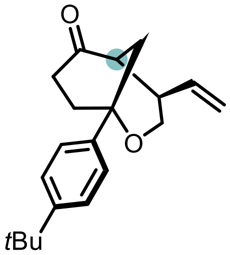

White solid, 83-84 °C, 94:6 er; **[α]$_D^{25}$** = 16.5 (c = 1.0, $CHCl_3$); **^{1}H-NMR** (400 MHz, $CDCl_3$) δ 7.36-7.41 (m, 4H), 5.71-5.80 (m, 1H), 5.25 (dt, *J* = 1.2 Hz, 17.2 Hz, 1H), 5.08 (dt, *J* = 1.2 Hz, 10.4 Hz, 1H), 3.91 (dd, *J* = 3.2 Hz, 12.0 Hz, 1H), 3.53 (dd, *J* = 8.8 Hz, 12.0 Hz, 1H), 2.98 (q, *J* = 8.0 Hz, 1H), 2.77-2.85 (m, 1H), 2.51-2.58 (m, 2H), 2.28-2.40 (m, 2H), 2.13-2.17 (m, 2H), 1.33 (s, 9H); **^{13}C-NMR** (100 MHz, $CDCl_3$) δ 213.7, 150.0, 142.9, 136.7, 125.3, 124.1, 116.3, 74.8, 63.1, 46.9, 43.3, 37.8, 36.1, 34.4, 31.3, 30.4, 22.3, 14.0; **HRMS (ESI+)** m/z calculated for $C_{20}H_{26}O_2$ $[M+H]^+$: 299.2006, found 299.2006; **HPLC** (Chiralpak IA, hexane/isopropanol 98:02, 1.0 mL/min, λ=220 nm) tR = 11.6 min (major), 23.6 min (minor).

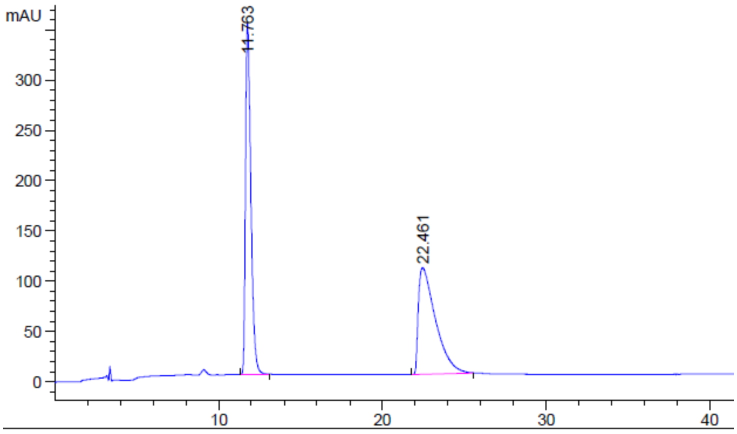

#	RetTime	Width	Area	Height	Area%
1	11.763	0.3499	7960.87891	349.34369	50.6829
2	22.461	1.0762	7746.34180	106.04739	49.3171

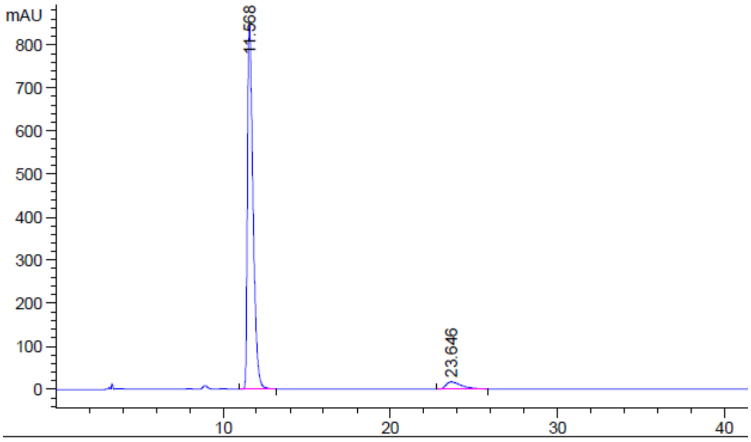

#	RetTime	Width	Area	Height	Area%
1	11.568	0.3608	2.30080e4	983.99896	94.3365
2	23.645	0.9907	1381.28088	19.97748	5.6635

Data for **4f**

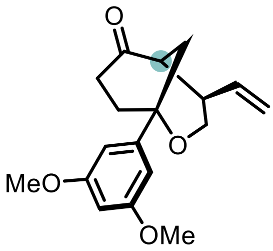

Pale yellow oil, 94:6 er; **[α]$_D^{25}$** = 11.1 (c = 0.65, $CHCl_3$); **^{1}H-NMR** (400 MHz, $CDCl_3$) δ 6.60 (d, *J* = 2.4 Hz, 2H), 6.38 (t, *J* = 2.4 Hz, 1H), 5.69-5.78 (m, 1H), 5.15 (dt, *J* = 1.2 Hz, 17.2 Hz, 1H), 5.08 (dt, *J* = 1.2 Hz, 10.4 Hz, 1H), 3.89 (dd, *J* = 7.2 Hz, 12.0 Hz, 1H), 3.81 (s, 6H), 3.50 (dd, *J* = 8.8 Hz, 12.0 Hz, 1H), 3.00 (q, *J* = 8.0 Hz, 1H), 2.74-2.82 (m, 1H), 2.50-2.57 (m, 2H), 2.25-2.37 (m, 2H), 2.10-2.15 (m, 2H); **^{13}C-NMR** (100 MHz, $CDCl_3$) δ 213.5, 160.9, 148.6, 136.5, 116.4, 102.8, 98.6, 75.2, 63.1, 55.3, 46.8, 43.3, 37.8, 36.0, 30.2; **HRMS (ESI+)** m/z calculated for $C_{18}H_{22}O_4$ $[M+H]^+$: 303.1591, found 303.1592; **HPLC** (Chiralpak AD-H, hexane/isopropanol 97:03, 1.0 mL/min, λ=220 nm) tR = 24.8 min (minor), 28.7 min (major).

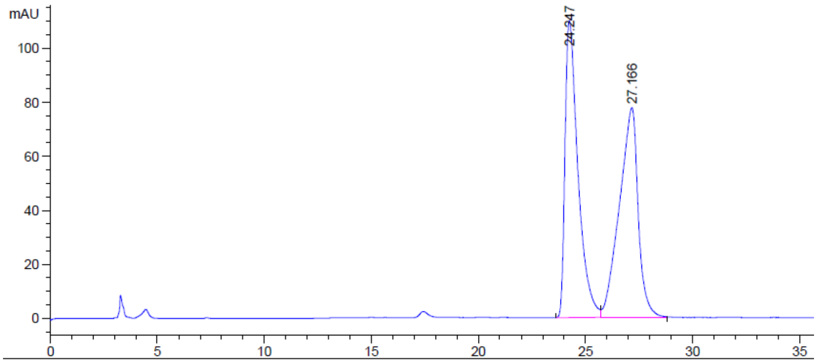

#	RetTime	Width	Area	Height	Area%
1	24.247	0.6300	4642.88330	110.09670	49.5100
2	27.166	0.8605	4734.79102	77.74995	50.4900

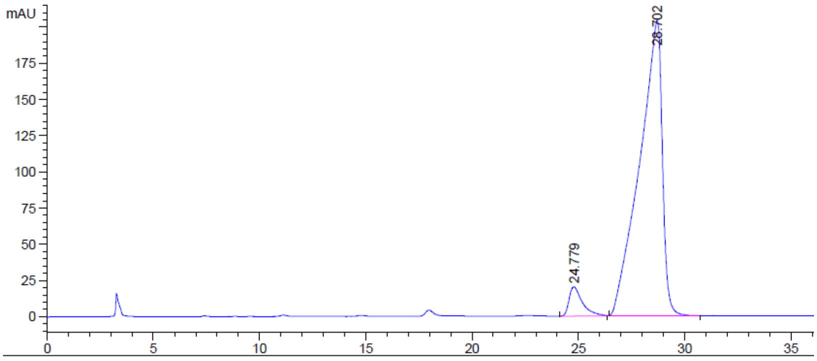

#	RetTime	Width	Area	Height	Area%
1	24.779	0.6462	887.36359	20.29042	5.5323
2	28.702	1.0094	1.51523e4	204.26244	94.4677

Data for **4g**

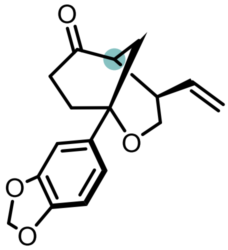

Pale yellow solid, 61-62 ^{o}C, 81:19 er; **[α]$_D^{25}$** = 9.2 (c = 0.9, $CHCl_3$); **^{1}H-NMR** (400 MHz, $CDCl_3$) δ 6.96 (d, *J* = 1.6 Hz, 1H), 6.89 (dd, *J* = 1.6 Hz, 8.0 Hz, 1H), 6.80 (d, *J* = 8.0 Hz, 1H), 5.95 (s, 2H), 5.70-5.78 (m, 1H), 5.24 (dt, *J* = 1.2 Hz, 17.2 Hz, 1H), 5.08 (dt, *J* = 1.2 Hz, 10.4 Hz, 1H), 3.87 (dd, *J* = 3.2 Hz, 12.0 Hz, 1H), 3.47 (dd, *J* = 8.8 Hz, 12.0 Hz, 1H), 2.98 (q, *J* = 7.6 Hz, 1H), 2.73-2.81 (m, 1H), 2.48-2.56 (m, 2H), 2.34 (dd, *J* = 3.6 Hz, 14.0 Hz, 1H), 2.26 (dt, *J* = 2.0 Hz, 14.0 Hz, 1H), 2.07-2.13 (m, 2H); **^{13}C-NMR** (100 MHz, $CDCl_3$) δ 213.5, 147.7, 146.5, 140.1, 136.6, 117.5, 116.3, 108.0, 105.5, 101.0, 75.0, 63.0, 46.8, 43.3, 38.1, 36.1, 30.4; **HRMS (ESI+)** m/z calculated for $C_{17}H_{18}O_4$ [M+H]$^+$: 287.1278, found 287.1279; **HPLC** (Chiralpak IB, hexane/isopropanol 98:02, 1.0 mL/min, λ=210 nm) tR = 21.3 min (major), 24.3 min (minor).

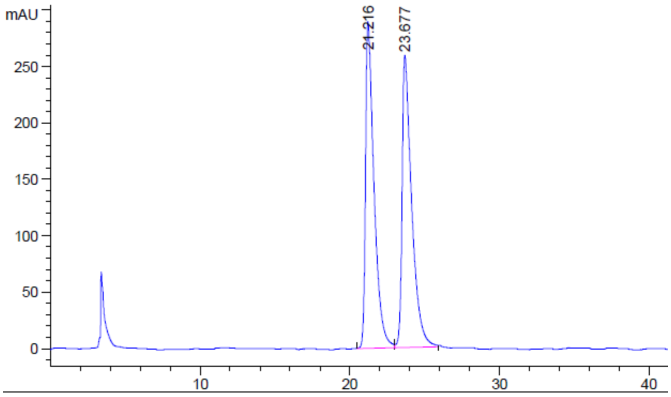

#	RetTime	Width	Area	Height	Area%
1	21.216	0.5697	3813.48267	96.35866	50.0818
2	23.677	0.6292	3801.01978	85.73341	49.9182

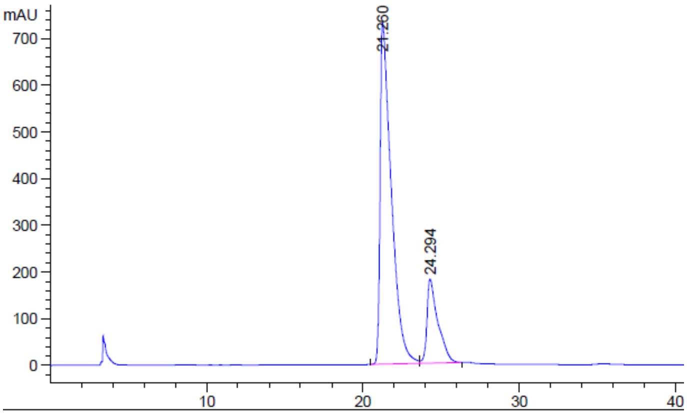

#	RetTime	Width	Area	Height	Area%
1	21.260	0.6974	3.62403e4	732.22626	80.5008
2	24.294	0.6904	8778.23730	180.18222	19.4992

Data for **4h**

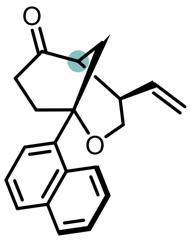

Pale yellow solid, 66-67 °C, 93:7 er; **[α]$_D^{25}$** = 72.3 (c = 1.0, $CHCl_3$); **^{1}H-NMR** (400 MHz, $CDCl_3$) δ 8.59-8.62 (m, 1H), 7.87-7.89 (m, 1H), 7.82 (d, *J* = 8.0 Hz, 1H), 7.59-7.60 (m, 1H), 7.42-7.52 (m, 3H), 5.80-5.89 (m, 1H), 5.19 (dt, *J* = 1.2 Hz, 17.2 Hz, 1H), 5.11 (dt, *J* = 1.2 Hz, 10.4 Hz, 1H), 3.93 (dd, *J* = 6.8 Hz, 12.0 Hz, 1H), 3.51 (dd, *J* = 7.6 Hz, 12.0 Hz, 1H), 3.14 (q, *J* = 7.2 Hz, 1H), 2.57-2.84 (m, 6H), 2.31-2.39 (m, 2H); **^{13}C-NMR** (100 MHz, $CDCl_3$) δ 214.2, 140.7, 136.8, 134.9, 131.0, 129.2, 128.9, 126.6, 125.5, 125.3, 124.8, 123.0, 116.3, 77.0, 62.7, 46.4, 43.9, 36.4, 35.9, 30.2; **HRMS (ESI+)** m/z calculated for $C_{20}H_{20}O_2$ $[M+H]^+$: 293.1536, found 293.1537; **HPLC** (Chiralpak IB, hexane/isopropanol 98:02, 1.0 mL/min, λ=220 nm) tR = 25.4 min (major), 29.7 min (minor).

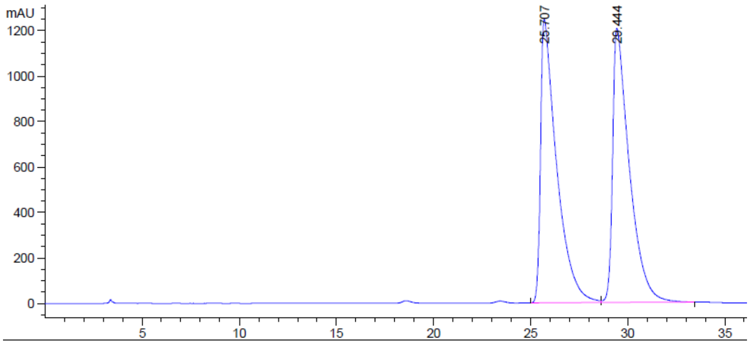

#	RetTime	Width	Area	Height	Area%
1	25.707	0.8183	7.16857e4	1247.62256	49.8024
2	29.444	0.8506	7.22545e4	1206.58789	50.1976

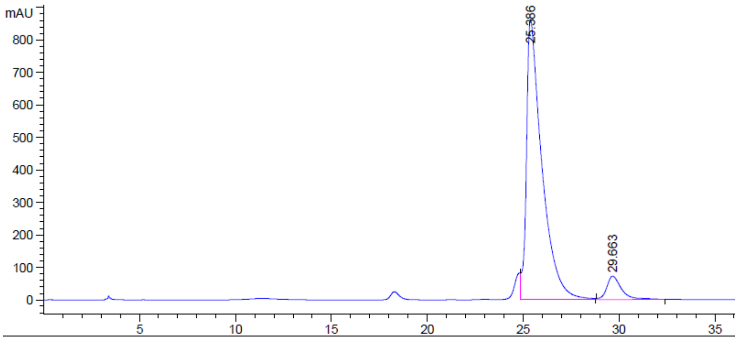

#	RetTime	Width	Area	Height	Area%
1	25.386	0.7926	4.82657e4	862.83795	92.5995
2	29.663	0.7853	3857.35107	72.19466	7.4005

Data for **4i**

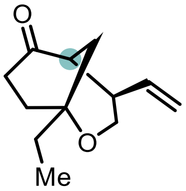

Pale yellow oil, 94:6 er; **[α]$_D$^{25}** = 55.7 (c = 0.58, $CHCl_3$); **^{1}H-NMR** (400 MHz, $CDCl_3$) δ 5.83-5.92 (m, 1H), 5.19 (dt, *J* = 1.2 Hz, 17.2 Hz, 1H), 5.13 (dt, *J* = 1.2 Hz, 10.4 Hz, 1H), 3.76 (dd, *J* = 5.6 Hz, 12.0 Hz, 1H), 3.61 (dd, *J* = 5.6 Hz, 12.0 Hz, 1H), 2.57-2.70 (m, 2H), 2.37-2.51 (m, 2H), 2.09 (dt, *J* = 2.4 Hz, 14.0 Hz, 1H), 1.83-1.97 (m, 2H), 1.68 (dd, *J* = 3.6 Hz, 14.0 Hz, 1H), 1.57 (q, *J* = 7.6 Hz, 2H), 0.93 (t, *J* = 7.6 Hz, 3H); **^{13}C-NMR** (100 MHz, $CDCl_3$) δ 214.8, 137.4, 116.3, 72.5, 63.0, 46.7, 42.2, 36.4, 33.9, 31.8, 28.8, 7.6; **HRMS (ESI+)** m/z calculated for $C_{12}H_{18}O_2$ $[M+H]^+$: 195.1380, found 195.1376; **HPLC** (Chiralpak AD-H, hexane/isopropanol 98:02, 1.0 mL/min, λ=210 nm) tR = 8.3 min (major), 9.6 min (minor).

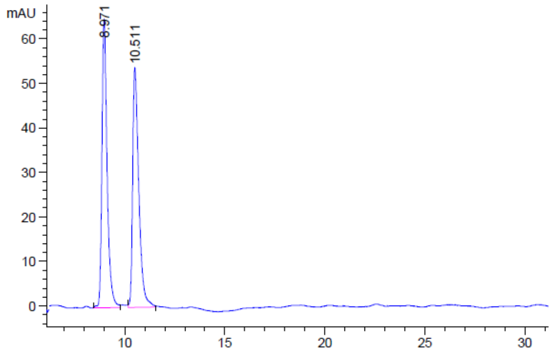

#	RetTime	Width	Area	Height	Area%
1	8.971	0.2695	1162.30017	64.72644	50.4823
2	10.511	0.3173	1140.08972	53.78853	49.5177

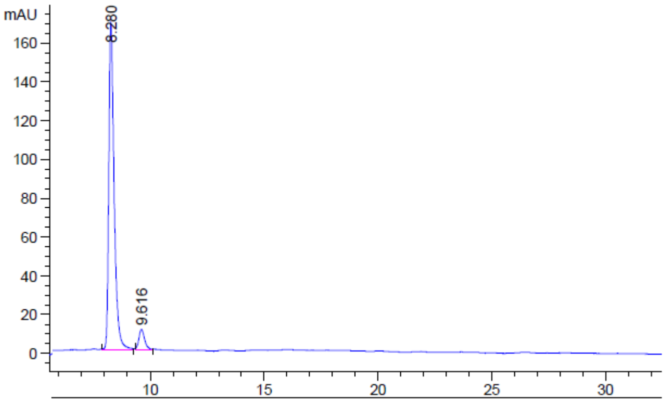

#	RetTime	Width	Area	Height	Area%
1	8.280	0.2433	2744.32861	169.13475	93.5825
2	9.616	0.2716	188.19427	10.57448	6.4175

Data for **4j**

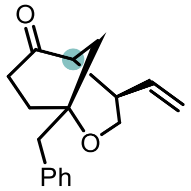

Pale yellow oil, 93:7 er; **[α]$_D^{25}$** = 18.3 (c = 0.84, $CHCl_3$); **^{1}H-NMR** (400 MHz, $CDCl_3$) δ 7.22-7.31 (m, 5H), 5.68-5.77 (m, 1H), 5.03-5.11 (m, 2H), 3.74 (dd, *J* = 6.0 Hz, 12.0 Hz, 1H), 3.45 (dd, *J* = 7.6 Hz, 12.0 Hz, 1H), 2.78-2.86 (m, 2H), 2.66-2.74 (m, 1H), 2.62 (q, *J* = 7.2 Hz, 1H), 2.41-2.49 (m, 1H), 2.30-2.38 (m, 1H), 2.07-2.16 (m, 1H), 1.85-1.91 (m, 2H), 1.68 (dd, *J* = 3.6 Hz, 14.0 Hz, 1H); **^{13}C-NMR** (100 MHz, $CDCl_3$) δ 213.8, 137.0, 136.7, 130.5, 127.9, 126.5, 116.2, 72.7, 63.0, 46.9, 46.8, 42.1, 35.8, 33.4, 29.1; **HRMS (ESI+)** m/z calculated for $C_{17}H_{20}O_2$ $[M+H]^+$: 257.1536, found 257.1538; **HPLC** (Chiralpak IA, hexane/isopropanol 98:02, 1.0 mL/min, λ=220 nm) tR = 12.1 min (major), 14.3 min (minor).

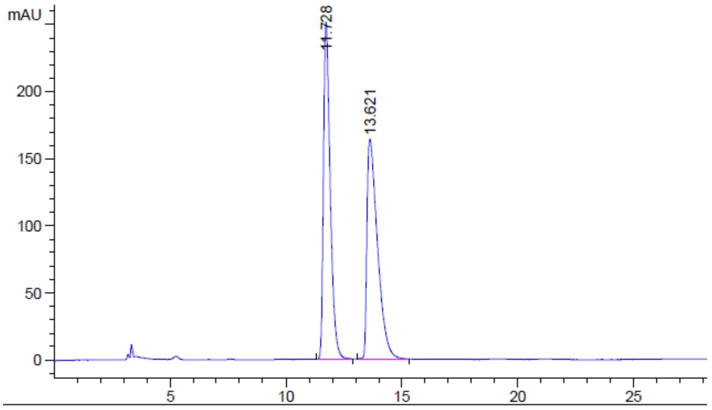

#	RetTime	Width	Area	Height	Area%
1	11.728	0.3180	5217.96777	251.55479	49.9000
2	13.621	0.4780	5238.88770	164.28943	50.1000

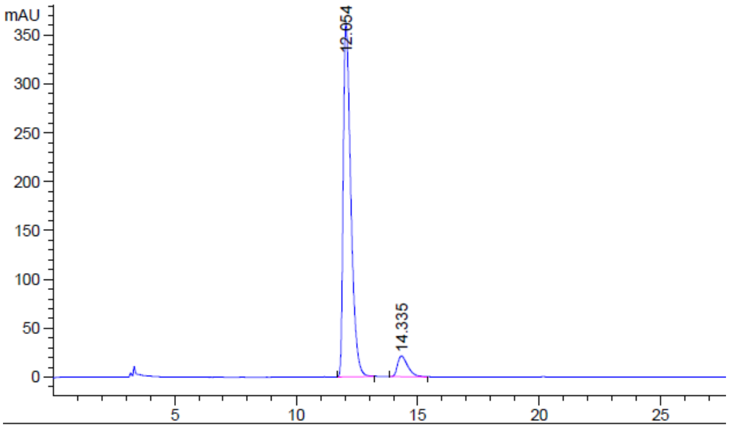

#	RetTime	Width	Area	Height	Area%
1	12.054	0.3328	7845.96631	362.15353	92.5629
2	14.335	0.4501	630.39764	21.24877	7.4371

Data for **4k**

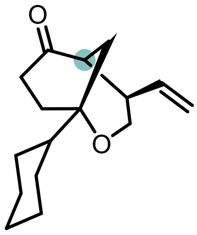

Pale yellow oil, 92:8 er; **[α]$_D^{25}$** = 42.2 (c = 0.6, $CHCl_3$); **^{1}H-NMR** (400 MHz, $CDCl_3$) δ 5.79-5.88 (m, 1H), 5.17 (dt, *J* = 1.2 Hz, 17.2 Hz, 1H), 5.11 (dt, *J* = 1.2 Hz, 10.4 Hz, 1H), 3.72 (dd, *J* = 6.0 Hz, 12.0 Hz, 1H), 3.56 (dd, *J* = 6.4 Hz, 12.0 Hz, 1H), 2.62-2.70 (m, 2H), 2.41-2.48 (m, 1H), 2.33-2.41 (m, 1H), 2.05 (dt, *J* = 2.4 Hz, 14.0 Hz, 1H), 1.90-1.97 (m, 1H), 1.66-1.86 (m, 7H), 1.46 (dt, *J* = 2.4 Hz, 12.0 Hz, 1H), 0.95-1.28 (m, 5H); **^{13}C-NMR** (100 MHz, $CDCl_3$) δ 215.0, 137.4, 116.2, 74.8, 62.9, 47.2, 46.5, 42.6, 36.4, 29.6, 27.1, 26.8, 26.7, 26.57, 26.55, 26.5; **HRMS (ESI+)** m/z calculated for $C_{16}H_{24}O_2$ $[M+H]^+$: 249.1849, found 249.1858; **HPLC** (Chiralpak IA, hexane/isopropanol 98:02, 1.0 mL/min, λ=210 nm) tR = 9.5 min (major), 10.9 min (minor).

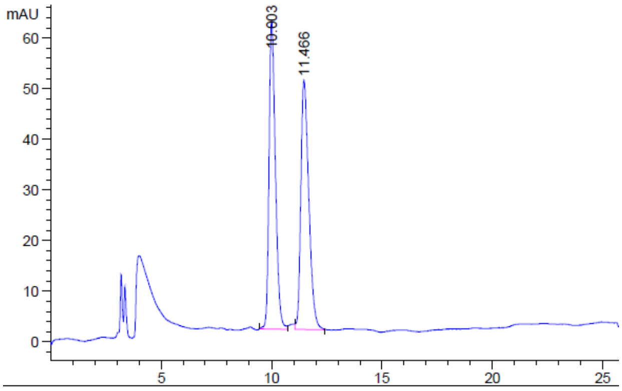

#	RetTime	Width	Area	Height	Area%
1	10.003	0.3069	1223.16943	60.75393	49.7020
2	11.466	0.3851	1237.83728	49.27198	50.2980

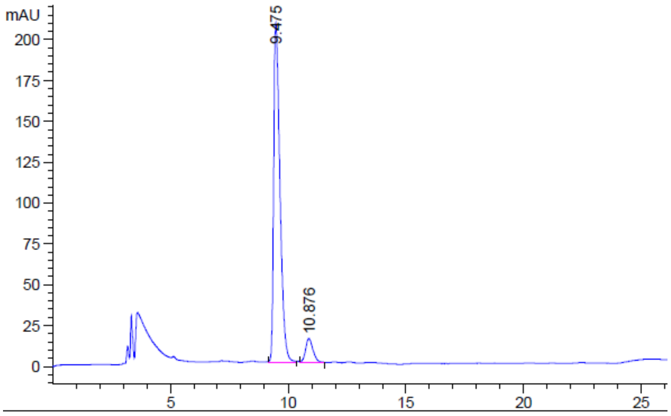

#	RetTime	Width	Area	Height	Area%
1	9.475	0.2902	3923.62671	207.91458	92.4712
2	10.876	0.3342	319.45474	14.65902	7.5288

Data for **4l**

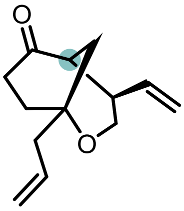

Pale yellow oil, 94:6; **[α]$_D$^{25}** = 49.4 (c = 0.85, $CHCl_3$); **^{1}H-NMR** (400 MHz, $CDCl_3$) δ 5.81-5.91 (m, 2H), 5.05-5.20 (m, 4H), 3.77 (dd, *J* = 5.2 Hz, 8.4 Hz, 1H), 3.62 (dd, *J* = 6.0 Hz, 8.4 Hz, 1H), 2.56-2.70 (m, 2H), 2.36-2.48 (m, 2H), 2.24-2.33 (m, 2H), 2.06-2.15 (m, 1H), 1.86-1.97 (m, 2H), 1.68 (dd, *J* = 3.6 Hz, 14.0 Hz, 1H); **^{13}C-NMR** (100 MHz, $CDCl_3$) δ 214.4, 137.3, 133.1, 118.4, 116.4, 72.2, 63.1, 46.7, 45.8, 42.1, 36.3, 32.2, 29.0; **HRMS (ESI+)** m/z calculated for $C_{13}H_{18}O_2$ $[M+H]^+$: 207.1380, found 207.1382; **HPLC** (Chiralpak AD-H, hexane/isopropanol 98:02, 1.0 mL/min, λ=210 nm) tR = 8.4 min (major), 9.4 min (minor).

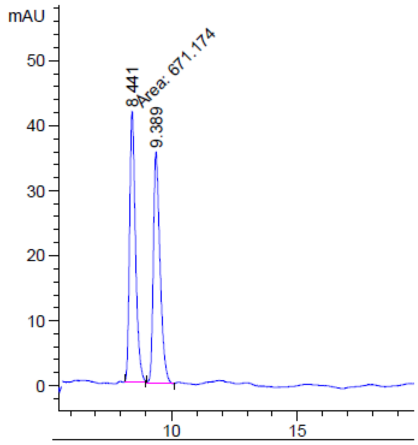

#	RetTime	Width	Area	Height	Area%
1	8.441	0.2695	671.17358	41.51271	50.0249
2	9.389	0.2882	670.50665	35.52407	49.9751

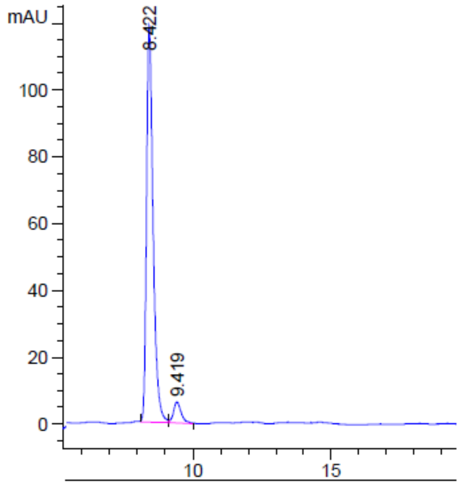

#	RetTime	Width	Area	Height	Area%
1	8.422	0.2481	1956.49976	118.84766	94.0698
2	9.419	0.2946	123.33768	6.35025	5.9302

Scheme S1: Use of a C-linked substrate in the methodology

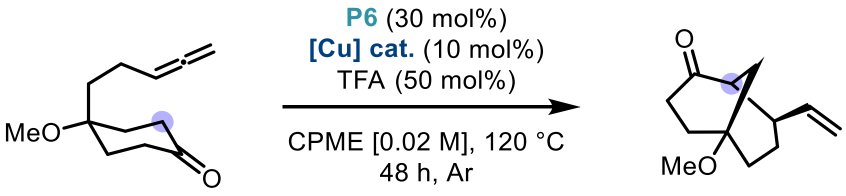

S5a **S6a**: Trace

Scheme S2: Attempted preparation of allene **S6b**

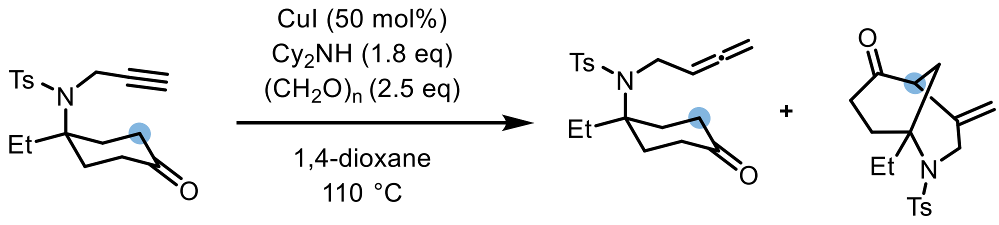

S5b **S6b** *not observed* **S6c** *observed*

7: Computational Details

7.1: Approach and Main Findings

Employ state-of-the-art density functional theory (DFT) computations that account for relativistic effects, solvation, and dispersion interactions, to evaluate the total electronic and Gibbs free energy potential energy surface (PES) associated with the proposed mechanism for the enantioselective desymmetrization of allene-tethered cyclohexanones. The current study elucidates the energetics of the complete catalytic cycle using a unified DFT approach at COSMO(diethylether)-ZORA-M06/TZ2P//COSMO(diethylether)-ZORA-BLYP-D3(BJ)/TZ2P.
Both the relative and absolute stereochemical configurations are set during the key nucleophilic attack of an enamine to copper-coordinated allene in the intramolecular cyclization step. The enantioselectivity is determined by the stabilization from a hydrogen bond between amide N-H bond and O atom on trifluoroacetate for the preference to the 5*S* configuration, and the diastereoselectivity is determined by the strain energy caused by the large dihedral angle of the enamine and the smaller angle of the allene for the preference to *exo* configuration. DFT calculations provide evidence for both a kinetic and thermodynamic preference for the formation of the 5*S*-*exo* product, which was confirmed by X-ray crystallography.

7.2: xyz Coordinates

Table S1. Cartesian coordinates (in Å), energies (in kcal mol^{-1}), and number of imaginary frequencies of all stationary points, computed at COSMO(Et_2O)-ZORA-BLYP-D3(BJ)/TZ2P. Energies (in kcal mol^{-1}) at COSMO(Et_2O)-ZORA-M06/TZ2P//COSMO(Et_2O)-ZORA-BLYP-D3(BJ)/TZ2P are also provided.

A1

COSMO(Et_2O)-ZORA-M06/TZ2P//COSMO(Et_2O)-ZORA-BLYP-D3(BJ)/TZ2P

E = -11072.01

G = -10783.10

COSMO(Et_2O)-ZORA-BLYP-D3(BJ)/TZ2P

E = -8925.01

G = -8636.10

N_{imag} = 0

C	0.06306410	-1.26086666	2.16448745
C	-0.63610709	-0.79777579	3.42416712
H	-1.91383683	0.22710625	0.25396355
H	0.08109200	-0.30195202	4.09828365
C	-1.77459477	0.21189999	3.17926356
C	-0.09033228	-0.67440354	0.94729185
C	-0.99218401	0.53530676	0.77107253
C	-1.33984567	1.21852978	2.10148200
H	-1.95225647	0.75917842	4.10585883
H	-2.12907633	1.96107626	1.95096852
H	-0.45485682	1.74671173	2.48193352
C	-5.87204773	-1.54900773	5.07355623
H	-0.51126359	1.26662050	0.11285201
H	-1.01234443	-1.65629710	3.99434037
N	-3.08573580	-0.43157466	2.81820171
C	-3.18093966	-1.74704773	2.13354488
H	-2.24871953	-1.86422157	1.57716469
H	-4.00777725	-1.70880118	1.42243760
C	-3.35757503	-2.93419353	3.06874390
C	-4.37761638	-3.74983586	3.01883866
C	-5.41597387	-4.54271988	2.97147619
S	-4.48153289	0.21255116	3.44376410
O	-5.57565882	-0.12126151	2.52808124
O	-4.19926282	1.61352478	3.77150885
H	-3.16144077	0.30757592	6.02545764
H	5.56098994	-6.41395923	3.42411058
H	7.37364441	-2.65649994	2.34877580
H	7.53491823	-4.88341238	3.45591280
N	0.50703079	-1.15787680	-0.20782884

C	1.21088385	-2.42867528	-0.22404933
H	-3.54163913	-0.94794740	8.13239975
C	0.34440105	-0.57820681	-1.55584439
H	0.60571756	0.48498615	-1.56474472
H	-0.69478721	-0.67650360	-1.90847838
C	1.31101278	-2.78597507	-1.73215465
C	1.30642661	-1.40810749	-2.42127615
H	2.19817657	-3.38277882	-1.96340825
H	0.42553529	-3.36603057	-2.01645996
H	2.30876558	-0.96943097	-2.38550753
H	0.98347352	-1.45877427	-3.46531907
C	2.62515439	-2.32369442	0.40502462
H	-5.42922104	-5.43486134	2.34567023
C	-6.08942664	-2.24220694	6.26758450
H	-6.30793241	-4.33907600	3.56428765
O	3.33522396	-1.32577499	0.26542985
N	3.00689862	-3.46842159	1.06476125
H	2.30139413	-4.19758034	1.11645702
H	5.27333656	-1.95915533	1.22554183
C	-5.24781178	-2.03315306	7.36557062
C	-4.19061094	-1.11818186	7.27702032
C	-3.96955287	-0.41393681	6.09151869
H	-2.58037156	-3.09761976	3.81589532
H	-6.50785775	-1.70705593	4.20940943
H	-6.91419767	-2.94703866	6.33755141
H	-5.41709430	-2.57745141	8.29127256
H	0.63106039	-3.18748547	0.32483030
C	4.23518273	-3.79218260	1.68759156
C	4.32606736	-5.05006533	2.31370304
C	5.50682580	-5.43895738	2.94548242
C	6.61410142	-4.58094938	2.96343838
C	6.52091946	-3.33183028	2.34131938
C	5.34365935	-2.92676205	1.70382715
H	3.46770158	-5.71972104	2.30096879
C	-4.81138619	-0.64296562	4.99590946
H	0.73001454	-2.10920606	2.28617005

A2

COSMO(Et_2O)-ZORA-M06/TZ2P//COSMO(Et_2O)-ZORA-BLYP-D3(BJ)/TZ2P

E = -11070.93

G = -10782.17

COSMO(Et_2O)-ZORA-BLYP-D3(BJ)/TZ2P

E = -8924.59

G = -8635.83

$N_{imag} = 0$

C	-0.45914742	-1.35487854	2.10733240
C	-0.88908642	-0.39520288	3.23187504
H	-1.13540340	-2.21794135	2.05226097
H	-0.01803115	0.20379934	3.52834159
C	-1.99016972	0.61161567	2.84720814
C	-0.38593724	-0.70427398	0.73846720
C	-0.91049325	0.52662757	0.51153865
C	-1.59687768	1.37267020	1.56053521
H	-2.06540065	1.34592571	3.64997540
H	-2.49791596	1.84100477	1.14290458
H	-0.94368230	2.20800906	1.86214978
H	-0.85411908	0.96148632	-0.48228527
H	0.51933647	-1.76929643	2.37719165
H	-1.19284787	-0.96863339	4.11373795
N	-3.36676411	0.03407041	2.75311554
C	-3.74605907	-0.94447802	1.70367906
H	-2.97649946	-0.84986145	0.93190717
H	-4.69955798	-0.64821813	1.26201261
C	-3.83554665	-2.38226606	2.18826947
C	-4.86675857	-3.15586222	1.97098753
C	-5.91673336	-3.90630045	1.76141163
S	-4.43902956	0.32858287	3.98874816
O	-5.76315383	-0.06413807	3.50324531
O	-4.20748483	1.69387828	4.47153001
H	-2.53818027	0.59438466	6.15751682
H	5.17114867	-6.40434396	3.96765905
H	6.81113197	-2.53904369	3.00373646
H	7.03157601	-4.74648278	4.13919541
N	0.17038546	-1.48048408	-0.27105493
C	0.86606117	-2.74110053	-0.05364498
H	-1.82531276	-0.97927198	7.94831783
C	0.36652057	-0.95617372	-1.63177483
H	0.85009381	0.02991599	-1.60083928
H	-0.60776719	-0.83383270	-2.13007138
C	1.05559107	-3.28447887	-1.49385857
C	1.25596120	-2.00434294	-2.33045323
H	1.89162079	-3.98603990	-1.56980227
H	0.13954419	-3.80721015	-1.79124548
H	2.30330676	-1.69019608	-2.28779674
H	0.98256050	-2.14596133	-3.38006905
C	2.24064368	-2.55347498	0.64376785
H	-6.00741318	-4.53014619	0.87219514
C	-4.22584492	-2.89726877	6.46306013

H	-6.74319128	-3.93442681	2.47240857
O	2.91431188	-1.53032458	0.50259497
N	2.61990627	-3.64335967	1.39269282
H	1.94955624	-4.40604668	1.42446547
H	4.76518109	-1.98750979	1.71198593
C	-3.21348352	-2.52776387	7.35668789
C	-2.60229623	-1.27277975	7.24701411
C	-2.99445936	-0.38670725	6.24034874
H	-2.98979252	-2.76955447	2.75653452
H	-5.40251109	-2.29546352	4.74719799
H	-4.70990613	-3.86624346	6.55609464
H	-2.90670850	-3.21339592	8.14269865
H	0.25435234	-3.43092272	0.54056750
C	3.81677427	-3.88359642	2.10844108
C	3.94046239	-5.13003632	2.75142258
C	5.09077524	-5.43707028	3.47724937
C	6.13431632	-4.50751075	3.57390722
C	6.00826536	-3.26964069	2.93534384
C	4.86074472	-2.94620514	2.20377336
H	3.13174307	-5.85511054	2.67751544
C	-3.99690875	-0.77525953	5.34316505
C	-4.62225999	-2.02205243	5.44818225

Cu(TFA)

COSMO(Et_2O)-ZORA-M06/TZ2P//COSMO(Et_2O)-ZORA-BLYP-D3(BJ)/TZ2P

$\boldsymbol{E}$ = -1388.43

$\boldsymbol{G}$ = -1395.06

COSMO(Et_2O)-ZORA-BLYP-D3(BJ)/TZ2P

$\boldsymbol{E}$ = -1006.03

$\boldsymbol{G}$ = -1012.66

$\boldsymbol{N}_{\mathbf{imag}}$ = 0

O	-2.31479210	-1.94800062	0.00000000
C	-2.19199413	-3.17077043	0.00000000
C	-3.46929460	-4.08519738	0.00000000
F	-4.61351488	-3.35591101	0.00000000
F	-3.49306885	-4.89334751	-1.10402120
F	-3.49306885	-4.89334751	1.10402120
O	-1.10805732	-3.88629671	0.00000000
Cu	0.45907336	-2.82914781	0.00000000

B1

COSMO(Et_2O)-ZORA-M06/TZ2P//COSMO(Et_2O)-ZORA-BLYP-D3(BJ)/TZ2P

$\boldsymbol{E}$ = -12501.77

$\boldsymbol{G}$ = -12205.60

COSMO(Et_2O)-ZORA-BLYP-D3(BJ)/TZ2P

E = -9971.05

G = -9674.88

N_{imag} = 0

C	2.43482053	-2.75469638	1.26899554
C	1.53446533	-3.71818937	0.46380757
H	2.04204917	-2.62608491	2.28767861
H	2.15346902	-4.25633931	-0.26507685
C	0.42260203	-3.03533709	-0.35289207
C	2.61122437	-1.38551657	0.63427074
C	1.99287879	-1.04888740	-0.52731820
C	1.03832467	-1.95647984	-1.26389951
H	-0.02966935	-3.79868179	-0.99087120
H	0.23503683	-1.36919154	-1.72282702
H	1.53966105	-2.47966698	-2.09486212
H	2.14448221	-0.06558490	-0.95997637
H	3.41807242	-3.22365345	1.40163176
H	1.10065436	-4.47032481	1.13144738
N	-0.70682916	-2.53332516	0.49202317
C	-0.57428678	-1.40674381	1.42177782
H	-1.38817049	-1.46796625	2.14932405
H	0.36340373	-1.51367025	1.96382528
C	-0.59223671	-0.01861668	0.77582865
C	-1.38499024	0.29092822	-0.21918621
C	-2.24106067	0.30406875	-1.26330575
S	-2.21833081	-3.12798534	0.13204341
O	-3.16459388	-2.44938034	1.02465132
O	-2.46027968	-3.10076677	-1.31415542
H	-2.38601144	-5.60509605	-1.37792348
H	4.25344346	4.65605565	-4.84706838
H	7.72007847	2.21349331	-4.08729617
H	6.53791991	3.88813901	-5.50611321
N	3.36522674	-0.48883017	1.37885806
C	3.56120025	0.88949804	0.95149376
H	2.59664827	1.35014141	0.71105797
C	4.27474955	-0.87244898	2.48112070
H	3.69842365	-1.20144006	3.35705919
H	4.93902619	-1.69360985	2.18474623
C	4.21320208	1.54901892	2.19188204
C	5.07632314	0.41329391	2.78029094
H	3.42112087	1.84375505	2.88982154
H	4.79307261	2.44082850	1.93647750
H	5.26231965	0.54054740	3.85092722
H	6.03973973	0.37505150	2.26421328

C	4.50527504	1.00445646	-0.27584355
H	-1.94316329	-0.08376462	-2.23886725
Cu	-1.27225736	2.13478422	-1.08060454
H	-3.30494364	0.49255355	-1.11268279
O	5.48064562	0.25806171	-0.41439058
N	4.17204109	2.03012254	-1.12378591
H	3.28673043	2.50575733	-0.92631366
H	6.64229899	1.31254991	-2.04335205
F	0.45987773	6.36663129	-0.43160220
F	2.50254241	5.56672491	-0.57447971
F	1.29809616	5.82174560	-2.40057167
H	0.08314184	0.72002939	1.20079032
O	-0.57755419	3.92886113	-1.18564780
O	1.46580016	3.07823830	-0.67335933
C	0.68605009	3.99749886	-0.95044752
C	1.24443472	5.45786819	-1.07749589
C	4.84247154	2.47681994	-2.28547346
C	4.17572426	3.42189795	-3.09007773
C	4.78319564	3.92585100	-4.23951004
C	6.06398570	3.49576891	-4.60976123
C	6.72480515	2.55571741	-3.81177967
C	6.12872676	2.04171621	-2.65560745
H	3.18203885	3.75735133	-2.80806629
C	-2.09319101	-4.85579493	0.61990120
C	-1.86599011	-5.15223497	1.96960677
C	-1.75809247	-6.48678793	2.36125645
C	-1.87109933	-7.51014389	1.40938194
C	-2.09549292	-7.19939483	0.06491807
C	-2.21001192	-5.86402075	-0.33911831
H	-1.77442424	-4.35243390	2.69875955
H	-1.58428119	-6.72916856	3.40649214
H	-1.78249289	-8.54855870	1.71883232
H	-2.18223971	-7.99256390	-0.67319960

B2

COSMO(Et_2O)-ZORA-M06/TZ2P//COSMO(Et_2O)-ZORA-BLYP-D3(BJ)/TZ2P

E = -12502.67

G = -12203.12

COSMO(Et_2O)-ZORA-BLYP-D3(BJ)/TZ2P

E = -9972.20

G = -9672.65

N_{imag} = 0

C	3.50256257	-2.75307816	-0.46356889
C	2.99850567	-3.27297183	-1.82892106

H	3.02188174	-3.29334066	0.36326108
H	3.80614561	-3.16100215	-2.56247954
C	1.79613408	-2.52305494	-2.43145410
C	3.29169169	-1.26919629	-0.23753462
C	2.61854388	-0.49494480	-1.12283263
C	2.03723230	-1.00316714	-2.41773777
H	1.71995764	-2.82665726	-3.47614654
H	1.08929682	-0.49807213	-2.63857867
H	2.70584379	-0.76796788	-3.26324514
H	2.48095201	0.56330054	-0.92703266
H	4.57006866	-2.99109967	-0.37936191
H	2.78535034	-4.34635677	-1.76748486
N	0.45814553	-2.87787369	-1.83544419
C	0.29208937	-3.17277402	-0.40440647
H	-0.67880682	-3.66092090	-0.28832898
H	1.05657145	-3.86839197	-0.04285613
C	0.26975749	-1.91278233	0.45433845
C	0.64413497	-1.93183805	1.70979333
C	1.11521534	-2.23470619	2.93839780
S	-0.61081558	-3.63485563	-2.88516168
O	-1.88397075	-3.77984351	-2.17536280
O	-0.55951982	-2.91050292	-4.15456739
H	1.05632235	-4.72201988	-4.99691765
H	2.39806208	7.04632177	-0.72972945
H	6.33443845	5.65555223	-1.79355090
H	4.62165891	7.46566493	-1.78772999
N	3.73348988	-0.79983173	0.99377356
C	3.45671632	0.55768090	1.43575909
H	2.38031487	0.75532298	1.35381027
C	4.72141231	-1.48645440	1.84999357
H	4.27591162	-2.37481883	2.32483238
H	5.59048898	-1.82227787	1.27242111
C	3.93082838	0.55869832	2.90939757
C	5.11745397	-0.42613047	2.89800510
H	3.11968544	0.18943574	3.54993916
H	4.20280889	1.55727469	3.26222872
H	5.30677667	-0.86825801	3.88056516
H	6.02257279	0.09367247	2.57142892
C	4.21859114	1.64162870	0.62596738
H	0.47386115	-2.69953678	3.68978069
Cu	0.51809220	-0.25024451	2.89821861
H	2.18496325	-2.21462122	3.15023976
O	5.38101555	1.47399645	0.24332328
N	3.48080989	2.78359029	0.44647399

H	2.52074556	2.75072894	0.79741560
H	5.84073519	3.45324710	-0.75880252
F	1.21686758	4.14383152	4.23415770
F	0.12255449	4.93010367	2.49224408
F	-0.98140657	3.95924053	4.12640251
H	-0.13418346	-1.01652646	-0.00899728
O	0.13920384	1.53305428	3.48321544
O	0.72044146	2.49503200	1.50181987
C	0.35753320	2.51755897	2.68316988
C	0.16187795	3.90780484	3.38598113
C	3.84415042	4.00777124	-0.16043952
C	2.87541500	5.03108390	-0.15742042
C	3.15583443	6.26619428	-0.74033171
C	4.40315345	6.50232778	-1.33344080
C	5.36275200	5.48460736	-1.33515028
C	5.09764268	4.23952982	-0.75501285
H	1.90760930	4.84887283	0.30441578
C	0.07679454	-5.27425517	-3.15434106
C	-0.15624292	-6.27272329	-2.19933389
C	0.45895190	-7.51687110	-2.35510366
C	1.29810420	-7.75544113	-3.45077641
C	1.51617797	-6.75209119	-4.40142307
C	0.90530314	-5.50289478	-4.25904627
H	-0.81810920	-6.08459105	-1.35991826
H	0.27866970	-8.30032145	-1.62346117
H	1.77484262	-8.72547626	-3.56694339
H	2.15802462	-6.94039237	-5.25823757

B3

COSMO(Et_2O)-ZORA-M06/TZ2P//COSMO(Et_2O)-ZORA-BLYP-D3(BJ)/TZ2P

E = -12498.51

G = -12201.52

COSMO(Et_2O)-ZORA-BLYP-D3(BJ)/TZ2P

E = -9967.37

G = -9670.38

$\boldsymbol{N}_{\mathbf{imag}}$ = 0

C	-3.45462436	-3.38357884	1.29359249
C	-4.37184920	-3.26065650	2.51924164
H	-4.05930477	-3.43631448	0.37488269
H	-3.80467340	-3.50890221	3.42583217
C	-4.91558057	-1.83616159	2.70318530
C	-2.46218128	-2.23568184	1.18171913
C	-2.59098856	-1.11463382	1.94140354
C	-3.72690834	-0.88139003	2.91359475

H	-5.54523557	-1.80764061	3.59225999
H	-4.07381545	0.15776550	2.86777391
H	-3.39107186	-1.02961329	3.95252491
H	-1.86390798	-0.31323762	1.85071805
H	-2.91836734	-4.33756193	1.35298200
H	-5.19244630	-3.98180580	2.45646057
N	-5.80705431	-1.40403754	1.57048655
C	-5.32969675	-0.72904839	0.33830482
H	-6.09171239	-0.87187921	-0.43346686
H	-4.42630482	-1.23358217	-0.00226563
C	-5.02250427	0.75190949	0.49098667
C	-5.87371811	1.73582758	0.32948407
C	-6.49408100	2.92936735	0.21042403
S	-7.44563125	-1.61739125	1.67228514
O	-8.13589504	-0.33647524	1.41473061
O	-7.72754811	-2.30971879	2.93003696
H	-6.59484052	-4.29802654	1.01298935
H	-0.81238043	-10.51326816	-0.53593940
H	1.65190739	-8.76711987	2.53630934
H	0.80362044	-10.78711748	1.34889275
N	-1.47897959	-2.37515116	0.21490217
C	-1.23378319	-3.58805984	-0.55315291
H	-2.16347346	-3.97305798	-0.98899586
C	-0.43119326	-1.36086875	0.02001348
H	-0.87618996	-0.43751166	-0.38301624
H	0.04745350	-1.10972922	0.97624137
C	-0.26923946	-3.10267741	-1.66720105
C	0.56244426	-2.01184140	-0.96232598
H	-0.86534956	-2.67513571	-2.48126953
H	0.33949976	-3.91404131	-2.07664916
H	0.98269588	-1.28808148	-1.66636910
H	1.38698346	-2.46984818	-0.40742796
C	-0.56909495	-4.70655871	0.29462475
H	-6.42570325	3.50934517	-0.71066574
Cu	-7.87540064	1.43758837	-0.15257507
H	-6.89544702	3.43999198	1.08785667
O	0.15528604	-4.45514793	1.26014364
N	-0.85048609	-5.97112962	-0.16610912
H	-1.48361485	-6.01714537	-0.95927398
H	0.89970308	-6.50130097	1.85947453
F	-13.05036840	1.24094842	-0.79027763
F	-11.87339408	-0.53183015	-1.34859233
F	-11.87208503	1.25289747	-2.64861525
H	-3.99232670	1.00723613	0.74528954

O	-9.52410000	0.96397666	-1.07944580
O	-10.79450677	2.16534100	0.38379847
C	-10.62379037	1.40373774	-0.56947648
C	-11.87354747	0.84057728	-1.33929756
C	-0.37703591	-7.22854470	0.28029128
C	-0.85638767	-8.36943066	-0.39080557
C	-0.43331003	-9.64180714	-0.00740873
C	0.47313237	-9.79572732	1.04945083
C	0.94775587	-8.66043039	1.71414048
C	0.53272288	-7.37743847	1.34209120
H	-1.56001890	-8.25318644	-1.21351350
C	-7.85780651	-2.69799315	0.28884350
C	-8.74965834	-2.26211121	-0.69467283
C	-9.06125721	-3.12301228	-1.75330308
C	-8.48857064	-4.39622551	-1.81940228
C	-7.59964598	-4.82133021	-0.82215087
C	-7.27945576	-3.97242889	0.23747052
H	-9.18949781	-1.27299465	-0.64368437
H	-9.75274321	-2.79123219	-2.52329553
H	-8.73346963	-5.06029572	-2.64462604
H	-7.15776751	-5.81332963	-0.86920980

B4

COSMO(Et_2O)-ZORA-M06/TZ2P//COSMO(Et_2O)-ZORA-BLYP-D3(BJ)/TZ2P

E = -12498.23

G = -12198.30

COSMO(Et_2O)-ZORA-BLYP-D3(BJ)/TZ2P

E = -9968.86

G = -9668.93

$\boldsymbol{N_{imag}}$ = 0

C	-0.01928831	-1.90103415	2.33776090
C	-0.33439492	-1.36458486	3.75360405
H	-0.63004440	-2.78319491	2.11092821
H	0.56680532	-0.87572754	4.14269245
C	-1.43369097	-0.29153640	3.84850820
C	-0.20456133	-0.88730099	1.22697718
C	-0.73052541	0.33608724	1.46250528
C	-1.15681492	0.82682706	2.82223725
H	-1.36972160	0.14267218	4.84667968
H	-2.03922172	1.46595715	2.72357569
H	-0.37954753	1.47569691	3.25982586
H	-0.86705338	1.03160101	0.64013323
H	1.01787446	-2.25824449	2.34626470
H	-0.55117045	-2.19812838	4.43087662

N	-2.82289308	-0.87028073	3.79482742
C	-3.18545764	-2.04391498	2.98993632
H	-4.18459319	-2.35540846	3.30607548
H	-2.51334629	-2.86866697	3.24365855
C	-3.17144191	-1.81297189	1.47904145
C	-2.87353009	-2.78005964	0.64436513
C	-2.47813765	-3.97193403	0.12113621
S	-4.08474002	0.01707805	4.44734167
O	-3.49671008	0.95971993	5.39907487
O	-5.10541265	-0.93743590	4.89016959
H	-5.98686275	-0.70303066	2.37946573
H	6.43212517	-6.29316769	1.61321831
H	7.31818988	-2.08010695	1.77350863
H	8.04885979	-4.44420621	2.06745764
N	0.09711083	-1.33262383	-0.06489240
C	0.98002707	-2.46935209	-0.32146047
H	-6.88306089	0.55985803	0.43642298
C	0.15013861	-0.36370258	-1.18673302
H	0.69211850	0.54046777	-0.88003601
H	-0.86757932	-0.06964057	-1.46678942
C	0.95554177	-2.57470912	-1.86483407
C	0.89339711	-1.10198664	-2.32292284
H	1.81975729	-3.11403852	-2.26269242
H	0.05078759	-3.11338954	-2.16730382
H	1.90409689	-0.69748743	-2.42827118
H	0.38809044	-0.99709008	-3.28614336
C	2.42670228	-2.24445326	0.18995351
H	-3.19959314	-4.76311828	-0.08737058
Cu	-2.62427917	-2.53314560	-1.33228170
H	-1.42579543	-4.26130415	0.10577810
O	2.91915232	-1.11862317	0.28822399
N	3.08228900	-3.41667942	0.48701050
H	2.53971173	-4.26623496	0.36199838
H	5.00655410	-1.55999588	1.03535208
F	-1.50698615	0.01925513	-5.02987370
F	-3.71222316	-0.01048768	-5.10781429
F	-2.55912527	-1.64694817	-6.01638053
H	-3.41572115	-0.81625909	1.12587055
O	-2.18628215	-2.73056057	-3.54774928
O	-3.01258263	-0.89059383	-2.57987462
C	-2.61219061	-1.54722055	-3.59210569
C	-2.60992757	-0.79544032	-4.95914268
C	4.41693676	-3.63107343	0.90762596
C	4.82915004	-4.96682307	1.07473059

C	6.12855264	-5.25625528	1.48960223
C	7.03562429	-4.21954724	1.74394426
C	6.62295977	-2.89353673	1.57816772
C	5.32301185	-2.58645027	1.16277259
H	4.12689373	-5.77477723	0.87599418
C	-4.81941703	0.96731431	3.10409416
C	-4.46684611	2.31076793	2.93720785
C	-4.98722048	3.01942672	1.85062810
C	-5.84521551	2.38712016	0.94374217
C	-6.20454728	1.04710068	1.13179147
C	-5.69766751	0.33021583	2.21826357
H	-3.80701831	2.79124339	3.65195127
H	-4.72151730	4.06460607	1.71480266
H	-6.24136315	2.94033293	0.09597394
H	0.58036395	-3.38003956	0.14025578

TSB1-C1

COSMO(Et_2O)-ZORA-M06/TZ2P//COSMO(Et_2O)-ZORA-BLYP-D3(BJ)/TZ2P

E = -12485.85

G = -12187.55

COSMO(Et_2O)-ZORA-BLYP-D3(BJ)/TZ2P

E = -9962.67

G = -9664.37

N_{imag} = 1, 214i cm^{-1}

C	2.21679112	-3.06198958	0.82082131
C	1.07427248	-3.82740668	0.09316514
H	2.04996602	-3.08149317	1.90547895
H	1.51775976	-4.47784937	-0.66870760
C	0.03894836	-2.95855997	-0.65379639
C	2.41767110	-1.61920274	0.40003193
C	1.69423711	-1.06790937	-0.62762765
C	0.76887745	-1.88854382	-1.48285302
H	-0.50905447	-3.60340091	-1.34235845
H	0.04225542	-1.24585341	-1.98601407
H	1.32265372	-2.41077972	-2.27953747
H	1.87865404	-0.04681632	-0.93864586
H	3.15844531	-3.60510397	0.66785992
H	0.56528038	-4.48715210	0.80314524
N	-1.01009492	-2.34260507	0.21764546
C	-0.62527239	-1.61932142	1.44033415
H	-1.51586220	-1.50323346	2.06163410
H	0.10711997	-2.19149275	2.01624114
C	-0.06404383	-0.24634943	1.09756230
C	-0.82812527	0.67655384	0.44567975

C	-1.67562268	0.73326208	-0.61070095
S	-2.54475477	-3.00575450	0.13111061
O	-3.41115184	-2.18838496	0.98102326
O	-2.86392291	-3.18798043	-1.28490129
H	-2.27202486	-5.65407962	-1.01931585
H	4.76991767	5.65539355	-3.56036274
H	6.71259311	2.00613127	-4.77282423
H	6.22950503	4.41888832	-5.16671410
N	3.27411380	-0.88011345	1.17060388
C	3.53398499	0.53734104	0.91843227
H	2.59254635	1.09610900	0.84904604
C	4.12654598	-1.42607980	2.25392520
H	3.51704650	-1.65487238	3.13960776
H	4.62225048	-2.34690666	1.93180553
C	4.34881078	0.98118097	2.15860454
C	5.12660495	-0.29290144	2.53959335
H	3.65351017	1.26931649	2.95515636
H	4.99287259	1.83750346	1.94457210
H	5.45847714	-0.29177021	3.58146335
H	6.00405052	-0.40266507	1.89445436
C	4.33799172	0.75350231	-0.39399224
H	-1.54703168	0.10355553	-1.49198312
Cu	-0.34291275	2.54240464	0.62596622
H	-2.49149716	1.45228758	-0.65639628
O	5.10192136	-0.10711693	-0.84154522
N	4.12376979	1.99057713	-0.93717973
H	3.48935594	2.60468233	-0.41708658
H	5.75646723	0.83766048	-2.80324913
F	2.91935771	6.21605953	1.87212171
F	2.52372665	6.48901568	-0.28116647
F	0.94876608	6.91934741	1.19591769
H	0.82058762	0.05347292	1.64636618
O	0.26193273	4.37734463	0.83047805
O	2.44008340	3.73850140	0.63277337
C	1.52702128	4.56507128	0.77713398
C	1.96745953	6.06737821	0.90161072
C	4.72338932	2.58502719	-2.07200656
C	4.44960216	3.94894829	-2.29496362
C	4.98920264	4.60187874	-3.40246586
C	5.80861611	3.90914683	-4.30351050
C	6.07878855	2.55518774	-4.07974985
C	5.54518895	1.88496129	-2.97375501
H	3.81819774	4.48783031	-1.59324288
C	-2.38715658	-4.63322140	0.87734098

C	-2.34204405	-4.73346780	2.27417734
C	-2.12162492	-5.98337324	2.85635479
C	-1.94764573	-7.11499534	2.04936008
C	-2.00272045	-7.00134297	0.65598660
C	-2.22180888	-5.75577420	0.05970283
H	-2.48966450	-3.85376739	2.89273866
H	-2.09065005	-6.07371378	3.93910255
H	-1.77653231	-8.08565988	2.50790848
H	-1.87801624	-7.88115822	0.03002285

TSB2-C2

COSMO(Et_2O)-ZORA-M06/TZ2P//COSMO(Et_2O)-ZORA-BLYP-D3(BJ)/TZ2P

E = -12484.83

G = -12185.32

COSMO(Et_2O)-ZORA-BLYP-D3(BJ)/TZ2P

E = -9960.61

G = -9661.10

N_{imag} = 1, 237i cm^{-1}

C	3.25723199	-1.52364274	-2.03694597
C	2.66143691	-0.79852189	-3.26681777
H	2.98696138	-2.58939736	-2.04153951
H	3.35417327	-0.00434067	-3.56985102
C	1.30202820	-0.10069526	-3.04900486
C	2.85894885	-0.94542300	-0.69763529
C	1.91701240	0.04706042	-0.60031174
C	1.35400847	0.77797094	-1.78996817
H	1.11437914	0.55031252	-3.90401944
H	0.34712134	1.14877194	-1.56863862
H	1.96432543	1.66668784	-2.01971368
H	1.65370229	0.44437122	0.37388578
H	4.35005705	-1.50823267	-2.12257945
H	2.60303102	-1.49416708	-4.10920777
N	0.11157857	-1.01257464	-2.99600134
C	0.14527707	-2.20337792	-2.12667388
H	-0.74102702	-2.80473730	-2.37446893
H	1.01882515	-2.83079935	-2.33128944
C	0.02264728	-1.91123369	-0.65265091
C	0.36875833	-2.85780727	0.26829705
C	0.34287039	-4.21526376	0.33043563
S	-0.78085234	-1.14554982	-4.41398856
O	-1.93791119	-1.98896418	-4.10421809
O	-0.97675236	0.20931481	-4.92853998
H	0.90268009	-0.23945371	-6.59106080
H	2.81110366	4.97733443	5.28391983

H	6.37138819	4.88124066	2.85969473
H	4.92149650	6.06333318	4.50623261
N	3.41947785	-1.53334478	0.40519580
C	3.30156625	-0.94138645	1.74252422
H	2.25172565	-0.77251838	1.98858001
C	4.47264752	-2.57978004	0.35614196
H	4.02884149	-3.53755694	0.05956110
H	5.24887783	-2.32173226	-0.37091181
C	3.92767916	-2.01207397	2.66325997
C	5.04028462	-2.62477356	1.78960699
H	3.16290910	-2.75899832	2.90731549
H	4.29823825	-1.58866179	3.60056250
H	5.29862294	-3.64250665	2.09462587
H	5.94115922	-2.00733292	1.84834270
C	4.06601978	0.40454756	1.84353126
H	-0.39458827	-4.80193531	-0.22679604
Cu	0.45595865	-2.42890466	2.16008058
H	0.99540059	-4.78657857	0.98817766
O	5.11511355	0.60754382	1.22339347
N	3.47180175	1.27857429	2.71378715
H	2.57746659	0.97386960	3.10911984
H	5.72676581	2.64543398	1.99430731
F	2.20675197	-0.79786075	6.26227585
F	0.61838914	0.73073610	6.30332223
F	0.11403922	-1.37826947	6.65840080
H	-0.52700130	-1.01808100	-0.36982353
O	0.49189212	-1.95950419	4.04413164
O	0.89352883	0.24696564	3.64438322
C	0.72914617	-0.74496068	4.37246217
C	0.89738550	-0.54461168	5.92033657
C	3.90935542	2.54962618	3.15204072
C	3.08933656	3.21745919	4.08307985
C	3.45341283	4.47422639	4.56478482
C	4.63766584	5.08369705	4.12956696
C	5.44973857	4.41802742	3.20546413
C	5.09859917	3.15745353	2.71110280
H	2.17150603	2.74246989	4.42341466
C	0.26826361	-2.03186095	-5.57228924
C	0.39090617	-3.42217140	-5.44732653
C	1.29477674	-4.09878564	-6.26954246
C	2.06262044	-3.39167181	-7.20328509
C	1.92120314	-2.00469797	-7.32573007
C	1.02230270	-1.31459529	-6.50750449
H	-0.21809306	-3.96485091	-4.73135769

H	1.39520066	-5.17771990	-6.18379545
H	2.76572986	-3.92322667	-7.83949001
H	2.50926221	-1.45750523	-8.05794675

TSB3-C3

COSMO(Et_2O)-ZORA-M06/TZ2P//COSMO(Et_2O)-ZORA-BLYP-D3(BJ)/TZ2P

E = -12466.82

G = -12166.27

COSMO(Et_2O)-ZORA-BLYP-D3(BJ)/TZ2P

E = -9946.99

G = -9646.44

N_{imag} = 1, 289*i* cm^{-1}

C	-3.47176145	-4.03080736	1.08729514
C	-4.67733559	-4.11858626	2.04732796
H	-3.73721462	-4.43978837	0.10222488
H	-4.30109524	-4.21763928	3.07275901
C	-5.60283354	-2.89068919	2.05328139
C	-2.93151141	-2.63086694	0.88936674
C	-3.62231256	-1.52178569	1.34003363
C	-4.78865619	-1.60450495	2.27833404
H	-6.29605318	-3.02016897	2.88593580
H	-5.43022430	-0.72577468	2.13841931
H	-4.45474758	-1.58175999	3.32669025
H	-3.19863244	-0.53830764	1.17765999
H	-2.67392883	-4.67994453	1.46667464
H	-5.25767863	-5.02095547	1.83019908
N	-6.47634758	-2.82095616	0.83814823
C	-5.89066661	-2.54984601	-0.50770786
H	-6.73379486	-2.42997189	-1.19695368
H	-5.38391051	-3.47056783	-0.80718738
C	-4.94855701	-1.37674655	-0.72442338
C	-5.34920718	-0.05278808	-0.73654584
C	-4.96167915	0.92102470	-1.61962297
S	-8.02940991	-2.27292362	1.19068439
O	-8.08186924	-0.81356514	1.42319450
O	-8.53393516	-3.11252132	2.28286459
H	-8.57962986	-4.76082880	-0.15461250
H	1.64229414	-9.93806893	0.39034734
H	2.62750522	-7.33255968	3.67687103
H	2.80150104	-9.55770190	2.56951328
N	-1.81962731	-2.50286059	0.11174674
C	-1.07601792	-3.62426712	-0.46956328
H	-1.75797756	-4.31332799	-0.97825106
C	-1.12923717	-1.20466831	-0.10183877

H	-1.75160292	-0.55460465	-0.72727607
H	-0.97046268	-0.70522611	0.85993653
C	-0.12561510	-2.92986061	-1.47833767
C	0.19017044	-1.58470829	-0.79527054
H	-0.66450895	-2.77452498	-2.41953103
H	0.76493497	-3.52854060	-1.68714057
H	0.51357390	-0.82016070	-1.50623236
H	0.97832250	-1.71820967	-0.04831866
C	-0.26784235	-4.39729289	0.60868840
H	-4.56825573	0.67657483	-2.61279464
Cu	-6.99890326	0.68667460	-0.07236486
H	-5.07013146	1.98353766	-1.41095910
O	0.12491135	-3.85583802	1.64365714
N	-0.02424218	-5.70000426	0.25698897
H	-0.43397751	-6.00449282	-0.62133228
H	1.31630695	-5.50548687	2.62962901
F	-10.85445592	3.61264127	2.00010932
F	-11.38735357	1.78514264	0.89597133
F	-10.75113244	3.59528070	-0.19675606
H	-4.00256566	-1.64878171	-1.19181809
O	-8.70627593	1.69181188	-0.07377992
O	-8.35043094	2.59321275	1.99176080
C	-9.02315883	2.33936624	0.98696325
C	-10.51278923	2.84473638	0.92835471
C	0.74255290	-6.69067177	0.92026774
C	0.83876401	-7.94730977	0.29431037
C	1.57615414	-8.97238415	0.88570559
C	2.22635046	-8.75915781	2.10787206
C	2.12753009	-7.50932858	2.72736304
C	1.39146586	-6.47074714	2.14742062
H	0.33490174	-8.11514914	-0.65601413
C	-8.93722717	-2.63391499	-0.31005304
C	-9.54838285	-1.58590088	-1.00536019
C	-10.28773595	-1.88757230	-2.15366959
C	-10.40727366	-3.21213506	-2.58681525
C	-9.79596724	-4.25065415	-1.87079329
C	-9.05764634	-3.96755906	-0.72134766
H	-9.45492513	-0.56267067	-0.65459545
H	-10.76875182	-1.08493814	-2.70622253
H	-10.98095188	-3.43893916	-3.48194672
H	-9.89671447	-5.27945793	-2.20636065

TSB4-C4

COSMO(Et_2O)-ZORA-M06/TZ2P//COSMO(Et_2O)-ZORA-BLYP-D3(BJ)/TZ2P

E = -12478.27

G = -12177.78

COSMO(Et_2O)-ZORA-BLYP-D3(BJ)/TZ2P

E = -9955.71

G = -9655.22

N_{imag} = 1, 123*i* cm^{-1}

C	0.16793960	-1.34686056	2.00315352
C	-0.10214769	-0.45202455	3.23932911
H	-0.49289005	-2.22595981	1.99642590
H	0.82927728	0.07000446	3.48968795
C	-1.17111902	0.64845815	3.06651785
C	0.01143339	-0.63748733	0.67916155
C	-0.64041856	0.56634695	0.59601429
C	-0.92822983	1.44767146	1.77902573
H	-1.09398760	1.32970164	3.91459747
H	-1.79713967	2.08389383	1.57890950
H	-0.07914498	2.13059170	1.94099852
H	-0.79222514	1.01022494	-0.38329581
H	1.18181675	-1.74564124	2.09771718
H	-0.34951148	-1.08189507	4.10039975
N	-2.58657086	0.16818513	3.08887313
C	-2.94468046	-1.01997234	2.29849125
H	-4.00015083	-1.26094505	2.51672668
H	-2.37852619	-1.90285254	2.60987647
C	-2.90348503	-0.85105413	0.80485623
C	-2.88898277	-1.99569670	0.01697348
C	-3.52413274	-3.17849242	0.21725786
S	-3.41043932	0.27226719	4.55476511
O	-2.93556141	1.48694276	5.22549102
O	-3.37553576	-0.99390612	5.30067136
H	-5.86354211	-1.18171503	5.04022882
H	5.56992494	-6.75800540	2.83323568
H	7.16658906	-2.77943263	2.40627007
H	7.42855846	-5.12924476	3.19316042
N	0.41920426	-1.28249327	-0.46723942
C	1.12848039	-2.57363862	-0.44777756
H	-8.20330134	-0.80428102	4.25055519
C	0.80851157	-0.49689553	-1.68949531
H	1.41675410	0.35779172	-1.37656328
H	-0.08244972	-0.12195218	-2.19590293
C	1.31841023	-2.87868676	-1.95133344
C	1.60481328	-1.48991508	-2.56218773
H	2.12209136	-3.59859264	-2.12779133
H	0.38746503	-3.29336381	-2.35013465

H	2.67259416	-1.26398316	-2.50046917
H	1.30389694	-1.44108828	-3.61038598
C	2.51263755	-2.47264134	0.24631258
H	-4.37490268	-3.26127263	0.90013277
Cu	-2.03360331	-1.90641327	-1.70125872
H	-3.28527517	-4.07643819	-0.34692503
O	3.15853816	-1.42263724	0.27029298
N	2.92957770	-3.66696135	0.77717482
H	2.27385804	-4.43849047	0.69616431
H	5.08034790	-2.05762270	1.27374640
F	-0.02583004	-1.73872767	-5.97866110
F	-1.39350887	-0.12155231	-6.57754570
F	-2.16389709	-2.16586314	-6.32926970
H	-3.15794965	0.12076789	0.39287687
O	-1.59236824	-2.04220651	-3.59957911
O	-1.74542039	0.21001588	-3.92340393
C	-1.58595933	-0.95891471	-4.29123013
C	-1.29723588	-1.24101151	-5.81126762
C	4.15082764	-3.99949424	1.41428683
C	4.29719885	-5.32670861	1.85905017
C	5.47087223	-5.72927374	2.49511945
C	6.51354201	-4.81598253	2.69677290
C	6.36406775	-3.49764277	2.25455754
C	5.19319205	-3.07795477	1.61446771
H	3.48869652	-6.03874537	1.70279912
C	-5.09305784	0.52364502	3.97152595
C	-5.35898770	1.60696696	3.12493438
C	-6.66696200	1.81291185	2.68487854
C	-7.69031363	0.94471377	3.09002377
C	-7.40943947	-0.13070674	3.93874936
C	-6.10206783	-0.34919041	4.38655410
H	-4.55560877	2.26704693	2.81266110
H	-6.88758812	2.64744312	2.02443792
H	-8.70654905	1.10736181	2.73982646
H	0.51276561	-3.32838172	0.04931759

C1

COSMO(Et_2O)-ZORA-M06/TZ2P//COSMO(Et_2O)-ZORA-BLYP-D3(BJ)/TZ2P

E = -12520.16

G = -12216.89

COSMO(Et_2O)-ZORA-BLYP-D3(BJ)/TZ2P

E = -9989.06

G = -9685.79

N_{imag} = 0

C	2.03681123	-2.92131409	0.85524139
C	1.12639792	-3.60221610	-0.19754548
H	1.54093956	-2.91611590	1.83821678
H	1.76424459	-4.02886711	-0.98033372
C	0.12681975	-2.66519795	-0.91274473
C	2.39234463	-1.48968176	0.56436100
C	1.45087114	-0.65337989	-0.24039731
C	0.87491516	-1.43541777	-1.43245779
H	-0.31952544	-3.20737727	-1.74735394
H	0.19823278	-0.77610419	-1.98283028
H	1.67892363	-1.74812188	-2.10732001
H	1.94805678	0.24380716	-0.61024607
H	2.94375360	-3.51545254	0.99512265
H	0.60915582	-4.43864213	0.27723235
N	-1.01065895	-2.17294867	-0.08399472
C	-0.69209106	-1.29626025	1.06823287
H	-1.62560072	-0.86204004	1.42636897
H	-0.26744883	-1.88650094	1.89267596
C	0.27652052	-0.14760052	0.69489621
C	-0.32369942	1.10261947	0.04083269
C	-1.52962482	1.05434960	-0.55553189
S	-2.39125740	-3.10693843	0.02067087
O	-3.42674294	-2.25708793	0.60854369
O	-2.59231792	-3.71861133	-1.29344135
H	-1.70594632	-5.89895243	-0.34238827
H	5.59430632	5.44536597	-3.58002840
H	5.95954950	1.54407071	-5.37109313
H	6.16904103	4.02052700	-5.54871354
N	3.44815097	-0.95325441	1.10917310
C	3.87707657	0.45586503	0.87435212
H	3.03440679	1.13716782	1.02313212
C	4.43688203	-1.67382901	1.97798029
H	3.96396719	-1.85021788	2.95011541
H	4.68904944	-2.63389455	1.52521428
C	4.98330044	0.67953062	1.92653031
C	5.62699435	-0.71100603	2.07278857
H	4.52315969	1.00150440	2.86663824
H	5.68566537	1.45280715	1.61127641
H	6.16393160	-0.83127848	3.01637453
H	6.32391445	-0.89895431	1.25011414
C	4.39554313	0.57237558	-0.59336778
H	-2.13473504	0.14700194	-0.61447509
Cu	0.71416014	2.69493111	0.16382526
H	-1.96995946	1.94010741	-1.01138563

O	4.68062036	-0.43178581	-1.25533301
N	4.48591375	1.86480365	-0.99542348
H	4.18872041	2.57351244	-0.30400128
H	5.18481931	0.49711579	-3.26069233
F	3.80583540	6.17483633	2.33364640
F	4.09062885	6.52155258	0.17258690
F	2.14126965	6.93030085	1.11275030
H	0.72937316	0.16571649	1.64376729
O	1.60365352	4.41817694	0.41275589
O	3.71944395	3.74675879	0.95535296
C	2.80213082	4.57901708	0.80349456
C	3.19472059	6.06974005	1.11387491
C	4.95271976	2.37953640	-2.23011554
C	5.07217951	3.77904297	-2.32860002
C	5.50692338	4.36330502	-3.51774825
C	5.82980633	3.56440254	-4.62199633
C	5.71180640	2.17443208	-4.51997497
C	5.27606603	1.57248037	-3.33511906
H	4.82880955	4.40006757	-1.47078016
C	-2.03492937	-4.42248038	1.19476810
C	-2.03571843	-4.12954702	2.56509755
C	-1.66379234	-5.12626388	3.47157569
C	-1.30447255	-6.39874520	3.01131260
C	-1.32374675	-6.68292316	1.64068162
C	-1.68630416	-5.69337501	0.72298560
H	-2.33938150	-3.14879018	2.91630441
H	-1.66356837	-4.90994896	4.53684006
H	-1.01920129	-7.17101183	3.72120214
H	-1.05926483	-7.67511052	1.28407621

C2

COSMO(Et_2O)-ZORA-M06/TZ2P//COSMO(Et_2O)-ZORA-BLYP-D3(BJ)/TZ2P

E = -12522.53

G = -12219.64

COSMO(Et_2O)-ZORA-BLYP-D3(BJ)/TZ2P

E = -9991.86

G = -9688.97

N_{imag} = 0

C	3.21371232	-1.76904810	-2.25509914
C	2.85529451	-0.78617459	-3.40147390
H	2.61586791	-2.68820189	-2.34030570
H	3.70814137	-0.11452778	-3.55351618
C	1.62675964	0.11505890	-3.14113711
C	2.94975038	-1.23980018	-0.87511727

C	1.75364897	-0.35214173	-0.68342907
C	1.75796072	0.75872932	-1.75744935
H	1.59467941	0.89035870	-3.90799478
H	0.91884925	1.43473344	-1.56737171
H	2.68563820	1.33873224	-1.70186481
H	1.76952683	0.10157701	0.30683450
H	4.25280181	-2.08495657	-2.36134585
H	2.73460176	-1.35671295	-4.32462194
N	0.30242470	-0.57050634	-3.18579399
C	0.08197867	-1.65580208	-2.19805260
H	-0.97155872	-1.93587101	-2.23376097
H	0.66399036	-2.54256047	-2.47248822
C	0.40647276	-1.17419916	-0.76945171
C	0.37268145	-2.25608696	0.30994916
C	0.46745304	-3.56339593	-0.00462813
S	-0.47331165	-0.71276157	-4.66342646
O	-1.83334078	-1.16751798	-4.37331669
O	-0.24842464	0.54088301	-5.38377818
H	1.46506403	-0.65997862	-6.81375559
H	3.01821431	4.10761160	6.00406831
H	4.98821482	5.34281526	2.37733091
H	4.14352908	5.88463083	4.65787847
N	3.71068142	-1.58930406	0.12027110
C	3.53498898	-1.10947553	1.51836388
H	2.48223593	-1.23721106	1.80638632
C	4.91222258	-2.48750726	0.01706398
H	4.55128173	-3.50574431	-0.16181625
H	5.53291735	-2.17333676	-0.82284453
C	4.45335140	-2.03193400	2.34292880
C	5.60806357	-2.34536546	1.37559857
H	3.89997152	-2.94041361	2.60499156
H	4.77806260	-1.55406082	3.26844154
H	6.15100127	-3.25503988	1.64234033
H	6.31855403	-1.51310162	1.34559695
C	3.93093625	0.39299895	1.58667548
H	0.53642185	-3.94604058	-1.02687646
Cu	0.22189108	-1.68811645	2.12327873
H	0.46877087	-4.33273029	0.76582750
O	4.54932019	0.94326416	0.66800426
N	3.51277472	0.97027648	2.74313409
H	2.98702686	0.35973731	3.38781210
H	4.71633628	3.05951009	1.44653467
F	1.21398923	-1.34218694	7.13089486
F	0.71630073	0.73525572	6.57811421

F	-0.83078972	-0.82919488	6.50504088
H	-0.34889046	-0.41586671	-0.52514994
O	-0.03057530	-1.18424387	3.99213712
O	2.13704390	-0.71877107	4.54391617
C	0.92044980	-0.85939335	4.77291676
C	0.48364930	-0.58062393	6.25778709
C	3.71622827	2.29586426	3.20012336
C	3.23787288	2.60042211	4.48914979
C	3.39211159	3.88574925	5.00732586
C	4.02331054	4.88294072	4.25259706
C	4.49696828	4.57646912	2.97290100
C	4.34992271	3.29271136	2.43725179
H	2.75022592	1.82563831	5.07500393
C	0.36610895	-2.02662701	-5.55869305
C	0.12741904	-3.35858989	-5.19393505
C	0.85979962	-4.37313101	-5.81604047
C	1.80988274	-4.05702553	-6.79453720
C	2.02550503	-2.72358225	-7.16233887
C	1.30650808	-1.69845435	-6.54194266
H	-0.62900690	-3.59797003	-4.45365617
H	0.68129954	-5.40955940	-5.54152792
H	2.37528290	-4.85054301	-7.27657811
H	2.75260204	-2.47946182	-7.93254694

C3

COSMO(Et_2O)-ZORA-M06/TZ2P//COSMO(Et_2O)-ZORA-BLYP-D3(BJ)/TZ2P

E = -12503.51

G = -12201.49

COSMO(Et_2O)-ZORA-BLYP-D3(BJ)/TZ2P

E = -9972.15

G = -9670.13

N_{imag} = 0

C	-3.62022707	-4.18736689	0.25710988
C	-4.59945150	-4.28573422	1.45737951
H	-4.15818578	-4.39871239	-0.67761632
H	-4.00689821	-4.40609990	2.37186997
C	-5.49547156	-3.05053351	1.65270080
C	-3.02182213	-2.81767237	0.10171216
C	-3.91879821	-1.64376244	0.32064395
C	-4.65645525	-1.76900885	1.66935366
H	-6.00741767	-3.17242392	2.61042210
H	-5.29731904	-0.89353320	1.80493941
H	-3.93582383	-1.79727424	2.49468556
H	-3.35301266	-0.71469722	0.29571699

H	-2.85358390	-4.95695789	0.35111746
H	-5.21414492	-5.18240299	1.34030430
N	-6.55983858	-2.95378428	0.61129209
C	-6.14665973	-2.54368059	-0.75813129
H	-6.99602457	-2.06845161	-1.24947695
H	-5.94840282	-3.46842314	-1.31284877
C	-4.92889803	-1.58411062	-0.90251875
C	-5.30316809	-0.12693871	-1.17255048
C	-4.63133783	0.47360143	-2.17787944
S	-8.02556509	-2.42445846	1.27934073
O	-7.93989666	-1.06847610	1.83467391
O	-8.44094003	-3.48965665	2.20534902
H	-9.02192296	-4.54589600	-0.41123742
H	2.59912010	-9.47983026	1.15269925
H	1.61936338	-7.10207443	4.60915490
H	2.69819964	-9.11826881	3.62127132
N	-1.78868513	-2.65509011	-0.29019402
C	-0.85860345	-3.76825343	-0.62868700
H	-1.37609468	-4.48900336	-1.26517183
C	-1.10446396	-1.32152840	-0.45229087
H	-1.55135837	-0.81467977	-1.31319172
H	-1.26722100	-0.72129775	0.44288647
C	0.29464294	-3.05804165	-1.37526553
C	0.36592317	-1.68349581	-0.68676978
H	0.02716677	-2.95315218	-2.43197073
H	1.22770487	-3.62187128	-1.31051232
H	0.86892840	-0.93164957	-1.29857973
H	0.89125791	-1.76062210	0.27014516
C	-0.37995721	-4.45939381	0.68249011
H	-3.87204423	-0.05387558	-2.77286686
Cu	-6.66672093	0.77088455	-0.21119883
H	-4.80593690	1.51101278	-2.45924702
O	-0.53942211	-3.93979222	1.78668688
N	0.23202085	-5.65522564	0.43845284
H	0.26156103	-5.95266453	-0.53277625
H	0.45631237	-5.46035256	3.15958388
F	-9.52399850	3.28421430	3.44032571
F	-10.43626124	1.64078391	2.29634865
F	-10.09769250	3.60205486	1.34107271
H	-4.37562050	-1.94991084	-1.78385542
O	-8.17794576	1.75280558	0.53568466
O	-7.18887432	2.27972789	2.52815592
C	-8.13470020	2.19730916	1.73728981
C	-9.55287863	2.69004171	2.21311027

C	0.87517912	-6.54460556	1.34017063
C	1.48128342	-7.68299037	0.77950714
C	2.13374538	-8.60411374	1.59809760
C	2.18923949	-8.40097597	2.98258082
C	1.58339642	-7.26823960	3.53514389
C	0.92432597	-6.33479363	2.72802222
H	1.44016973	-7.84141564	-0.29670006
C	-9.11911589	-2.40385566	-0.13769004
C	-9.64915934	-1.18029589	-0.55773169
C	-10.52562870	-1.17261439	-1.64798571
C	-10.85310330	-2.36737641	-2.29751556
C	-10.31441084	-3.58500597	-1.85863600
C	-9.44357809	-3.61106232	-0.76828048
H	-9.37883210	-0.26249457	-0.04469613
H	-10.94800083	-0.23090001	-1.98852740
H	-11.53206232	-2.35308799	-3.14666253
H	-10.57584743	-4.51201947	-2.36240348

C4

COSMO(Et_2O)-ZORA-M06/TZ2P//COSMO(Et_2O)-ZORA-BLYP-D3(BJ)/TZ2P

E = -12511.80

G = -12209.35

COSMO(Et_2O)-ZORA-BLYP-D3(BJ)/TZ2P

E = -9979.75

G = -9677.30

N_{imag} = 0

C	-0.12903073	-1.65122247	2.13772238
C	-0.24182633	-0.74832771	3.39627180
H	-0.90945091	-2.42546453	2.15922374
H	0.74533180	-0.31612891	3.59788016
C	-1.23238747	0.42838165	3.26807070
C	-0.29429381	-0.92996860	0.82760498
C	-1.21545733	0.24656990	0.77156775
C	-0.96038402	1.18867610	1.96772856
H	-1.09501001	1.08993321	4.12463579
H	-1.62307751	2.05499170	1.88121070
H	0.07377338	1.55071365	1.96151432
H	-1.08970010	0.78602415	-0.16785400
H	0.82261787	-2.18451936	2.16933226
H	-0.50042385	-1.37392686	4.25544775
N	-2.66858919	0.04376474	3.26479741
C	-3.14738053	-0.82115871	2.15952477
H	-4.24099613	-0.84105511	2.18988883
H	-2.81253824	-1.85229163	2.30353840

C	-2.72748075	-0.24160978	0.79672052
C	-3.03284028	-1.12551914	-0.41343643
C	-3.07336689	-2.46830119	-0.29905233
S	-3.43856568	-0.19097702	4.73086864
O	-2.77509377	0.68479406	5.70446537
O	-3.57365941	-1.61784004	5.05774082
H	-6.07742500	-1.34515797	5.01217225
H	6.45320244	-6.38979994	1.90186652
H	7.27316294	-2.16903934	2.17503337
H	8.01337751	-4.52790185	2.47977381
N	0.28642791	-1.37474372	-0.24946127
C	1.08694874	-2.62918051	-0.30544661
H	-8.34936628	-0.43253264	4.51637053
C	0.22577484	-0.72234050	-1.60233558
H	0.50486923	0.32763439	-1.50435014
H	-0.81308021	-0.78781150	-1.95281152
C	1.15674922	-2.93728849	-1.81922310
C	1.19525304	-1.53964881	-2.46261525
H	2.02356433	-3.55359933	-2.06765103
H	0.25058281	-3.47704220	-2.11304873
H	2.20359981	-1.11814559	-2.40754364
H	0.88153161	-1.55211272	-3.50871807
C	2.49153879	-2.37817194	0.31389503
H	-2.93640513	-3.01027310	0.64172901
Cu	-3.29666528	-0.18313966	-2.04303680
H	-3.26061721	-3.10381057	-1.16331699
O	2.93443861	-1.24151202	0.48013315
N	3.14113200	-3.54250801	0.60988955
H	2.62569446	-4.40002522	0.43224436
H	5.00639656	-1.66491848	1.30487805
F	-2.77451738	2.63225745	-5.79414838
F	-4.27307763	4.03152111	-4.99205315
F	-4.91173014	2.07823866	-5.77159459
H	-3.27024623	0.70625114	0.68576803
O	-3.58327985	0.79635552	-3.70504267
O	-3.70014547	2.81987694	-2.64738225
C	-3.72527587	2.07139305	-3.63064402
C	-3.93239567	2.71381245	-5.05375261
C	4.45442622	-3.74309210	1.11195541
C	4.86908808	-5.07600025	1.28373349
C	6.14419340	-5.35532547	1.77380013
C	7.01920329	-4.31086799	2.09768371
C	6.60174075	-2.98735814	1.92576768
C	5.32585477	-2.68993658	1.43567339

H	4.19008697	-5.88880924	1.03211301
C	-5.07671217	0.44716312	4.35663743
C	-5.19149478	1.73878921	3.82823921
C	-6.46157078	2.24242851	3.54653994
C	-7.59813784	1.46083791	3.79602514
C	-7.46740296	0.17402734	4.32793756
C	-6.19904179	-0.34435058	4.61107740
H	-4.30228169	2.32891262	3.62921184
H	-6.56337312	3.23933165	3.12544355
H	-8.58543908	1.85569795	3.56977573
H	0.56216650	-3.41159101	0.24726207

CF_3COOH

COSMO(Et_2O)-ZORA-M06/TZ2P//COSMO(Et_2O)-ZORA-BLYP-D3(BJ)/TZ2P

E = -1442.28

G = -1439.08

COSMO(Et_2O)-ZORA-BLYP-D3(BJ)/TZ2P

E = -1055.78

G = -1052.58

$\boldsymbol{N}_{\mathbf{imag}}$ = 0

O	-2.24015579	-1.97840415	0.00000000
C	-2.19913822	-3.18562112	0.00000000
C	-3.47982018	-4.08594897	0.00000000
F	-4.59450778	-3.32434788	0.00000000
F	-3.50536176	-4.88635597	-1.10364457
F	-3.50536176	-4.88635597	1.10364457
O	-1.10385078	-3.96446685	0.00000000
H	-0.30966103	-3.38590417	0.00000000

D1

COSMO(Et_2O)-ZORA-M06/TZ2P//COSMO(Et_2O)-ZORA-BLYP-D3(BJ)/TZ2P

E = -13967.71

G = -13645.11

COSMO(Et_2O)-ZORA-BLYP-D3(BJ)/TZ2P

E = -11052.33

G = -10729.73

$\boldsymbol{N}_{\mathbf{imag}}$ = 0

C	6.31226208	2.39459529	0.14493317
C	6.78402007	3.72346779	0.78729421
H	6.21983623	2.51304834	-0.94602385
H	7.33450778	3.48657930	1.70495809
C	5.65649452	4.69792599	1.19515085
C	4.97772027	1.88728396	0.61564030
C	3.96284324	2.85911313	1.13131654

C	4.61074003	3.93832869	2.01488090
H	6.09006998	5.49677203	1.79803807
H	3.82810670	4.61579231	2.36642856
H	5.08663414	3.47971985	2.88811734
H	3.19374316	2.34270719	1.70703596
H	7.07786265	1.62657539	0.28410550
H	7.49311471	4.20544700	0.11085265
N	4.93896144	5.37056381	0.07430198
C	4.14871936	4.51828408	-0.84472439
H	3.53816129	5.17408492	-1.46595813
H	4.81166845	3.95748216	-1.51851207
C	3.22181998	3.53532685	-0.09034492
C	1.88484334	4.07665444	0.43166149
C	1.68167002	5.40529119	0.55618878
S	5.56899249	6.78906838	-0.54963186
O	4.51301478	7.38323680	-1.36943866
O	6.14047441	7.52875664	0.57567667
H	8.44592913	6.80708641	-0.21122128
H	-1.28998063	-1.15300931	5.84137076
H	2.53397645	-0.07384440	7.50713087
H	0.15531354	-0.73891754	7.83500839
N	4.66625722	0.63148424	0.46547094
C	3.36248469	0.04101685	0.88582970
H	2.53514273	0.64933023	0.50980580
C	5.57977170	-0.42786707	-0.07926703
H	5.67896397	-0.26284831	-1.15753482
H	6.56178073	-0.33605516	0.38718314
C	3.37516194	-1.36185997	0.24346835
C	4.86578249	-1.74516476	0.24783969
H	2.98949984	-1.29093095	-0.77893010
H	2.74469886	-2.06075466	0.79538041
H	5.10486866	-2.52086639	-0.48298252
H	5.16836409	-2.09649979	1.23912162
C	3.31761312	0.00150440	2.44621151
H	2.43820783	6.15499049	0.31762100
Cu	0.55765898	2.77135016	0.85918933
H	0.73049659	5.79621871	0.91463092
O	4.34239903	0.14184451	3.12253692
N	2.05830118	-0.20456329	2.90568131
H	1.32421649	-0.28636612	2.18181653
H	3.46415828	0.17709904	5.22172407
F	-2.26266246	-1.41646381	-0.14477611
F	-2.63396092	-0.97042199	1.98503568
F	-3.28655441	0.44200049	0.42295935

H	2.99049333	2.74200590	-0.81218531
O	-0.93989559	1.54819259	1.05492116
O	0.10420859	-0.47643347	0.88923599
C	-0.88272814	0.28692031	0.90652435
C	-2.28693774	-0.40828151	0.77682682
C	1.61248092	-0.34589149	4.24337870
C	0.26862223	-0.72273611	4.42708658
C	-0.25018186	-0.86174748	5.71381404
C	0.56045052	-0.62992679	6.83206157
C	1.89562856	-0.25721639	6.64579693
C	2.43116484	-0.11210409	5.36200566
H	-0.36017478	-0.91137872	3.56163785
C	6.91289254	6.32065880	-1.64855178
C	6.60756755	5.81833729	-2.92051372
C	7.64802870	5.37481287	-3.74152009
C	8.97367855	5.44236862	-3.29648159
C	9.26588268	5.96058643	-2.02923688
C	8.23476755	6.40050643	-1.19500244
H	5.58044649	5.79444725	-3.26947190
H	7.42241615	4.98641240	-4.73130301
H	9.77945876	5.10019154	-3.94099403
H	10.29630663	6.02672464	-1.68964178
H	0.55870215	4.52224523	-1.14449860
O	-1.76219292	4.75999495	-0.56696858
C	-1.28680872	4.53096723	-1.65679472
C	-2.17882700	4.25420983	-2.91319491
F	-3.46791900	4.58189788	-2.67003610
F	-1.75728414	4.95508116	-4.00273085
F	-2.13144536	2.92251420	-3.22647538
O	0.00459626	4.43110845	-1.98148266

D2

COSMO(Et_2O)-ZORA-M06/TZ2P//COSMO(Et_2O)-ZORA-BLYP-D3(BJ)/TZ2P

E = -13970.09

G = -13650.82

COSMO(Et_2O)-ZORA-BLYP-D3(BJ)/TZ2P

E = -11055.67

G = -10736.40

N_{imag} = 0

C	3.18573187	-1.72840578	-2.14549094
C	2.85050020	-0.69612084	-3.25605758
H	2.51634728	-2.59673932	-2.22273616
H	3.72811796	-0.05616888	-3.40372826
C	1.66352469	0.24186595	-2.94241395

C	3.01118897	-1.19905247	-0.75265893
C	1.84114827	-0.28955119	-0.50037621
C	1.85018970	0.84788033	-1.54836187
H	1.64088116	1.03617582	-3.68960796
H	1.03640913	1.54236690	-1.31887275
H	2.79634242	1.39753413	-1.50200853
H	1.90032374	0.14031519	0.49868123
H	4.19307752	-2.11647760	-2.30041680
H	2.68925848	-1.23430634	-4.19233098
N	0.31376149	-0.39111620	-2.96850238
C	0.06374300	-1.48209341	-1.99503312
H	-1.00827143	-1.68508237	-1.98829545
H	0.57403813	-2.39799118	-2.31433970
C	0.46900165	-1.06272685	-0.56769749
C	0.45249539	-2.18215600	0.47432138
C	0.47552964	-3.48438659	0.11111515
S	-0.49748316	-0.49656294	-4.43383351
O	-1.88858356	-0.81173028	-4.11286186
O	-0.16176741	0.70993909	-5.19054214
H	1.46039144	-0.71014198	-6.56164026
H	3.31741202	4.21046083	6.03715854
H	5.16784338	5.41969899	2.33930550
H	4.38352101	5.98150429	4.63641580
N	3.83050804	-1.54376299	0.19675087
C	3.73995722	-1.05492562	1.60096665
H	2.71156924	-1.19000893	1.95884730
C	5.02880611	-2.43851484	0.02642470
H	4.66283296	-3.46115707	-0.11178568
H	5.58993111	-2.13366510	-0.85732178
C	4.72120994	-1.95957524	2.37202333
C	5.81189222	-2.27171586	1.33335460
H	4.19711937	-2.87076158	2.68071732
H	5.10124848	-1.46762719	3.26862054
H	6.38185685	-3.17210711	1.57367414
H	6.50880751	-1.43214429	1.24589187
C	4.12408807	0.45262316	1.63538619
H	0.47781043	-3.83458377	-0.92401904
Cu	0.43891775	-1.66476698	2.31197556
H	0.49575048	-4.27802655	0.85645413
O	4.69886886	0.99602337	0.68521279
N	3.74960322	1.03794404	2.80220303
H	3.26079124	0.42677528	3.47399351
H	4.89373898	3.12166501	1.44519463
F	1.69172083	-1.43383904	7.25145837

F	1.18459701	0.66378275	6.78921784
F	-0.37722749	-0.88671973	6.74313627
H	-0.25822538	-0.30183532	-0.25910591
O	0.30218746	-1.21286875	4.19610056
O	2.48389742	-0.69978166	4.63538434
C	1.28694106	-0.87558264	4.92980827
C	0.92510659	-0.63893418	6.44091563
C	3.95333820	2.37084708	3.23688952
C	3.50971457	2.68608101	4.53563316
C	3.66470195	3.97953697	5.03291855
C	4.26285576	4.97345422	4.24747742
C	4.70256857	4.65582783	2.95837646
C	4.55403376	3.36383567	2.44335164
H	3.04772186	1.91376729	5.14549301
C	0.19111367	-1.91459140	-5.30012634
C	-0.21697141	-3.20227449	-4.92719071
C	0.38450981	-4.30735333	-5.53558377
C	1.37419677	-4.12327658	-6.50823729
C	1.76068191	-2.83107718	-6.88406178
C	1.17261255	-1.71744796	-6.27840795
H	-1.00431590	-3.33645652	-4.19280108
H	0.07280069	-5.31009103	-5.25498307
H	1.83665018	-4.98625939	-6.98041756
H	2.51785773	-2.68796793	-7.65070214
H	-1.53074528	-2.89224553	0.78671688
O	-2.84733626	-1.12521085	-0.20687268
C	-3.22724084	-2.03378141	0.49871976
C	-4.72703627	-2.20111381	0.91750057
F	-5.48795511	-1.22073155	0.38167108
F	-5.21794209	-3.40251686	0.49289625
F	-4.85858076	-2.15062259	2.27483569
O	-2.49848182	-3.01497671	1.03382529

TSD1-E1

COSMO(Et_2O)-ZORA-M06/TZ2P//COSMO(Et_2O)-ZORA-BLYP-D3(BJ)/TZ2P

E = -13955.88

G = -13637.03

COSMO(Et_2O)-ZORA-BLYP-D3(BJ)/TZ2P

E = -11044.63

G = -10725.78

N_{imag} = 1, 1269*i* cm^{-1}

C	6.11787322	2.28376295	-0.10804361
C	6.63616954	3.54188829	0.64167986
H	5.97097582	2.51243843	-1.17394663

H	7.19220177	3.20636704	1.52440207
C	5.54544294	4.51154099	1.15247554
C	4.80208107	1.75920300	0.38977723
C	3.79427006	2.74787707	0.89642189
C	4.46136430	3.71984386	1.88838591
H	6.00777480	5.22567797	1.83516797
H	3.70090151	4.39048400	2.29699391
H	4.90232088	3.15969864	2.71961314
H	2.97341873	2.23650036	1.39949809
H	6.88043642	1.50292579	-0.08186929
H	7.34916396	4.06181534	-0.00211237
N	4.85784394	5.32140494	0.10734004
C	4.11776128	4.57644588	-0.93414967
H	3.52638882	5.29169928	-1.50646008
H	4.81157559	4.09193273	-1.63406187
C	3.15752024	3.52765659	-0.32118953
C	1.78900396	4.08116793	0.08585740
C	1.62775835	5.31234754	0.61839134
S	5.50516433	6.80703215	-0.32201417
O	4.47781468	7.48770577	-1.10854397
O	6.02279097	7.40803688	0.90678499
H	8.36117057	6.74133134	0.15050519
H	-1.00362805	-1.00167198	6.21602391
H	2.92393968	0.21128511	7.50632890
H	0.58824215	-0.45562220	8.06106252
N	4.51197028	0.49290099	0.31193114
C	3.25184100	-0.10338292	0.84680610
H	2.38195004	0.43822324	0.46891469
C	5.43159299	-0.58206977	-0.19582234
H	5.46402128	-0.50288825	-1.28734734
H	6.43343350	-0.42462524	0.20516518
C	3.28183664	-1.55491154	0.32547901
C	4.78338112	-1.88890974	0.27727103
H	2.83880860	-1.58510260	-0.67537752
H	2.70927730	-2.22330864	0.97099894
H	5.01076308	-2.71451257	-0.40071207
H	5.15119864	-2.14695911	1.27504568
C	3.30785519	-0.01347215	2.40589889
H	2.45983669	5.97862400	0.84467034
Cu	0.35900580	2.80988441	0.60207082
H	0.63130303	5.70538815	0.80955969
O	4.37225542	0.20137592	2.99574420
N	2.09116483	-0.20732293	2.97240015
H	1.30434092	-0.32430653	2.31638502

H	3.66741749	0.33420627	5.14378729
F	-2.46025003	-1.50084067	0.64939706
F	-2.51991218	-0.67201792	2.69320104
F	-3.43728044	0.43119633	1.02000736
H	2.97547371	2.79558306	-1.11708885
O	-1.05994580	1.63685004	1.14016676
O	0.00661894	-0.38034932	1.05981056
C	-0.98688858	0.36472457	1.15072749
C	-2.37538541	-0.34227475	1.36334336
C	1.75235397	-0.26556517	4.34852761
C	0.43310676	-0.64297417	4.65988527
C	0.01873888	-0.70898108	5.98968358
C	0.91147367	-0.40315363	7.02447446
C	2.22234549	-0.02971016	6.71090607
C	2.65314328	0.04364718	5.38228484
H	-0.26010255	-0.88834467	3.86050401
C	6.89486301	6.46356296	-1.40745908
C	6.64566613	6.12737415	-2.74473960
C	7.72023159	5.77326160	-3.56505707
C	9.02317153	5.76312851	-3.05329288
C	9.25907513	6.11447869	-1.71874089
C	8.19349119	6.46396007	-0.88510158
H	5.63600353	6.16235652	-3.14085120
H	7.53881195	5.51435697	-4.60494145
H	9.85558254	5.49063855	-3.69714489
H	10.27240041	6.12029653	-1.32563219
H	0.91063077	3.81016654	-0.96794291
O	-1.47870927	4.44598175	-1.24748524
C	-0.77660646	4.13738532	-2.21137749
C	-1.40212954	4.15210571	-3.65721516
F	-2.68681122	4.59386445	-3.66078357
F	-0.68795726	4.95949146	-4.50360880
F	-1.40004648	2.89131975	-4.20109836
O	0.46193662	3.76750987	-2.22890169

TSD2-E2

COSMO(Et_2O)-ZORA-M06/TZ2P//COSMO(Et_2O)-ZORA-BLYP-D3(BJ)/TZ2P

E = -13957.95

G = -13638.03

COSMO(Et_2O)-ZORA-BLYP-D3(BJ)/TZ2P

E = -11047.10

G = -10727.18

$\boldsymbol{N}_{\mathbf{imag}}$ = 1, 1284*i* cm^{-1}

C	3.18236363	-1.59583701	-2.27169138

C	2.64451483	-0.61583389	-3.34561611
H	2.69223550	-2.57373465	-2.38354668
H	3.39240955	0.17011936	-3.50090717
C	1.32499393	0.09258197	-2.97324007
C	2.94278566	-1.16156400	-0.85257571
C	1.68444044	-0.40387580	-0.54124244
C	1.47152274	0.71863897	-1.58285982
H	1.12864876	0.87452596	-3.70803232
H	0.57168800	1.27914955	-1.31325063
H	2.32144856	1.40893238	-1.57541426
H	1.74202071	0.03917522	0.45230221
H	4.24364210	-1.78123124	-2.44558172
H	2.55018331	-1.15477639	-4.29038373
N	0.10869557	-0.77130508	-2.95557961
C	0.05226736	-1.85940332	-1.95414488
H	-0.97115581	-2.23673994	-1.92191834
H	0.69831088	-2.69889241	-2.24280075
C	0.41307187	-1.33581671	-0.54866154
C	0.46749662	-2.45906027	0.48489753
C	1.15314896	-3.60575883	0.27845910
S	-0.72749434	-1.01647445	-4.39072628
O	-2.01414409	-1.60296180	-4.02027511
O	-0.66402789	0.24163143	-5.13357392
H	1.11134937	-0.80686100	-6.61900642
H	3.27307559	3.90524842	6.26715820
H	4.87949020	5.40254518	2.56042834
H	4.20143151	5.79515563	4.92516287
N	3.78018518	-1.49384679	0.08631650
C	3.65313022	-1.07820684	1.51021765
H	2.63683620	-1.27539442	1.86333061
C	5.04889337	-2.27459014	-0.12362142
H	4.76721043	-3.30884713	-0.34714594
H	5.59578669	-1.86096463	-0.97160361
C	4.67547118	-1.96795361	2.24315873
C	5.79887280	-2.14699658	1.20763386
H	4.20326704	-2.92654902	2.48443660
H	5.01074397	-1.51190197	3.17545437
H	6.41779467	-3.02570797	1.40151034
H	6.44619335	-1.26460000	1.18813236
C	3.96414440	0.44912957	1.60697698
H	1.74724286	-3.80558325	-0.61553968
Cu	0.26693157	-1.91686921	2.38176422
H	1.12810026	-4.41683637	1.00221199
O	4.44520389	1.07093712	0.65391911

N	3.64228596	0.94953068	2.82650048
H	3.21215721	0.28452961	3.48373065
H	4.63398441	3.15530111	1.53946796
F	2.04658715	-0.62505736	7.15841473
F	0.19594884	0.36852280	6.50153792
F	0.18904511	-1.78778499	6.97656996
H	-0.39154750	-0.65212430	-0.25332969
O	0.22051733	-1.42089541	4.23224072
O	2.43719538	-0.96747414	4.52681123
C	1.25957565	-1.09297348	4.89957676
C	0.92446282	-0.79030538	6.40560211
C	3.81808927	2.26359894	3.32822185
C	3.43555750	2.48301911	4.66501960
C	3.57270823	3.74847327	5.23380850
C	4.09380147	4.80872480	4.48119122
C	4.47400206	4.58614756	3.15381533
C	4.34092452	3.32332056	2.56720073
H	3.03783030	1.65773492	5.25036810
C	0.19463867	-2.25612513	-5.31053139
C	0.08583428	-3.60178054	-4.93481074
C	0.87150997	-4.55338979	-5.59099733
C	1.74550072	-4.16186154	-6.61221615
C	1.83175581	-2.81625698	-6.98885383
C	1.05712045	-1.85321841	-6.33665401
H	-0.61404959	-3.90140227	-4.16168271
H	0.79256523	-5.60029811	-5.30980617
H	2.35159768	-4.90693973	-7.12134909
H	2.49901072	-2.51468518	-7.79209622
H	-0.83201084	-2.65611877	0.96056512
O	-2.26327322	-0.65465956	1.48440123
C	-2.75937751	-1.75134995	1.21324103
C	-4.32525648	-1.91657452	1.24186708
F	-4.95549756	-0.77574092	1.62488026
F	-4.80255629	-2.25501583	0.00237108
F	-4.70205380	-2.90730238	2.10937653
O	-2.16104077	-2.85104071	0.89127176

H_2O

COSMO(Et_2O)-ZORA-M06/TZ2P//COSMO(Et_2O)-ZORA-BLYP-D3(BJ)/TZ2P

E = -421.49

G = -419.81

COSMO(Et_2O)-ZORA-BLYP-D3(BJ)/TZ2P

E = -322.26

G = -320.58

***N*imag** = 0

H	0.76870868	0.00000000	-0.54110322
O	0.00000000	0.00000000	0.05739252
H	-0.76870868	0.00000000	-0.54110322

organocatalyst

COSMO(Et_2O)-ZORA-M06/TZ2P//COSMO(Et_2O)-ZORA-BLYP-D3(BJ)/TZ2P

E = -4770.81

G = -4650.91

COSMO(Et_2O)-ZORA-BLYP-D3(BJ)/TZ2P

E = -3852.40

G = -3732.50

***N*imag** = 0

H	8.10273013	-4.69708837	2.27087751
N	0.37998477	-1.20445832	0.00822436
C	1.17004012	-2.43852047	-0.22656400
C	6.75054090	-3.09082966	1.75242866
C	-0.25789296	-0.84458031	-1.28883402
H	-0.44837849	0.23244827	-1.32148201
H	-1.22260452	-1.36463560	-1.37396424
C	1.21739187	-2.67373313	-1.78831529
C	0.71222930	-1.34123975	-2.37878974
H	2.21972438	-2.94396363	-2.13438995
H	0.53998891	-3.49454472	-2.04787653
H	1.54193501	-0.63194883	-2.48756177
H	0.23699460	-1.47032303	-3.35745140
C	2.58643163	-2.27883676	0.34871233
C	5.46770861	-2.73105623	1.32598288
H	4.12909476	-5.86477132	1.07956910
H	0.68574247	-3.29647412	0.25763157
O	3.11827868	-1.16958572	0.48304246
N	3.19619442	-3.47119801	0.64299186
H	2.61895524	-4.29712128	0.51430925
H	5.19958710	-1.69285262	1.18423978
C	4.51565770	-3.73639688	1.08079743
C	4.86657789	-5.08681261	1.26965470
C	6.14961196	-5.42913348	1.69525878
C	7.10216370	-4.43148569	1.93903568
H	6.40455936	-6.47695653	1.83546614
H	7.48086738	-2.30681002	1.93991546
H	1.06280882	-0.47556895	0.23683444

E1

COSMO(Et_2O)-ZORA-M06/TZ2P//COSMO(Et_2O)-ZORA-BLYP-D3(BJ)/TZ2P

E = -6753.93

G = -6584.27

COSMO(Et_2O)-ZORA-BLYP-D3(BJ)/TZ2P

E = -5420.65

G = -5250.99

N_{imag} = 0

C	5.65685028	2.39323399	-0.69776966
C	6.44546948	3.28717410	0.29443662
H	5.46853033	2.93960651	-1.63318855
H	7.04764611	2.63958638	0.94328849
C	5.57542369	4.14753311	1.24159837
C	4.28570146	1.91334160	-0.22928478
C	3.52711016	2.78392633	0.76935899
C	4.48334520	3.27489408	1.87642725
H	6.21489689	4.56168548	2.02225970
H	3.93237086	3.84057741	2.63370329
H	4.95415268	2.42118242	2.37786500
H	2.72521720	2.16608919	1.18425167
H	6.23942898	1.51085959	-0.98273929
H	7.14940221	3.91682709	-0.25598510
N	4.89393630	5.31923868	0.60891823
C	3.90628343	5.02802984	-0.45595381
H	3.40525267	5.96152363	-0.71472642
H	4.40166480	4.65392853	-1.36039850
C	2.85545333	4.00951675	0.04715453
C	1.85779109	4.67917976	0.96286645
C	0.53458534	4.54671971	0.84852570
S	5.70110856	6.77986554	0.53402941
O	4.70471230	7.78885183	0.17092226
O	6.46401225	6.90929021	1.77619798
H	8.55429779	6.28569357	0.46891654
C	7.29215585	6.58906748	-3.20008997
C	6.87031864	6.65557593	-0.82649501
C	6.39999557	6.77623124	-2.14098206
O	3.79453832	0.87703853	-0.67018457
H	10.14108431	5.96546263	-1.42788841
H	0.08845524	3.93216575	0.06691825
C	8.63585204	6.29073685	-2.94460934
H	-0.14790110	5.04767357	1.53135250
H	2.27381130	5.30529571	1.75249447
C	9.09526828	6.18543555	-1.62641003
C	8.21248144	6.36452987	-0.55793919
H	5.36198840	7.02855026	-2.33148388
H	2.32033608	3.63059349	-0.83219137

H	6.93755685	6.68249535	-4.22336772
H	9.32575364	6.14725530	-3.77243760

E2

COSMO(Et_2O)-ZORA-M06/TZ2P//COSMO(Et_2O)-ZORA-BLYP-D3(BJ)/TZ2P

E = -6754.16

G = -6584.05

COSMO(Et_2O)-ZORA-BLYP-D3(BJ)/TZ2P

E = -5420.45

G = -5250.34

$\boldsymbol{N}_{\mathbf{imag}}$ = 0

C	3.02394284	-2.12441832	-2.17311514
C	2.66503781	-1.07874419	-3.26310408
H	2.44178974	-3.04328529	-2.32630652
H	3.48941714	-0.35842977	-3.33543302
C	1.38937384	-0.25305069	-2.97281973
C	2.73668980	-1.65851981	-0.75463284
C	1.44603714	-0.86541003	-0.53186817
C	1.44328890	0.30969973	-1.54466449
H	1.33524323	0.57000682	-3.68734501
H	0.57997782	0.95732980	-1.35672224
H	2.35028856	0.91469035	-1.43159460
H	1.48378603	-0.48091040	0.49222311
H	4.07907984	-2.40840801	-2.23476707
H	2.59338897	-1.57163425	-4.23558409
N	0.09916404	-0.99632528	-3.10832832
C	-0.13839749	-2.11307963	-2.16651980
H	-1.18295902	-2.41375637	-2.26071101
H	0.48086880	-2.98506454	-2.41450982
C	0.11796529	-1.65719389	-0.71016257
C	-0.09887281	-2.82812088	0.22012715
C	0.67228210	-3.20823653	1.24233696
S	-0.62523149	-1.08505035	-4.61160977
O	-1.97030122	-1.62070450	-4.39388412
O	-0.44335445	0.21989158	-5.24858098
H	1.35222410	-0.80171540	-6.70858040
C	0.29769948	-2.29722830	-5.56769964
H	-0.67300669	-0.92134415	-0.49233070
C	0.11554213	-3.65963311	-5.29591185
O	3.53133656	-1.86017998	0.15987543
H	0.39096810	-4.05994828	1.85759236
H	-1.01268587	-3.39104679	0.01528383
C	1.84936975	-4.17345893	-6.91179165
H	1.59421912	-2.69778290	1.50461684

C	2.01018911	-2.81011180	-7.18504735
C	1.23598953	-1.86219695	-6.51040357
H	-0.63631547	-3.98196162	-4.58301087
H	0.76784751	-5.65589639	-5.76918257
C	0.90130967	-4.59647842	-5.97241666
H	2.45722187	-4.90683412	-7.43570621
H	2.73801932	-2.48229106	-7.92274028

8: References

[1] Á. L. Fuentes de Arriba, L. Simón, C. Raposo, V. Alcázar, J. R. Morán, *Tetrahedron*, **2009**, *65*, 4841–4845.

[2] Z. He, A. K. Yudin, *Angew. Chem. Int. Ed.* **2010**, *49*, 1607–1610; *Angew. Chem.* **2010**, *122*, 1651–1654.

[3] P. Wipf, M. Furegati, *Org. Lett.* **2006**, *8*, 1901–1904.

[4] a) G. te Velde, et al. *J. Comput. Chem.* **2001**, *22*, 931–967; b) C. Fonseca Guerra, J. G. Snijders, G. te Velde, E. J. Baerends, *Theor. Chem. Acc.* **1998**, *99*, 391 – 403; ADF2018.105, SCM Theoretical Chemistry, Vrije Universiteit: Amsterdam (The Netherlands), 2017. http://www.scm.com.

[5] a) J. C. Slater, Quantum Theory of Molecules and Solids. (McGraw-Hill, New York, 1974); b) A. D. Becke, *J. Chem. Phys.* **1986**, *84*, 4524–4529; c) A. D. Becke, *Phys. Rev. A* **1988**, *38*, 3098–3100.

[6] C. Lee, W. Yang, R. G. Parr, *Phys. Rev. B* **1988**, *37*, 785–789.

[7] E. van Lenthe, E. J. Baerends, *J. Comput. Chem.* **2003**, *24*, 1142–1156.

[8] a) G. T. de Jong, M. Solà, L. Visscher, F. M. Bickelhaupt, *J. Chem. Phys.* **2004**, *121*, 9982–9992; b) G. T. de Jong, F. M. Bickelhaupt, *J. Phys. Chem. A.* **2005**, *109*, 9685– 9699; c) G. T. de Jong, F. M. Bickelhaupt, *J. Chem. Theory Comput.* **2006**, *2*, 322– 335.

[9] a) A. Klamt, G. Schüürmann, *J. Chem. Soc. Perkin Trans. 2* **1993**, 799–805; b) A. Klamt, *J. Phys. Chem.* **1995**, *99*, 2224–2235; c) A. Klamt, V. Jonas, *J. Chem. Phys.* **1996**, *105*, 9972–9981; d) C. C. Pye, T. Ziegler, *Theor. Chem. Acc.* **1999**, *101*, 396– 408.

[10] a) S. Grimme, J. Antony, S. Ehrlich, S. Krieg, *J. Chem. Phys.* **2010**, *132*, 154104; b) A. D. Becke, E. R. Johnson, *J. Chem. Phys.* **2005**, *123*, 154101.

[11] Legault, C. Y. CYLview (Unversité de Sherbrooke: Sherbrooke, QC, Canada, 1.0b, 2009) http://www.cylview.org.

[12] Y. Zhao, D. G. Truhlar, *Theor. Chem. Acc.* **2008**, *120*, 215.

[13] a) P. Vermeeren, S. C. C. van der Lubbe, C. Fonseca Guerra, F. M. Bickelhaupt, T. A. Hamlin, *Nature Protoc.* **2020**, *15*, 649; b) F. M. Bickelhaupt, K. N. Houk, *Angew. Chem. Int. Ed.* **2017**, *56*, 10070–10086; *Angew. Chem.* **2017**, *129*, 10204–10221; c) L. P. Wolters, F. M. Bickelhaupt, WIRES Comput. Mol. Sci. 2015, 5, 324–343; d) I. Fernández, F. M. Bickelhaupt, Chem. Soc. Rev. 2014, 43, 4953–4967; e) W.-J. van Zeist, F. M. Bickelhaupt, Org. Biomol. Chem. 2010, 8, 3118–3127.

9: NMR Spectra

9.1 Spectra of prolineamide catalysts

P1

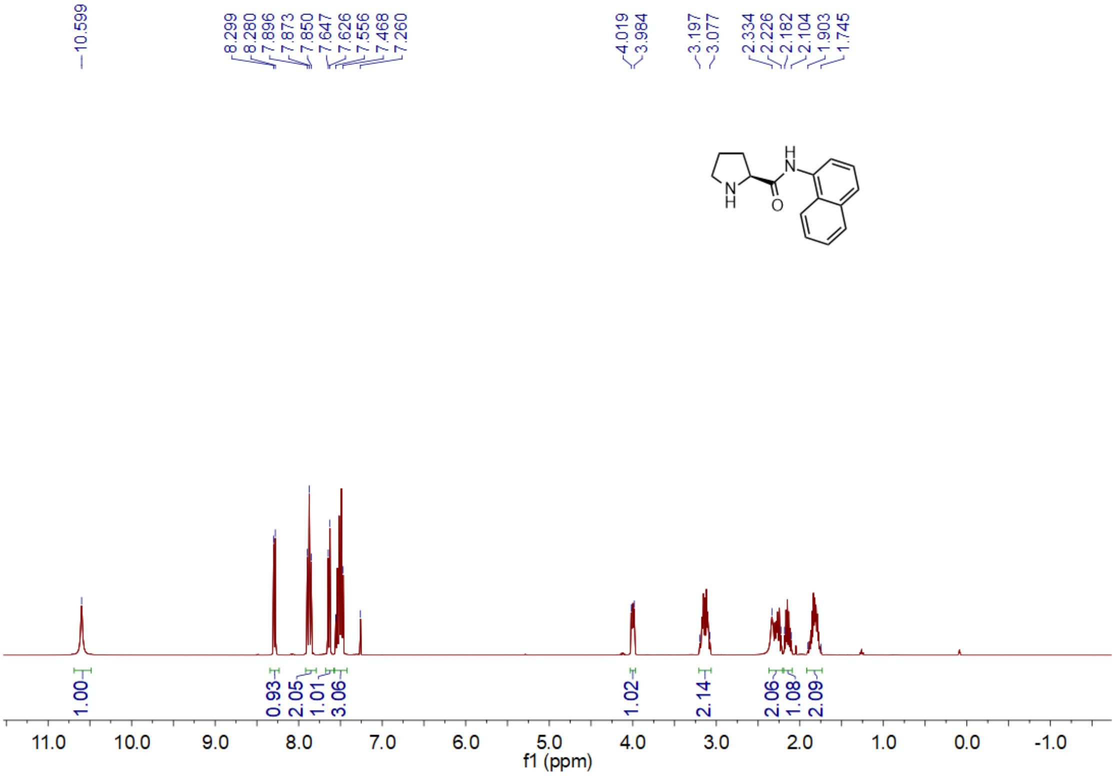

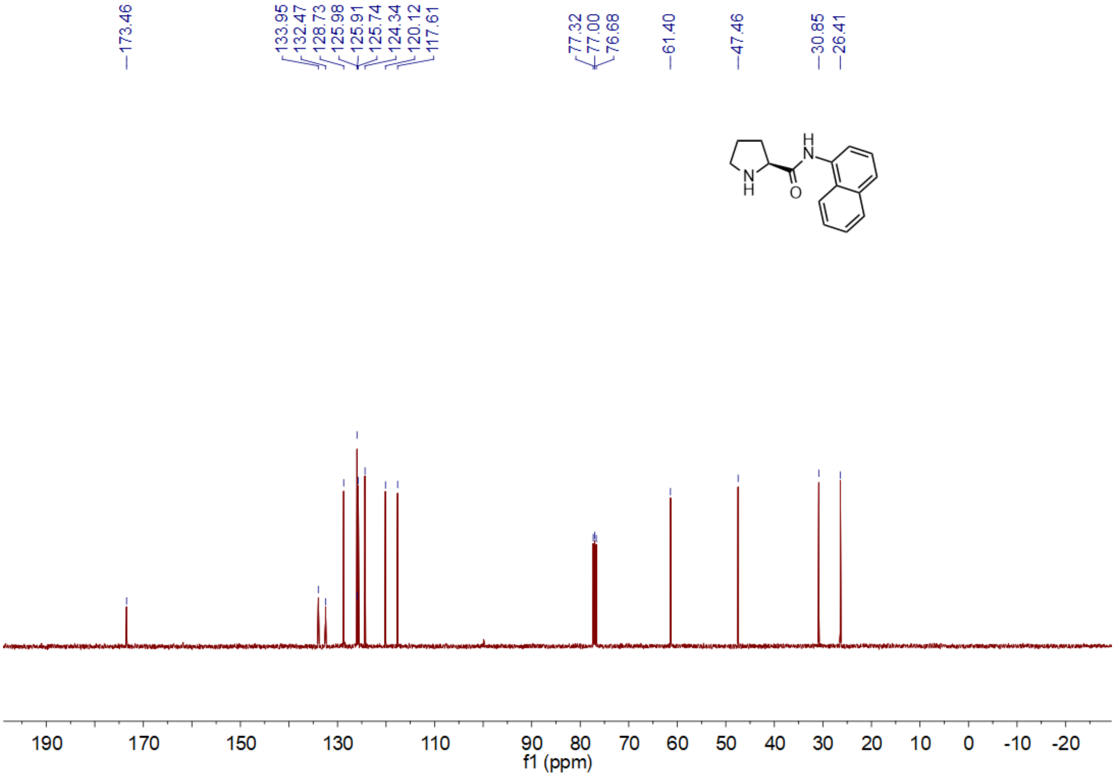

P3

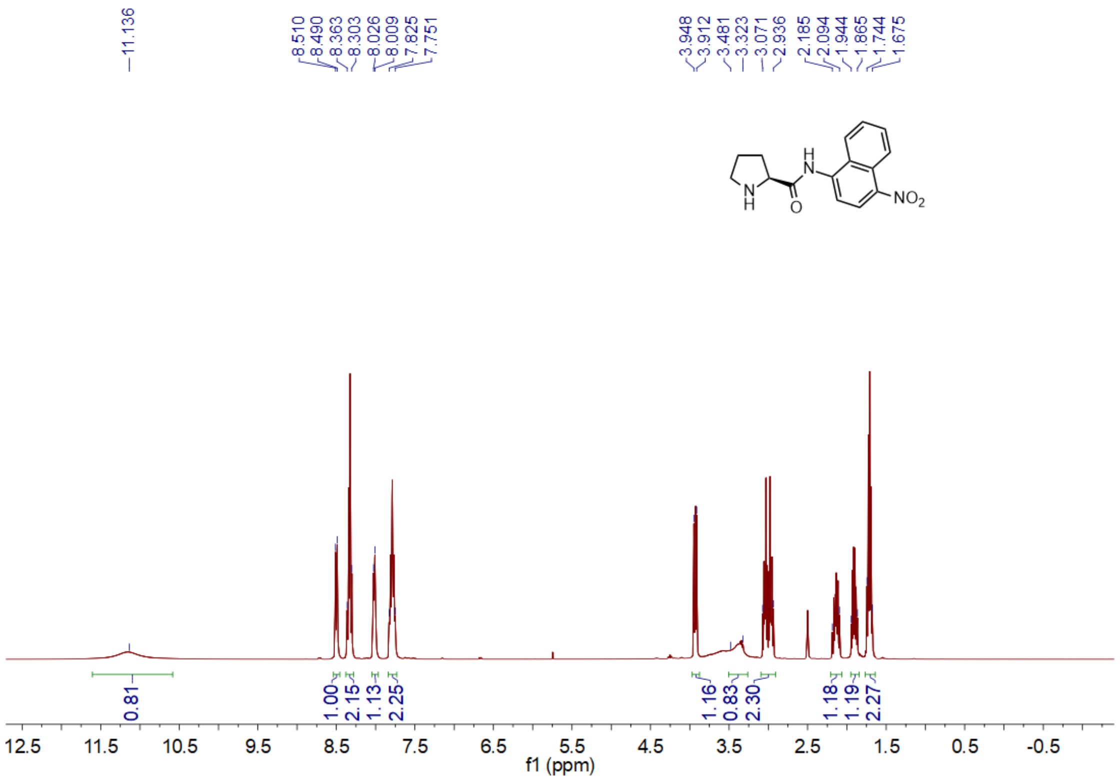

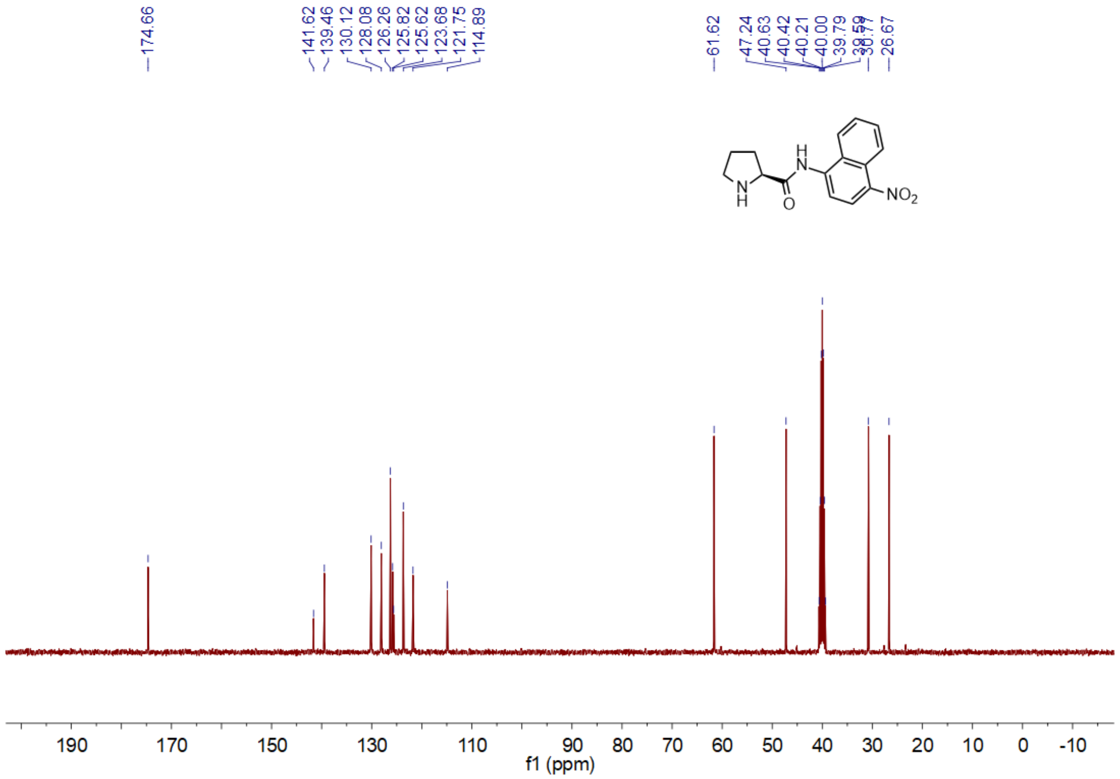

P4

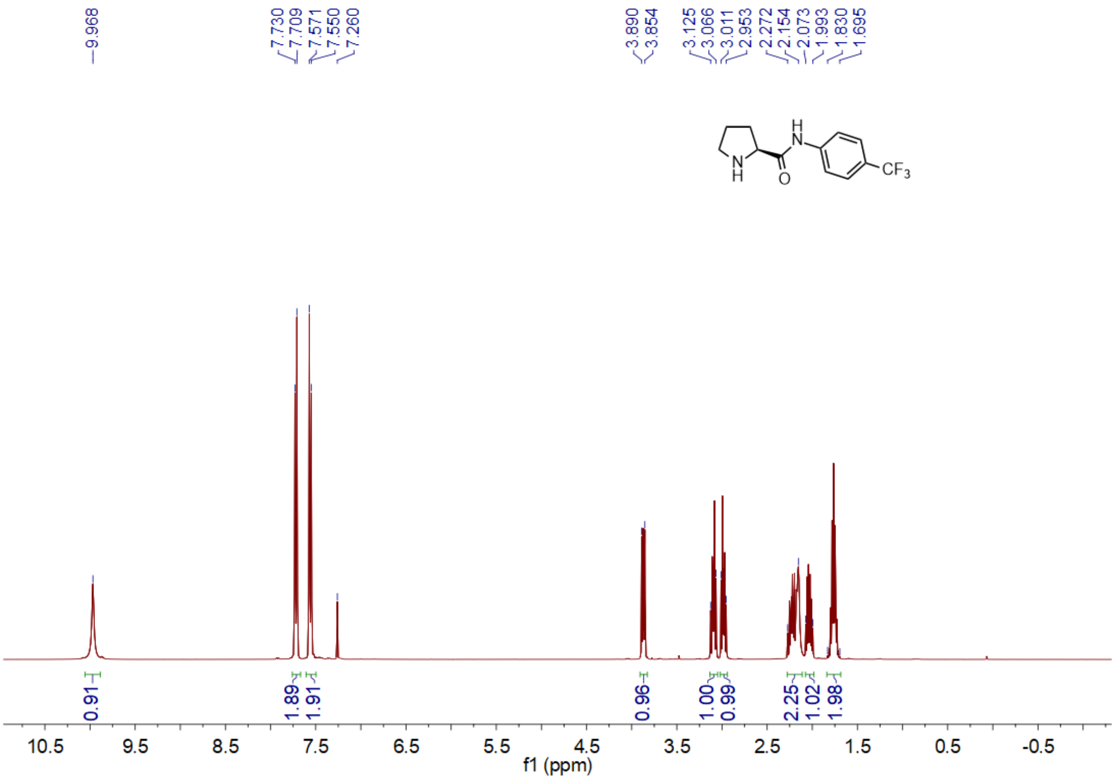
9.968
7.730
7.709
7.571
7.550
7.260
3.890
3.854
3.125
3.066
3.011
2.953
2.272
2.154
2.073
1.993
1.830
1.695
H
N
N
H
O
CF3
0.91
1.89
1.91
0.96
1.00
0.99
2.25
1.02
1.98
10.5
9.5
8.5
7.5
6.5
5.5
4.5
3.5
2.5
1.5
0.5
-0.5
f1 (ppm)

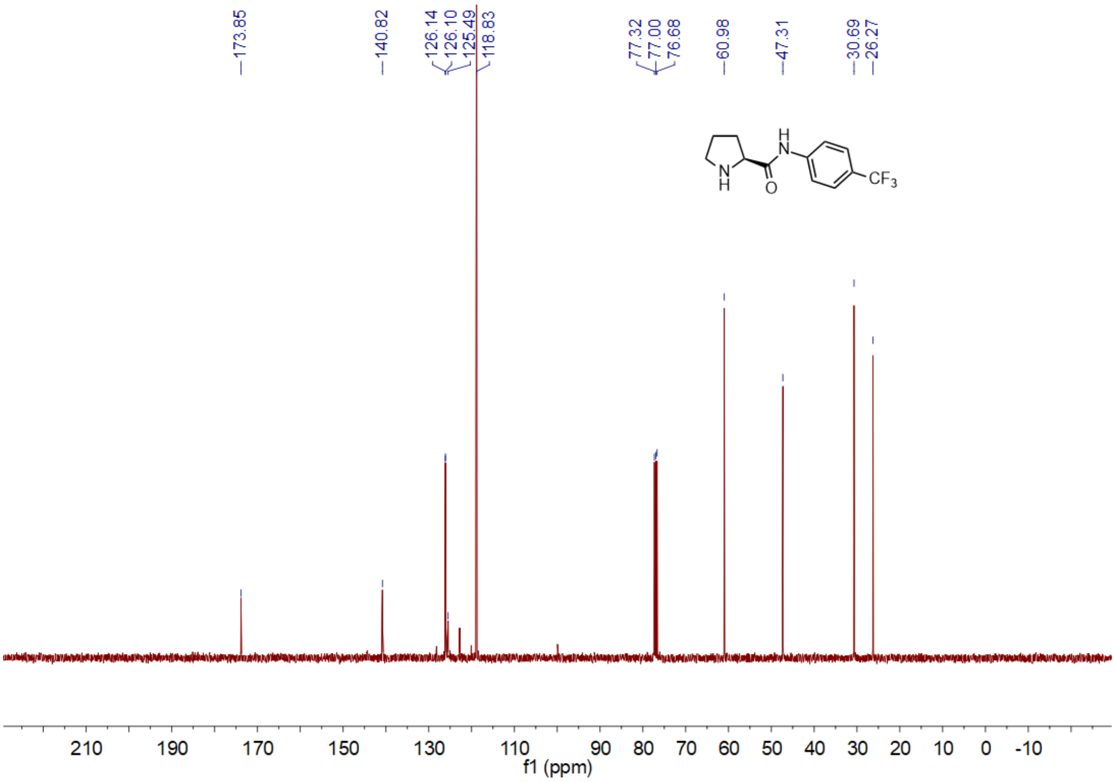
173.85
140.82
126.14
126.10
125.49
118.83
77.32
77.00
76.68
60.98
47.31
30.69
26.27
H
N
N
H
O
CF3
210
190
170
150
130
110
90
80
70
60
50
40
30
20
10
0
-10
f1 (ppm)

P5

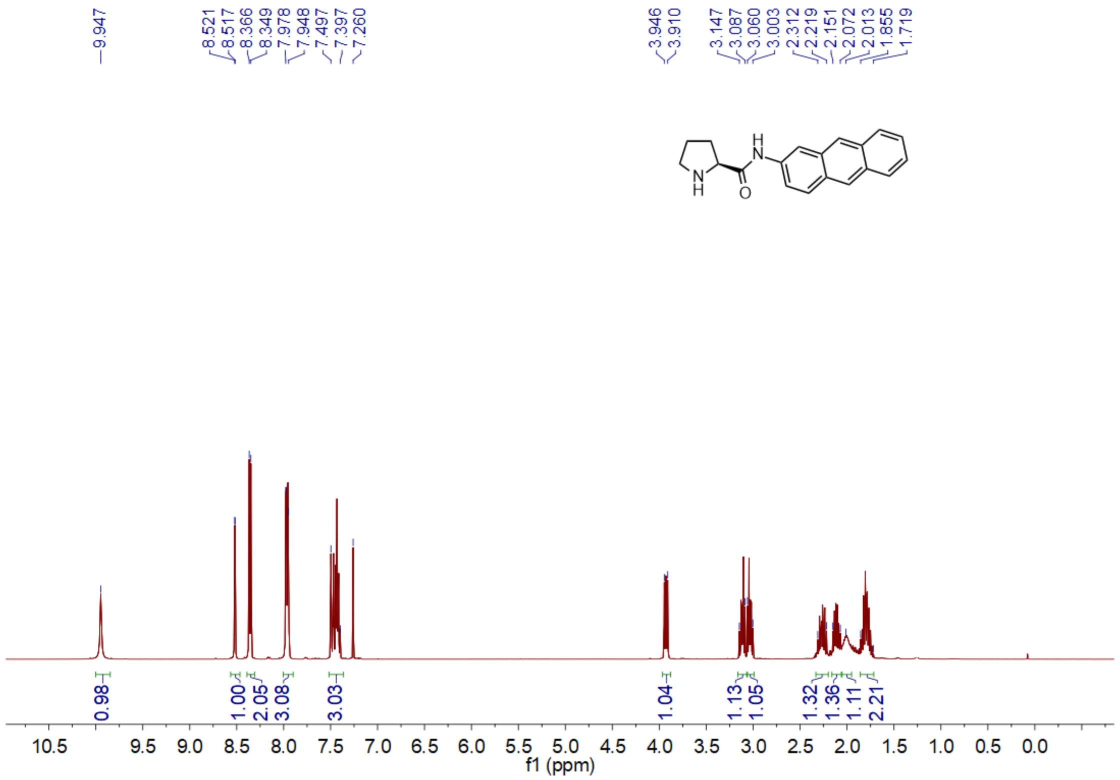

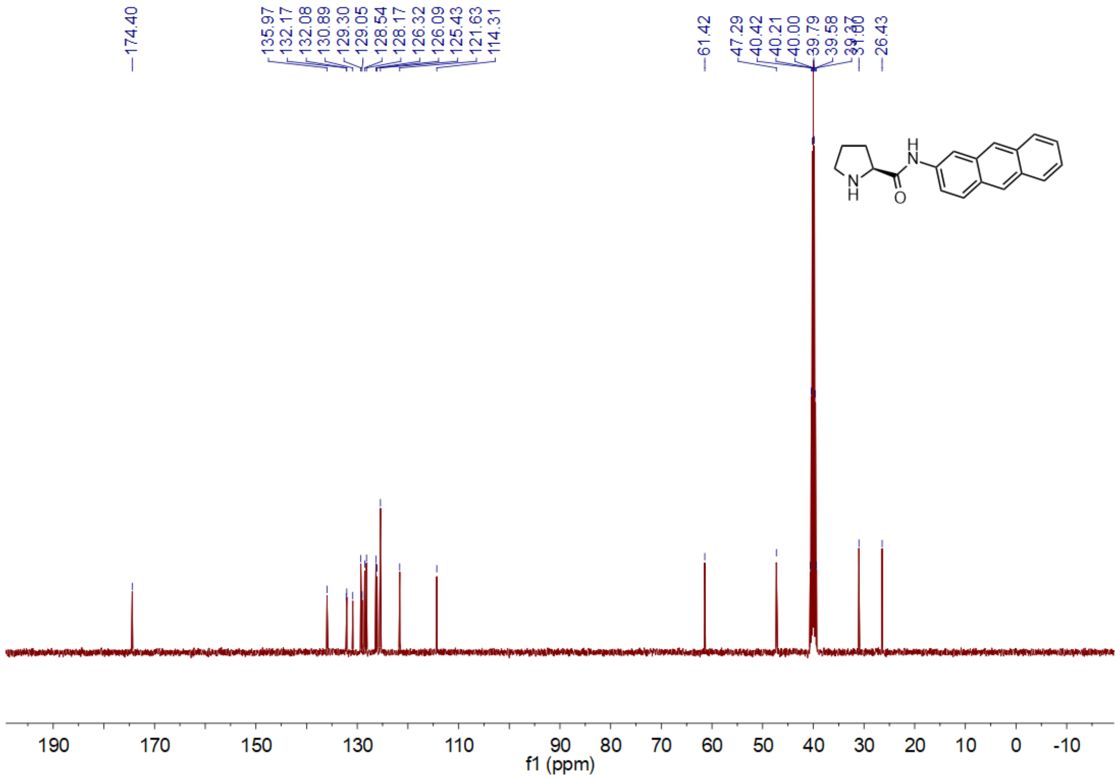

P6

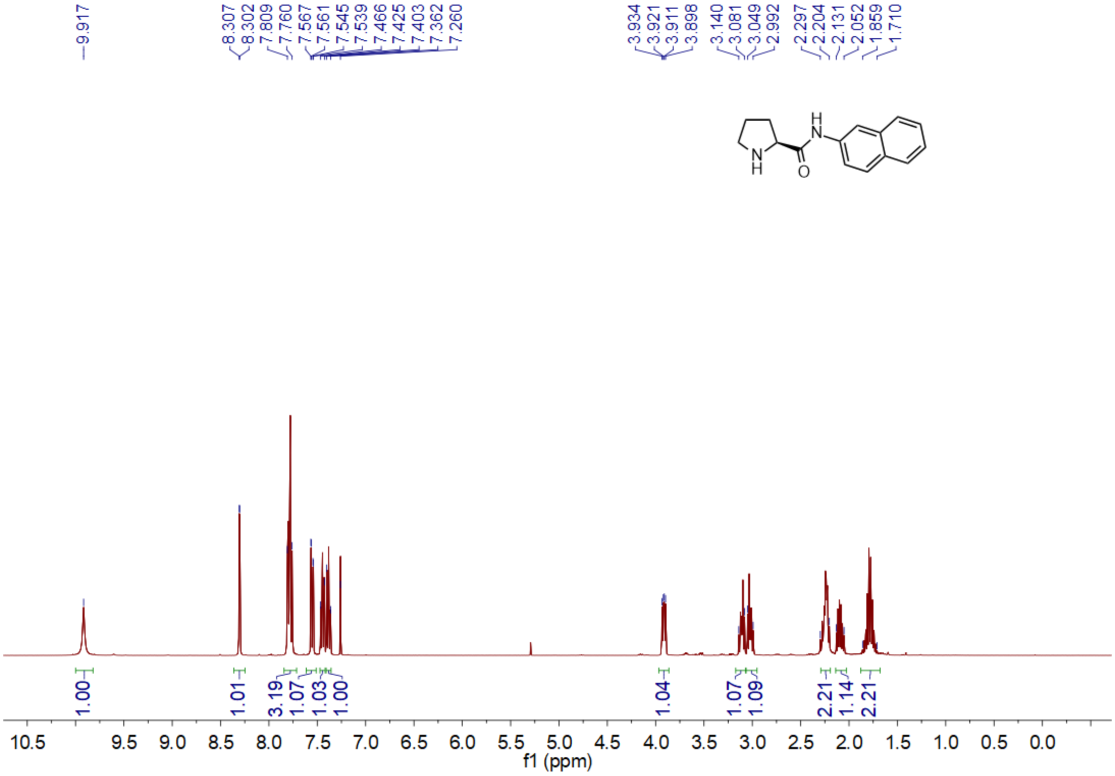
9.917
8.307
8.302
7.809
7.760
7.567
7.561
7.545
7.539
7.466
7.425
7.403
7.362
7.260
3.934
3.921
3.911
3.898
3.140
3.081
3.049
2.992
2.297
2.204
2.131
2.052
1.859
1.710
H
N
N
H
O
1.00
1.01
3.19
1.07
1.03
1.00
1.04
1.07
1.09
2.21
1.14
2.21
10.5 9.5 9.0 8.5 8.0 7.5 7.0 6.5 6.0 5.5 5.0 4.5 4.0 3.5 3.0 2.5 2.0 1.5 1.0 0.5 0.0
f1 (ppm)

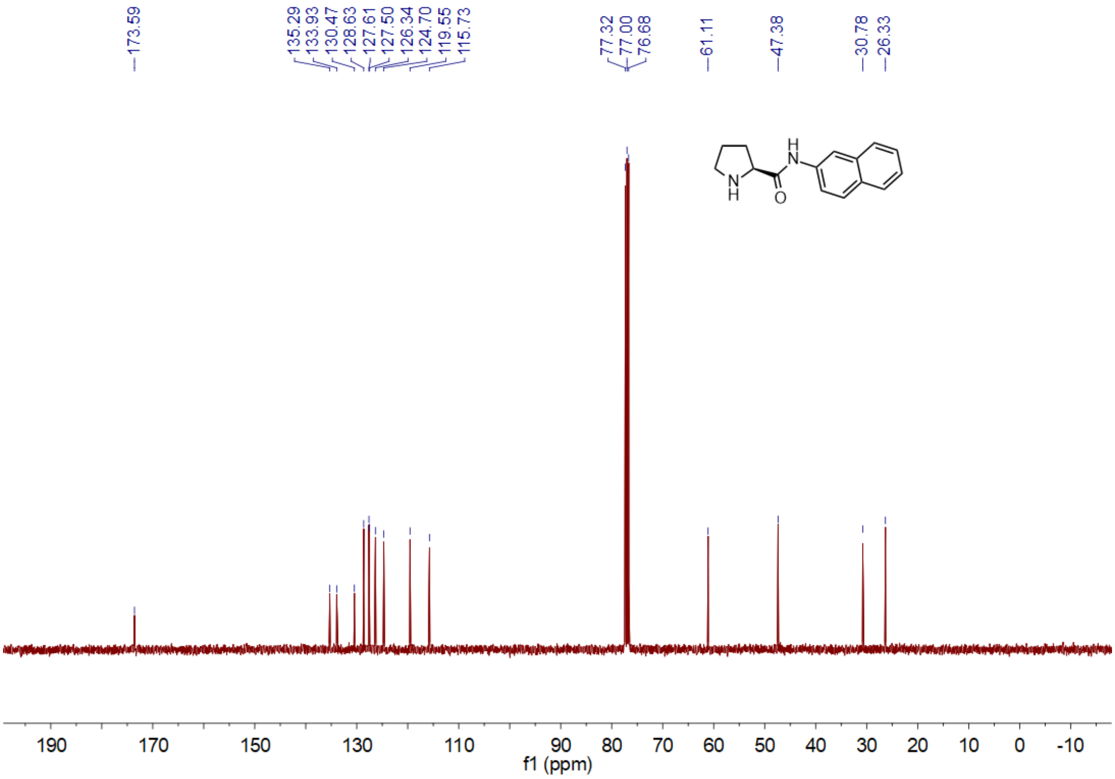
173.59
135.29
133.93
130.47
128.63
127.61
127.50
126.34
124.70
119.55
115.73
77.32
77.00
76.68
61.11
47.38
30.78
26.33
H
N
N
H
O
190 170 150 130 110 90 80 70 60 50 40 30 20 10 0 -10
f1 (ppm)

9.2 Spectra of starting materials

1a

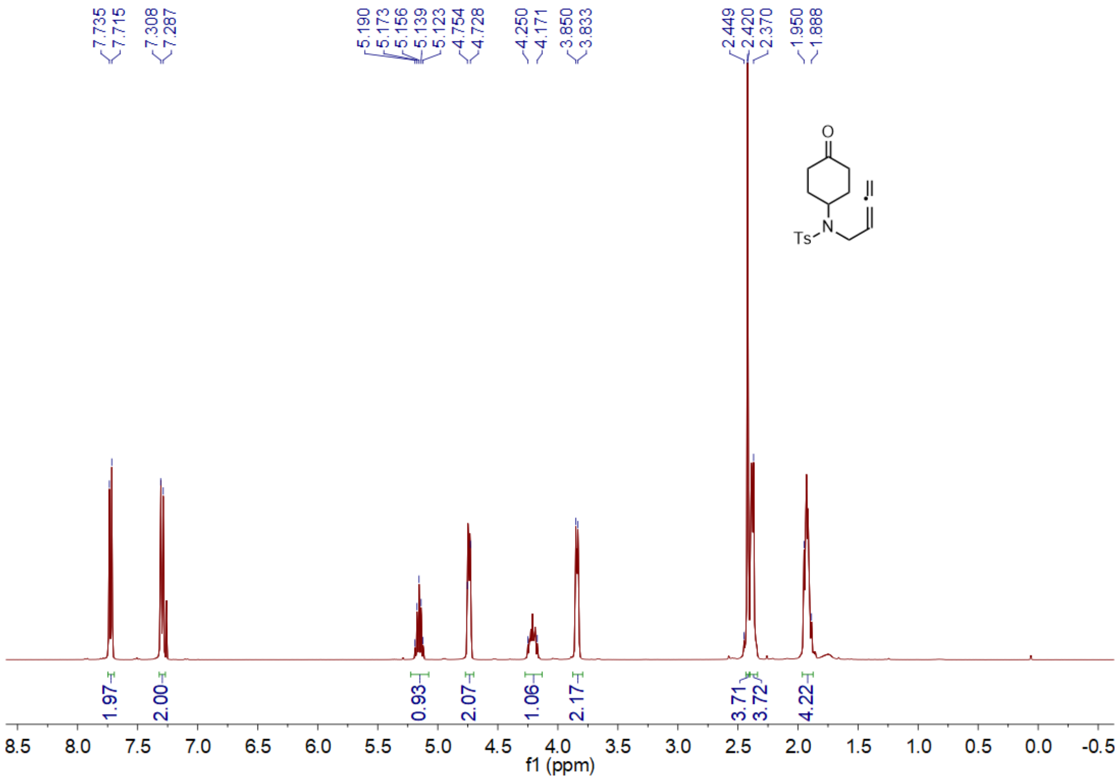

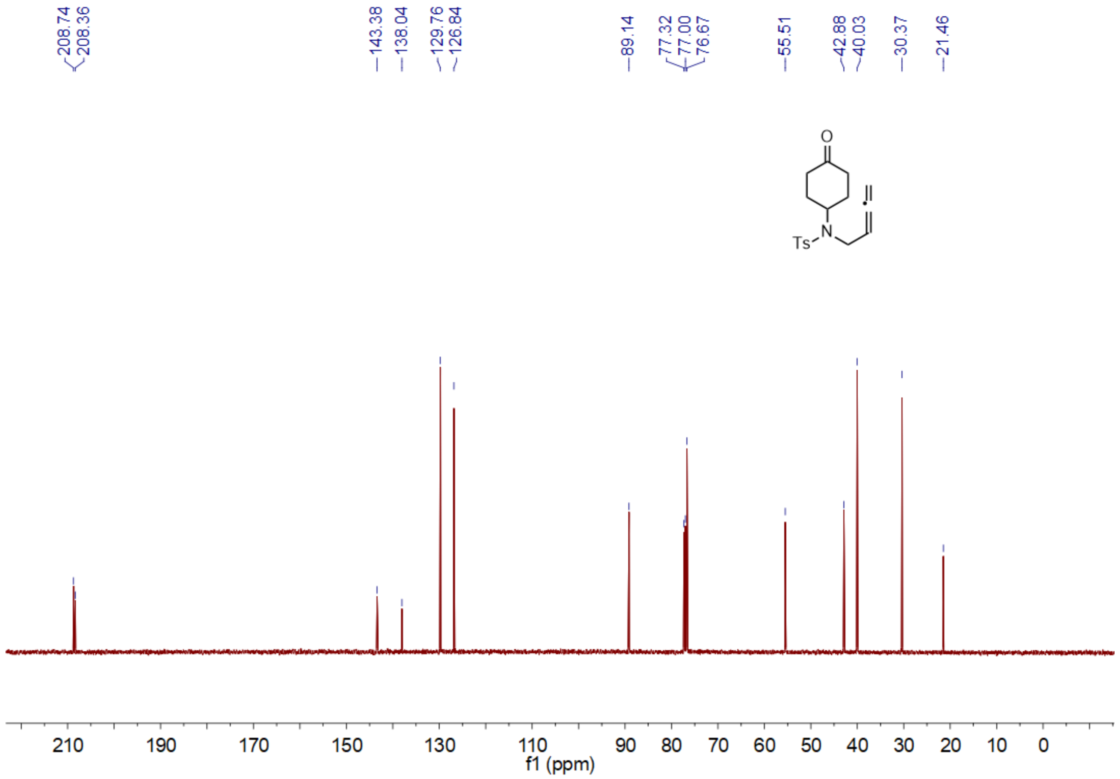

1b

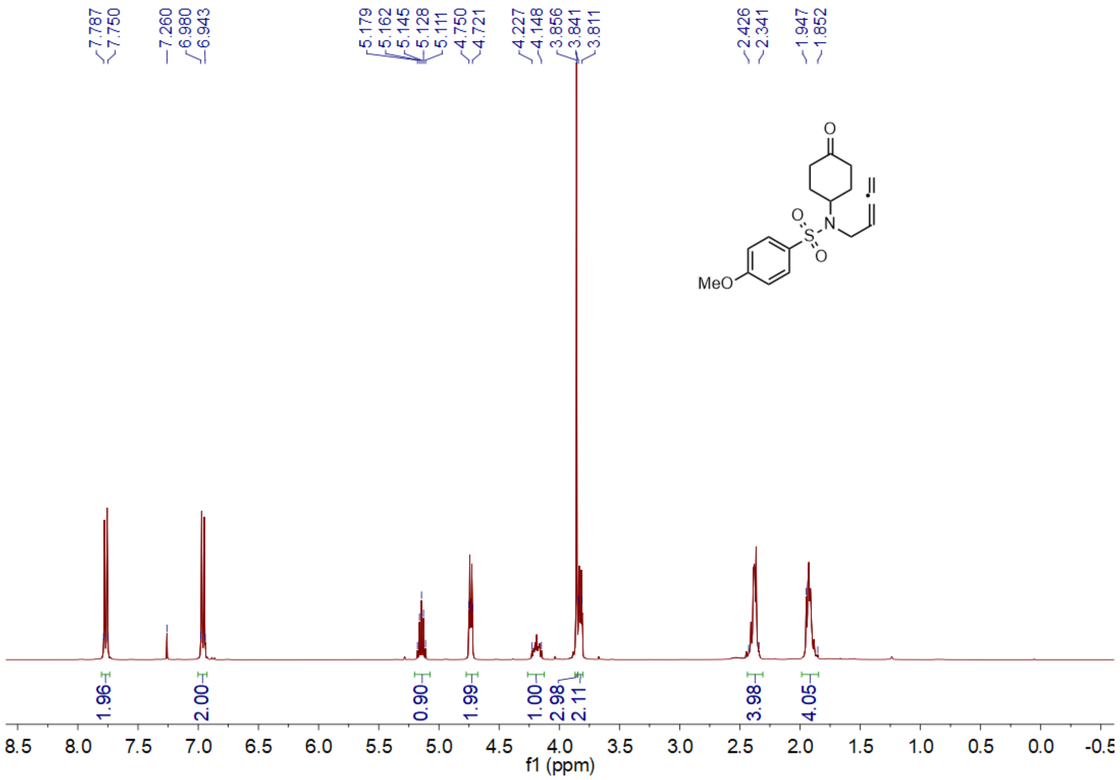
7.787
7.750
7.260
6.980
6.943
5.179
5.162
5.145
5.128
5.111
4.750
4.721
4.227
4.148
3.856
3.841
3.811
2.426
2.341
1.947
1.852
MeO
O
S
O
O
N
1.96
2.00
0.90
1.99
1.00
2.98
2.11
3.98
4.05
8.5
8.0
7.5
7.0
6.5
6.0
5.5
5.0
4.5
4.0
3.5
3.0
2.5
2.0
1.5
1.0
0.5
0.0
-0.5
f1 (ppm)

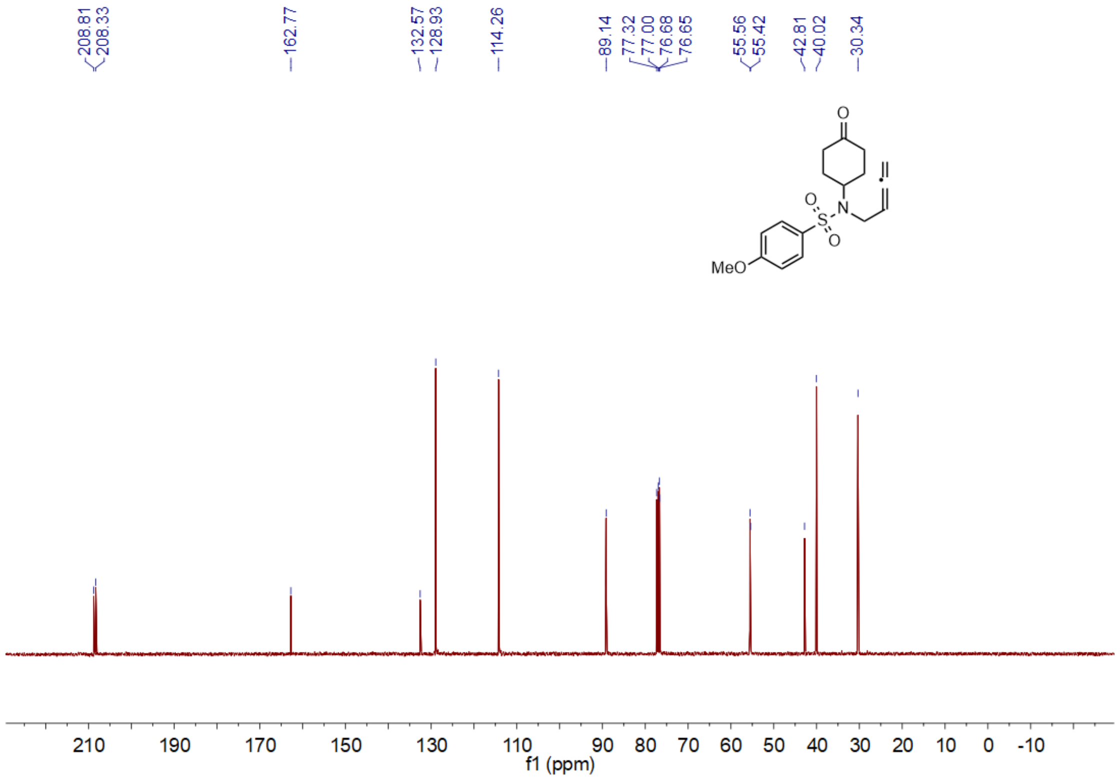
208.81
208.33
162.77
132.57
128.93
114.26
89.14
77.32
77.00
76.68
76.65
55.56
55.42
42.81
40.02
30.34
MeO
O
S
O
O
N
210
190
170
150
130
110
90
80
70
60
50
40
30
20
10
0
-10
f1 (ppm)

1c

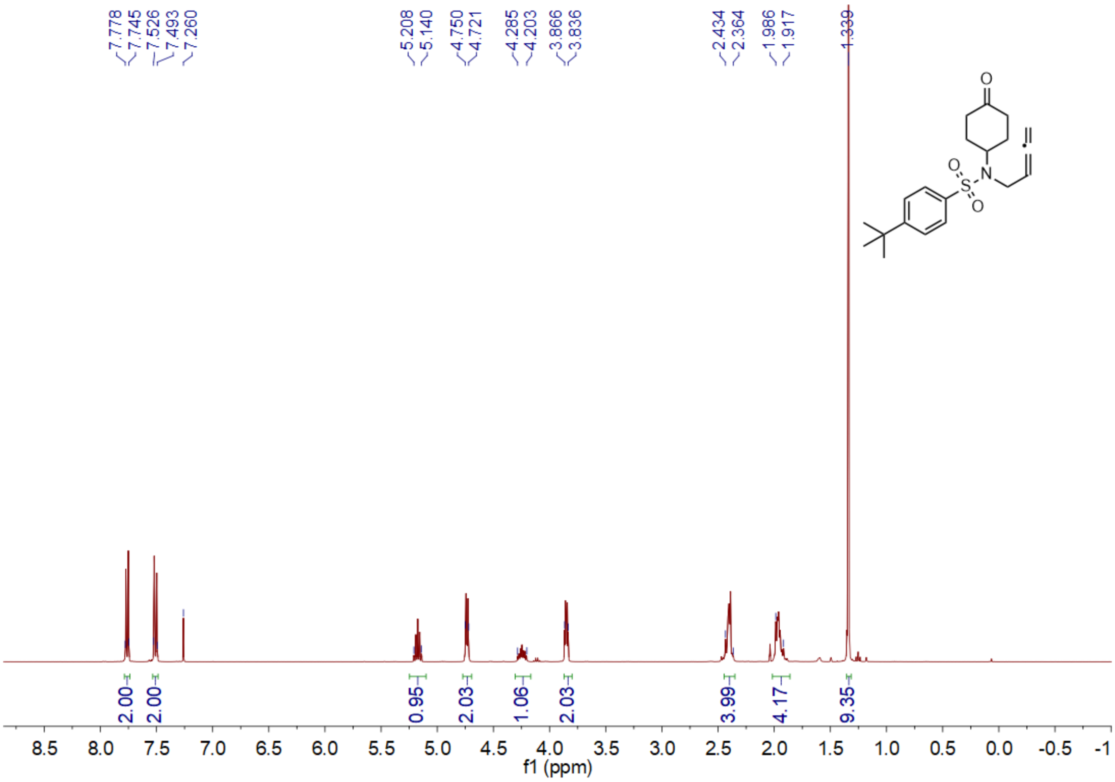

7.778
7.745
7.526
7.493
7.260
5.208
5.140
4.750
4.721
4.285
4.203
3.866
3.836
2.434
2.364
1.986
1.917
1.339
2.00
2.00
0.95
2.03
1.06
2.03
3.99
4.17
9.35
8.5
8.0
7.5
7.0
6.5
6.0
5.5
5.0
4.5
4.0
3.5
3.0
2.5
2.0
1.5
1.0
0.5
0.0
-0.5
-1
f1 (ppm)
O
S
O
N
O

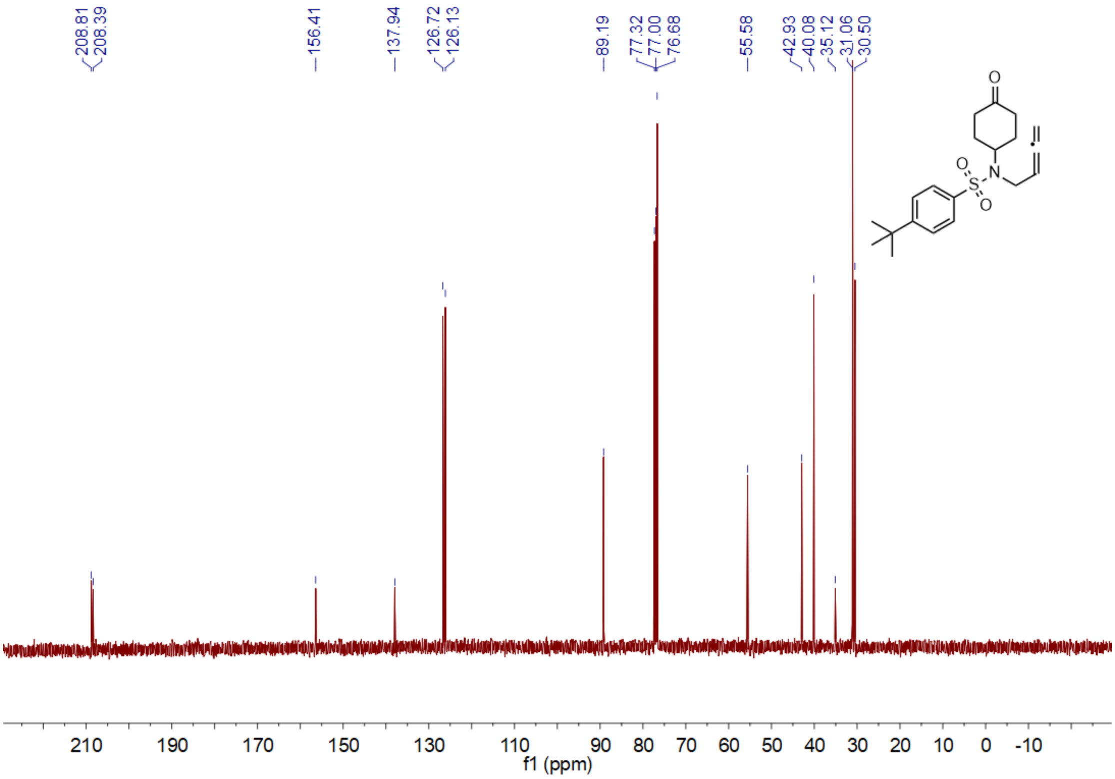

208.81
208.39
156.41
137.94
126.72
126.13
89.19
77.32
77.00
76.68
55.58
42.93
40.08
35.12
31.06
30.50
210
190
170
150
130
110
90
80
70
60
50
40
30
20
10
0
-10
f1 (ppm)
O
S
O
N
O

1d

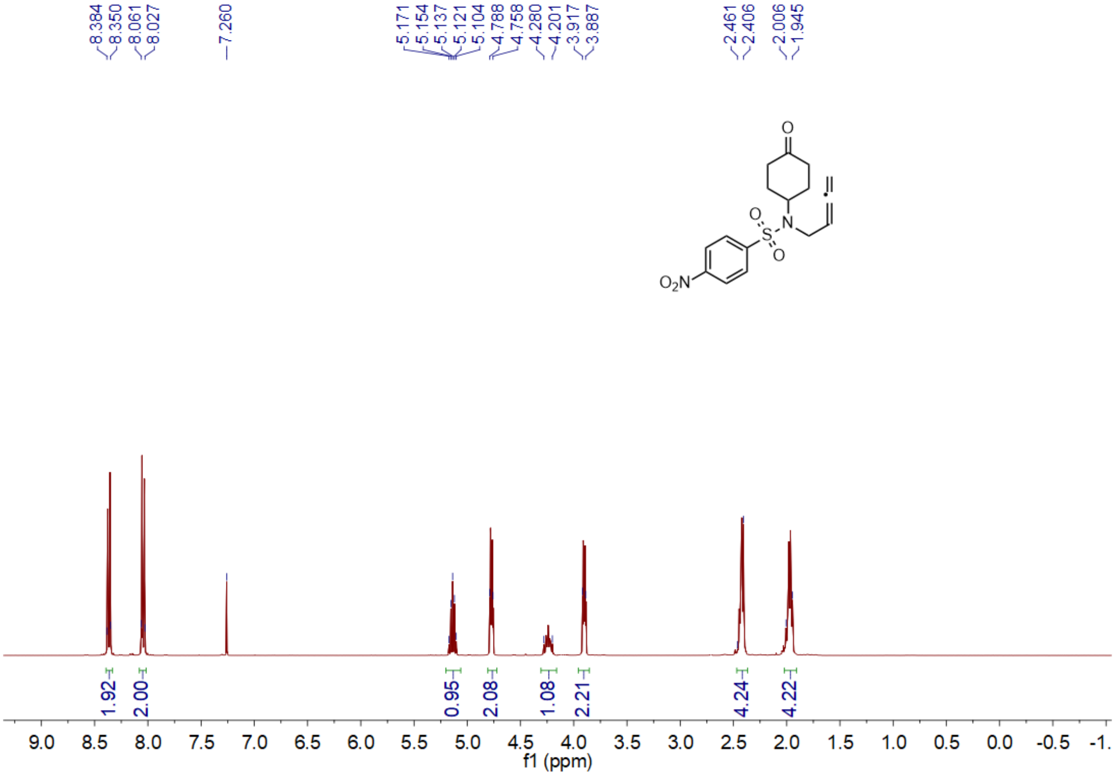
8.384
8.350
8.061
8.027
7.260
5.171
5.154
5.137
5.121
5.104
4.788
4.758
4.280
4.201
3.917
3.887
2.461
2.406
2.006
1.945
O
O
S
O
N
O_2N
1.92
2.00
0.95
2.08
1.08
2.21
4.24
4.22
9.0 8.5 8.0 7.5 7.0 6.5 6.0 5.5 5.0 4.5 4.0 3.5 3.0 2.5 2.0 1.5 1.0 0.5 0.0 -0.5 -1.
f1 (ppm)

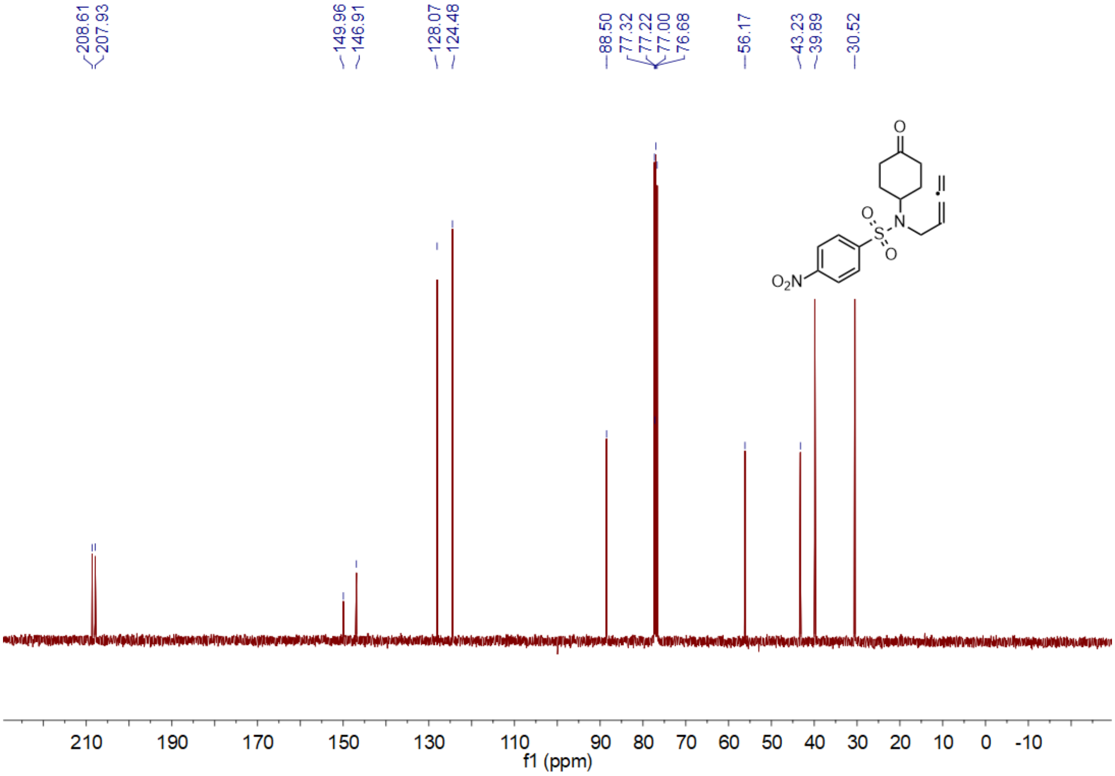
208.61
207.93
149.96
146.91
128.07
124.48
88.50
77.32
77.22
77.00
76.68
56.17
43.23
39.89
30.52
O
O
S
O
N
O_2N
210 190 170 150 130 110 90 80 70 60 50 40 30 20 10 0 -10
f1 (ppm)

1e

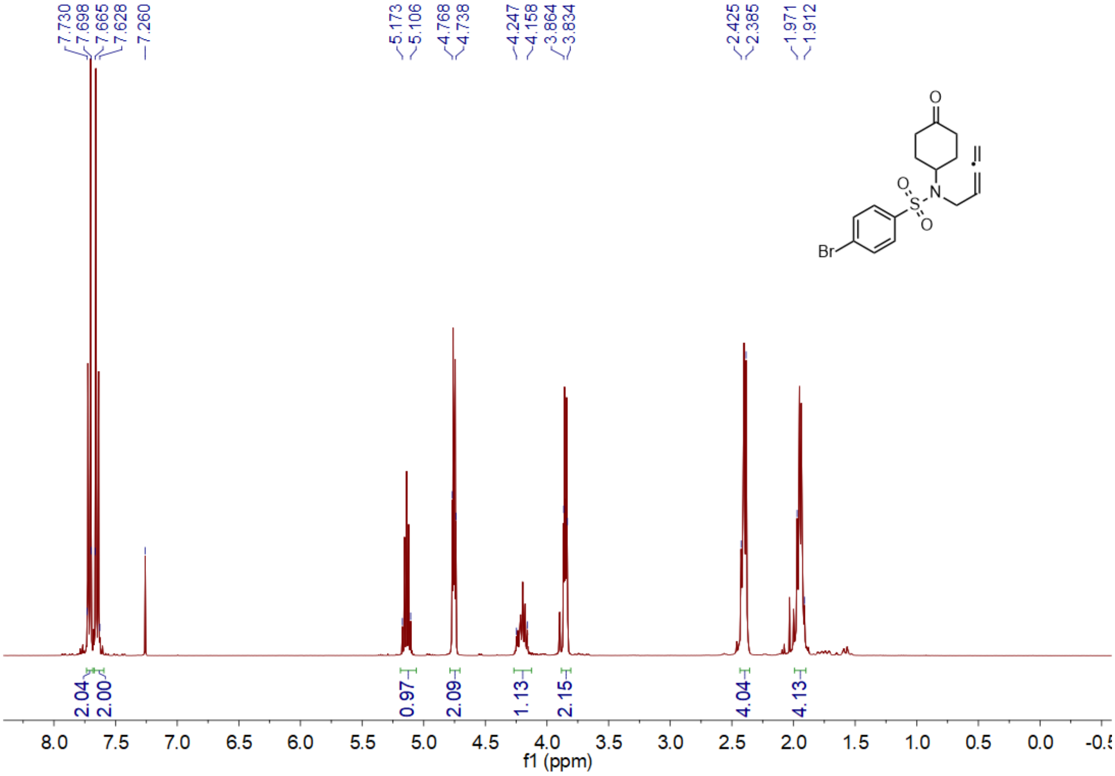

7.730
7.698
7.665
7.628
7.260
5.173
5.106
4.768
4.738
4.247
4.158
3.864
3.834
2.425
2.385
1.971
1.912
2.04
2.00
0.97
2.09
1.13
2.15
4.04
4.13
f1 (ppm)
Br
O
S
N

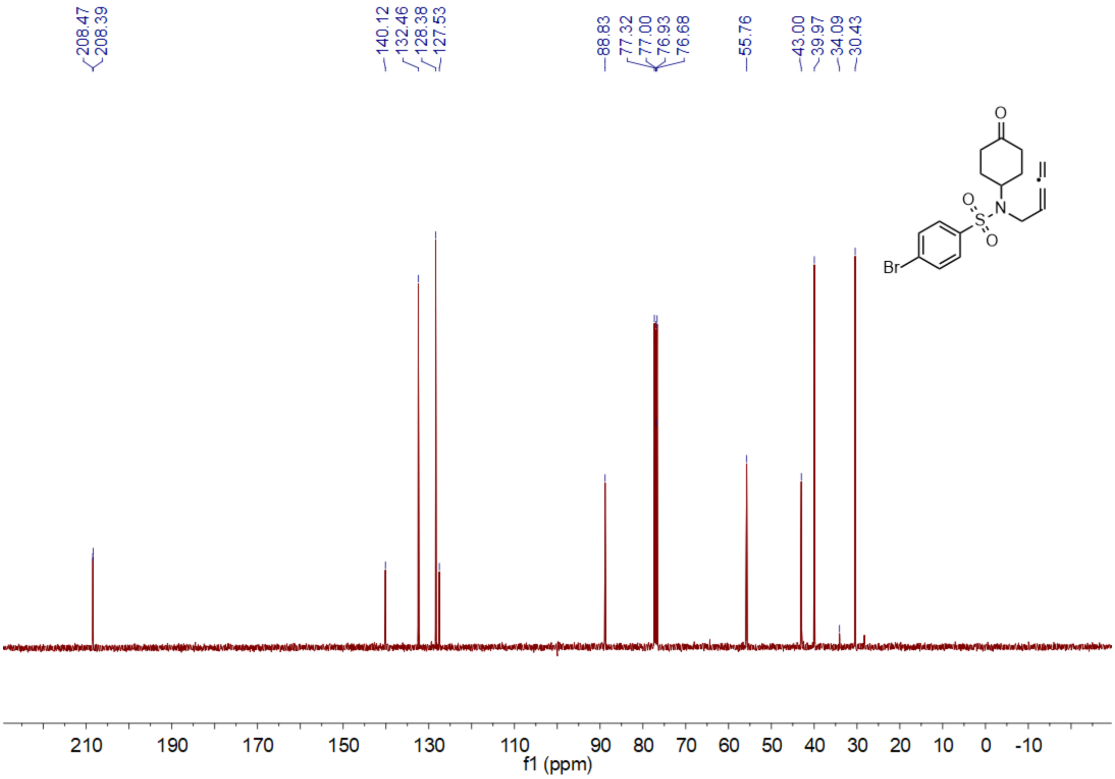

208.47
208.39
140.12
132.46
128.38
127.53
88.83
77.32
77.00
76.93
76.68
55.76
43.00
39.97
34.09
30.43
f1 (ppm)
Br
O
S
N

1f

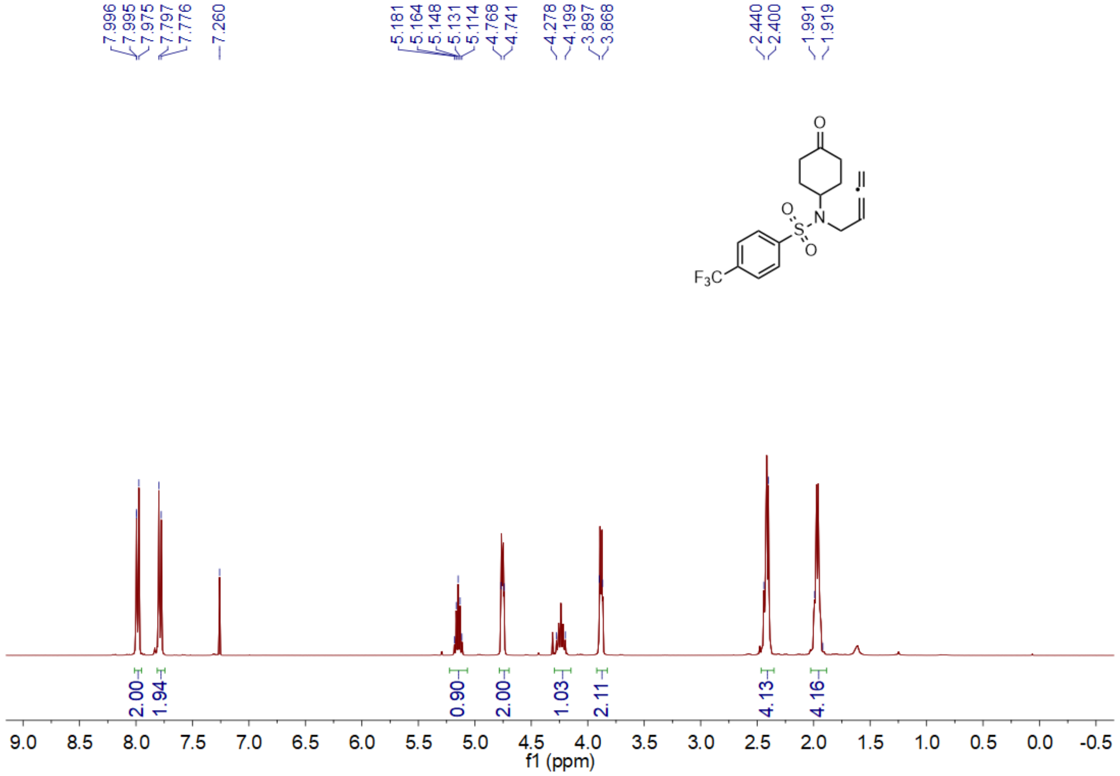
7.996
7.995
7.975
7.797
7.776
7.260
5.181
5.164
5.148
5.131
5.114
4.768
4.741
4.278
4.199
3.897
3.868
2.440
2.400
1.991
1.919
2.00
1.94
0.90
2.00
1.03
2.11
4.13
4.16
f1 (ppm)

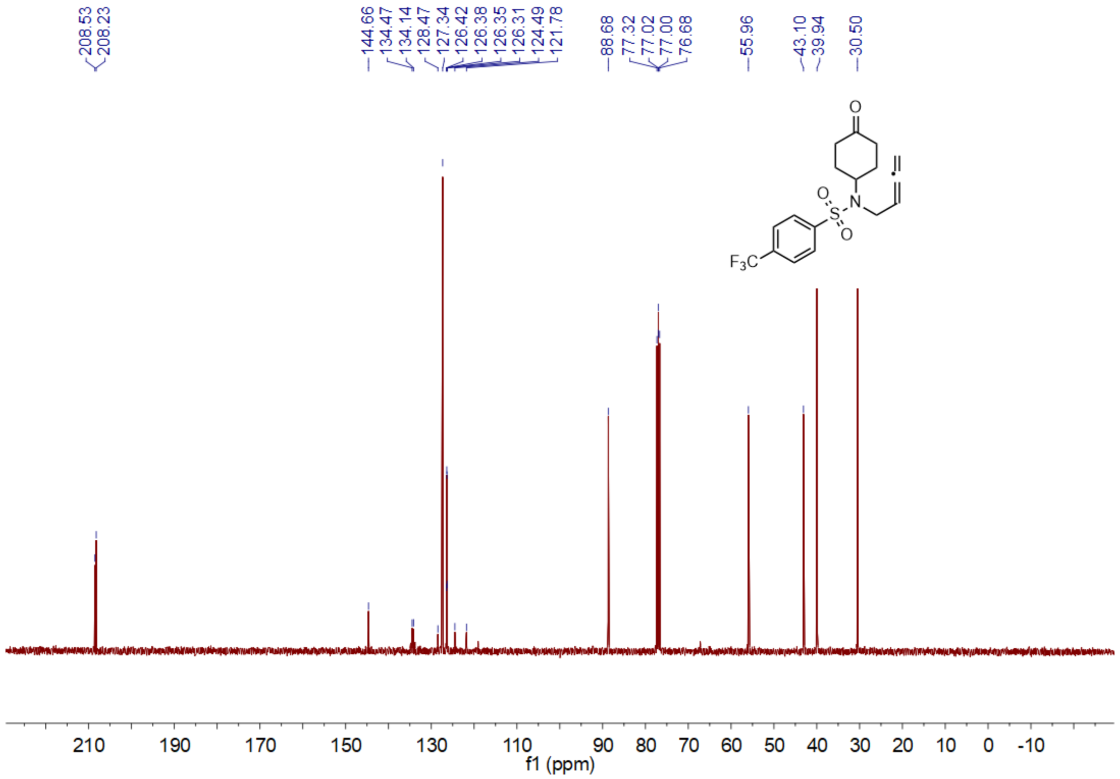
208.53
208.23
144.66
134.47
134.14
128.47
127.34
126.42
126.38
126.35
126.31
124.49
121.78
88.68
77.32
77.02
77.00
76.68
55.96
43.10
39.94
30.50
f1 (ppm)

1g

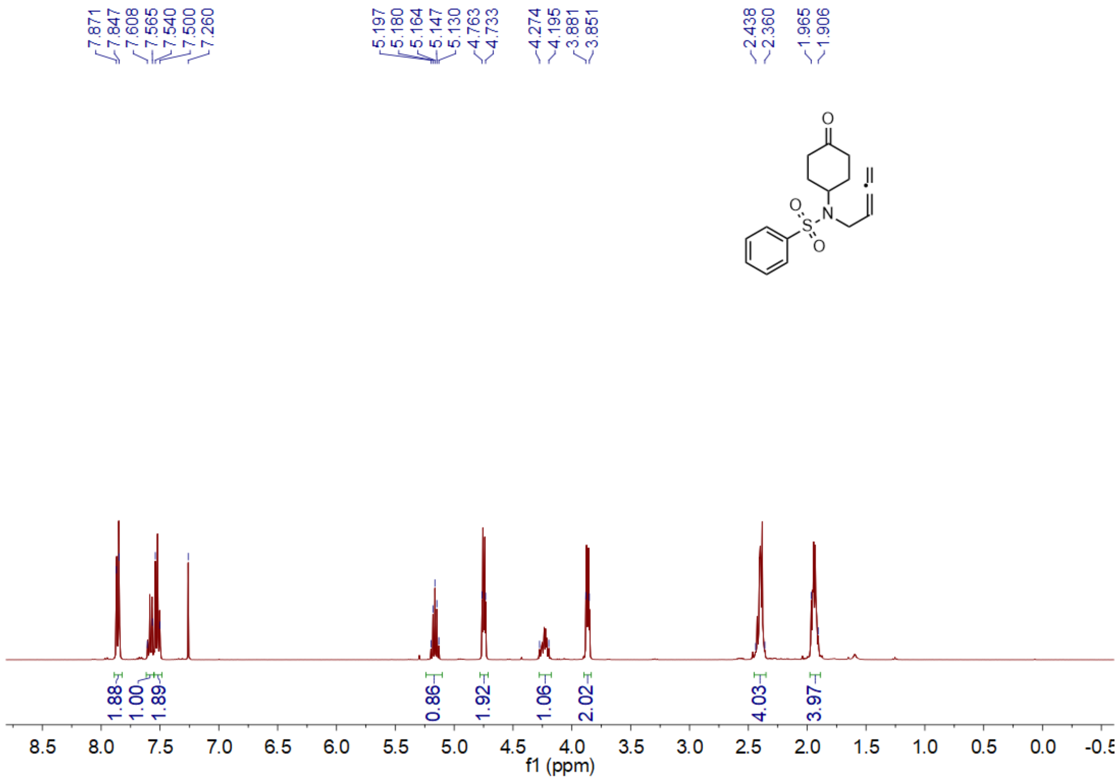

7.871
7.847
7.608
7.565
7.540
7.500
7.260
5.197
5.180
5.164
5.147
5.130
4.763
4.733
4.274
4.195
3.881
3.851
2.438
2.360
1.965
1.906
1.88
1.00
1.89
0.86
1.92
1.06
2.02
4.03
3.97
8.5 8.0 7.5 7.0 6.5 6.0 5.5 5.0 4.5 4.0 3.5 3.0 2.5 2.0 1.5 1.0 0.5 0.0 -0.5
f1 (ppm)

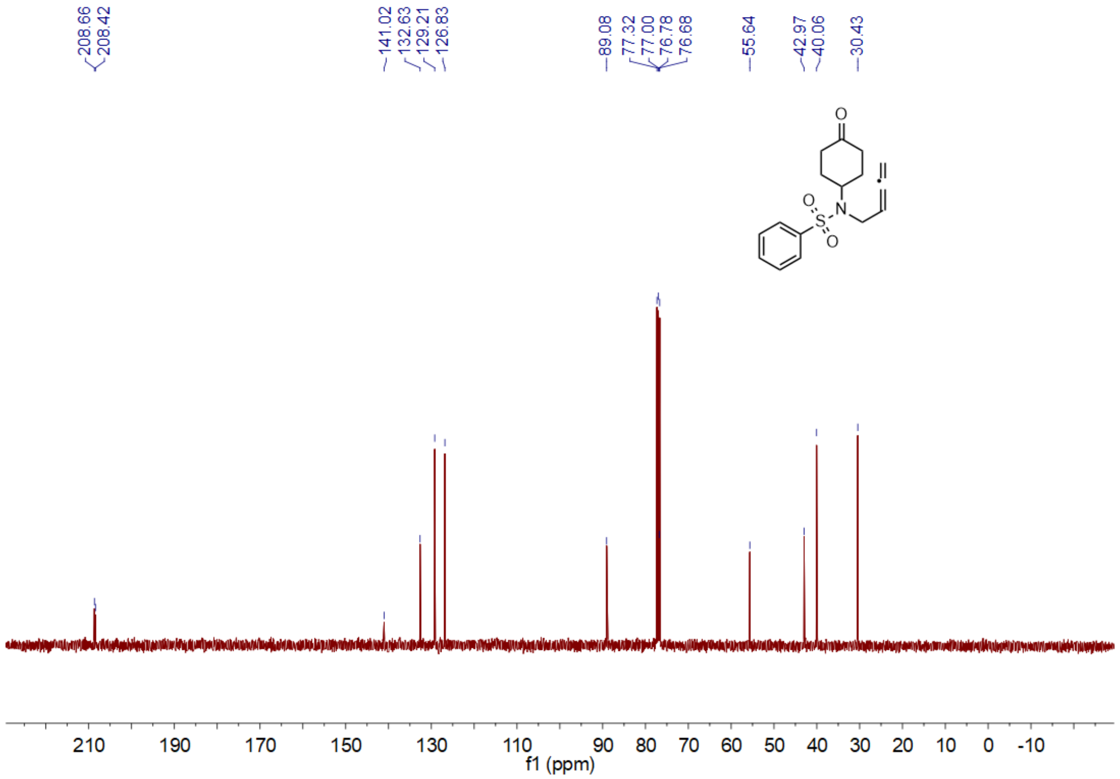

208.66
208.42
141.02
132.63
129.21
126.83
89.08
77.32
77.00
76.78
76.68
55.64
42.97
40.06
30.43
210 190 170 150 130 110 90 80 70 60 50 40 30 20 10 0 -10
f1 (ppm)

1h

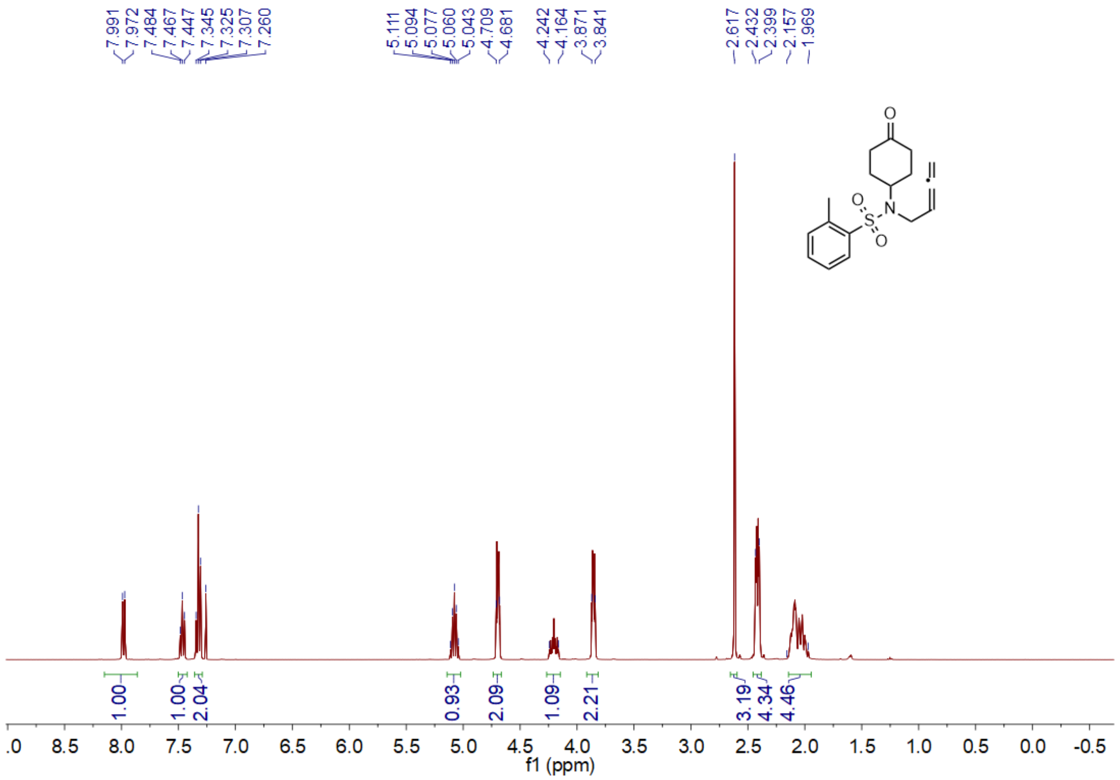
7.991
7.972
7.484
7.467
7.447
7.345
7.325
7.307
7.260
5.111
5.094
5.077
5.060
5.043
4.709
4.681
4.242
4.164
3.871
3.841
2.617
2.432
2.399
2.157
1.969
1.00
1.00
2.04
0.93
2.09
1.09
2.21
3.19
4.34
4.46
f1 (ppm)

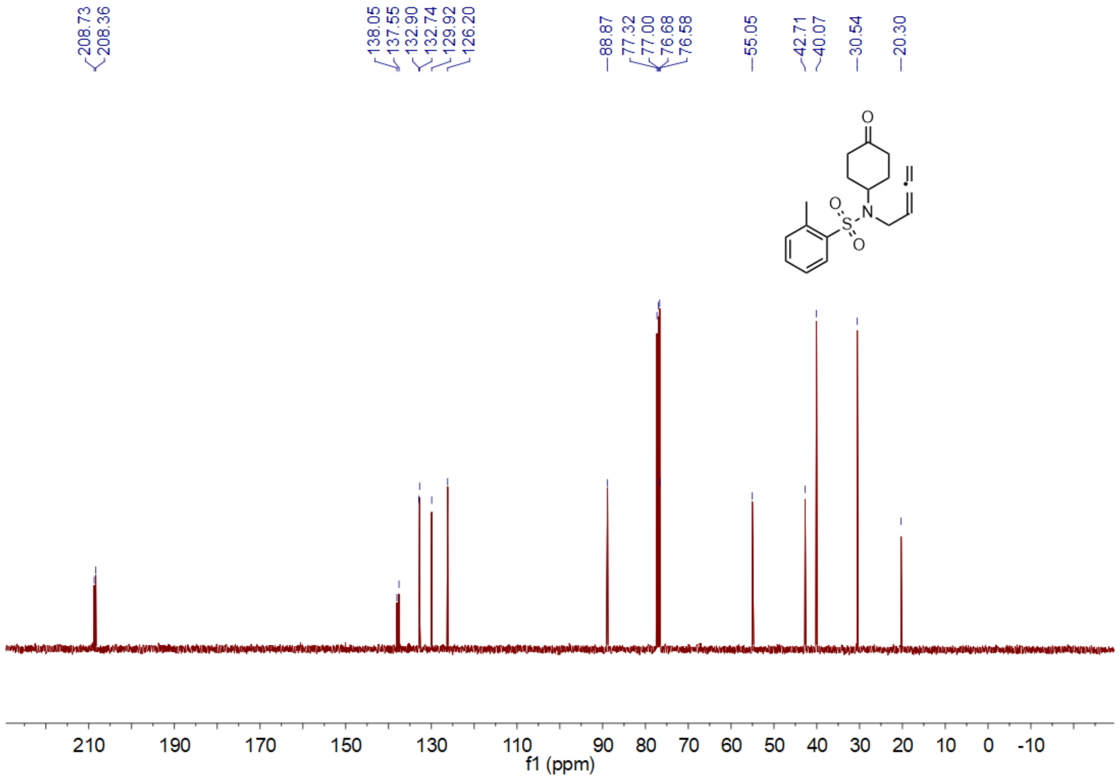
208.73
208.36
138.05
137.55
132.90
132.74
129.92
126.20
88.87
77.32
77.00
76.68
76.58
55.05
42.71
40.07
30.54
20.30
f1 (ppm)

1i

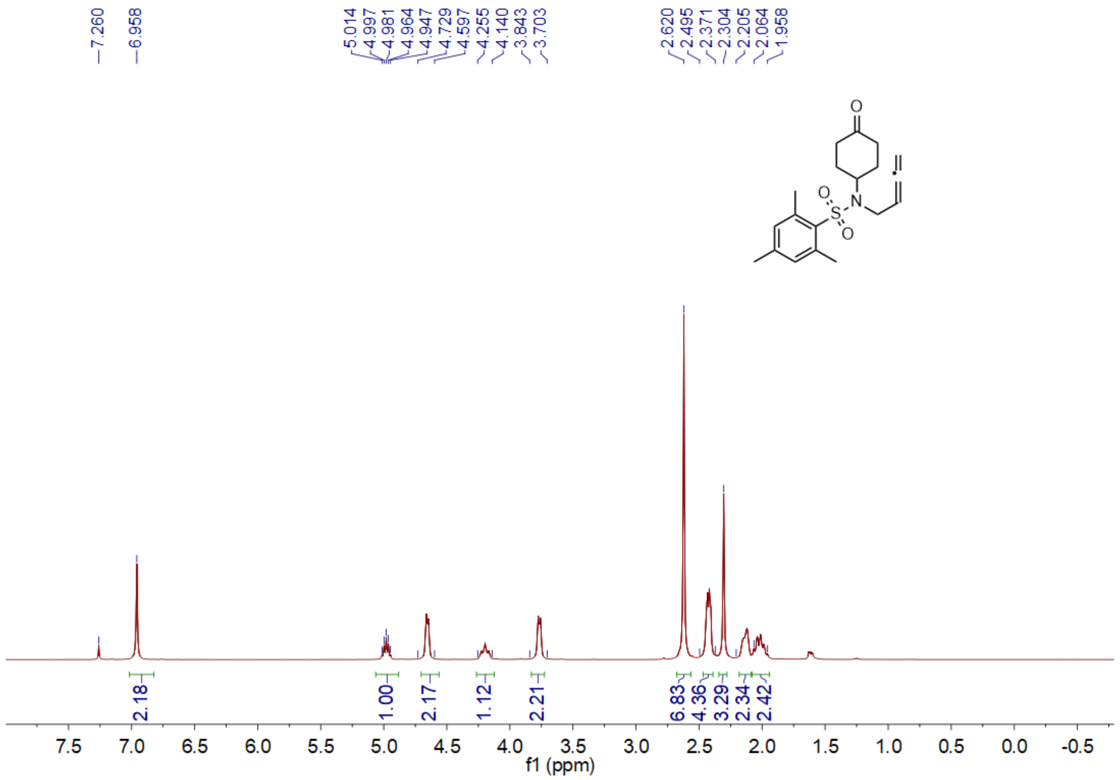

7.260
6.958
5.014
4.997
4.981
4.964
4.947
4.729
4.597
4.255
4.140
3.843
3.703
2.620
2.495
2.371
2.304
2.205
2.064
1.958
O
O
S
O
N
2.18
1.00
2.17
1.12
2.21
6.83
4.36
3.29
2.34
2.42
7.5 7.0 6.5 6.0 5.5 5.0 4.5 4.0 3.5 3.0 2.5 2.0 1.5 1.0 0.5 0.0 -0.5
f1 (ppm)

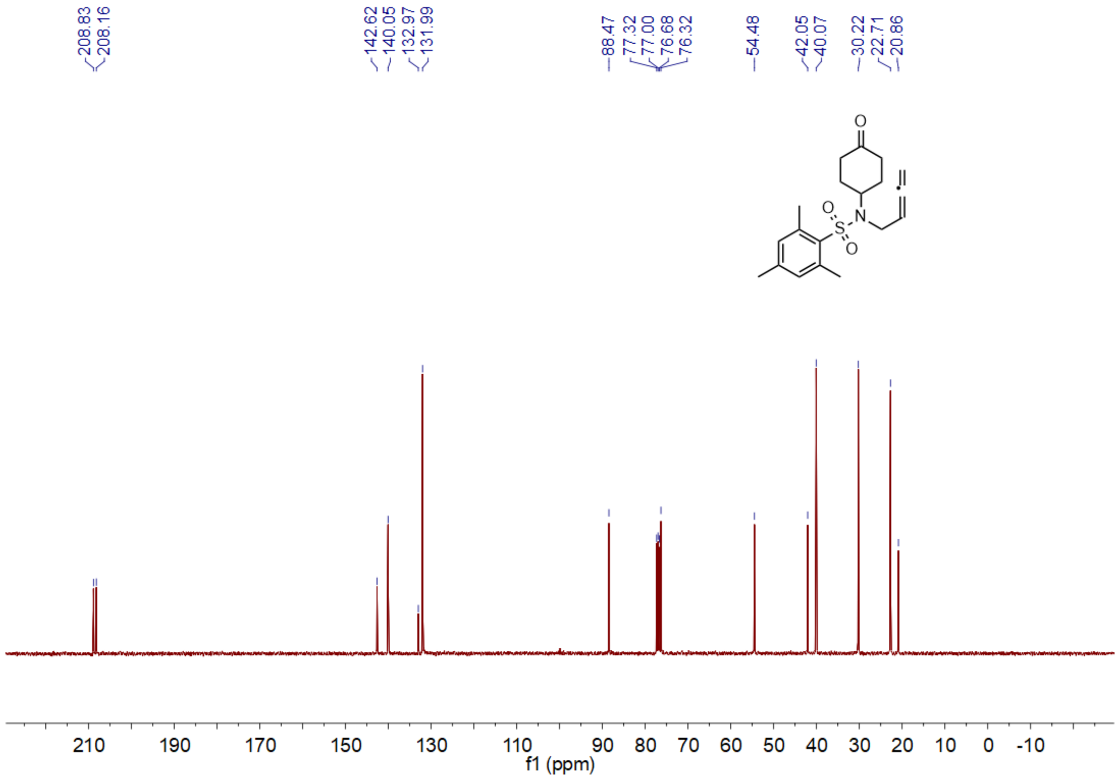

208.83
208.16
142.62
140.05
132.97
131.99
88.47
77.32
77.00
76.68
76.32
54.48
42.05
40.07
30.22
22.71
20.86
O
O
S
O
N
210 190 170 150 130 110 90 80 70 60 50 40 30 20 10 0 -10
f1 (ppm)

1j

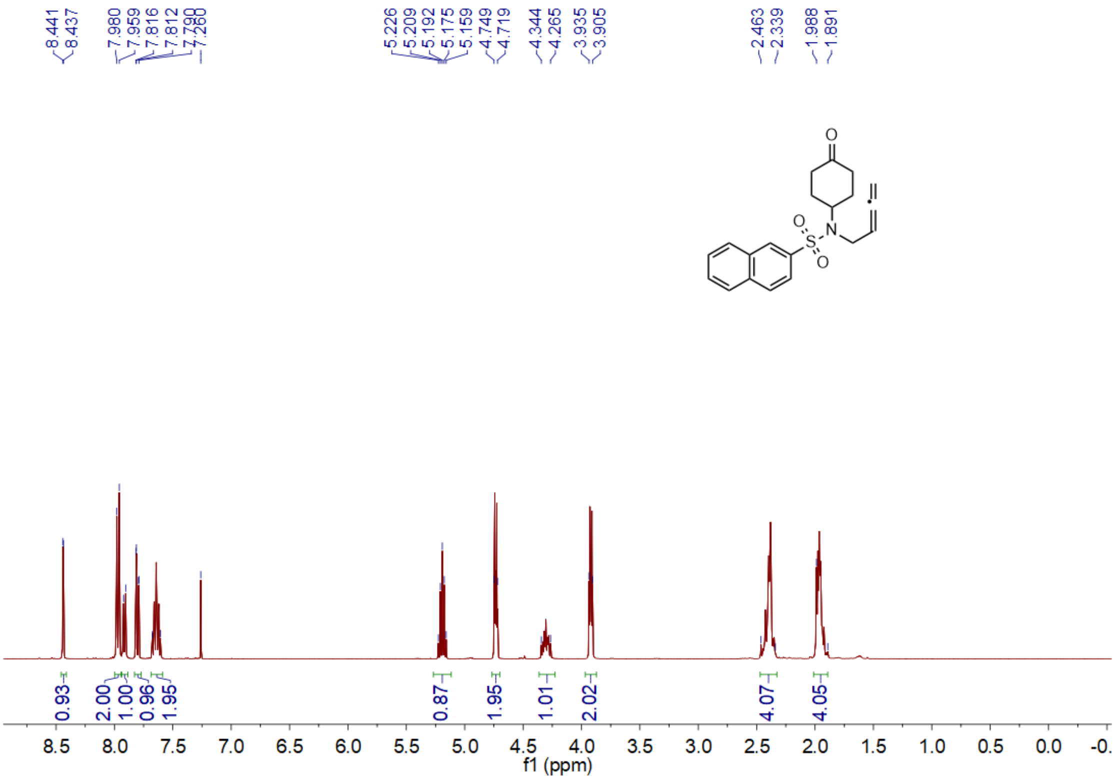
8.441
8.437
7.980
7.959
7.816
7.812
7.790
7.260
5.226
5.209
5.192
5.175
5.159
4.749
4.719
4.344
4.265
3.935
3.905
2.463
2.339
1.988
1.891
0.93
2.00
1.00
0.96
1.95
0.87
1.95
1.01
2.02
4.07
4.05
f1 (ppm)

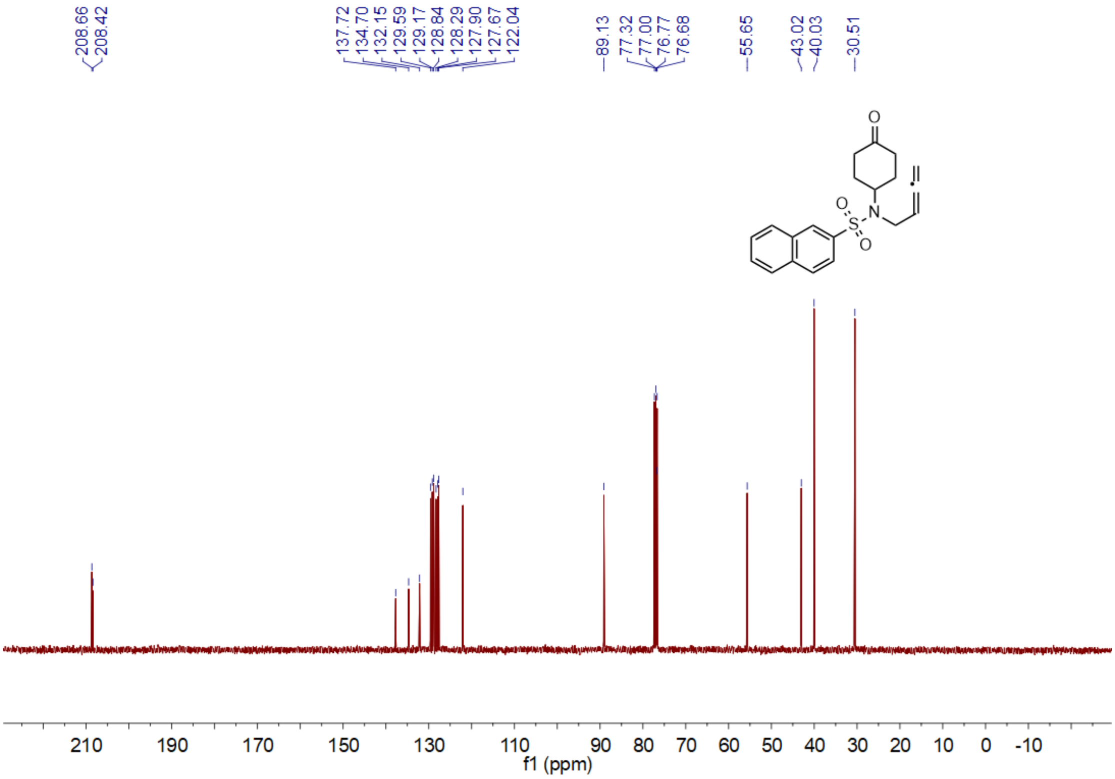
208.66
208.42
137.72
134.70
132.15
129.59
129.17
128.84
128.29
127.90
127.67
122.04
89.13
77.32
77.00
76.77
76.68
55.65
43.02
40.03
30.51
f1 (ppm)

2k

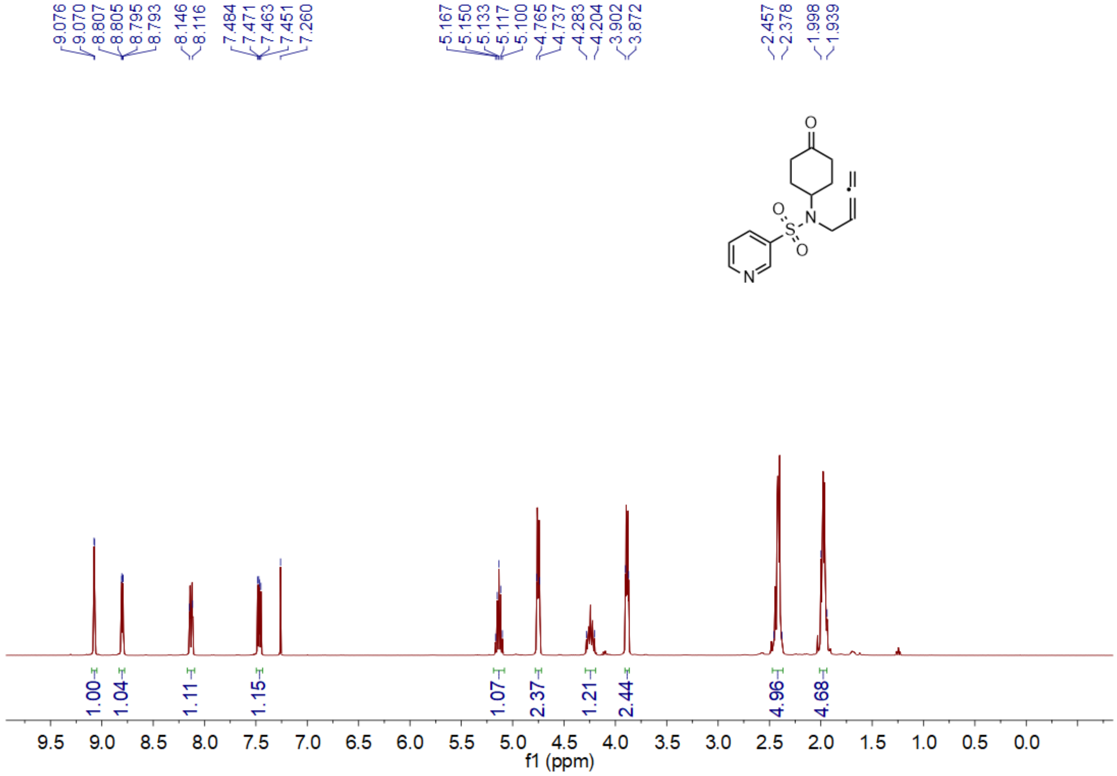
9.076
9.070
8.807
8.805
8.795
8.793
8.146
8.116
7.484
7.471
7.463
7.451
7.260
5.167
5.150
5.133
5.117
5.100
4.765
4.737
4.283
4.204
3.902
3.872
2.457
2.378
1.998
1.939
O
O
S
O
N
N
1.00
1.04
1.11
1.15
1.07
2.37
1.21
2.44
4.96
4.68
9.5 9.0 8.5 8.0 7.5 7.0 6.5 6.0 5.5 5.0 4.5 4.0 3.5 3.0 2.5 2.0 1.5 1.0 0.5 0.0
f1 (ppm)

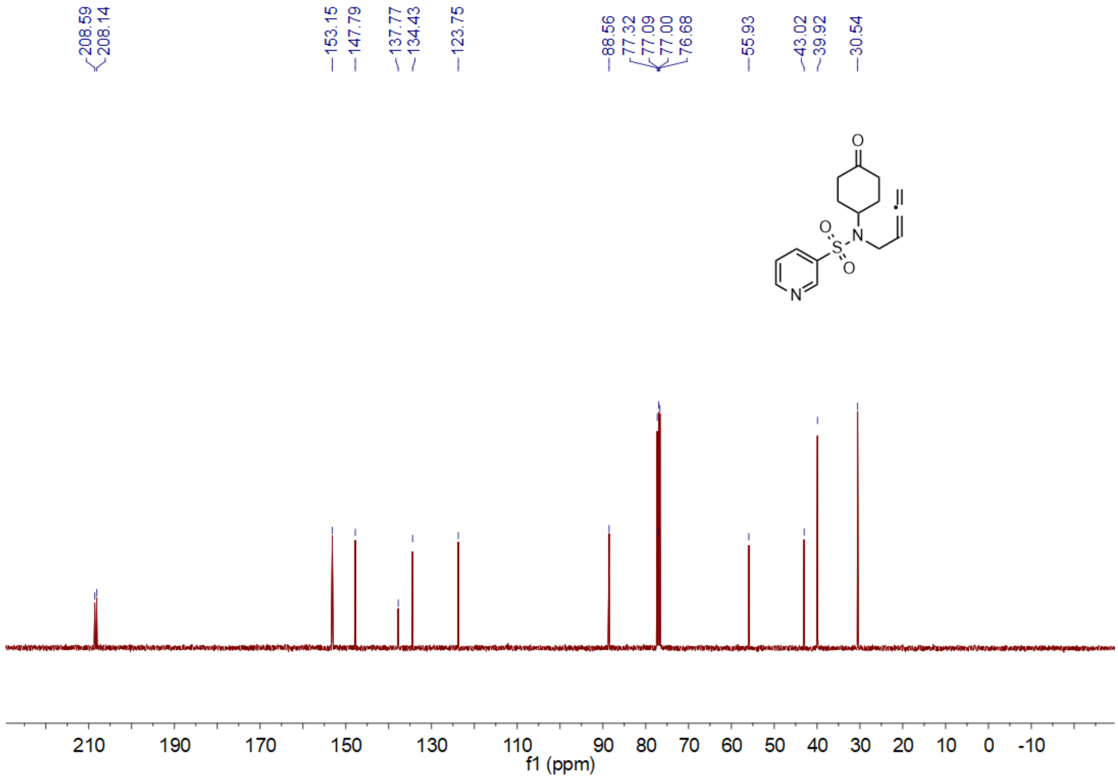
208.59
208.14
153.15
147.79
137.77
134.43
123.75
88.56
77.32
77.09
77.00
76.68
55.93
43.02
39.92
30.54
O
O
S
O
N
N
210 190 170 150 130 110 90 80 70 60 50 40 30 20 10 0 -10
f1 (ppm)

21

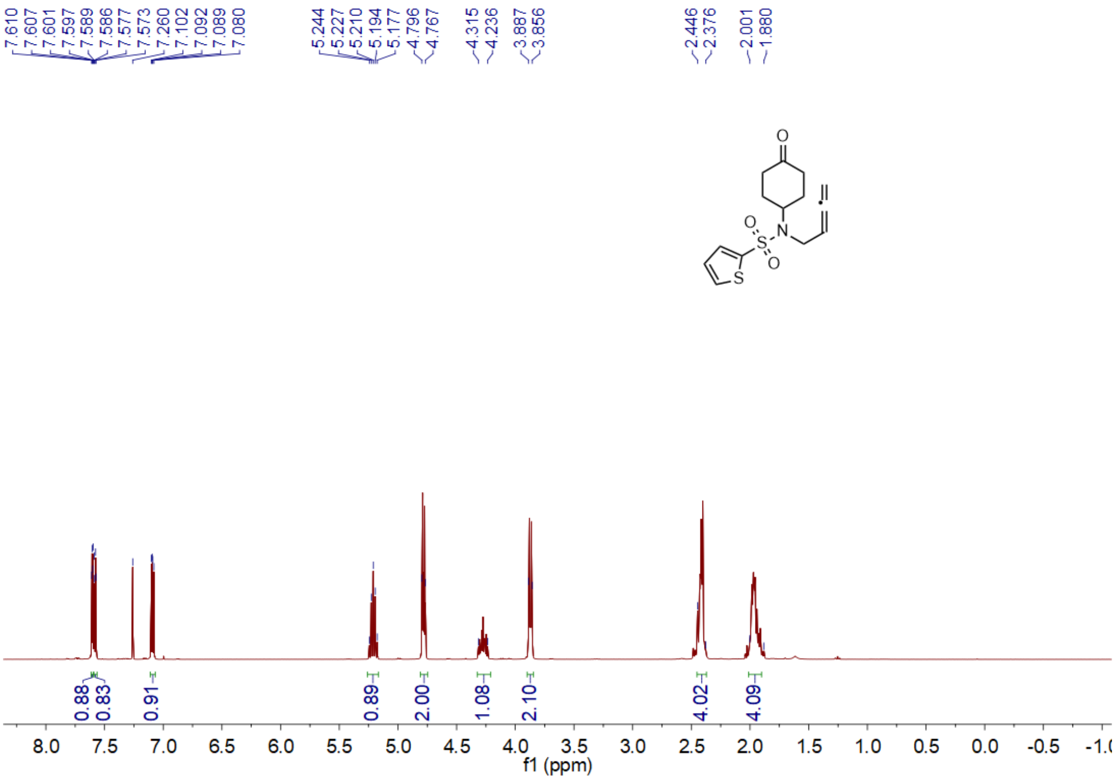
7.610
7.607
7.601
7.597
7.589
7.586
7.577
7.573
7.260
7.102
7.092
7.089
7.080
5.244
5.227
5.210
5.194
5.177
4.796
4.767
4.315
4.236
3.887
3.856
2.446
2.376
2.001
1.880
0.88
0.83
0.91
0.89
2.00
1.08
2.10
4.02
4.09
f1 (ppm)

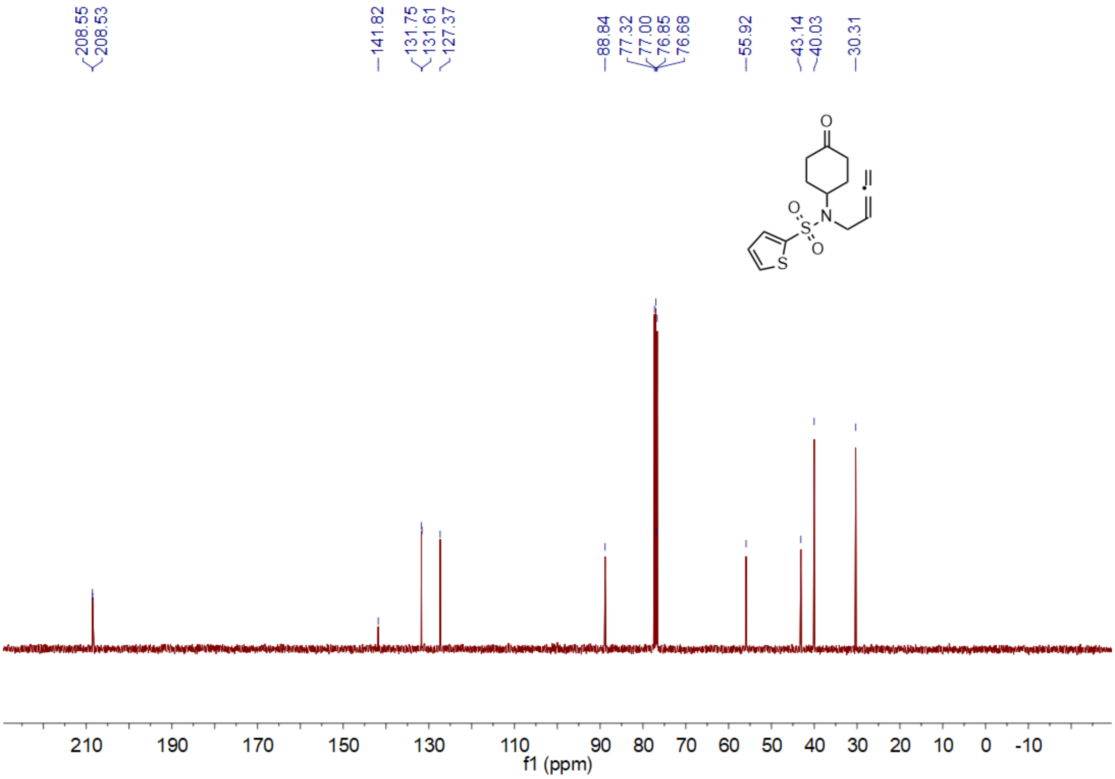
208.55
208.53
141.82
131.75
131.61
127.37
88.84
77.32
77.00
76.85
76.68
55.92
43.14
40.03
30.31
f1 (ppm)

2m

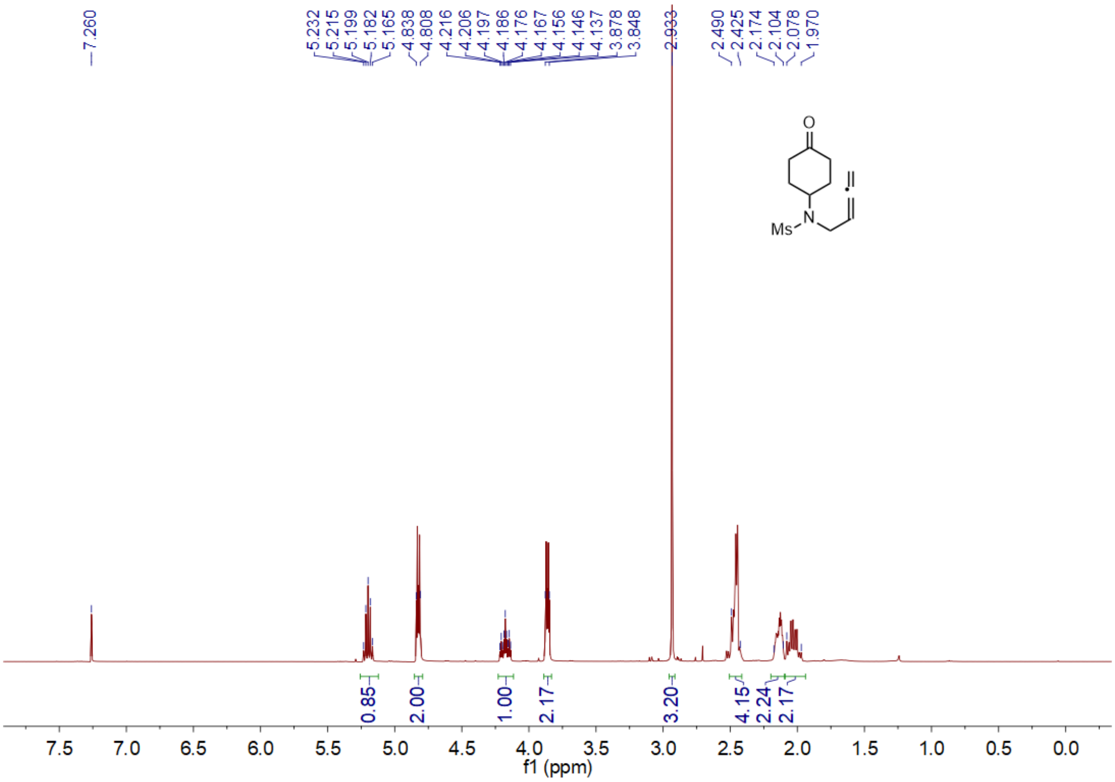

7.260
5.232
5.215
5.199
5.182
5.165
4.838
4.808
4.216
4.206
4.197
4.186
4.176
4.167
4.156
4.146
4.137
3.878
3.848
2.933
2.490
2.425
2.174
2.104
2.078
1.970
O
N
Ms
0.85
2.00
1.00
2.17
3.20
4.15
2.24
2.17
7.5
7.0
6.5
6.0
5.5
5.0
4.5
4.0
3.5
3.0
2.5
2.0
1.5
1.0
0.5
0.0
f1 (ppm)

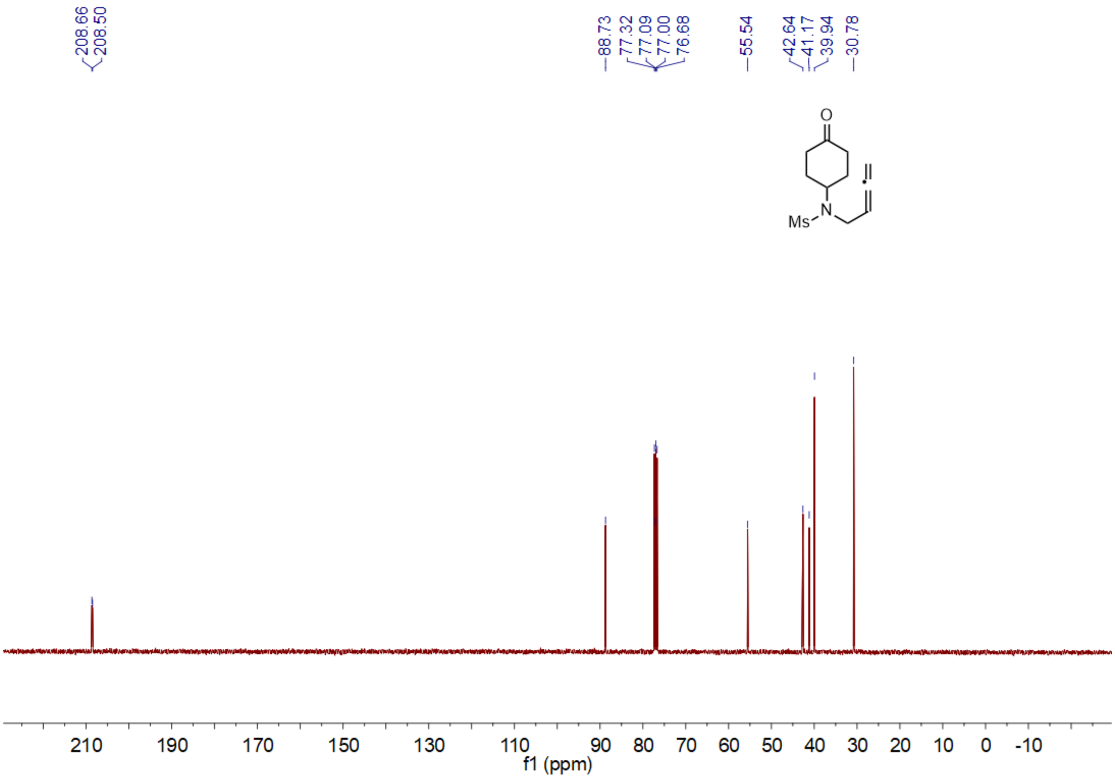

208.66
208.50
88.73
77.32
77.09
77.00
76.68
55.54
42.64
41.17
39.94
30.78
O
N
Ms
210
190
170
150
130
110
90
80
70
60
50
40
30
20
10
0
-10
f1 (ppm)

1n

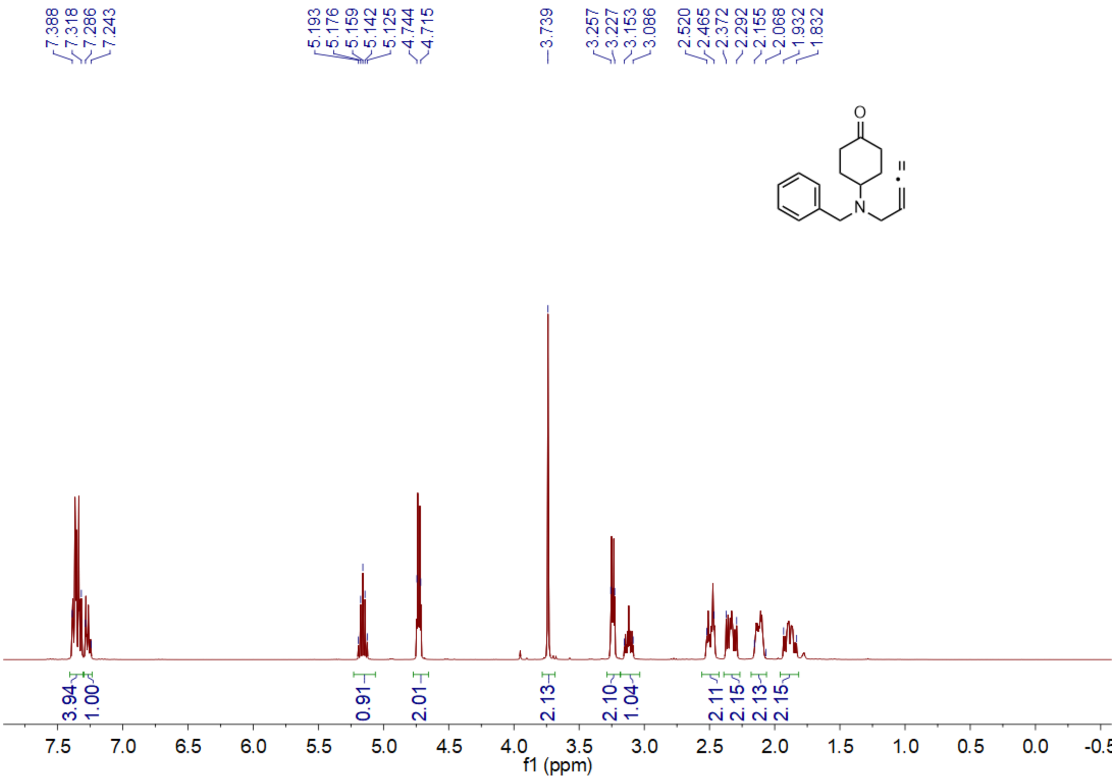
7.388
7.318
7.286
7.243
5.193
5.176
5.159
5.142
5.125
4.744
4.715
3.739
3.257
3.227
3.153
3.086
2.520
2.465
2.372
2.292
2.155
2.068
1.932
1.832
O
N
3.94
1.00
0.91
2.01
2.13
2.10
1.04
2.11
2.15
2.13
2.15
7.5 7.0 6.5 6.0 5.5 5.0 4.5 4.0 3.5 3.0 2.5 2.0 1.5 1.0 0.5 0.0 -0.5
f1 (ppm)

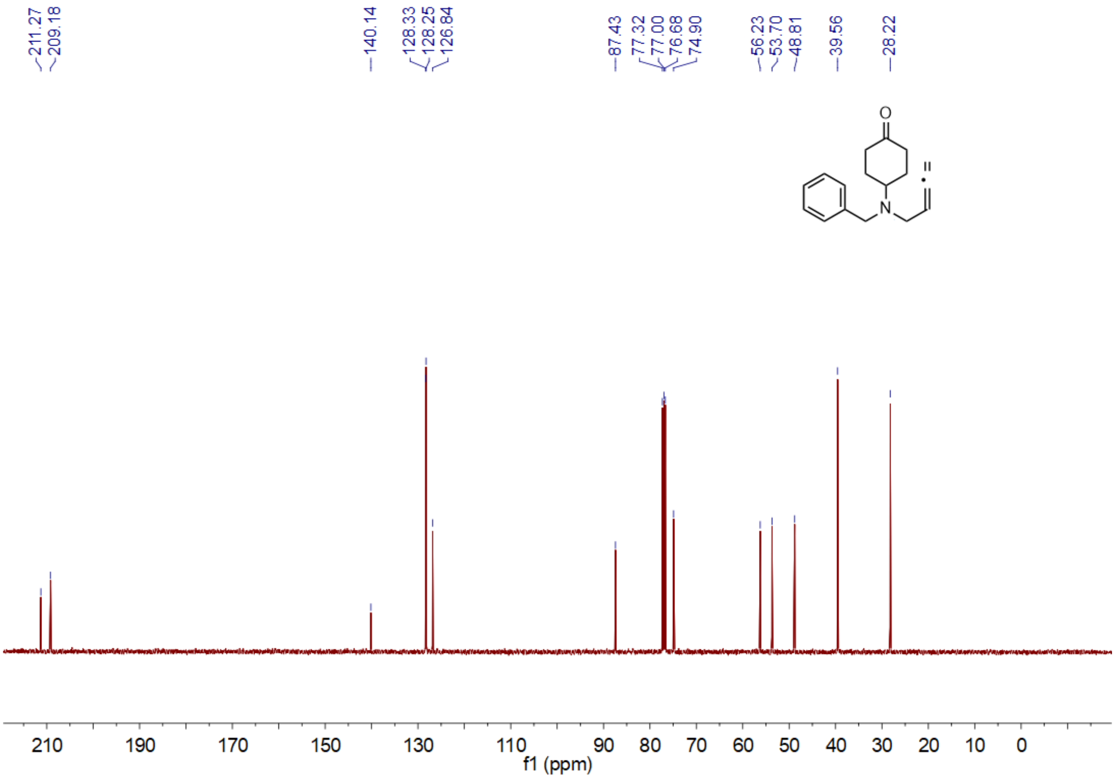
211.27
209.18
140.14
128.33
128.25
126.84
87.43
77.32
77.00
76.68
74.90
56.23
53.70
48.81
39.56
28.22
O
N
210 190 170 150 130 110 90 80 70 60 50 40 30 20 10 0
f1 (ppm)

1o

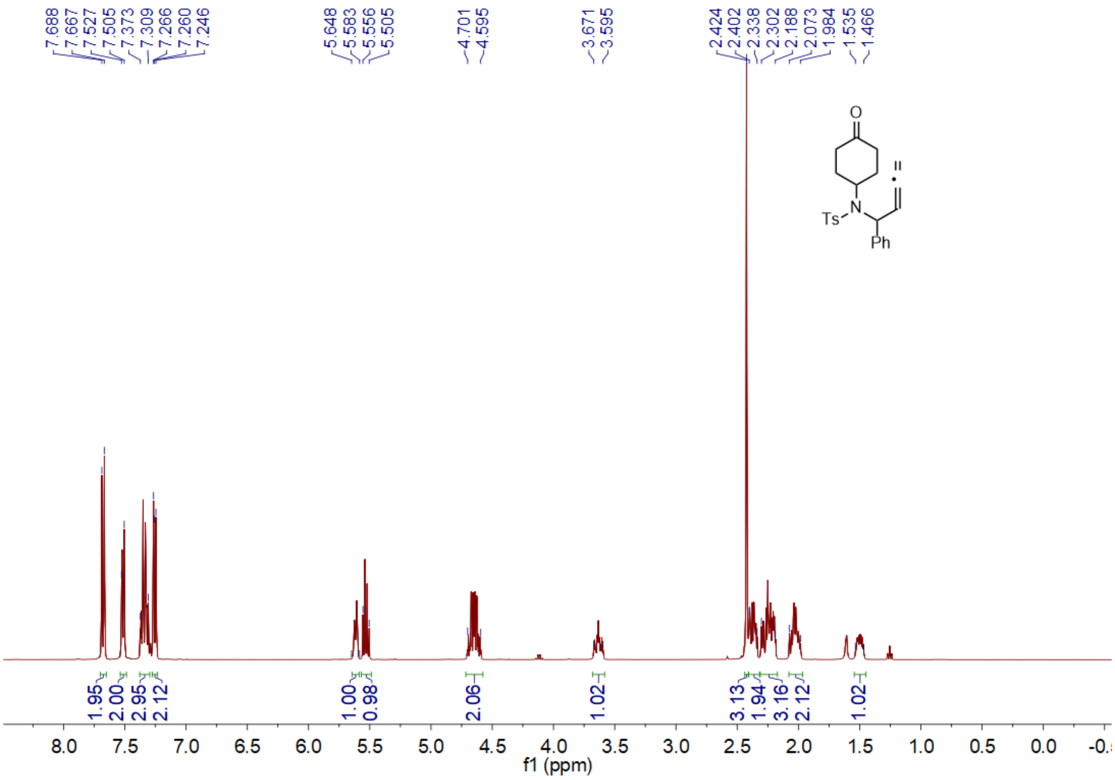
7.688
7.667
7.527
7.505
7.373
7.309
7.266
7.260
7.246
5.648
5.583
5.556
5.505
4.701
4.595
3.671
3.595
2.424
2.402
2.338
2.302
2.188
2.073
1.984
1.535
1.466
O
Ts
N
Ph
1.95
2.00
2.95
2.12
1.00
0.98
2.06
1.02
3.13
1.94
3.16
2.12
1.02
8.0
7.5
7.0
6.5
6.0
5.5
5.0
4.5
4.0
3.5
3.0
2.5
2.0
1.5
1.0
0.5
0.0
f1 (ppm)

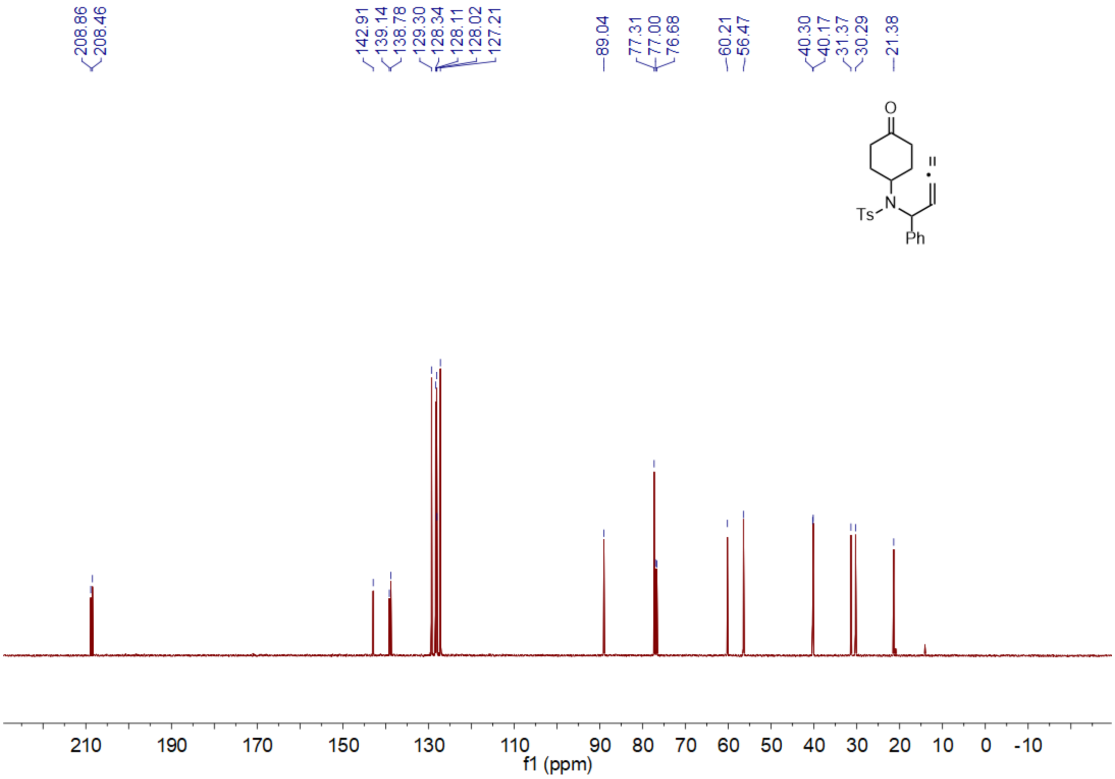
208.86
208.46
142.91
139.14
138.78
129.30
128.34
128.11
128.02
127.21
89.04
77.31
77.00
76.68
60.21
56.47
40.30
40.17
31.37
30.29
21.38
O
Ts
N
Ph
210
190
170
150
130
110
90
80
70
60
50
40
30
20
10
0
-10
f1 (ppm)

1p

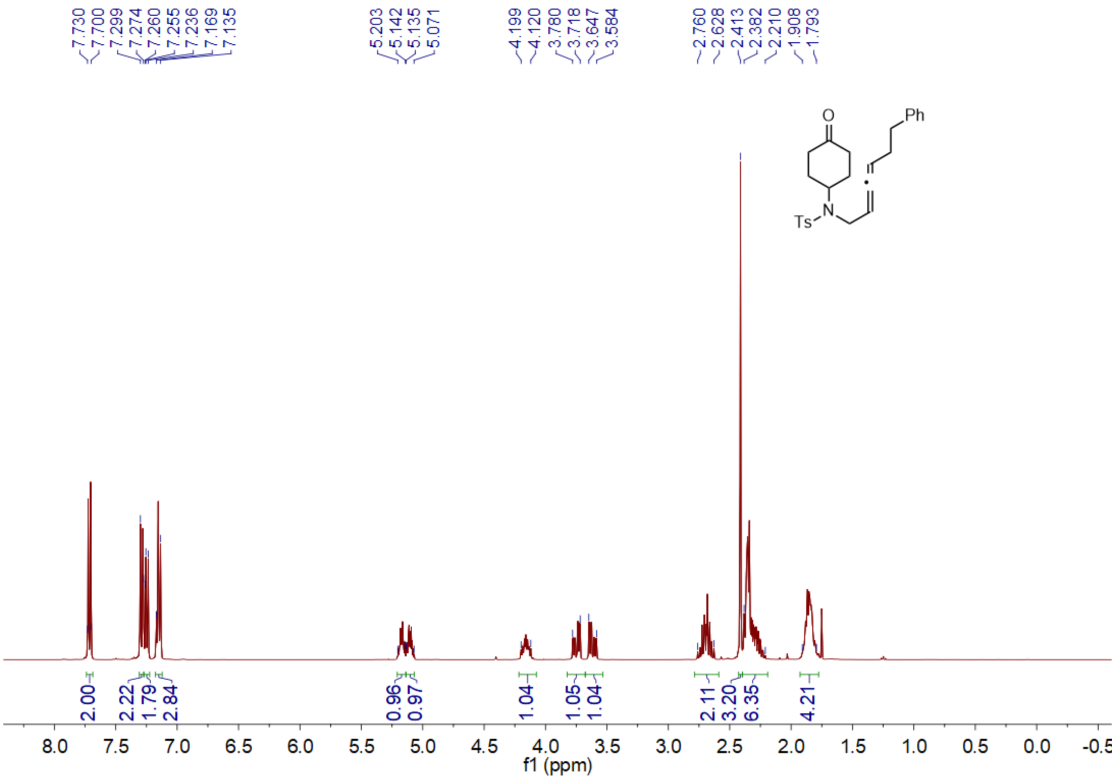

7.730
7.700
7.299
7.274
7.260
7.255
7.236
7.169
7.135
5.203
5.142
5.135
5.071
4.199
4.120
3.780
3.718
3.647
3.584
2.760
2.628
2.413
2.382
2.210
1.908
1.793
O
Ph
Ts
N
2.00
2.22
1.79
2.84
0.96
0.97
1.04
1.05
1.04
2.11
3.20
6.35
4.21
8.0
7.5
7.0
6.5
6.0
5.5
5.0
4.5
4.0
3.5
3.0
2.5
2.0
1.5
1.0
0.5
0.0
f1 (ppm)

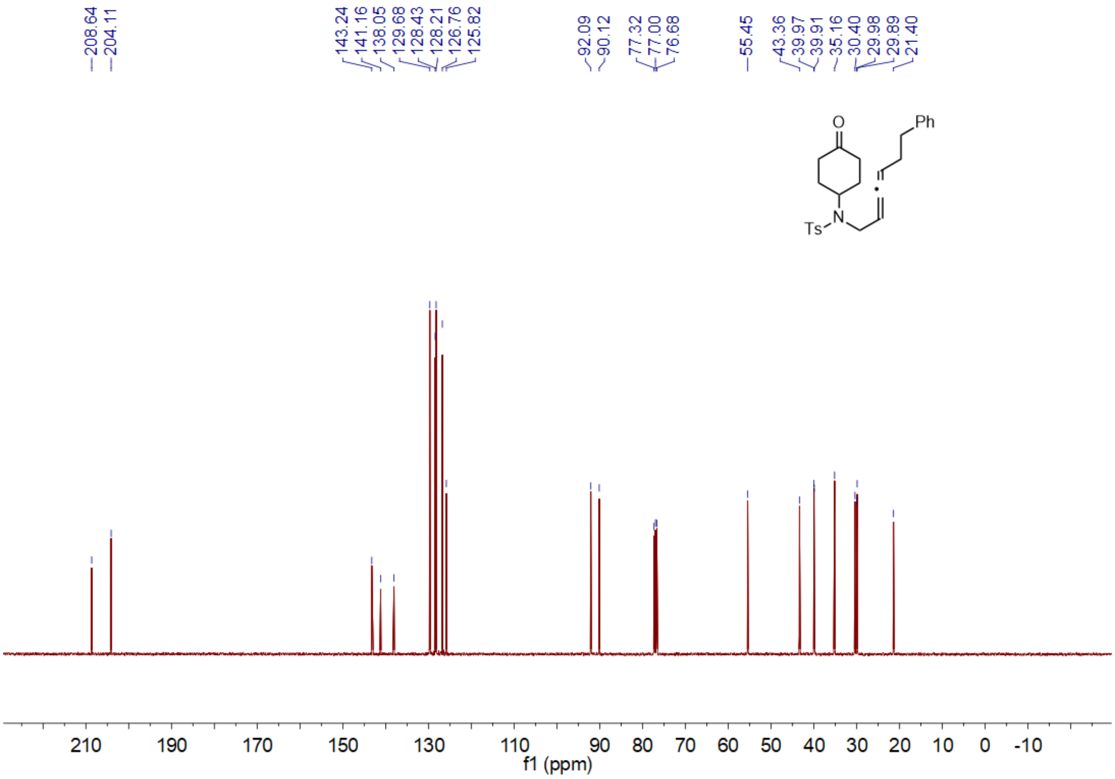

208.64
204.11
143.24
141.16
138.05
129.68
128.43
128.21
126.76
125.82
92.09
90.12
77.32
77.00
76.68
55.45
43.36
39.97
39.91
35.16
30.40
29.98
29.89
21.40
O
Ph
Ts
N
210
190
170
150
130
110
90
80
70
60
50
40
30
20
10
0
-10
f1 (ppm)

3a

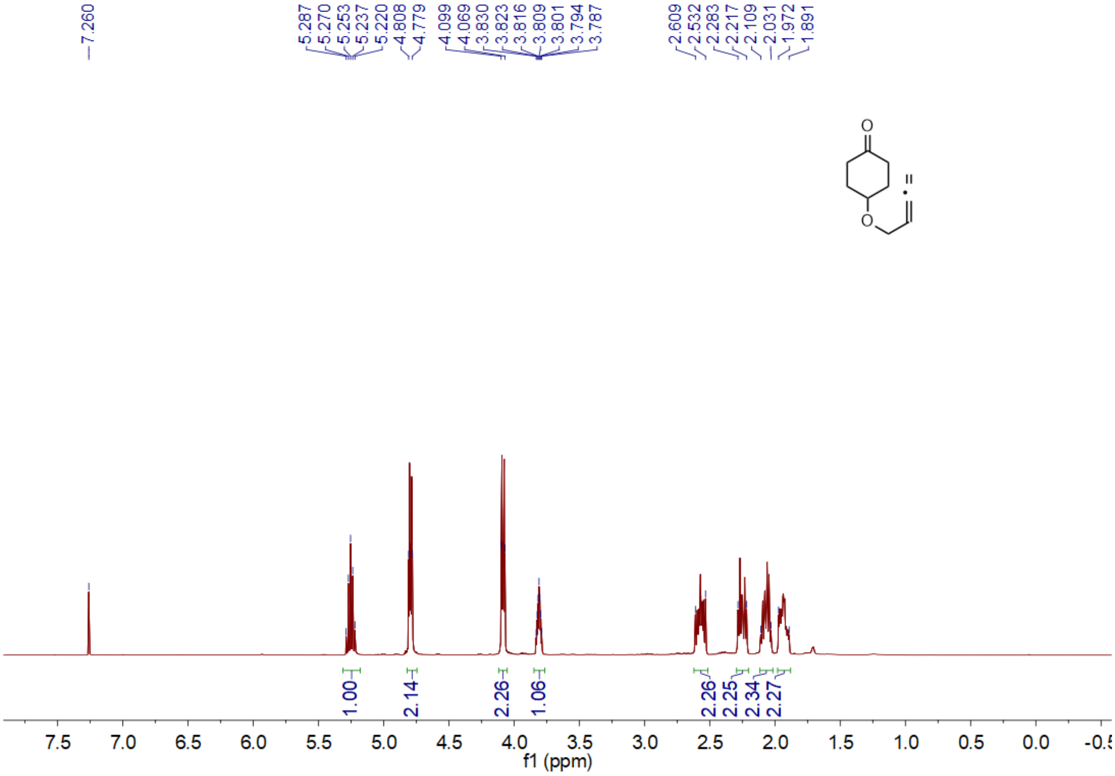
7.260
5.287
5.270
5.253
5.237
5.220
4.808
4.779
4.099
4.069
3.830
3.823
3.816
3.809
3.801
3.794
3.787
2.609
2.532
2.283
2.217
2.109
2.031
1.972
1.891
O
O
1.00
2.14
2.26
1.06
2.26
2.25
2.34
2.27
7.5
7.0
6.5
6.0
5.5
5.0
4.5
4.0
3.5
3.0
2.5
2.0
1.5
1.0
0.5
0.0
f1 (ppm)

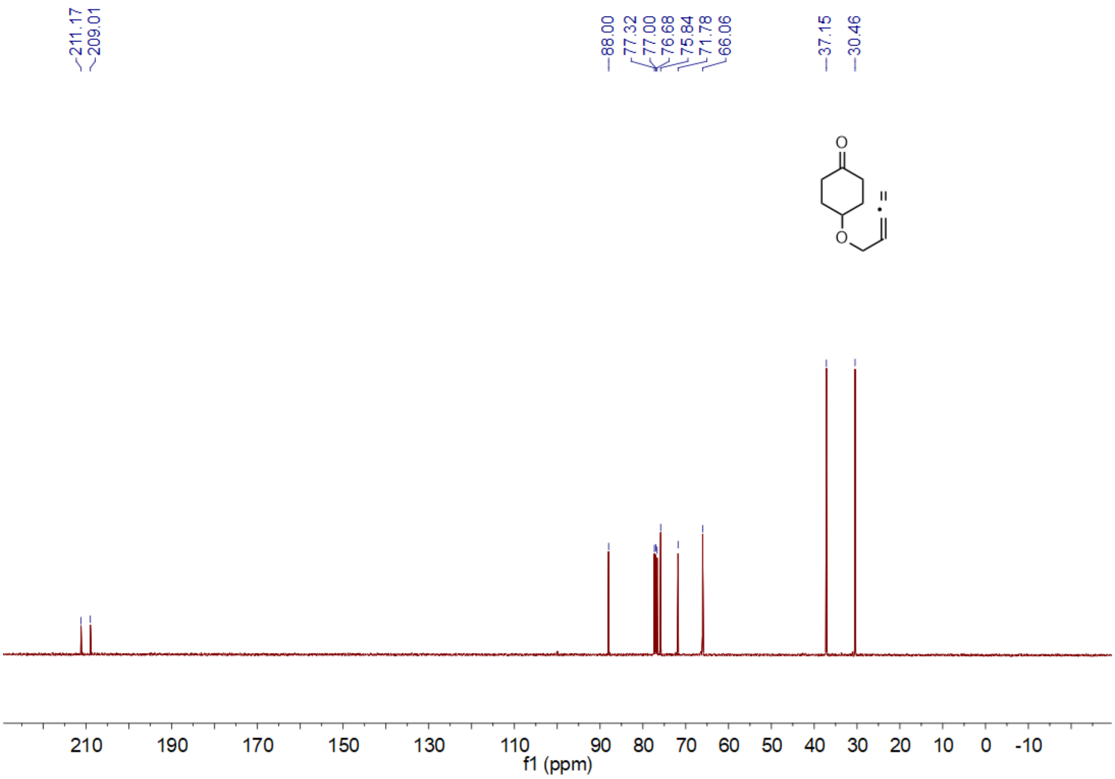
211.17
209.01
88.00
77.32
77.00
76.68
75.84
71.78
66.06
37.15
30.46
O
O
210
190
170
150
130
110
90
80
70
60
50
40
30
20
10
0
-10
f1 (ppm)

3b

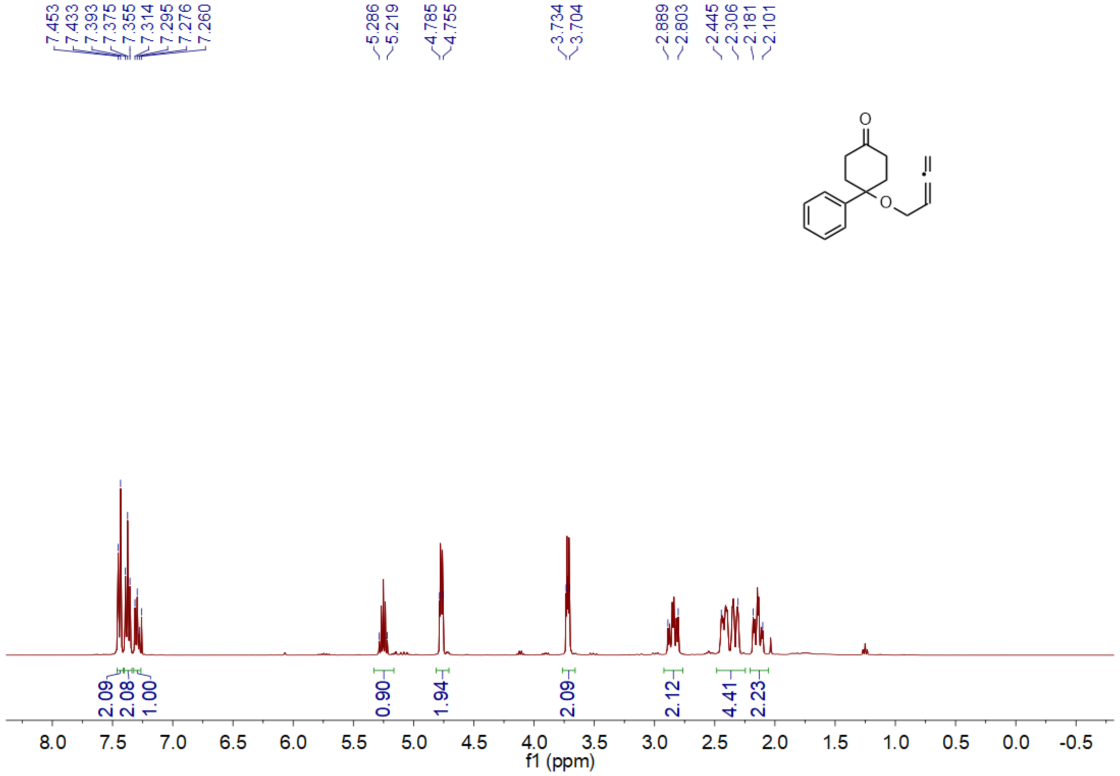
7.453
7.433
7.393
7.375
7.355
7.314
7.295
7.276
7.260
5.286
5.219
4.785
4.755
3.734
3.704
2.889
2.803
2.445
2.306
2.181
2.101
O
O
2.09
2.08
1.00
0.90
1.94
2.09
2.12
4.41
2.23
8.0 7.5 7.0 6.5 6.0 5.5 5.0 4.5 4.0 3.5 3.0 2.5 2.0 1.5 1.0 0.5 0.0 -0.5
f1 (ppm)

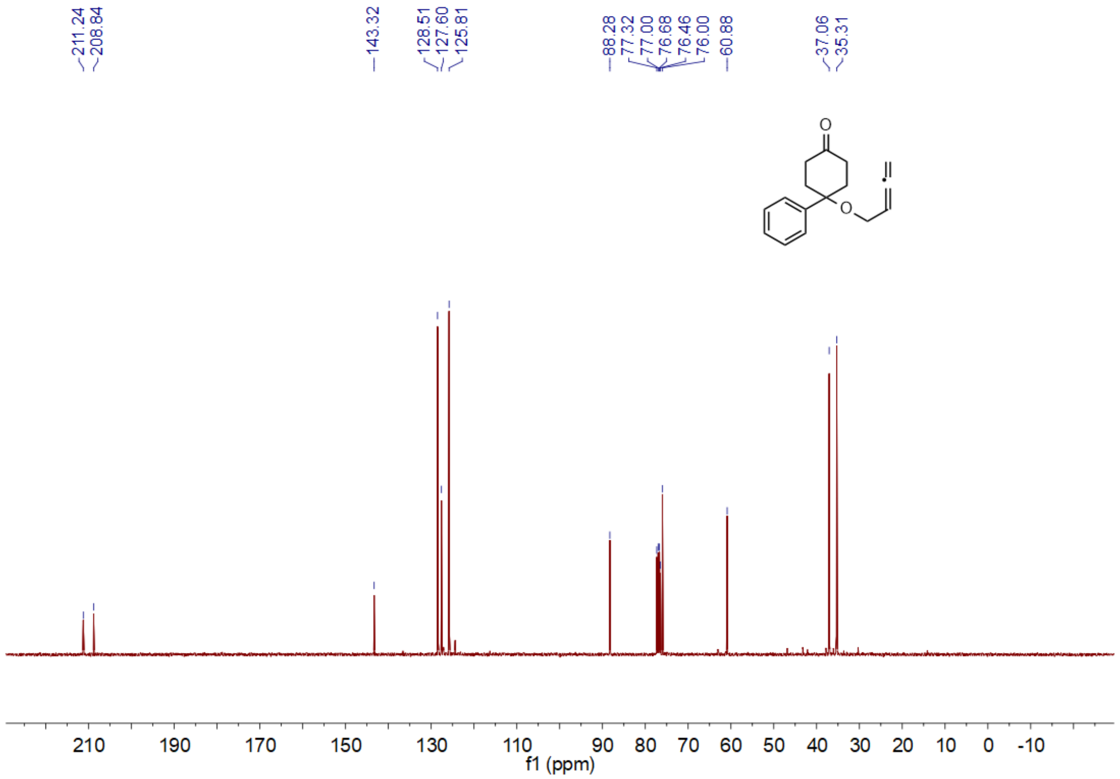
211.24
208.84
143.32
128.51
127.60
125.81
88.28
77.32
77.00
76.68
76.46
76.00
60.88
37.06
35.31
O
O
210 190 170 150 130 110 90 80 70 60 50 40 30 20 10 0 -10
f1 (ppm)

3c

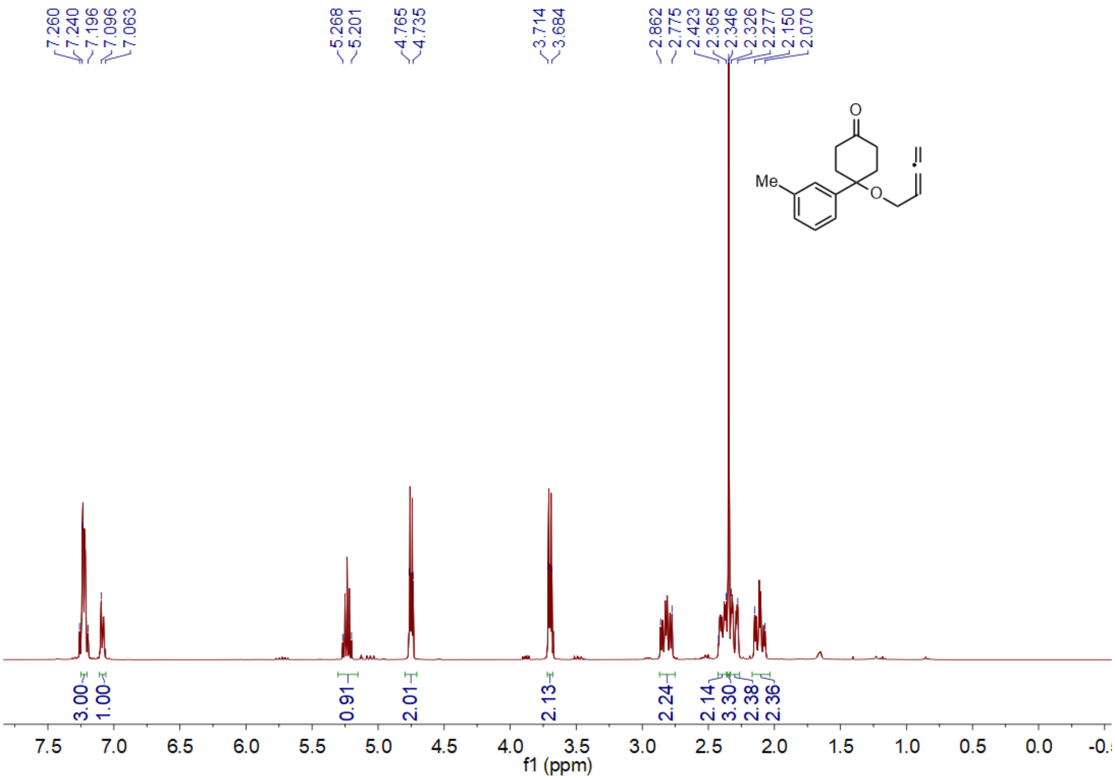
7.260
7.240
7.196
7.096
7.063
5.268
5.201
4.765
4.735
3.714
3.684
2.862
2.775
2.423
2.365
2.346
2.326
2.277
2.150
2.070
Me
O
3.00
1.00
0.91
2.01
2.13
2.24
2.14
3.30
2.38
2.36
7.5
7.0
6.5
6.0
5.5
5.0
4.5
4.0
3.5
3.0
2.5
2.0
1.5
1.0
0.5
0.0
f1 (ppm)

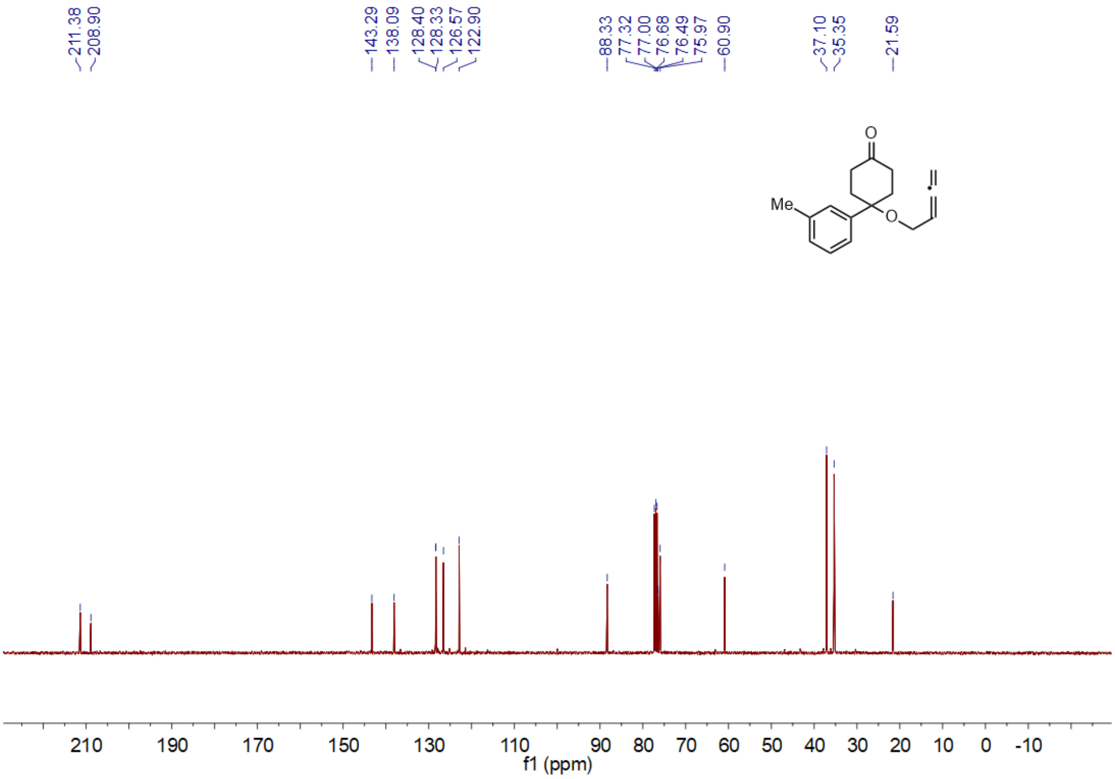
211.38
208.90
143.29
138.09
128.40
128.33
126.57
122.90
88.33
77.32
77.00
76.68
76.49
75.97
60.90
37.10
35.35
21.59
Me
O
210
190
170
150
130
110
90
80
70
60
50
40
30
20
10
0
-10
f1 (ppm)

3d

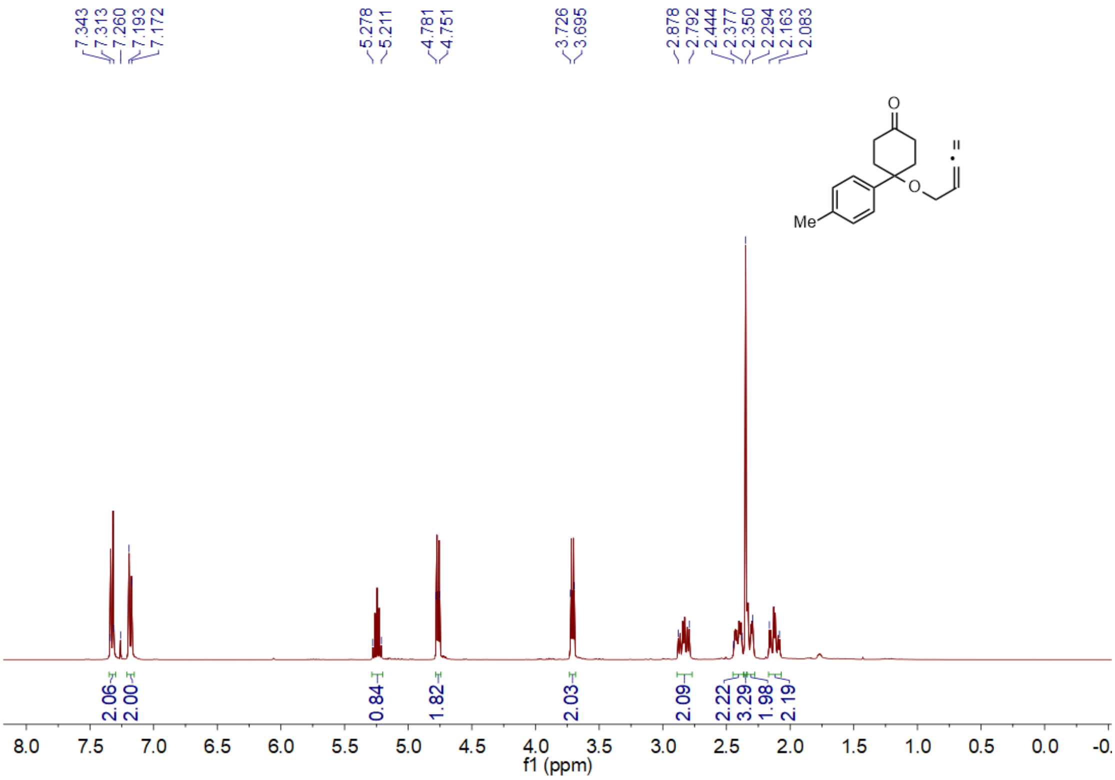

7.343
7.313
7.260
7.193
7.172
5.278
5.211
4.781
4.751
3.726
3.695
2.878
2.792
2.444
2.377
2.350
2.294
2.163
2.083
O
Me
2.06
2.00
0.84
1.82
2.03
2.09
2.22
3.29
1.98
2.19
8.0
7.5
7.0
6.5
6.0
5.5
5.0
4.5
4.0
3.5
3.0
2.5
2.0
1.5
1.0
0.5
0.0
f1 (ppm)

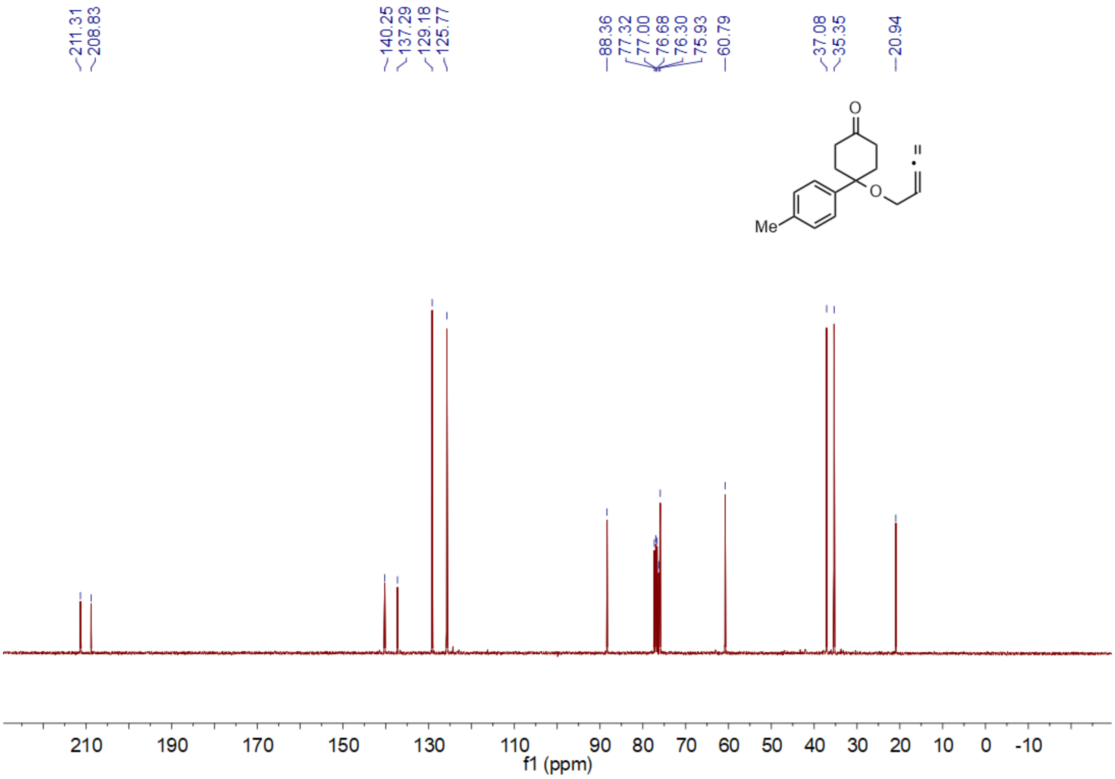

211.31
208.83
140.25
137.29
129.18
125.77
88.36
77.32
77.00
76.68
76.30
75.93
60.79
37.08
35.35
20.94
O
Me
210
190
170
150
130
110
90
80
70
60
50
40
30
20
10
0
-10
f1 (ppm)

3e

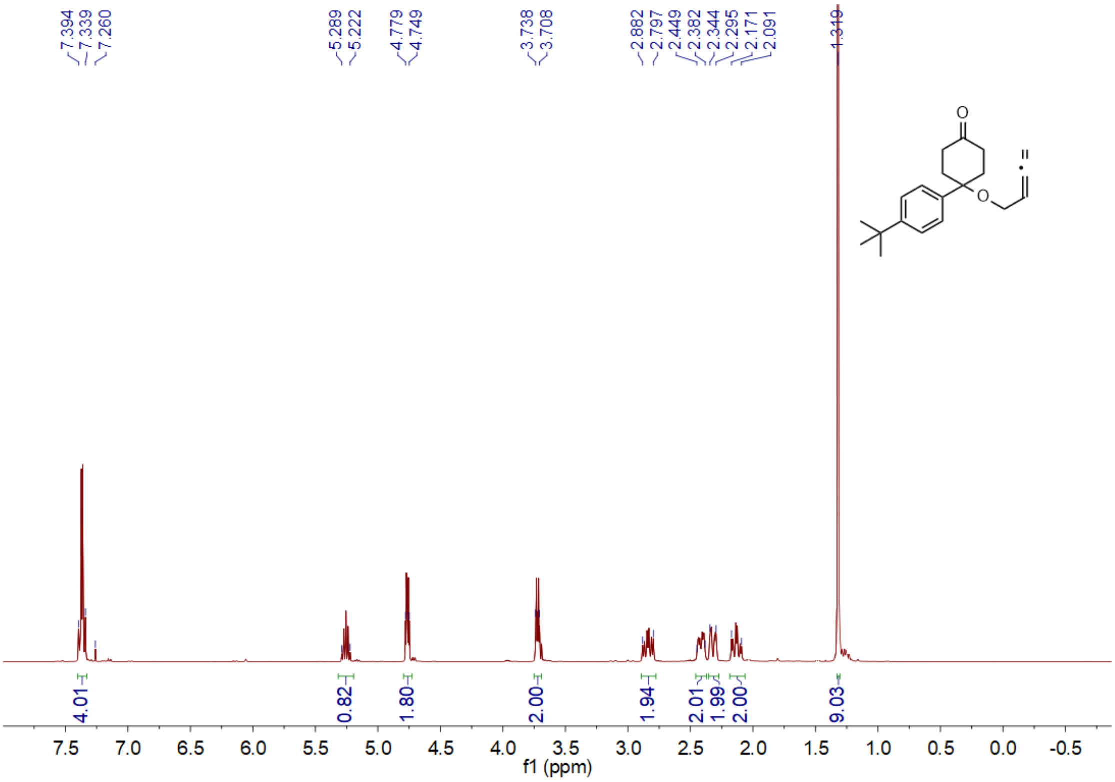
7.394
7.339
7.260
5.289
5.222
4.779
4.749
3.738
3.708
2.882
2.797
2.449
2.382
2.344
2.295
2.171
2.091
1.319
O
O
4.01
0.82
1.80
2.00
1.94
2.01
1.99
2.00
9.03
7.5 7.0 6.5 6.0 5.5 5.0 4.5 4.0 3.5 3.0 2.5 2.0 1.5 1.0 0.5 0.0 -0.5
f1 (ppm)

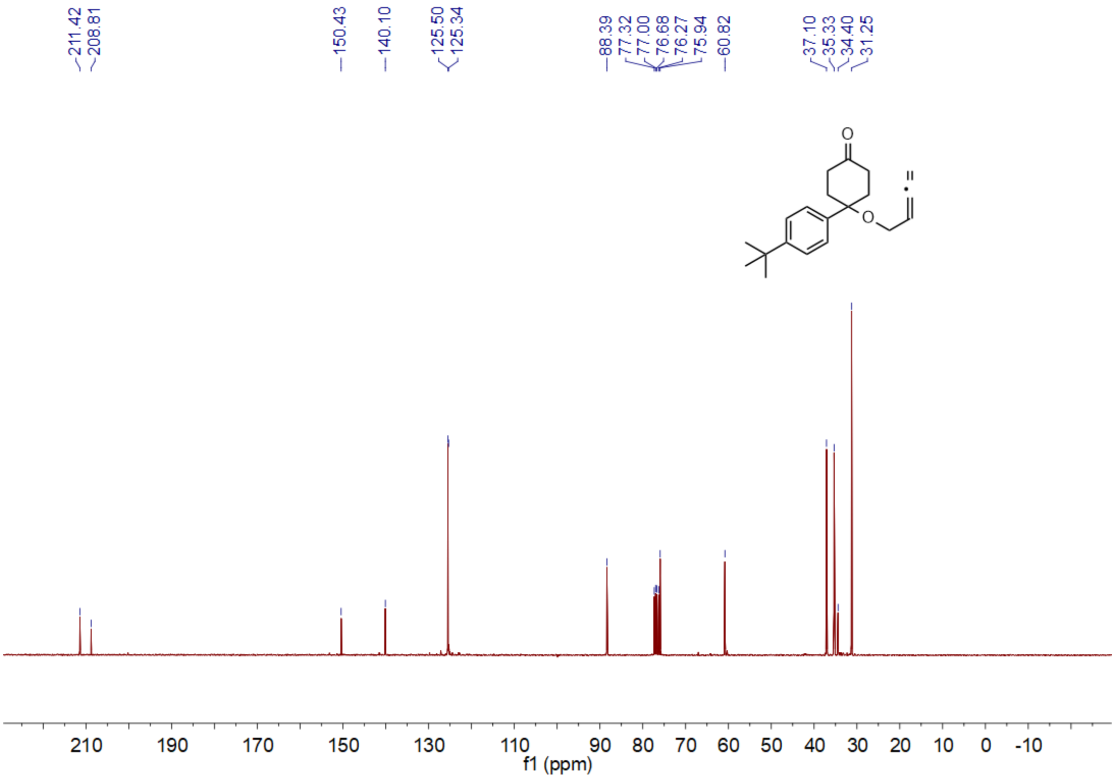
211.42
208.81
150.43
140.10
125.50
125.34
88.39
77.32
77.00
76.68
76.27
75.94
60.82
37.10
35.33
34.40
31.25
O
O
210 190 170 150 130 110 90 80 70 60 50 40 30 20 10 0 -10
f1 (ppm)

3f

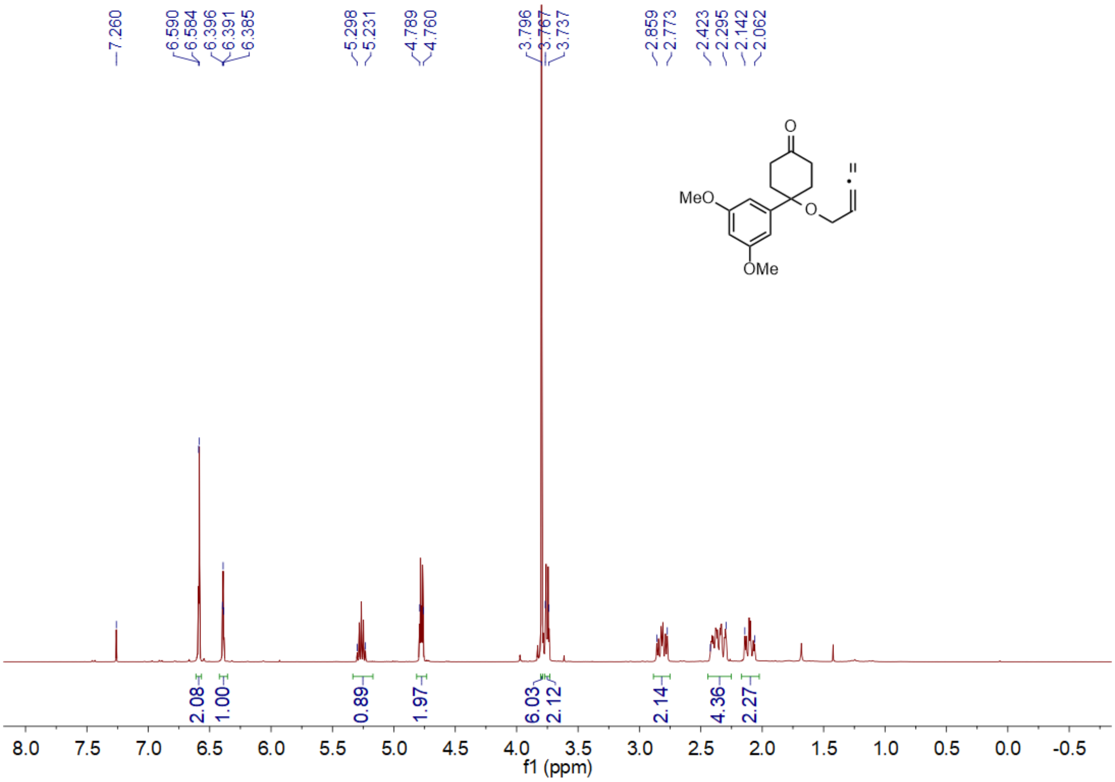
7.260
6.590
6.584
6.396
6.391
6.385
5.298
5.231
4.789
4.760
3.796
3.767
3.737
2.859
2.773
2.423
2.295
2.142
2.062
MeO
OMe
O
2.08
1.00
0.89
1.97
6.03
2.12
2.14
4.36
2.27
8.0 7.5 7.0 6.5 6.0 5.5 5.0 4.5 4.0 3.5 3.0 2.5 2.0 1.5 1.0 0.5 0.0 -0.5
f1 (ppm)

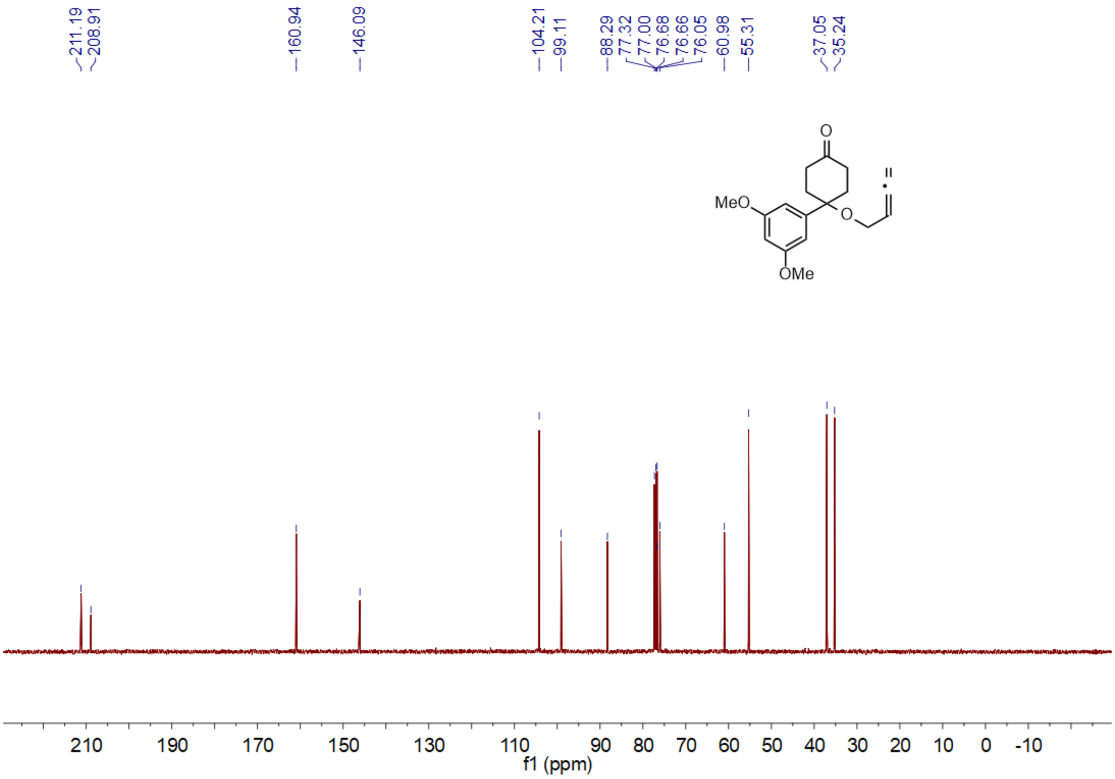
211.19
208.91
160.94
146.09
104.21
99.11
88.29
77.32
77.00
76.68
76.66
76.05
60.98
55.31
37.05
35.24
MeO
OMe
O
210 190 170 150 130 110 90 80 70 60 50 40 30 20 10 0 -10
f1 (ppm)

3g

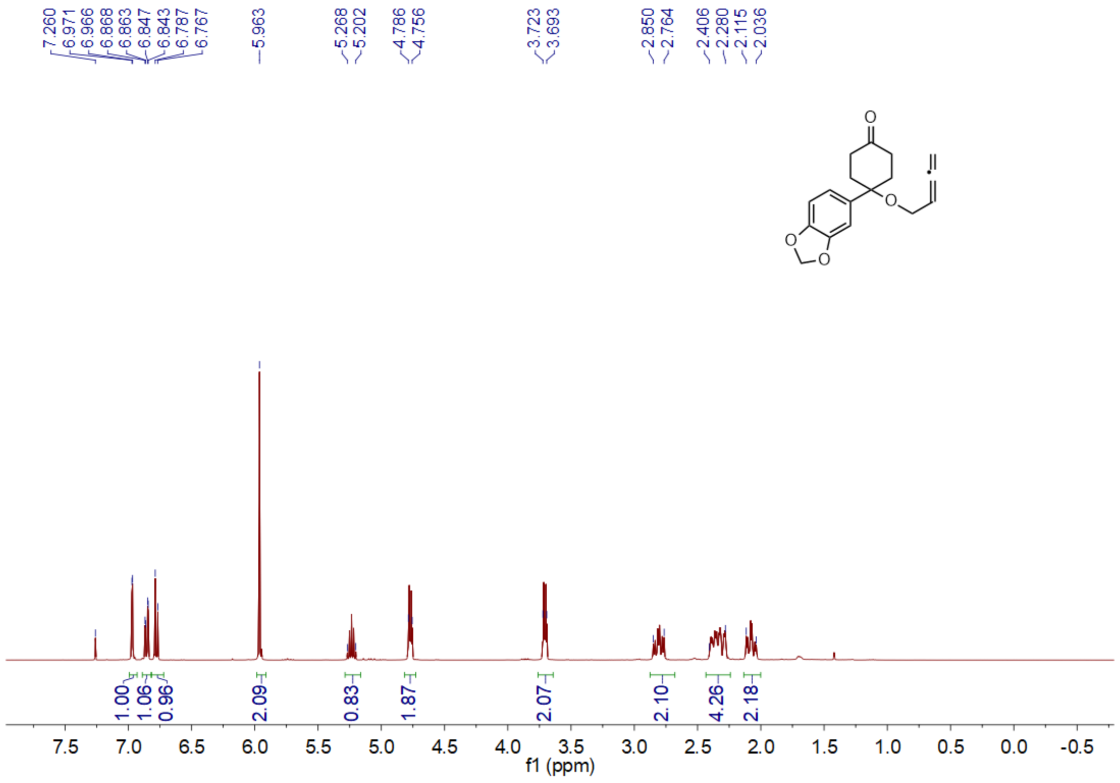
7.260
6.971
6.966
6.868
6.863
6.847
6.843
6.787
6.767
5.963
5.268
5.202
4.786
4.756
3.723
3.693
2.850
2.764
2.406
2.280
2.115
2.036
1.00
1.06
0.96
2.09
0.83
1.87
2.07
2.10
4.26
2.18
f1 (ppm)

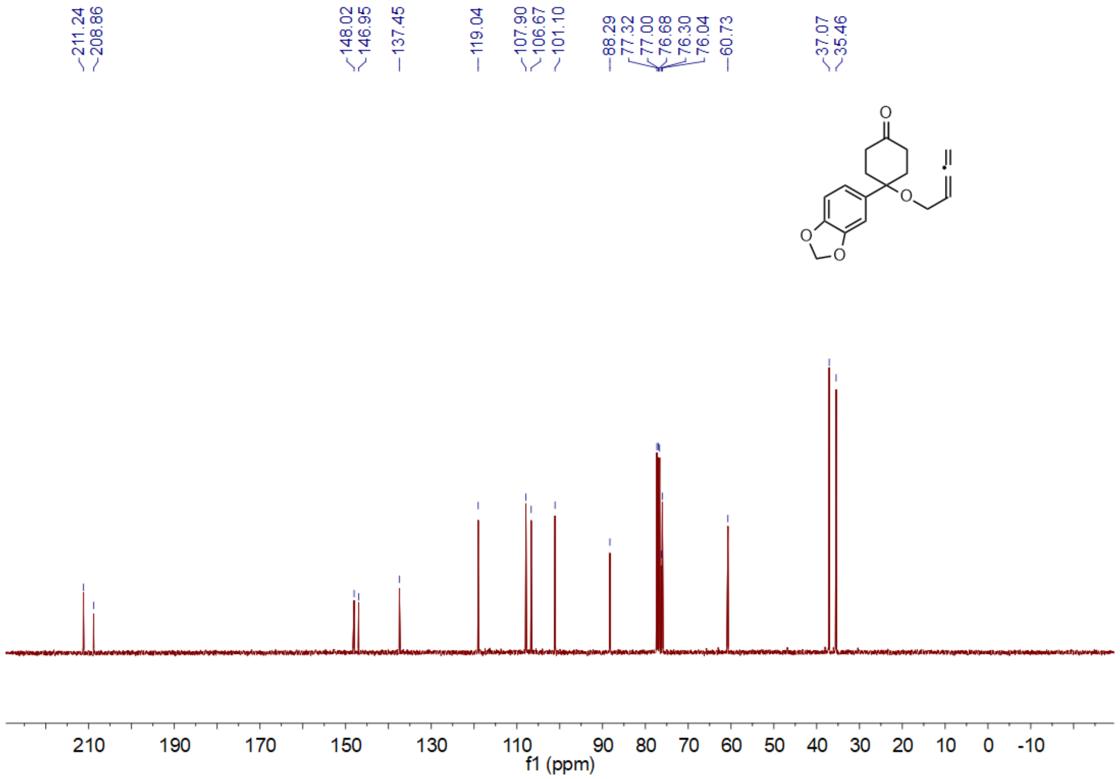
211.24
208.86
148.02
146.95
137.45
119.04
107.90
106.67
101.10
88.29
77.32
77.00
76.68
76.30
76.04
60.73
37.07
35.46
f1 (ppm)

3h

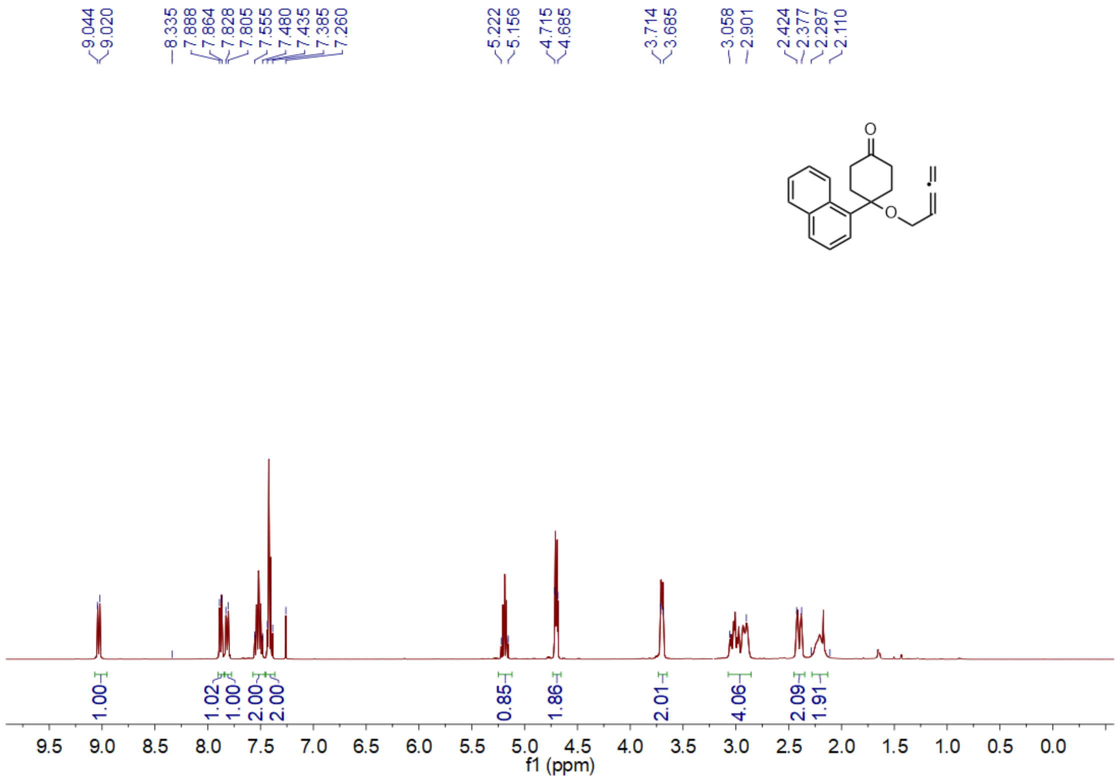

9.044
9.020
8.335
7.888
7.864
7.828
7.805
7.555
7.480
7.435
7.385
7.260
5.222
5.156
4.715
4.685
3.714
3.685
3.058
2.901
2.424
2.377
2.287
2.110
O
O
1.00
1.02
1.00
2.00
2.00
0.85
1.86
2.01
4.06
2.09
1.91
9.5 9.0 8.5 8.0 7.5 7.0 6.5 6.0 5.5 5.0 4.5 4.0 3.5 3.0 2.5 2.0 1.5 1.0 0.5 0.0
f1 (ppm)

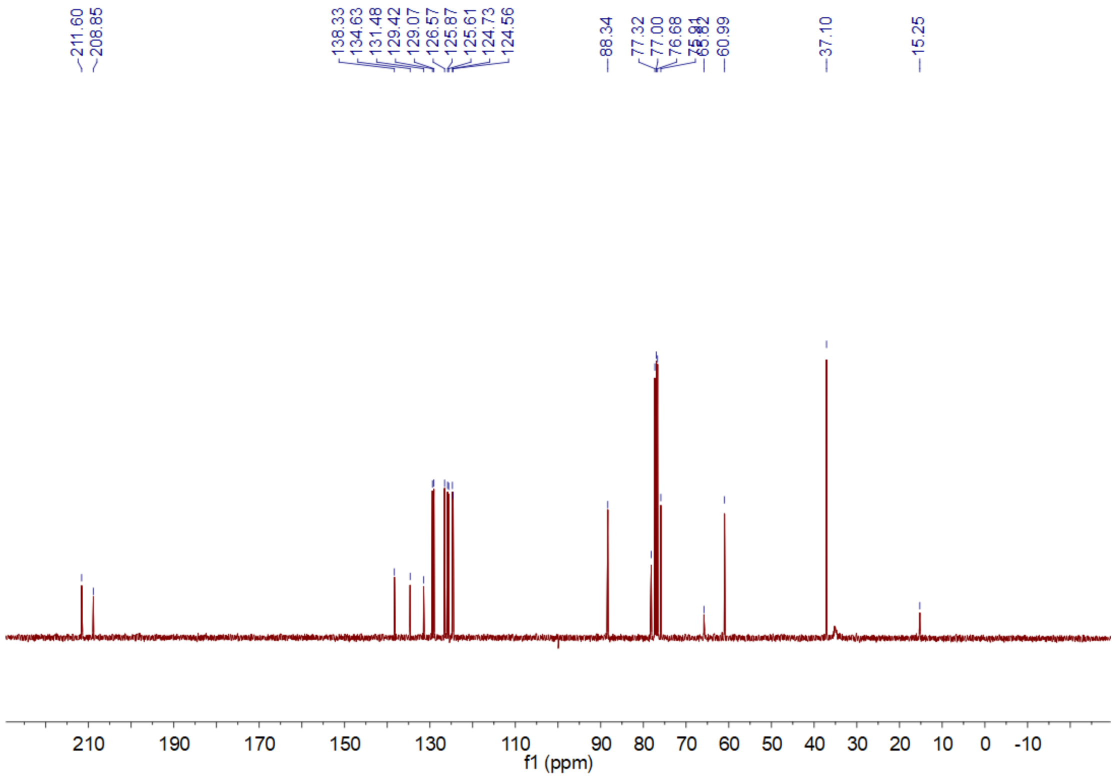

211.60
208.85
138.33
134.63
131.48
129.42
129.07
126.57
125.87
125.61
124.73
124.56
88.34
77.32
77.00
76.68
75.91
65.82
60.99
37.10
15.25
210 190 170 150 130 110 90 80 70 60 50 40 30 20 10 0 -10
f1 (ppm)

3i

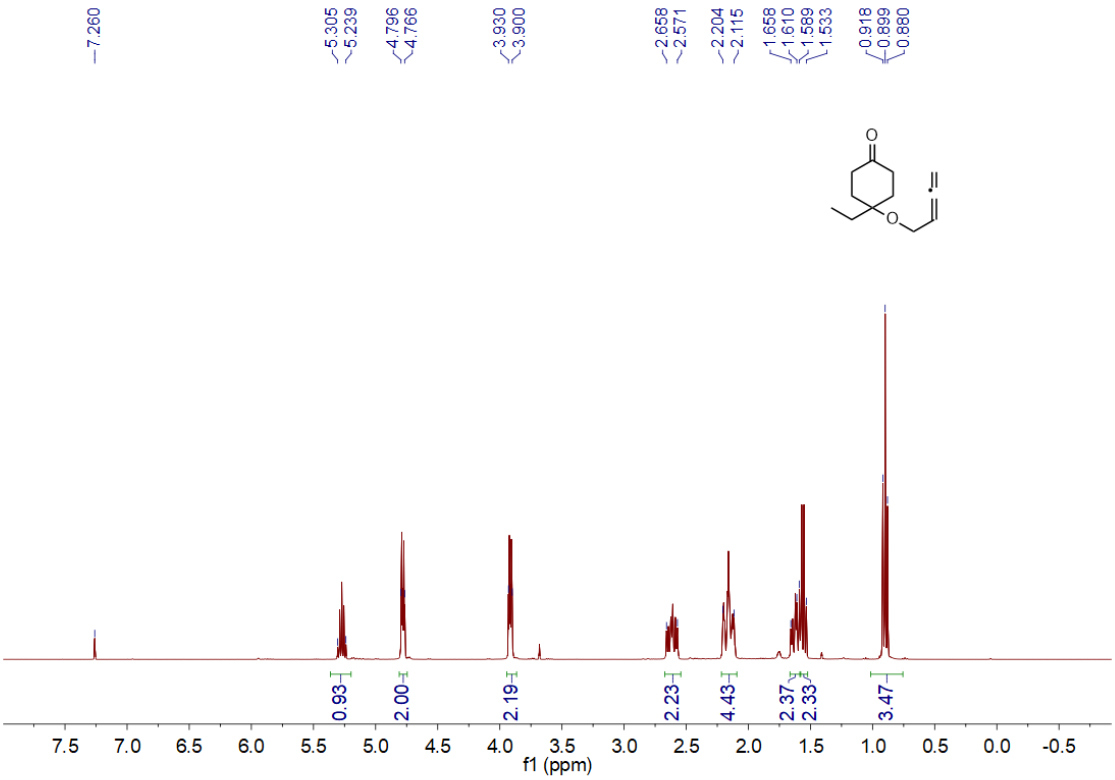
7.260
5.305
5.239
4.796
4.766
3.930
3.900
2.658
2.571
2.204
2.115
1.658
1.610
1.589
1.533
0.918
0.899
0.880
O
O
0.93
2.00
2.19
2.23
4.43
2.37
2.33
3.47
7.5 7.0 6.5 6.0 5.5 5.0 4.5 4.0 3.5 3.0 2.5 2.0 1.5 1.0 0.5 0.0 -0.5
f1 (ppm)

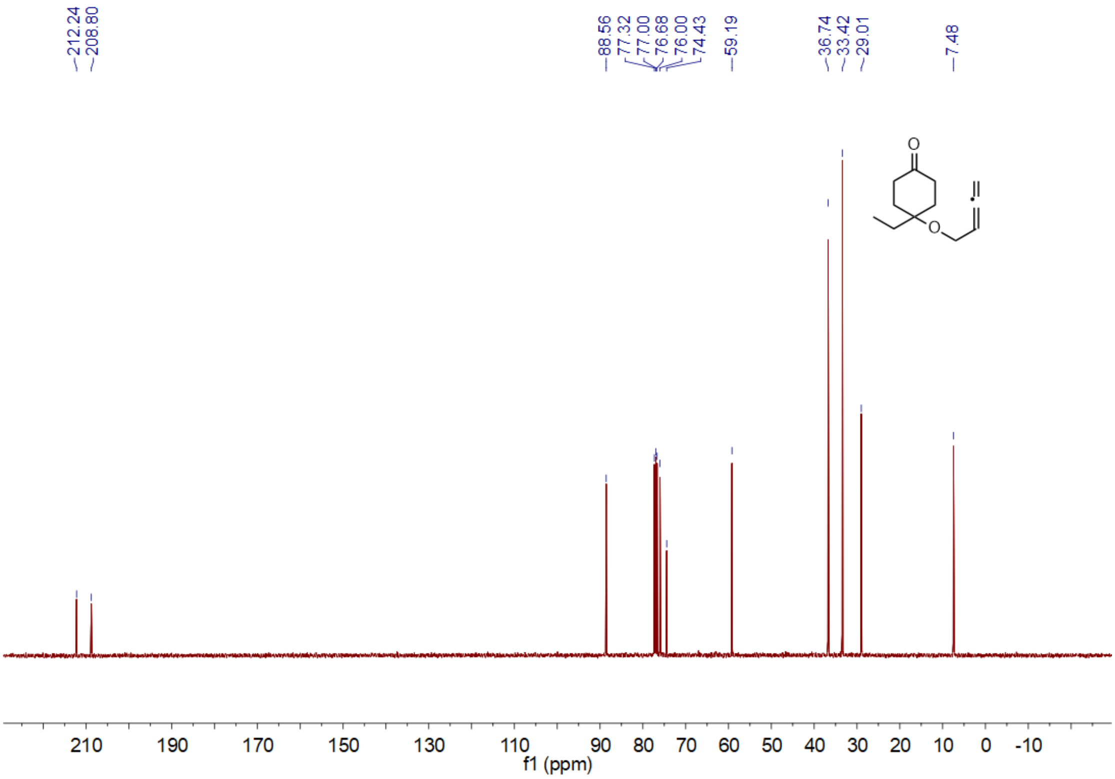
212.24
208.80
88.56
77.32
77.00
76.68
76.00
74.43
59.19
36.74
33.42
29.01
7.48
O
O
210 190 170 150 130 110 90 80 70 60 50 40 30 20 10 0 -10
f1 (ppm)

3j

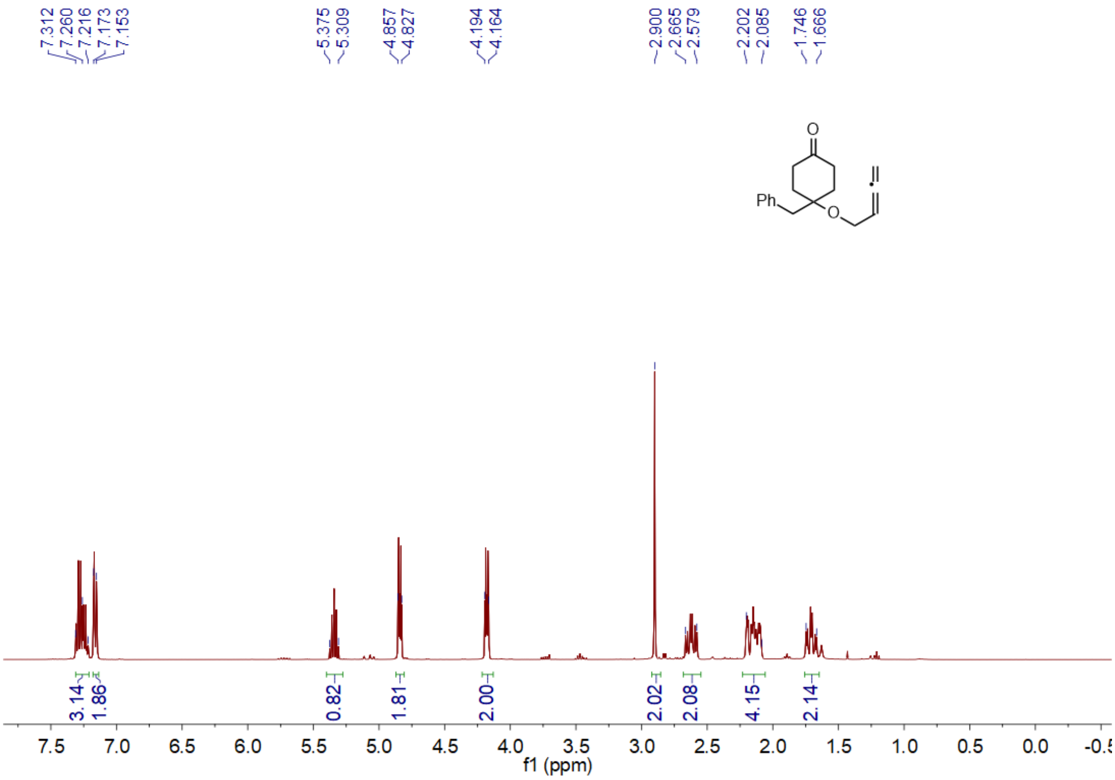
7.312
7.260
7.216
7.173
7.153
5.375
5.309
4.857
4.827
4.194
4.164
2.900
2.665
2.579
2.202
2.085
1.746
1.666
O
Ph
O
3.14
1.86
0.82
1.81
2.00
2.02
2.08
4.15
2.14
7.5 7.0 6.5 6.0 5.5 5.0 4.5 4.0 3.5 3.0 2.5 2.0 1.5 1.0 0.5 0.0
f1 (ppm)

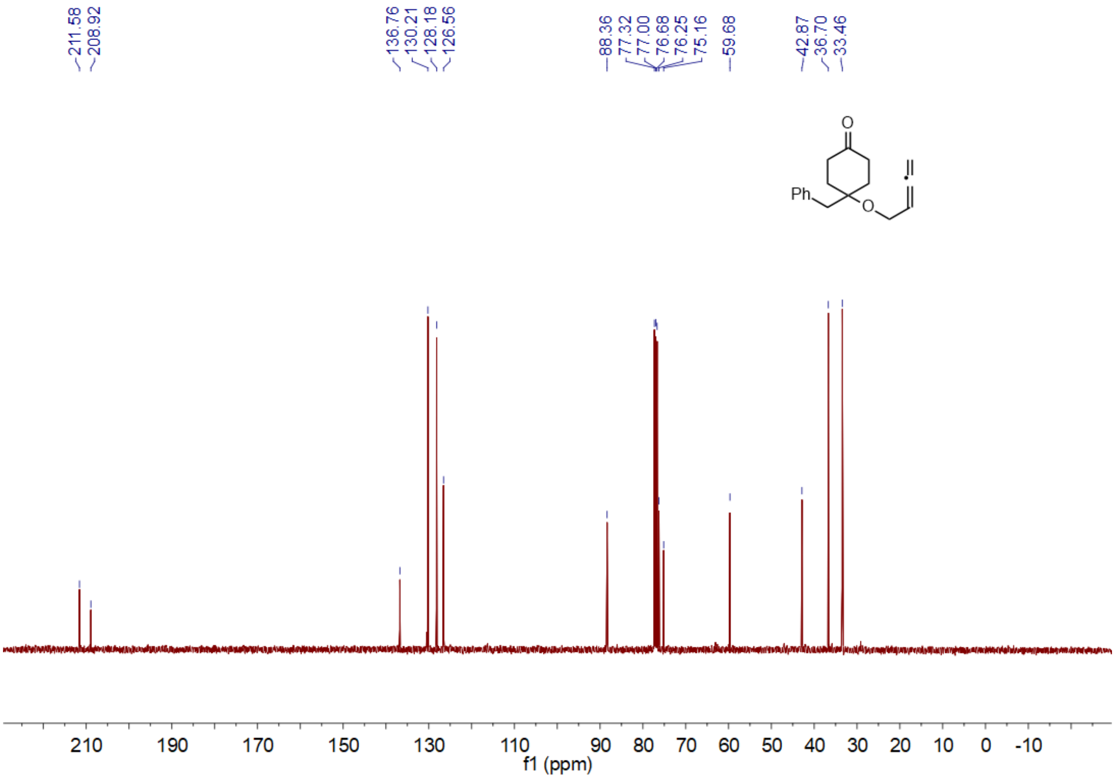
211.58
208.92
136.76
130.21
128.18
126.56
88.36
77.32
77.00
76.68
76.25
75.16
59.68
42.87
36.70
33.46
O
Ph
O
210 190 170 150 130 110 90 80 70 60 50 40 30 20 10 0 -10
f1 (ppm)

3k

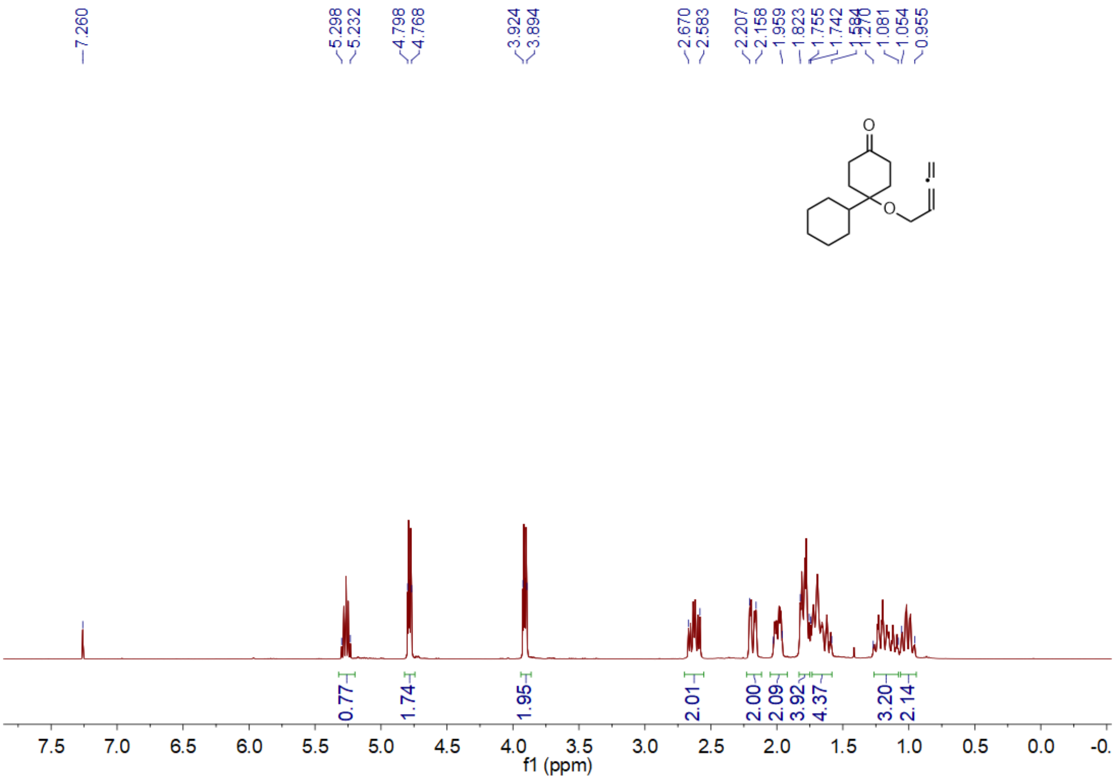

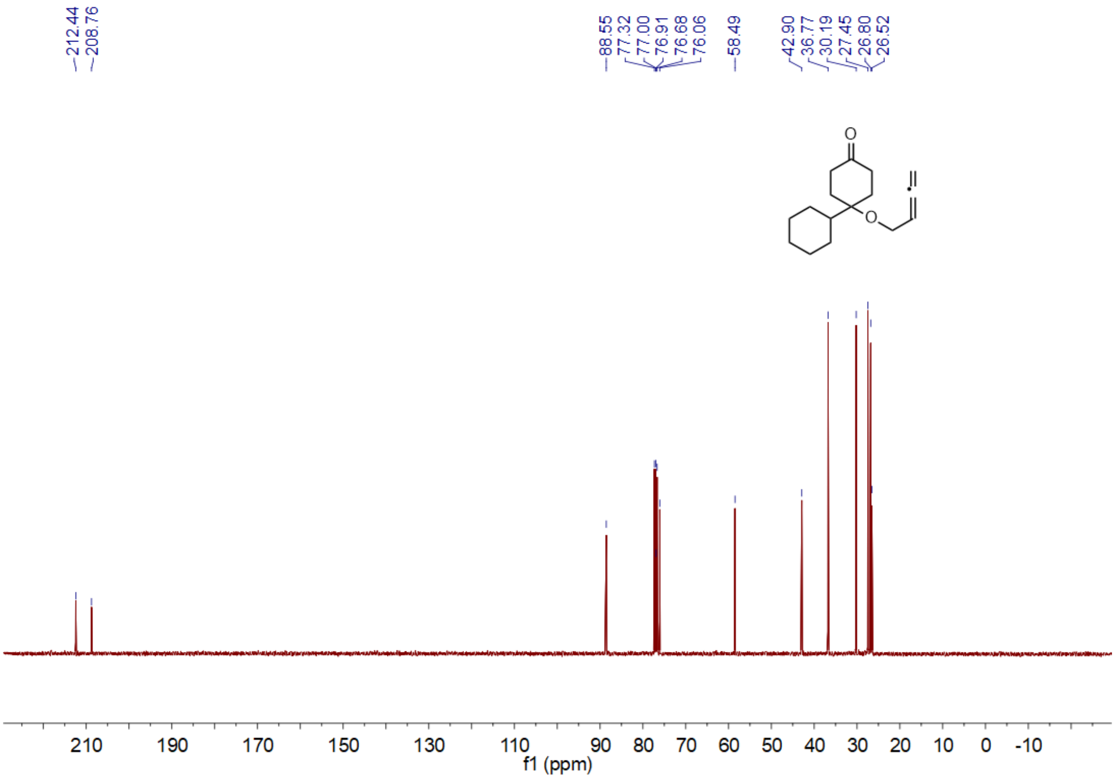

31

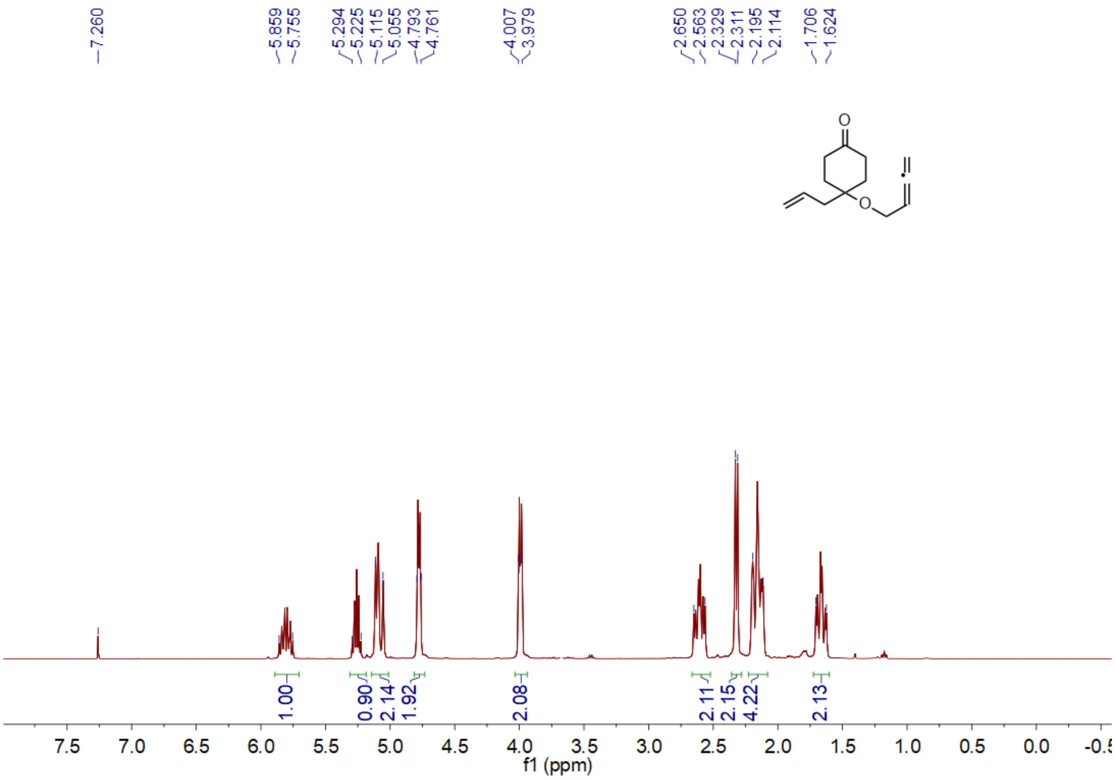
7.260
5.859
5.755
5.294
5.225
5.115
5.055
4.793
4.761
4.007
3.979
2.650
2.563
2.329
2.311
2.195
2.114
1.706
1.624
O
O
1.00
0.90
2.14
1.92
2.08
2.11
2.15
4.22
2.13
f1 (ppm)

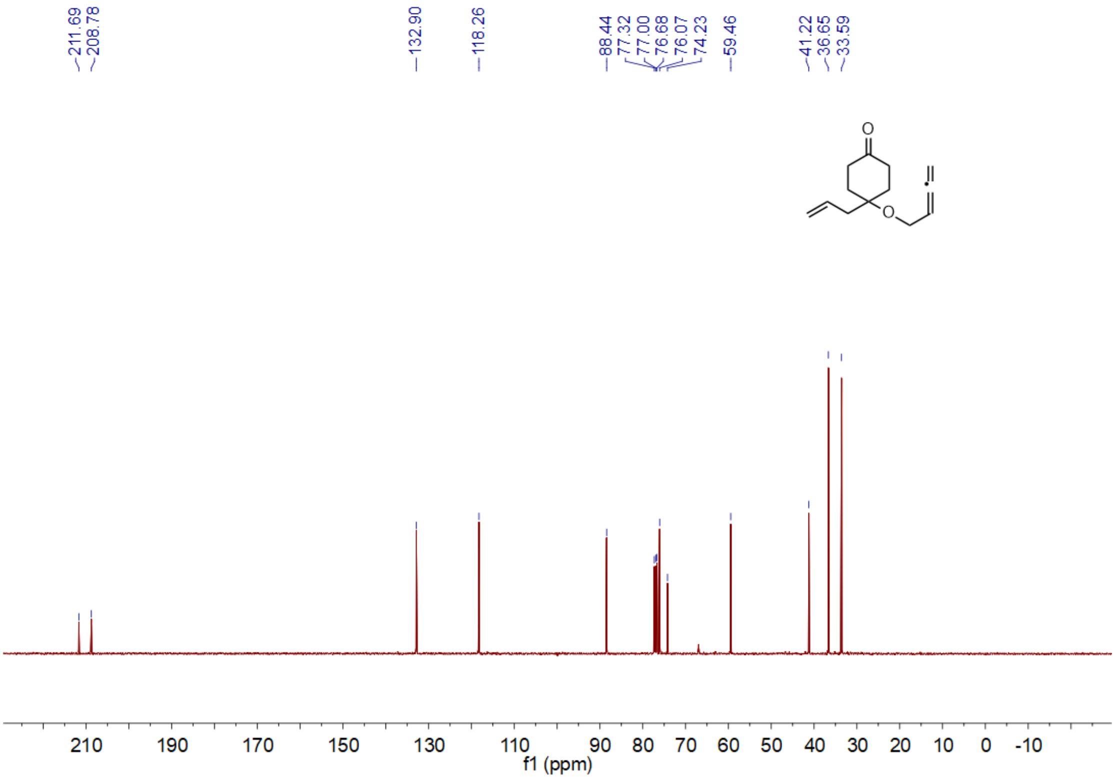
211.69
208.78
132.90
118.26
88.44
77.32
77.00
76.68
76.07
74.23
59.46
41.22
36.65
33.59
O
O
f1 (ppm)

S5a

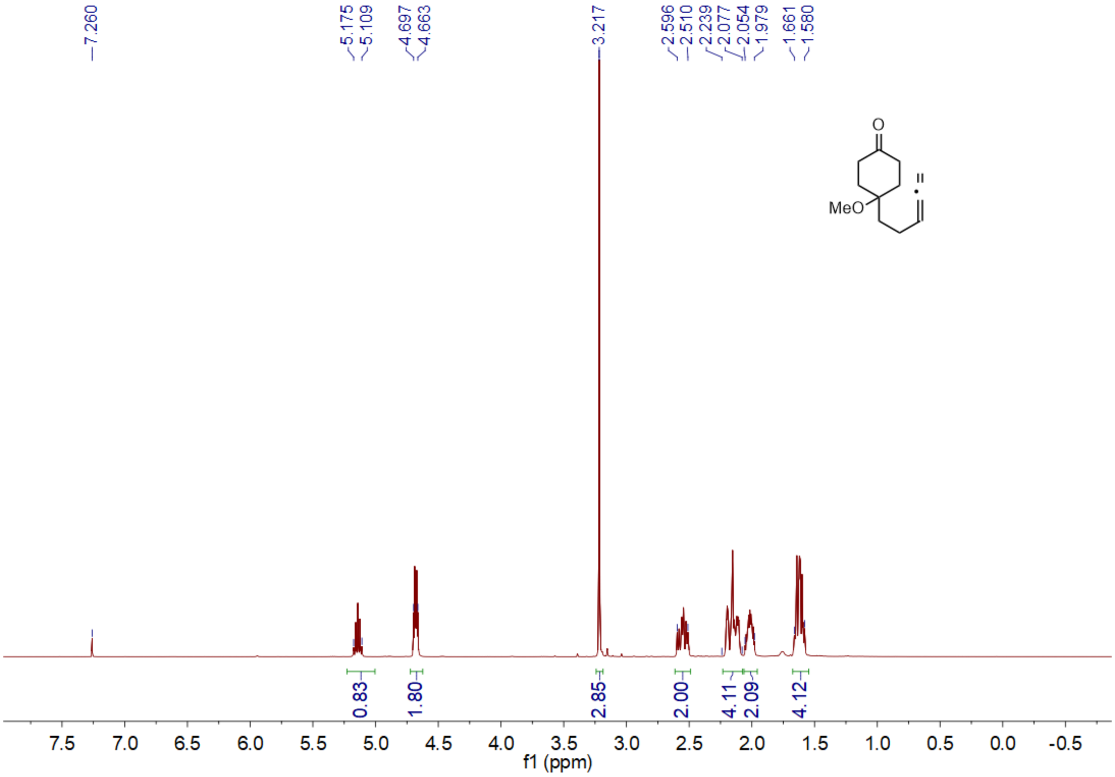
7.260
5.175
5.109
4.697
4.663
3.217
2.596
2.510
2.239
2.077
2.054
1.979
1.661
1.580
O
MeO
0.83
1.80
2.85
2.00
4.11
2.09
4.12
7.5 7.0 6.5 6.0 5.5 5.0 4.5 4.0 3.5 3.0 2.5 2.0 1.5 1.0 0.5 0.0 -0.5
f1 (ppm)

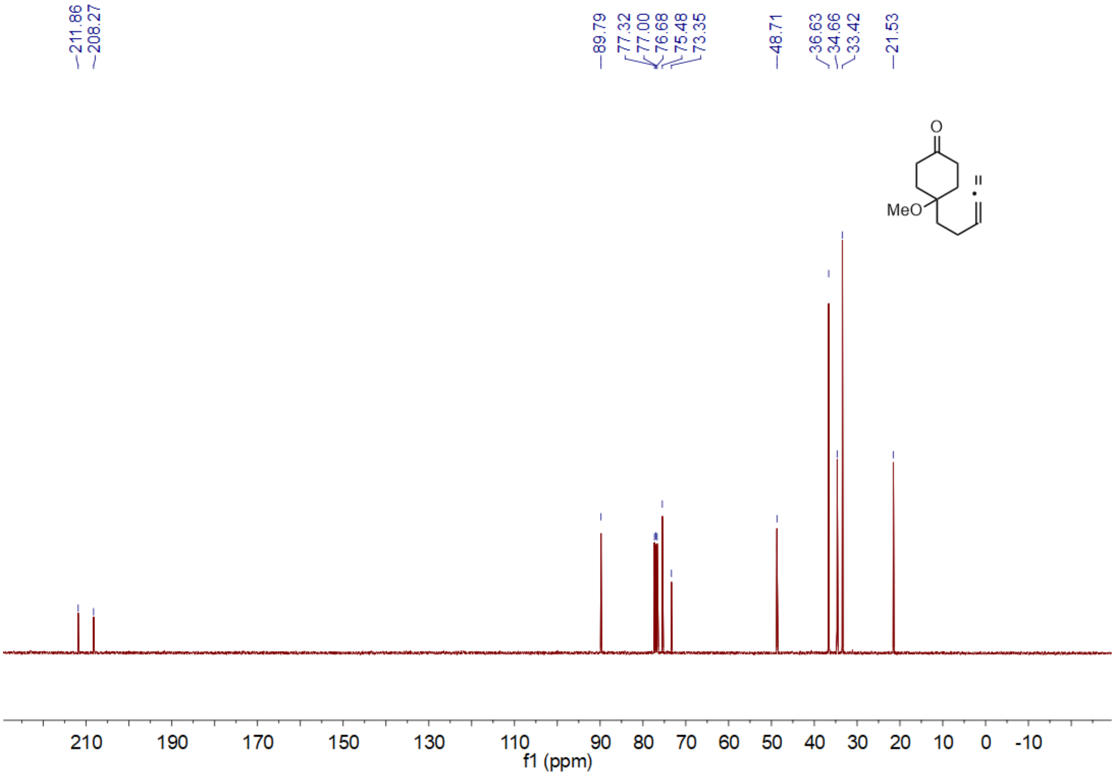
211.86
208.27
89.79
77.32
77.00
76.68
75.48
73.35
48.71
36.63
34.66
33.42
21.53
O
MeO
210 190 170 150 130 110 90 80 70 60 50 40 30 20 10 0 -10
f1 (ppm)

5a

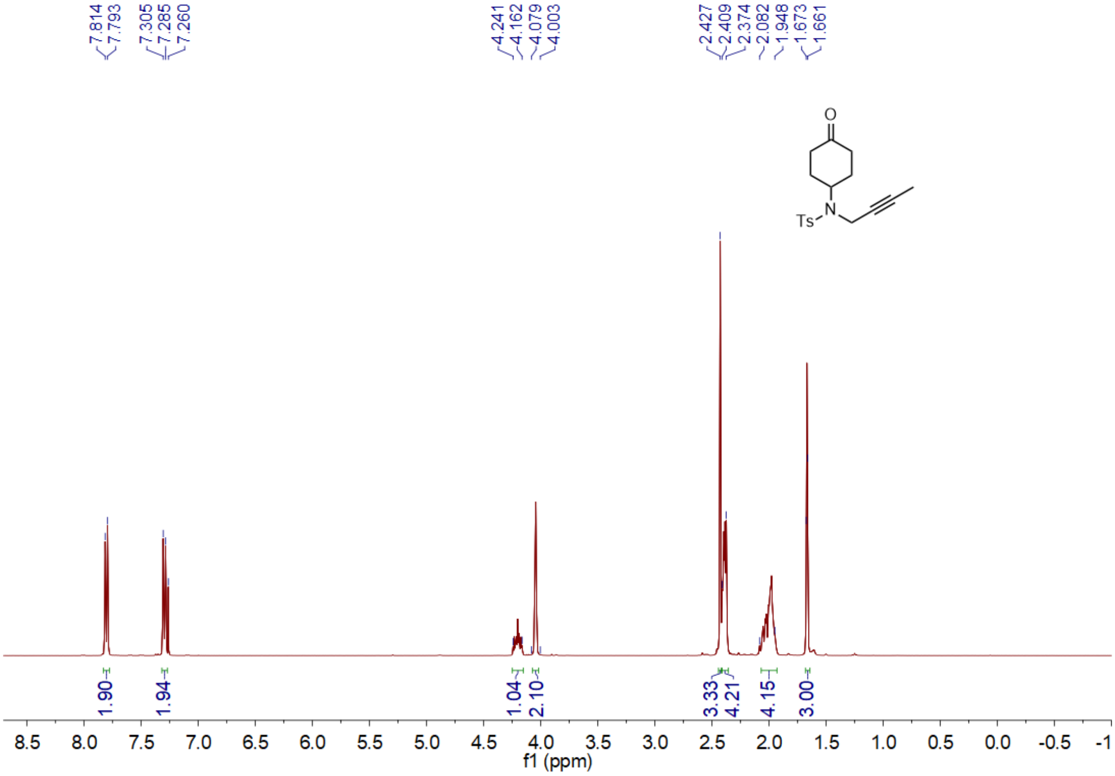
7.814
7.793
7.305
7.285
7.260
4.241
4.162
4.079
4.003
2.427
2.409
2.374
2.082
1.948
1.673
1.661
O
Ts
N
1.90
1.94
1.04
2.10
3.33
4.21
4.15
3.00
8.5 8.0 7.5 7.0 6.5 6.0 5.5 5.0 4.5 4.0 3.5 3.0 2.5 2.0 1.5 1.0 0.5 0.0 -0.5 -1
f1 (ppm)

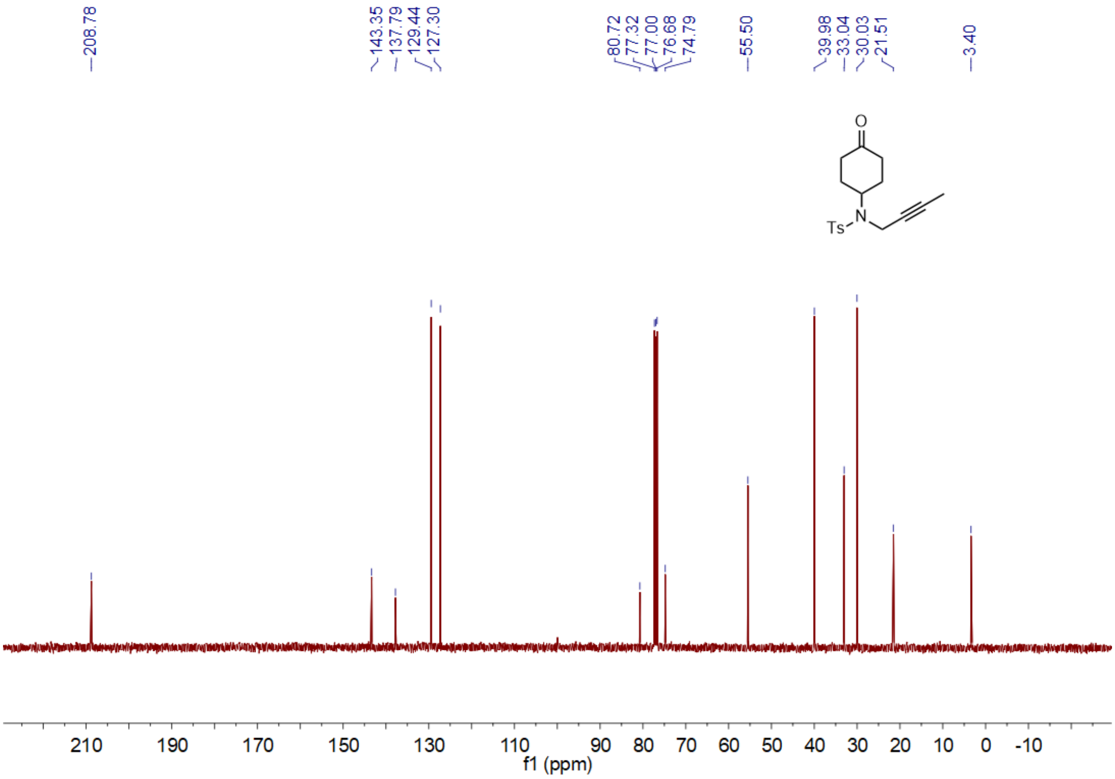
208.78
143.35
137.79
129.44
127.30
80.72
77.32
77.00
76.68
74.79
55.50
39.98
33.04
30.03
21.51
3.40
O
Ts
N
210 190 170 150 130 110 90 80 70 60 50 40 30 20 10 0 -10
f1 (ppm)

9.3: Spectra of products 2 and 4

2a

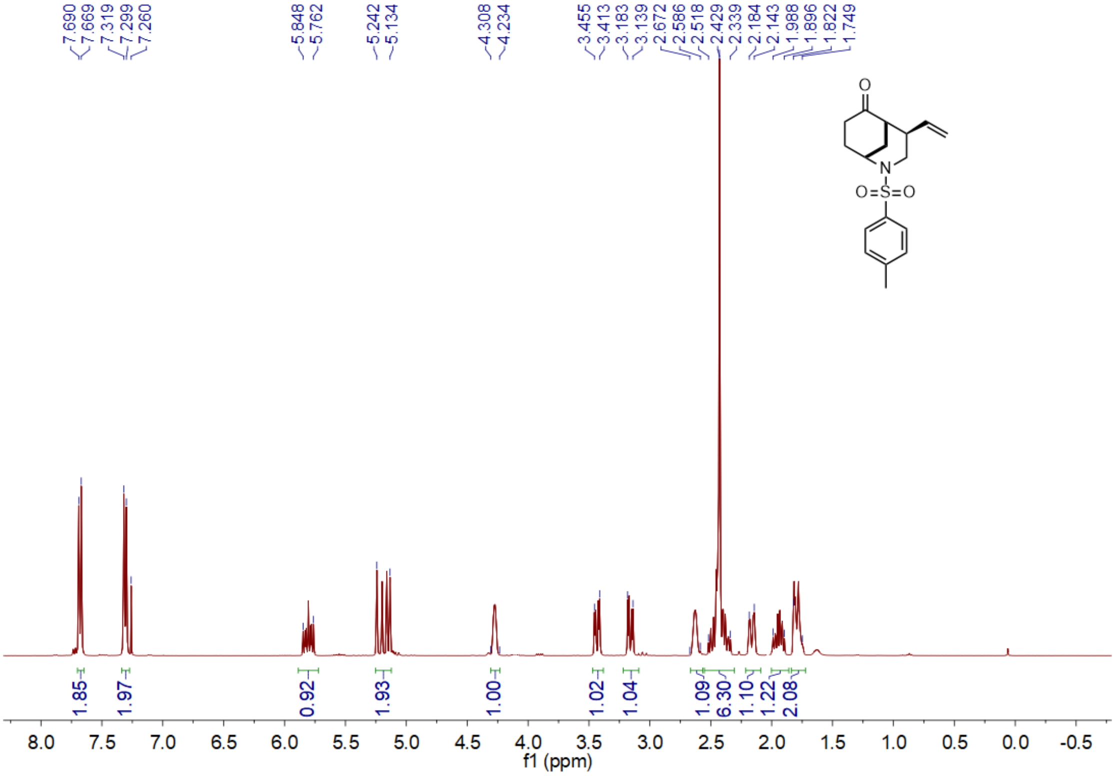

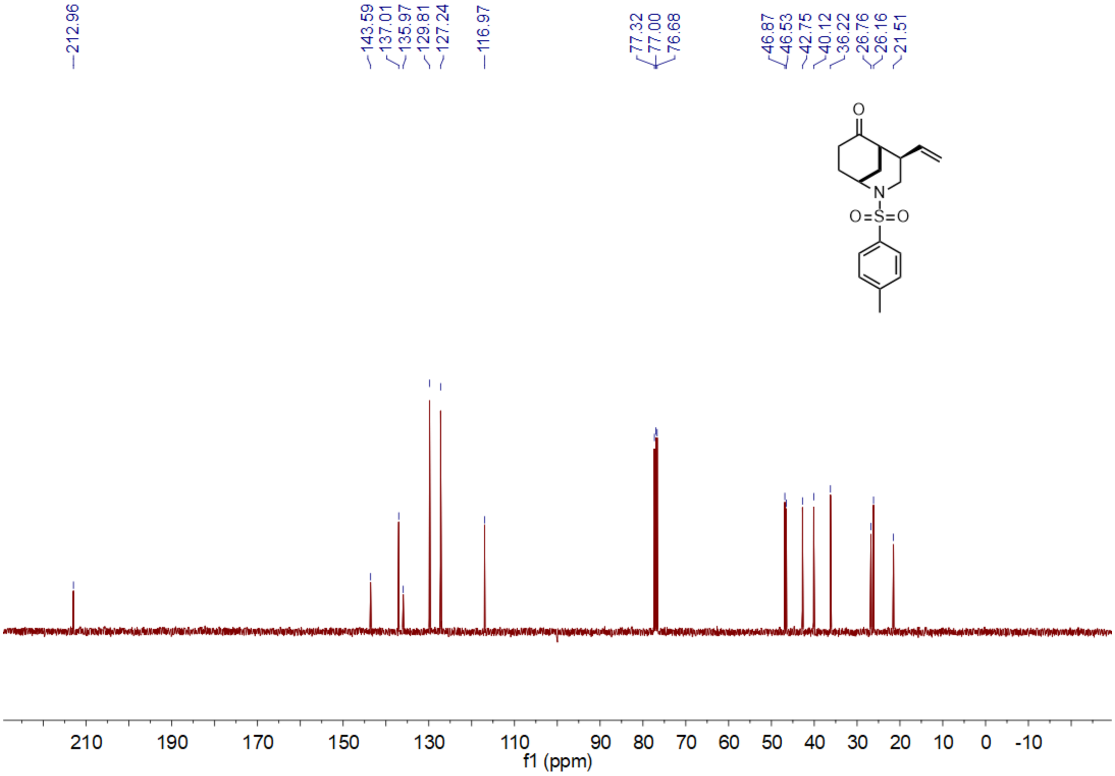

2b

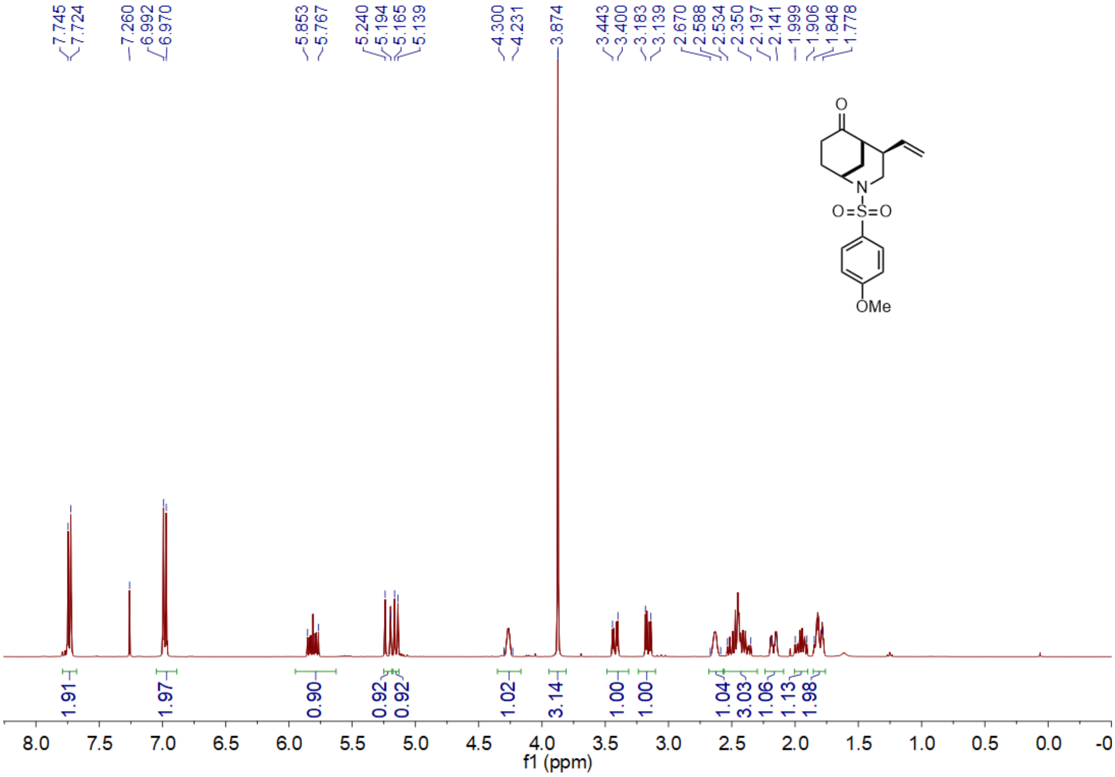
7.745
7.724
7.260
6.992
6.970
5.853
5.767
5.240
5.194
5.165
5.139
4.300
4.231
3.874
3.443
3.400
3.183
3.139
2.670
2.588
2.534
2.350
2.197
2.141
1.999
1.906
1.848
1.778
O
N
O=S=O
OMe
1.91
1.97
0.90
0.92
0.92
1.02
3.14
1.00
1.00
1.04
3.03
1.06
1.13
1.98
8.0 7.5 7.0 6.5 6.0 5.5 5.0 4.5 4.0 3.5 3.0 2.5 2.0 1.5 1.0 0.5 0.0 -0
f1 (ppm)

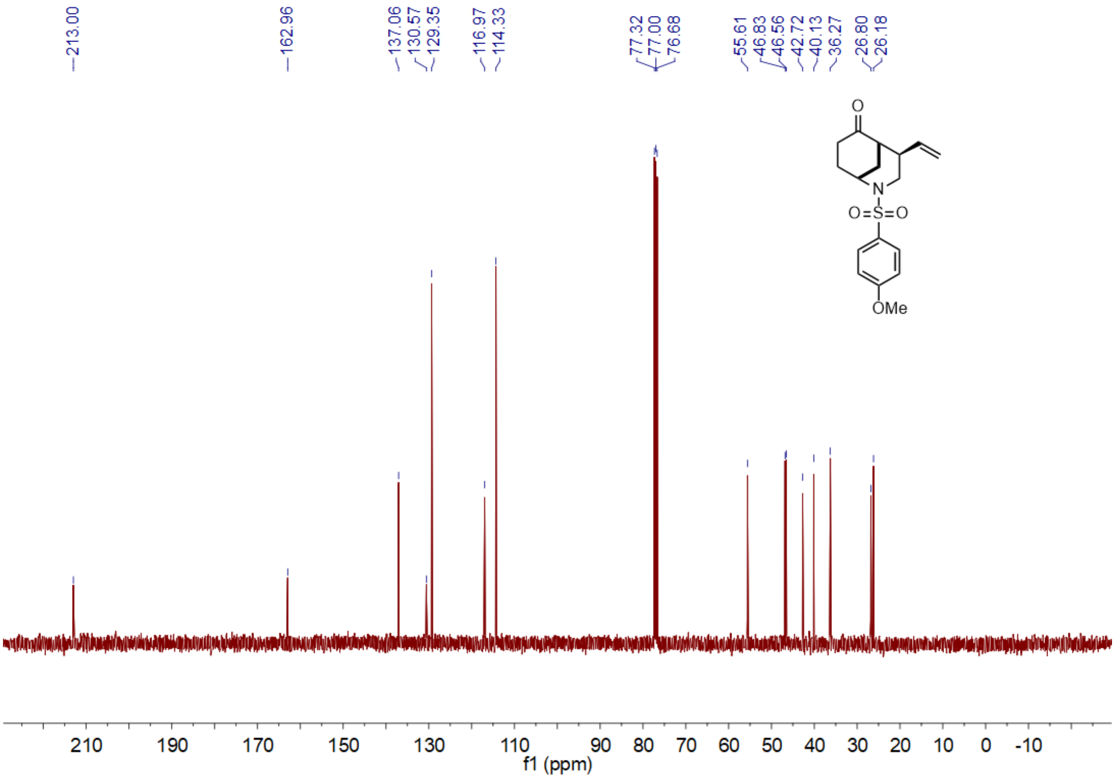
213.00
162.96
137.06
130.57
129.35
116.97
114.33
77.32
77.00
76.68
55.61
46.83
46.56
42.72
40.13
36.27
26.80
26.18
O
N
O=S=O
OMe
210 190 170 150 130 110 90 80 70 60 50 40 30 20 10 0 -10
f1 (ppm)

2c

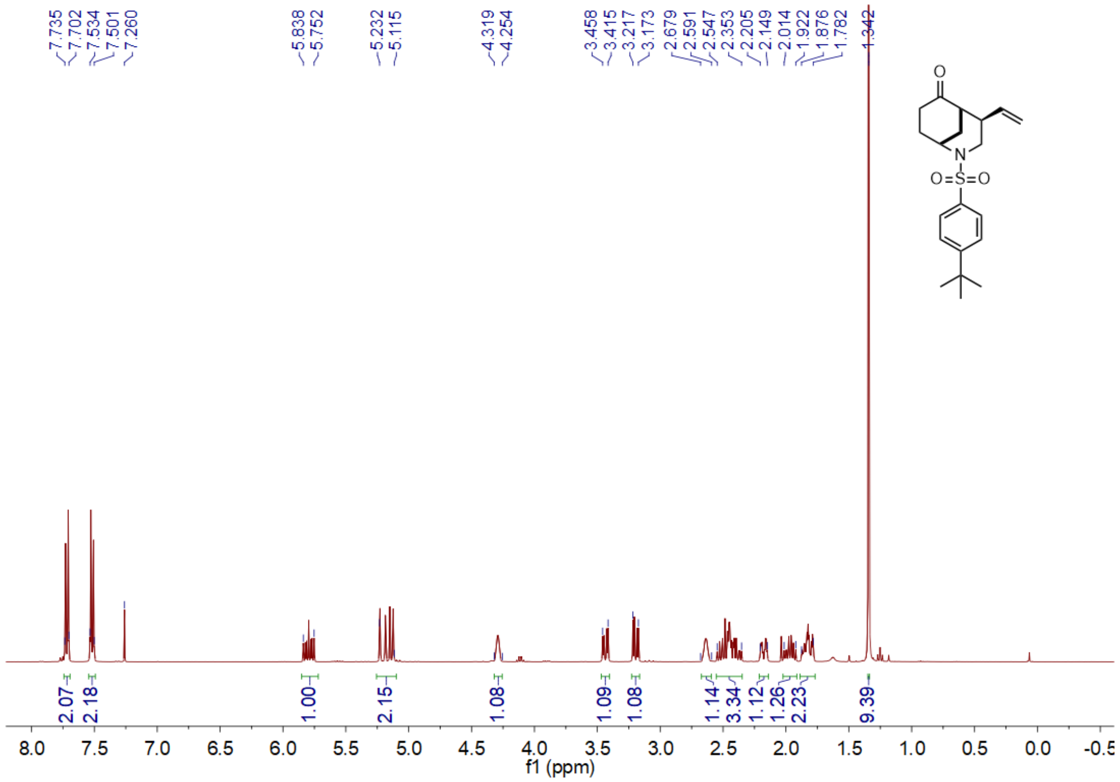

7.735
7.702
7.534
7.501
7.260
5.838
5.752
5.232
5.115
4.319
4.254
3.458
3.415
3.217
3.173
2.679
2.591
2.547
2.353
2.205
2.149
2.014
1.922
1.876
1.782
1.342
O
N
O=S=O
2.07
2.18
1.00
2.15
1.08
1.09
1.08
1.14
3.34
1.12
1.26
2.23
9.39
8.0 7.5 7.0 6.5 6.0 5.5 5.0 4.5 4.0 3.5 3.0 2.5 2.0 1.5 1.0 0.5 0.0 -0.5
f1 (ppm)

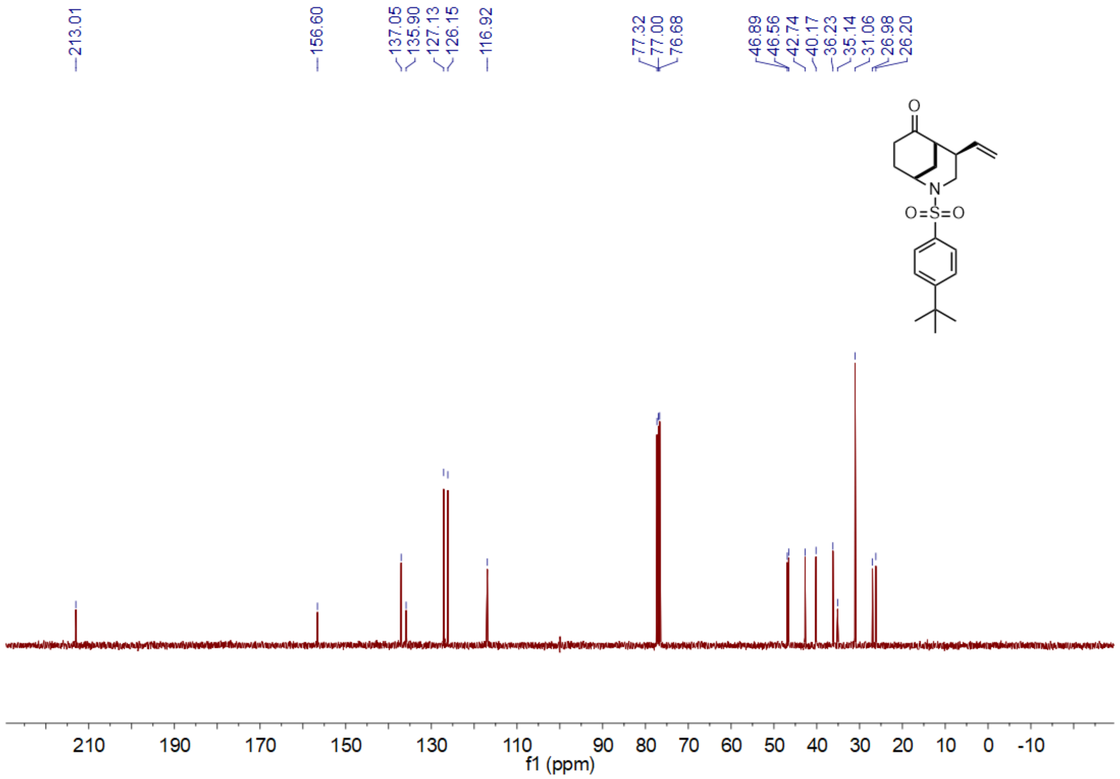

213.01
156.60
137.05
135.90
127.13
126.15
116.92
77.32
77.00
76.68
46.89
46.56
42.74
40.17
36.23
35.14
31.06
26.98
26.20
O
N
O=S=O
210 190 170 150 130 110 90 80 70 60 50 40 30 20 10 0 -10
f1 (ppm)

2d

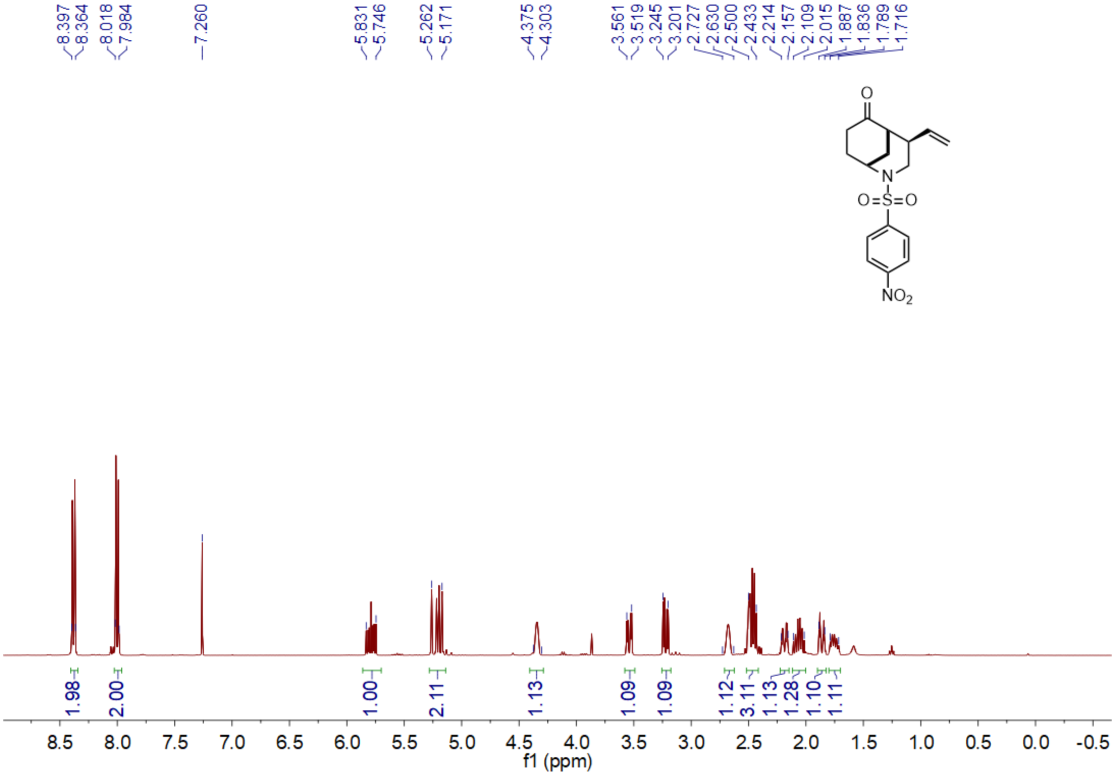
8.397
8.364
8.018
7.984
7.260
5.831
5.746
5.262
5.171
4.375
4.303
3.561
3.519
3.245
3.201
2.727
2.630
2.500
2.433
2.214
2.157
2.109
2.015
1.887
1.836
1.789
1.716
O
N
O=S=O
NO2
1.98
2.00
1.00
2.11
1.13
1.09
1.09
1.12
3.11
1.13
1.28
1.10
1.11
8.5 8.0 7.5 7.0 6.5 6.0 5.5 5.0 4.5 4.0 3.5 3.0 2.5 2.0 1.5 1.0 0.5 0.0 -0.5
f1 (ppm)

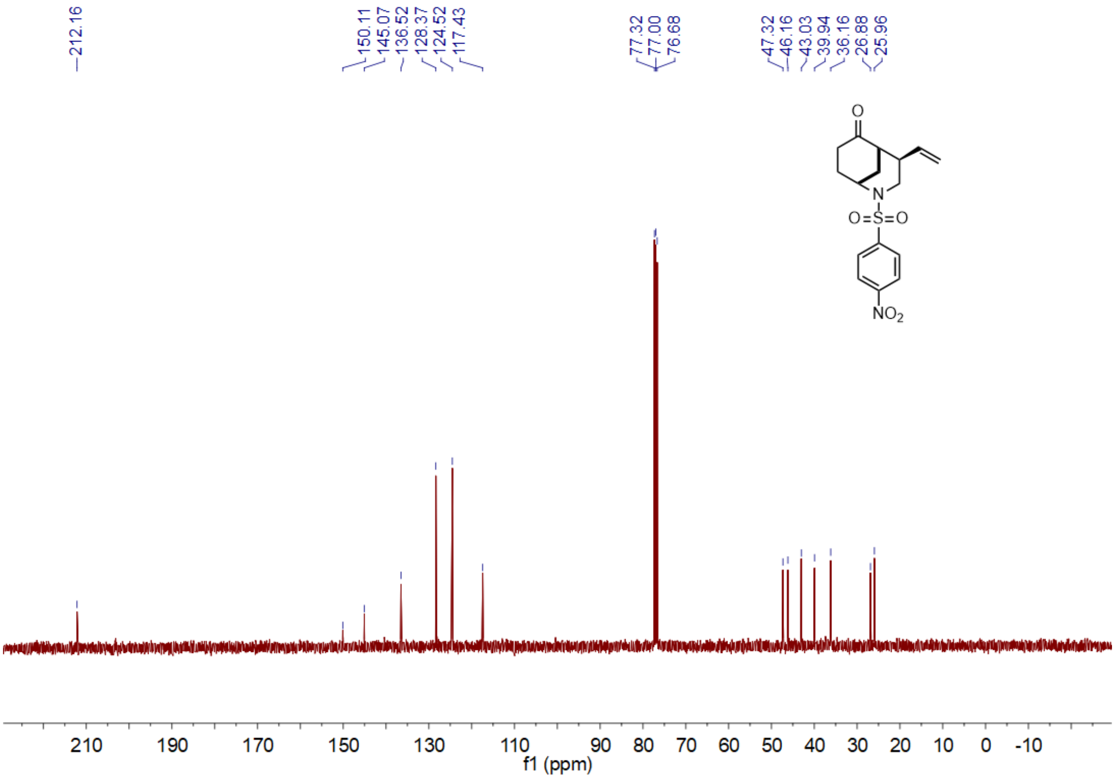
212.16
150.11
145.07
136.52
128.37
124.52
117.43
77.32
77.00
76.68
47.32
46.16
43.03
39.94
36.16
26.88
25.96
O
N
O=S=O
NO2
210 190 170 150 130 110 90 80 70 60 50 40 30 20 10 0 -10
f1 (ppm)

2e

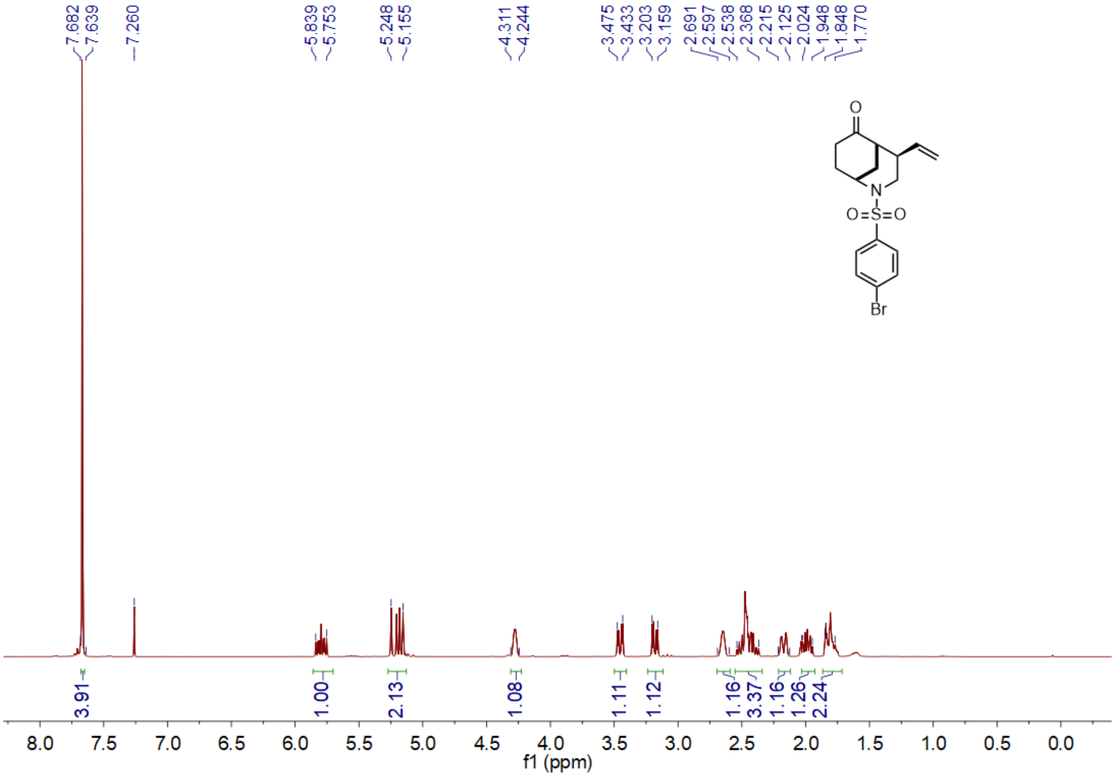

7.682
7.639
7.260
5.839
5.753
5.248
5.155
4.311
4.244
3.475
3.433
3.203
3.159
2.691
2.597
2.538
2.368
2.215
2.125
2.024
1.948
1.848
1.770
O
N
O=S=O
Br
3.91
1.00
2.13
1.08
1.11
1.12
1.16
3.37
1.16
1.26
2.24
f1 (ppm)

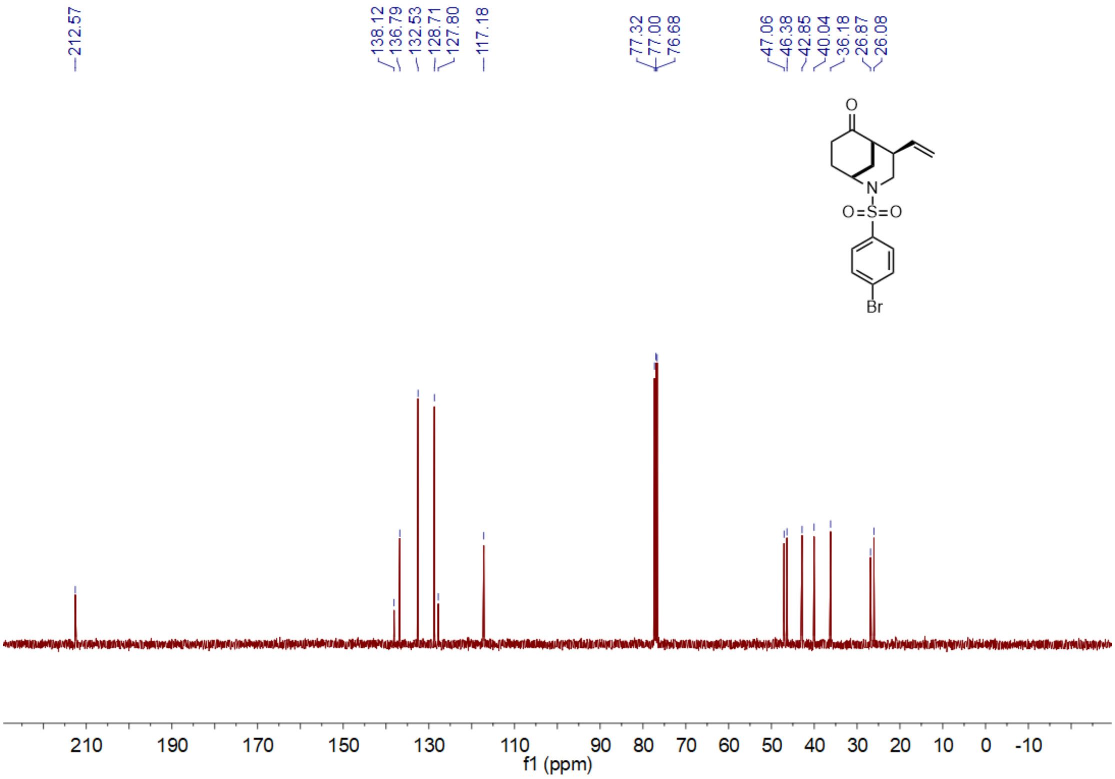

212.57
138.12
136.79
132.53
128.71
127.80
117.18
77.32
77.00
76.68
47.06
46.38
42.85
40.04
36.18
26.87
26.08
O
N
O=S=O
Br
f1 (ppm)

2f

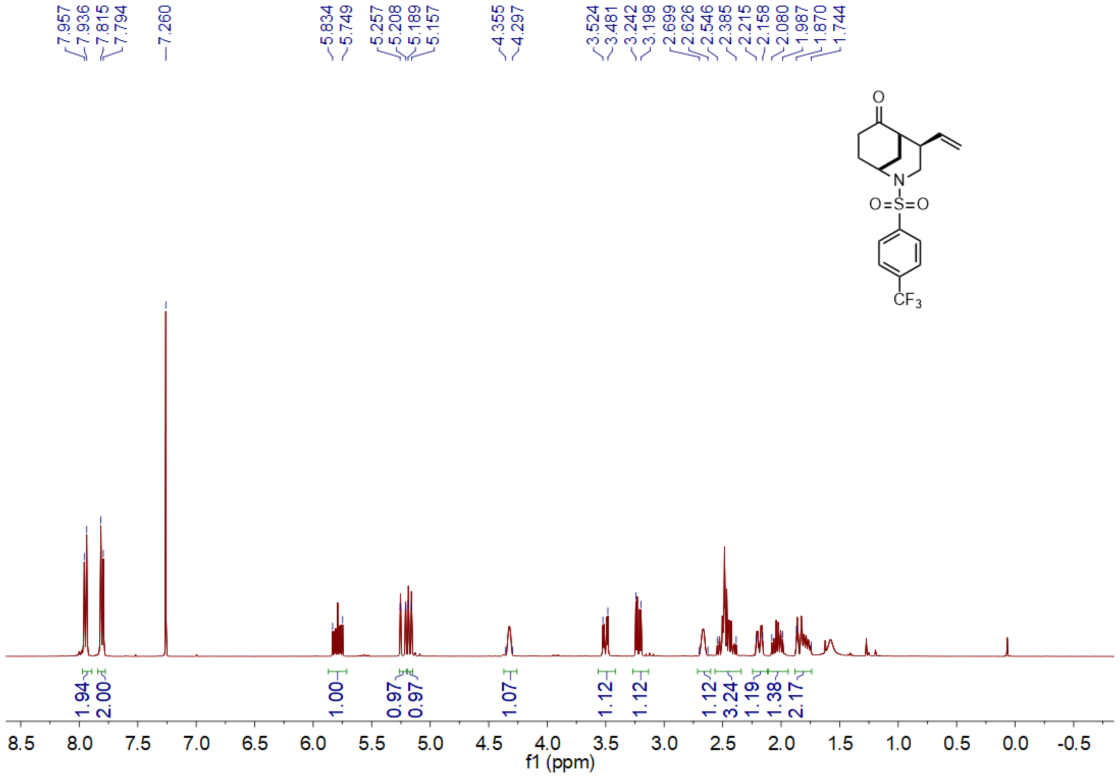
7.957
7.936
7.815
7.794
7.260
5.834
5.749
5.257
5.208
5.189
5.157
4.355
4.297
3.524
3.481
3.242
3.198
2.699
2.626
2.546
2.385
2.215
2.158
2.080
1.987
1.870
1.744
O
N
O=S=O
CF3
1.94
2.00
1.00
0.97
0.97
1.07
1.12
1.12
1.12
3.24
1.19
1.38
2.17
8.5 8.0 7.5 7.0 6.5 6.0 5.5 5.0 4.5 4.0 3.5 3.0 2.5 2.0 1.5 1.0 0.5 0.0 -0.5
f1 (ppm)

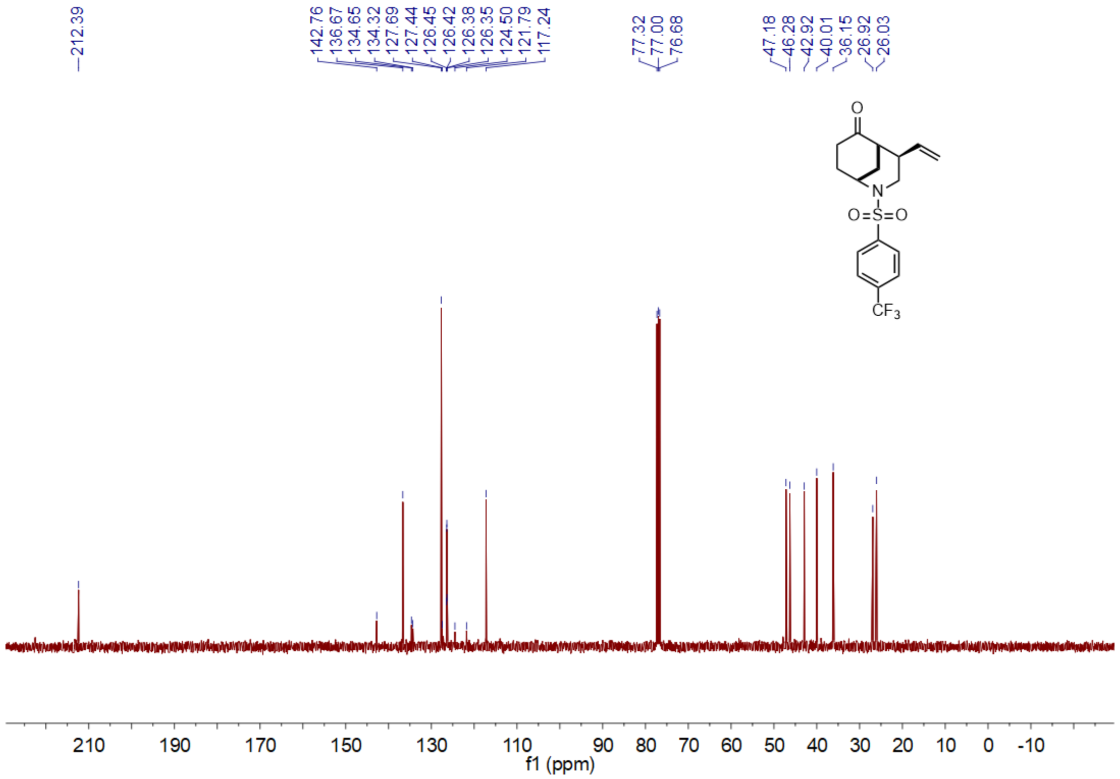
212.39
142.76
136.67
134.65
134.32
127.69
127.44
126.45
126.42
126.38
126.35
124.50
121.79
117.24
77.32
77.00
76.68
47.18
46.28
42.92
40.01
36.15
26.92
26.03
O
N
O=S=O
CF3
210 190 170 150 130 110 90 80 70 60 50 40 30 20 10 0 -10
f1 (ppm)

2g

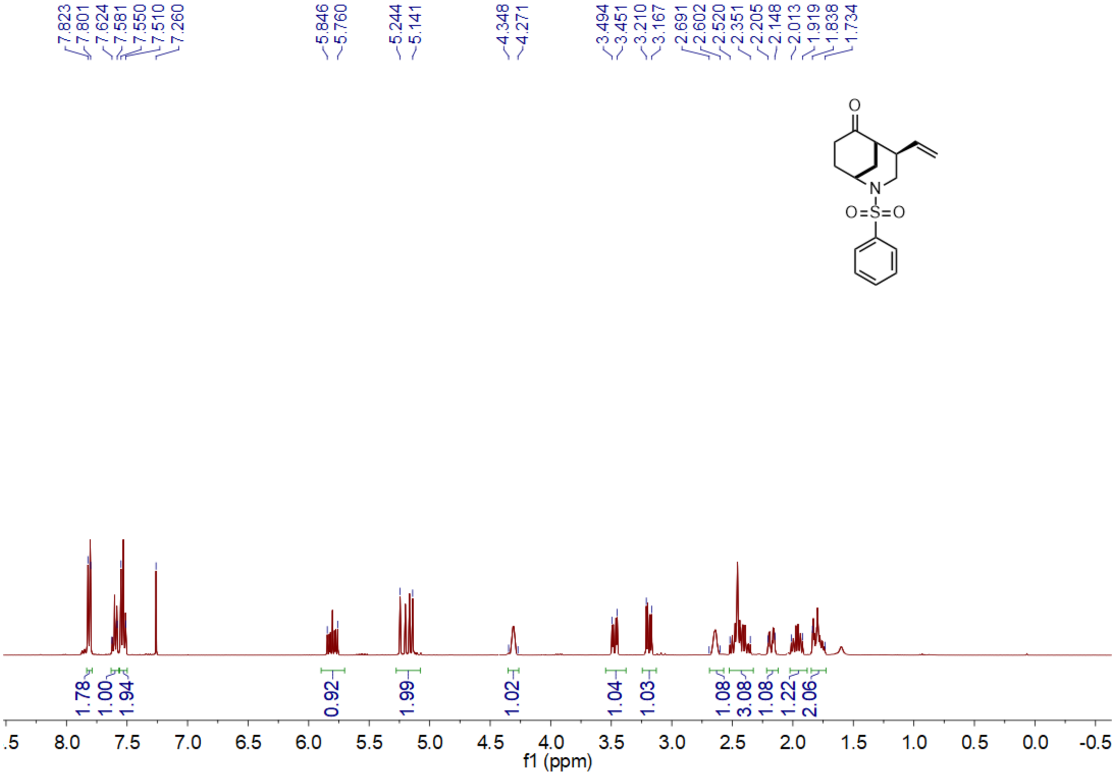
7.823
7.801
7.624
7.581
7.550
7.510
7.260
5.846
5.760
5.244
5.141
4.348
4.271
3.494
3.451
3.210
3.167
2.691
2.602
2.520
2.351
2.205
2.148
2.013
1.919
1.838
1.734
O
N
O=S=O
1.78
1.00
1.94
0.92
1.99
1.02
1.04
1.03
1.08
3.08
1.08
1.22
2.06
.5 8.0 7.5 7.0 6.5 6.0 5.5 5.0 4.5 4.0 3.5 3.0 2.5 2.0 1.5 1.0 0.5 0.0 -0.5
f1 (ppm)

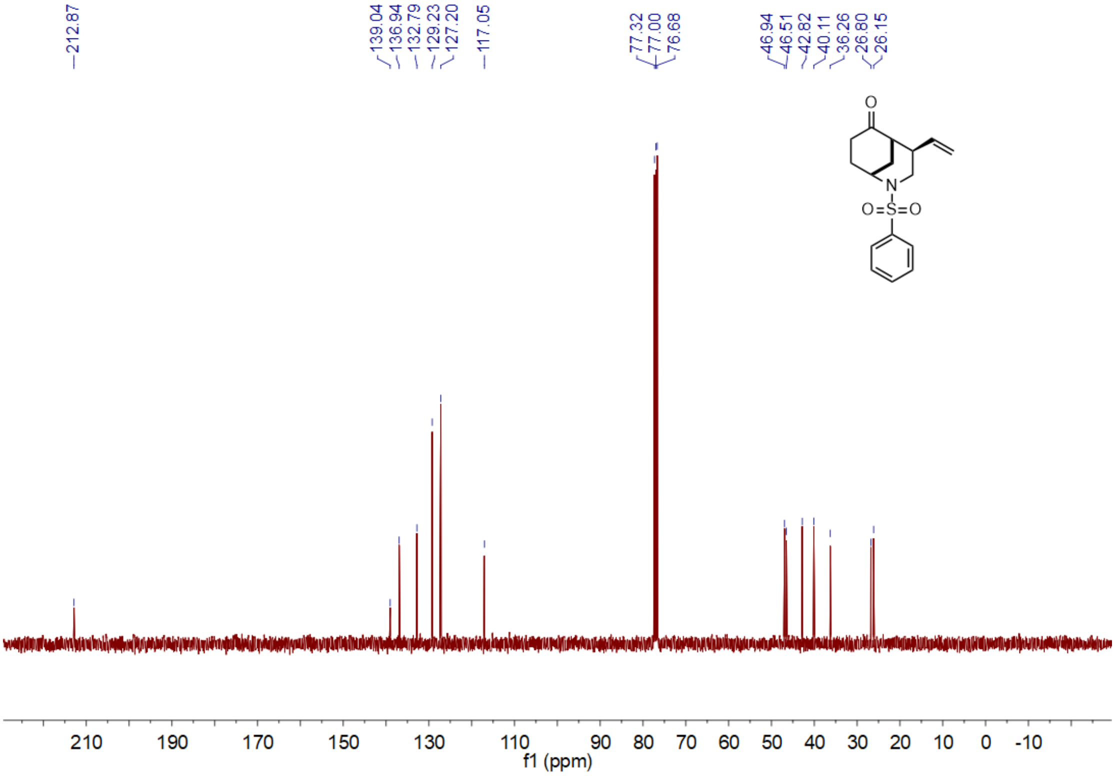
212.87
139.04
136.94
132.79
129.23
127.20
117.05
77.32
77.00
76.68
46.94
46.51
42.82
40.11
36.26
26.80
26.15
O
N
O=S=O
210 190 170 150 130 110 90 80 70 60 50 40 30 20 10 0 -10
f1 (ppm)

2h

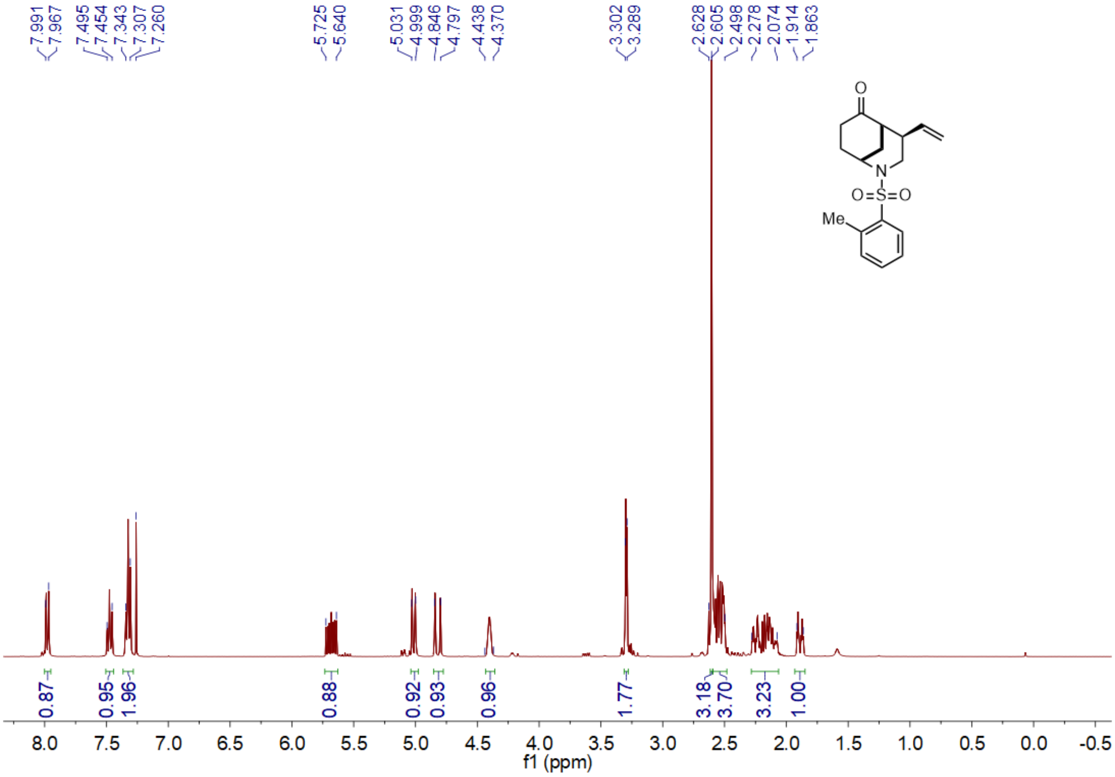
7.991
7.967
7.495
7.454
7.343
7.307
7.260
5.725
5.640
5.031
4.999
4.846
4.797
4.438
4.370
3.302
3.289
2.628
2.605
2.498
2.278
2.074
1.914
1.863
O
N
O=S=O
Me
0.87
0.95
1.96
0.88
0.92
0.93
0.96
1.77
3.18
3.70
3.23
1.00
8.0 7.5 7.0 6.5 6.0 5.5 5.0 4.5 4.0 3.5 3.0 2.5 2.0 1.5 1.0 0.5 0.0 -0.5
f1 (ppm)

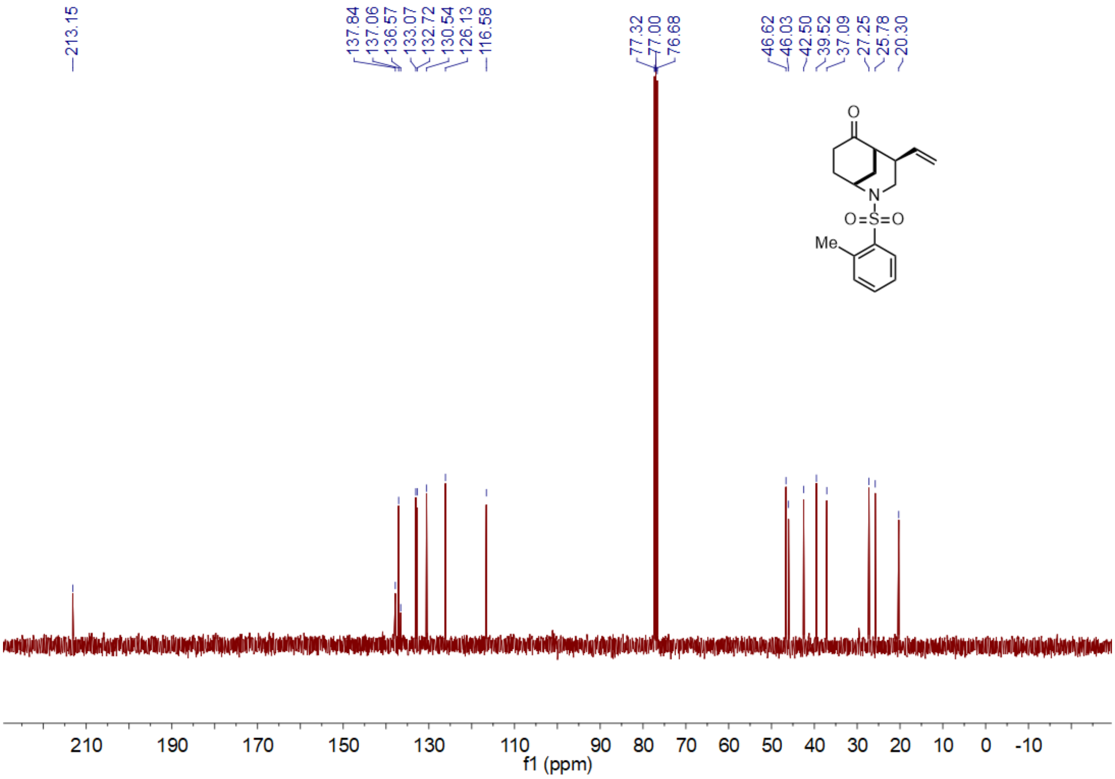
213.15
137.84
137.06
136.57
133.07
132.72
130.54
126.13
116.58
77.32
77.00
76.68
46.62
46.03
42.50
39.52
37.09
27.25
25.78
20.30
O
N
O=S=O
Me
210 190 170 150 130 110 90 80 70 60 50 40 30 20 10 0 -10
f1 (ppm)

2i

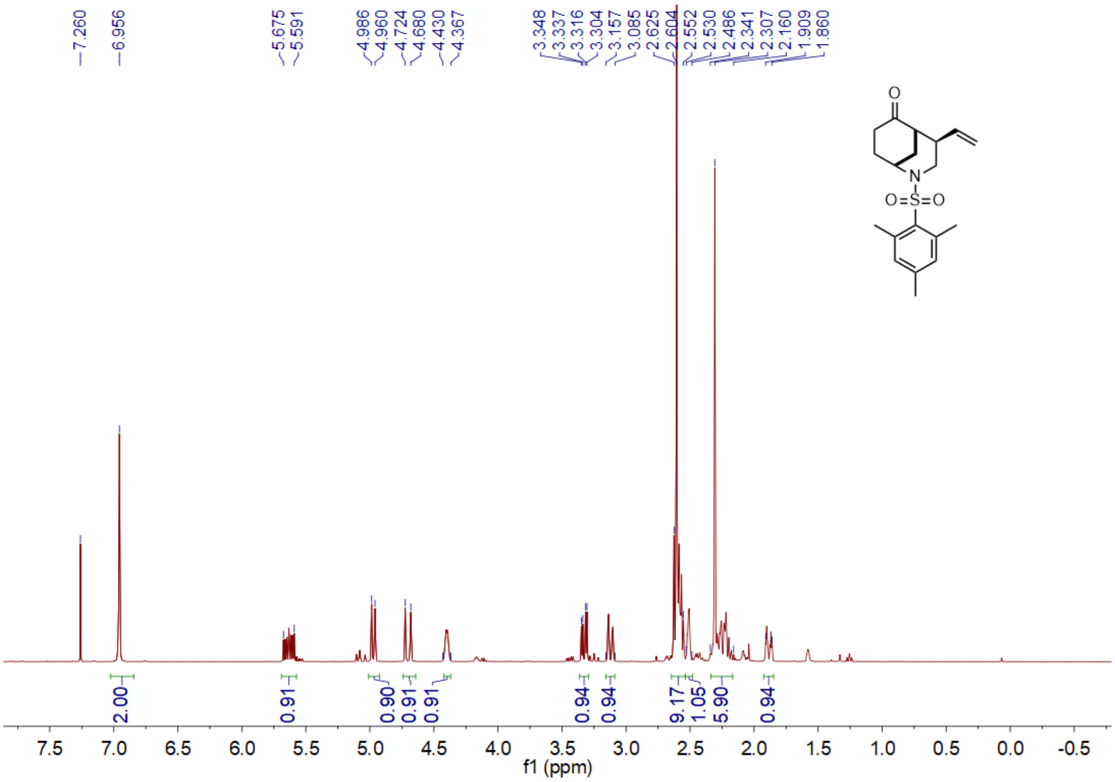
7.260
6.956
5.675
5.591
4.986
4.960
4.724
4.680
4.430
4.367
3.348
3.337
3.316
3.304
3.157
3.085
2.625
2.604
2.552
2.530
2.486
2.341
2.307
2.160
1.909
1.860
2.00
0.91
0.90
0.91
0.91
0.94
0.94
9.17
1.05
5.90
0.94
f1 (ppm)

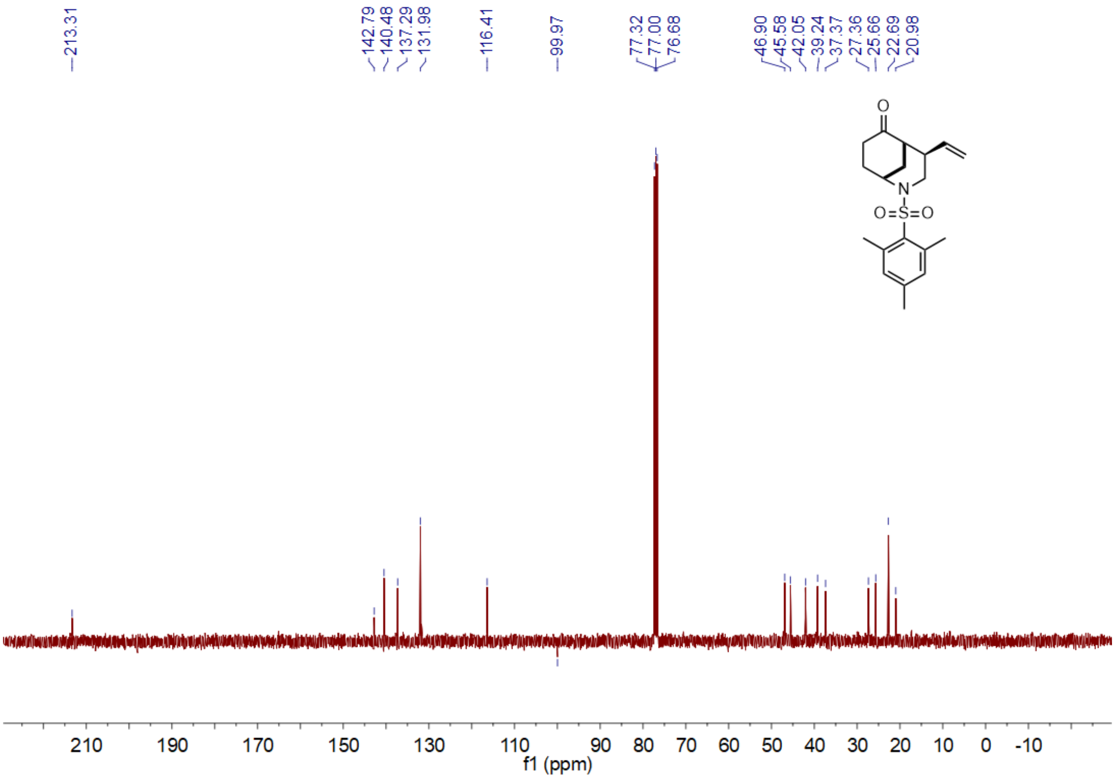
213.31
142.79
140.48
137.29
131.98
116.41
99.97
77.32
77.00
76.68
46.90
45.58
42.05
39.24
37.37
27.36
25.66
22.69
20.98
f1 (ppm)

2j

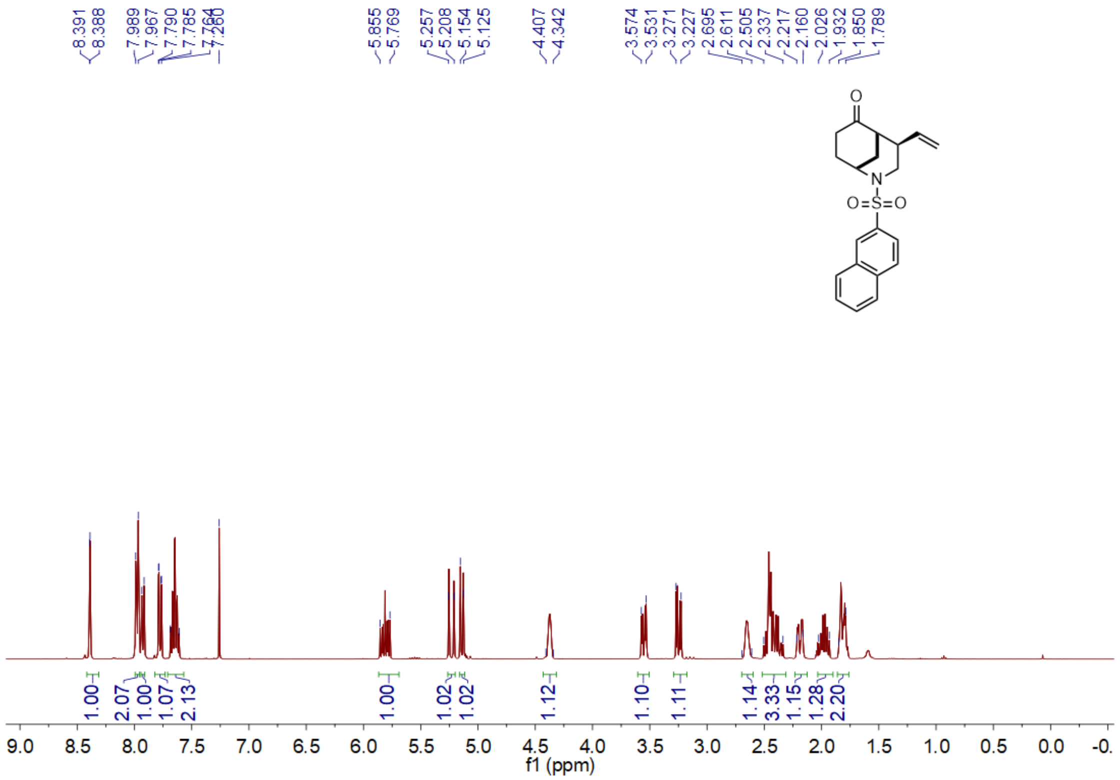
8.391
8.388
7.989
7.967
7.790
7.785
7.764
7.260
5.855
5.769
5.257
5.208
5.154
5.125
4.407
4.342
3.574
3.531
3.271
3.227
2.695
2.611
2.505
2.337
2.217
2.160
2.026
1.932
1.850
1.789
O
N
O=S=O
1.00
2.07
1.00
1.07
2.13
1.00
1.02
1.02
1.12
1.10
1.11
1.14
3.33
1.15
1.28
2.20
9.0
8.5
8.0
7.5
7.0
6.5
6.0
5.5
5.0
4.5
4.0
3.5
3.0
2.5
2.0
1.5
1.0
0.5
0.0
f1 (ppm)

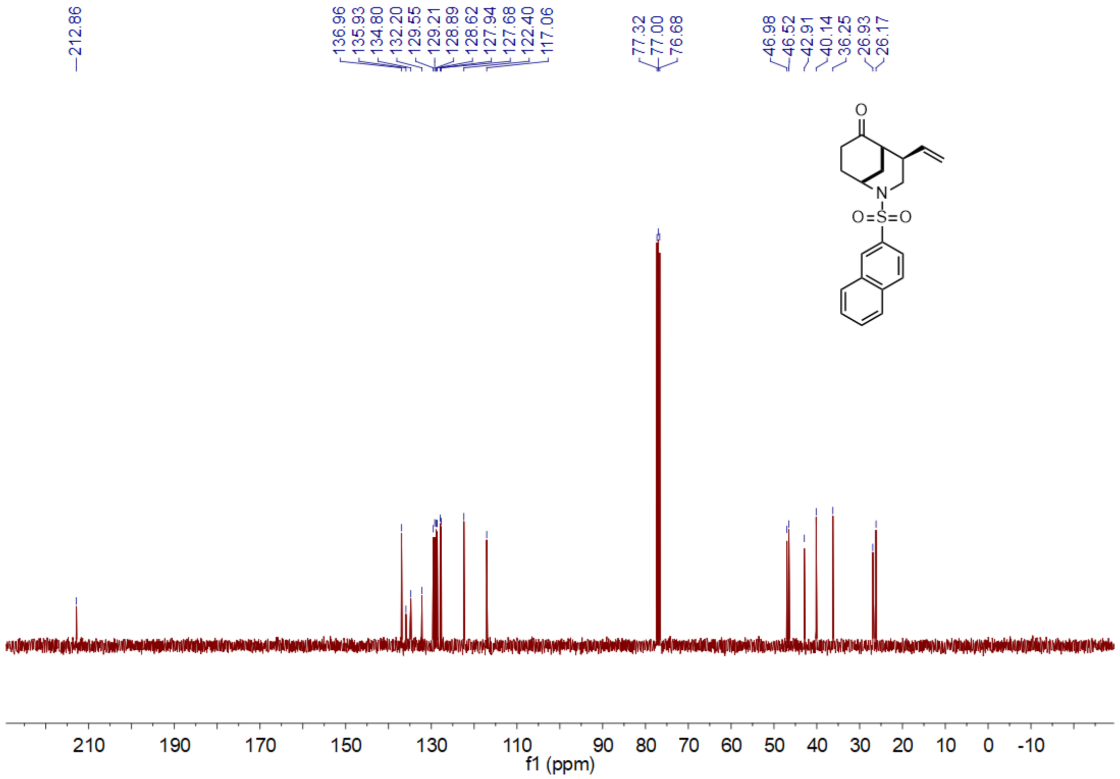
212.86
136.96
135.93
134.80
132.20
129.55
129.21
128.89
128.62
127.94
127.68
122.40
117.06
77.32
77.00
76.68
46.98
46.52
42.91
40.14
36.25
26.93
26.17
O
N
O=S=O
210
190
170
150
130
110
90
80
70
60
50
40
30
20
10
0
-10
f1 (ppm)

2k

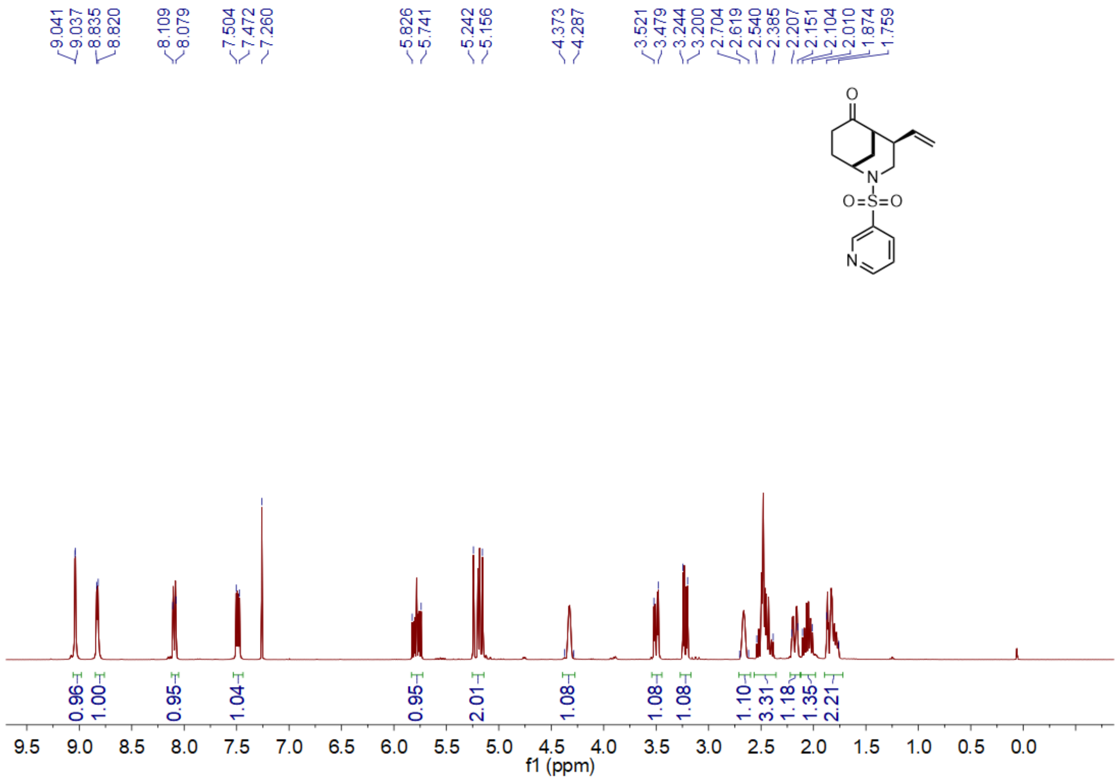
9.041
9.037
8.835
8.820
8.109
8.079
7.504
7.472
7.260
5.826
5.741
5.242
5.156
4.373
4.287
3.521
3.479
3.244
3.200
2.704
2.619
2.540
2.385
2.207
2.151
2.104
2.010
1.874
1.759
0.96
1.00
0.95
1.04
0.95
2.01
1.08
1.08
1.08
1.10
3.31
1.18
1.35
2.21
f1 (ppm)

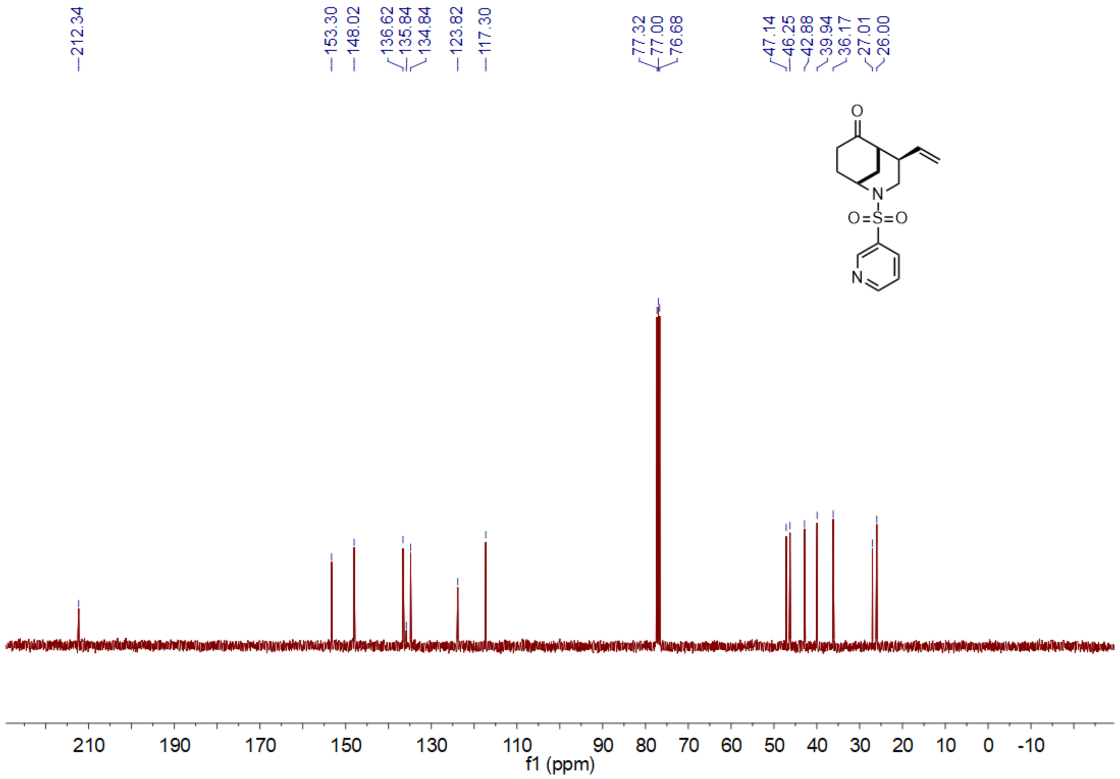
212.34
153.30
148.02
136.62
135.84
134.84
123.82
117.30
77.32
77.00
76.68
47.14
46.25
42.88
39.94
36.17
27.01
26.00
f1 (ppm)

21

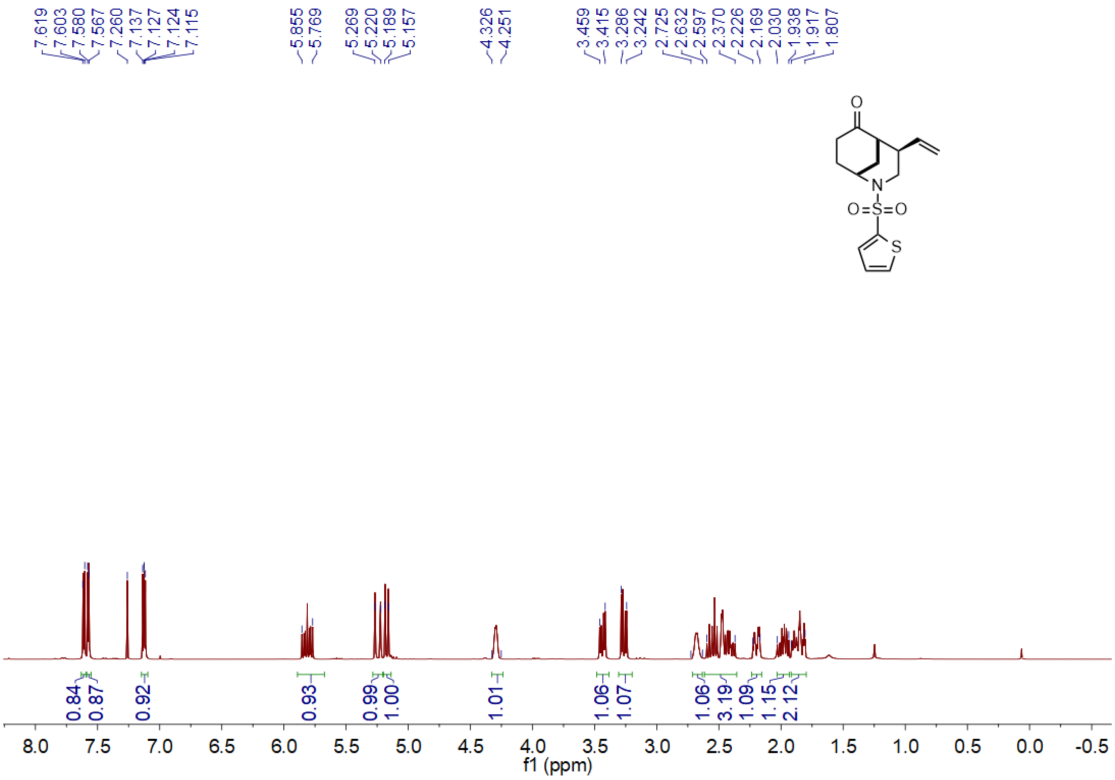
7.619
7.603
7.580
7.567
7.260
7.137
7.127
7.124
7.115
5.855
5.769
5.269
5.220
5.189
5.157
4.326
4.251
3.459
3.415
3.286
3.242
2.725
2.632
2.597
2.370
2.226
2.169
2.030
1.938
1.917
1.807
O
N
O=S=O
S
0.84
0.87
0.92
0.93
0.99
1.00
1.01
1.06
1.07
1.06
3.19
1.09
1.15
2.12
8.0 7.5 7.0 6.5 6.0 5.5 5.0 4.5 4.0 3.5 3.0 2.5 2.0 1.5 1.0 0.5 0.0 -0.5
f1 (ppm)

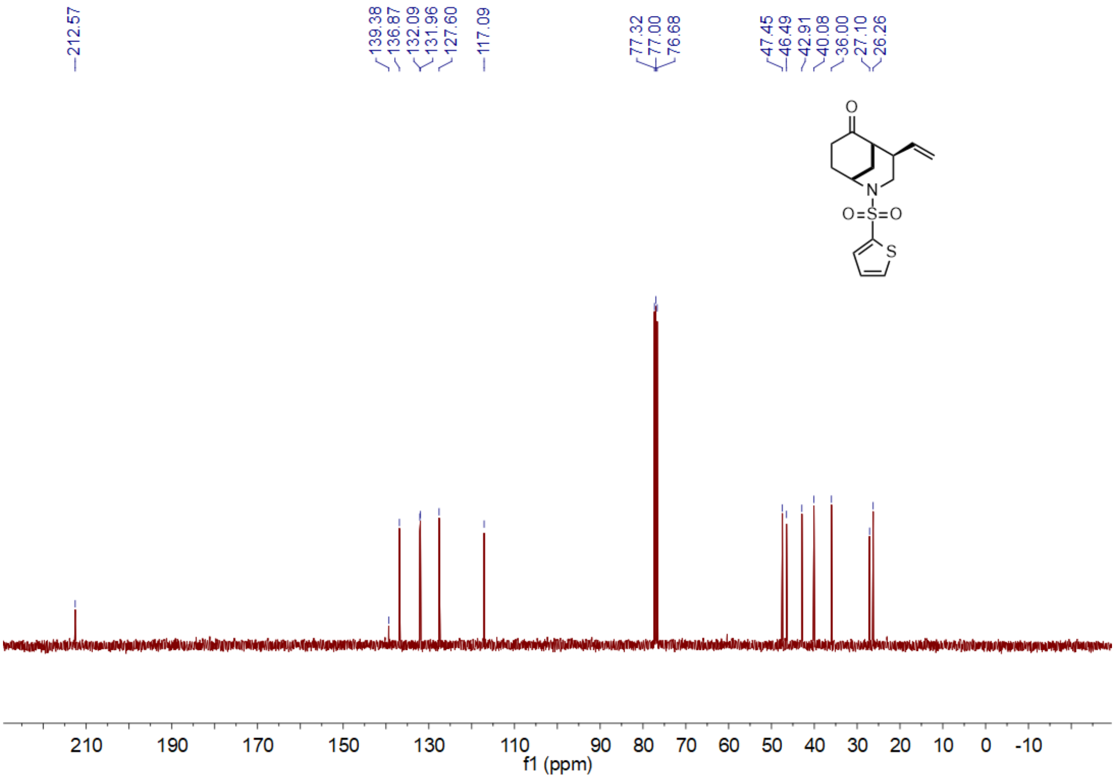
212.57
139.38
136.87
132.09
131.96
127.60
117.09
77.32
77.00
76.68
47.45
46.49
42.91
40.08
36.00
27.10
26.26
O
N
O=S=O
S
210 190 170 150 130 110 90 80 70 60 50 40 30 20 10 0 -10
f1 (ppm)

2m

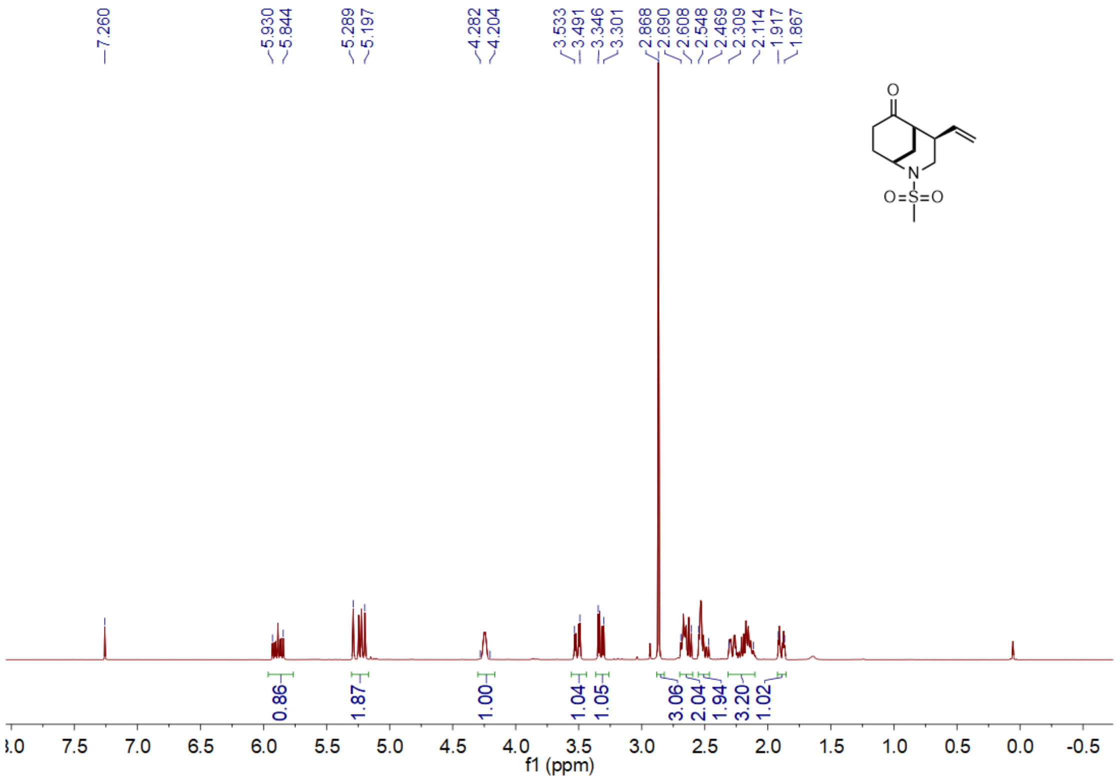
7.260
5.930
5.844
5.289
5.197
4.282
4.204
3.533
3.491
3.346
3.301
2.868
2.690
2.608
2.548
2.469
2.309
2.114
1.917
1.867
0.86
1.87
1.00
1.04
1.05
3.06
2.04
1.94
3.20
1.02
f1 (ppm)

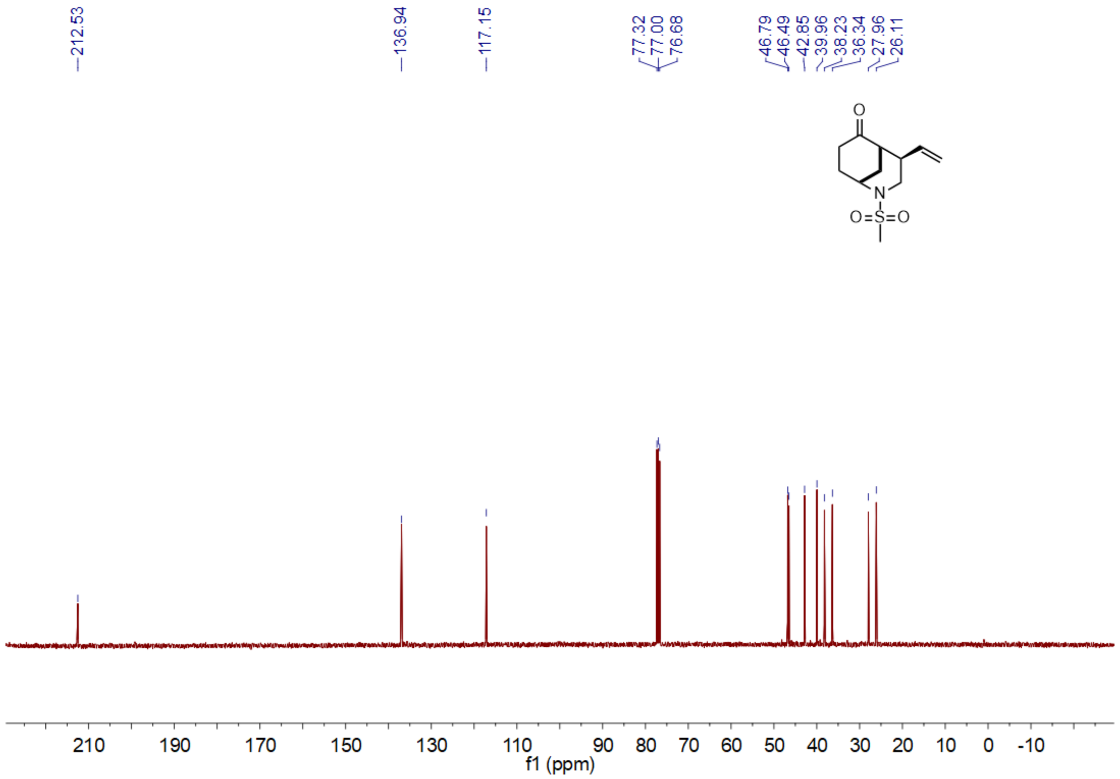
212.53
136.94
117.15
77.32
77.00
76.68
46.79
46.49
42.85
39.96
38.23
36.34
27.96
26.11
f1 (ppm)

2p

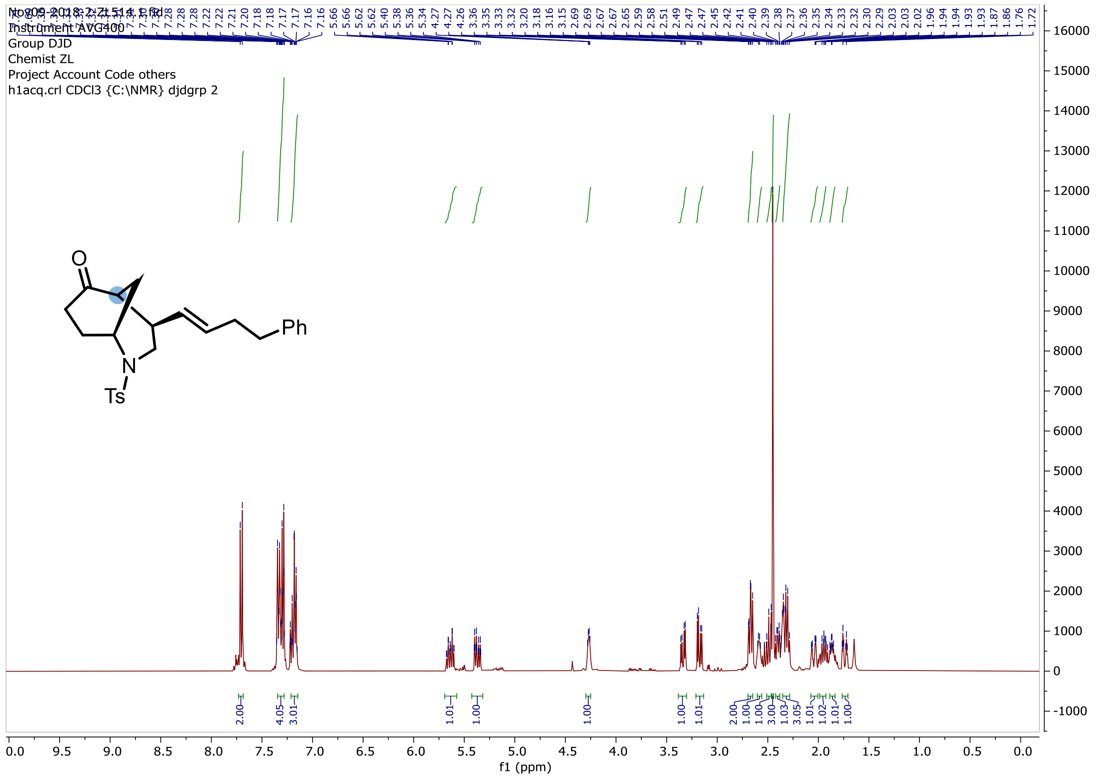

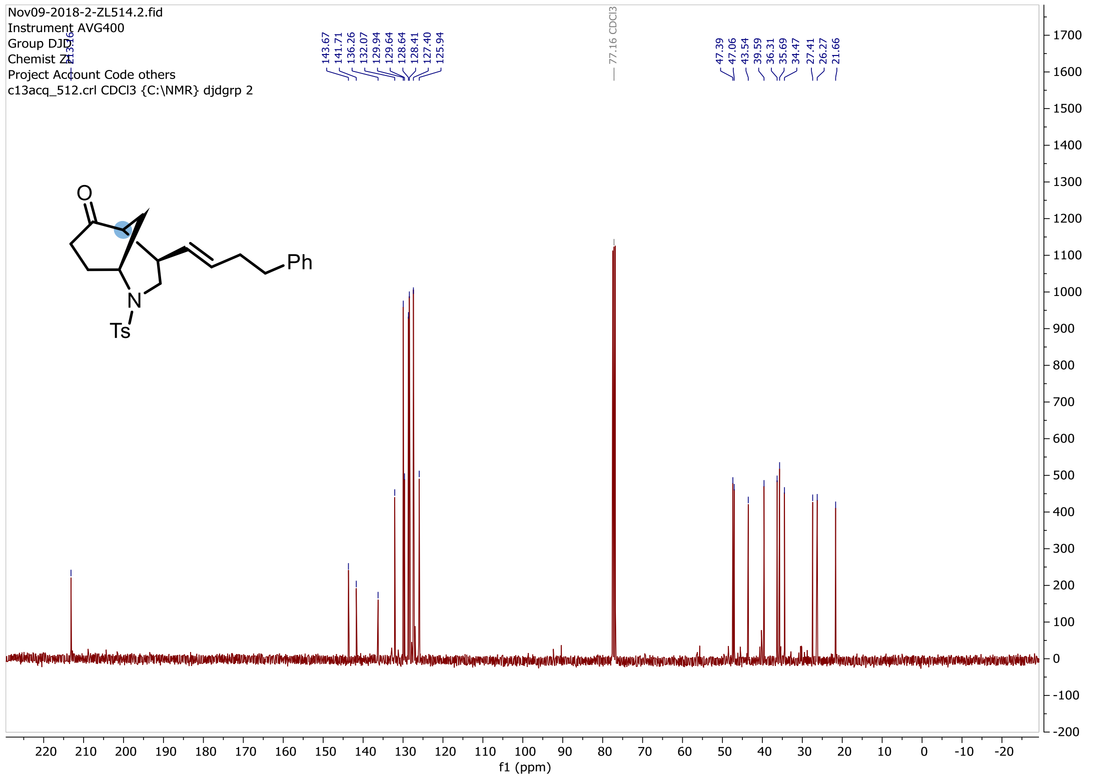

4b

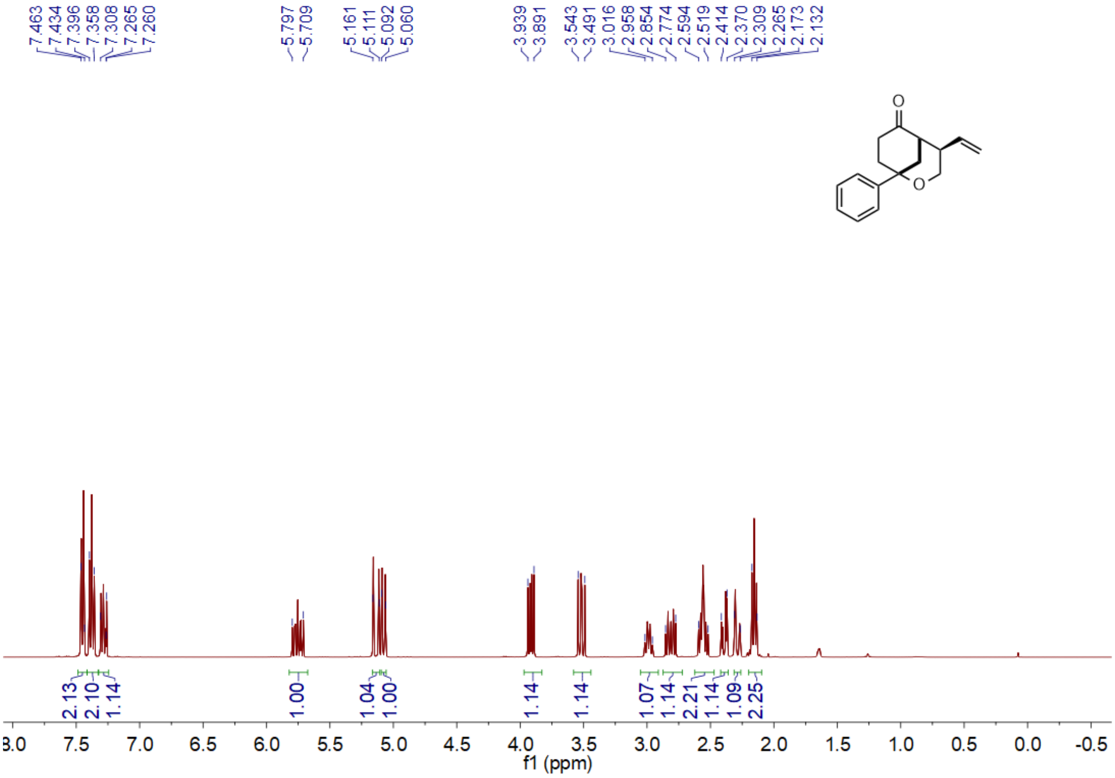
7.463
7.434
7.396
7.358
7.308
7.265
7.260
5.797
5.709
5.161
5.111
5.092
5.060
3.939
3.891
3.543
3.491
3.016
2.958
2.854
2.774
2.594
2.519
2.414
2.370
2.309
2.265
2.173
2.132
O
O
2.13
2.10
1.14
1.00
1.04
1.00
1.14
1.14
1.07
1.14
2.21
1.14
1.09
2.25
f1 (ppm)

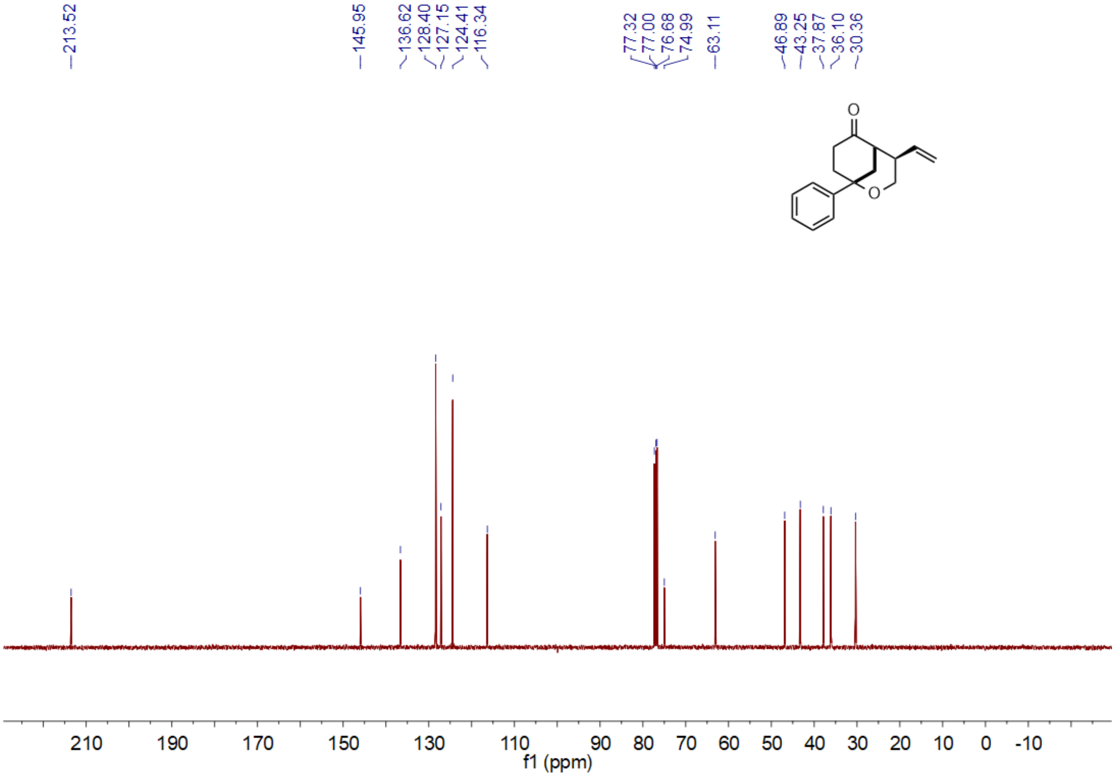
213.52
145.95
136.62
128.40
127.15
124.41
116.34
77.32
77.00
76.68
74.99
63.11
46.89
43.25
37.87
36.10
30.36
O
O
f1 (ppm)

4c

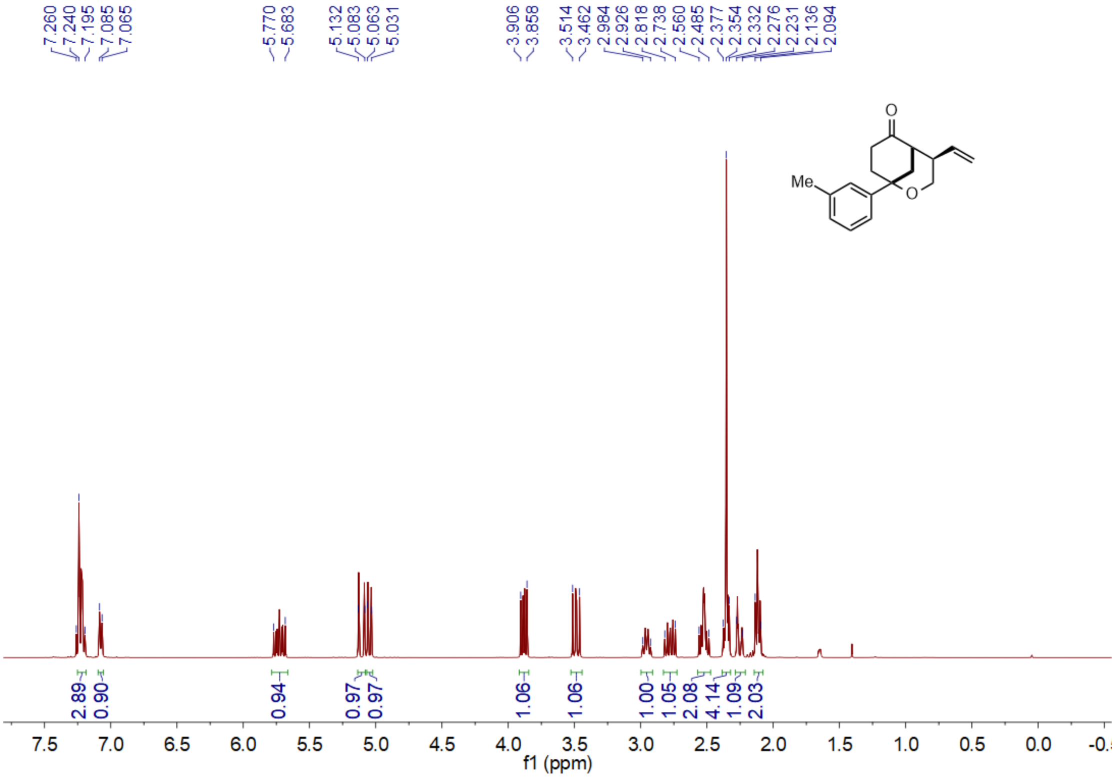
7.260
7.240
7.195
7.085
7.065
5.770
5.683
5.132
5.083
5.063
5.031
3.906
3.858
3.514
3.462
2.984
2.926
2.818
2.738
2.560
2.485
2.377
2.354
2.332
2.276
2.231
2.136
2.094
O
Me
O
2.89
0.90
0.94
0.97
0.97
1.06
1.06
1.00
1.05
2.08
4.14
1.09
2.03
f1 (ppm)

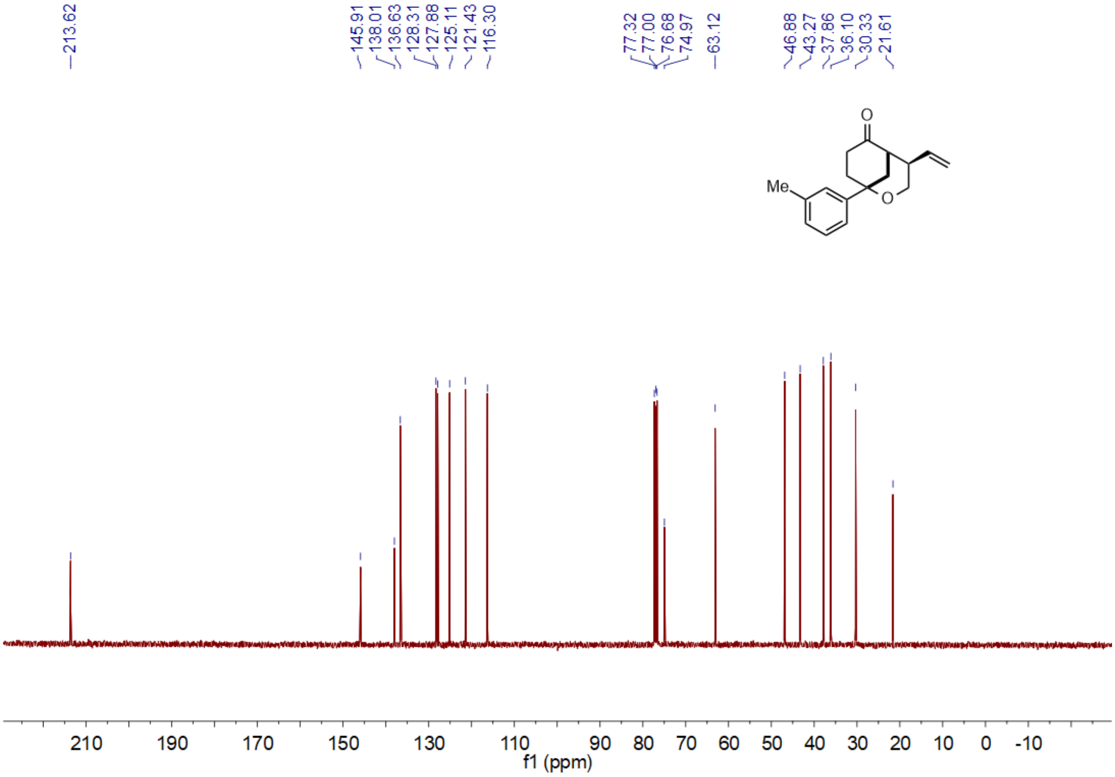
213.62
145.91
138.01
136.63
128.31
127.88
125.11
121.43
116.30
77.32
77.00
76.68
74.97
63.12
46.88
43.27
37.86
36.10
30.33
21.61
O
Me
O
f1 (ppm)

4d

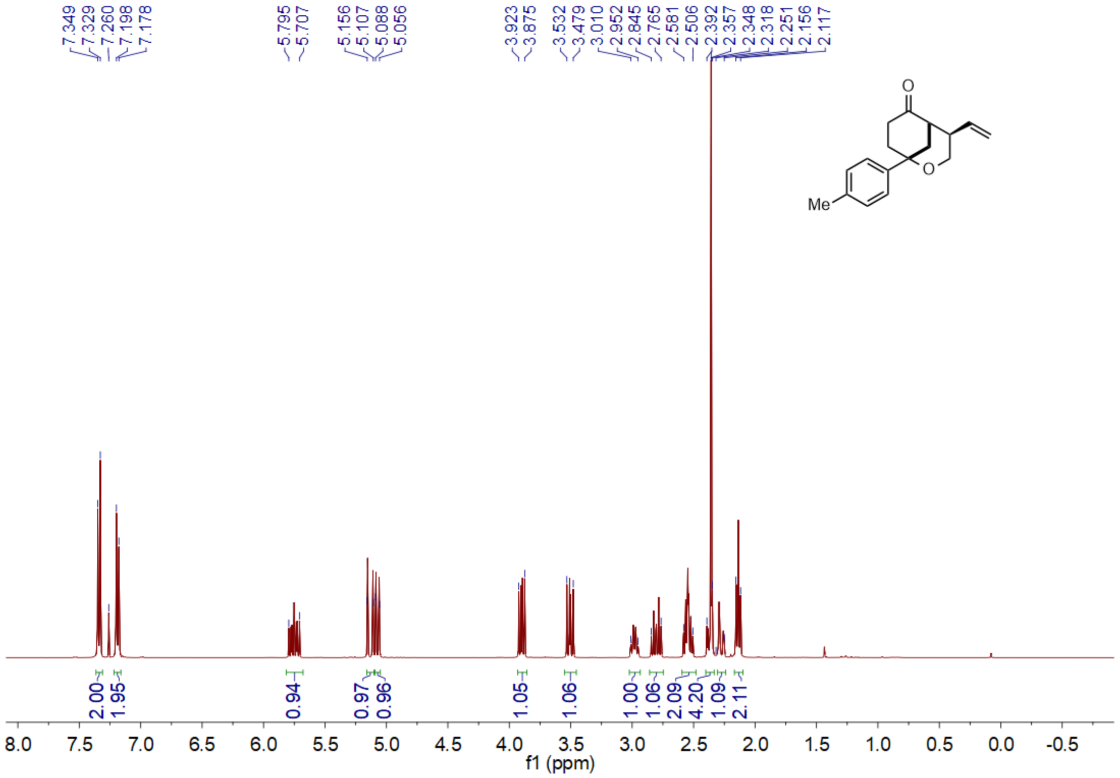
7.349
7.329
7.260
7.198
7.178
5.795
5.707
5.156
5.107
5.088
5.056
3.923
3.875
3.532
3.479
3.010
2.952
2.845
2.765
2.581
2.506
2.392
2.357
2.348
2.318
2.251
2.156
2.117
Me
O
2.00
1.95
0.94
0.97
0.96
1.05
1.06
1.00
1.06
2.09
4.20
1.09
2.11
8.0
7.5
7.0
6.5
6.0
5.5
5.0
4.5
4.0
3.5
3.0
2.5
2.0
1.5
1.0
0.5
0.0
-0.5
f1 (ppm)

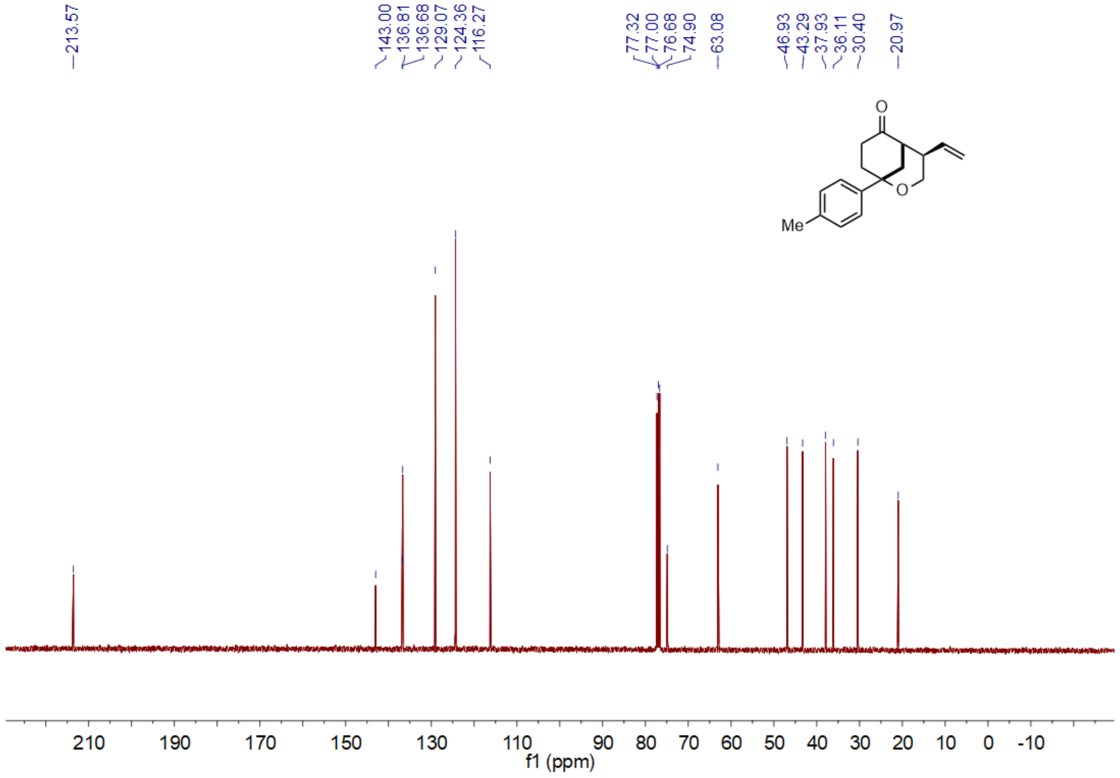
213.57
143.00
136.81
136.68
129.07
124.36
116.27
77.32
77.00
76.68
74.90
63.08
46.93
43.29
37.93
36.11
30.40
20.97
Me
O
210
190
170
150
130
110
90
80
70
60
50
40
30
20
10
0
-10
f1 (ppm)

4e

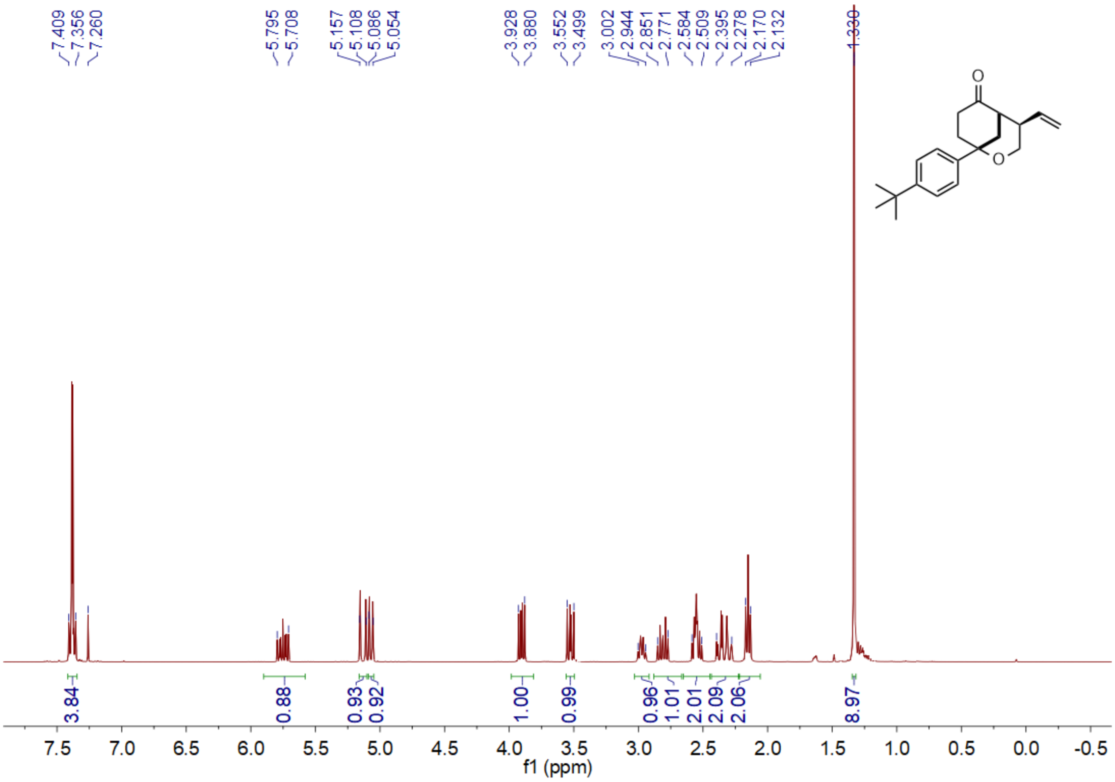

7.409
7.356
7.260
5.795
5.708
5.157
5.108
5.086
5.054
3.928
3.880
3.552
3.499
3.002
2.944
2.851
2.771
2.584
2.509
2.395
2.278
2.170
2.132
1.330
O
O
3.84
0.88
0.93
0.92
1.00
0.99
0.96
1.01
2.01
2.09
2.06
8.97
7.5 7.0 6.5 6.0 5.5 5.0 4.5 4.0 3.5 3.0 2.5 2.0 1.5 1.0 0.5 0.0 -0.5
f1 (ppm)

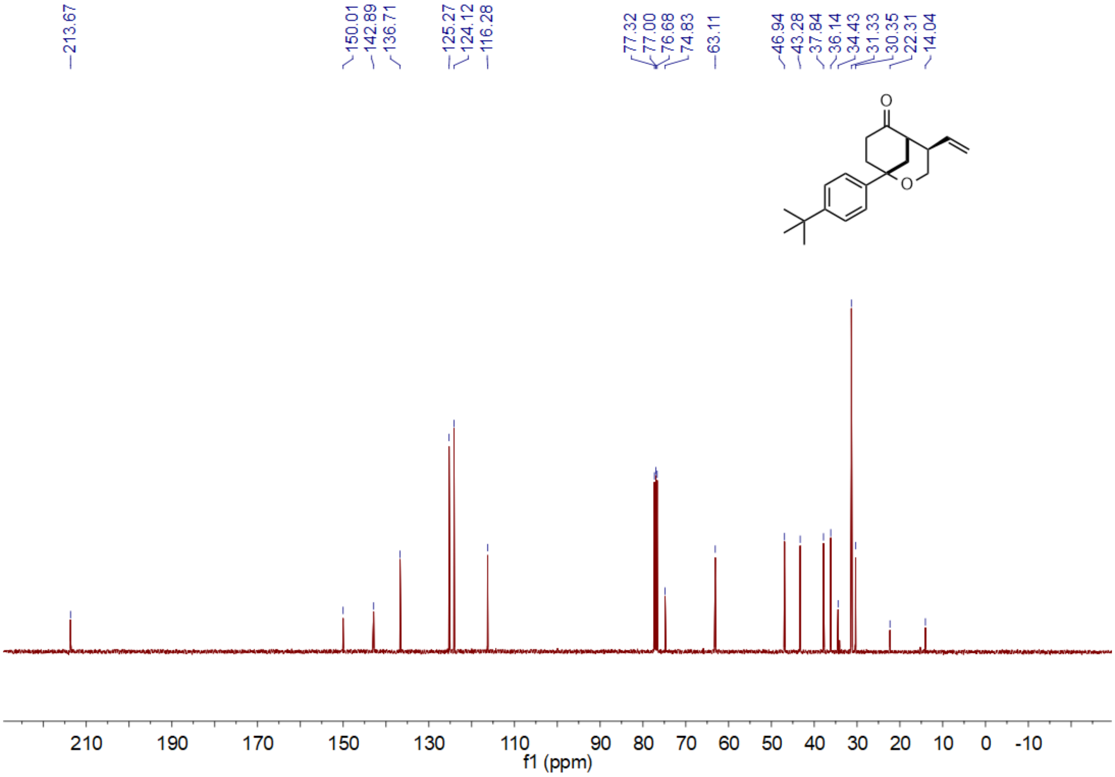

213.67
150.01
142.89
136.71
125.27
124.12
116.28
77.32
77.00
76.68
74.83
63.11
46.94
43.28
37.84
36.14
34.43
31.33
30.35
22.31
14.04
O
O
210 190 170 150 130 110 90 80 70 60 50 40 30 20 10 0 -10
f1 (ppm)

4f

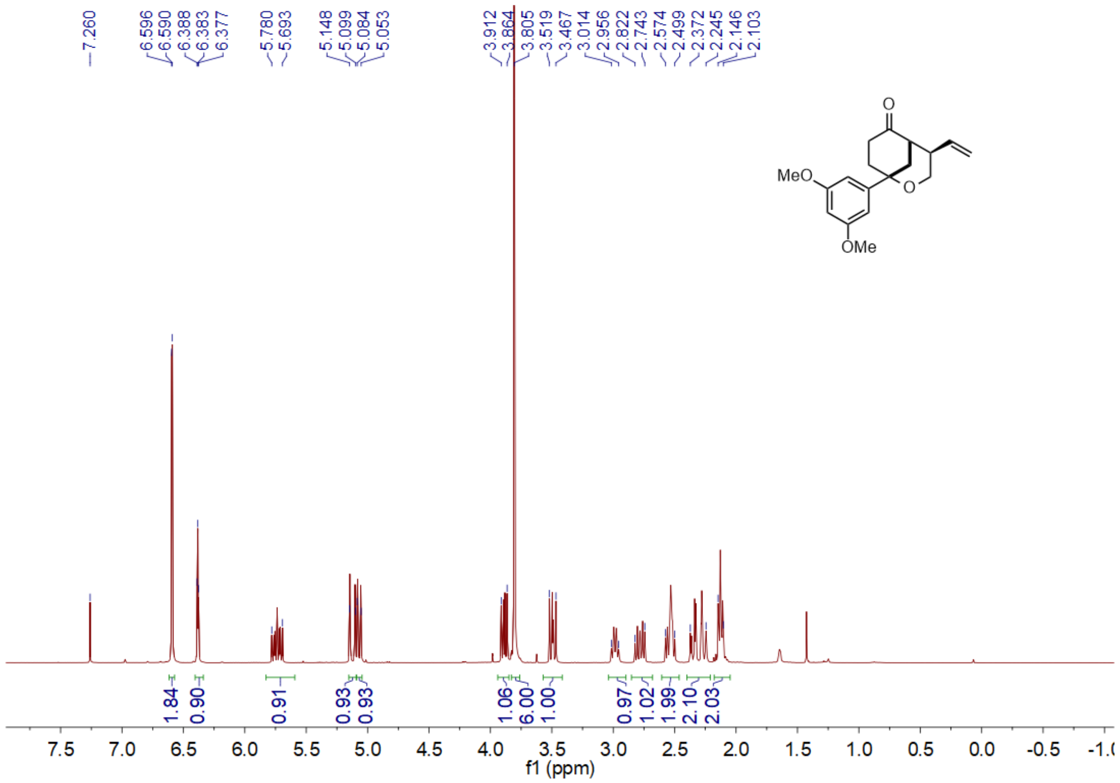

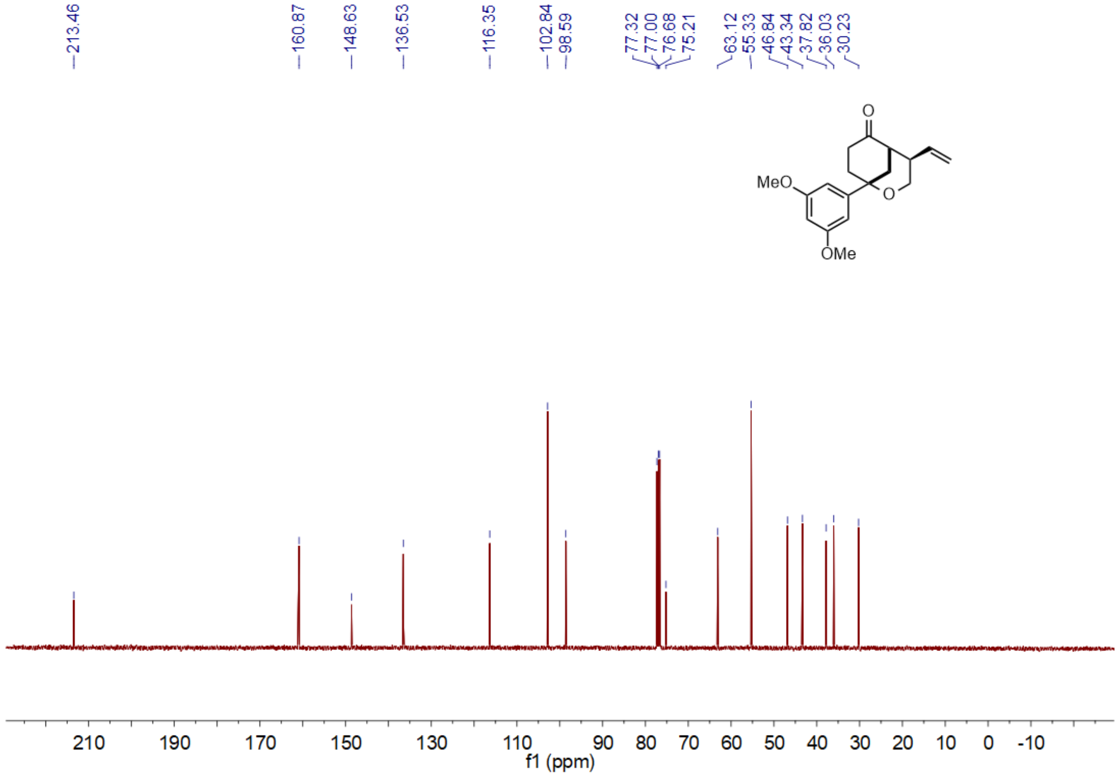

4g

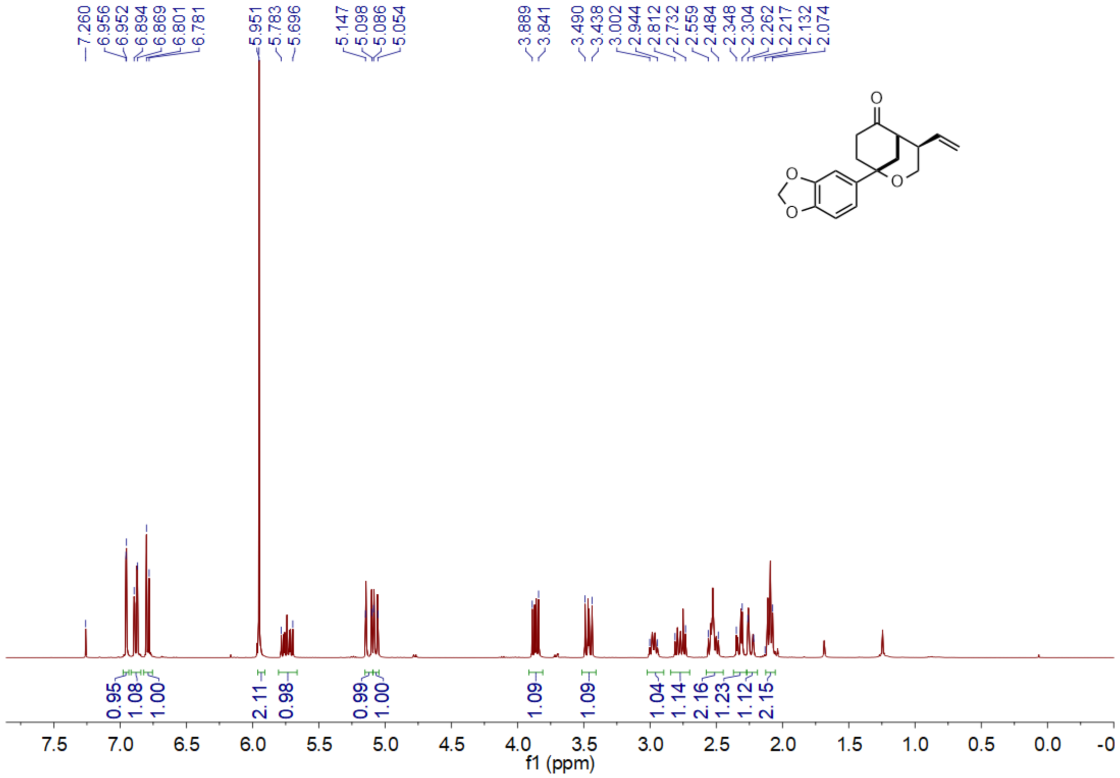
7.260
6.956
6.952
6.894
6.869
6.801
6.781
5.951
5.783
5.696
5.147
5.098
5.086
5.054
3.889
3.841
3.490
3.438
3.002
2.944
2.812
2.732
2.559
2.484
2.348
2.304
2.262
2.217
2.132
2.074
O
O
O
O
0.95
1.08
1.00
2.11
0.98
0.99
1.00
1.09
1.09
1.04
1.14
2.16
1.23
1.12
2.15
7.5 7.0 6.5 6.0 5.5 5.0 4.5 4.0 3.5 3.0 2.5 2.0 1.5 1.0 0.5 0.0 -0
f1 (ppm)

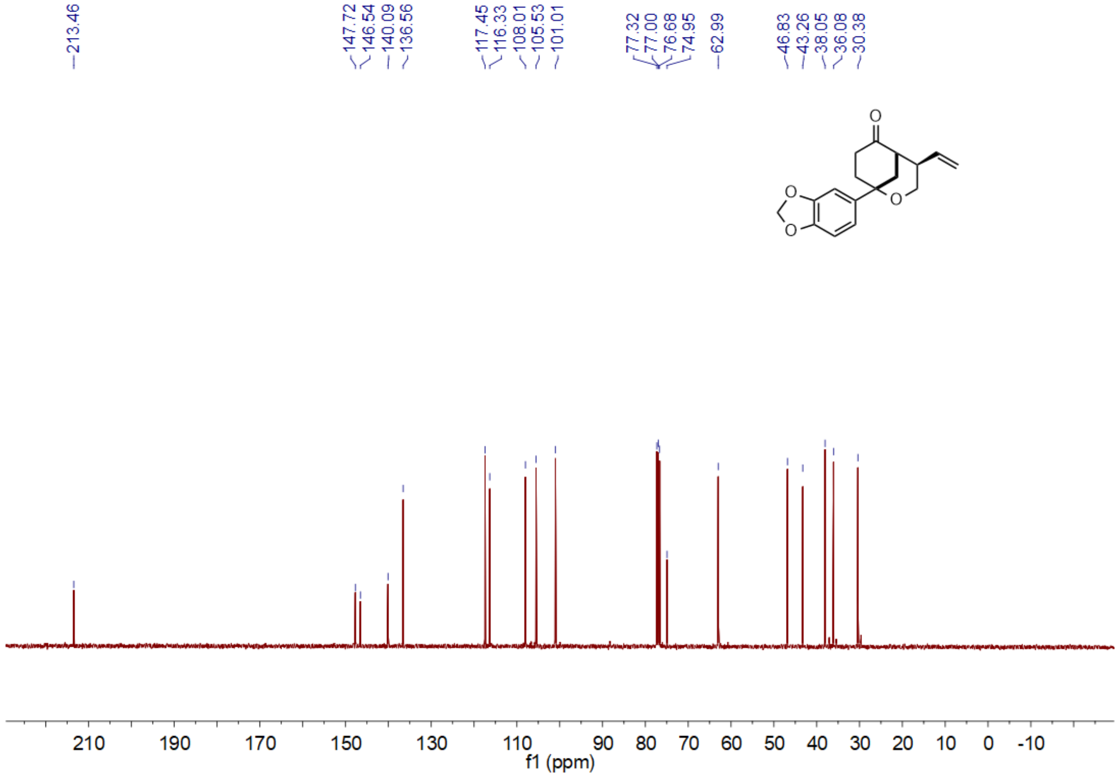
213.46
147.72
146.54
140.09
136.56
117.45
116.33
108.01
105.53
101.01
77.32
77.00
76.68
74.95
62.99
46.83
43.26
38.05
36.08
30.38
O
O
O
O
210 190 170 150 130 110 90 80 70 60 50 40 30 20 10 0 -10
f1 (ppm)

4h

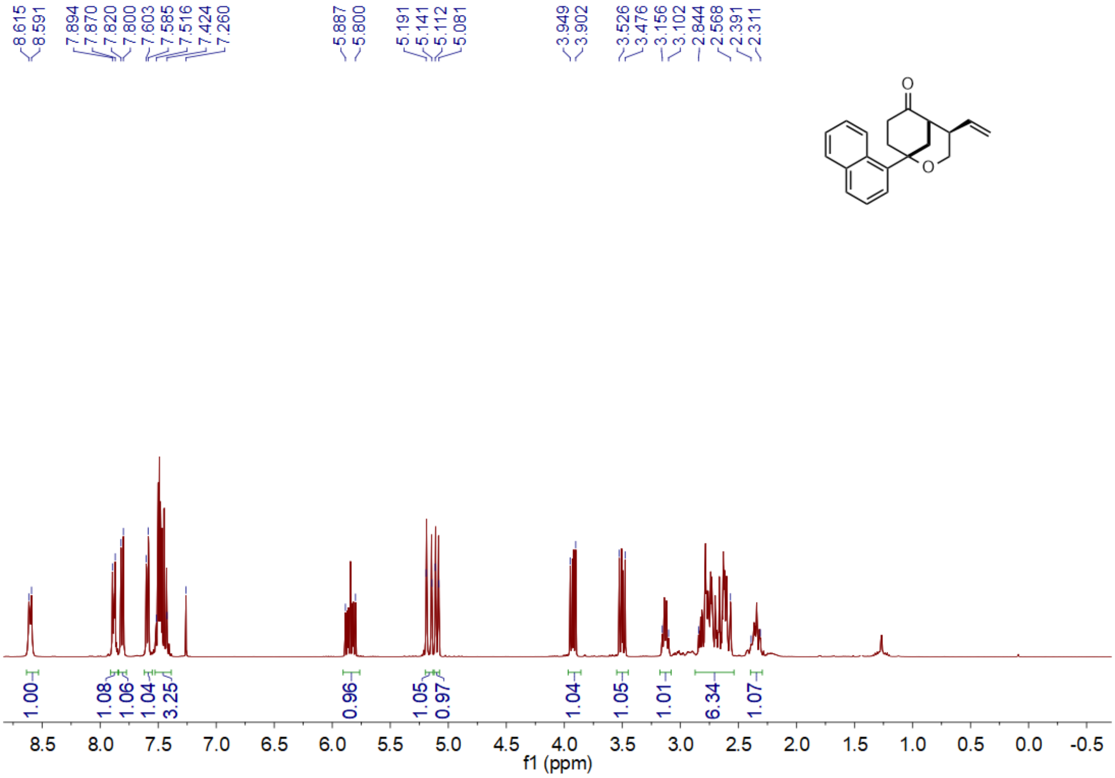

8.615
8.591
7.894
7.870
7.820
7.800
7.603
7.585
7.516
7.424
7.260
5.887
5.800
5.191
5.141
5.112
5.081
3.949
3.902
3.526
3.476
3.156
3.102
2.844
2.568
2.391
2.311
O
O
1.00
1.08
1.06
1.04
3.25
0.96
1.05
0.97
1.04
1.05
1.01
6.34
1.07
8.5 8.0 7.5 7.0 6.5 6.0 5.5 5.0 4.5 4.0 3.5 3.0 2.5 2.0 1.5 1.0 0.5 0.0 -0.5
f1 (ppm)

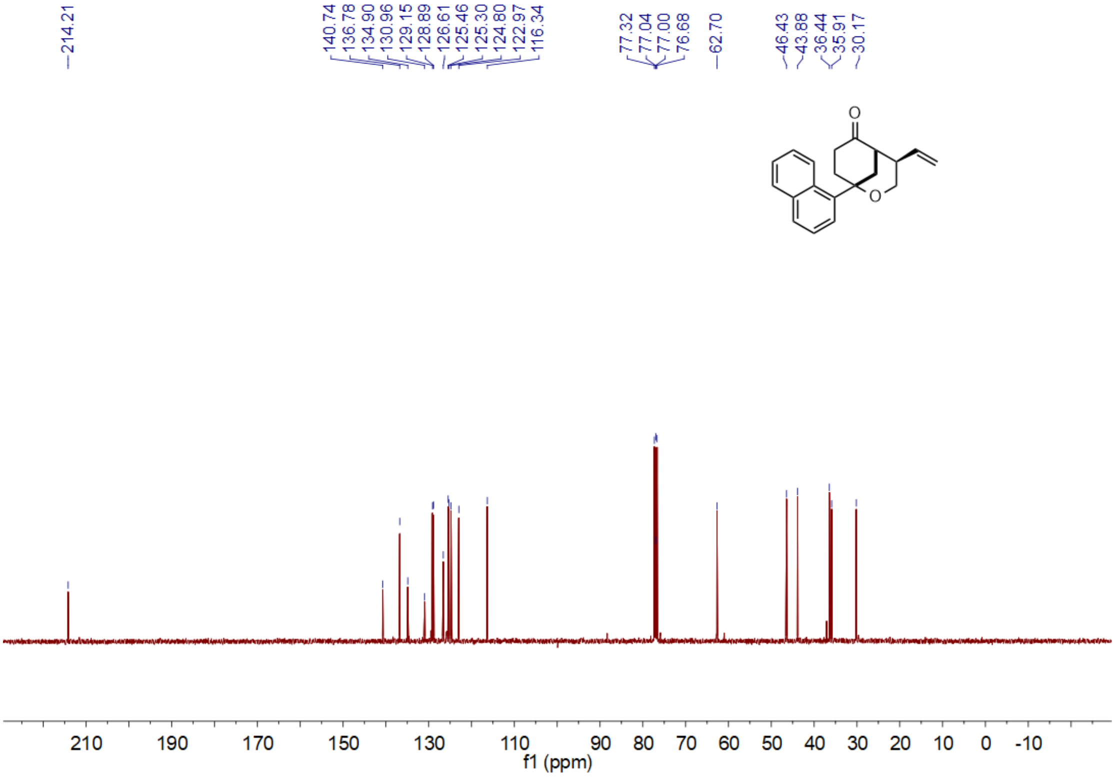

214.21
140.74
136.78
134.90
130.96
129.15
128.89
126.61
125.46
125.30
124.80
122.97
116.34
77.32
77.04
77.00
76.68
62.70
46.43
43.88
36.44
35.91
30.17
O
O
210 190 170 150 130 110 90 80 70 60 50 40 30 20 10 0 -10
f1 (ppm)

4i

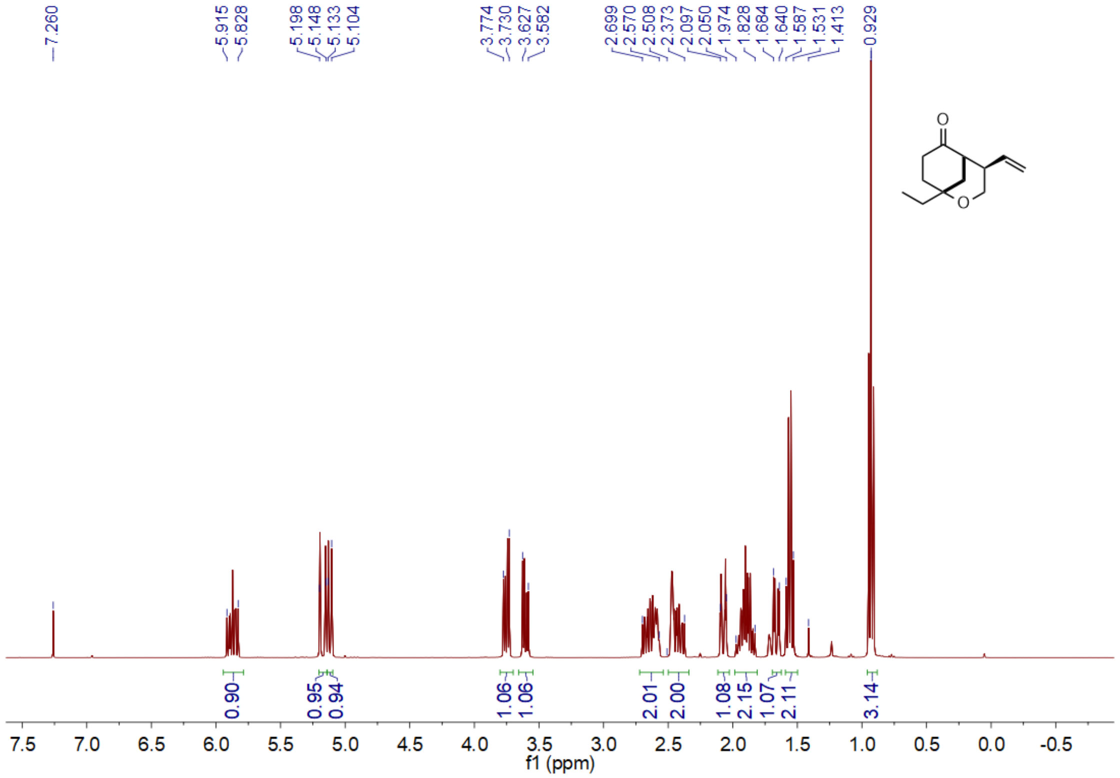
7.260
5.915
5.828
5.198
5.148
5.133
5.104
3.774
3.730
3.627
3.582
2.699
2.570
2.508
2.373
2.097
2.050
1.974
1.828
1.684
1.640
1.587
1.531
1.413
0.929
O
O
0.90
0.95
0.94
1.06
1.06
2.01
2.00
1.08
2.15
1.07
2.11
3.14
7.5 7.0 6.5 6.0 5.5 5.0 4.5 4.0 3.5 3.0 2.5 2.0 1.5 1.0 0.5 0.0 -0.5
f1 (ppm)

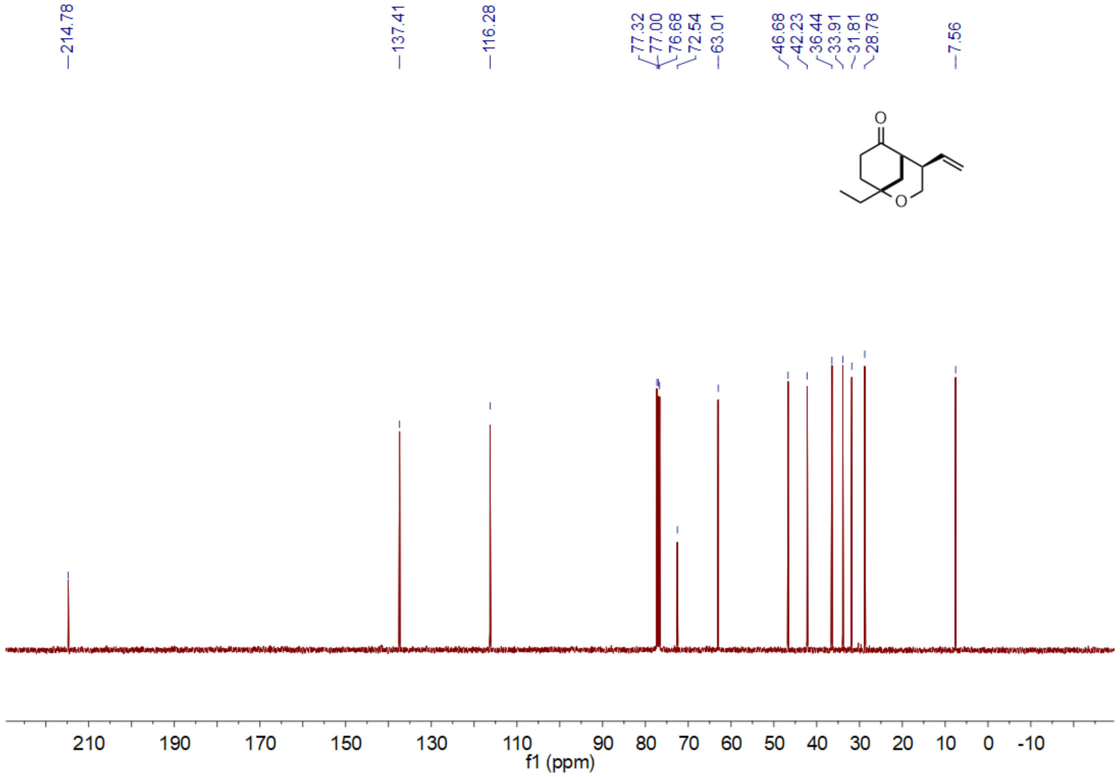
214.78
137.41
116.28
77.32
77.00
76.68
72.54
63.01
46.68
42.23
36.44
33.91
31.81
28.78
7.56
O
O
210 190 170 150 130 110 90 80 70 60 50 40 30 20 10 0 -10
f1 (ppm)

4j

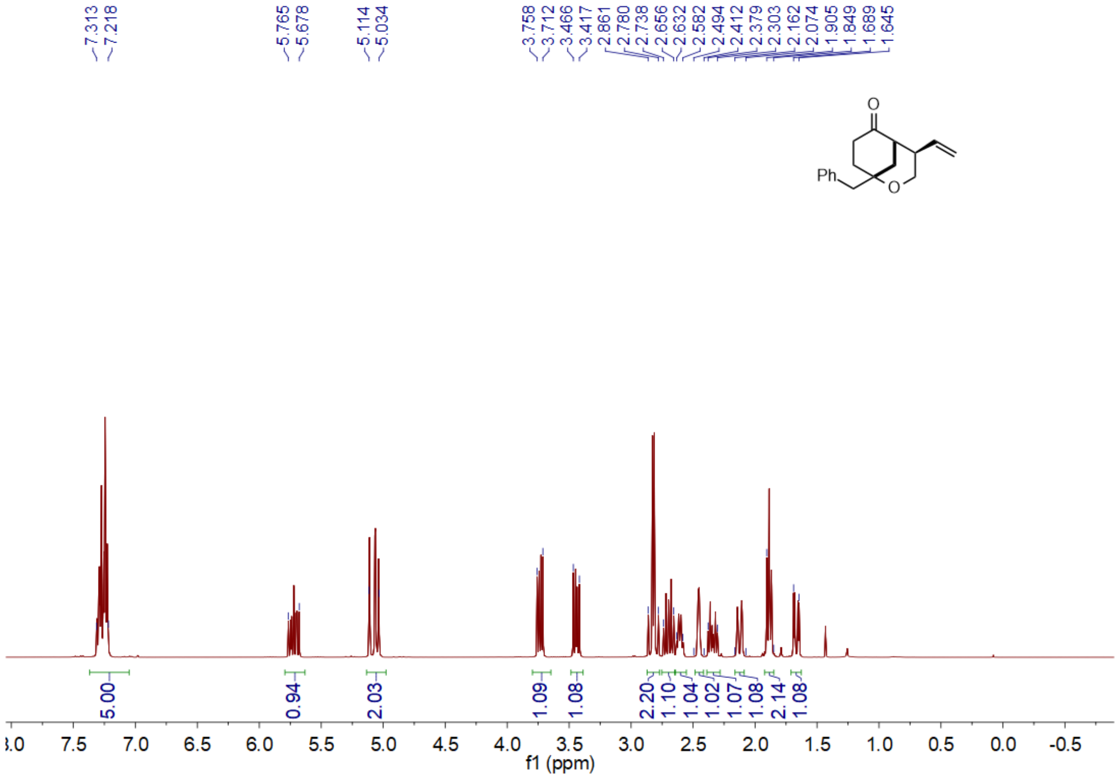
7.313
7.218
5.765
5.678
5.114
5.034
3.758
3.712
3.466
3.417
2.861
2.780
2.738
2.656
2.632
2.582
2.494
2.412
2.379
2.303
2.162
2.074
1.905
1.849
1.689
1.645
O
Ph
O
5.00
0.94
2.03
1.09
1.08
2.20
1.10
1.04
1.02
1.07
1.08
2.14
1.08
7.5 7.0 6.5 6.0 5.5 5.0 4.5 4.0 3.5 3.0 2.5 2.0 1.5 1.0 0.5 0.0 -0.5
f1 (ppm)

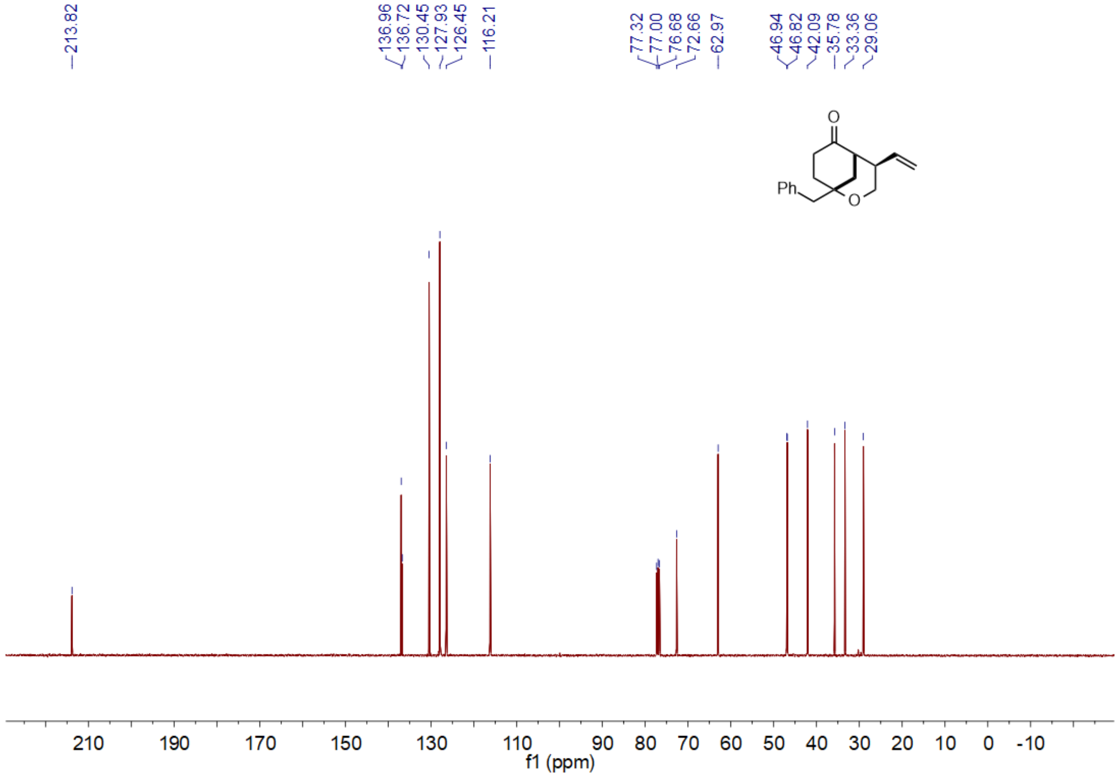
213.82
136.96
136.72
130.45
127.93
126.45
116.21
77.32
77.00
76.68
72.66
62.97
46.94
46.82
42.09
35.78
33.36
29.06
O
Ph
O
210 190 170 150 130 110 90 80 70 60 50 40 30 20 10 0 -10
f1 (ppm)

4k

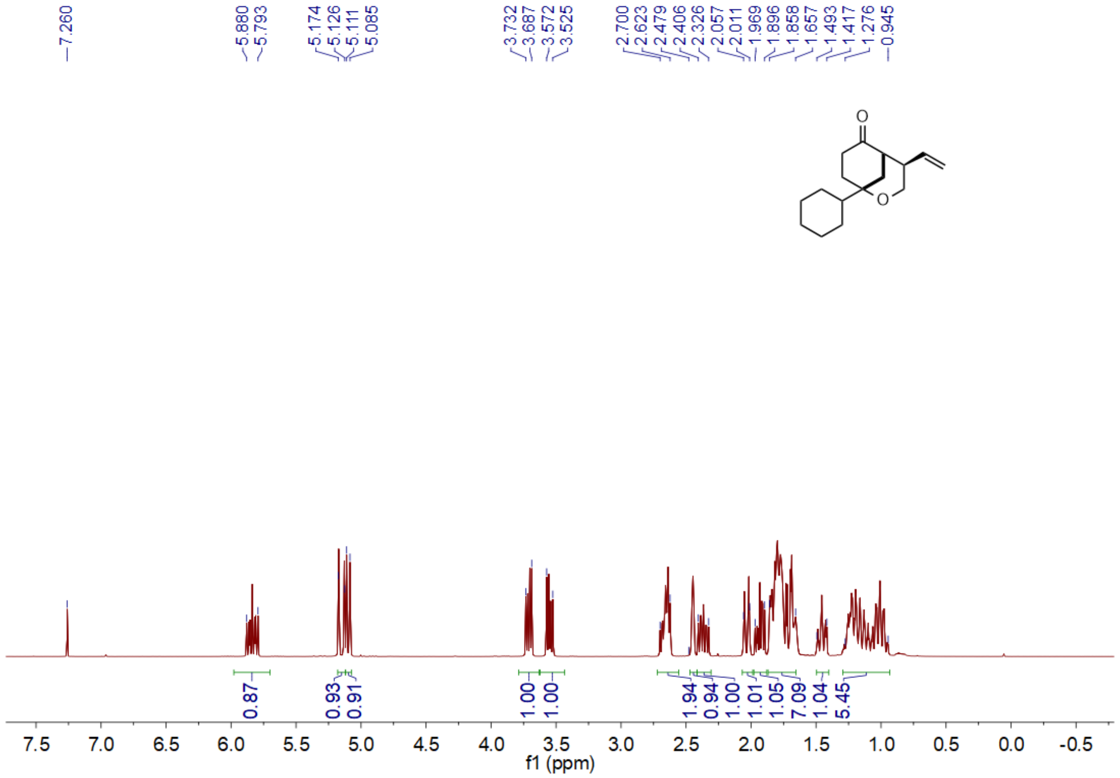
7.260
5.880
5.793
5.174
5.126
5.111
5.085
3.732
3.687
3.572
3.525
2.700
2.623
2.479
2.406
2.326
2.057
2.011
1.969
1.896
1.858
1.657
1.493
1.417
1.276
0.945
O
O
0.87
0.93
0.91
1.00
1.00
1.94
0.94
1.00
1.01
1.05
7.09
1.04
5.45
7.5 7.0 6.5 6.0 5.5 5.0 4.5 4.0 3.5 3.0 2.5 2.0 1.5 1.0 0.5 0.0 -0.5
f1 (ppm)

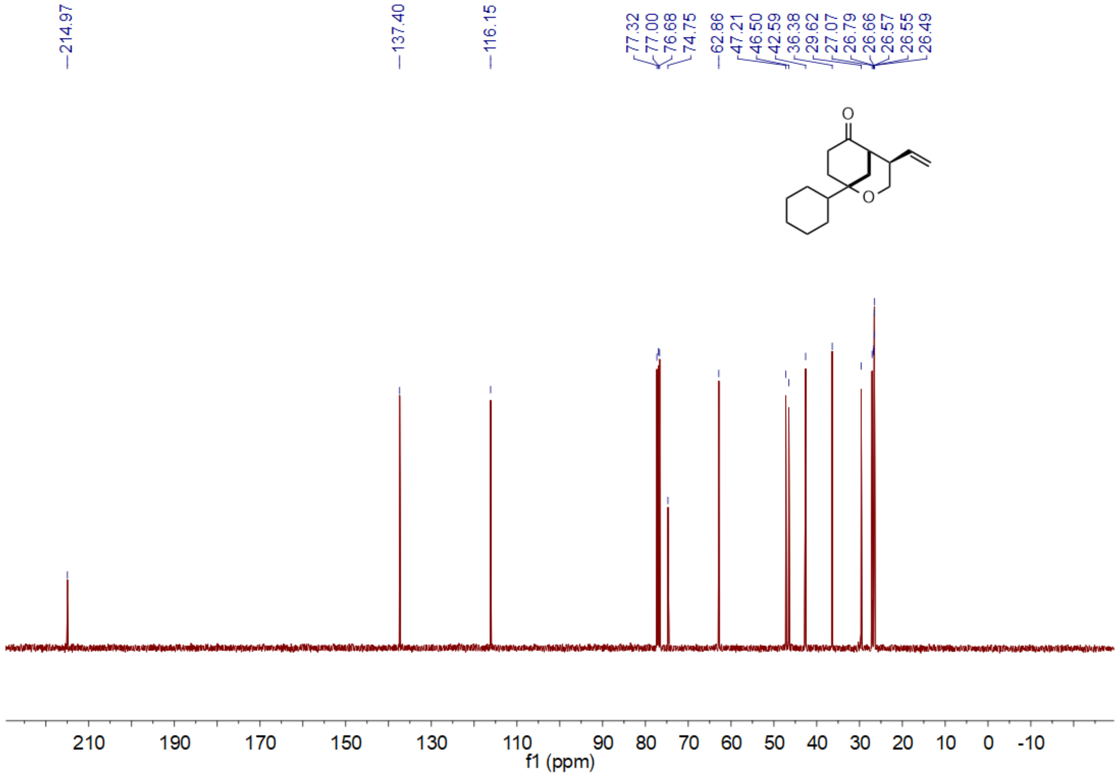
214.97
137.40
116.15
77.32
77.00
76.68
74.75
62.86
47.21
46.50
42.59
36.38
29.62
27.07
26.79
26.66
26.57
26.55
26.49
O
O
210 190 170 150 130 110 90 80 70 60 50 40 30 20 10 0 -10
f1 (ppm)

41

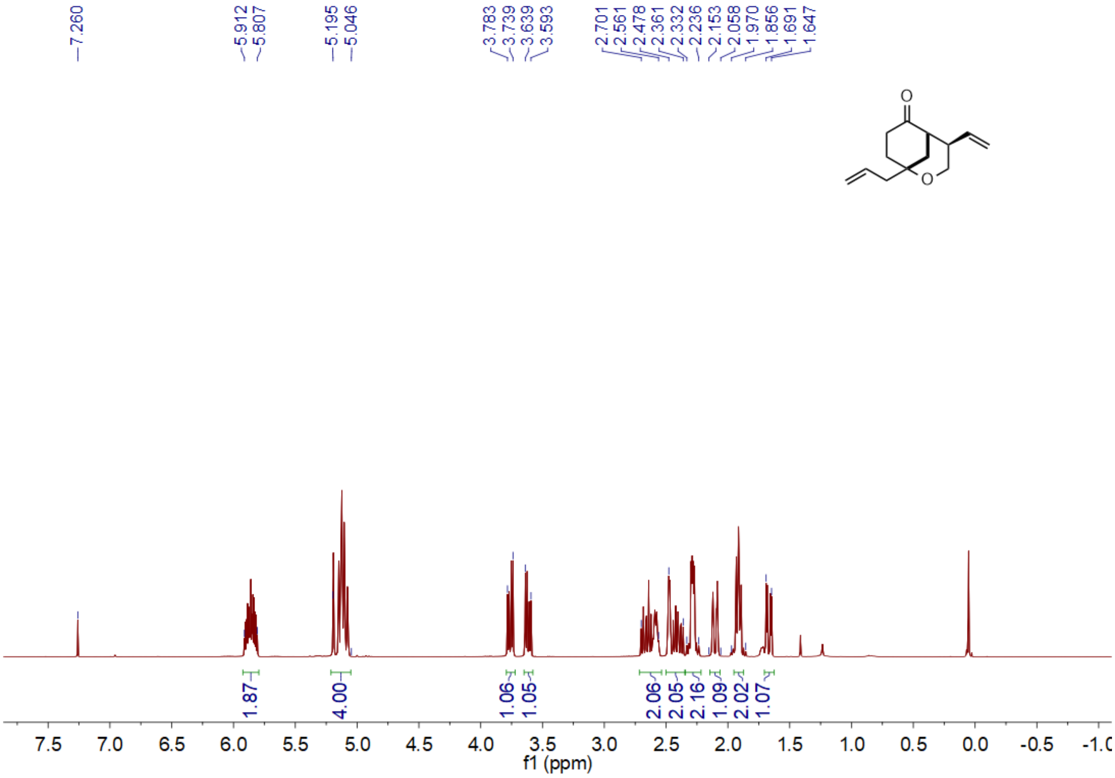
7.260
5.912
5.807
5.195
5.046
3.783
3.739
3.639
3.593
2.701
2.561
2.478
2.361
2.332
2.236
2.153
2.058
1.970
1.856
1.691
1.647
O
O
1.87
4.00
1.06
1.05
2.06
2.05
2.16
1.09
2.02
1.07
7.5 7.0 6.5 6.0 5.5 5.0 4.5 4.0 3.5 3.0 2.5 2.0 1.5 1.0 0.5 0.0 -0.5
f1 (ppm)

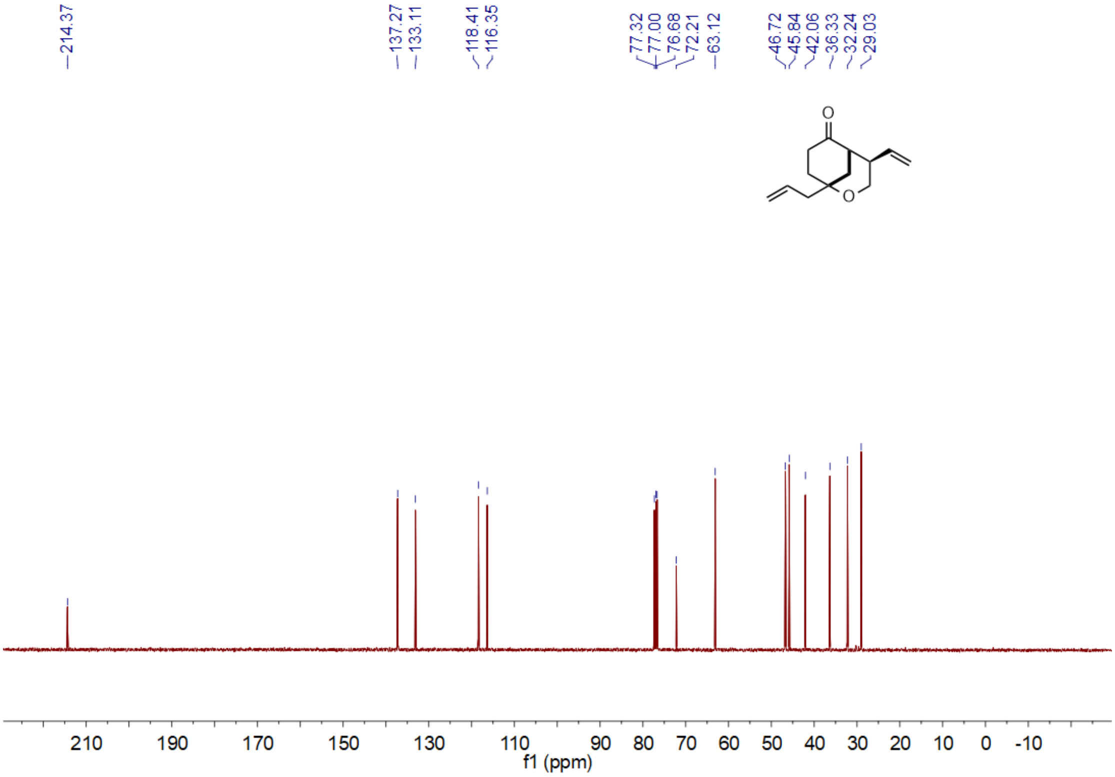
214.37
137.27
133.11
118.41
116.35
77.32
77.00
76.68
72.21
63.12
46.72
45.84
42.06
36.33
32.24
29.03
O
O
210 190 170 150 130 110 90 80 70 60 50 40 30 20 10 0 -10
f1 (ppm)

8.4 Spectra of the intermediates towards starting materials

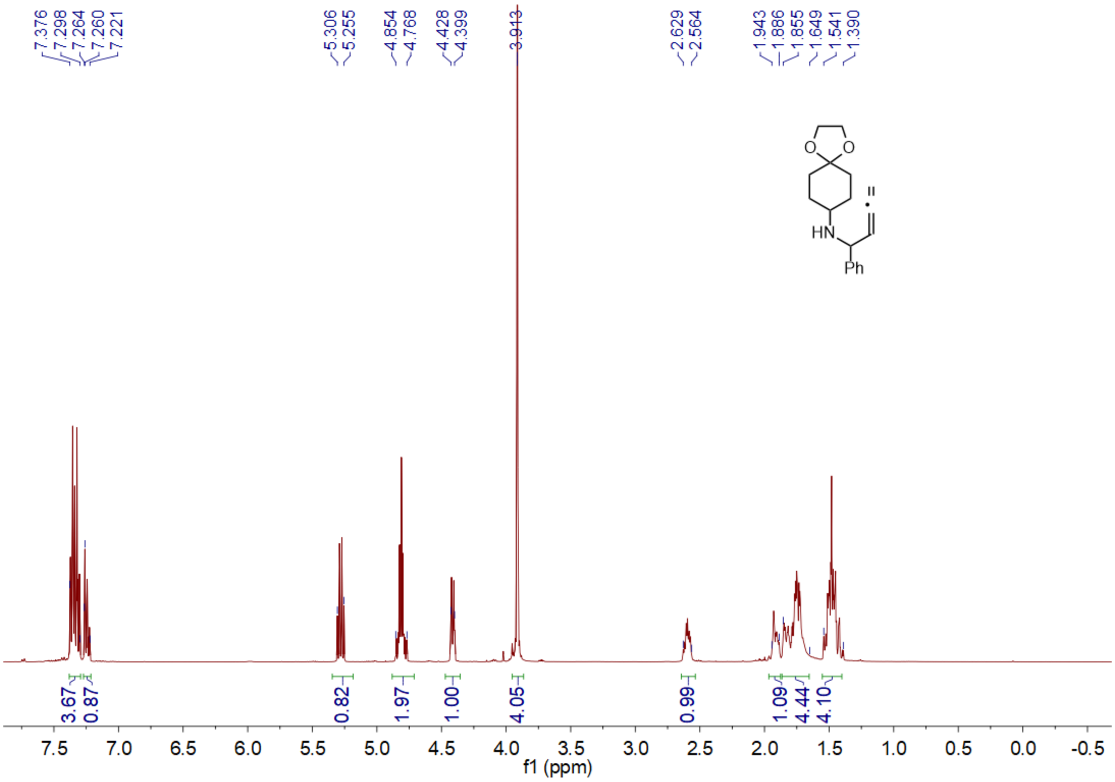

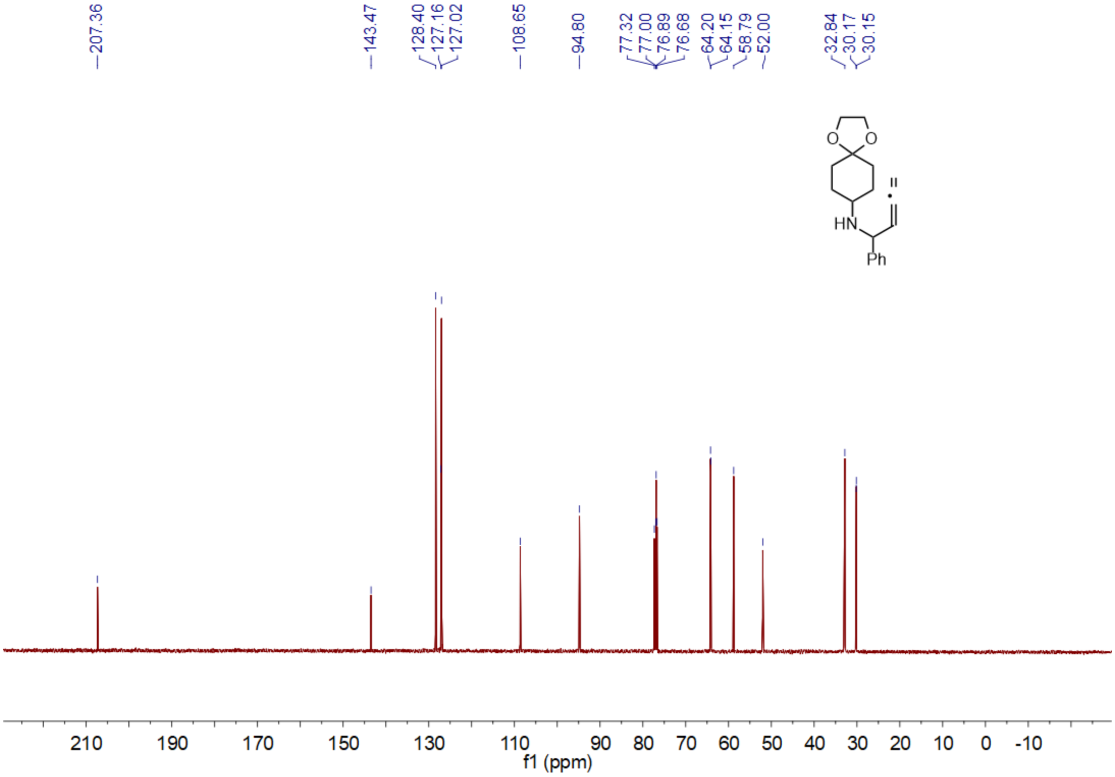

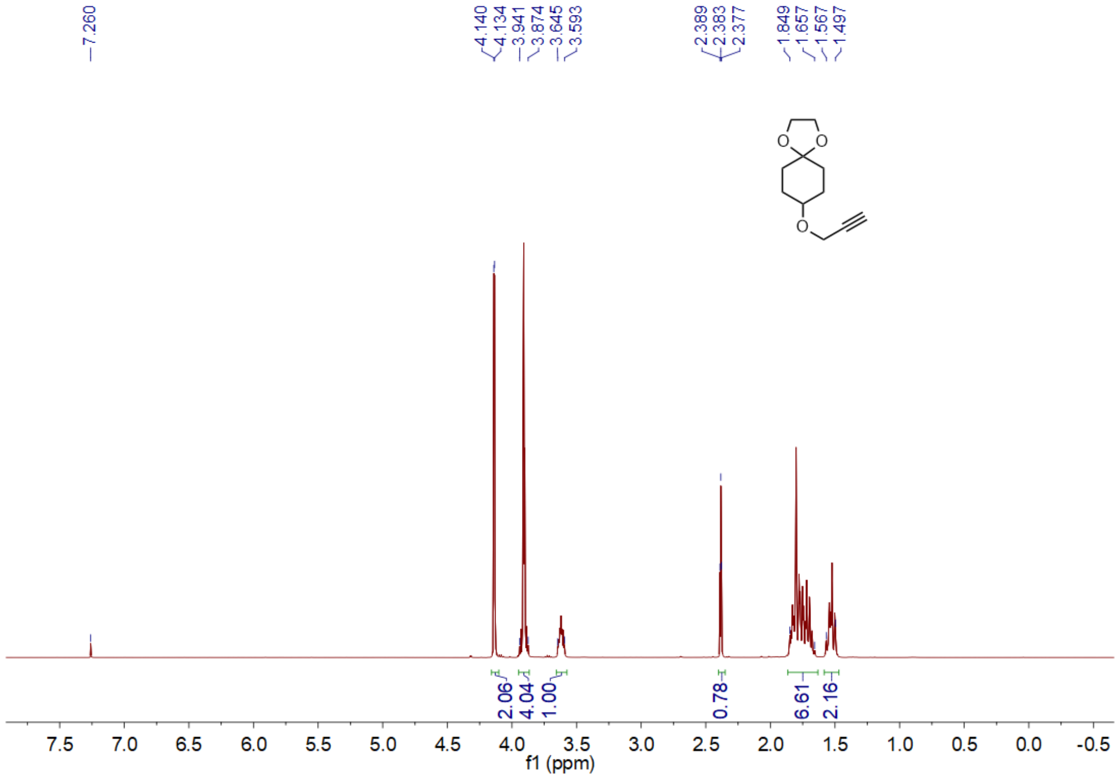
7.260
4.140
4.134
3.941
3.874
3.645
3.593
2.389
2.383
2.377
1.849
1.657
1.567
1.497
O
O
O
2.06
4.04
1.00
0.78
6.61
2.16
7.5 7.0 6.5 6.0 5.5 5.0 4.5 4.0 3.5 3.0 2.5 2.0 1.5 1.0 0.5 0.0 -0.5
f1 (ppm)

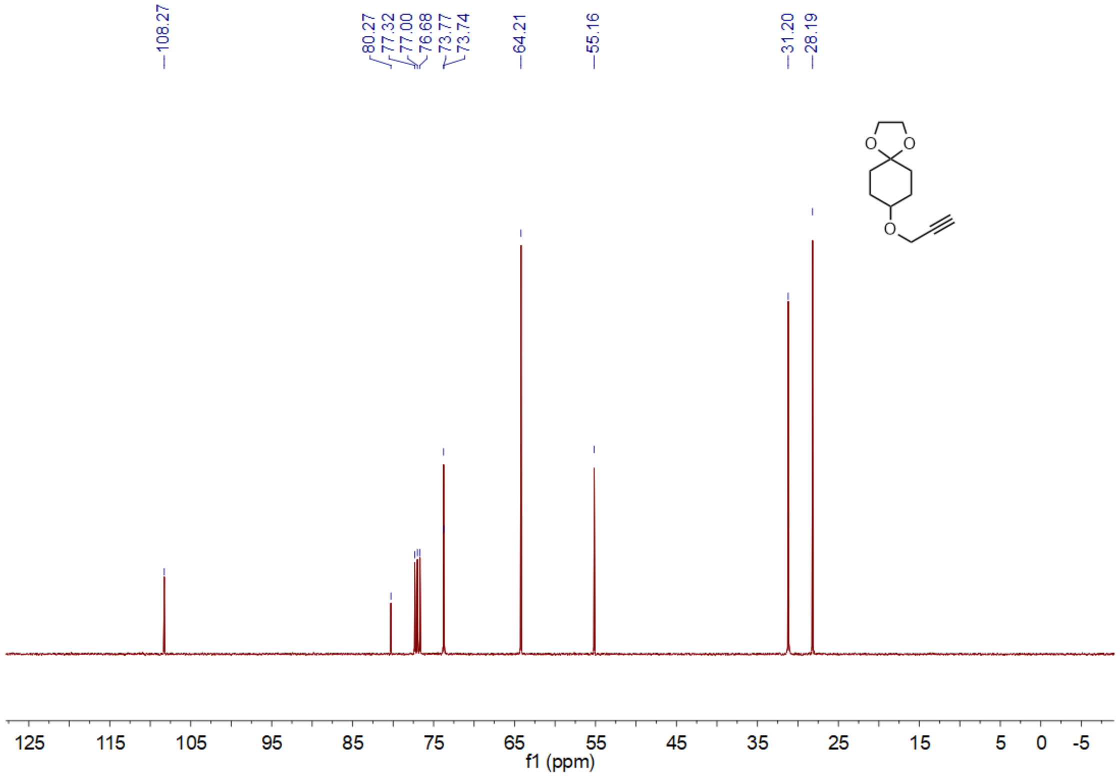
108.27
80.27
77.32
77.00
76.68
73.77
73.74
64.21
55.16
31.20
28.19
O
O
O
125 115 105 95 85 75 65 55 45 35 25 15 5 0 -5
f1 (ppm)

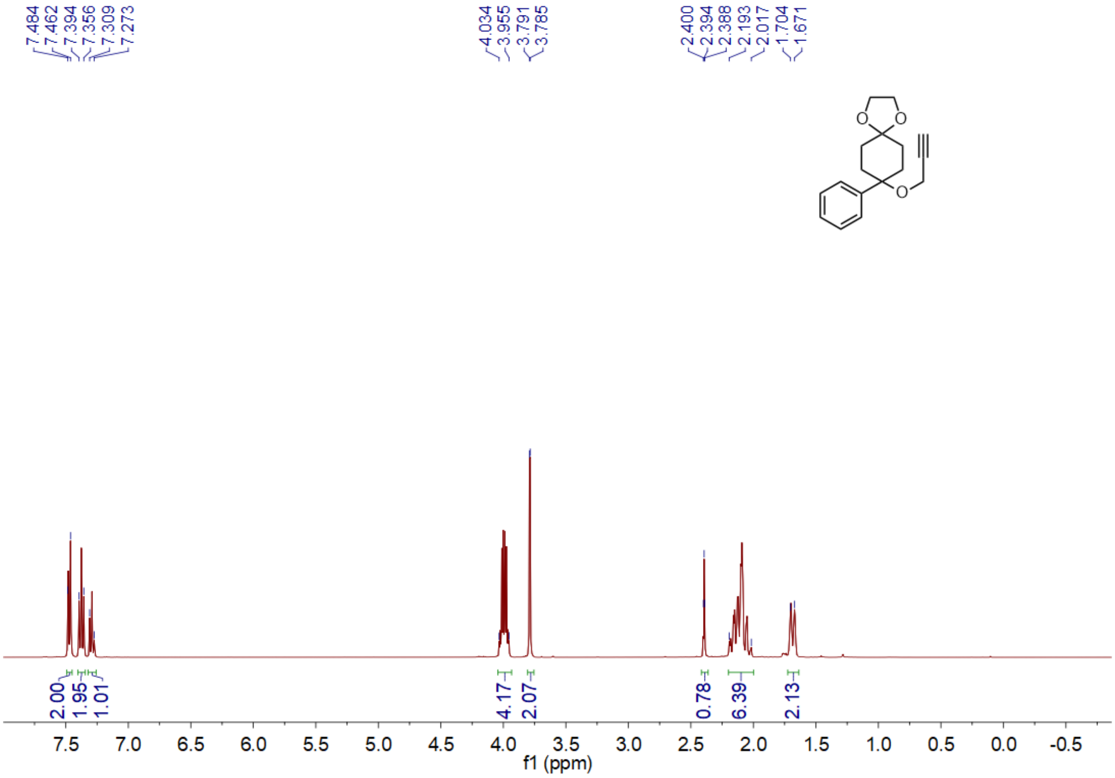
7.484
7.462
7.394
7.356
7.309
7.273
4.034
3.955
3.791
3.785
2.400
2.394
2.388
2.193
2.017
1.704
1.671
O
O
O
2.00
1.95
1.01
4.17
2.07
0.78
6.39
2.13
7.5 7.0 6.5 6.0 5.5 5.0 4.5 4.0 3.5 3.0 2.5 2.0 1.5 1.0 0.5 0.0 -0.5
f1 (ppm)

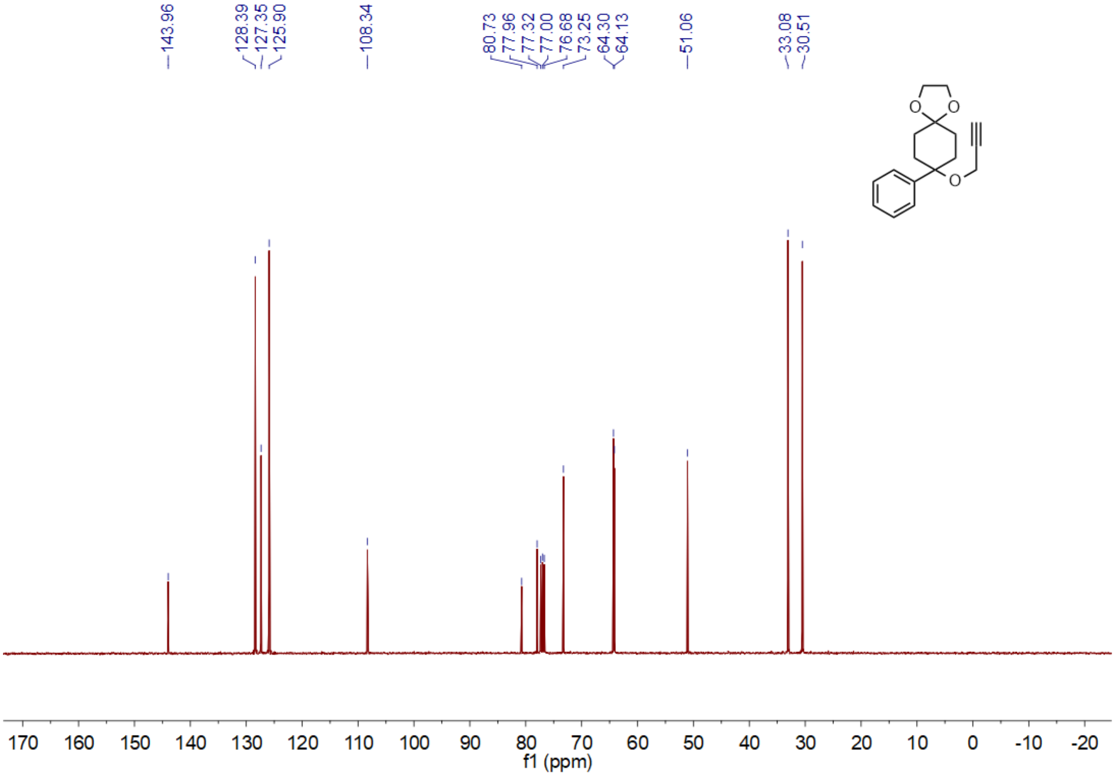
143.96
128.39
127.35
125.90
108.34
80.73
77.96
77.32
77.00
76.68
73.25
64.30
64.13
51.06
33.08
30.51
O
O
O
170 160 150 140 130 120 110 100 90 80 70 60 50 40 30 20 10 0 -10 -20
f1 (ppm)

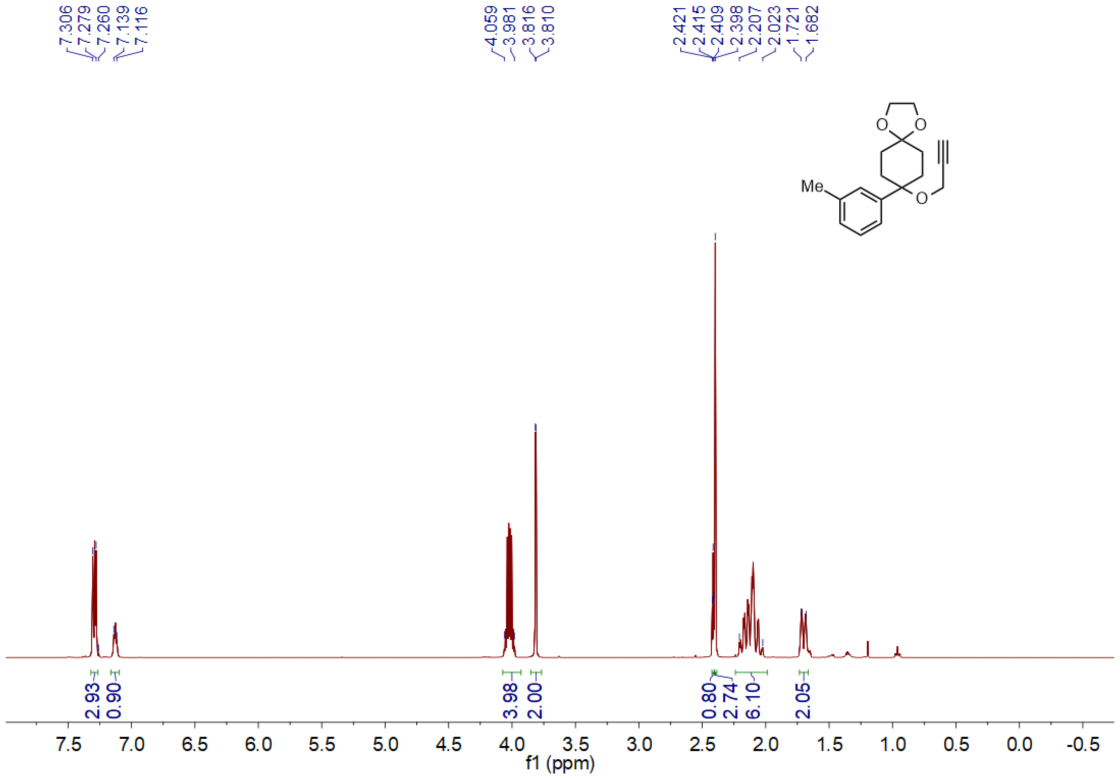
7.306
7.279
7.260
7.139
7.116
4.059
3.981
3.816
3.810
2.421
2.415
2.409
2.398
2.207
2.023
1.721
1.682
Me
O
O
O
2.93
0.90
3.98
2.00
0.80
2.74
6.10
2.05
7.5 7.0 6.5 6.0 5.5 5.0 4.5 4.0 3.5 3.0 2.5 2.0 1.5 1.0 0.5 0.0 -0.5
f1 (ppm)

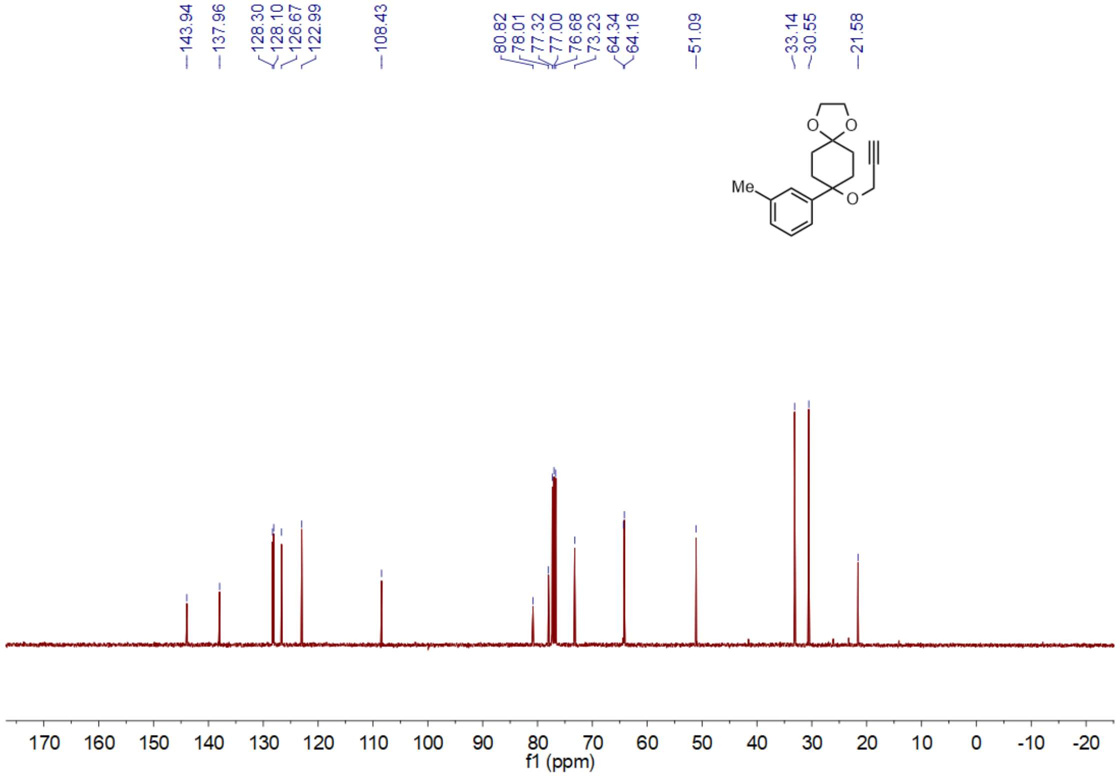
143.94
137.96
128.30
128.10
126.67
122.99
108.43
80.82
78.01
77.32
77.00
76.68
73.23
64.34
64.18
51.09
33.14
30.55
21.58
Me
O
O
O
170 160 150 140 130 120 110 100 90 80 70 60 50 40 30 20 10 0 -10 -20
f1 (ppm)

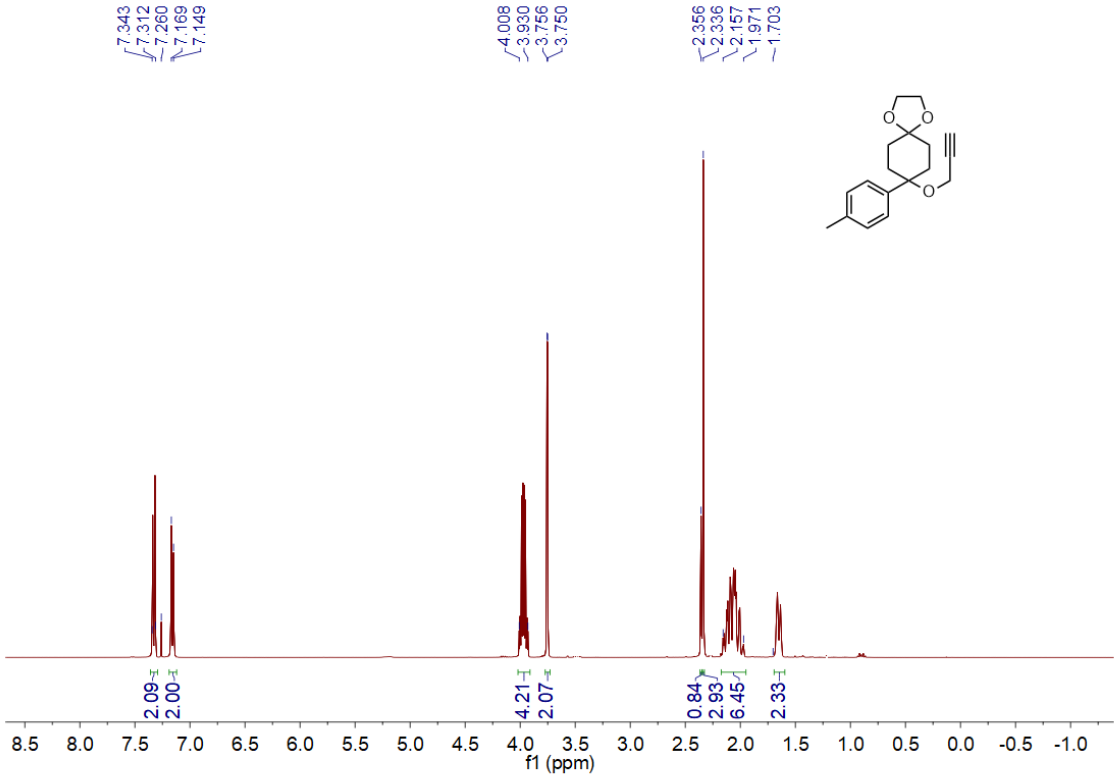
7.343
7.312
7.260
7.169
7.149
4.008
3.930
3.756
3.750
2.356
2.336
2.157
1.971
1.703
O
O
O
2.09
2.00
4.21
2.07
0.84
2.93
6.45
2.33
8.5 8.0 7.5 7.0 6.5 6.0 5.5 5.0 4.5 4.0 3.5 3.0 2.5 2.0 1.5 1.0 0.5 0.0 -0.5 -1.0
f1 (ppm)

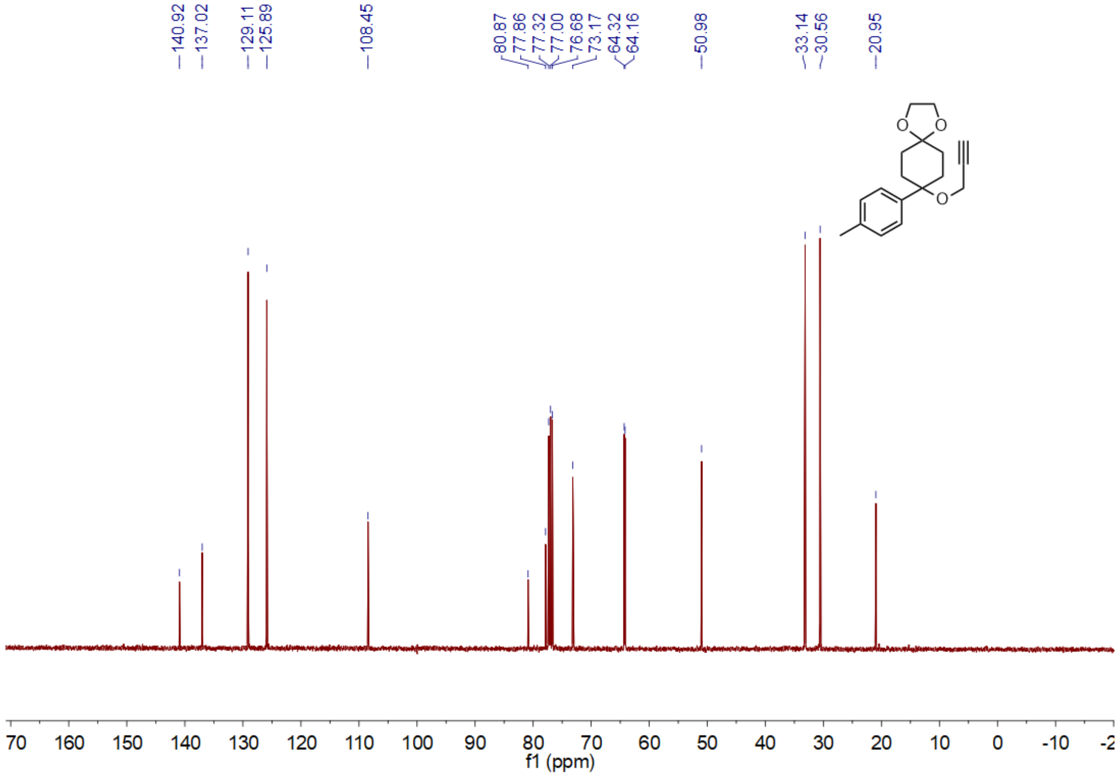
140.92
137.02
129.11
125.89
108.45
80.87
77.86
77.32
77.00
76.68
73.17
64.32
64.16
50.98
33.14
30.56
20.95
O
O
O
70 160 150 140 130 120 110 100 90 80 70 60 50 40 30 20 10 0 -10 -2
f1 (ppm)

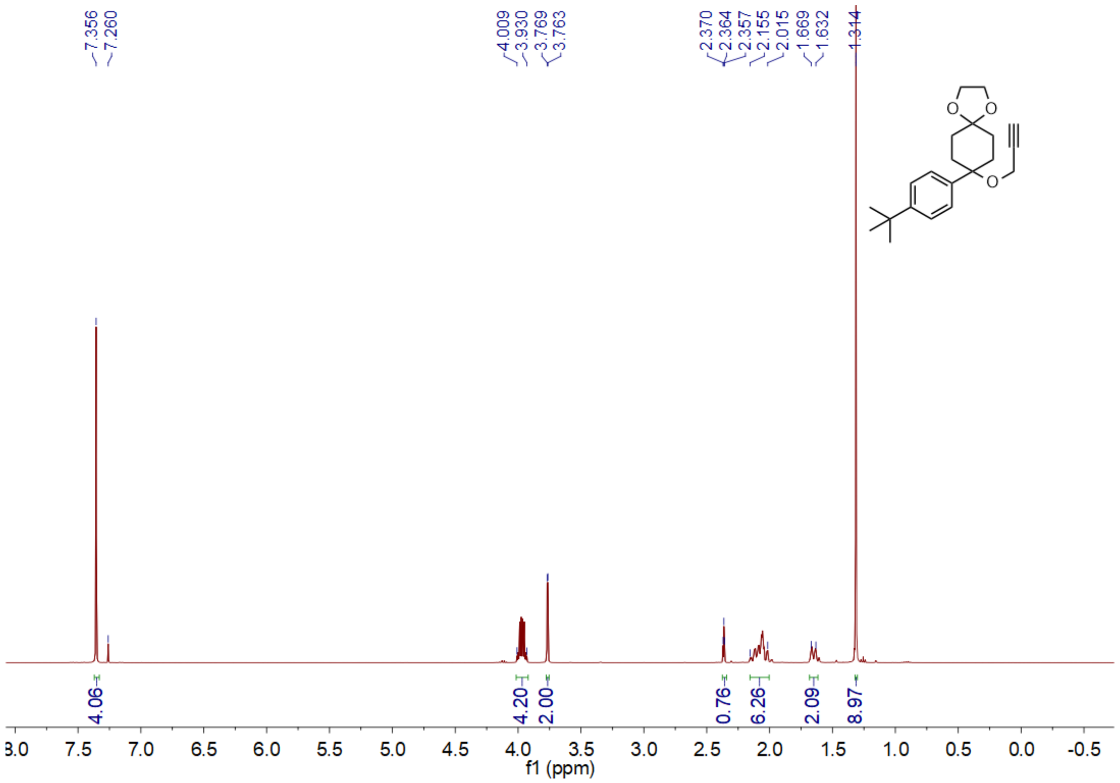
7.356
7.260
4.009
3.930
3.769
3.763
2.370
2.364
2.357
2.155
2.015
1.669
1.632
1.314
4.06
4.20
2.00
0.76
6.26
2.09
8.97
f1 (ppm)

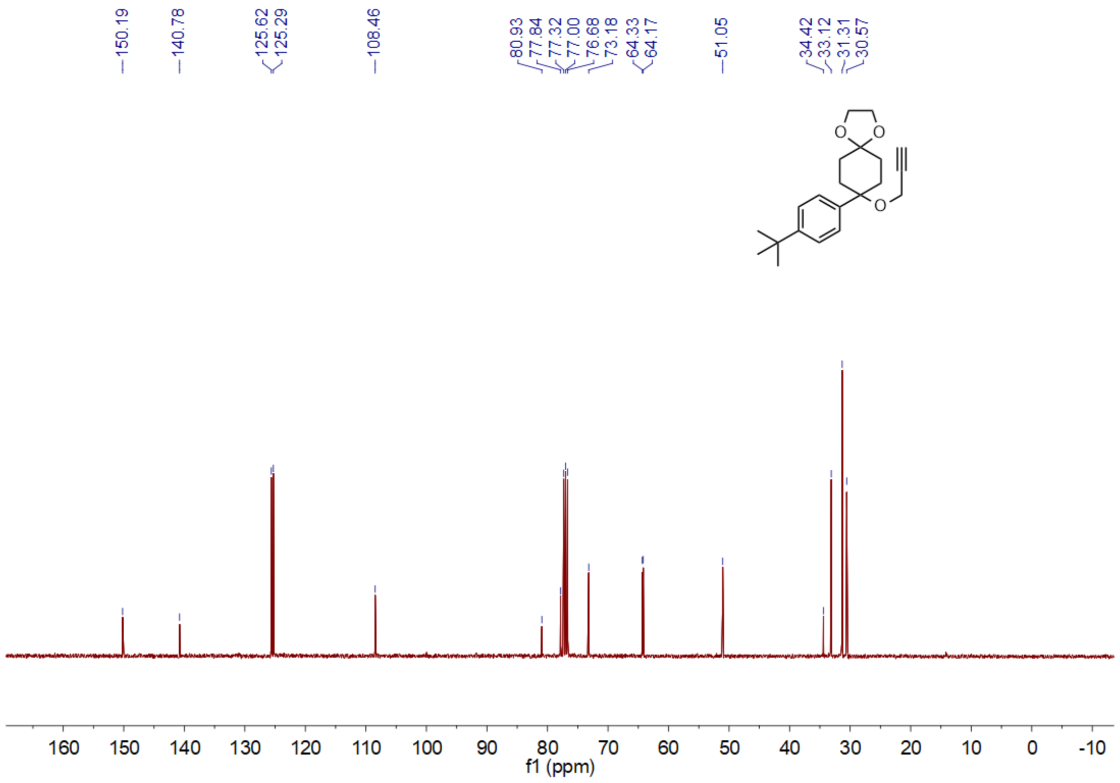
150.19
140.78
125.62
125.29
108.46
80.93
77.84
77.32
77.00
76.68
73.18
64.33
64.17
51.05
34.42
33.12
31.31
30.57
f1 (ppm)

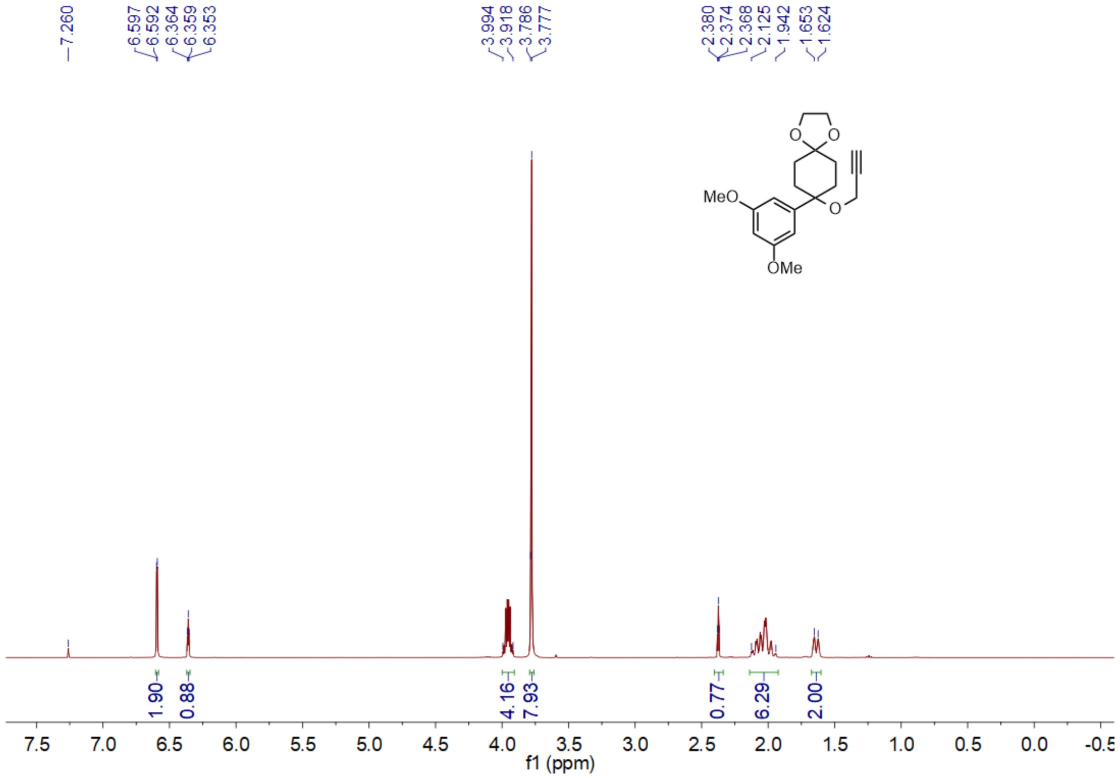
7.260
6.597
6.592
6.364
6.359
6.353
3.994
3.918
3.786
3.777
2.380
2.374
2.368
2.125
1.942
1.653
1.624
MeO
O
O
O
OMe
1.90
0.88
4.16
7.93
0.77
6.29
2.00
7.5 7.0 6.5 6.0 5.5 5.0 4.5 4.0 3.5 3.0 2.5 2.0 1.5 1.0 0.5 0.0 -0.5
f1 (ppm)

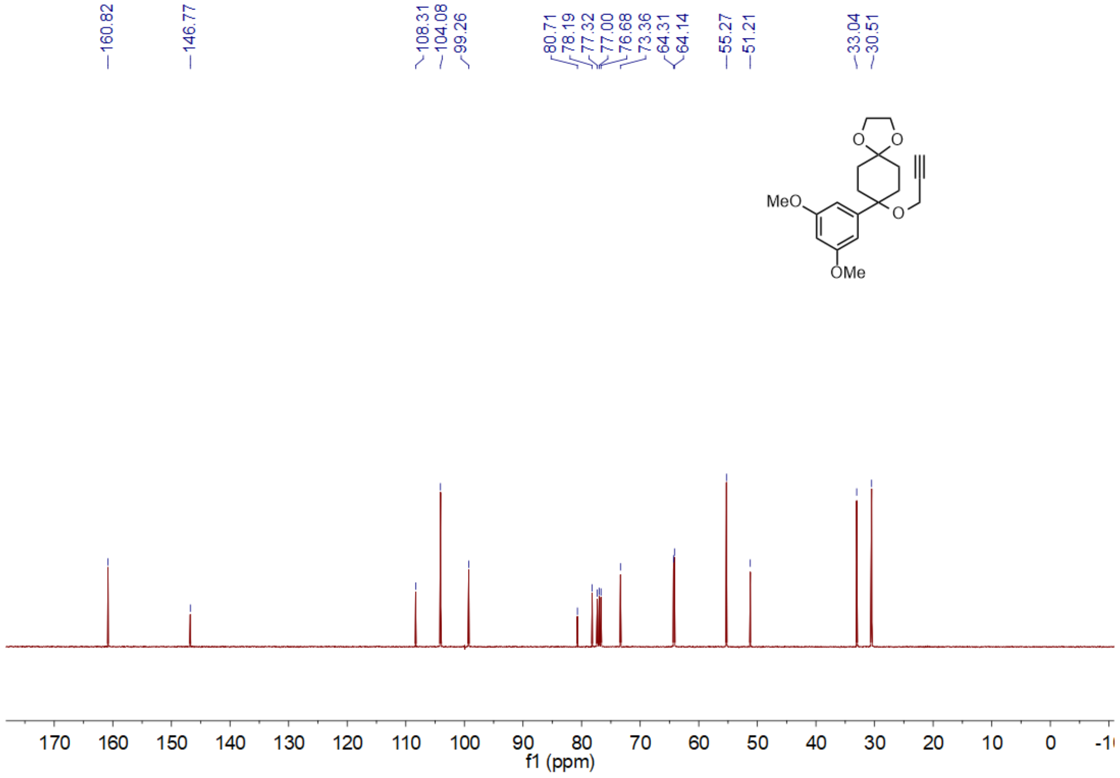
160.82
146.77
108.31
104.08
99.26
80.71
78.19
77.32
77.00
76.68
73.36
64.31
64.14
55.27
51.21
33.04
30.51
MeO
O
O
O
OMe
170 160 150 140 130 120 110 100 90 80 70 60 50 40 30 20 10 0 -10
f1 (ppm)

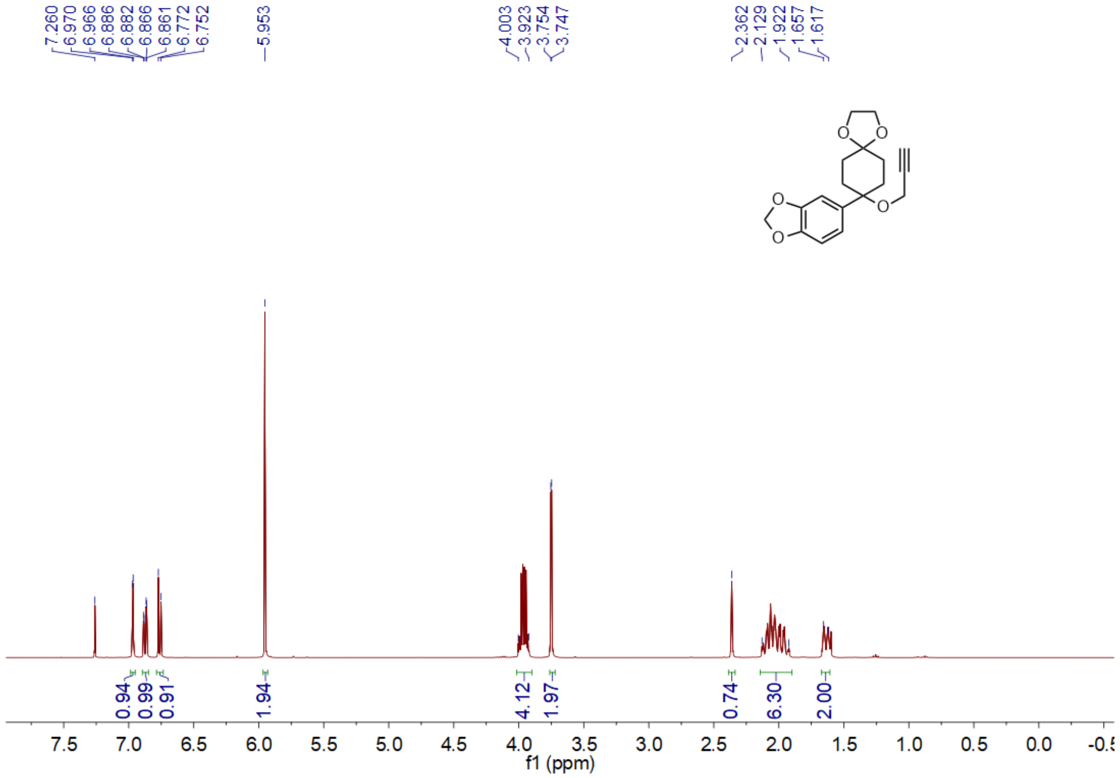
7.260
6.970
6.966
6.886
6.882
6.866
6.861
6.772
6.752
5.953
4.003
3.923
3.754
3.747
2.362
2.129
1.922
1.657
1.617
0.94
0.99
0.91
1.94
4.12
1.97
0.74
6.30
2.00
7.5 7.0 6.5 6.0 5.5 5.0 4.5 4.0 3.5 3.0 2.5 2.0 1.5 1.0 0.5 0.0 -0.5
f1 (ppm)

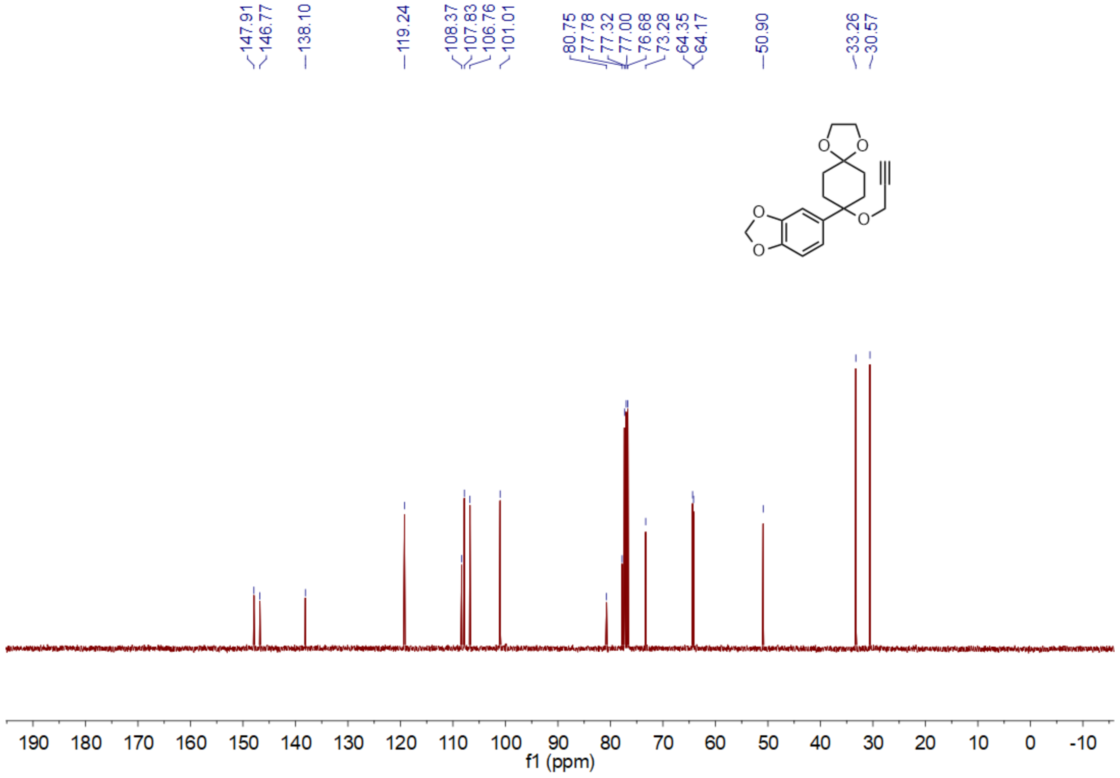
147.91
146.77
138.10
119.24
108.37
107.83
106.76
101.01
80.75
77.78
77.32
77.00
76.68
73.28
64.35
64.17
50.90
33.26
30.57
190 180 170 160 150 140 130 120 110 100 90 80 70 60 50 40 30 20 10 0 -10
f1 (ppm)

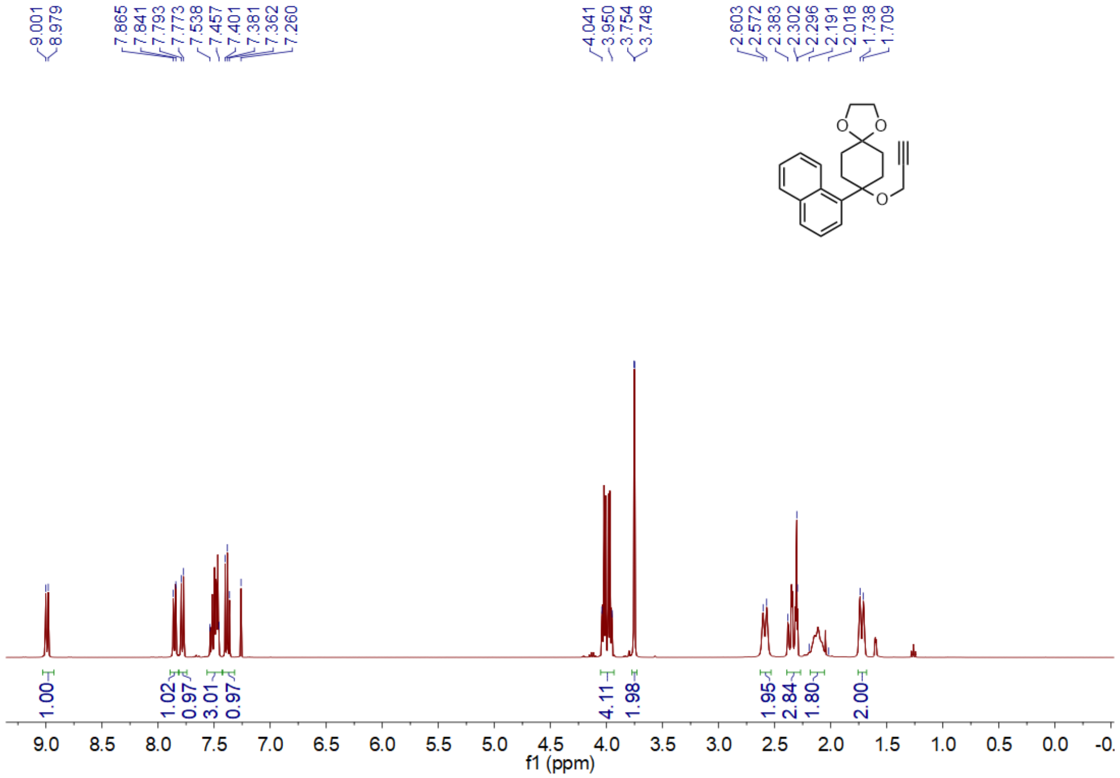
9.001
8.979
7.865
7.841
7.793
7.773
7.538
7.457
7.401
7.381
7.362
7.260
4.041
3.950
3.754
3.748
2.603
2.572
2.383
2.302
2.296
2.191
2.018
1.738
1.709
1.00
1.02
0.97
3.01
0.97
4.11
1.98
1.95
2.84
1.80
2.00
f1 (ppm)

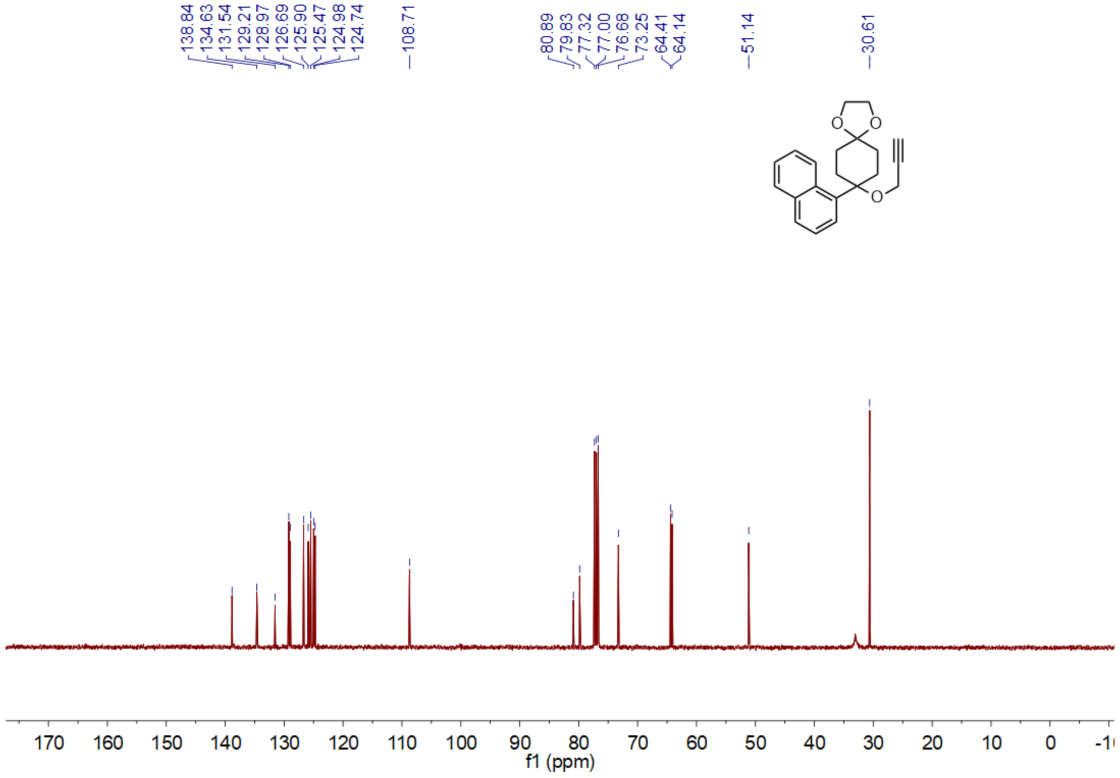
138.84
134.63
131.54
129.21
128.97
126.69
125.90
125.47
124.98
124.74
108.71
80.89
79.83
77.32
77.00
76.68
73.25
64.41
64.14
51.14
30.61
f1 (ppm)

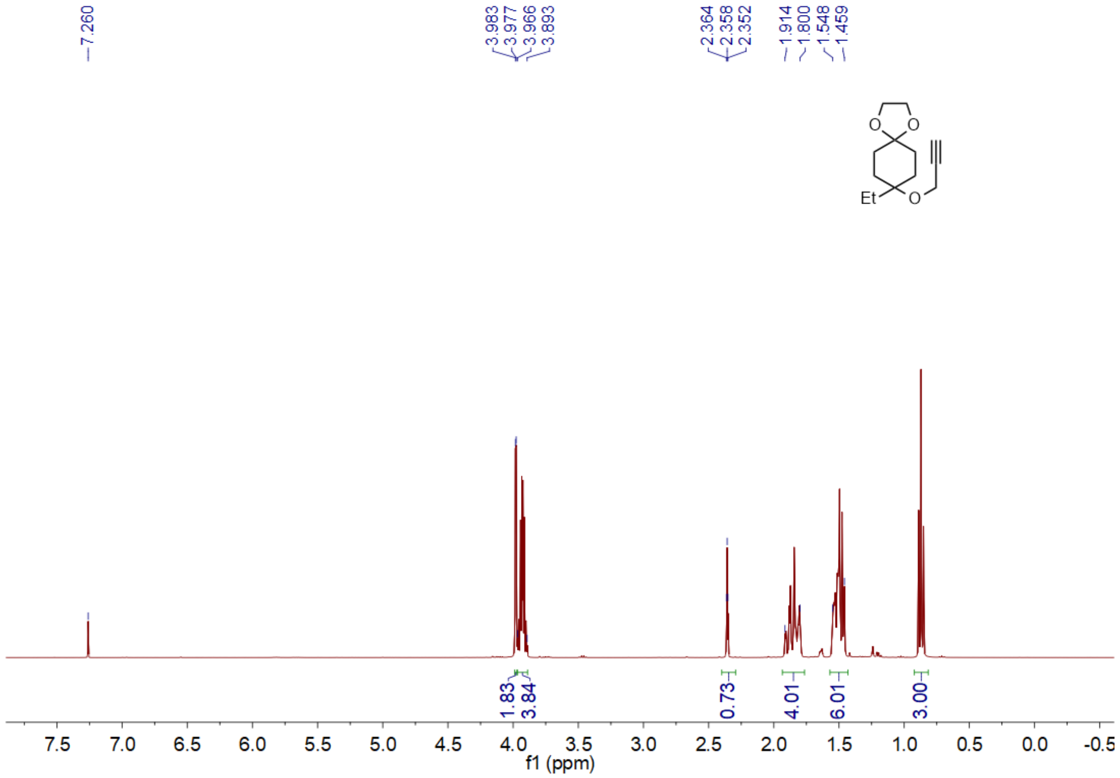
7.260
3.983
3.977
3.966
3.893
2.364
2.358
2.352
1.914
1.800
1.548
1.459
O
O
Et
O
1.83
3.84
0.73
4.01
6.01
3.00
7.5 7.0 6.5 6.0 5.5 5.0 4.5 4.0 3.5 3.0 2.5 2.0 1.5 1.0 0.5 0.0 -0.5
f1 (ppm)

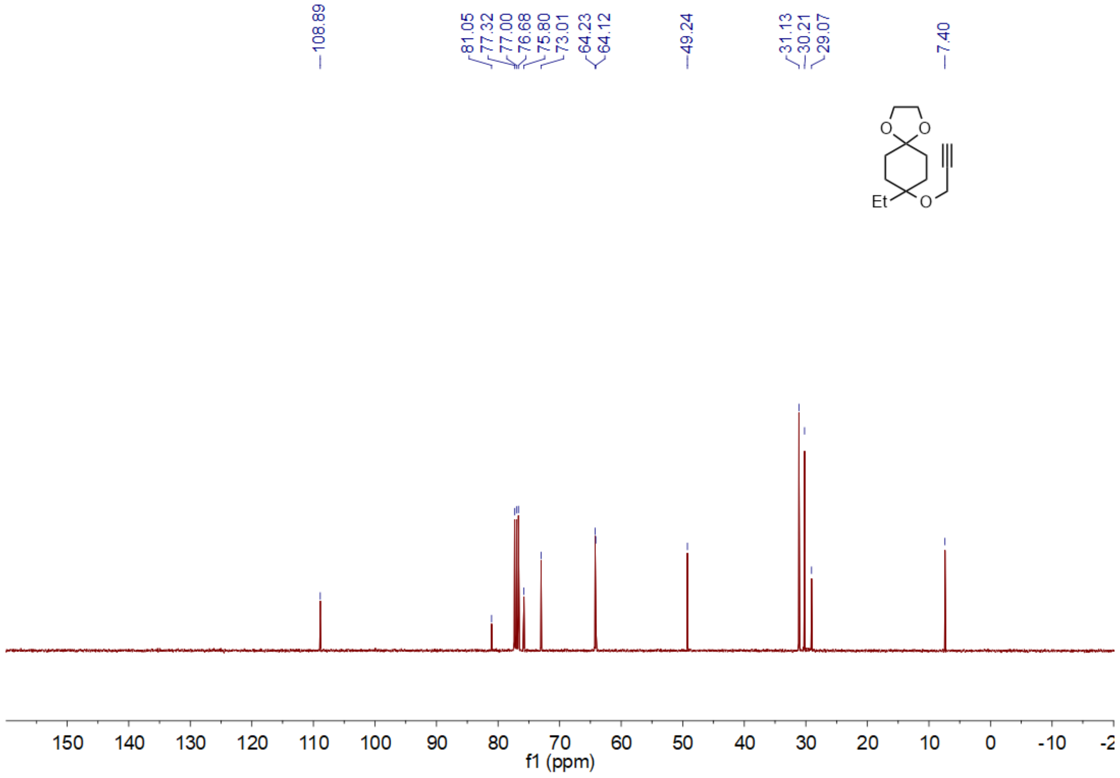
108.89
81.05
77.32
77.00
76.68
75.80
73.01
64.23
64.12
49.24
31.13
30.21
29.07
7.40
O
O
Et
O
150 140 130 120 110 100 90 80 70 60 50 40 30 20 10 0 -10
f1 (ppm)

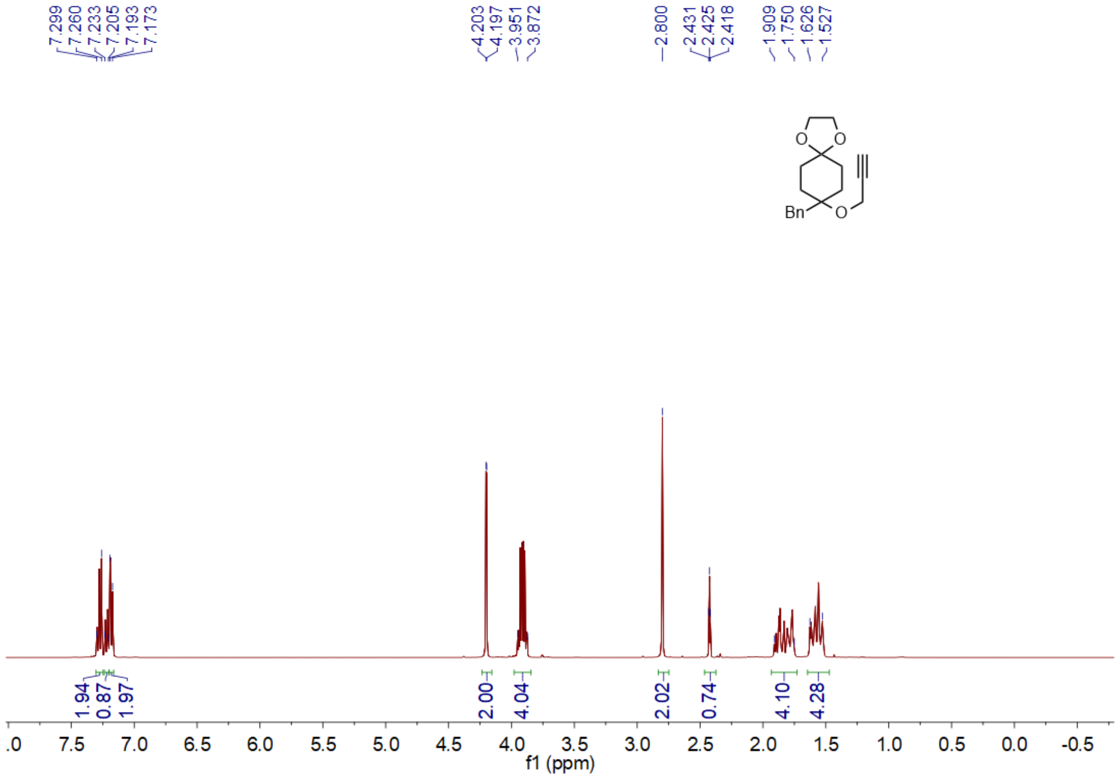

7.299
7.260
7.233
7.205
7.193
7.173
4.203
4.197
3.951
3.872
2.800
2.431
2.425
2.418
1.909
1.750
1.626
1.527
O
O
Bn
O
1.94
0.87
1.97
2.00
4.04
2.02
0.74
4.10
4.28
f1 (ppm)

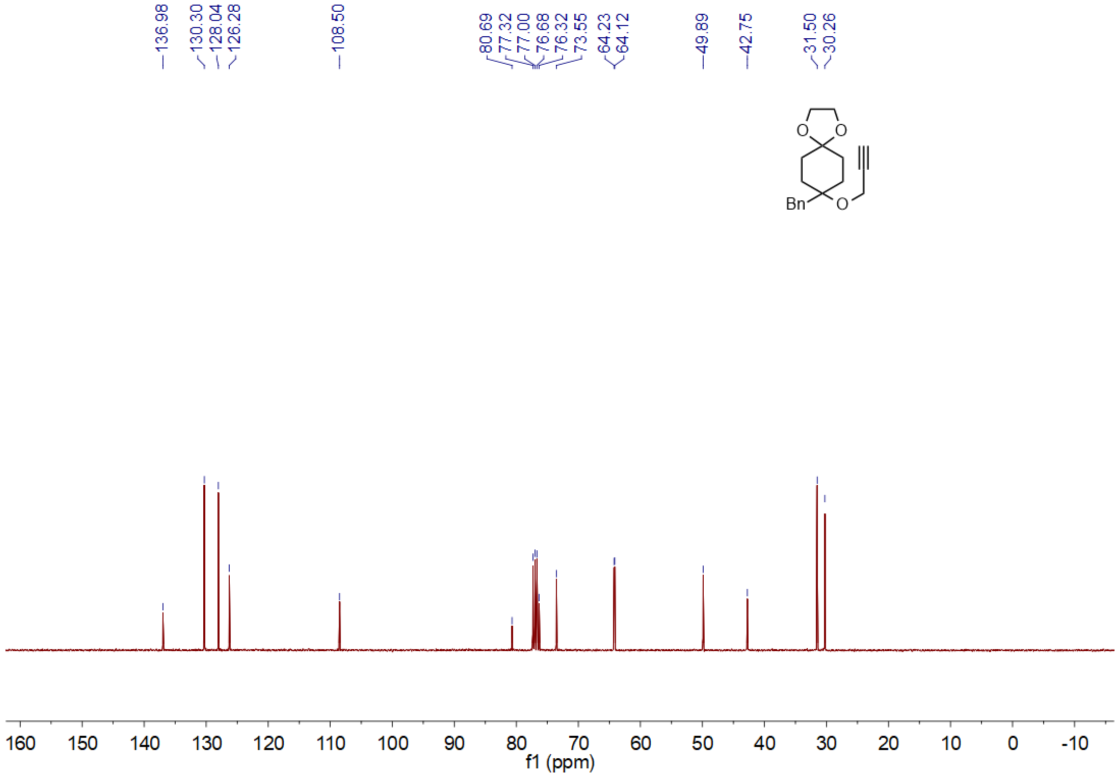

136.98
130.30
128.04
126.28
108.50
80.69
77.32
77.00
76.68
76.32
73.55
64.23
64.12
49.89
42.75
31.50
30.26
O
O
Bn
O
f1 (ppm)

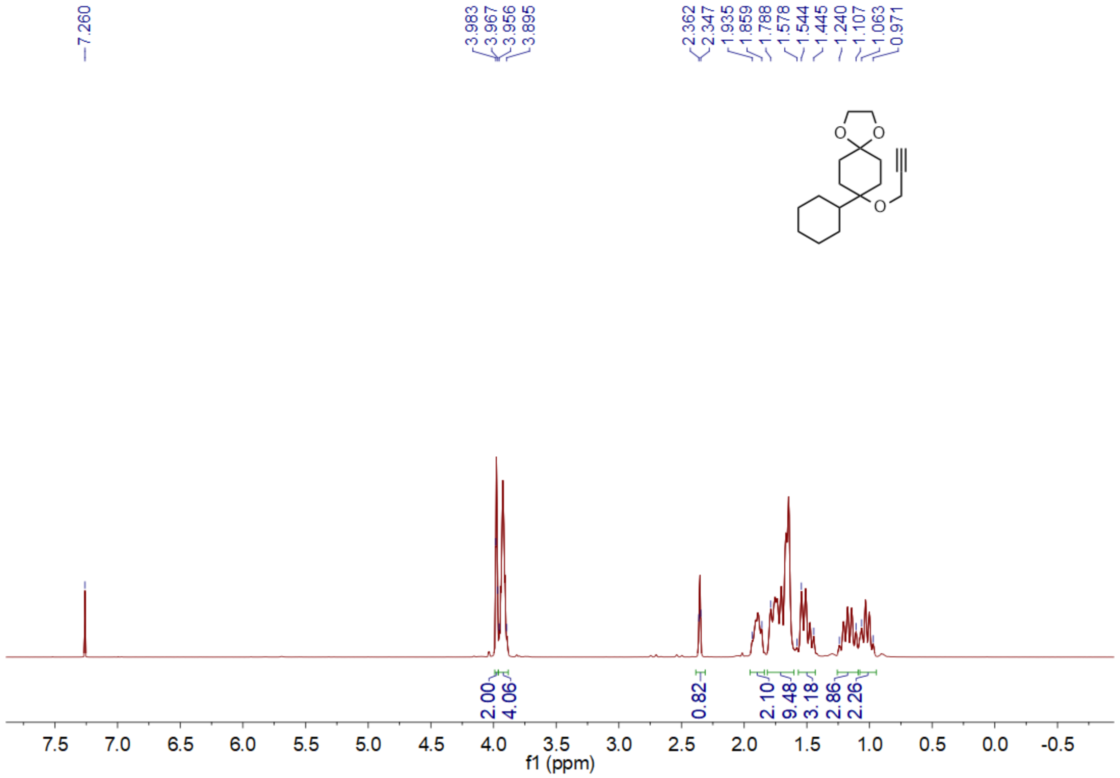
7.260
3.983
3.967
3.956
3.895
2.362
2.347
1.935
1.859
1.788
1.578
1.544
1.445
1.240
1.107
1.063
0.971
O
O
O
2.00
4.06
0.82
2.10
9.48
3.18
2.86
2.26
7.5 7.0 6.5 6.0 5.5 5.0 4.5 4.0 3.5 3.0 2.5 2.0 1.5 1.0 0.5 0.0 -0.5
f1 (ppm)

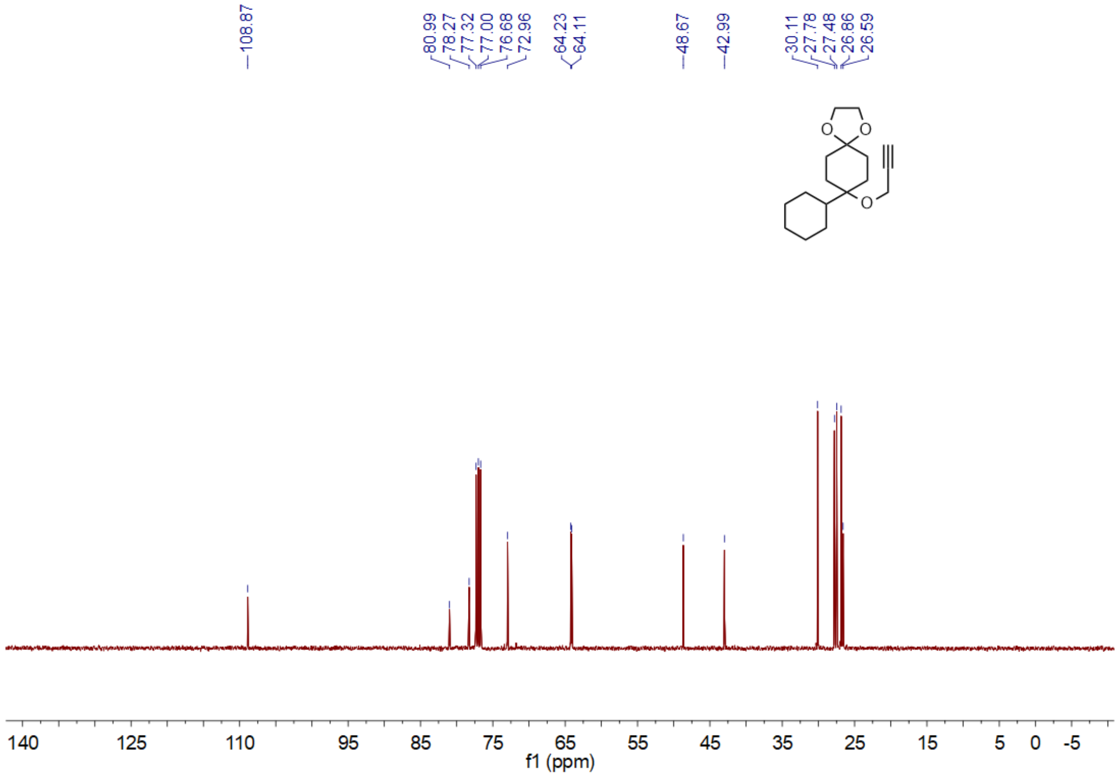
108.87
80.99
78.27
77.32
77.00
76.68
72.96
64.23
64.11
48.67
42.99
30.11
27.78
27.48
26.86
26.59
O
O
O
140 125 110 95 85 75 65 55 45 35 25 15 5 0 -5
f1 (ppm)

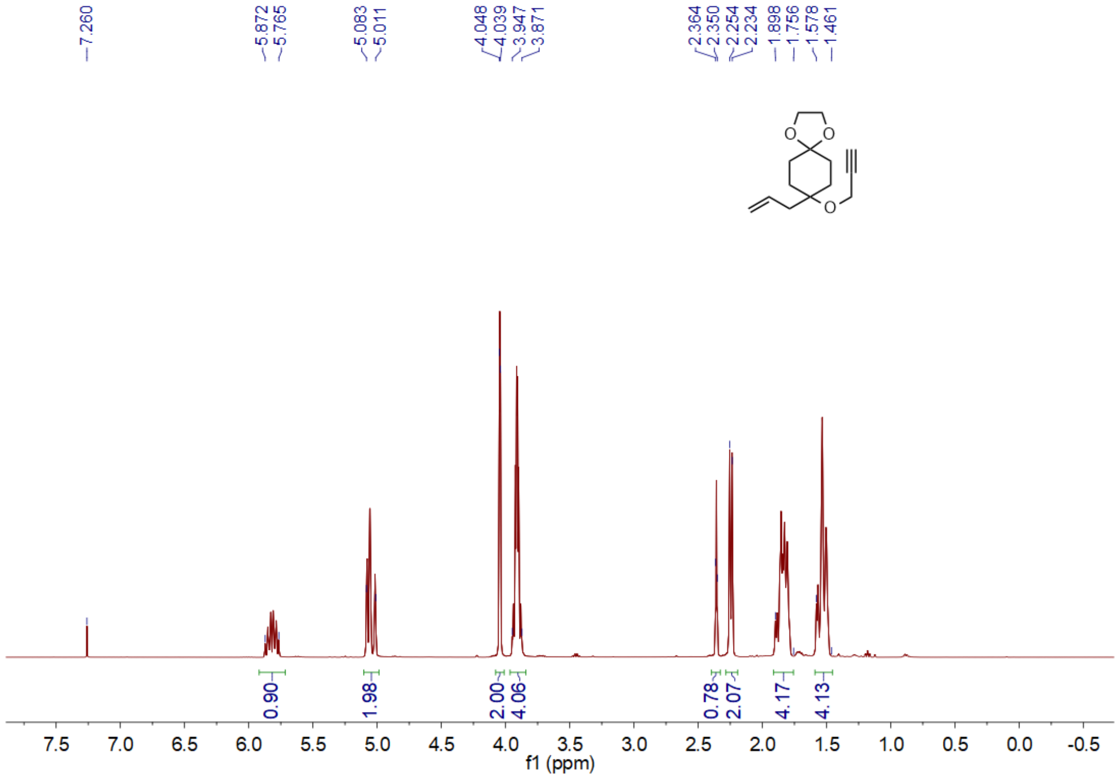
7.260
5.872
5.765
5.083
5.011
4.048
4.039
3.947
3.871
2.364
2.350
2.254
2.234
1.898
1.756
1.578
1.461
0.90
1.98
2.00
4.06
0.78
2.07
4.17
4.13
7.5
7.0
6.5
6.0
5.5
5.0
4.5
4.0
3.5
3.0
2.5
2.0
1.5
1.0
0.5
0.0
-0.5
f1 (ppm)

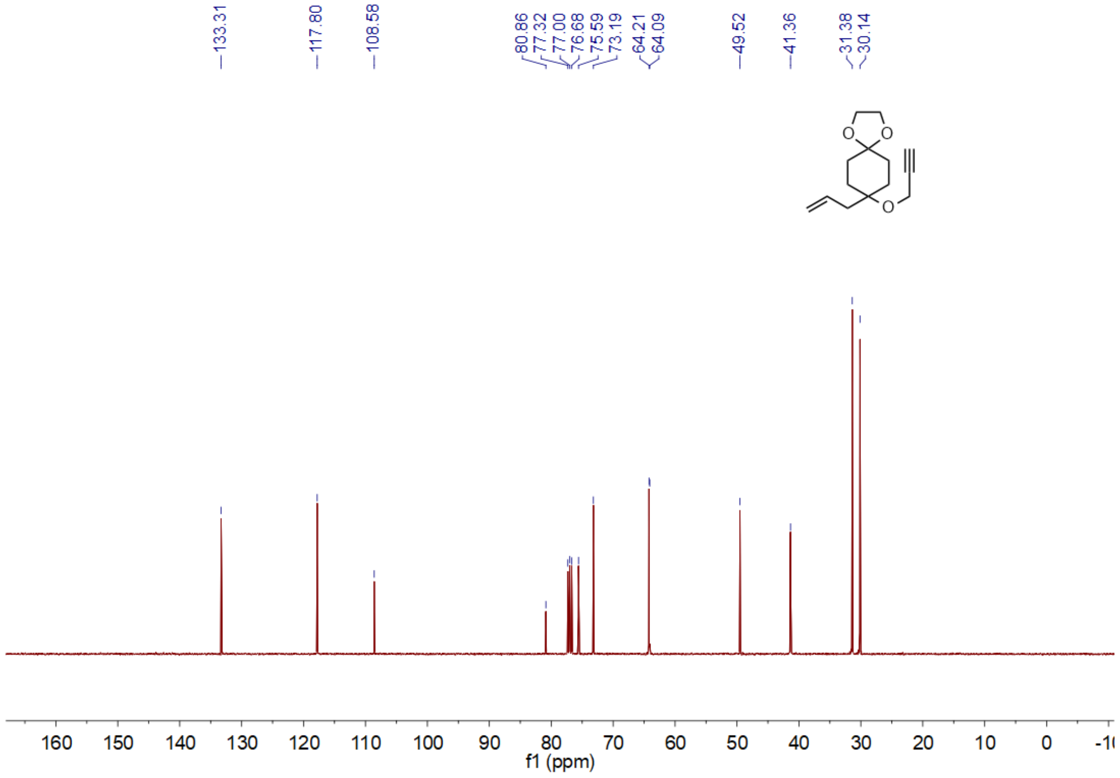
133.31
117.80
108.58
80.86
77.32
77.00
76.68
75.59
73.19
64.21
64.09
49.52
41.36
31.38
30.14
160
150
140
130
120
110
100
90
80
70
60
50
40
30
20
10
0
f1 (ppm)

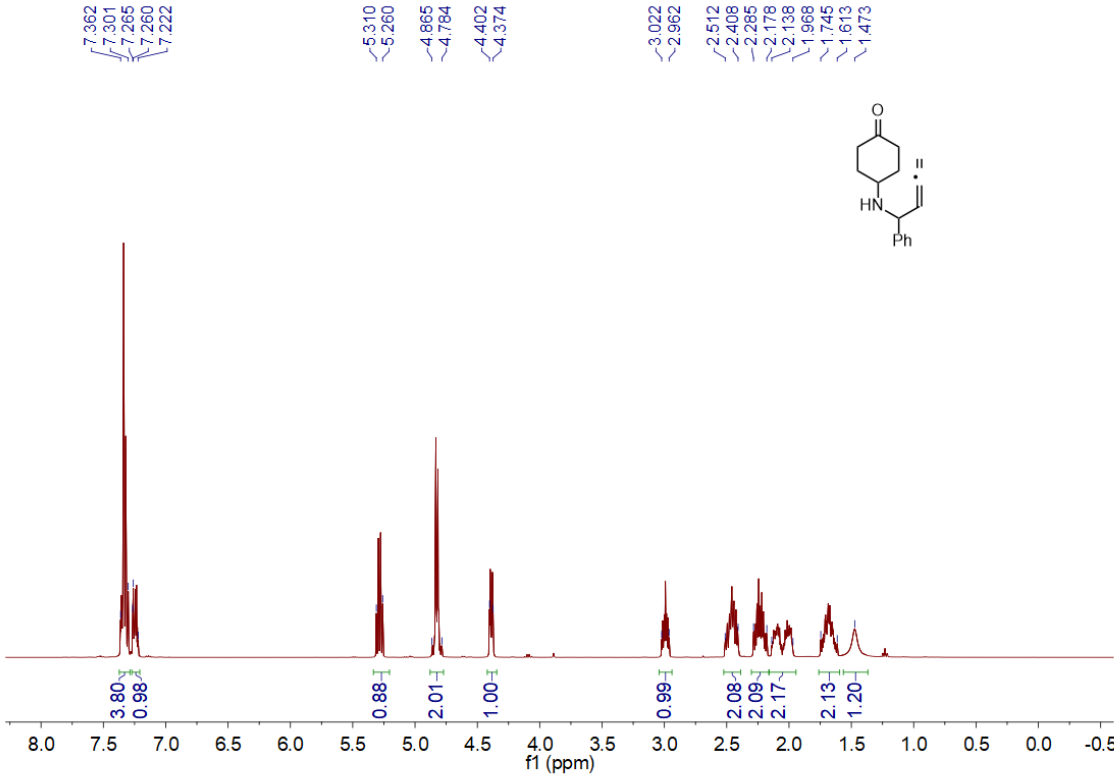
7.362
7.301
7.265
7.260
7.222
5.310
5.260
4.865
4.784
4.402
4.374
3.022
2.962
2.512
2.408
2.285
2.178
2.138
1.968
1.745
1.613
1.473
O
HN
Ph
3.80
0.98
0.88
2.01
1.00
0.99
2.08
2.09
2.17
2.13
1.20
8.0 7.5 7.0 6.5 6.0 5.5 5.0 4.5 4.0 3.5 3.0 2.5 2.0 1.5 1.0 0.5 0.0 -0.5
f1 (ppm)

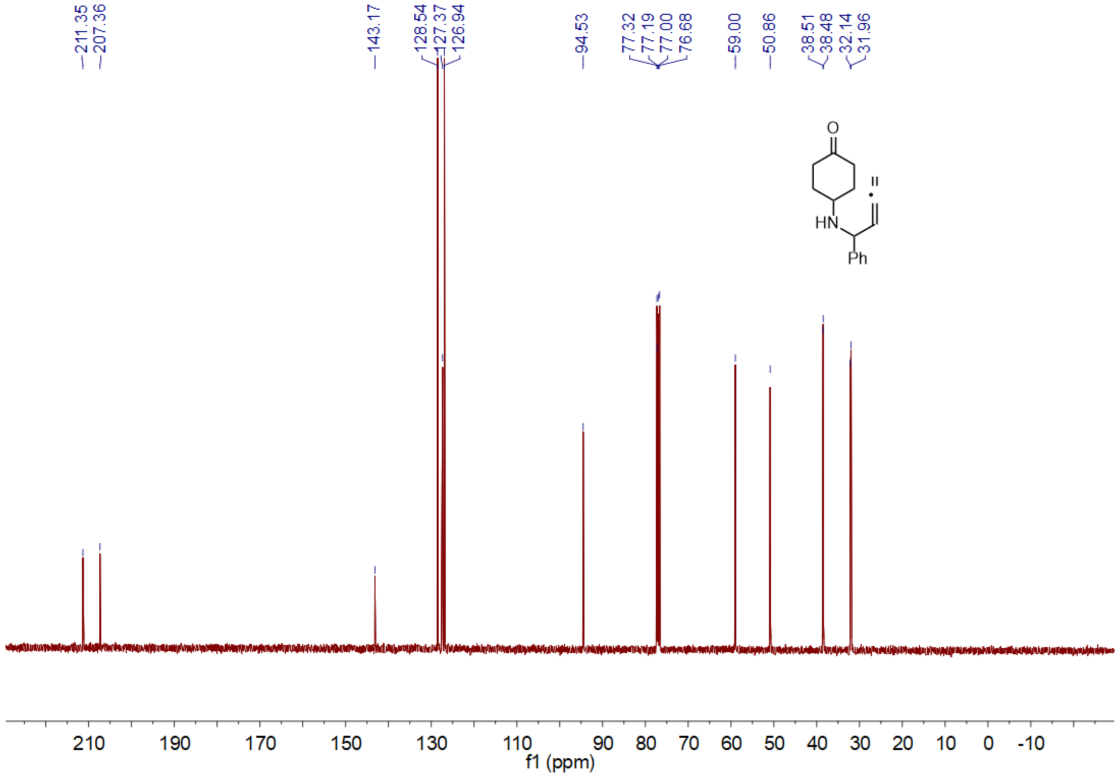
211.35
207.36
143.17
128.54
127.37
126.94
94.53
77.32
77.19
77.00
76.68
59.00
50.86
38.51
38.48
32.14
31.96
O
HN
Ph
210 190 170 150 130 110 90 80 70 60 50 40 30 20 10 0 -10
f1 (ppm)

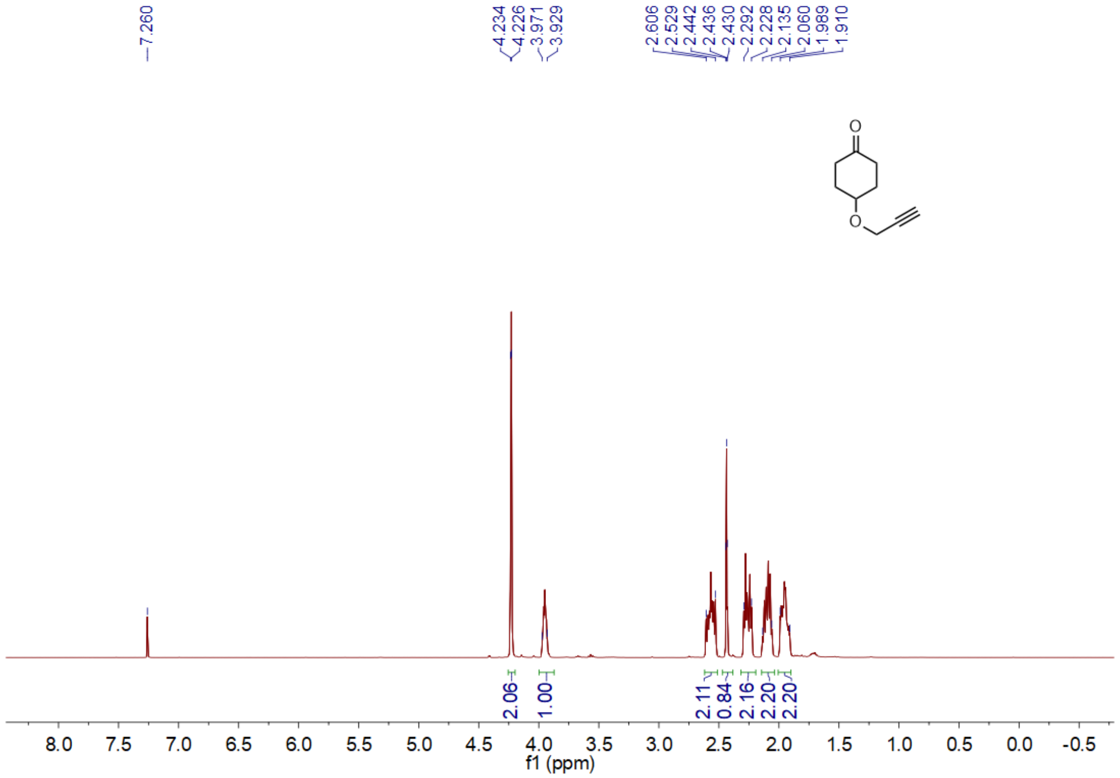

7.260
4.234
4.226
3.971
3.929
2.606
2.529
2.442
2.436
2.430
2.292
2.228
2.135
2.060
1.989
1.910
2.06
1.00
2.11
0.84
2.16
2.20
2.20
f1 (ppm)

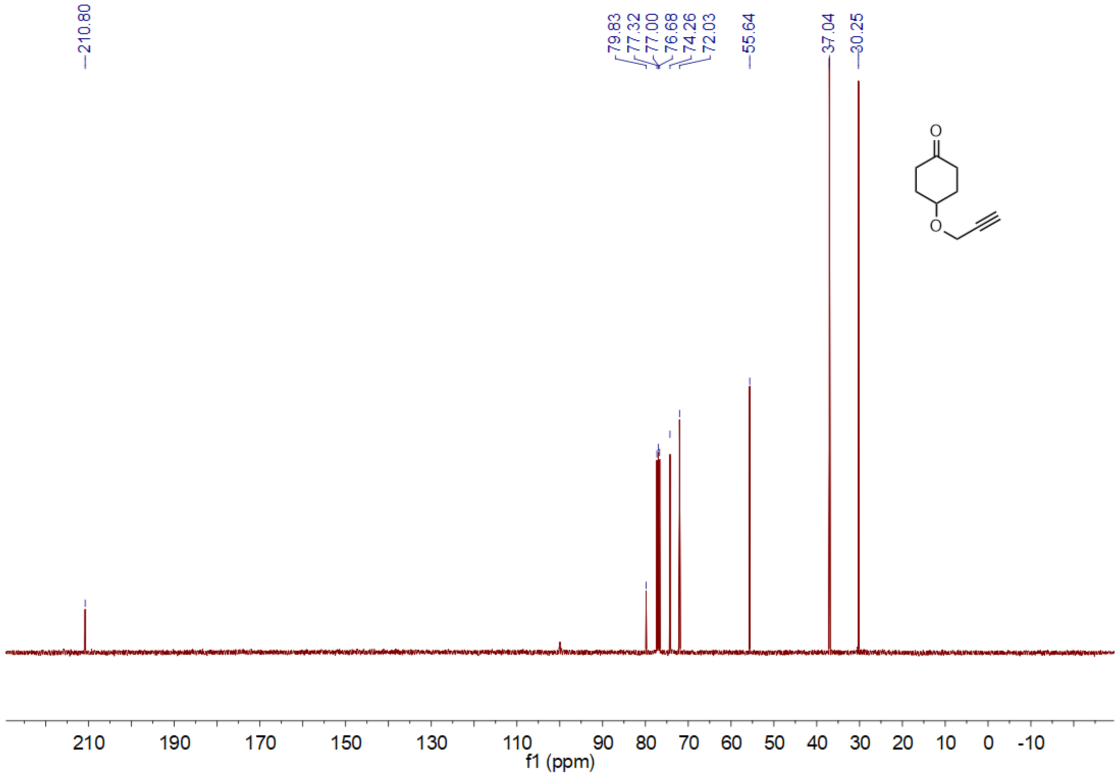

210.80
79.83
77.32
77.00
76.68
74.26
72.03
55.64
37.04
30.25
f1 (ppm)

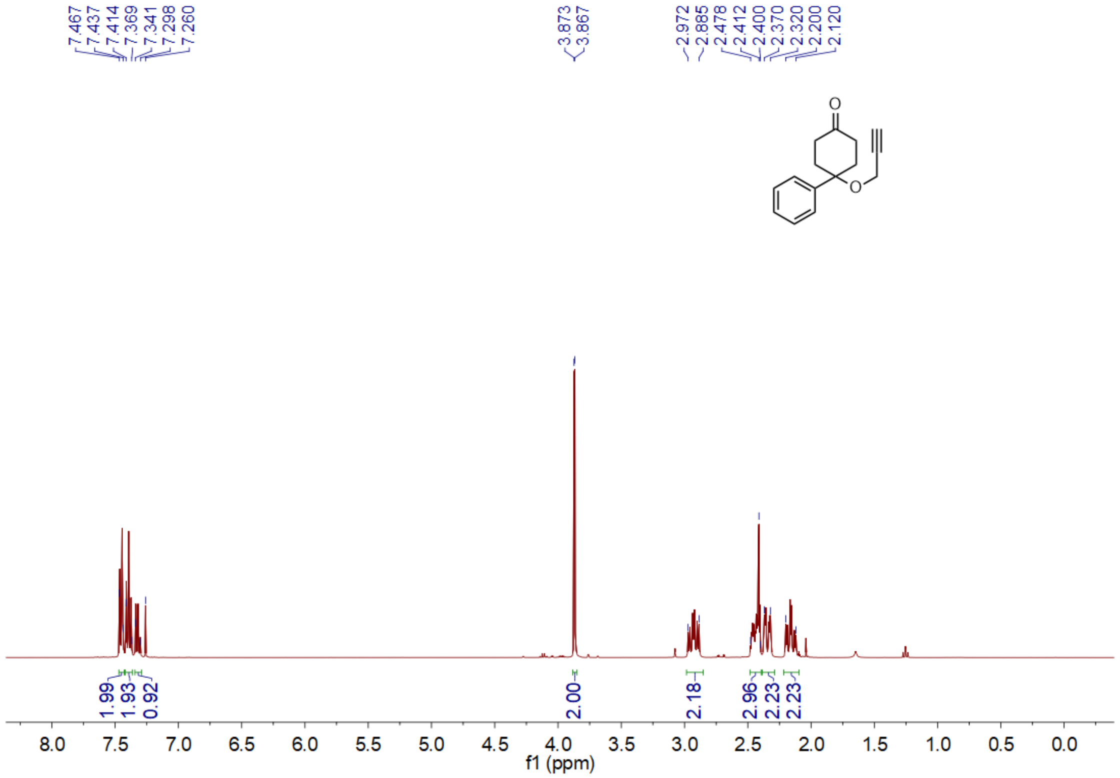
7.467
7.437
7.414
7.369
7.341
7.298
7.260
3.873
3.867
2.972
2.885
2.478
2.412
2.400
2.370
2.320
2.200
2.120
O
O
1.99
1.93
0.92
2.00
2.18
2.96
2.23
2.23
8.0 7.5 7.0 6.5 6.0 5.5 5.0 4.5 4.0 3.5 3.0 2.5 2.0 1.5 1.0 0.5 0.0
f1 (ppm)

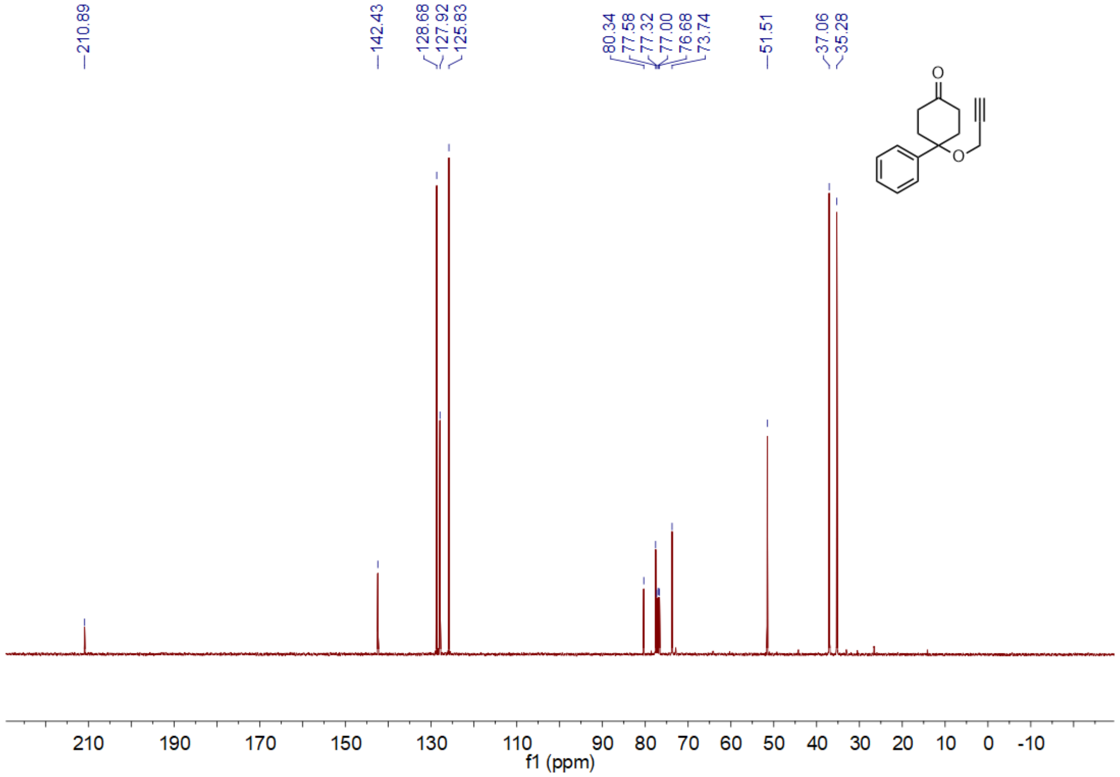
210.89
142.43
128.68
127.92
125.83
80.34
77.58
77.32
77.00
76.68
73.74
51.51
37.06
35.28
O
O
210 190 170 150 130 110 90 80 70 60 50 40 30 20 10 0 -10
f1 (ppm)

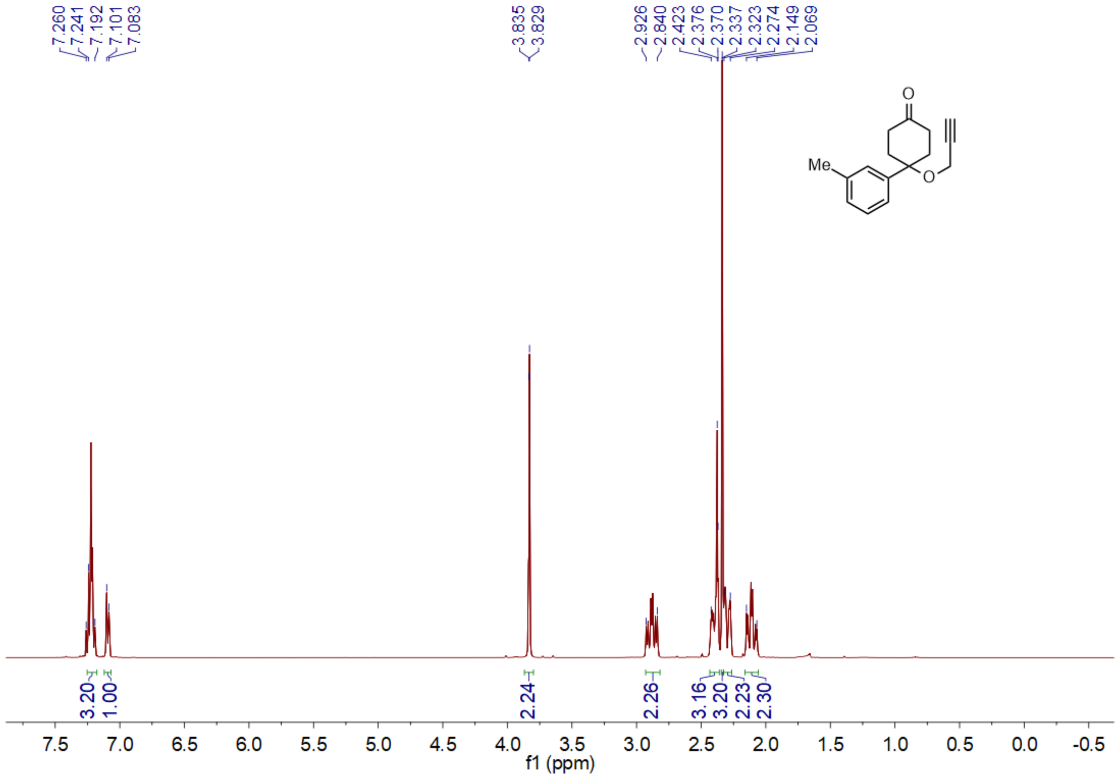
7.260
7.241
7.192
7.101
7.083
3.835
3.829
2.926
2.840
2.423
2.376
2.370
2.337
2.323
2.274
2.149
2.069
Me
O
3.20
1.00
2.24
2.26
3.16
3.20
2.23
2.30
f1 (ppm)

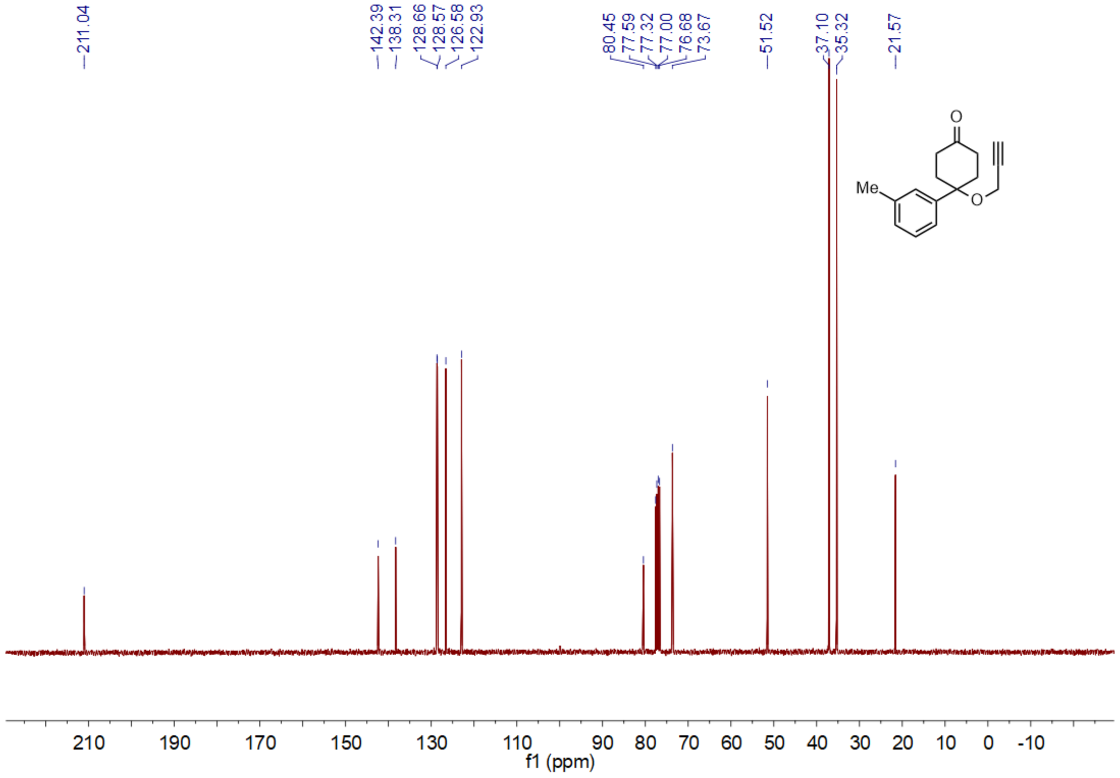
211.04
142.39
138.31
128.66
128.57
126.58
122.93
80.45
77.59
77.32
77.00
76.68
73.67
51.52
37.10
35.32
21.57
Me
O
f1 (ppm)

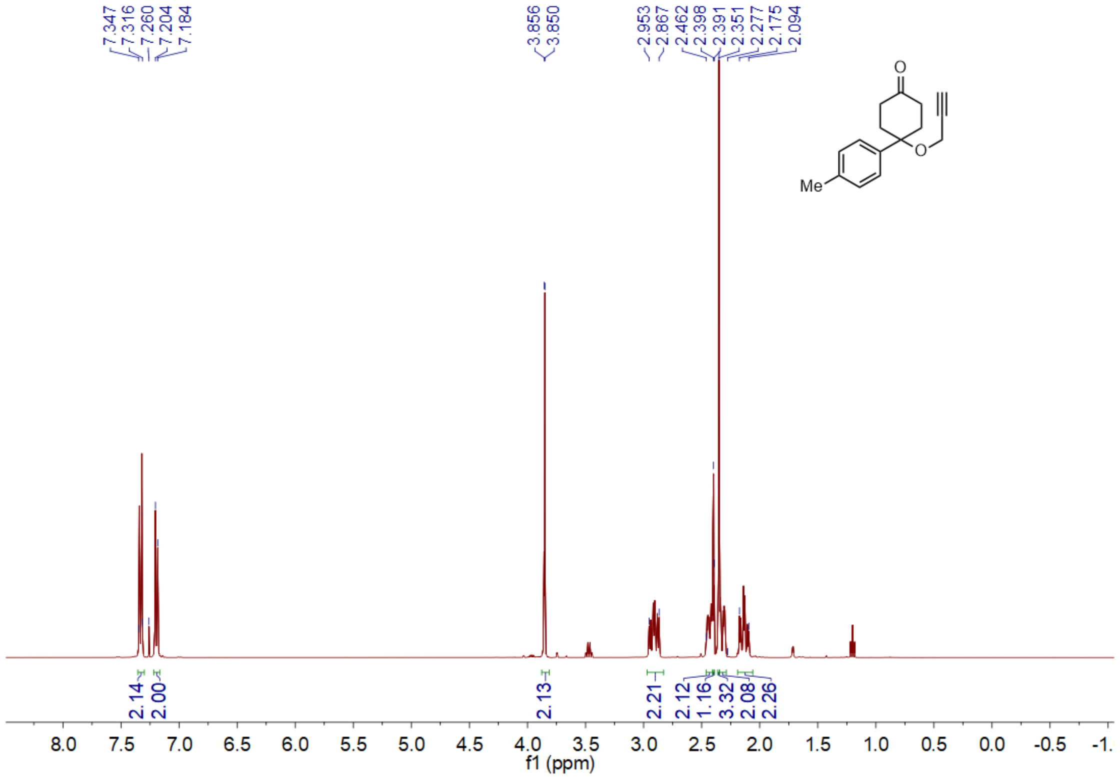
7.347
7.316
7.260
7.204
7.184
3.856
3.850
2.953
2.867
2.462
2.398
2.391
2.351
2.277
2.175
2.094
O
O
Me
2.14
2.00
2.13
2.21
2.12
1.16
3.32
2.08
2.26
f1 (ppm)

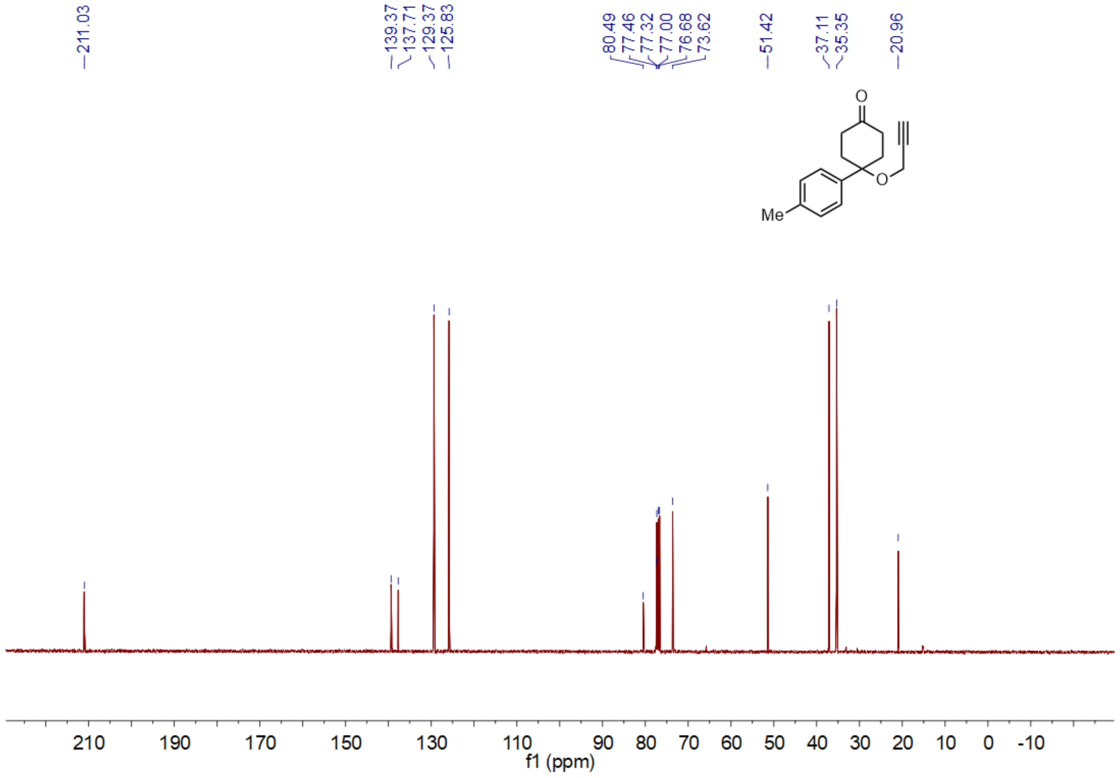
211.03
139.37
137.71
129.37
125.83
80.49
77.46
77.32
77.00
76.68
73.62
51.42
37.11
35.35
20.96
O
O
Me
f1 (ppm)

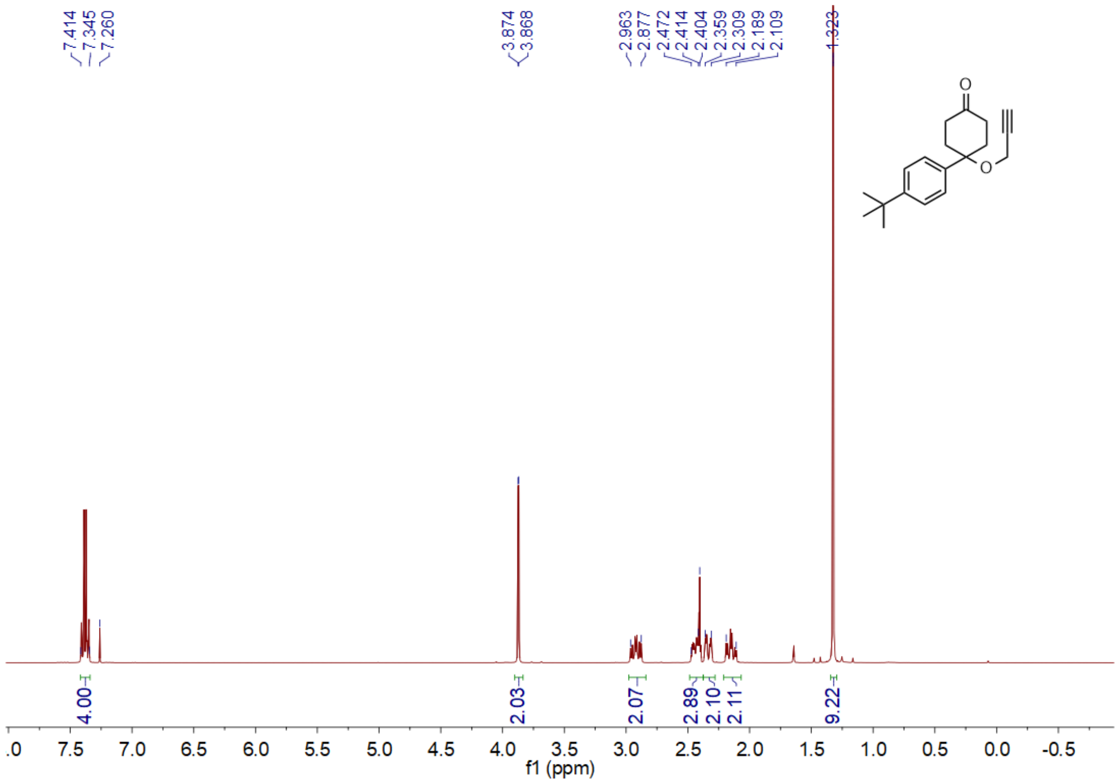
7.414
7.345
7.260
3.874
3.868
2.963
2.877
2.472
2.414
2.404
2.359
2.309
2.189
2.109
1.323
O
O
4.00
2.03
2.07
2.89
2.10
2.11
9.22
f1 (ppm)

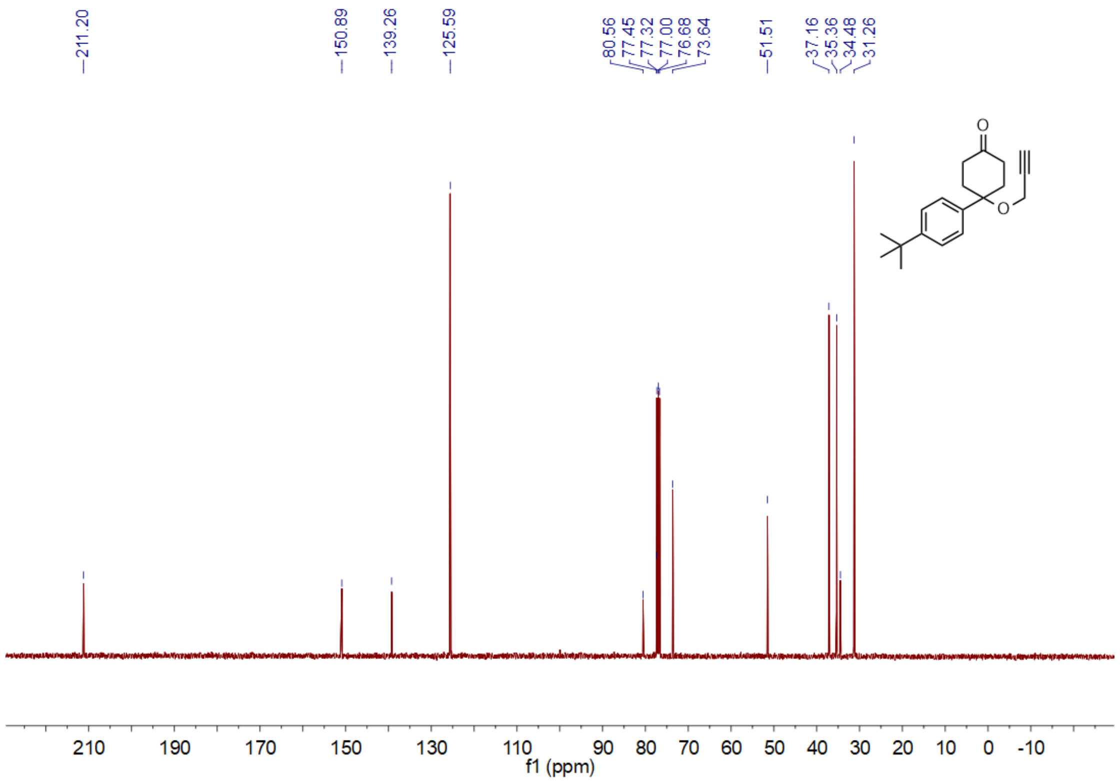
211.20
150.89
139.26
125.59
80.56
77.45
77.32
77.00
76.68
73.64
51.51
37.16
35.36
34.48
31.26
O
O
f1 (ppm)

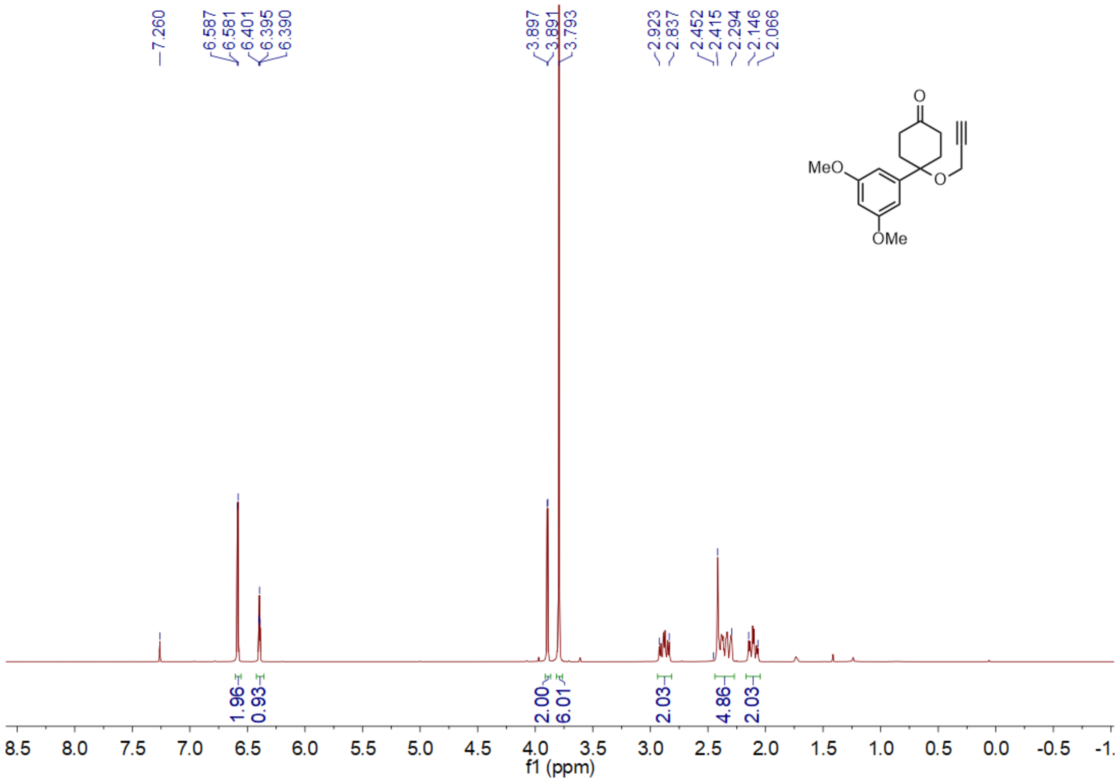
7.260
6.587
6.581
6.401
6.395
6.390
3.897
3.891
3.793
2.923
2.837
2.452
2.415
2.294
2.146
2.066
MeO
OMe
O
1.96
0.93
2.00
6.01
2.03
4.86
2.03
8.5 8.0 7.5 7.0 6.5 6.0 5.5 5.0 4.5 4.0 3.5 3.0 2.5 2.0 1.5 1.0 0.5 0.0 -0.5 -1.
f1 (ppm)

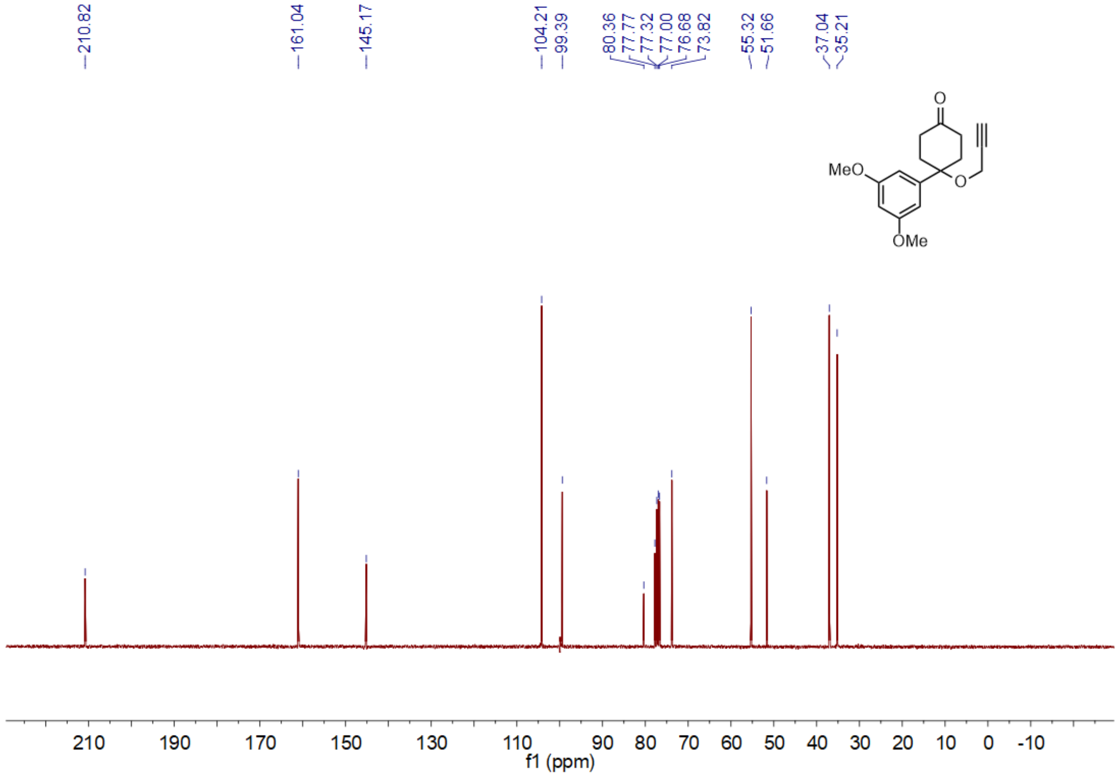
210.82
161.04
145.17
104.21
99.39
80.36
77.77
77.32
77.00
76.68
73.82
55.32
51.66
37.04
35.21
MeO
OMe
O
210 190 170 150 130 110 90 80 70 60 50 40 30 20 10 0 -10
f1 (ppm)

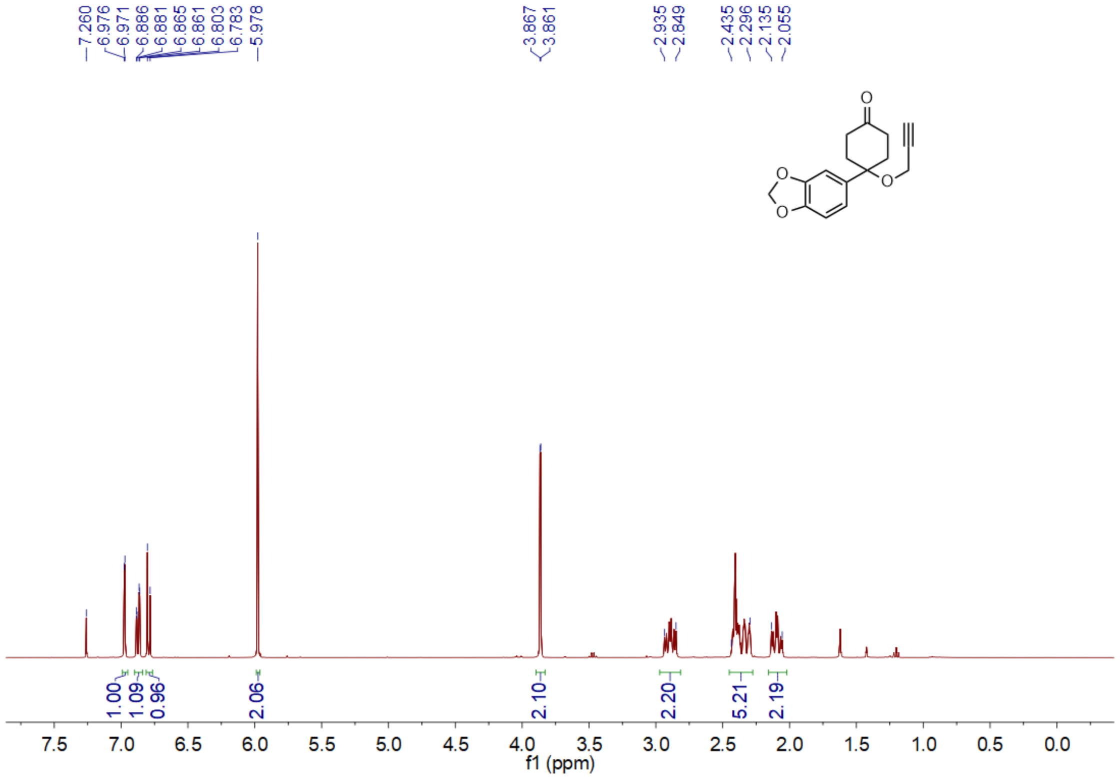
7.260
6.976
6.971
6.886
6.881
6.865
6.861
6.803
6.783
5.978
3.867
3.861
2.935
2.849
2.435
2.296
2.135
2.055
O
O
O
O
1.00
1.09
0.96
2.06
2.10
2.20
5.21
2.19
7.5 7.0 6.5 6.0 5.5 5.0 4.5 4.0 3.5 3.0 2.5 2.0 1.5 1.0 0.5 0.0
f1 (ppm)

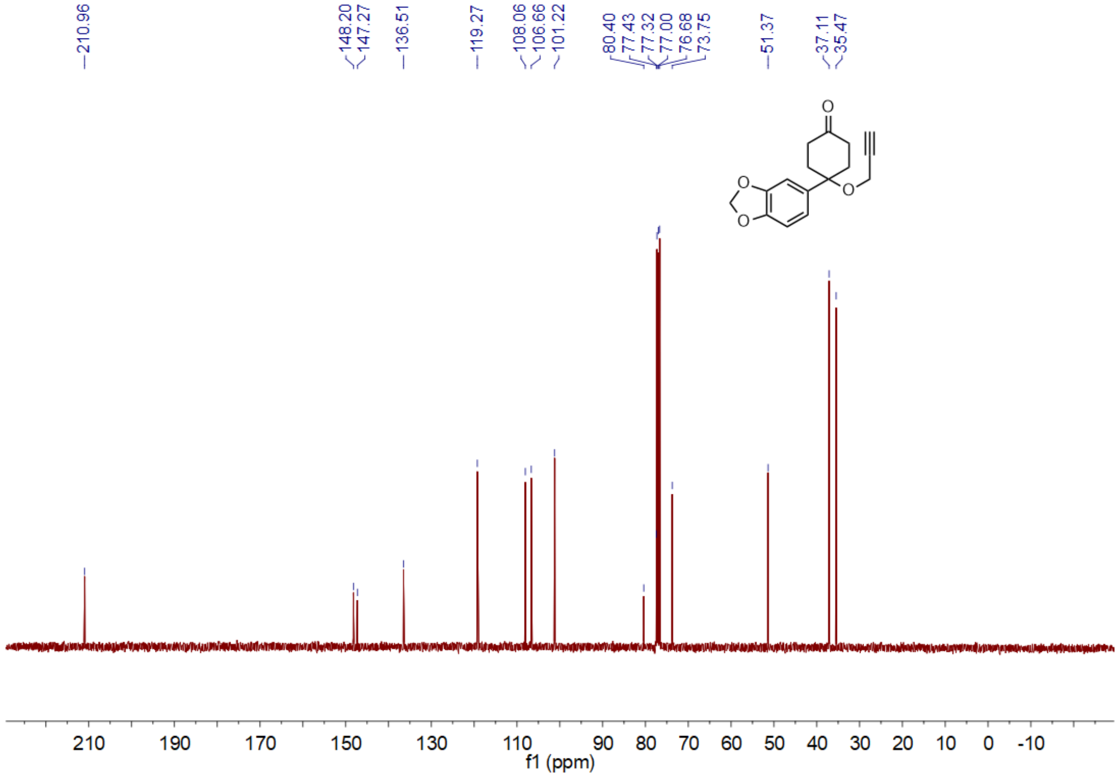
210.96
148.20
147.27
136.51
119.27
108.06
106.66
101.22
80.40
77.43
77.32
77.00
76.68
73.75
51.37
37.11
35.47
O
O
O
O
210 190 170 150 130 110 90 80 70 60 50 40 30 20 10 0 -10
f1 (ppm)

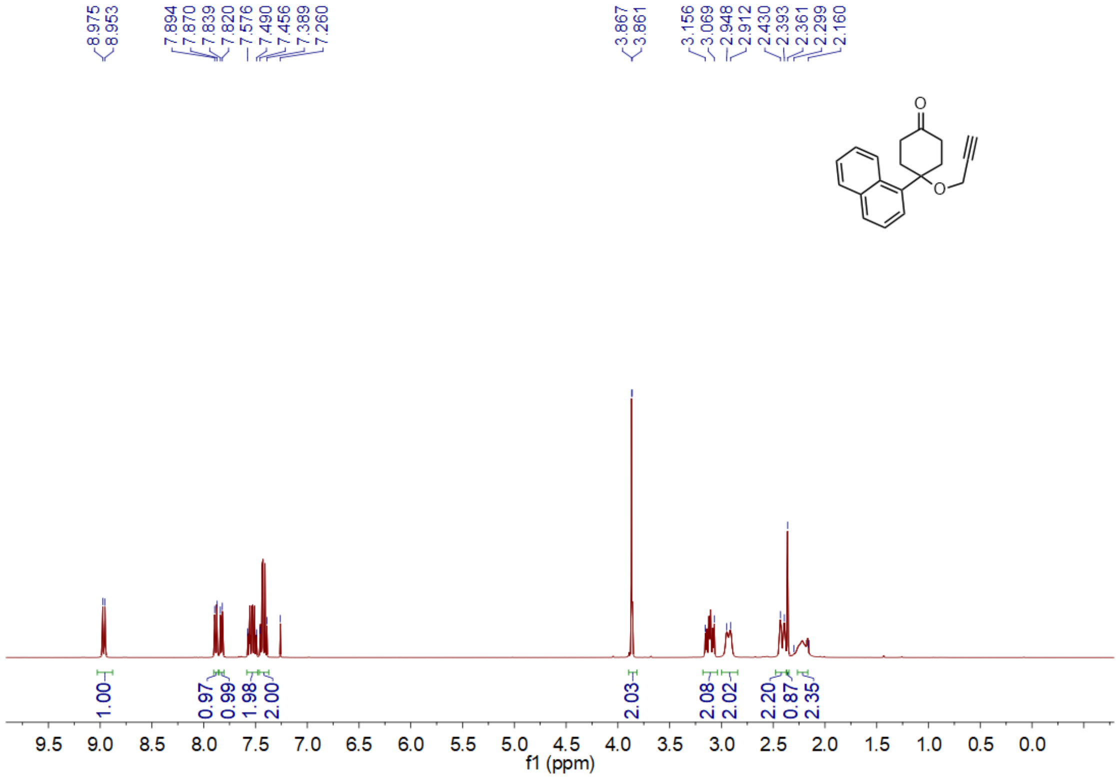
8.975
8.953
7.894
7.870
7.839
7.820
7.576
7.490
7.456
7.389
7.260
3.867
3.861
3.156
3.069
2.948
2.912
2.430
2.393
2.361
2.299
2.160
O
O
1.00
0.97
0.99
1.98
2.00
2.03
2.08
2.02
2.20
0.87
2.35
9.5
9.0
8.5
8.0
7.5
7.0
6.5
6.0
5.5
5.0
4.5
4.0
3.5
3.0
2.5
2.0
1.5
1.0
0.5
0.0
f1 (ppm)

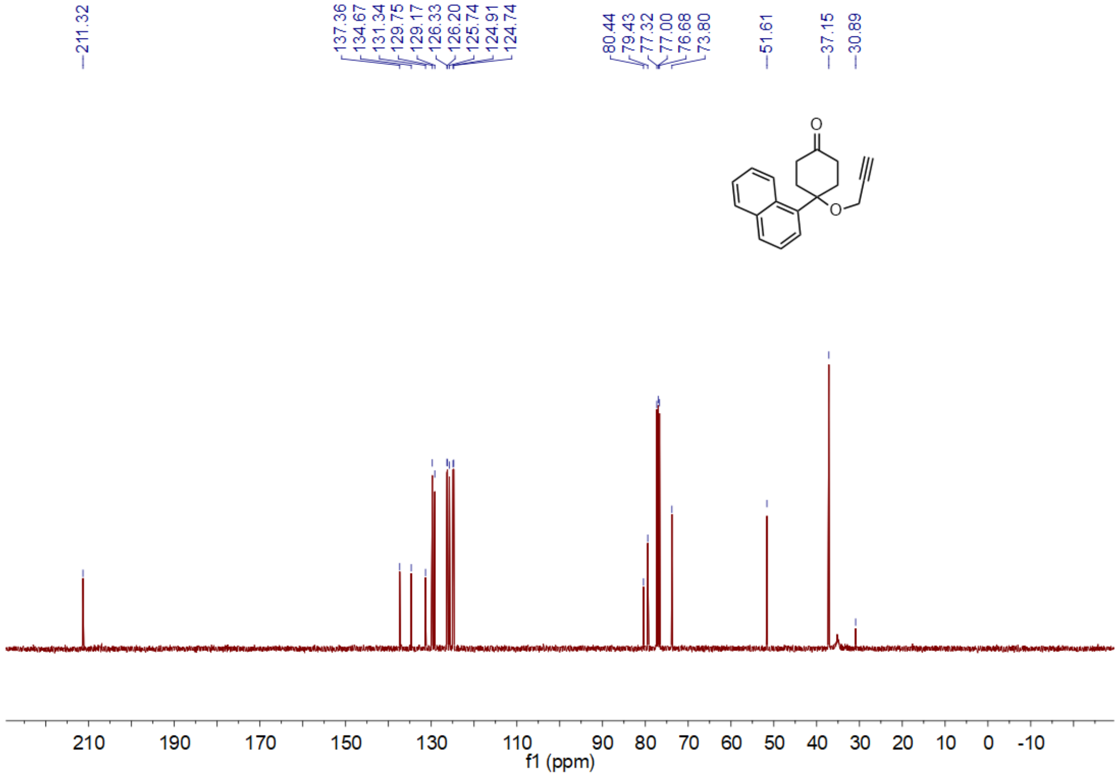
211.32
137.36
134.67
131.34
129.75
129.17
126.33
126.20
125.74
124.91
124.74
80.44
79.43
77.32
77.00
76.68
73.80
51.61
37.15
30.89
O
O
210
190
170
150
130
110
90
80
70
60
50
40
30
20
10
0
-10
f1 (ppm)

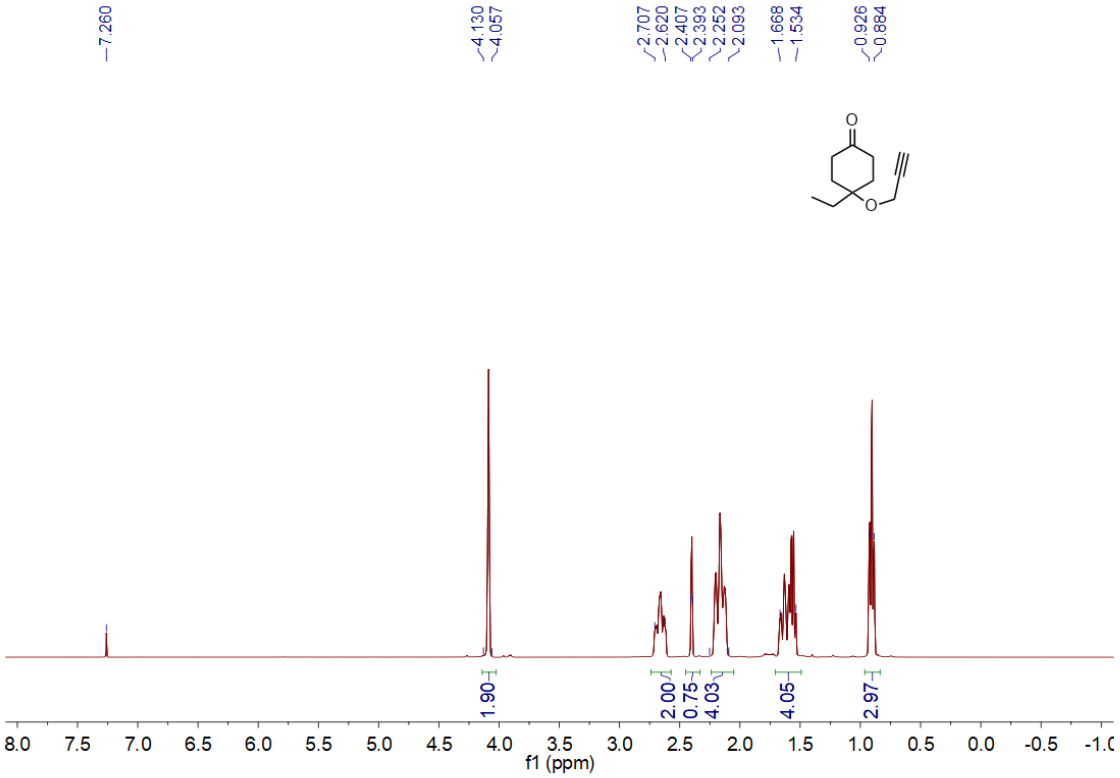
7.260
4.130
4.057
2.707
2.620
2.407
2.393
2.252
2.093
1.668
1.534
0.926
0.884
O
O
1.90
2.00
0.75
4.03
4.05
2.97
8.0 7.5 7.0 6.5 6.0 5.5 5.0 4.5 4.0 3.5 3.0 2.5 2.0 1.5 1.0 0.5 0.0 -0.5
f1 (ppm)

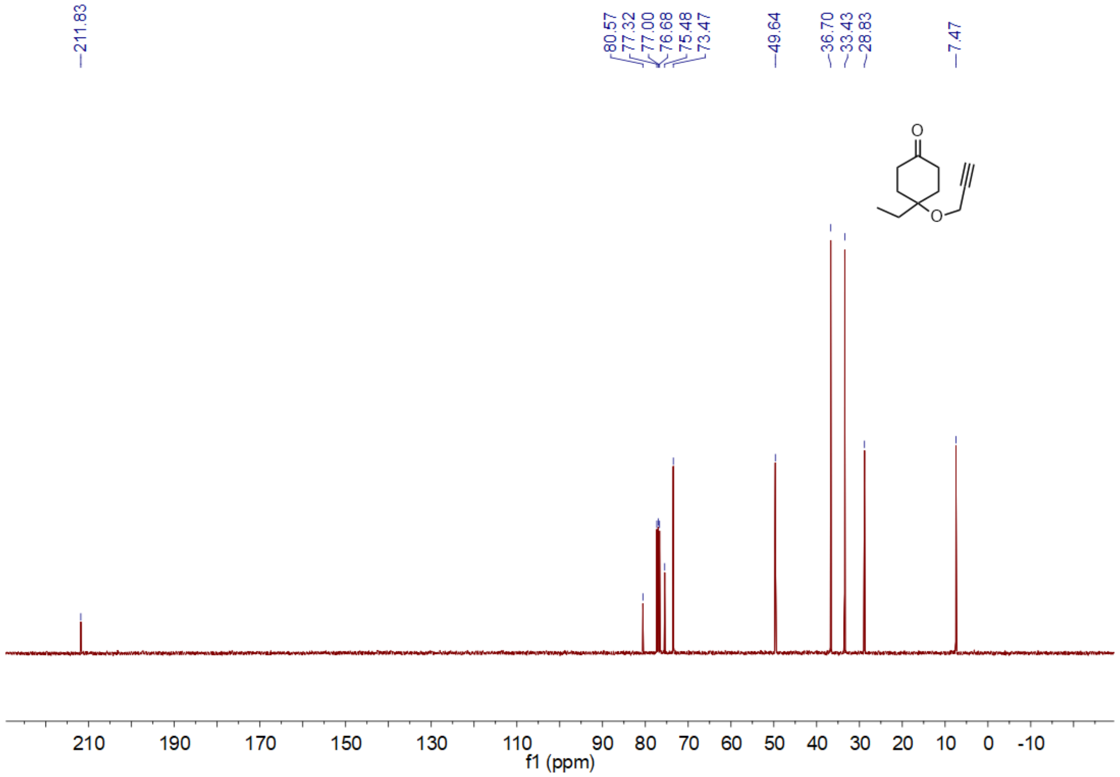
211.83
80.57
77.32
77.00
76.68
75.48
73.47
49.64
36.70
33.43
28.83
7.47
O
O
210 190 170 150 130 110 90 80 70 60 50 40 30 20 10 0 -10
f1 (ppm)

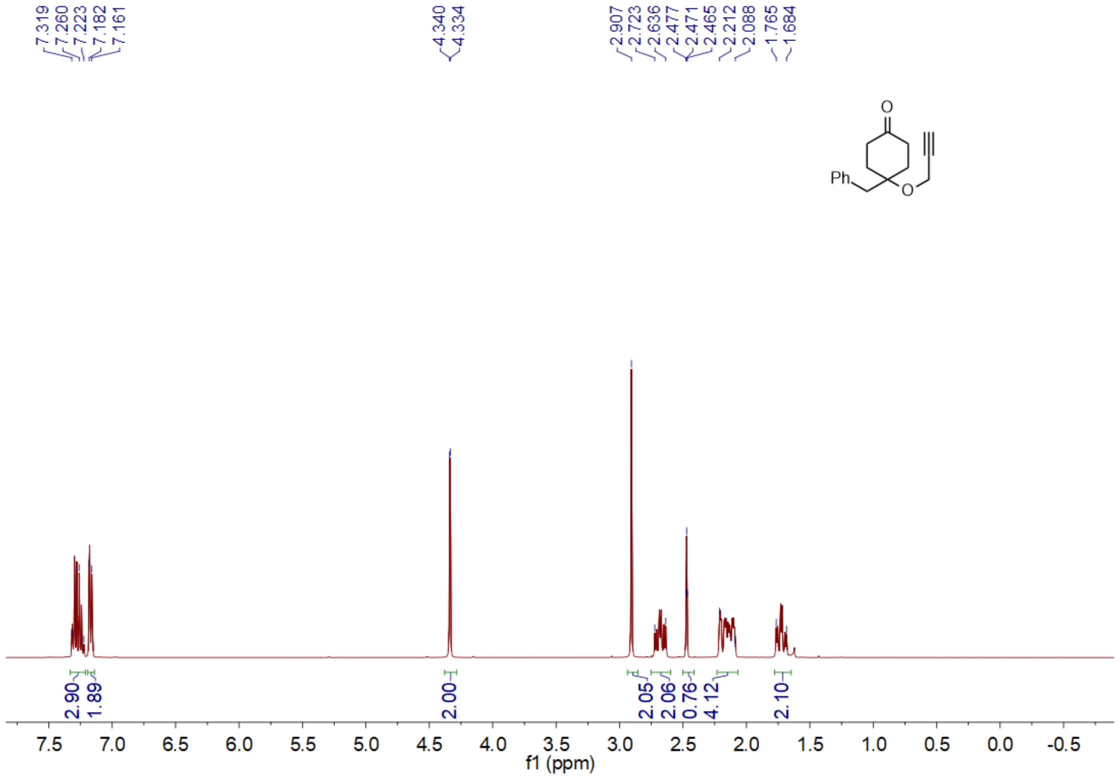
7.319
7.260
7.223
7.182
7.161
4.340
4.334
2.907
2.723
2.636
2.477
2.471
2.465
2.212
2.088
1.765
1.684
O
Ph
O
2.90
1.89
2.00
2.05
2.06
0.76
4.12
2.10
7.5 7.0 6.5 6.0 5.5 5.0 4.5 4.0 3.5 3.0 2.5 2.0 1.5 1.0 0.5 0.0 -0.5
f1 (ppm)

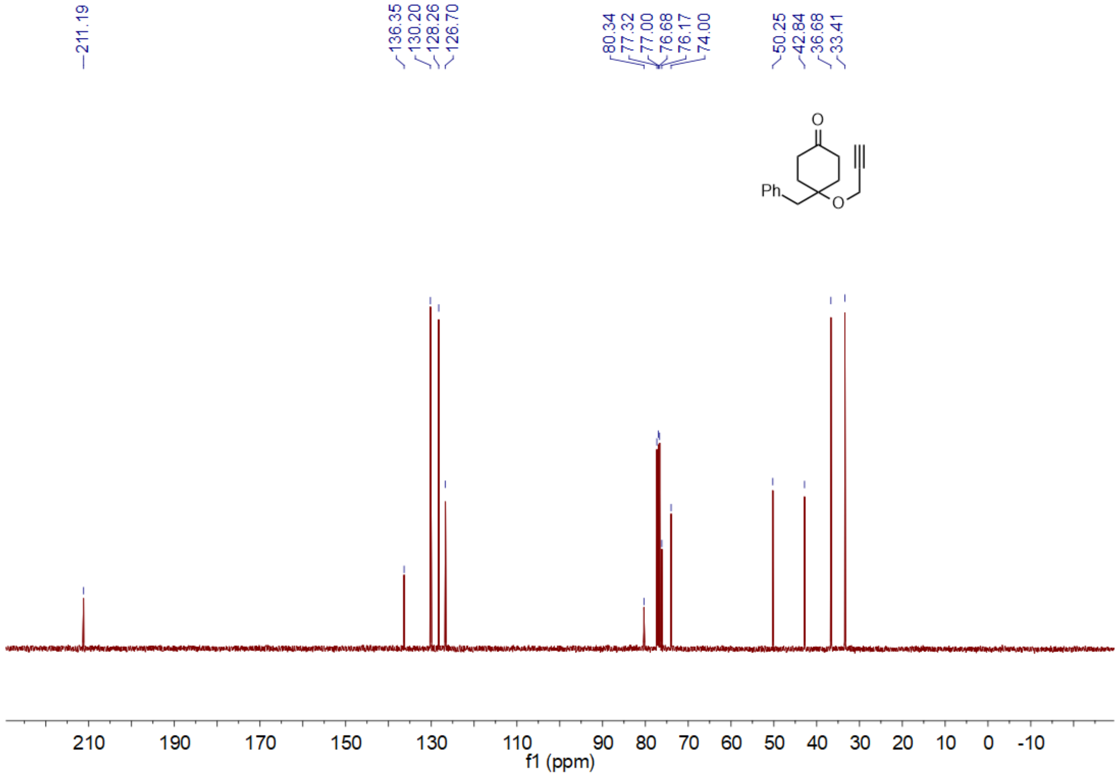
211.19
136.35
130.20
128.26
126.70
80.34
77.32
77.00
76.68
76.17
74.00
50.25
42.84
36.68
33.41
O
Ph
O
210 190 170 150 130 110 90 80 70 60 50 40 30 20 10 0 -10
f1 (ppm)

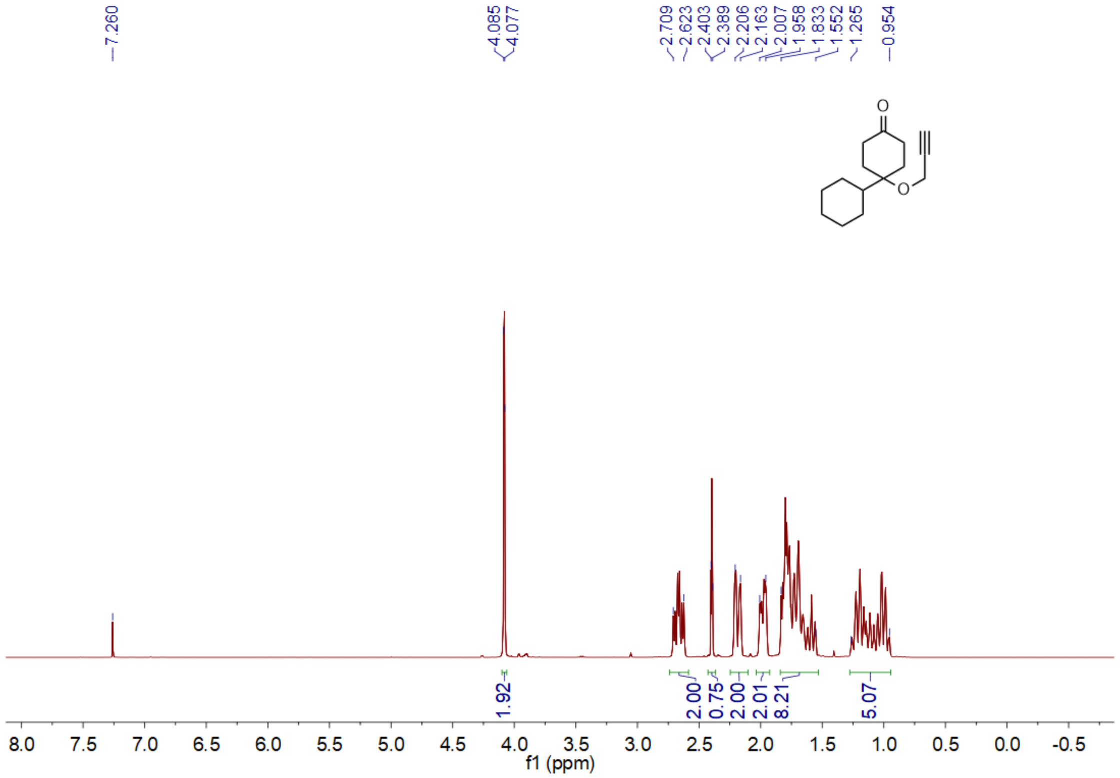

7.260
4.085
4.077
2.709
2.623
2.403
2.389
2.206
2.163
2.007
1.958
1.833
1.552
1.265
0.954
O
O
1.92
2.00
0.75
2.00
2.01
8.21
5.07
8.0 7.5 7.0 6.5 6.0 5.5 5.0 4.5 4.0 3.5 3.0 2.5 2.0 1.5 1.0 0.5 0.0 -0.5
f1 (ppm)

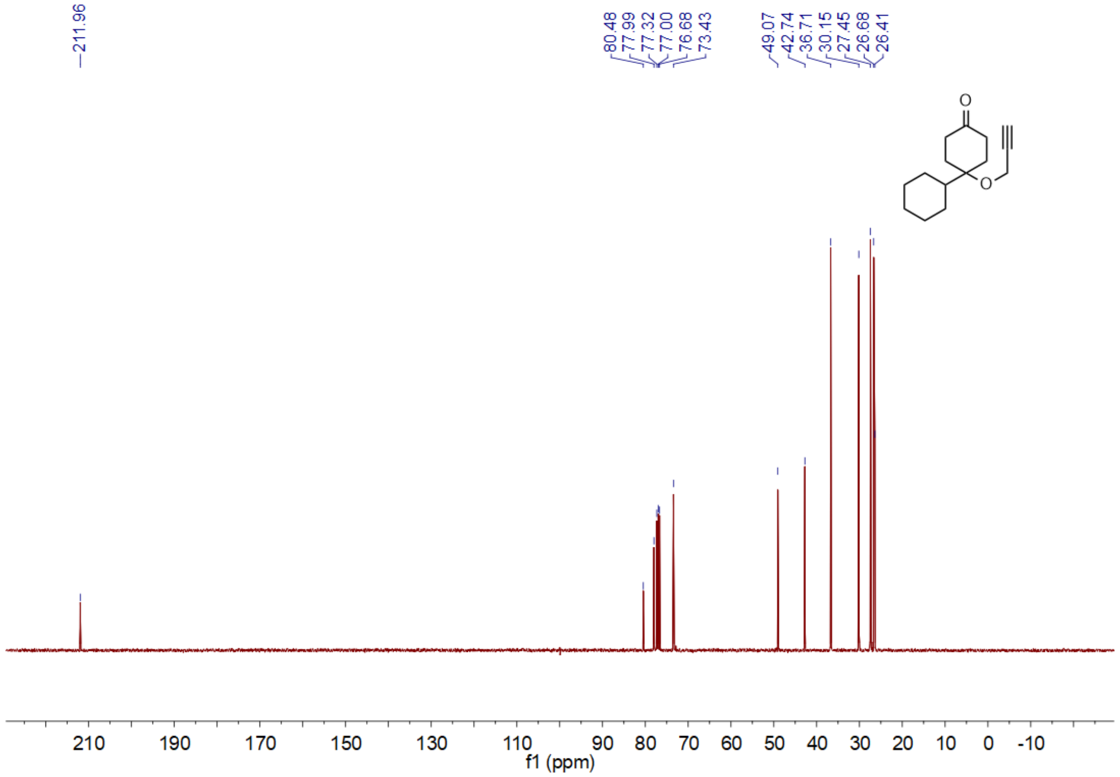

211.96
80.48
77.99
77.32
77.00
76.68
73.43
49.07
42.74
36.71
30.15
27.45
26.68
26.41
O
O
210 190 170 150 130 110 90 80 70 60 50 40 30 20 10 0 -10
f1 (ppm)

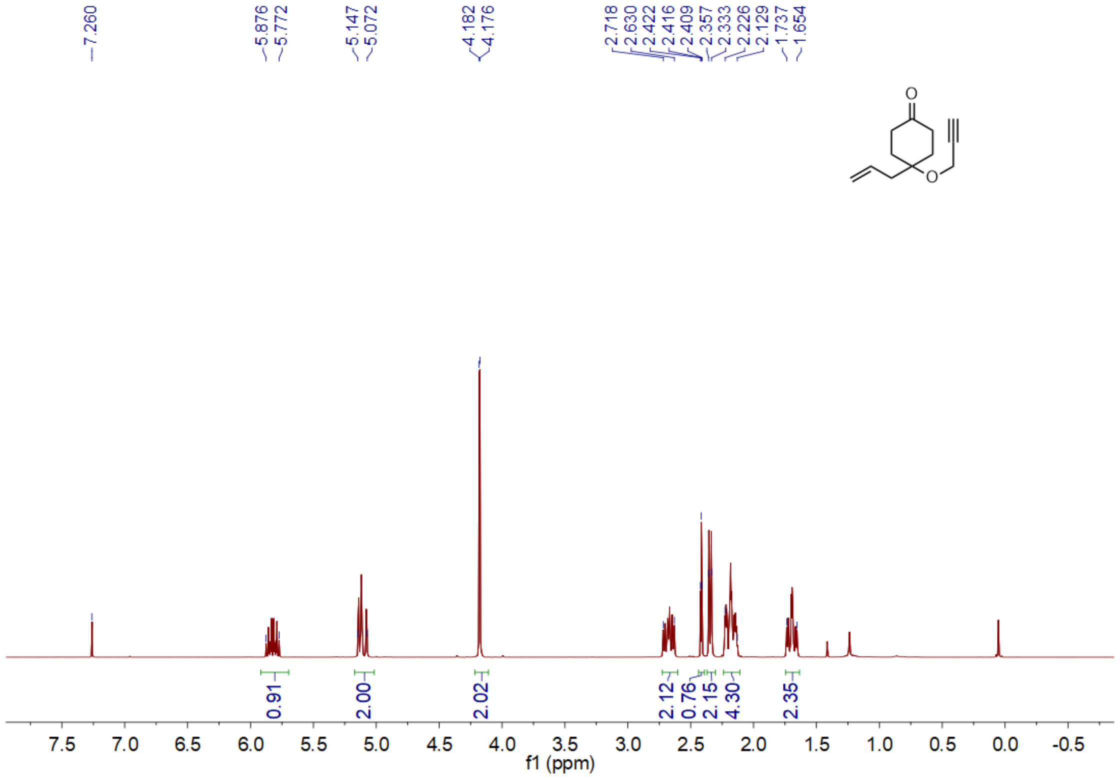
7.260
5.876
5.772
5.147
5.072
4.182
4.176
2.718
2.630
2.422
2.416
2.409
2.357
2.333
2.226
2.129
1.737
1.654
O
O
0.91
2.00
2.02
2.12
0.76
2.15
4.30
2.35
7.5 7.0 6.5 6.0 5.5 5.0 4.5 4.0 3.5 3.0 2.5 2.0 1.5 1.0 0.5 0.0 -0.5
f1 (ppm)

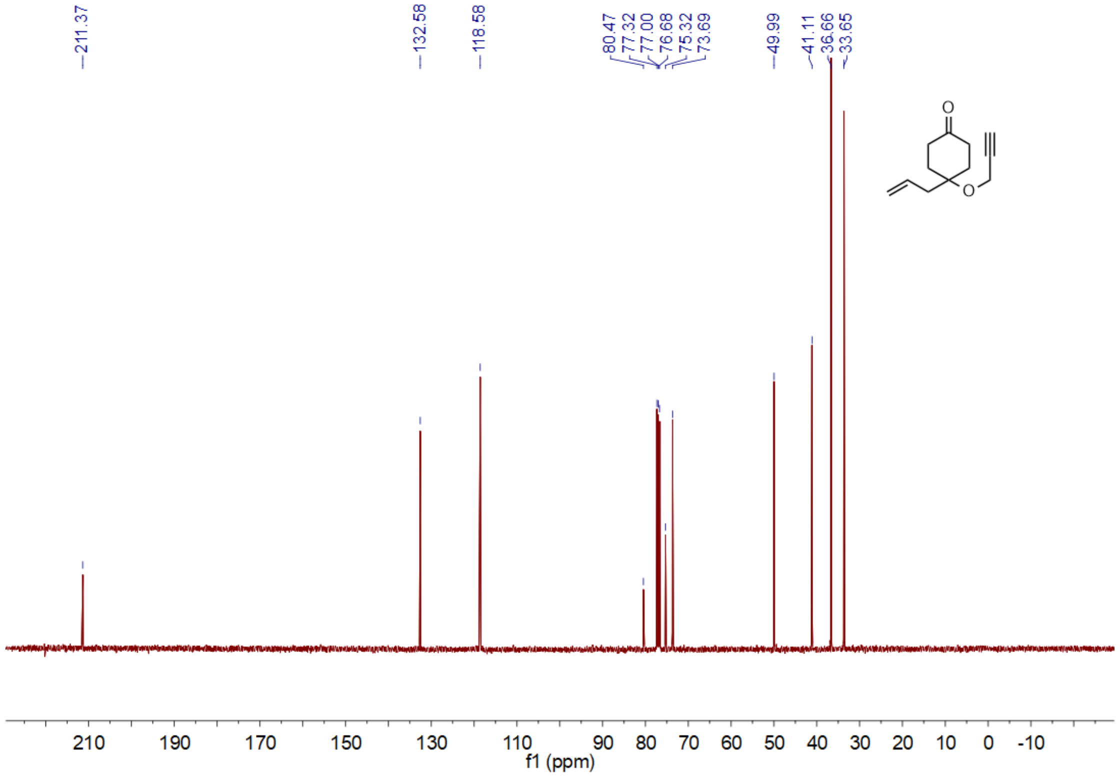
211.37
132.58
118.58
80.47
77.32
77.00
76.68
75.32
73.69
49.99
41.11
36.66
33.65
O
O
210 190 170 150 130 110 90 80 70 60 50 40 30 20 10 0 -10
f1 (ppm)

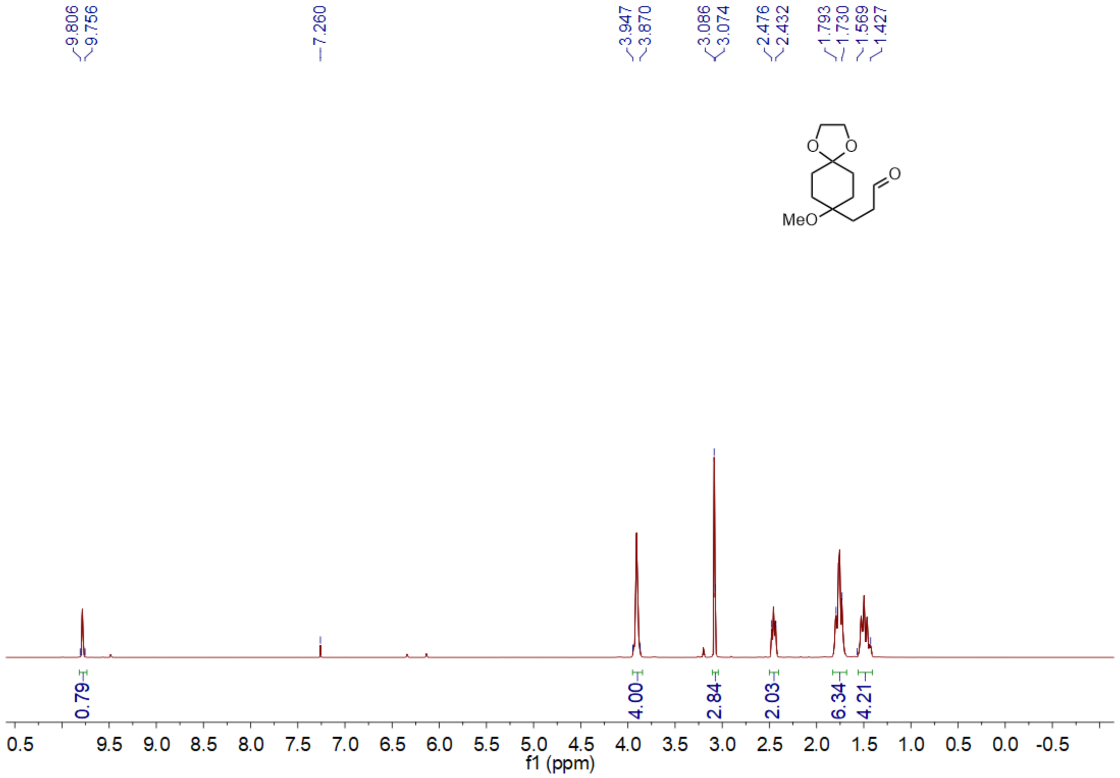
9.806
9.756
7.260
3.947
3.870
3.086
3.074
2.476
2.432
1.793
1.730
1.569
1.427
O
O
O
MeO
0.79
4.00
2.84
2.03
6.34
4.21
0.5 9.5 9.0 8.5 8.0 7.5 7.0 6.5 6.0 5.5 5.0 4.5 4.0 3.5 3.0 2.5 2.0 1.5 1.0 0.5 0.0 -0.5
f1 (ppm)

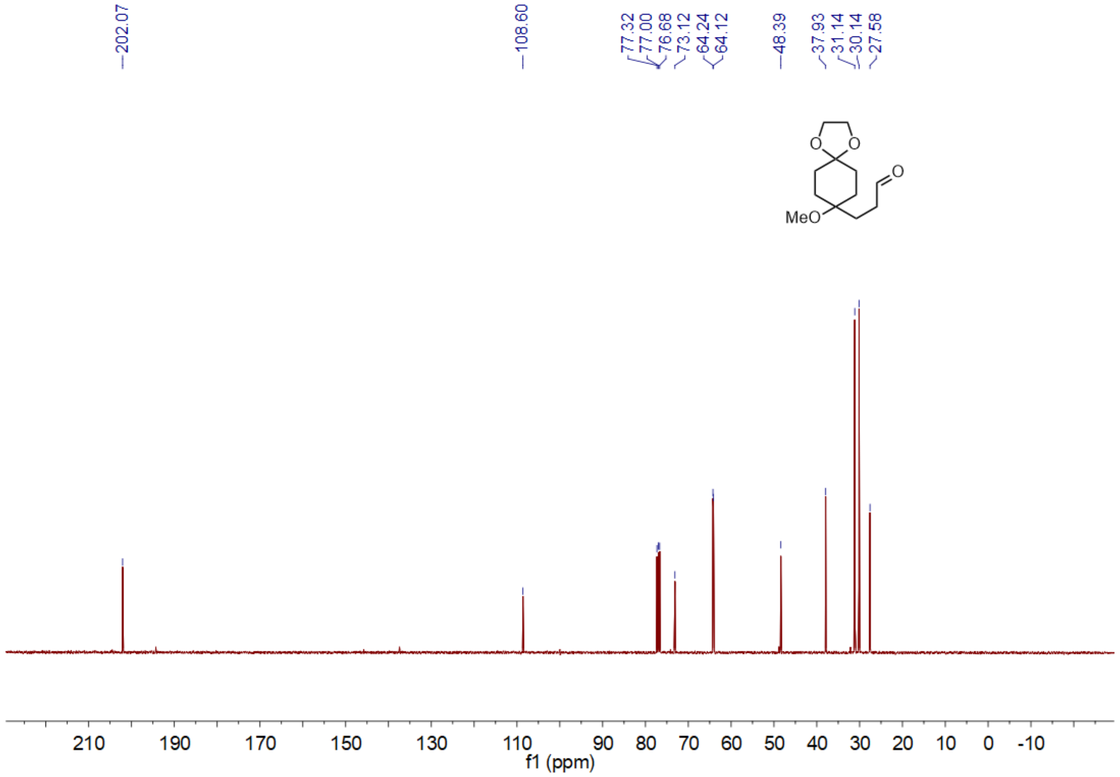
202.07
108.60
77.32
77.00
76.68
73.12
64.24
64.12
48.39
37.93
31.14
30.14
27.58
O
O
O
MeO
210 190 170 150 130 110 90 80 70 60 50 40 30 20 10 0 -10
f1 (ppm)

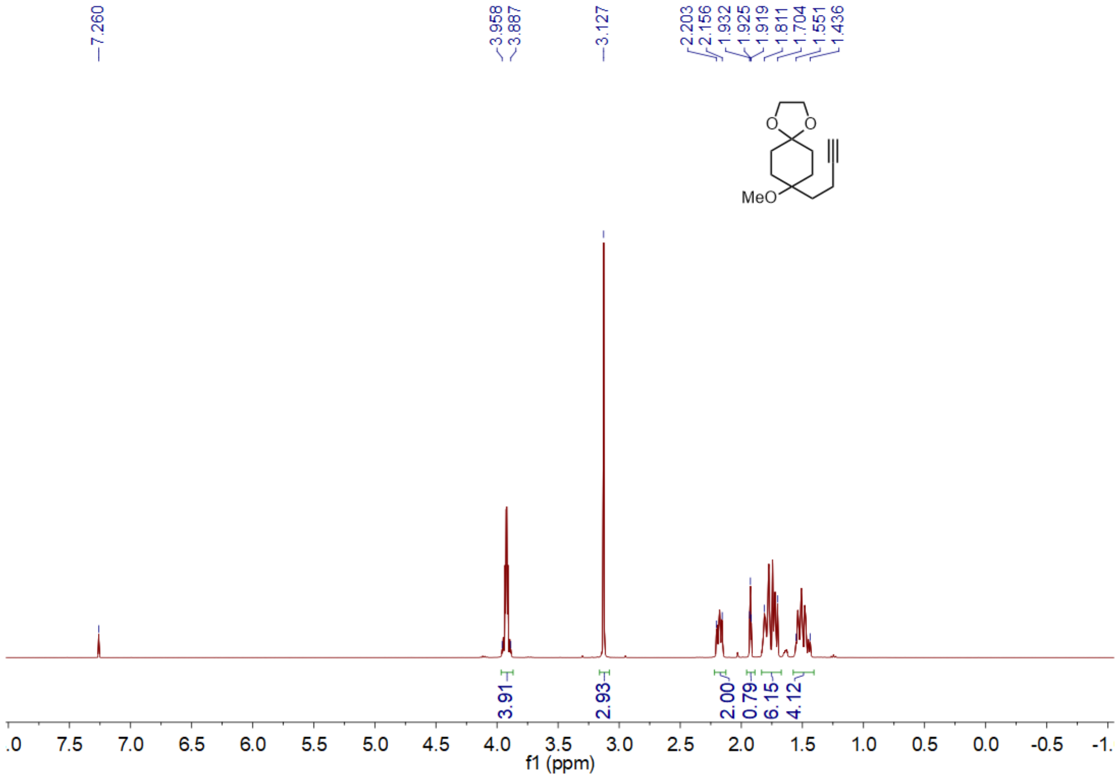
7.260
3.958
3.887
3.127
2.203
2.156
1.932
1.925
1.919
1.811
1.704
1.551
1.436
MeO
O
O
3.91
2.93
2.00
0.79
6.15
4.12
f1 (ppm)

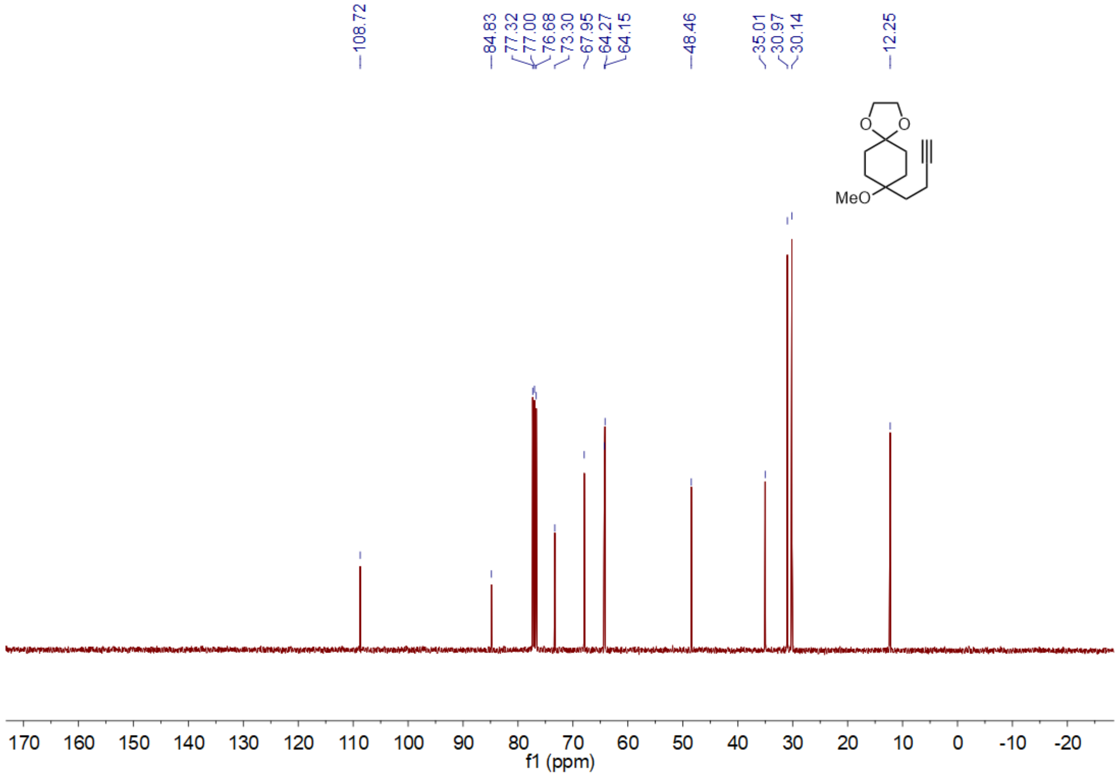
108.72
84.83
77.32
77.00
76.68
73.30
67.95
64.27
64.15
48.46
35.01
30.97
30.14
12.25
MeO
O
O
f1 (ppm)

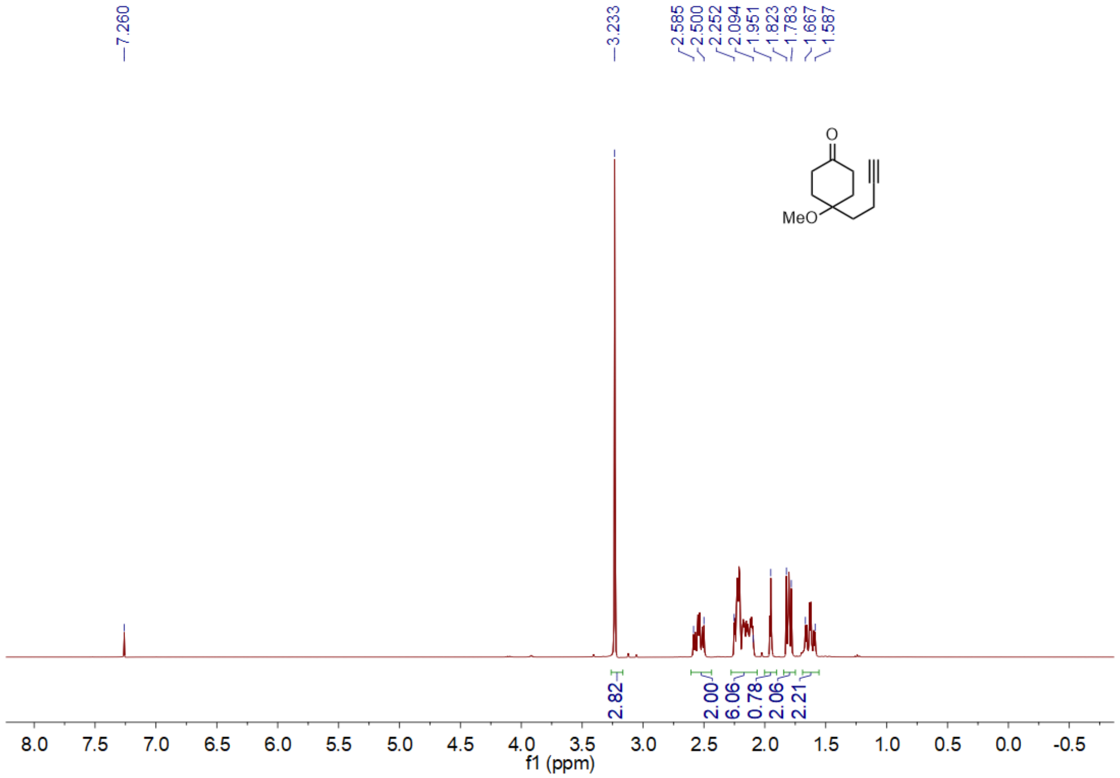
7.260
3.233
2.585
2.500
2.252
2.094
1.951
1.823
1.783
1.667
1.587
O
MeO
2.82
2.00
6.06
0.78
2.06
2.21
8.0 7.5 7.0 6.5 6.0 5.5 5.0 4.5 4.0 3.5 3.0 2.5 2.0 1.5 1.0 0.5 0.0 -0.5
f1 (ppm)

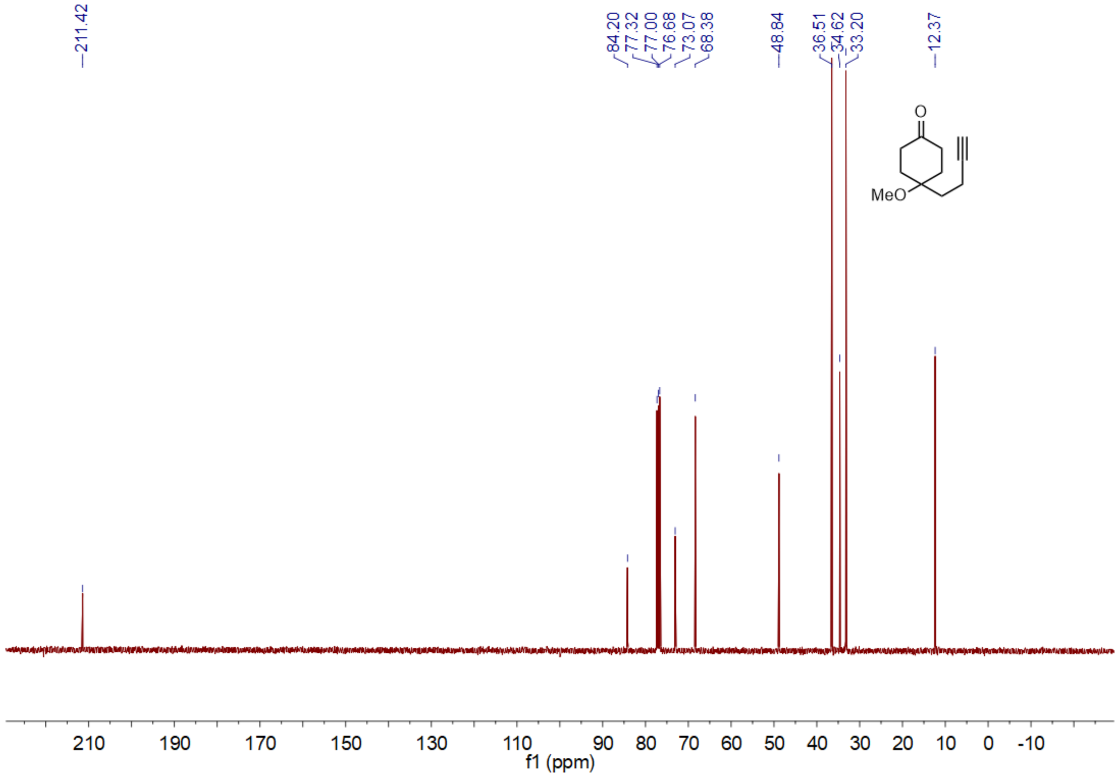
211.42
84.20
77.32
77.00
76.68
73.07
68.38
48.84
36.51
34.62
33.20
12.37
O
MeO
210 190 170 150 130 110 90 80 70 60 50 40 30 20 10 0 -10
f1 (ppm)
